# Supplementary material for: Pan-cancer analyses of the associations between 109 pre-existing conditions and cancer treatment patterns across 19 adult cancers
Source: Sci Rep. 2024 Jan 3;14:464. doi: 10.1038/s41598-024-51161-0 (PMC10764847; doi:10.1038/s41598-024-51161-0)

Supplementary figures

Pan-cancer analyses of the associations between 109 pre-existing conditions and cancer treatment patterns across 19 adult cancers

Supplementary figure legends

Figure S1. Non-cancer conditions diagnosed before cancer. Boxplots show the proportion of patients having a first diagnosis of any of the 109 non-cancer conditions (grouped into 9 organ systems) in the 5-year period before cancer diagnosis. Boxplots for 11 cancer types are shown in this figure. Conditions with proportions ≥ 5% are annotated on the plots. Boxplots for the remaining 8 cancer types are shown in Figure 1. Full data and confidence intervals are presented in Table S6.

Figure S2. Multinomial logistic regression investigating the associations between endocrine conditions and cancer treatment decisions. Forest plots show odds ratios for a particular treatment type, adjusted for age, sex, socioeconomic status, tumour grade, tumour stage, tumour count and multimorbidity count. Seven treatment categories were considered (i) surgery alone, (ii) chemotherapy alone, (iii) radiotherapy alone, (iv) chemotherapy and radiotherapy, (v) chemotherapy and surgery, (vi) radiotherapy and surgery, and (vii) chemotherapy, radiotherapy and surgery. Multinomial logistic regression models were fitted using surgery alone as the baseline choice of treatment for each cancer type (colour-coded). Only results with $P < 0.05$ are shown in the figure. P values are annotated on the plots. Full data and confidence intervals are presented in Table S8.

Figure S3. Multinomial logistic regression investigating the associations between gastrointestinal conditions and cancer treatment decisions. Forest plots show odds ratios for a particular treatment type, adjusted for age, sex, socioeconomic status, tumour grade, tumour stage, tumour count and multimorbidity count. Seven treatment categories were considered (i) surgery alone, (ii) chemotherapy alone, (iii) radiotherapy alone, (iv) chemotherapy and radiotherapy, (v) chemotherapy and surgery, (vi) radiotherapy and surgery, and (vii) chemotherapy, radiotherapy and surgery. Multinomial logistic regression models were fitted using surgery alone as the baseline choice of treatment for each cancer type (colour-coded). Only results with $P < 0.05$ are shown in the figure. P values are annotated on the plots. Full data and confidence intervals are presented in Table S8.

Figure S4. Multinomial logistic regression investigating the associations between haematological conditions and cancer treatment decisions. Forest plots show odds ratios for a particular treatment type, adjusted for age, sex, socioeconomic status, tumour grade, tumour stage, tumour count and multimorbidity count. Seven treatment categories were considered (i) surgery alone, (ii) chemotherapy alone, (iii) radiotherapy alone, (iv) chemotherapy and radiotherapy, (v) chemotherapy and surgery, (vi) radiotherapy and surgery, and (vii) chemotherapy, radiotherapy and surgery. Multinomial logistic regression models were fitted using surgery alone as the baseline choice of treatment for each cancer type (colour-coded). Only results with $P < 0.05$ are shown in the figure. P values are annotated on the plots. Full data and confidence intervals are presented in Table S8.

Figure S5. Multinomial logistic regression investigating the associations between immunological conditions and infections and cancer treatment decisions. Forest plots show odds ratios for a particular treatment type, adjusted for age, sex, socioeconomic status, tumour grade, tumour stage, tumour count and multimorbidity count. Seven treatment categories were considered (i) surgery alone, (ii) chemotherapy alone, (iii) radiotherapy alone, (iv) chemotherapy and radiotherapy, (v) chemotherapy and surgery, (vi) radiotherapy and surgery, and (vii) chemotherapy, radiotherapy and surgery. Multinomial logistic regression models were fitted using surgery alone as the baseline choice of treatment for each cancer type (colour-coded). Only results with $P < 0.05$ are shown in the figure. P values are annotated on the plots. Full data and confidence intervals are presented in Table S8.

Figure S6. Multinomial logistic regression investigating the associations between musculoskeletal conditions and cancer treatment decisions. Forest plots show odds ratios for a particular treatment type, adjusted for age, sex, socioeconomic status, tumour grade, tumour stage, tumour count and multimorbidity count. Seven treatment categories were considered (i) surgery alone, (ii) chemotherapy alone, (iii) radiotherapy alone, (iv) chemotherapy and radiotherapy, (v) chemotherapy and surgery, (vi) radiotherapy and surgery, and (vii) chemotherapy, radiotherapy and surgery. Multinomial logistic regression models were fitted using surgery alone as the baseline choice of treatment for each cancer type (colour-coded). Only results with $P < 0.05$ are shown in the figure. P values are annotated on the plots. Full data and confidence intervals are presented in Table S8.

Figure S7. Multinomial logistic regression investigating the associations between neurological conditions and cancer treatment decisions. Forest plots show odds ratios for a particular treatment type, adjusted for age, sex, socioeconomic status, tumour grade, tumour stage, tumour count and multimorbidity count. Seven treatment categories were considered (i) surgery alone, (ii) chemotherapy alone, (iii) radiotherapy alone, (iv) chemotherapy and radiotherapy, (v) chemotherapy and surgery, (vi) radiotherapy and surgery, and (vii) chemotherapy, radiotherapy and surgery. Multinomial logistic regression models were fitted using surgery alone as the baseline choice of treatment for each cancer type (colour-coded). Only results with $P < 0.05$ are shown in the figure. P values are annotated on the plots. Full data and confidence intervals are presented in Table S8.

Figure S8. Multinomial logistic regression investigating the associations between pulmonary conditions and cancer treatment decisions. Forest plots show odds ratios for a particular treatment type, adjusted for age, sex, socioeconomic status, tumour grade, tumour stage, tumour count and multimorbidity count. Seven treatment categories were considered (i) surgery alone, (ii) chemotherapy alone, (iii) radiotherapy alone, (iv) chemotherapy and radiotherapy, (v) chemotherapy and surgery, (vi) radiotherapy and surgery, and (vii) chemotherapy, radiotherapy and surgery. Multinomial logistic regression models were fitted using surgery alone as the baseline choice of treatment for each cancer type (colour-coded). Only results with $P < 0.05$ are shown in the figure. P values are annotated on the plots. Full data and confidence intervals are presented in Table S8.

Figure S9. Multinomial logistic regression investigating the associations between renal conditions and cancer treatment decisions. Forest plots show odds ratios for a particular treatment type, adjusted for age, sex, socioeconomic status, tumour grade, tumour stage, tumour count and multimorbidity count. Seven treatment categories were considered (i) surgery alone, (ii) chemotherapy alone, (iii) radiotherapy alone, (iv) chemotherapy and radiotherapy, (v) chemotherapy and surgery, (vi) radiotherapy and surgery, and (vii)

chemotherapy, radiotherapy and surgery. Multinomial logistic regression models were fitted using surgery alone as the baseline choice of treatment for each cancer type (colour-coded). Only results with $P < 0.05$ are shown in the figure. P values are annotated on the plots. Full data and confidence intervals are presented in Table S8.

Figure S10. Binomial logistic regression investigating the associations between comorbidity and chemotherapy decisions. Forest plots show odds ratios for a particular chemotherapy type, adjusted for cancer type, age, sex, socioeconomic status, tumour grade, tumour stage, tumour count and multimorbidity count. Ten chemotherapy classes were considered – results for 8 classes are shown in this figure. Plots for the remaining 2 chemotherapy classes are shown in Figure 4. Binomial logistic regression models were fitted. Conditions were colour-coded according to the 9 organ systems. Only results with $P < 0.05$ are shown in the figure. P values are annotated on the plots. Full data and confidence intervals are presented in Table S9.

Figure S1. Non-cancer conditions diagnosed before cancer. Boxplots show the proportion of patients having a first diagnosis of any of the 109 non-cancer conditions (grouped into 9 organ systems) in the 5-year period before cancer diagnosis. Boxplots for 11 cancer types are shown in this figure. Conditions with proportions ≥ 5% are annotated on the plots. Boxplots for the remaining 8 cancer types are shown in Figure 1. Full data and confidence intervals are presented in Table S6.

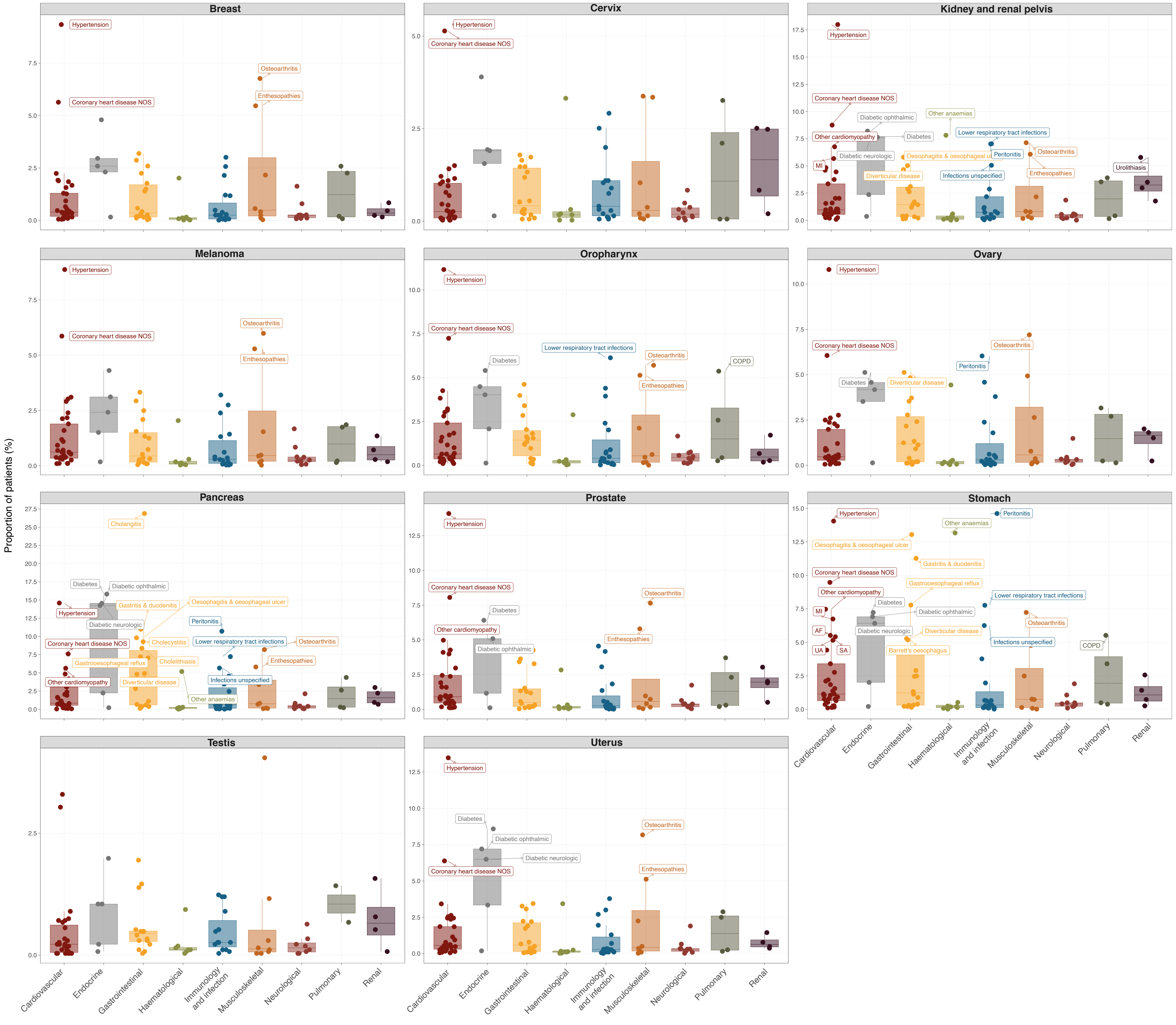

Figure S2. Multinomial logistic regression investigating the associations between endocrine conditions and cancer treatment decisions. Forest plots show odds ratios for a particular treatment type, adjusted for age, sex, socioeconomic status, tumour grade, tumour stage, tumour count and multimorbidity count. Seven treatment categories were considered (i) surgery alone, (ii) chemotherapy alone, (iii) radiotherapy alone, (iv) chemotherapy and radiotherapy, (v) chemotherapy and surgery, (vi) radiotherapy and surgery, and (vii) chemotherapy, radiotherapy and surgery. Multinomial logistic regression models were fitted using surgery alone as the baseline choice of treatment for each cancer type (colour-coded). Only results with P < 0.05 are shown in the figure. P values are annotated on the plots. Full data and confidence intervals are presented in Table S8.

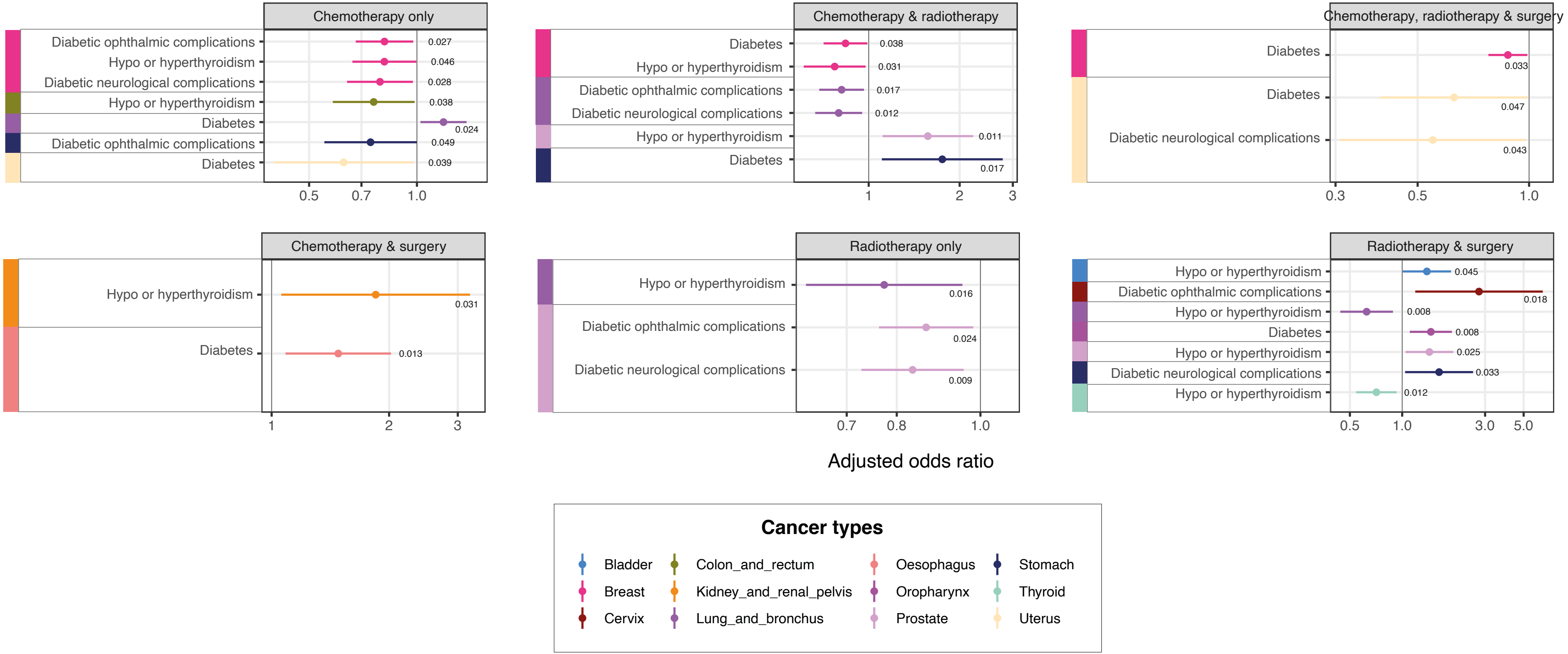

Figure S3. Multinomial logistic regression investigating the associations between gastrointestinal conditions and cancer treatment decisions. Forest plots show odds ratios for a particular treatment type, adjusted for age, sex, socioeconomic status, tumour grade, tumour stage, tumour count and multimorbidity count. Seven treatment categories were considered (i) surgery alone, (ii) chemotherapy alone, (iii) radiotherapy alone, (iv) chemotherapy and radiotherapy, (v) chemotherapy and surgery, (vi) radiotherapy and surgery, and (vii) chemotherapy, radiotherapy and surgery. Multinomial logistic regression models were fitted using surgery alone as the baseline choice of treatment for each cancer type (colour-coded). Only results with P < 0.05 are shown in the figure. P values are annotated on the plots. Full data and confidence intervals are presented in Table S8.

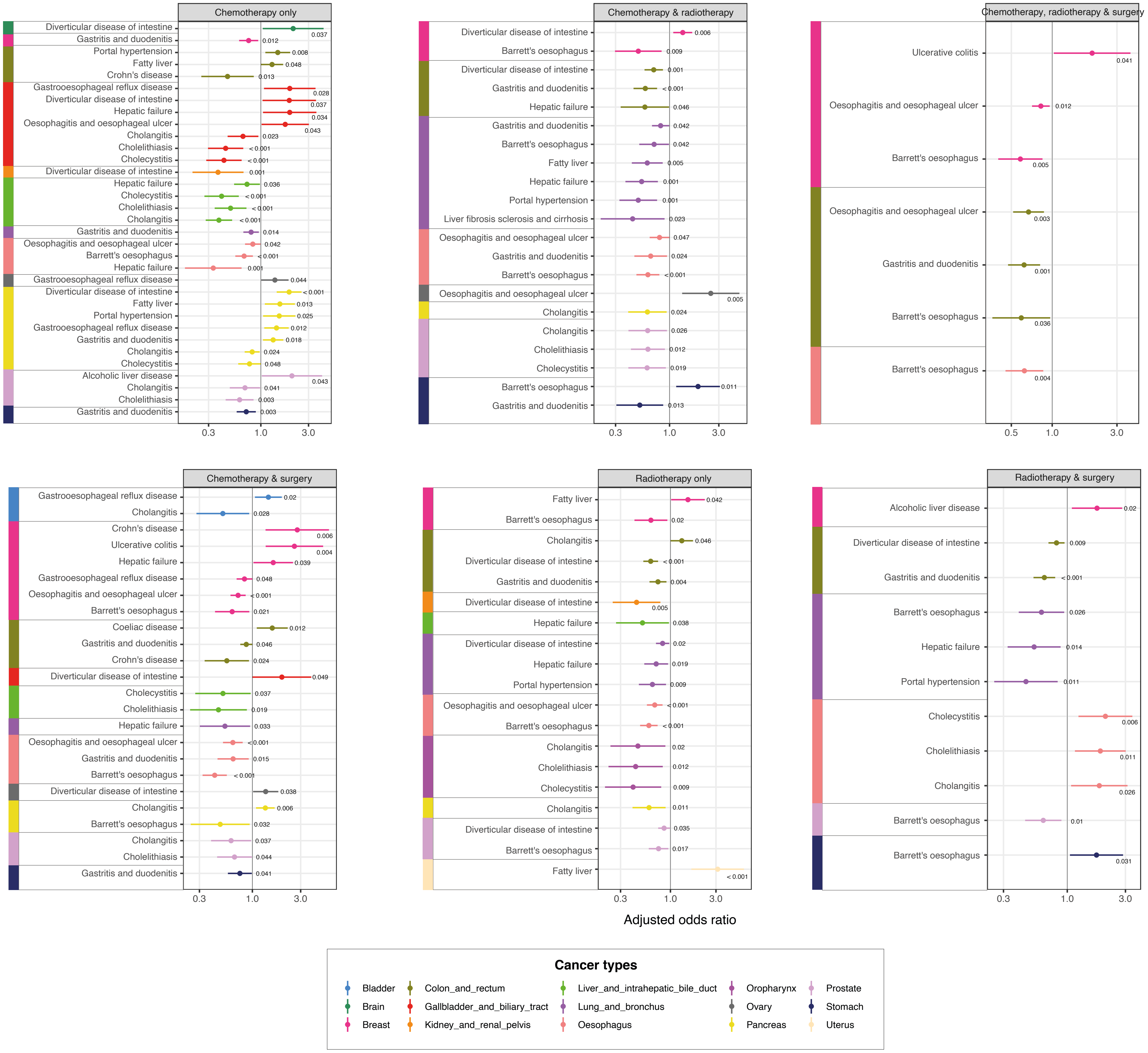

Figure S4. Multinomial logistic regression investigating the associations between haematological conditions and cancer treatment decisions. Forest plots show odds ratios for a particular treatment type, adjusted for age, sex, socioeconomic status, tumour grade, tumour stage, tumour count and multimorbidity count. Seven treatment categories were considered (i) surgery alone, (ii) chemotherapy alone, (iii) radiotherapy alone, (iv) chemotherapy and radiotherapy, (v) chemotherapy and surgery, (vi) radiotherapy and surgery, and (vii) chemotherapy, radiotherapy and surgery. Multinomial logistic regression models were fitted using surgery alone as the baseline choice of treatment for each cancer type (colour-coded). Only results with $P < 0.05$ are shown in the figure. P values are annotated on the plots. Full data and confidence intervals are presented in Table S8.

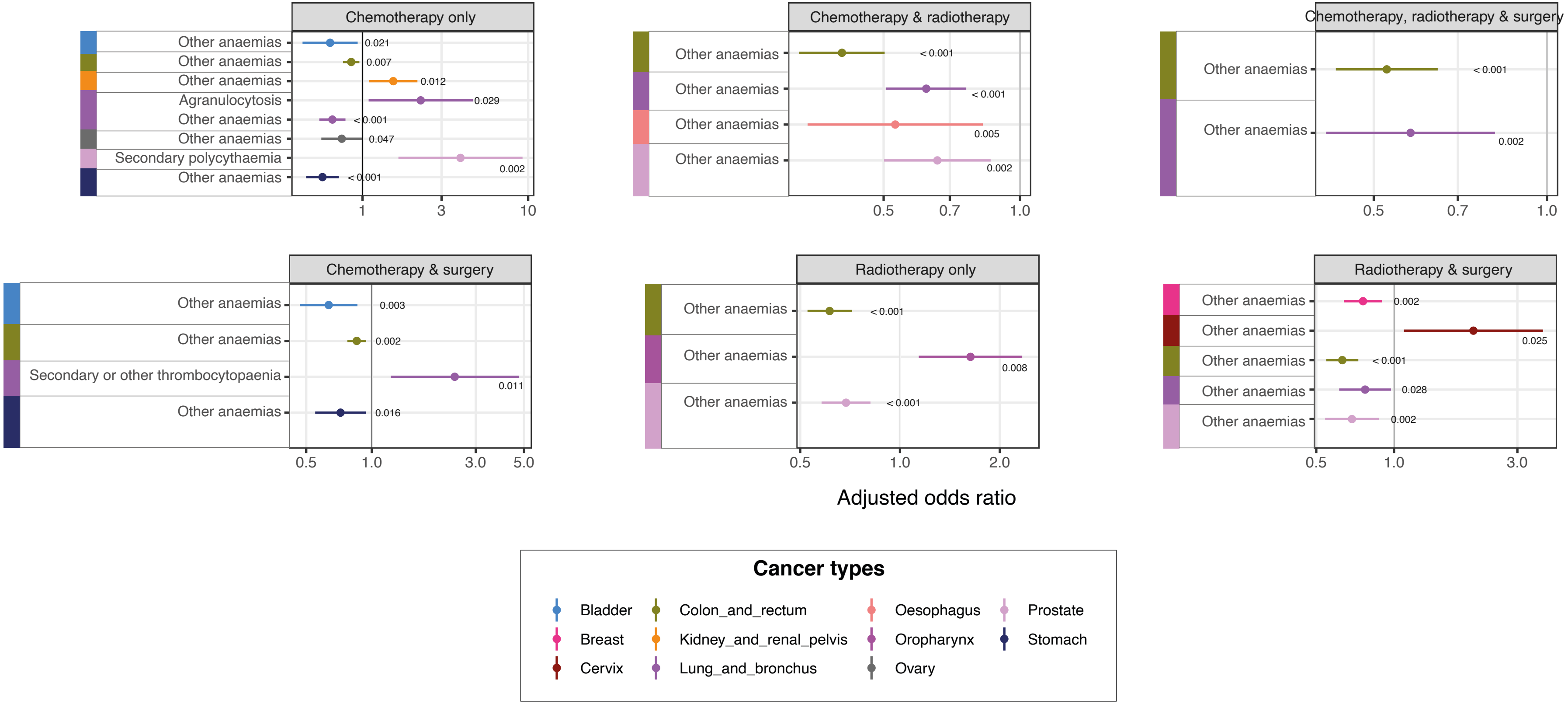

Figure S5. Multinomial logistic regression investigating the associations between immunological conditions and infections and cancer treatment decisions. Forest plots show odds ratios for a particular treatment type, adjusted for age, sex, socioeconomic status, tumour grade, tumour stage, tumour count and multimorbidity count. Seven treatment categories were considered (i) surgery alone, (ii) chemotherapy alone, (iii) radiotherapy alone, (iv) chemotherapy and radiotherapy, (v) chemotherapy and surgery, (vi) radiotherapy and surgery, and (vii) chemotherapy, radiotherapy and surgery. Multinomial logistic regression models were fitted using surgery alone as the baseline choice of treatment for each cancer type (colour-coded). Only results with P < 0.05 are shown in the figure. P values are annotated on the plots. Full data and confidence intervals are presented in Table S8.

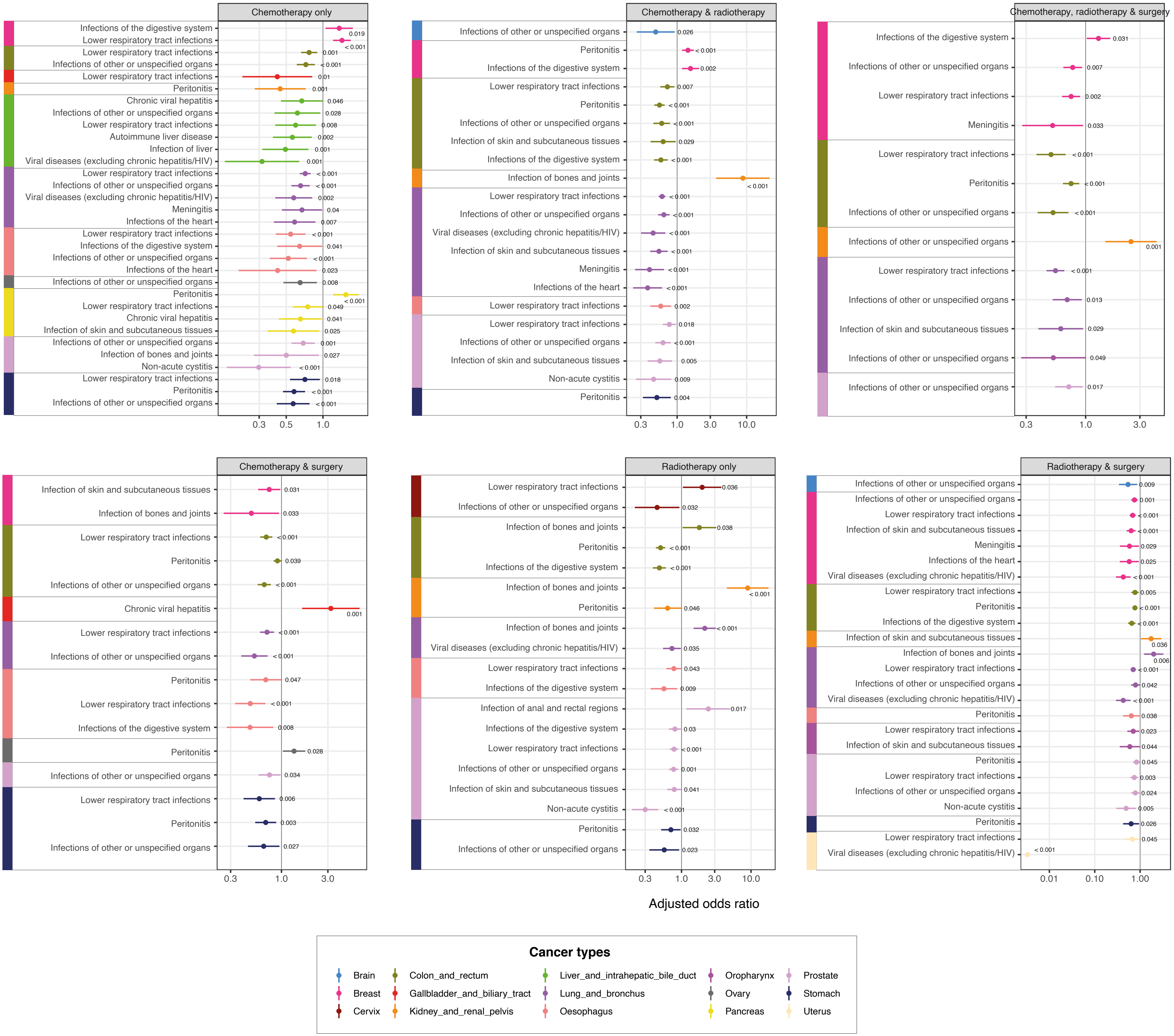

Figure S6. Multinomial logistic regression investigating the associations between musculoskeletal conditions and cancer treatment decisions. Forest plots show odds ratios for a particular treatment type, adjusted for age, sex, socioeconomic status, tumour grade, tumour stage, tumour count and multimorbidity count. Seven treatment categories were considered (i) surgery alone, (ii) chemotherapy alone, (iii) radiotherapy alone, (iv) chemotherapy and radiotherapy, (v) chemotherapy and surgery, (vi) radiotherapy and surgery, and (vii) chemotherapy, radiotherapy and surgery. Multinomial logistic regression models were fitted using surgery alone as the baseline choice of treatment for each cancer type (colour-coded). Only results with P < 0.05 are shown in the figure. P values are annotated on the plots. Full data and confidence intervals are presented in Table S8.

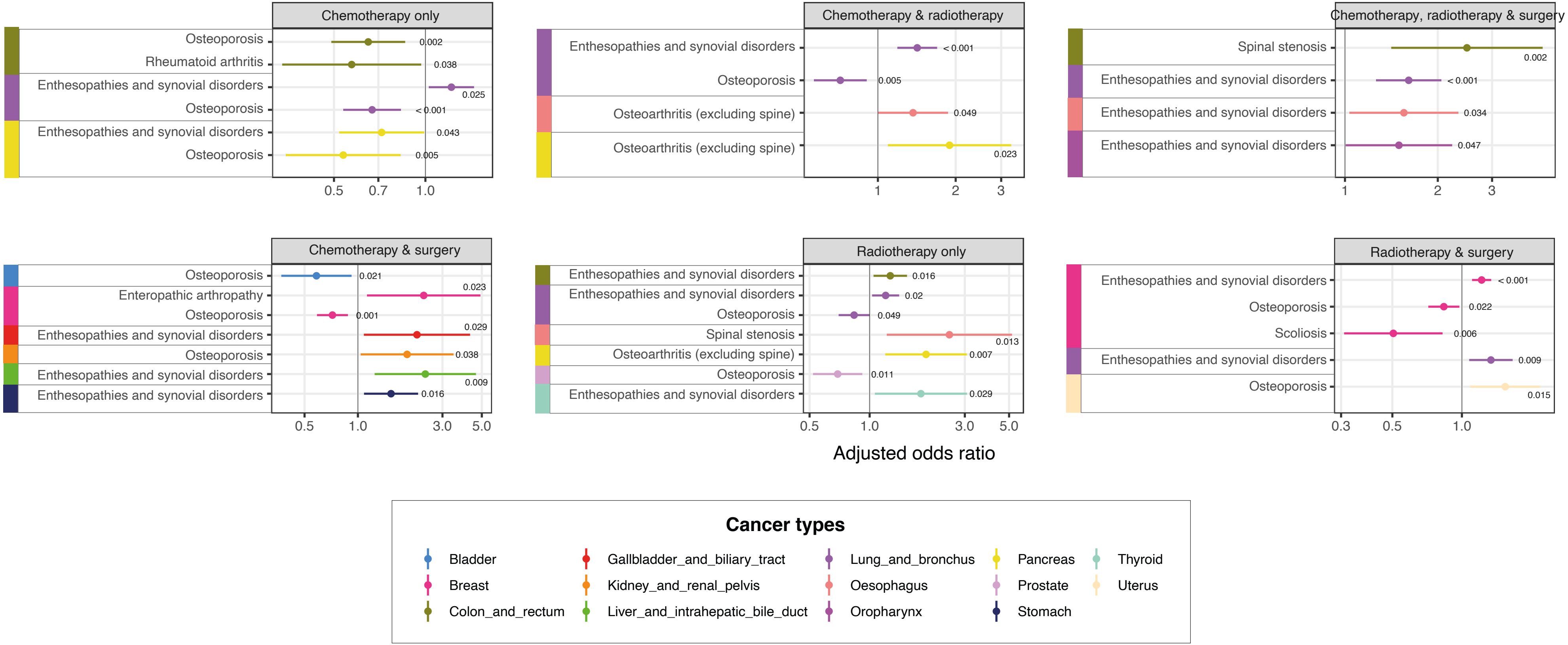

Figure S7. Multinomial logistic regression investigating the associations between neurological conditions and cancer treatment decisions. Forest plots show odds ratios for a particular treatment type, adjusted for age, sex, socioeconomic status, tumour grade, tumour stage, tumour count and multimorbidity count. Seven treatment categories were considered (i) surgery alone, (ii) chemotherapy alone, (iii) radiotherapy alone, (iv) chemotherapy and radiotherapy, (v) chemotherapy and surgery, (vi) radiotherapy and surgery, and (vii) chemotherapy, radiotherapy and surgery. Multinomial logistic regression models were fitted using surgery alone as the baseline choice of treatment for each cancer type (colour-coded). Only results with P < 0.05 are shown in the figure. P values are annotated on the plots. Full data and confidence intervals are presented in Table S8.

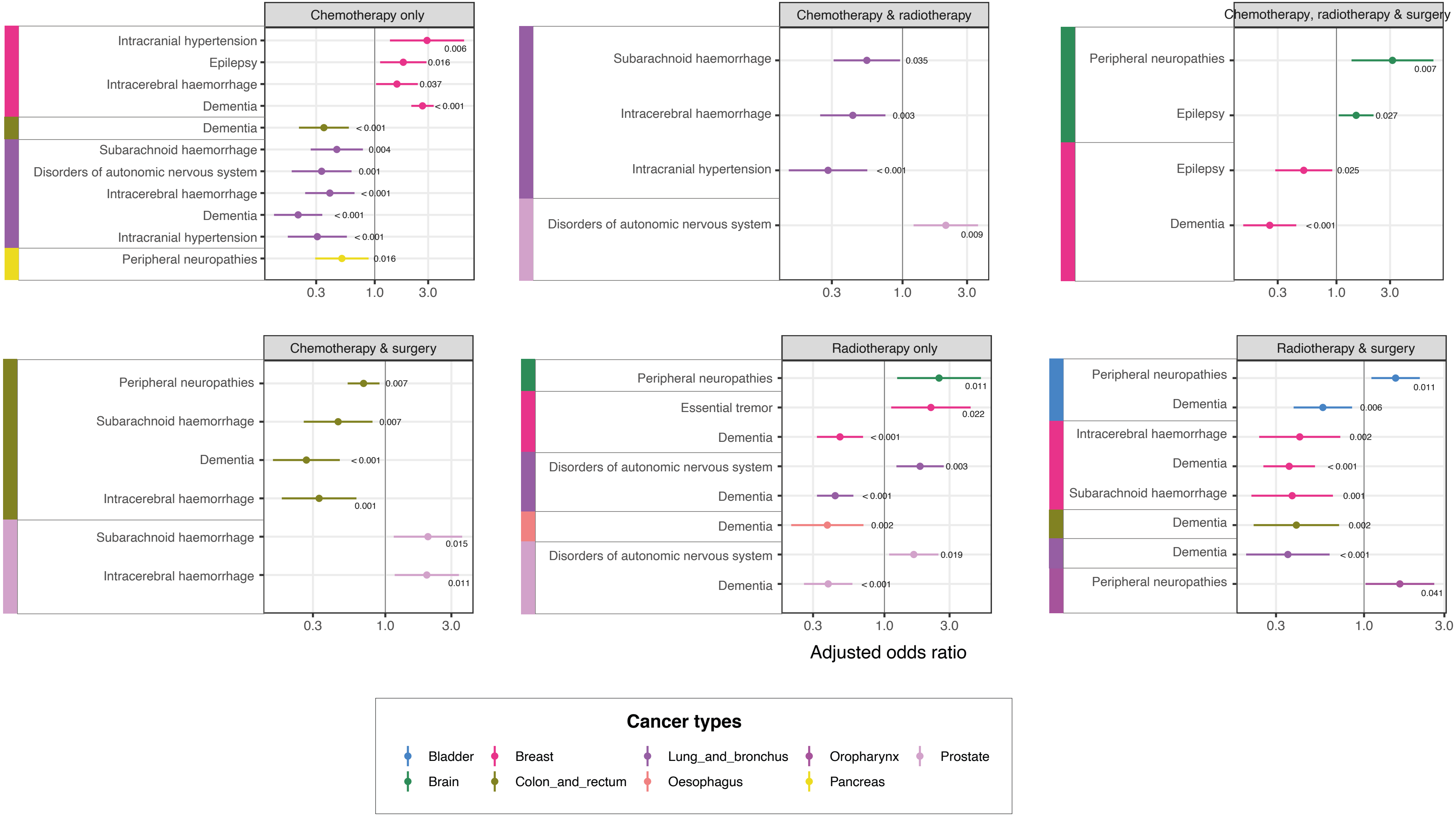

Figure S8. Multinomial logistic regression investigating the associations between pulmonary conditions and cancer treatment decisions. Forest plots show odds ratios for a particular treatment type, adjusted for age, sex, socioeconomic status, tumour grade, tumour stage, tumour count and multimorbidity count. Seven treatment categories were considered (i) surgery alone, (ii) chemotherapy alone, (iii) radiotherapy alone, (iv) chemotherapy and radiotherapy, (v) chemotherapy and surgery, (vi) radiotherapy and surgery, and (vii) chemotherapy, radiotherapy and surgery. Multinomial logistic regression models were fitted using surgery alone as the baseline choice of treatment for each cancer type (colour-coded). Only results with P < 0.05 are shown in the figure. P values are annotated on the plots. Full data and confidence intervals are presented in Table S8.

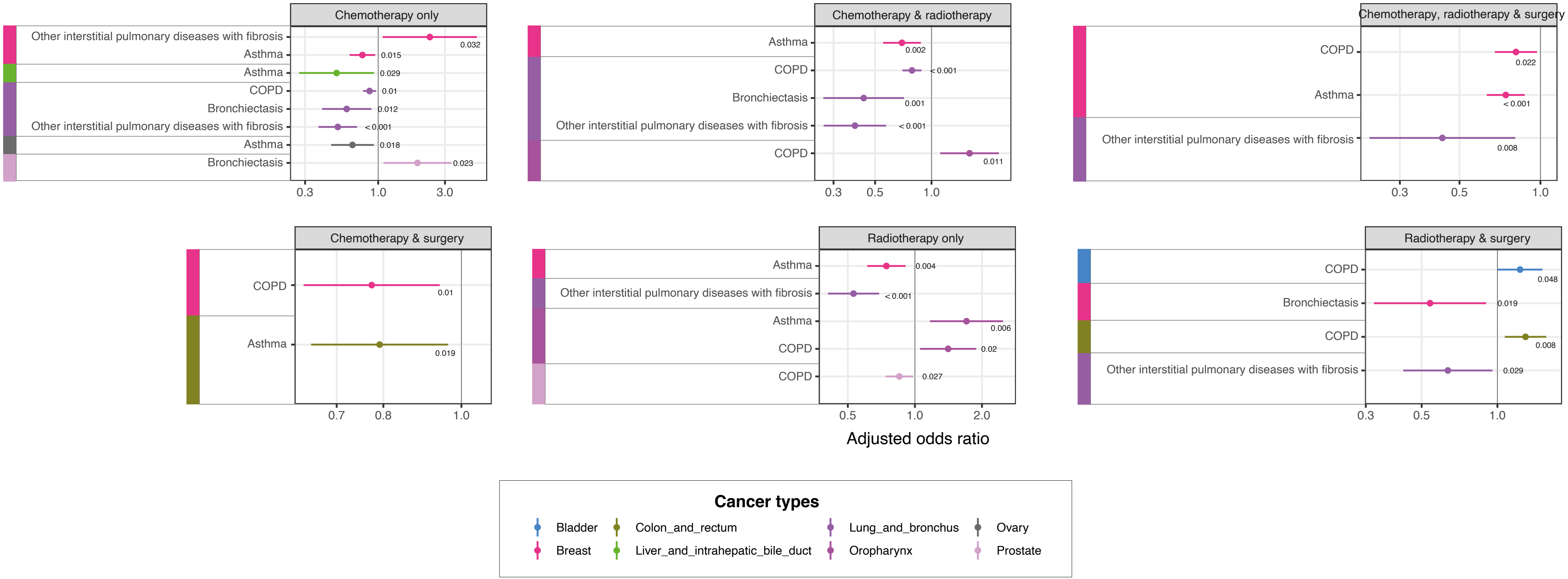

Figure S9. Multinomial logistic regression investigating the associations between renal conditions and cancer treatment decisions. Forest plots show odds ratios for a particular treatment type, adjusted for age, sex, socioeconomic status, tumour grade, tumour stage, tumour count and multimorbidity count. Seven treatment categories were considered (i) surgery alone, (ii) chemotherapy alone, (iii) radiotherapy alone, (iv) chemotherapy and radiotherapy, (v) chemotherapy and surgery, (vi) radiotherapy and surgery, and (vii) chemotherapy, radiotherapy and surgery. Multinomial logistic regression models were fitted using surgery alone as the baseline choice of treatment for each cancer type (colour-coded). Only results with P < 0.05 are shown in the figure. P values are annotated on the plots. Full data and confidence intervals are presented in Table S8.

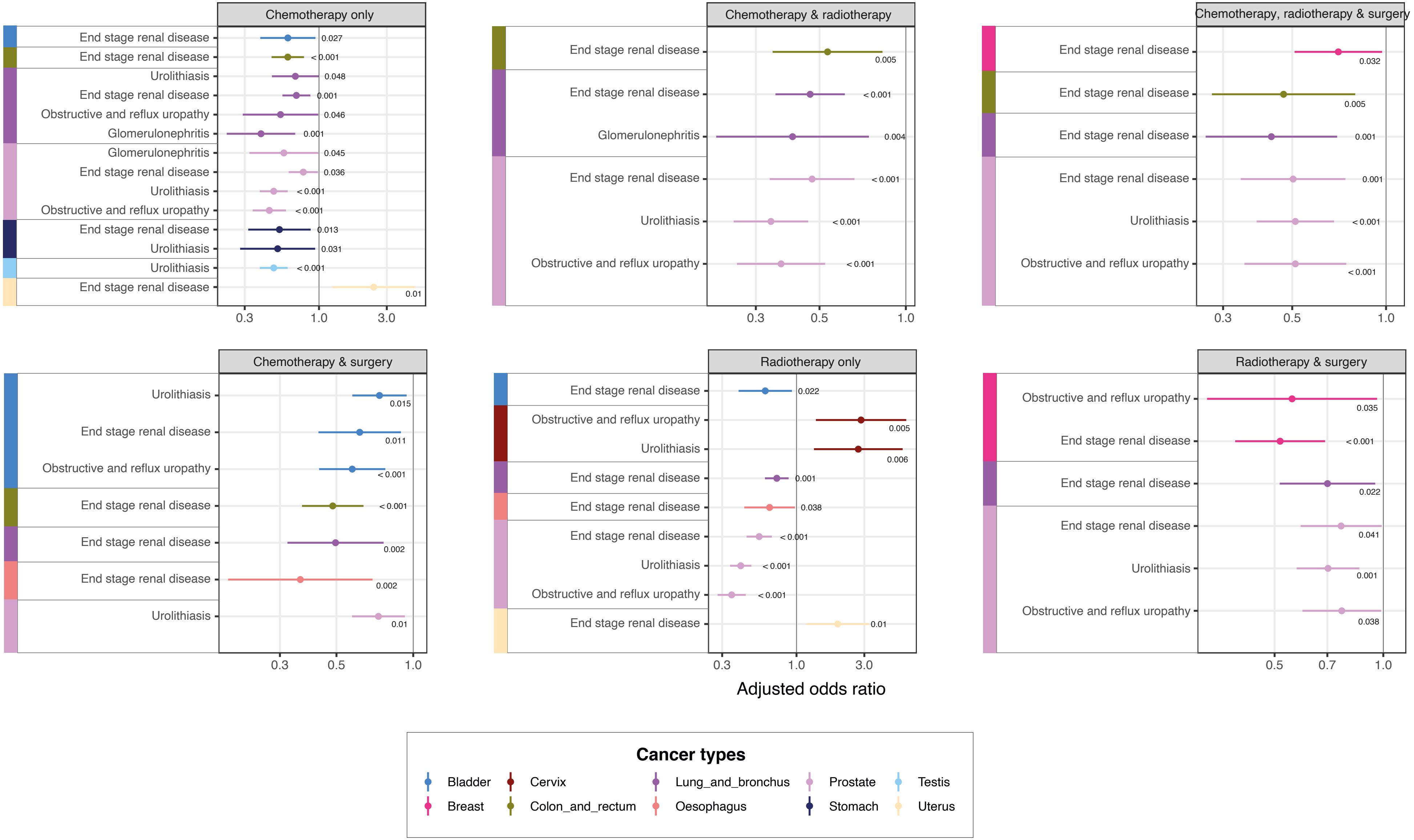

Figure S10. Binomial logistic regression investigating the associations between comorbidity and chemotherapy decisions. Forest plots show odds ratios for a particular chemotherapy type, adjusted for cancer type, age, sex, socioeconomic status, tumour grade, tumour stage, tumour count and multimorbidity count. Ten chemotherapy classes were considered – results for 8 classes are shown in this figure. Plots for the remaining 2 chemotherapy classes are shown in Figure 4. Binomial logistic regression models were fitted. Conditions were colour-coded according to the 9 organ systems. Only results with P < 0.05 are shown in the figure. P values are annotated on the plots. Full data and confidence intervals are presented in Table S9.

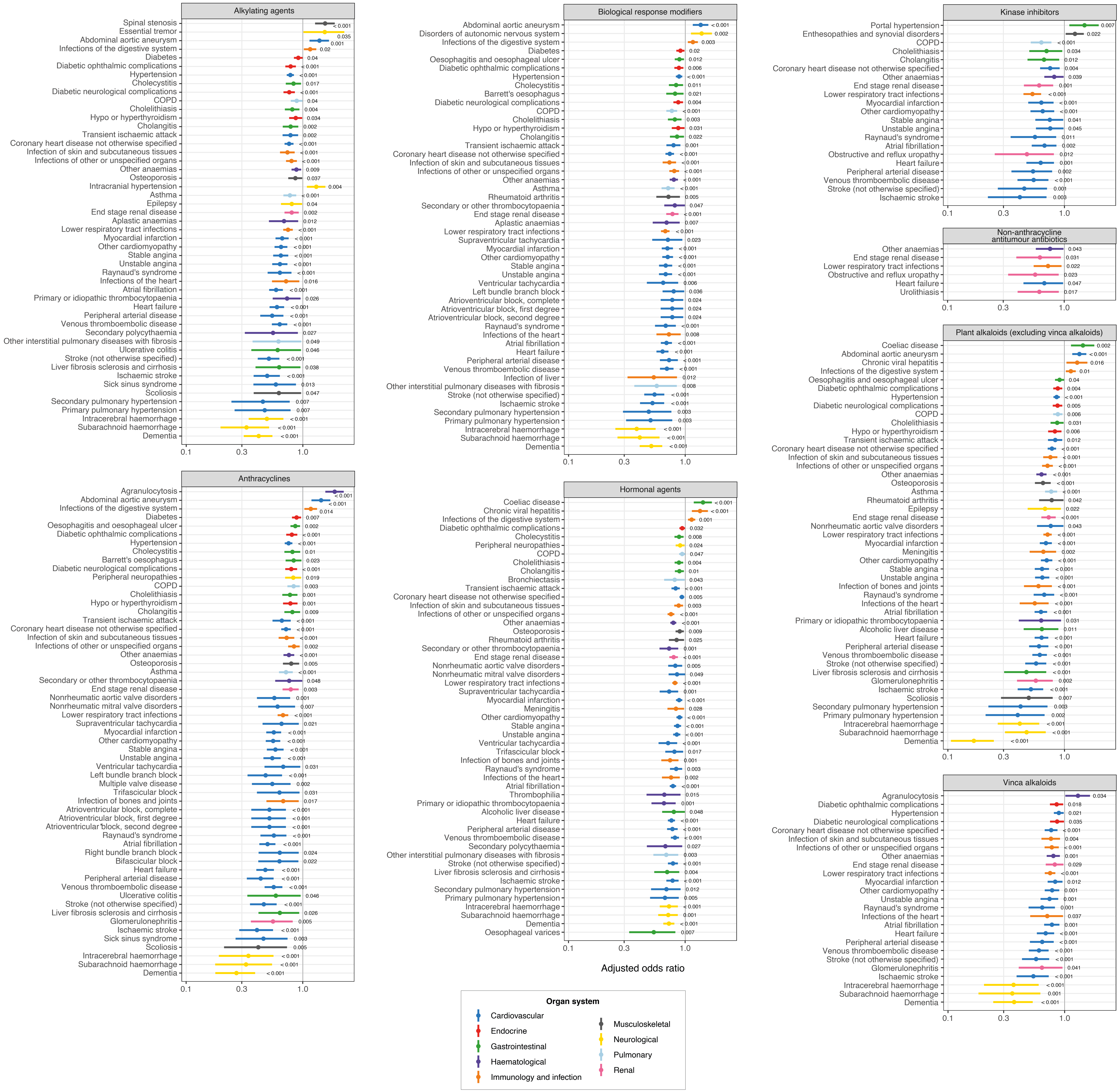

Supplement: Supplementary file 1 — Supplementary Figures. [file 41598_2024_51161_MOESM1_ESM.pdf]
