# Supplementary material for: Pan-cancer analyses of the associations between 109 pre-existing conditions and cancer treatment patterns across 19 adult cancers
Source: Sci Rep. 2024 Jan 3;14:464. doi: 10.1038/s41598-024-51161-0 (PMC10764847; doi:10.1038/s41598-024-51161-0)
Supplement: Supplementary file 2 — Supplementary Tables. [file 41598_2024_51161_MOESM2_ESM.pdf]

Supplementary tables

Pan-cancer analyses of the associations between 109 pre-existing conditions and cancer treatment patterns across 19 adult cancers

Supplementary table legends

Table S1. Baseline characteristics of patients with cancer stratified by treatment type.

Table S2. Baseline characteristics of patients with cancer stratified by chemotherapy type.

Table S3. Treatment patterns by cancer type. 95% confidence intervals (CIs) are shown.

Table S4. Chemotherapy patterns by cancer type. 95% confidence intervals (CIs) are shown.

Table S5. List of 109 health conditions categorised by organ system.

Table S6. Non-cancer conditions diagnosed, for the first time, in the 5-year period before cancer diagnosis. 95% confidence intervals (CIs) are shown.

Table S7. Multimorbidity patterns by cancer type. 95% confidence intervals (CIs) are shown.

Table S8. Multinomial logistic regression results for the associations between health conditions and cancer treatment decisions. Models were fitted using 'surgery alone' as the baseline choice of treatment. Odds ratios were adjusted for age, sex, socioeconomic status, tumour grade, tumour stage, tumour count and multimorbidity count. Strata with < 10 patients were not analysed.

Table S9. Binomial logistic regression, for each chemotherapy type, for the associations between health conditions and chemotherapy decisions. Odds ratios were adjusted for cancer type, age, sex, socioeconomic status, tumour grade, tumour stage, tumour count and multimorbidity count. Strata with < 10 patients were not analysed.

Table S1. Baseline characteristics of patients with cancer stratified by treatment type.

	Full cohort	Surgery alone	Chemotherapy alone	Chemotherapy & radiotherapy	Chemotherapy, radiotherapy & surgery	Chemotherapy & surgery	Radiotherapy alone	Radiotherapy & surgery
**n**	280543	117007	30007	18326	19586	24396	36100	35121
**Male (%)**	131528 (46.9)	55471 (47.4)	15399 (51.3)	9528 (52.0)	6489 (33.1)	10597 (43.4)	21784 (60.3)	12260 (34.9)
**Age groups (%)**								
18-34	5127 (1.8)	3064 (2.6)	395 (1.3)	220 (1.2)	304 (1.6)	470 (1.9)	199 (0.6)	475 (1.4)
35-50	29553 (10.5)	11784 (10.1)	2666 (8.9)	2319 (12.7)	3429 (17.5)	3097 (12.7)	1860 (5.2)	4398 (12.5)
51-65	89511 (31.9)	33031 (28.2)	9848 (32.8)	7277 (39.7)	8039 (41.0)	8312 (34.1)	10185 (28.2)	12819 (36.5)
66-80	120080 (42.8)	48610 (41.5)	13564 (45.2)	7700 (42.0)	7091 (36.2)	10103 (41.4)	18835 (52.2)	14177 (40.4)
≥ 81	36272 (12.9)	20518 (17.5)	3534 (11.8)	810 (4.4)	723 (3.7)	2414 (9.9)	5021 (13.9)	3252 (9.3)
**Age at index (mean (SD))**	66.16 (13.13)	67.15 (14.18)	66.60 (12.32)	63.43 (11.30)	61.45 (11.88)	64.70 (13.07)	69.10 (10.98)	64.49 (12.48)
**Indices of multiple deprivation (%)**								
Least deprived	69124 (24.6)	28981 (24.8)	7656 (25.5)	4206 (23.0)	4939 (25.2)	6121 (25.1)	8549 (23.7)	8672 (24.7)
Most deprived	43350 (15.5)	17562 (15.0)	4882 (16.3)	3375 (18.4)	2522 (12.9)	3575 (14.7)	6290 (17.4)	5144 (14.6)
**Cancer types (%)**								
Bladder	13230 (4.7)	7936 (6.8)	741 (2.5)	268 (1.5)	485 (2.5)	1338 (5.5)	587 (1.6)	1875 (5.3)
Brain	4426 (1.6)	1177 (1.0)	225 (0.7)	432 (2.4)	433 (2.2)	195 (0.8)	1024 (2.8)	940 (2.7)
Breast	66145 (23.6)	18172 (15.5)	5082 (16.9)	4352 (23.7)	10163 (51.9)	6961 (28.5)	5912 (16.4)	15503 (44.1)
Cervix	3463 (1.2)	1768 (1.5)	135 (0.4)	343 (1.9)	242 (1.2)	118 (0.5)	474 (1.3)	383 (1.1)
Colon and rectum	44540 (15.9)	25536 (21.8)	4555 (15.2)	2028 (11.1)	1763 (9.0)	5528 (22.7)	2318 (6.4)	2812 (8.0)
Gallbladder and biliary tract	1257 (0.4)	777 (0.7)	219 (0.7)	19 (0.1)	19 (0.1)	170 (0.7)	25 (0.1)	28 (0.1)
Kidney and renal pelvis	7281 (2.6)	4741 (4.1)	529 (1.8)	219 (1.2)	230 (1.2)	425 (1.7)	555 (1.5)	582 (1.7)
Liver and intrahepatic bile duct	1779 (0.6)	773 (0.7)	647 (2.2)	50 (0.3)	13 (0.1)	159 (0.7)	107 (0.3)	30 (0.1)
Lung and bronchus	31207 (11.1)	5535 (4.7)	5946 (19.8)	4922 (26.9)	1457 (7.4)	1382 (5.7)	9759 (27.0)	2206 (6.3)
Melanoma	16146 (5.8)	14253 (12.2)	80 (0.3)	43 (0.2)	238 (1.2)	528 (2.2)	113 (0.3)	891 (2.5)
Oesophagus	8183 (2.9)	2182 (1.9)	1815 (6.0)	864 (4.7)	500 (2.6)	976 (4.0)	1287 (3.6)	559 (1.6)
Oropharynx	7730 (2.8)	2443 (2.1)	415 (1.4)	935 (5.1)	609 (3.1)	234 (1.0)	1499 (4.2)	1595 (4.5)
Ovary	7803 (2.8)	3507 (3.0)	2149 (7.2)	131 (0.7)	152 (0.8)	1574 (6.5)	107 (0.3)	183 (0.5)
Pancreas	4648 (1.7)	1928 (1.6)	1566 (5.2)	164 (0.9)	97 (0.5)	620 (2.5)	199 (0.6)	74 (0.2)
Prostate	41242 (14.7)	14599 (12.5)	3687 (12.3)	3055 (16.7)	2560 (13.1)	2342 (9.6)	10702 (29.6)	4297 (12.2)
Stomach	6557 (2.3)	3269 (2.8)	1507 (5.0)	225 (1.2)	170 (0.9)	791 (3.2)	355 (1.0)	240 (0.7)
Testis	2670 (1.0)	1474 (1.3)	331 (1.1)	18 (0.1)	29 (0.1)	532 (2.2)	69 (0.2)	217 (0.6)
Thyroid	2532 (0.9)	1411 (1.2)	19 (0.1)	41 (0.2)	47 (0.2)	63 (0.3)	241 (0.7)	710 (2.0)
Uterus	9704 (3.5)	5526 (4.7)	359 (1.2)	217 (1.2)	379 (1.9)	460 (1.9)	767 (2.1)	1996 (5.7)
**Tumour, node, metastasis (TNM) stage (%)**								
I	39564 (14.1)	19769 (16.9)	1907 (6.4)	1349 (7.4)	2334 (11.9)	1820 (7.5)	4156 (11.5)	8229 (23.4)
II	28745 (10.2)	9361 (8.0)	3355 (11.2)	2458 (13.4)	2455 (12.5)	2068 (8.5)	3777 (10.5)	5271 (15.0)
III	20673 (7.4)	4255 (3.6)	4127 (13.8)	2947 (16.1)	1579 (8.1)	2353 (9.6)	3496 (9.7)	1916 (5.5)
IV	25508 (9.1)	4309 (3.7)	7072 (23.6)	3466 (18.9)	1289 (6.6)	2425 (9.9)	5308 (14.7)	1639 (4.7)
**Grade of tumour (%)**								
G1 (well differentiated)	23170 (8.3)	11725 (10.0)	1122 (3.7)	686 (3.7)	1674 (8.5)	1729 (7.1)	1998 (5.5)	4236 (12.1)
G2 (moderately differentiated)	91367 (32.6)	40173 (34.3)	7550 (25.2)	5022 (27.4)	7431 (37.9)	9338 (38.3)	8767 (24.3)	13086 (37.3)
G3 (poorly differentiated)	68189 (24.3)	22489 (19.2)	7585 (25.3)	5515 (30.1)	6823 (34.8)	7349 (30.1)	8449 (23.4)	9979 (28.4)
G4 (undifferentiated)	6386 (2.3)	1494 (1.3)	686 (2.3)	804 (4.4)	706 (3.6)	529 (2.2)	1211 (3.4)	956 (2.7)
**Tumour count (%)**								
0	9758 (3.5)	3414 (2.9)	823 (2.7)	684 (3.7)	1032 (5.3)	1040 (4.3)	1259 (3.5)	1506 (4.3)
1	214281 (76.4)	87668 (74.9)	25468 (84.9)	14975 (81.7)	13947 (71.2)	17850 (73.2)	29285 (81.1)	25088 (71.4)
2	46525 (16.6)	21173 (18.1)	3194 (10.6)	2273 (12.4)	3772 (19.3)	4460 (18.3)	4789 (13.3)	6864 (19.5)
3	8225 (2.9)	3899 (3.3)	453 (1.5)	345 (1.9)	679 (3.5)	856 (3.5)	630 (1.7)	1363 (3.9)
≥ 4	1754 (0.6)	853 (0.7)	69 (0.2)	49 (0.3)	156 (0.8)	190 (0.8)	137 (0.4)	300 (0.9)
**Number of non-cancer conditions (%)**								
0	39965 (14.2)	16426 (14.0)	3733 (12.4)	2650 (14.5)	3451 (17.6)	3651 (15.0)	4362 (12.1)	5692 (16.2)
1	41445 (14.8)	16968 (14.5)	3874 (12.9)	2732 (14.9)	3371 (17.2)	3661 (15.0)	4968 (13.8)	5871 (16.7)
2	36070 (12.9)	14728 (12.6)	3491 (11.6)	2434 (13.3)	2835 (14.5)	3217 (13.2)	4487 (12.4)	4878 (13.9)
3	30331 (10.8)	12251 (10.5)	3090 (10.3)	1985 (10.8)	2375 (12.1)	2732 (11.2)	3924 (10.9)	3974 (11.3)
4	24633 (8.8)	10114 (8.6)	2639 (8.8)	1690 (9.2)	1809 (9.2)	2134 (8.7)	3184 (8.8)	3063 (8.7)
5	20216 (7.2)	8336 (7.1)	2217 (7.4)	1326 (7.2)	1365 (7.0)	1848 (7.6)	2620 (7.3)	2504 (7.1)
≥ 6	87883 (31.3)	38184 (32.6)	10963 (36.5)	5509 (30.1)	4380 (22.4)	7153 (29.3)	12555 (34.8)	9139 (26.0)

Table S2. Baseline characteristics of patients with cancer stratified by chemotherapy type.

	Full cohort	Alkylating agents	Anthracyclines	Antimetabolites	Biological response modifiers including immunotherapy	Hormonal agents	Kinase inhibitors	Non-anthracycline antitumour antibiotics	Plant alkaloids (excluding vinca alkaloids)	Platinum agents	Vinca alkaloids
**n**	57384	8224	8960	21224	7638	30795	1785	791	12819	18490	2147
**Male (%)**	24651 (43.0)	974 (11.8)	1564 (17.5)	9383 (44.2)	2064 (27.0)	13234 (43.0)	944 (52.9)	586 (74.1)	4173 (32.6)	9590 (51.9)	1139 (53.1)
**Age groups (%)**											
18-34	834 (1.5)	230 (2.8)	169 (1.9)	261 (1.2)	158 (2.1)	305 (1.0)	28 (1.6)	125 (15.8)	353 (2.8)	414 (2.2)	37 (1.7)
35-50	6971 (12.1)	2492 (30.3)	2375 (26.5)	3045 (14.3)	1320 (17.3)	3329 (10.8)	198 (11.1)	104 (13.1)	2415 (18.8)	1811 (9.8)	226 (10.5)
51-65	20218 (35.2)	3752 (45.6)	4081 (45.5)	8700 (41.0)	3194 (41.8)	9719 (31.6)	695 (38.9)	169 (21.4)	5513 (43.0)	7407 (40.1)	863 (40.2)
66-80	23972 (41.8)	1686 (20.5)	2254 (25.2)	8587 (40.5)	2618 (34.3)	13234 (43.0)	769 (43.1)	304 (38.4)	4319 (33.7)	8230 (44.5)	965 (44.9)
≥ 81	5389 (9.4)	64 (0.8)	81 (0.9)	631 (3.0)	348 (4.6)	4208 (13.7)	95 (5.3)	89 (11.3)	219 (1.7)	628 (3.4)	56 (2.6)
**Age at index (mean (SD))**	64.85 (12.56)	55.89 (11.07)	57.56 (11.03)	62.43 (11.15)	61.20 (12.12)	66.68 (12.83)	63.71 (11.52)	59.85 (19.02)	59.82 (11.76)	63.49 (11.25)	63.44 (10.93)
**Indices of multiple deprivation (%)**											
Least deprived	14934 (26.0)	2290 (27.8)	2443 (27.3)	5544 (26.1)	2062 (27.0)	8056 (26.2)	474 (26.6)	219 (27.7)	3387 (26.4)	4463 (24.1)	470 (21.9)
Most deprived	8230 (14.3)	1144 (13.9)	1284 (14.3)	3152 (14.9)	1148 (15.0)	3894 (12.6)	285 (16.0)	111 (14.0)	1989 (15.5)	3129 (16.9)	432 (20.1)
**Cancer types (%)**											
Bladder	1096 (1.9)	30 (0.4)	67 (0.7)	718 (3.4)	52 (0.7)	326 (1.1)	21 (1.2)	232 (29.3)	101 (0.8)	682 (3.7)	64 (3.0)
Brain	791 (1.4)	709 (8.6)	3 (0.0)	6 (0.0)	26 (0.3)	111 (0.4)	1 (0.1)	NA	9 (0.1)	18 (0.1)	200 (9.3)
Breast	20599 (35.9)	6596 (80.2)	6227 (69.5)	5931 (27.9)	4068 (53.3)	13876 (45.1)	203 (11.4)	50 (6.3)	5105 (39.8)	823 (4.5)	281 (13.1)
Cervix	432 (0.8)	17 (0.2)	19 (0.2)	29 (0.1)	56 (0.7)	126 (0.4)	1 (0.1)	6 (0.8)	128 (1.0)	379 (2.0)	7 (0.3)
Colon and rectum	7572 (13.2)	122 (1.5)	110 (1.2)	6643 (31.3)	1320 (17.3)	2001 (6.5)	36 (2.0)	129 (16.3)	1758 (13.7)	4014 (21.7)	70 (3.3)
Gallbladder and biliary tract	234 (0.4)	1 (0.0)	5 (0.1)	218 (1.0)	4 (0.1)	63 (0.2)	2 (0.1)	1 (0.1)	17 (0.1)	129 (0.7)	1 (0.0)
Kidney and renal pelvis	929 (1.6)	25 (0.3)	19 (0.2)	143 (0.7)	351 (4.6)	145 (0.5)	461 (25.8)	14 (1.8)	38 (0.3)	131 (0.7)	23 (1.1)
Liver and intrahepatic bile duct	373 (0.7)	8 (0.1)	30 (0.3)	230 (1.1)	14 (0.2)	89 (0.3)	102 (5.7)	NA	5 (0.0)	181 (1.0)	4 (0.2)
Lung and bronchus	5950 (10.4)	226 (2.7)	197 (2.2)	2570 (12.1)	401 (5.3)	1909 (6.2)	665 (37.3)	36 (4.6)	1752 (13.7)	4877 (26.4)	1294 (60.3)
Melanoma	642 (1.1)	139 (1.7)	39 (0.4)	92 (0.4)	326 (4.3)	131 (0.4)	129 (7.2)	18 (2.3)	38 (0.3)	87 (0.5)	34 (1.6)
Oesophagus	1798 (3.1)	16 (0.2)	892 (10.0)	1431 (6.7)	117 (1.5)	658 (2.1)	9 (0.5)	11 (1.4)	247 (1.9)	1623 (8.8)	7 (0.3)
Oropharynx	1216 (2.1)	21 (0.3)	28 (0.3)	388 (1.8)	211 (2.8)	438 (1.4)	13 (0.7)	5 (0.6)	179 (1.4)	1009 (5.5)	21 (1.0)
Ovary	2029 (3.5)	63 (0.8)	425 (4.7)	303 (1.4)	309 (4.0)	706 (2.3)	5 (0.3)	6 (0.8)	1107 (8.6)	1769 (9.6)	5 (0.2)
Pancreas	1216 (2.1)	9 (0.1)	8 (0.1)	1139 (5.4)	19 (0.2)	223 (0.7)	21 (1.2)	7 (0.9)	323 (2.5)	352 (1.9)	2 (0.1)
Prostate	9877 (17.2)	155 (1.9)	102 (1.1)	428 (2.0)	209 (2.7)	9000 (29.2)	48 (2.7)	73 (9.2)	1135 (8.9)	387 (2.1)	104 (4.8)
Stomach	1049 (1.8)	14 (0.2)	633 (7.1)	823 (3.9)	74 (1.0)	366 (1.2)	48 (2.7)	11 (1.4)	135 (1.1)	863 (4.7)	7 (0.3)
Testis	526 (0.9)	19 (0.2)	4 (0.0)	17 (0.1)	4 (0.1)	165 (0.5)	2 (0.1)	185 (23.4)	227 (1.8)	489 (2.6)	7 (0.3)
Thyroid	102 (0.2)	5 (0.1)	12 (0.1)	16 (0.1)	13 (0.2)	60 (0.2)	11 (0.6)	1 (0.1)	15 (0.1)	21 (0.1)	2 (0.1)
Uterus	953 (1.7)	49 (0.6)	140 (1.6)	99 (0.5)	64 (0.8)	402 (1.3)	7 (0.4)	6 (0.8)	500 (3.9)	656 (3.5)	14 (0.7)
**Tumour, node, metastasis (TNM) stage (%)**											
I	5905 (10.3)	1265 (15.4)	1295 (14.5)	1696 (8.0)	1157 (15.1)	3338 (10.8)	110 (6.2)	182 (23.0)	1241 (9.7)	1493 (8.1)	186 (8.7)
II	8274 (14.4)	2992 (36.4)	3008 (33.6)	4350 (20.5)	1582 (20.7)	3857 (12.5)	138 (7.7)	109 (13.8)	2662 (20.8)	1980 (10.7)	335 (15.6)
III	8301 (14.5)	1097 (13.3)	1695 (18.9)	4813 (22.7)	1134 (14.8)	3220 (10.5)	273 (15.3)	72 (9.1)	2745 (21.4)	4883 (26.4)	512 (23.8)
IV	10223 (17.8)	325 (4.0)	896 (10.0)	5142 (24.2)	1858 (24.3)	4168 (13.5)	775 (43.4)	102 (12.9)	3466 (27.0)	5992 (32.4)	354 (16.5)
**Grade of tumour (%)**											
G1 (well differentiated)	3823 (6.7)	313 (3.8)	338 (3.8)	780 (3.7)	380 (5.0)	2750 (8.9)	83 (4.6)	41 (5.2)	424 (3.3)	662 (3.6)	97 (4.5)
G2 (moderately differentiated)	19652 (34.2)	2924 (35.6)	3158 (35.2)	8711 (41.0)	2865 (37.5)	10682 (34.7)	350 (19.6)	187 (23.6)	3770 (29.4)	5408 (29.2)	471 (21.9)
G3 (poorly differentiated)	17142 (29.9)	3753 (45.6)	4416 (49.3)	7236 (34.1)	2800 (36.7)	8823 (28.7)	405 (22.7)	216 (27.3)	4691 (36.6)	5744 (31.1)	616 (28.7)
G4 (undifferentiated)	1691 (2.9)	461 (5.6)	40 (0.4)	96 (0.5)	111 (1.5)	966 (3.1)	74 (4.1)	28 (3.5)	288 (2.2)	196 (1.1)	117 (5.4)
**Tumour count (%)**											
0	2076 (3.6)	253 (3.1)	179 (2.0)	452 (2.1)	212 (2.8)	1365 (4.4)	63 (3.5)	21 (2.7)	211 (1.6)	344 (1.9)	87 (4.1)
1	44058 (76.8)	6335 (77.0)	7217 (80.5)	16760 (79.0)	5681 (74.4)	23172 (75.2)	1371 (76.8)	539 (68.1)	10544 (82.3)	14871 (80.4)	1521 (70.8)
2	9273 (16.2)	1355 (16.5)	1322 (14.8)	3327 (15.7)	1390 (18.2)	5158 (16.7)	291 (16.3)	168 (21.2)	1754 (13.7)	2724 (14.7)	420 (19.6)
3	1635 (2.8)	222 (2.7)	198 (2.2)	572 (2.7)	275 (3.6)	912 (3.0)	48 (2.7)	43 (5.4)	266 (2.1)	459 (2.5)	89 (4.1)
≥ 4	342 (0.6)	59 (0.7)	44 (0.5)	113 (0.5)	80 (1.0)	188 (0.6)	12 (0.7)	20 (2.5)	44 (0.3)	92 (0.5)	30 (1.4)
**Number of non-cancer conditions (%)**											
0	7826 (13.6)	1453 (17.7)	1451 (16.2)	2649 (12.5)	1077 (14.1)	4489 (14.6)	209 (11.7)	150 (19.0)	1790 (14.0)	2086 (11.3)	239 (11.1)
1	8201 (14.3)	1461 (17.8)	1530 (17.1)	2824 (13.3)	1149 (15.0)	4639 (15.1)	223 (12.5)	139 (17.6)	1875 (14.6)	2283 (12.3)	271 (12.6)
2	7353 (12.8)	1223 (14.9)	1280 (14.3)	2628 (12.4)	993 (13.0)	4096 (13.3)	194 (10.9)	78 (9.9)	1661 (13.0)	2095 (11.3)	251 (11.7)
3	6318 (11.0)	987 (12.0)	1048 (11.7)	2303 (10.9)	897 (11.7)	3467 (11.3)	189 (10.6)	71 (9.0)	1424 (11.1)	1903 (10.3)	211 (9.8)
4	5151 (9.0)	729 (8.9)	828 (9.2)	1955 (9.2)	680 (8.9)	2806 (9.1)	169 (9.5)	62 (7.8)	1179 (9.2)	1706 (9.2)	172 (8.0)
5	4310 (7.5)	625 (7.6)	708 (7.9)	1657 (7.8)	599 (7.8)	2241 (7.3)	124 (6.9)	51 (6.4)	1006 (7.8)	1436 (7.8)	184 (8.6)
≥ 6	18225 (31.8)	1746 (21.2)	2115 (23.6)	7208 (34.0)	2243 (29.4)	9057 (29.4)	677 (37.9)	240 (30.3)	3884 (30.3)	6981 (37.8)	819 (38.1)

Table S3. Treatment patterns by cancer type. 95% confidence intervals (CIs) are shown.

Cancer type	Treatment type	Proportion (%)	Lower CI	Upper CI
Bladder	Chemotherapy & radiotherapy	2.03	0.34	3.71
Bladder	Chemotherapy & surgery	10.11	8.50	11.73
Bladder	Chemotherapy alone	5.60	3.95	7.26
Bladder	Chemotherapy, radiotherapy & surgery	3.67	1.99	5.34
Bladder	Radiotherapy & surgery	14.17	12.59	15.75
Bladder	Radiotherapy alone	4.44	2.77	6.10
Bladder	Surgery alone	59.98	58.91	61.06
Brain	Chemotherapy & radiotherapy	9.76	6.96	12.56
Brain	Chemotherapy & surgery	4.41	1.53	7.29
Brain	Chemotherapy alone	5.08	2.21	7.95
Brain	Chemotherapy, radiotherapy & surgery	9.78	6.98	12.58
Brain	Radiotherapy & surgery	21.24	18.62	23.85
Brain	Radiotherapy alone	23.14	20.55	25.72
Brain	Surgery alone	26.59	24.07	29.12
Breast	Chemotherapy & radiotherapy	6.58	5.84	7.32
Breast	Chemotherapy & surgery	10.52	9.80	11.24
Breast	Chemotherapy alone	7.68	6.95	8.42
Breast	Chemotherapy, radiotherapy & surgery	15.36	14.66	16.07
Breast	Radiotherapy & surgery	23.44	22.77	24.10
Breast	Radiotherapy alone	8.94	8.21	9.67
Breast	Surgery alone	27.47	26.82	28.12
Cervix	Chemotherapy & radiotherapy	9.90	6.74	13.07
Cervix	Chemotherapy & surgery	3.41	0.13	6.68
Cervix	Chemotherapy alone	3.90	0.63	7.16
Cervix	Chemotherapy, radiotherapy & surgery	6.99	3.78	10.20
Cervix	Radiotherapy & surgery	11.06	7.92	14.20
Cervix	Radiotherapy alone	13.69	10.59	16.78
Cervix	Surgery alone	51.05	48.72	53.38
Colon and rectum	Chemotherapy & radiotherapy	4.55	3.65	5.46
Colon and rectum	Chemotherapy & surgery	12.41	11.54	13.28
Colon and rectum	Chemotherapy alone	10.23	9.35	11.11
Colon and rectum	Chemotherapy, radiotherapy & surgery	3.96	3.05	4.87
Colon and rectum	Radiotherapy & surgery	6.31	5.41	7.21
Colon and rectum	Radiotherapy alone	5.20	4.30	6.11
Colon and rectum	Surgery alone	57.33	56.73	57.94
Gallbladder and biliary tract	Chemotherapy & radiotherapy	1.51	0.00	7.00
Gallbladder and biliary tract	Chemotherapy & surgery	13.52	8.38	18.67
Gallbladder and biliary tract	Chemotherapy alone	17.42	12.40	22.45
Gallbladder and biliary tract	Chemotherapy, radiotherapy & surgery	1.51	0.00	7.00
Gallbladder and biliary tract	Radiotherapy & surgery	2.23	0.00	7.69
Gallbladder and biliary tract	Radiotherapy alone	1.99	0.00	7.46
Gallbladder and biliary tract	Surgery alone	61.81	58.40	65.23
Kidney and renal pelvis	Chemotherapy & radiotherapy	3.01	0.75	5.27
Kidney and renal pelvis	Chemotherapy & surgery	5.84	3.61	8.07
Kidney and renal pelvis	Chemotherapy alone	7.27	5.05	9.48
Kidney and renal pelvis	Chemotherapy, radiotherapy & surgery	3.16	0.90	5.42
Kidney and renal pelvis	Radiotherapy & surgery	7.99	5.79	10.20
Kidney and renal pelvis	Radiotherapy alone	7.62	5.41	9.83
Kidney and renal pelvis	Surgery alone	65.11	63.76	66.47
Liver and intrahepatic bile duct	Chemotherapy & radiotherapy	2.81	0.00	7.39
Liver and intrahepatic bile duct	Chemotherapy & surgery	8.94	4.50	13.37
Liver and intrahepatic bile duct	Chemotherapy alone	36.37	32.66	40.08
Liver and intrahepatic bile duct	Chemotherapy, radiotherapy & surgery	0.73	0.00	5.36
Liver and intrahepatic bile duct	Radiotherapy & surgery	1.69	0.00	6.29
Liver and intrahepatic bile duct	Radiotherapy alone	6.01	1.51	10.52
Liver and intrahepatic bile duct	Surgery alone	43.45	39.96	46.95
Lung and bronchus	Chemotherapy & radiotherapy	15.77	14.75	16.79
Lung and bronchus	Chemotherapy & surgery	4.43	3.34	5.51
Lung and bronchus	Chemotherapy alone	19.05	18.06	20.05
Lung and bronchus	Chemotherapy, radiotherapy & surgery	4.67	3.59	5.75
Lung and bronchus	Radiotherapy & surgery	7.07	6.00	8.14
Lung and bronchus	Radiotherapy alone	31.27	30.35	32.19
Lung and bronchus	Surgery alone	17.74	16.73	18.74
Melanoma	Chemotherapy & radiotherapy	0.27	0.00	1.81
Melanoma	Chemotherapy & surgery	3.27	1.75	4.79
Melanoma	Chemotherapy alone	0.50	0.00	2.03
Melanoma	Chemotherapy, radiotherapy & surgery	1.47	0.00	3.01
Melanoma	Radiotherapy & surgery	5.52	4.02	7.02
Melanoma	Radiotherapy alone	0.70	0.00	2.24
Melanoma	Surgery alone	88.28	87.75	88.80
Oesophagus	Chemotherapy & radiotherapy	10.56	8.51	12.61
Oesophagus	Chemotherapy & surgery	11.93	9.89	13.96
Oesophagus	Chemotherapy alone	22.18	20.27	24.09
Oesophagus	Chemotherapy, radiotherapy & surgery	6.11	4.01	8.21
Oesophagus	Radiotherapy & surgery	6.83	4.74	8.92
Oesophagus	Radiotherapy alone	15.73	13.74	17.72
Oesophagus	Surgery alone	26.67	24.81	28.52
Oropharynx	Chemotherapy & radiotherapy	12.10	10.01	14.19
Oropharynx	Chemotherapy & surgery	3.03	0.83	5.22
Oropharynx	Chemotherapy alone	5.37	3.20	7.54
Oropharynx	Chemotherapy, radiotherapy & surgery	7.88	5.74	10.02
Oropharynx	Radiotherapy & surgery	20.63	18.65	22.62
Oropharynx	Radiotherapy alone	19.39	17.39	21.39
Oropharynx	Surgery alone	31.60	29.76	33.45
Ovary	Chemotherapy & radiotherapy	1.68	0.00	3.88
Ovary	Chemotherapy & surgery	20.17	18.19	22.15
Ovary	Chemotherapy alone	27.54	25.65	29.43
Ovary	Chemotherapy, radiotherapy & surgery	1.95	0.00	4.15
Ovary	Radiotherapy & surgery	2.35	0.15	4.54
Ovary	Radiotherapy alone	1.37	0.00	3.57
Ovary	Surgery alone	44.94	43.30	46.59
Pancreas	Chemotherapy & radiotherapy	3.53	0.70	6.35
Pancreas	Chemotherapy & surgery	13.34	10.66	16.02
Pancreas	Chemotherapy alone	33.69	31.35	36.03
Pancreas	Chemotherapy, radiotherapy & surgery	2.09	0.00	4.93
Pancreas	Radiotherapy & surgery	1.59	0.00	4.44
Pancreas	Radiotherapy alone	4.28	1.47	7.09
Pancreas	Surgery alone	41.48	39.28	43.68
Prostate	Chemotherapy & radiotherapy	7.41	6.48	8.34
Prostate	Chemotherapy & surgery	5.68	4.74	6.62
Prostate	Chemotherapy alone	8.94	8.02	9.86
Prostate	Chemotherapy, radiotherapy & surgery	6.21	5.27	7.14
Prostate	Radiotherapy & surgery	10.42	9.51	11.33
Prostate	Radiotherapy alone	25.95	25.12	26.78
Prostate	Surgery alone	35.40	34.62	36.17
Stomach	Chemotherapy & radiotherapy	3.43	1.05	5.81
Stomach	Chemotherapy & surgery	12.06	9.79	14.33
Stomach	Chemotherapy alone	22.98	20.86	25.11
Stomach	Chemotherapy, radiotherapy & surgery	2.59	0.20	4.98
Stomach	Radiotherapy & surgery	3.66	1.28	6.04
Stomach	Radiotherapy alone	5.41	3.06	7.77
Stomach	Surgery alone	49.86	48.14	51.57
Testis	Chemotherapy & radiotherapy	0.67	0.00	4.45
Testis	Chemotherapy & surgery	19.93	16.53	23.32
Testis	Chemotherapy alone	12.40	8.85	15.95
Testis	Chemotherapy, radiotherapy & surgery	1.09	0.00	4.86
Testis	Radiotherapy & surgery	8.13	4.49	11.76
Testis	Radiotherapy alone	2.58	0.00	6.33
Testis	Surgery alone	55.21	52.67	57.74
Thyroid	Chemotherapy & radiotherapy	1.62	0.00	5.48
Thyroid	Chemotherapy & surgery	2.49	0.00	6.33
Thyroid	Chemotherapy alone	0.75	0.00	4.63
Thyroid	Chemotherapy, radiotherapy & surgery	1.86	0.00	5.72
Thyroid	Radiotherapy & surgery	28.04	24.74	31.35
Thyroid	Radiotherapy alone	9.52	5.81	13.22
Thyroid	Surgery alone	55.73	53.13	58.32
Uterus	Chemotherapy & radiotherapy	2.24	0.27	4.20
Uterus	Chemotherapy & surgery	4.74	2.80	6.68
Uterus	Chemotherapy alone	3.70	1.75	5.65
Uterus	Chemotherapy, radiotherapy & surgery	3.91	1.96	5.86
Uterus	Radiotherapy & surgery	20.57	18.80	22.34
Uterus	Radiotherapy alone	7.90	5.99	9.81
Uterus	Surgery alone	56.95	55.64	58.25

Table S4. Chemotherapy patterns by cancer type. 95% confidence intervals (CIs) are shown.

Cancer type	Chemotherapy type	Proportion (%)	Lower CI	Upper CI
Bladder	Alkylating agents	1.31	0.84	1.77
Bladder	Anthracyclines	2.92	2.23	3.61
Bladder	Antimetabolites	31.31	29.41	33.21
Bladder	Biological response modifiers including immunotherapy	2.27	1.66	2.88
Bladder	Hormonal agents	14.22	12.79	15.65
Bladder	Kinase inhibitors	0.92	0.53	1.31
Bladder	Non-anthracycline antitumour antibiotics	10.12	8.88	11.35
Bladder	Plant alkaloids excluding vinca alkaloids	4.40	3.56	5.24
Bladder	Platinum agents	29.74	27.87	31.61
Bladder	Vinca alkaloid	2.79	2.12	3.47
Brain	Alkylating agents	65.47	62.63	68.30
Brain	Anthracyclines	0.28	0.00	0.59
Brain	Antimetabolites	0.55	0.11	1.00
Brain	Biological response modifiers including immunotherapy	2.40	1.49	3.31
Brain	Hormonal agents	10.25	8.44	12.06
Brain	Kinase inhibitors	0.09	0.00	0.27
Brain	Plant alkaloids excluding vinca alkaloids	0.83	0.29	1.37
Brain	Platinum agents	1.66	0.90	2.42
Brain	Vinca alkaloid	18.47	16.16	20.78
Breast	Alkylating agents	15.28	14.94	15.62
Breast	Anthracyclines	14.43	14.10	14.76
Breast	Antimetabolites	13.74	13.42	14.07
Breast	Biological response modifiers including immunotherapy	9.43	9.15	9.70
Breast	Hormonal agents	32.15	31.71	32.59
Breast	Kinase inhibitors	0.47	0.41	0.53
Breast	Non-anthracycline antitumour antibiotics	0.12	0.08	0.15
Breast	Plant alkaloids excluding vinca alkaloids	11.83	11.52	12.13
Breast	Platinum agents	1.91	1.78	2.04
Breast	Vinca alkaloid	0.65	0.58	0.73
Cervix	Alkylating agents	2.21	1.17	3.25
Cervix	Anthracyclines	2.47	1.38	3.57
Cervix	Antimetabolites	3.78	2.43	5.12
Cervix	Biological response modifiers including immunotherapy	7.29	5.45	9.13
Cervix	Hormonal agents	16.41	13.79	19.03
Cervix	Kinase inhibitors	0.13	0.00	0.39
Cervix	Non-anthracycline antitumour antibiotics	0.78	0.16	1.40
Cervix	Plant alkaloids excluding vinca alkaloids	16.67	14.03	19.30
Cervix	Platinum agents	49.35	45.81	52.88
Cervix	Vinca alkaloid	0.91	0.24	1.58
Colon and rectum	Alkylating agents	0.75	0.62	0.89
Colon and rectum	Anthracyclines	0.68	0.55	0.81
Colon and rectum	Antimetabolites	41.00	40.24	41.76
Colon and rectum	Biological response modifiers including immunotherapy	8.15	7.73	8.57
Colon and rectum	Hormonal agents	12.35	11.84	12.86
Colon and rectum	Kinase inhibitors	0.22	0.15	0.29
Colon and rectum	Non-anthracycline antitumour antibiotics	0.80	0.66	0.93
Colon and rectum	Plant alkaloids excluding vinca alkaloids	10.85	10.37	11.33
Colon and rectum	Platinum agents	24.77	24.11	25.44
Colon and rectum	Vinca alkaloid	0.43	0.33	0.53
Gallbladder and biliary tract	Alkylating agents	0.23	0.00	0.67
Gallbladder and biliary tract	Anthracyclines	1.13	0.15	2.12
Gallbladder and biliary tract	Antimetabolites	49.43	44.77	54.10
Gallbladder and biliary tract	Biological response modifiers including immunotherapy	0.91	0.02	1.79
Gallbladder and biliary tract	Hormonal agents	14.29	11.02	17.55
Gallbladder and biliary tract	Kinase inhibitors	0.45	0.00	1.08
Gallbladder and biliary tract	Non-anthracycline antitumour antibiotics	0.23	0.00	0.67
Gallbladder and biliary tract	Plant alkaloids excluding vinca alkaloids	3.85	2.06	5.65
Gallbladder and biliary tract	Platinum agents	29.25	25.01	33.50
Gallbladder and biliary tract	Vinca alkaloid	0.23	0.00	0.67
Kidney and renal pelvis	Alkylating agents	1.85	1.13	2.57
Kidney and renal pelvis	Anthracyclines	1.41	0.78	2.04
Kidney and renal pelvis	Antimetabolites	10.59	8.95	12.23
Kidney and renal pelvis	Biological response modifiers including immunotherapy	26.00	23.66	28.34
Kidney and renal pelvis	Hormonal agents	10.74	9.09	12.39
Kidney and renal pelvis	Kinase inhibitors	34.15	31.62	36.68
Kidney and renal pelvis	Non-anthracycline antitumour antibiotics	1.04	0.50	1.58
Kidney and renal pelvis	Plant alkaloids excluding vinca alkaloids	2.81	1.93	3.70
Kidney and renal pelvis	Platinum agents	9.70	8.12	11.28
Kidney and renal pelvis	Vinca alkaloid	1.70	1.01	2.39
Liver and intrahepatic bile duct	Alkylating agents	1.21	0.38	2.04
Liver and intrahepatic bile duct	Anthracyclines	4.52	2.94	6.11
Liver and intrahepatic bile duct	Antimetabolites	34.69	31.07	38.31
Liver and intrahepatic bile duct	Biological response modifiers including immunotherapy	2.11	1.02	3.21
Liver and intrahepatic bile duct	Hormonal agents	13.42	10.83	16.02
Liver and intrahepatic bile duct	Kinase inhibitors	15.38	12.64	18.13
Liver and intrahepatic bile duct	Plant alkaloids excluding vinca alkaloids	0.75	0.10	1.41
Liver and intrahepatic bile duct	Platinum agents	27.30	23.91	30.69
Liver and intrahepatic bile duct	Vinca alkaloid	0.60	0.01	1.19
Lung and bronchus	Alkylating agents	1.62	1.41	1.83
Lung and bronchus	Anthracyclines	1.41	1.22	1.61
Lung and bronchus	Antimetabolites	18.45	17.81	19.10
Lung and bronchus	Biological response modifiers including immunotherapy	2.88	2.60	3.16
Lung and bronchus	Hormonal agents	13.71	13.14	14.28
Lung and bronchus	Kinase inhibitors	4.77	4.42	5.13
Lung and bronchus	Non-anthracycline antitumour antibiotics	0.26	0.17	0.34
Lung and bronchus	Plant alkaloids excluding vinca alkaloids	12.58	12.03	13.13
Lung and bronchus	Platinum agents	35.02	34.23	35.81
Lung and bronchus	Vinca alkaloid	9.29	8.81	9.77
Melanoma	Alkylating agents	13.46	11.37	15.54
Melanoma	Anthracyclines	3.78	2.61	4.94
Melanoma	Antimetabolites	8.91	7.17	10.64
Melanoma	Biological response modifiers including immunotherapy	31.56	28.72	34.39
Melanoma	Hormonal agents	12.68	10.65	14.71
Melanoma	Kinase inhibitors	12.49	10.47	14.50
Melanoma	Non-anthracycline antitumour antibiotics	1.74	0.94	2.54
Melanoma	Plant alkaloids excluding vinca alkaloids	3.68	2.53	4.83
Melanoma	Platinum agents	8.42	6.73	10.12
Melanoma	Vinca alkaloid	3.29	2.20	4.38
Oesophagus	Alkylating agents	0.32	0.16	0.48
Oesophagus	Anthracyclines	17.80	16.74	18.86
Oesophagus	Antimetabolites	28.56	27.31	29.81
Oesophagus	Biological response modifiers including immunotherapy	2.33	1.92	2.75
Oesophagus	Hormonal agents	13.13	12.20	14.07
Oesophagus	Kinase inhibitors	0.18	0.06	0.30
Oesophagus	Non-anthracycline antitumour antibiotics	0.22	0.09	0.35
Oesophagus	Plant alkaloids excluding vinca alkaloids	4.93	4.33	5.53
Oesophagus	Platinum agents	32.39	31.09	33.68
Oesophagus	Vinca alkaloid	0.14	0.04	0.24
Oropharynx	Alkylating agents	0.91	0.52	1.29
Oropharynx	Anthracyclines	1.21	0.76	1.66
Oropharynx	Antimetabolites	16.77	15.25	18.30
Oropharynx	Biological response modifiers including immunotherapy	9.12	7.95	10.30
Oropharynx	Hormonal agents	18.94	17.34	20.53
Oropharynx	Kinase inhibitors	0.56	0.26	0.87
Oropharynx	Non-anthracycline antitumour antibiotics	0.22	0.03	0.41
Oropharynx	Plant alkaloids excluding vinca alkaloids	7.74	6.65	8.83
Oropharynx	Platinum agents	43.62	41.60	45.64
Oropharynx	Vinca alkaloid	0.91	0.52	1.29
Ovary	Alkylating agents	1.34	1.01	1.67
Ovary	Anthracyclines	9.05	8.23	9.87
Ovary	Antimetabolites	6.45	5.75	7.15
Ovary	Biological response modifiers including immunotherapy	6.58	5.87	7.29
Ovary	Hormonal agents	15.03	14.01	16.05
Ovary	Kinase inhibitors	0.11	0.01	0.20
Ovary	Non-anthracycline antitumour antibiotics	0.13	0.03	0.23
Ovary	Plant alkaloids excluding vinca alkaloids	23.56	22.35	24.78
Ovary	Platinum agents	37.65	36.27	39.04
Ovary	Vinca alkaloid	0.11	0.01	0.20
Pancreas	Alkylating agents	0.43	0.15	0.71
Pancreas	Anthracyclines	0.38	0.12	0.64
Pancreas	Antimetabolites	54.16	52.03	56.29
Pancreas	Biological response modifiers including immunotherapy	0.90	0.50	1.31
Pancreas	Hormonal agents	10.60	9.29	11.92
Pancreas	Kinase inhibitors	1.00	0.57	1.42
Pancreas	Non-anthracycline antitumour antibiotics	0.33	0.09	0.58
Pancreas	Plant alkaloids excluding vinca alkaloids	15.36	13.82	16.90
Pancreas	Platinum agents	16.74	15.14	18.33
Pancreas	Vinca alkaloid	0.10	0.00	0.23
Prostate	Alkylating agents	1.33	1.12	1.54
Prostate	Anthracyclines	0.88	0.71	1.05
Prostate	Antimetabolites	3.68	3.33	4.02
Prostate	Biological response modifiers including immunotherapy	1.80	1.55	2.04
Prostate	Hormonal agents	77.31	76.55	78.07
Prostate	Kinase inhibitors	0.41	0.30	0.53
Prostate	Non-anthracycline antitumour antibiotics	0.63	0.48	0.77
Prostate	Plant alkaloids excluding vinca alkaloids	9.75	9.21	10.29
Prostate	Platinum agents	3.32	3.00	3.65
Prostate	Vinca alkaloid	0.89	0.72	1.06
Stomach	Alkylating agents	0.47	0.22	0.72
Stomach	Anthracyclines	21.28	19.81	22.76
Stomach	Antimetabolites	27.67	26.07	29.28
Stomach	Biological response modifiers including immunotherapy	2.49	1.93	3.05
Stomach	Hormonal agents	12.31	11.13	13.49
Stomach	Kinase inhibitors	1.61	1.16	2.07
Stomach	Non-anthracycline antitumour antibiotics	0.37	0.15	0.59
Stomach	Plant alkaloids excluding vinca alkaloids	4.54	3.79	5.29
Stomach	Platinum agents	29.02	27.39	30.65
Stomach	Vinca alkaloid	0.24	0.06	0.41
Testis	Alkylating agents	1.70	0.94	2.45
Testis	Anthracyclines	0.36	0.01	0.71
Testis	Antimetabolites	1.52	0.80	2.24
Testis	Biological response modifiers including immunotherapy	0.36	0.01	0.71
Testis	Hormonal agents	14.75	12.67	16.82
Testis	Kinase inhibitors	0.18	0.00	0.43
Testis	Non-anthracycline antitumour antibiotics	16.53	14.36	18.71
Testis	Plant alkaloids excluding vinca alkaloids	20.29	17.93	22.64
Testis	Platinum agents	43.70	40.79	46.61
Testis	Vinca alkaloid	0.63	0.16	1.09
Thyroid	Alkylating agents	3.21	0.44	5.97
Thyroid	Anthracyclines	7.69	3.51	11.87
Thyroid	Antimetabolites	10.26	5.50	15.02
Thyroid	Biological response modifiers including immunotherapy	8.33	4.00	12.67
Thyroid	Hormonal agents	38.46	30.83	46.10
Thyroid	Kinase inhibitors	7.05	3.03	11.07
Thyroid	Non-anthracycline antitumour antibiotics	0.64	0.00	1.89
Thyroid	Plant alkaloids excluding vinca alkaloids	9.62	4.99	14.24
Thyroid	Platinum agents	13.46	8.11	18.82
Thyroid	Vinca alkaloid	1.28	0.00	3.05
Uterus	Alkylating agents	2.53	1.83	3.23
Uterus	Anthracyclines	7.23	6.07	8.38
Uterus	Antimetabolites	5.11	4.13	6.09
Uterus	Biological response modifiers including immunotherapy	3.30	2.51	4.10
Uterus	Hormonal agents	20.75	18.95	22.56
Uterus	Kinase inhibitors	0.36	0.09	0.63
Uterus	Non-anthracycline antitumour antibiotics	0.31	0.06	0.56
Uterus	Plant alkaloids excluding vinca alkaloids	25.81	23.86	27.76
Uterus	Platinum agents	33.87	31.76	35.97
Uterus	Vinca alkaloid	0.72	0.35	1.10

Table S5. List of 109 health conditions categorised by organ system.

Condition	Organ system
Abdominal aortic aneurysm	Cardiovascular
Atrial fibrillation	Cardiovascular
Atrioventricular block, complete	Cardiovascular
Atrioventricular block, first degree	Cardiovascular
Atrioventricular block, second degree	Cardiovascular
Bifascicular block	Cardiovascular
Coronary heart disease not otherwise specified	Cardiovascular
Dilated cardiomyopathy	Cardiovascular
Heart failure	Cardiovascular
Hypertension	Cardiovascular
Hypertrophic cardiomyopathy	Cardiovascular
Ischaemic stroke	Cardiovascular
Left bundle branch block	Cardiovascular
Multiple valve disease	Cardiovascular
Myocardial infarction	Cardiovascular
Nonrheumatic aortic valve disorders	Cardiovascular
Nonrheumatic mitral valve disorders	Cardiovascular
Other cardiomyopathy	Cardiovascular
Pericardial effusion (noninflammatory)	Cardiovascular
Peripheral arterial disease	Cardiovascular
Primary pulmonary hypertension	Cardiovascular
Raynaud's syndrome	Cardiovascular
Right bundle branch block	Cardiovascular
Secondary pulmonary hypertension	Cardiovascular
Sick sinus syndrome	Cardiovascular
Stable angina	Cardiovascular
Stroke (not otherwise specified)	Cardiovascular
Supraventricular tachycardia	Cardiovascular
Transient ischaemic attack	Cardiovascular
Trifascicular block	Cardiovascular
Unstable angina	Cardiovascular
Venous thromboembolic disease	Cardiovascular
Ventricular tachycardia	Cardiovascular
Diabetes	Endocrine
Diabetic neurological complications	Endocrine
Diabetic ophthalmic complications	Endocrine
Hyperparathyroidism	Endocrine
Hypo or hyperthyroidism	Endocrine
Alcoholic liver disease	Gastrointestinal
Angiodysplasia of colon	Gastrointestinal
Barrett's oesophagus	Gastrointestinal
Cholangitis	Gastrointestinal
Cholecystitis	Gastrointestinal
Cholelithiasis	Gastrointestinal
Coeliac disease	Gastrointestinal
Crohn's disease	Gastrointestinal
Diverticular disease of intestine	Gastrointestinal
Fatty liver	Gastrointestinal
Gastritis and duodenitis	Gastrointestinal
Gastrooesophageal reflux disease	Gastrointestinal
Hepatic failure	Gastrointestinal
Liver fibrosis sclerosis and cirrhosis	Gastrointestinal
Oesophageal varices	Gastrointestinal
Oesophagitis and oesophageal ulcer	Gastrointestinal
Portal hypertension	Gastrointestinal
Ulcerative colitis	Gastrointestinal
Agranulocytosis	Haematological
Aplastic anaemias	Haematological
Other anaemias	Haematological
Other haemolytic anaemias	Haematological
Primary or idiopathic thrombocytopaenia	Haematological
Secondary or other thrombocytopaenia	Haematological
Secondary polycythaemia	Haematological
Thrombophilia	Haematological
Autoimmune liver disease	Immunology and infection
Chronic viral hepatitis	Immunology and infection
Encephalitis	Immunology and infection
HIV	Immunology and infection
Immunodeficiencies	Immunology and infection
Infection of anal and rectal regions	Immunology and infection
Infection of bones and joints	Immunology and infection
Infection of liver	Immunology and infection
Infection of skin and subcutaneous tissues	Immunology and infection
Infections of other or unspecified organs	Immunology and infection
Infections of the digestive system	Immunology and infection
Infections of the heart	Immunology and infection
Lower respiratory tract infections	Immunology and infection
Lupus erythematosus (local and systemic)	Immunology and infection
Meningitis	Immunology and infection
Non-acute cystitis	Immunology and infection
Peritonitis	Immunology and infection
Systemic sclerosis	Immunology and infection
Viral diseases (excluding chronic hepatitis/HIV)	Immunology and infection
Ankylosing spondylitis	Musculoskeletal
Enteropathic arthropathy	Musculoskeletal
Enthesopathies and synovial disorders	Musculoskeletal
Osteoarthritis (excluding spine)	Musculoskeletal
Osteoporosis	Musculoskeletal
Rheumatoid arthritis	Musculoskeletal
Scoliosis	Musculoskeletal
Spinal stenosis	Musculoskeletal
Bell's palsy	Neurological
Dementia	Neurological
Disorders of autonomic nervous system	Neurological
Epilepsy	Neurological
Essential tremor	Neurological
Intracerebral haemorrhage	Neurological
Intracranial hypertension	Neurological
Multiple sclerosis	Neurological
Peripheral neuropathies	Neurological
Subarachnoid haemorrhage	Neurological
Asthma	Pulmonary
Bronchiectasis	Pulmonary
COPD	Pulmonary
Other interstitial pulmonary diseases with fibrosis	Pulmonary
End stage renal disease	Renal
Glomerulonephritis	Renal
Obstructive and reflux uropathy	Renal
Urolithiasis	Renal

Table S6. Non-cancer conditions diagnosed, for the first time, in the 5-year period before cancer diagnosis. 95% confidence intervals (CIs) are shown.

Health condition	Organ system	Cancer type	Proportion (%)	Lower CI	Upper CI
Abdominal aortic aneurysm	Cardiovascular	Bladder	2.36	2.10	2.62
Atrial fibrillation	Cardiovascular	Bladder	6.39	5.98	6.81
Atrioventricular block, complete	Cardiovascular	Bladder	1.30	1.11	1.49
Atrioventricular block, first degree	Cardiovascular	Bladder	1.30	1.11	1.49
Atrioventricular block, second degree	Cardiovascular	Bladder	1.30	1.11	1.49
Bifascicular block	Cardiovascular	Bladder	0.78	0.63	0.93
Coronary heart disease not otherwise specified	Cardiovascular	Bladder	9.75	9.25	10.26
Dilated cardiomyopathy	Cardiovascular	Bladder	0.25	0.16	0.33
Heart failure	Cardiovascular	Bladder	5.46	5.07	5.84
Hypertension	Cardiovascular	Bladder	16.42	15.79	17.06
Hypertrophic cardiomyopathy	Cardiovascular	Bladder	0.24	0.16	0.33
Ischaemic stroke	Cardiovascular	Bladder	1.76	1.54	1.99
Left bundle branch block	Cardiovascular	Bladder	1.35	1.15	1.54
Multiple valve disease	Cardiovascular	Bladder	0.68	0.54	0.82
Myocardial infarction	Cardiovascular	Bladder	6.80	6.37	7.22
Nonrheumatic aortic valve disorders	Cardiovascular	Bladder	1.21	1.02	1.40
Nonrheumatic mitral valve disorders	Cardiovascular	Bladder	0.80	0.65	0.95
Other cardiomyopathy	Cardiovascular	Bladder	8.16	7.69	8.62
Pericardial effusion (noninflammatory)	Cardiovascular	Bladder	0.13	0.07	0.19
Peripheral arterial disease	Cardiovascular	Bladder	2.77	2.49	3.05
Primary pulmonary hypertension	Cardiovascular	Bladder	0.33	0.23	0.43
Raynaud's syndrome	Cardiovascular	Bladder	1.91	1.68	2.15
Right bundle branch block	Cardiovascular	Bladder	0.78	0.63	0.93
Secondary pulmonary hypertension	Cardiovascular	Bladder	0.33	0.23	0.42
Sick sinus syndrome	Cardiovascular	Bladder	0.60	0.47	0.74
Stable angina	Cardiovascular	Bladder	5.04	4.67	5.41
Stroke (not otherwise specified)	Cardiovascular	Bladder	2.48	2.21	2.74
Supraventricular tachycardia	Cardiovascular	Bladder	0.67	0.53	0.80
Transient ischaemic attack	Cardiovascular	Bladder	3.34	3.03	3.65
Trifascicular block	Cardiovascular	Bladder	0.70	0.56	0.85
Unstable angina	Cardiovascular	Bladder	5.80	5.40	6.20
Venous thromboembolic disease	Cardiovascular	Bladder	2.96	2.67	3.25
Ventricular tachycardia	Cardiovascular	Bladder	0.66	0.52	0.80
Diabetes	Endocrine	Bladder	7.20	6.76	7.64
Diabetic neurological complications	Endocrine	Bladder	7.35	6.90	7.79
Diabetic ophthalmic complications	Endocrine	Bladder	7.96	7.50	8.42
Hyperparathyroidism	Endocrine	Bladder	0.14	0.07	0.20
Hypo or hyperthyroidism	Endocrine	Bladder	2.15	1.91	2.40
Alcoholic liver disease	Gastrointestinal	Bladder	0.34	0.24	0.44
Angiodysplasia of colon	Gastrointestinal	Bladder	0.16	0.09	0.23
Barrett's oesophagus	Gastrointestinal	Bladder	1.11	0.93	1.29
Cholangitis	Gastrointestinal	Bladder	1.71	1.49	1.93
Cholecystitis	Gastrointestinal	Bladder	1.75	1.52	1.97
Cholelithiasis	Gastrointestinal	Bladder	2.00	1.76	2.24
Coeliac disease	Gastrointestinal	Bladder	0.18	0.11	0.25
Crohn's disease	Gastrointestinal	Bladder	0.11	0.06	0.17
Diverticular disease of intestine	Gastrointestinal	Bladder	3.79	3.47	4.12
Fatty liver	Gastrointestinal	Bladder	0.81	0.66	0.96
Gastritis and duodenitis	Gastrointestinal	Bladder	3.29	2.98	3.59
Gastrooesophageal reflux disease	Gastrointestinal	Bladder	3.30	2.99	3.60
Hepatic failure	Gastrointestinal	Bladder	0.63	0.50	0.77
Liver fibrosis sclerosis and cirrhosis	Gastrointestinal	Bladder	0.29	0.20	0.38
Oesophageal varices	Gastrointestinal	Bladder	0.08	0.03	0.12
Oesophagitis and oesophageal ulcer	Gastrointestinal	Bladder	4.27	3.93	4.62

Portal hypertension	Gastrointestinal	Bladder	0.45	0.34	0.57
Ulcerative colitis	Gastrointestinal	Bladder	0.21	0.13	0.29
Agranulocytosis	Haematological	Bladder	0.11	0.06	0.17
Aplastic anaemias	Haematological	Bladder	0.14	0.07	0.20
Other anaemias	Haematological	Bladder	6.08	5.67	6.48
Other haemolytic anaemias	Haematological	Bladder	0.05	0.01	0.08
Primary or idiopathic thrombocytopaenia	Haematological	Bladder	0.32	0.22	0.41
Secondary or other thrombocytopaenia	Haematological	Bladder	0.55	0.43	0.68
Secondary polycythaemia	Haematological	Bladder	0.11	0.05	0.16
Thrombophilia	Haematological	Bladder	0.21	0.13	0.29
Autoimmune liver disease	Immunology and infection	Bladder	0.14	0.07	0.20
Chronic viral hepatitis	Immunology and infection	Bladder	0.13	0.07	0.19
Encephalitis	Immunology and infection	Bladder	0.09	0.04	0.14
HIV	Immunology and infection	Bladder	0.04	0.00	0.07
Immunodeficiencies	Immunology and infection	Bladder	0.02	0.00	0.05
Infection of anal and rectal regions	Immunology and infection	Bladder	0.09	0.04	0.14
Infection of bones and joints	Immunology and infection	Bladder	0.39	0.28	0.49
Infection of liver	Immunology and infection	Bladder	0.08	0.03	0.12
Infection of skin and subcutaneous tissues	Immunology and infection	Bladder	2.22	1.97	2.47
Infections of other or unspecified organs	Immunology and infection	Bladder	5.28	4.89	5.66
Infections of the digestive system	Immunology and infection	Bladder	2.12	1.87	2.36
Infections of the heart	Immunology and infection	Bladder	0.37	0.27	0.47
Lower respiratory tract infections	Immunology and infection	Bladder	8.17	7.70	8.64
Lupus erythematosus (local and systemic)	Immunology and infection	Bladder	0.06	0.02	0.10
Meningitis	Immunology and infection	Bladder	0.44	0.33	0.55
Non-acute cystitis	Immunology and infection	Bladder	3.11	2.81	3.40
Peritonitis	Immunology and infection	Bladder	5.29	4.91	5.67
Systemic sclerosis	Immunology and infection	Bladder	0.02	0.00	0.05
Viral diseases (excluding chronic hepatitis/HIV)	Immunology and infection	Bladder	0.53	0.41	0.65
Ankylosing spondylitis	Musculoskeletal	Bladder	0.04	0.00	0.07
Enteropathic arthropathy	Musculoskeletal	Bladder	0.15	0.08	0.22
Enthesopathies and synovial disorders	Musculoskeletal	Bladder	4.38	4.03	4.73
Osteoarthritis (excluding spine)	Musculoskeletal	Bladder	7.56	7.11	8.01
Osteoporosis	Musculoskeletal	Bladder	2.69	2.42	2.97
Rheumatoid arthritis	Musculoskeletal	Bladder	0.69	0.55	0.83
Scoliosis	Musculoskeletal	Bladder	0.26	0.18	0.35
Spinal stenosis	Musculoskeletal	Bladder	0.63	0.49	0.76
Bell's palsy	Neurological	Bladder	0.19	0.11	0.26
Dementia	Neurological	Bladder	1.83	1.60	2.06
Disorders of autonomic nervous system	Neurological	Bladder	0.23	0.15	0.32
Epilepsy	Neurological	Bladder	0.48	0.36	0.59
Essential tremor	Neurological	Bladder	0.18	0.11	0.25
Intracerebral haemorrhage	Neurological	Bladder	0.82	0.66	0.97
Intracranial hypertension	Neurological	Bladder	0.08	0.03	0.12
Multiple sclerosis	Neurological	Bladder	0.05	0.01	0.09
Peripheral neuropathies	Neurological	Bladder	1.97	1.74	2.21
Subarachnoid haemorrhage	Neurological	Bladder	0.68	0.54	0.82
Asthma	Pulmonary	Bladder	3.10	2.80	3.39
Bronchiectasis	Pulmonary	Bladder	0.33	0.23	0.42
COPD	Pulmonary	Bladder	5.98	5.57	6.38
Other interstitial pulmonary diseases with fibrosis	Pulmonary	Bladder	0.36	0.26	0.47
End stage renal disease	Renal	Bladder	3.91	3.58	4.24
Glomerulonephritis	Renal	Bladder	1.83	1.60	2.06
Obstructive and reflux uropathy	Renal	Bladder	6.59	6.17	7.01
Urolithiasis	Renal	Bladder	7.97	7.51	8.44
Abdominal aortic aneurysm	Cardiovascular	Brain	0.90	0.62	1.18
Atrial fibrillation	Cardiovascular	Brain	2.64	2.17	3.12
Atrioventricular block, complete	Cardiovascular	Brain	0.70	0.45	0.95
Atrioventricular block, first degree	Cardiovascular	Brain	0.70	0.45	0.95
Atrioventricular block, second degree	Cardiovascular	Brain	0.70	0.45	0.95
Bifascicular block	Cardiovascular	Brain	0.72	0.47	0.97
Coronary heart disease not otherwise specified	Cardiovascular	Brain	6.53	5.80	7.26

Dilated cardiomyopathy	Cardiovascular	Brain	0.18	0.06	0.31
Heart failure	Cardiovascular	Brain	1.08	0.78	1.39
Hypertension	Cardiovascular	Brain	13.65	12.64	14.66
Hypertrophic cardiomyopathy	Cardiovascular	Brain	0.16	0.04	0.28
Ischaemic stroke	Cardiovascular	Brain	3.30	2.77	3.82
Left bundle branch block	Cardiovascular	Brain	0.79	0.53	1.05
Multiple valve disease	Cardiovascular	Brain	0.18	0.06	0.31
Myocardial infarction	Cardiovascular	Brain	3.05	2.54	3.56
Nonrheumatic aortic valve disorders	Cardiovascular	Brain	0.54	0.33	0.76
Nonrheumatic mitral valve disorders	Cardiovascular	Brain	0.38	0.20	0.57
Other cardiomyopathy	Cardiovascular	Brain	3.50	2.96	4.04
Pericardial effusion (noninflammatory)	Cardiovascular	Brain	0.07	0.00	0.14
Peripheral arterial disease	Cardiovascular	Brain	0.86	0.59	1.13
Primary pulmonary hypertension	Cardiovascular	Brain	0.07	0.00	0.14
Raynaud's syndrome	Cardiovascular	Brain	0.79	0.53	1.05
Right bundle branch block	Cardiovascular	Brain	0.72	0.47	0.97
Secondary pulmonary hypertension	Cardiovascular	Brain	0.07	0.00	0.14
Sick sinus syndrome	Cardiovascular	Brain	0.38	0.20	0.57
Stable angina	Cardiovascular	Brain	2.55	2.09	3.02
Stroke (not otherwise specified)	Cardiovascular	Brain	5.08	4.44	5.73
Supraventricular tachycardia	Cardiovascular	Brain	0.50	0.29	0.70
Transient ischaemic attack	Cardiovascular	Brain	4.88	4.25	5.52
Trifascicular block	Cardiovascular	Brain	0.63	0.40	0.87
Unstable angina	Cardiovascular	Brain	2.91	2.42	3.41
Venous thromboembolic disease	Cardiovascular	Brain	4.68	4.05	5.30
Ventricular tachycardia	Cardiovascular	Brain	0.52	0.31	0.73
Diabetes	Endocrine	Brain	5.96	5.27	6.66
Diabetic neurological complications	Endocrine	Brain	5.20	4.54	5.85
Diabetic ophthalmic complications	Endocrine	Brain	5.35	4.69	6.02
Hyperparathyroidism	Endocrine	Brain	0.11	0.01	0.21
Hypo or hyperthyroidism	Endocrine	Brain	2.82	2.34	3.31
Alcoholic liver disease	Gastrointestinal	Brain	0.20	0.07	0.34
Angiodysplasia of colon	Gastrointestinal	Brain	0.05	0.00	0.11
Barrett's oesophagus	Gastrointestinal	Brain	0.88	0.61	1.16
Cholangitis	Gastrointestinal	Brain	1.49	1.13	1.85
Cholecystitis	Gastrointestinal	Brain	1.63	1.25	2.00
Cholelithiasis	Gastrointestinal	Brain	1.58	1.21	1.95
Coeliac disease	Gastrointestinal	Brain	0.36	0.18	0.54
Crohn's disease	Gastrointestinal	Brain	0.09	0.00	0.18
Diverticular disease of intestine	Gastrointestinal	Brain	3.25	2.73	3.78
Fatty liver	Gastrointestinal	Brain	1.45	1.09	1.80
Gastritis and duodenitis	Gastrointestinal	Brain	2.71	2.23	3.19
Gastrooesophageal reflux disease	Gastrointestinal	Brain	3.64	3.09	4.19
Hepatic failure	Gastrointestinal	Brain	1.31	0.98	1.65
Liver fibrosis sclerosis and cirrhosis	Gastrointestinal	Brain	0.16	0.04	0.28
Oesophagitis and oesophageal ulcer	Gastrointestinal	Brain	4.27	3.67	4.87
Portal hypertension	Gastrointestinal	Brain	1.15	0.84	1.47
Ulcerative colitis	Gastrointestinal	Brain	0.23	0.09	0.37
Agranulocytosis	Haematological	Brain	0.09	0.00	0.18
Aplastic anaemias	Haematological	Brain	0.02	0.00	0.07
Other anaemias	Haematological	Brain	1.45	1.09	1.80
Other haemolytic anaemias	Haematological	Brain	0.02	0.00	0.07
Primary or idiopathic thrombocytopaenia	Haematological	Brain	0.29	0.13	0.45
Secondary or other thrombocytopaenia	Haematological	Brain	0.38	0.20	0.57
Secondary polycythaemia	Haematological	Brain	0.09	0.00	0.18
Thrombophilia	Haematological	Brain	0.14	0.03	0.24
Autoimmune liver disease	Immunology and infection	Brain	0.20	0.07	0.34
Chronic viral hepatitis	Immunology and infection	Brain	0.38	0.20	0.57
Encephalitis	Immunology and infection	Brain	0.52	0.31	0.73
HIV	Immunology and infection	Brain	0.29	0.13	0.45
Infection of anal and rectal regions	Immunology and infection	Brain	0.07	0.00	0.14
Infection of bones and joints	Immunology and infection	Brain	0.18	0.06	0.31

Infection of liver	Immunology and infection	Brain	0.29	0.13	0.45
Infection of skin and subcutaneous tissues	Immunology and infection	Brain	1.40	1.05	1.75
Infections of other or unspecified organs	Immunology and infection	Brain	4.09	3.51	4.67
Infections of the digestive system	Immunology and infection	Brain	1.78	1.39	2.17
Infections of the heart	Immunology and infection	Brain	0.43	0.24	0.62
Lower respiratory tract infections	Immunology and infection	Brain	4.27	3.67	4.87
Lupus erythematosus (local and systemic)	Immunology and infection	Brain	0.02	0.00	0.07
Meningitis	Immunology and infection	Brain	0.56	0.34	0.79
Non-acute cystitis	Immunology and infection	Brain	0.38	0.20	0.57
Peritonitis	Immunology and infection	Brain	4.20	3.61	4.79
Systemic sclerosis	Immunology and infection	Brain	0.02	0.00	0.07
Viral diseases (excluding chronic hepatitis/HIV)	Immunology and infection	Brain	1.47	1.11	1.82
Ankylosing spondylitis	Musculoskeletal	Brain	0.07	0.00	0.14
Enteropathic arthropathy	Musculoskeletal	Brain	0.25	0.10	0.40
Enthesopathies and synovial disorders	Musculoskeletal	Brain	5.87	5.18	6.57
Osteoarthritis (excluding spine)	Musculoskeletal	Brain	5.24	4.59	5.90
Osteoporosis	Musculoskeletal	Brain	1.51	1.15	1.87
Rheumatoid arthritis	Musculoskeletal	Brain	0.50	0.29	0.70
Scoliosis	Musculoskeletal	Brain	0.14	0.03	0.24
Spinal stenosis	Musculoskeletal	Brain	0.47	0.27	0.68
Bell's palsy	Neurological	Brain	1.17	0.86	1.49
Dementia	Neurological	Brain	0.52	0.31	0.73
Disorders of autonomic nervous system	Neurological	Brain	0.50	0.29	0.70
Epilepsy	Neurological	Brain	10.96	10.04	11.88
Essential tremor	Neurological	Brain	0.25	0.10	0.40
Intracerebral haemorrhage	Neurological	Brain	2.94	2.44	3.43
Intracranial hypertension	Neurological	Brain	14.03	13.01	15.05
Multiple sclerosis	Neurological	Brain	0.07	0.00	0.14
Peripheral neuropathies	Neurological	Brain	1.58	1.21	1.95
Subarachnoid haemorrhage	Neurological	Brain	0.93	0.64	1.21
Asthma	Pulmonary	Brain	2.91	2.42	3.41
Bronchiectasis	Pulmonary	Brain	0.34	0.17	0.51
COPD	Pulmonary	Brain	2.55	2.09	3.02
Other interstitial pulmonary diseases with fibrosis	Pulmonary	Brain	0.14	0.03	0.24
End stage renal disease	Renal	Brain	1.13	0.82	1.44
Glomerulonephritis	Renal	Brain	0.34	0.17	0.51
Obstructive and reflux uropathy	Renal	Brain	0.36	0.18	0.54
Urolithiasis	Renal	Brain	1.02	0.72	1.31
Abdominal aortic aneurysm	Cardiovascular	Breast	0.19	0.16	0.22
Atrial fibrillation	Cardiovascular	Breast	2.24	2.13	2.35
Atrioventricular block, complete	Cardiovascular	Breast	0.35	0.31	0.40
Atrioventricular block, first degree	Cardiovascular	Breast	0.35	0.31	0.40
Atrioventricular block, second degree	Cardiovascular	Breast	0.35	0.31	0.40
Bifascicular block	Cardiovascular	Breast	0.20	0.16	0.23
Coronary heart disease not otherwise specified	Cardiovascular	Breast	5.64	5.46	5.81
Dilated cardiomyopathy	Cardiovascular	Breast	0.06	0.04	0.07
Heart failure	Cardiovascular	Breast	1.57	1.47	1.66
Hypertension	Cardiovascular	Breast	9.34	9.12	9.56
Hypertrophic cardiomyopathy	Cardiovascular	Breast	0.06	0.04	0.08
Ischaemic stroke	Cardiovascular	Breast	0.50	0.45	0.56
Left bundle branch block	Cardiovascular	Breast	0.39	0.35	0.44
Multiple valve disease	Cardiovascular	Breast	0.32	0.27	0.36
Myocardial infarction	Cardiovascular	Breast	1.89	1.78	1.99
Nonrheumatic aortic valve disorders	Cardiovascular	Breast	0.47	0.42	0.53
Nonrheumatic mitral valve disorders	Cardiovascular	Breast	0.43	0.38	0.48
Other cardiomyopathy	Cardiovascular	Breast	1.92	1.82	2.02
Pericardial effusion (noninflammatory)	Cardiovascular	Breast	0.07	0.05	0.09
Peripheral arterial disease	Cardiovascular	Breast	0.66	0.59	0.72
Primary pulmonary hypertension	Cardiovascular	Breast	0.11	0.09	0.14
Raynaud's syndrome	Cardiovascular	Breast	0.55	0.49	0.60
Right bundle branch block	Cardiovascular	Breast	0.19	0.16	0.23
Secondary pulmonary hypertension	Cardiovascular	Breast	0.10	0.07	0.12

Sick sinus syndrome	Cardiovascular	Breast	0.22	0.19	0.26
Stable angina	Cardiovascular	Breast	1.85	1.75	1.95
Stroke (not otherwise specified)	Cardiovascular	Breast	0.93	0.86	1.01
Supraventricular tachycardia	Cardiovascular	Breast	0.36	0.32	0.41
Transient ischaemic attack	Cardiovascular	Breast	1.30	1.21	1.38
Trifascicular block	Cardiovascular	Breast	0.16	0.13	0.19
Unstable angina	Cardiovascular	Breast	1.68	1.58	1.77
Venous thromboembolic disease	Cardiovascular	Breast	1.27	1.18	1.36
Ventricular tachycardia	Cardiovascular	Breast	0.35	0.31	0.40
Diabetes	Endocrine	Breast	4.80	4.64	4.96
Diabetic neurological complications	Endocrine	Breast	2.31	2.20	2.43
Diabetic ophthalmic complications	Endocrine	Breast	2.96	2.83	3.09
Hyperparathyroidism	Endocrine	Breast	0.16	0.13	0.19
Hypo or hyperthyroidism	Endocrine	Breast	2.59	2.47	2.71
Alcoholic liver disease	Gastrointestinal	Breast	0.19	0.16	0.22
Angiodysplasia of colon	Gastrointestinal	Breast	0.08	0.05	0.10
Barrett's oesophagus	Gastrointestinal	Breast	0.58	0.53	0.64
Cholangitis	Gastrointestinal	Breast	1.40	1.31	1.49
Cholecystitis	Gastrointestinal	Breast	1.50	1.40	1.59
Cholelithiasis	Gastrointestinal	Breast	1.77	1.67	1.87
Coeliac disease	Gastrointestinal	Breast	0.27	0.23	0.31
Crohn's disease	Gastrointestinal	Breast	0.11	0.08	0.13
Diverticular disease of intestine	Gastrointestinal	Breast	2.60	2.47	2.72
Fatty liver	Gastrointestinal	Breast	0.42	0.37	0.47
Gastritis and duodenitis	Gastrointestinal	Breast	2.25	2.14	2.36
Gastrooesophageal reflux disease	Gastrointestinal	Breast	2.87	2.74	3.00
Hepatic failure	Gastrointestinal	Breast	0.31	0.27	0.36
Liver fibrosis sclerosis and cirrhosis	Gastrointestinal	Breast	0.11	0.09	0.14
Oesophageal varices	Gastrointestinal	Breast	0.03	0.02	0.05
Oesophagitis and oesophageal ulcer	Gastrointestinal	Breast	3.19	3.06	3.33
Portal hypertension	Gastrointestinal	Breast	0.21	0.18	0.25
Ulcerative colitis	Gastrointestinal	Breast	0.16	0.13	0.19
Agranulocytosis	Haematological	Breast	0.12	0.10	0.15
Aplastic anaemias	Haematological	Breast	0.02	0.01	0.03
Other anaemias	Haematological	Breast	2.02	1.91	2.13
Other haemolytic anaemias	Haematological	Breast	0.02	0.01	0.04
Primary or idiopathic thrombocytopaenia	Haematological	Breast	0.08	0.06	0.11
Secondary or other thrombocytopaenia	Haematological	Breast	0.16	0.13	0.19
Secondary polycythaemia	Haematological	Breast	0.05	0.03	0.07
Thrombophilia	Haematological	Breast	0.12	0.10	0.15
Autoimmune liver disease	Immunology and infection	Breast	0.08	0.06	0.10
Chronic viral hepatitis	Immunology and infection	Breast	0.39	0.34	0.43
Encephalitis	Immunology and infection	Breast	0.06	0.04	0.08
HIV	Immunology and infection	Breast	0.15	0.12	0.18
Immunodeficiencies	Immunology and infection	Breast	0.02	0.01	0.02
Infection of anal and rectal regions	Immunology and infection	Breast	0.05	0.03	0.07
Infection of bones and joints	Immunology and infection	Breast	0.29	0.25	0.33
Infection of liver	Immunology and infection	Breast	0.07	0.05	0.09
Infection of skin and subcutaneous tissues	Immunology and infection	Breast	1.18	1.10	1.26
Infections of other or unspecified organs	Immunology and infection	Breast	2.15	2.04	2.26
Infections of the digestive system	Immunology and infection	Breast	1.21	1.13	1.29
Infections of the heart	Immunology and infection	Breast	0.23	0.20	0.27
Lower respiratory tract infections	Immunology and infection	Breast	2.57	2.45	2.69
Lupus erythematosus (local and systemic)	Immunology and infection	Breast	0.06	0.04	0.08
Meningitis	Immunology and infection	Breast	0.26	0.22	0.30
Non-acute cystitis	Immunology and infection	Breast	0.25	0.21	0.29
Peritonitis	Immunology and infection	Breast	3.01	2.88	3.14
Systemic sclerosis	Immunology and infection	Breast	0.03	0.02	0.05
Viral diseases (excluding chronic hepatitis/HIV)	Immunology and infection	Breast	0.49	0.44	0.54
Ankylosing spondylitis	Musculoskeletal	Breast	0.03	0.02	0.05
Enteropathic arthropathy	Musculoskeletal	Breast	0.12	0.09	0.15
Enthesopathies and synovial disorders	Musculoskeletal	Breast	5.47	5.30	5.64

Osteoarthritis (excluding spine)	Musculoskeletal	Breast	6.77	6.58	6.96
Osteoporosis	Musculoskeletal	Breast	2.16	2.05	2.28
Rheumatoid arthritis	Musculoskeletal	Breast	0.57	0.51	0.62
Scoliosis	Musculoskeletal	Breast	0.24	0.20	0.28
Spinal stenosis	Musculoskeletal	Breast	0.42	0.37	0.46
Bell's palsy	Neurological	Breast	0.16	0.13	0.19
Dementia	Neurological	Breast	0.80	0.73	0.87
Disorders of autonomic nervous system	Neurological	Breast	0.22	0.18	0.26
Epilepsy	Neurological	Breast	0.25	0.22	0.29
Essential tremor	Neurological	Breast	0.12	0.09	0.14
Intracerebral haemorrhage	Neurological	Breast	0.26	0.22	0.30
Intracranial hypertension	Neurological	Breast	0.10	0.08	0.12
Multiple sclerosis	Neurological	Breast	0.08	0.06	0.10
Peripheral neuropathies	Neurological	Breast	1.63	1.53	1.73
Subarachnoid haemorrhage	Neurological	Breast	0.26	0.23	0.30
Asthma	Pulmonary	Breast	2.59	2.47	2.71
Bronchiectasis	Pulmonary	Breast	0.20	0.16	0.23
COPD	Pulmonary	Breast	2.26	2.15	2.37
Other interstitial pulmonary diseases with fibrosis	Pulmonary	Breast	0.08	0.06	0.10
End stage renal disease	Renal	Breast	0.84	0.77	0.91
Glomerulonephritis	Renal	Breast	0.24	0.20	0.28
Obstructive and reflux uropathy	Renal	Breast	0.17	0.14	0.20
Urolithiasis	Renal	Breast	0.47	0.41	0.52
Abdominal aortic aneurysm	Cardiovascular	Cervix	0.03	0.00	0.09
Atrial fibrillation	Cardiovascular	Cervix	1.07	0.73	1.41
Atrioventricular block, complete	Cardiovascular	Cervix	0.09	0.00	0.18
Atrioventricular block, first degree	Cardiovascular	Cervix	0.09	0.00	0.18
Atrioventricular block, second degree	Cardiovascular	Cervix	0.09	0.00	0.18
Bifascicular block	Cardiovascular	Cervix	0.17	0.03	0.31
Coronary heart disease not otherwise specified	Cardiovascular	Cervix	5.14	4.40	5.88
Dilated cardiomyopathy	Cardiovascular	Cervix	0.06	0.00	0.14
Heart failure	Cardiovascular	Cervix	1.01	0.68	1.34
Hypertension	Cardiovascular	Cervix	5.31	4.57	6.06
Hypertrophic cardiomyopathy	Cardiovascular	Cervix	0.09	0.00	0.18
Ischaemic stroke	Cardiovascular	Cervix	0.43	0.21	0.65
Left bundle branch block	Cardiovascular	Cervix	0.12	0.00	0.23
Multiple valve disease	Cardiovascular	Cervix	0.20	0.05	0.35
Myocardial infarction	Cardiovascular	Cervix	1.41	1.02	1.81
Nonrheumatic aortic valve disorders	Cardiovascular	Cervix	0.26	0.09	0.43
Nonrheumatic mitral valve disorders	Cardiovascular	Cervix	0.17	0.03	0.31
Other cardiomyopathy	Cardiovascular	Cervix	1.50	1.10	1.91
Peripheral arterial disease	Cardiovascular	Cervix	0.69	0.42	0.97
Primary pulmonary hypertension	Cardiovascular	Cervix	0.03	0.00	0.09
Raynaud's syndrome	Cardiovascular	Cervix	0.46	0.24	0.69
Right bundle branch block	Cardiovascular	Cervix	0.17	0.03	0.31
Secondary pulmonary hypertension	Cardiovascular	Cervix	0.03	0.00	0.09
Sick sinus syndrome	Cardiovascular	Cervix	0.06	0.00	0.14
Stable angina	Cardiovascular	Cervix	1.21	0.85	1.58
Stroke (not otherwise specified)	Cardiovascular	Cervix	0.78	0.49	1.07
Supraventricular tachycardia	Cardiovascular	Cervix	0.29	0.11	0.47
Transient ischaemic attack	Cardiovascular	Cervix	0.95	0.63	1.28
Trifascicular block	Cardiovascular	Cervix	0.14	0.02	0.27
Unstable angina	Cardiovascular	Cervix	1.10	0.75	1.44
Venous thromboembolic disease	Cardiovascular	Cervix	1.16	0.80	1.51
Ventricular tachycardia	Cardiovascular	Cervix	0.26	0.09	0.43
Diabetes	Endocrine	Cervix	3.90	3.25	4.54
Diabetic neurological complications	Endocrine	Cervix	1.56	1.15	1.97
Diabetic ophthalmic complications	Endocrine	Cervix	1.93	1.48	2.39
Hyperparathyroidism	Endocrine	Cervix	0.14	0.02	0.27
Hypo or hyperthyroidism	Endocrine	Cervix	1.91	1.45	2.36

Alcoholic liver disease	Gastrointestinal	Cervix	0.26	0.09	0.43
Angiodysplasia of colon	Gastrointestinal	Cervix	0.06	0.00	0.14
Barrett's oesophagus	Gastrointestinal	Cervix	0.32	0.13	0.51
Cholangitis	Gastrointestinal	Cervix	1.18	0.82	1.54
Cholecystitis	Gastrointestinal	Cervix	1.33	0.95	1.71
Cholelithiasis	Gastrointestinal	Cervix	1.47	1.07	1.87
Coeliac disease	Gastrointestinal	Cervix	0.17	0.03	0.31
Crohn's disease	Gastrointestinal	Cervix	0.23	0.07	0.39
Diverticular disease of intestine	Gastrointestinal	Cervix	1.47	1.07	1.87
Fatty liver	Gastrointestinal	Cervix	0.55	0.30	0.79
Gastritis and duodenitis	Gastrointestinal	Cervix	1.73	1.30	2.17
Gastrooesophageal reflux disease	Gastrointestinal	Cervix	1.65	1.22	2.07
Hepatic failure	Gastrointestinal	Cervix	0.52	0.28	0.76
Liver fibrosis sclerosis and cirrhosis	Gastrointestinal	Cervix	0.20	0.05	0.35
Oesophageal varices	Gastrointestinal	Cervix	0.06	0.00	0.14
Oesophagitis and oesophageal ulcer	Gastrointestinal	Cervix	1.79	1.35	2.23
Portal hypertension	Gastrointestinal	Cervix	0.32	0.13	0.51
Ulcerative colitis	Gastrointestinal	Cervix	0.09	0.00	0.18
Agranulocytosis	Haematological	Cervix	0.17	0.03	0.31
Other anaemias	Haematological	Cervix	3.32	2.72	3.92
Other haemolytic anaemias	Haematological	Cervix	0.03	0.00	0.09
Primary or idiopathic thrombocytopaenia	Haematological	Cervix	0.17	0.03	0.31
Secondary or other thrombocytopaenia	Haematological	Cervix	0.20	0.05	0.35
Secondary polycythaemia	Haematological	Cervix	0.03	0.00	0.09
Thrombophilia	Haematological	Cervix	0.32	0.13	0.51
Autoimmune liver disease	Immunology and infection	Cervix	0.06	0.00	0.14
Chronic viral hepatitis	Immunology and infection	Cervix	1.10	0.75	1.44
Encephalitis	Immunology and infection	Cervix	0.06	0.00	0.14
HIV	Immunology and infection	Cervix	0.90	0.58	1.21
Infection of anal and rectal regions	Immunology and infection	Cervix	0.20	0.05	0.35
Infection of bones and joints	Immunology and infection	Cervix	0.12	0.00	0.23
Infection of liver	Immunology and infection	Cervix	0.14	0.02	0.27
Infection of skin and subcutaneous tissues	Immunology and infection	Cervix	1.04	0.70	1.38
Infections of other or unspecified organs	Immunology and infection	Cervix	2.51	1.99	3.03
Infections of the digestive system	Immunology and infection	Cervix	1.10	0.75	1.44
Infections of the heart	Immunology and infection	Cervix	0.29	0.11	0.47
Lower respiratory tract infections	Immunology and infection	Cervix	2.92	2.36	3.48
Lupus erythematosus (local and systemic)	Immunology and infection	Cervix	0.09	0.00	0.18
Meningitis	Immunology and infection	Cervix	0.32	0.13	0.51
Non-acute cystitis	Immunology and infection	Cervix	0.40	0.19	0.62
Peritonitis	Immunology and infection	Cervix	1.99	1.53	2.46
Viral diseases (excluding chronic hepatitis/HIV)	Immunology and infection	Cervix	0.75	0.46	1.04
Ankylosing spondylitis	Musculoskeletal	Cervix	0.06	0.00	0.14
Enteropathic arthropathy	Musculoskeletal	Cervix	0.09	0.00	0.18
Enthesopathies and synovial disorders	Musculoskeletal	Cervix	3.35	2.75	3.95
Osteoarthritis (excluding spine)	Musculoskeletal	Cervix	3.38	2.78	3.98
Osteoporosis	Musculoskeletal	Cervix	1.04	0.70	1.38
Rheumatoid arthritis	Musculoskeletal	Cervix	0.38	0.17	0.58
Scoliosis	Musculoskeletal	Cervix	0.14	0.02	0.27
Spinal stenosis	Musculoskeletal	Cervix	0.20	0.05	0.35
Bell's palsy	Neurological	Cervix	0.12	0.00	0.23
Dementia	Neurological	Cervix	0.49	0.26	0.72
Disorders of autonomic nervous system	Neurological	Cervix	0.09	0.00	0.18
Epilepsy	Neurological	Cervix	0.38	0.17	0.58
Essential tremor	Neurological	Cervix	0.03	0.00	0.09
Intracerebral haemorrhage	Neurological	Cervix	0.32	0.13	0.51
Intracranial hypertension	Neurological	Cervix	0.09	0.00	0.18
Multiple sclerosis	Neurological	Cervix	0.14	0.02	0.27
Peripheral neuropathies	Neurological	Cervix	0.84	0.53	1.14
Subarachnoid haemorrhage	Neurological	Cervix	0.23	0.07	0.39
Asthma	Pulmonary	Cervix	3.26	2.67	3.85
Bronchiectasis	Pulmonary	Cervix	0.06	0.00	0.14

COPD	Pulmonary	Cervix	2.11	1.63	2.59
Other interstitial pulmonary diseases with fibrosis	Pulmonary	Cervix	0.06	0.00	0.14
End stage renal disease	Renal	Cervix	0.84	0.53	1.14
Glomerulonephritis	Renal	Cervix	0.20	0.05	0.35
Obstructive and reflux uropathy	Renal	Cervix	2.48	1.97	3.00
Urolithiasis	Renal	Cervix	2.51	1.99	3.03
Abdominal aortic aneurysm	Cardiovascular	Colon and rectum	1.60	1.48	1.72
Atrial fibrillation	Cardiovascular	Colon and rectum	5.80	5.59	6.02
Atrioventricular block, complete	Cardiovascular	Colon and rectum	1.00	0.91	1.09
Atrioventricular block, first degree	Cardiovascular	Colon and rectum	1.00	0.91	1.09
Atrioventricular block, second degree	Cardiovascular	Colon and rectum	1.00	0.91	1.09
Bifascicular block	Cardiovascular	Colon and rectum	0.62	0.55	0.69
Coronary heart disease not otherwise specified	Cardiovascular	Colon and rectum	8.30	8.05	8.56
Dilated cardiomyopathy	Cardiovascular	Colon and rectum	0.20	0.16	0.24
Heart failure	Cardiovascular	Colon and rectum	4.13	3.94	4.31
Hypertension	Cardiovascular	Colon and rectum	13.51	13.19	13.83
Hypertrophic cardiomyopathy	Cardiovascular	Colon and rectum	0.20	0.16	0.24
Ischaemic stroke	Cardiovascular	Colon and rectum	1.32	1.21	1.42
Left bundle branch block	Cardiovascular	Colon and rectum	1.07	0.98	1.17
Multiple valve disease	Cardiovascular	Colon and rectum	0.63	0.56	0.70
Myocardial infarction	Cardiovascular	Colon and rectum	5.41	5.20	5.62
Nonrheumatic aortic valve disorders	Cardiovascular	Colon and rectum	0.90	0.82	0.99
Nonrheumatic mitral valve disorders	Cardiovascular	Colon and rectum	0.64	0.56	0.71
Other cardiomyopathy	Cardiovascular	Colon and rectum	5.90	5.69	6.12
Pericardial effusion (noninflammatory)	Cardiovascular	Colon and rectum	0.12	0.09	0.15
Peripheral arterial disease	Cardiovascular	Colon and rectum	1.79	1.66	1.91
Primary pulmonary hypertension	Cardiovascular	Colon and rectum	0.18	0.14	0.22
Raynaud's syndrome	Cardiovascular	Colon and rectum	1.19	1.09	1.29
Right bundle branch block	Cardiovascular	Colon and rectum	0.61	0.54	0.68
Secondary pulmonary hypertension	Cardiovascular	Colon and rectum	0.17	0.13	0.21
Sick sinus syndrome	Cardiovascular	Colon and rectum	0.50	0.43	0.56
Stable angina	Cardiovascular	Colon and rectum	4.31	4.12	4.50
Stroke (not otherwise specified)	Cardiovascular	Colon and rectum	1.89	1.77	2.02
Supraventricular tachycardia	Cardiovascular	Colon and rectum	0.60	0.53	0.68
Transient ischaemic attack	Cardiovascular	Colon and rectum	2.40	2.26	2.54
Trifascicular block	Cardiovascular	Colon and rectum	0.53	0.46	0.59
Unstable angina	Cardiovascular	Colon and rectum	4.39	4.20	4.58
Venous thromboembolic disease	Cardiovascular	Colon and rectum	2.49	2.35	2.63
Ventricular tachycardia	Cardiovascular	Colon and rectum	0.62	0.55	0.69
Diabetes	Endocrine	Colon and rectum	6.45	6.22	6.68
Diabetic neurological complications	Endocrine	Colon and rectum	5.93	5.71	6.15
Diabetic ophthalmic complications	Endocrine	Colon and rectum	6.73	6.50	6.97
Hyperparathyroidism	Endocrine	Colon and rectum	0.15	0.11	0.18
Hypo or hyperthyroidism	Endocrine	Colon and rectum	2.23	2.09	2.37
Alcoholic liver disease	Gastrointestinal	Colon and rectum	0.27	0.22	0.32
Angiodysplasia of colon	Gastrointestinal	Colon and rectum	0.32	0.26	0.37
Barrett's oesophagus	Gastrointestinal	Colon and rectum	1.99	1.86	2.12
Cholangitis	Gastrointestinal	Colon and rectum	2.18	2.04	2.31
Cholecystitis	Gastrointestinal	Colon and rectum	2.32	2.18	2.46
Cholelithiasis	Gastrointestinal	Colon and rectum	2.48	2.34	2.63
Coeliac disease	Gastrointestinal	Colon and rectum	0.56	0.49	0.63
Crohn's disease	Gastrointestinal	Colon and rectum	0.31	0.26	0.36
Diverticular disease of intestine	Gastrointestinal	Colon and rectum	9.55	9.27	9.82
Fatty liver	Gastrointestinal	Colon and rectum	1.34	1.23	1.45
Gastritis and duodenitis	Gastrointestinal	Colon and rectum	5.83	5.61	6.05
Gastrooesophageal reflux disease	Gastrointestinal	Colon and rectum	3.75	3.57	3.93
Hepatic failure	Gastrointestinal	Colon and rectum	1.22	1.12	1.33
Liver fibrosis sclerosis and cirrhosis	Gastrointestinal	Colon and rectum	0.29	0.24	0.34
Oesophageal varices	Gastrointestinal	Colon and rectum	0.08	0.05	0.10
Oesophagitis and oesophageal ulcer	Gastrointestinal	Colon and rectum	5.93	5.71	6.15
Portal hypertension	Gastrointestinal	Colon and rectum	0.99	0.90	1.08

Ulcerative colitis	Gastrointestinal	Colon and rectum	0.36	0.31	0.42
Agranulocytosis	Haematological	Colon and rectum	0.15	0.11	0.19
Aplastic anaemias	Haematological	Colon and rectum	0.05	0.03	0.08
Other anaemias	Haematological	Colon and rectum	12.31	12.01	12.62
Other haemolytic anaemias	Haematological	Colon and rectum	0.04	0.02	0.06
Primary or idiopathic thrombocytopaenia	Haematological	Colon and rectum	0.24	0.20	0.29
Secondary or other thrombocytopaenia	Haematological	Colon and rectum	0.41	0.35	0.47
Secondary polycythaemia	Haematological	Colon and rectum	0.11	0.08	0.14
Thrombophilia	Haematological	Colon and rectum	0.18	0.14	0.22
Autoimmune liver disease	Immunology and infection	Colon and rectum	0.24	0.19	0.29
Chronic viral hepatitis	Immunology and infection	Colon and rectum	0.38	0.33	0.44
Encephalitis	Immunology and infection	Colon and rectum	0.13	0.10	0.17
HIV	Immunology and infection	Colon and rectum	0.10	0.07	0.13
Immunodeficiencies	Immunology and infection	Colon and rectum	0.03	0.01	0.05
Infection of anal and rectal regions	Immunology and infection	Colon and rectum	0.17	0.13	0.20
Infection of bones and joints	Immunology and infection	Colon and rectum	0.33	0.28	0.39
Infection of liver	Immunology and infection	Colon and rectum	0.15	0.11	0.18
Infection of skin and subcutaneous tissues	Immunology and infection	Colon and rectum	2.15	2.02	2.29
Infections of other or unspecified organs	Immunology and infection	Colon and rectum	5.31	5.10	5.52
Infections of the digestive system	Immunology and infection	Colon and rectum	7.65	7.41	7.90
Infections of the heart	Immunology and infection	Colon and rectum	0.60	0.53	0.68
Lower respiratory tract infections	Immunology and infection	Colon and rectum	6.68	6.45	6.91
Lupus erythematosus (local and systemic)	Immunology and infection	Colon and rectum	0.06	0.04	0.08
Meningitis	Immunology and infection	Colon and rectum	0.63	0.56	0.70
Non-acute cystitis	Immunology and infection	Colon and rectum	0.32	0.27	0.38
Peritonitis	Immunology and infection	Colon and rectum	15.25	14.92	15.59
Systemic sclerosis	Immunology and infection	Colon and rectum	0.02	0.01	0.03
Viral diseases (excluding chronic hepatitis/HIV)	Immunology and infection	Colon and rectum	0.63	0.55	0.70
Ankylosing spondylitis	Musculoskeletal	Colon and rectum	0.05	0.03	0.07
Enteropathic arthropathy	Musculoskeletal	Colon and rectum	0.18	0.14	0.22
Enthesopathies and synovial disorders	Musculoskeletal	Colon and rectum	4.73	4.53	4.93
Osteoarthritis (excluding spine)	Musculoskeletal	Colon and rectum	7.22	6.98	7.47
Osteoporosis	Musculoskeletal	Colon and rectum	2.50	2.36	2.65
Rheumatoid arthritis	Musculoskeletal	Colon and rectum	0.61	0.54	0.69
Scoliosis	Musculoskeletal	Colon and rectum	0.16	0.13	0.20
Spinal stenosis	Musculoskeletal	Colon and rectum	0.51	0.44	0.57
Bell's palsy	Neurological	Colon and rectum	0.17	0.13	0.20
Dementia	Neurological	Colon and rectum	1.08	0.98	1.17
Disorders of autonomic nervous system	Neurological	Colon and rectum	0.24	0.19	0.28
Epilepsy	Neurological	Colon and rectum	0.42	0.36	0.48
Essential tremor	Neurological	Colon and rectum	0.18	0.14	0.22
Intracerebral haemorrhage	Neurological	Colon and rectum	0.62	0.54	0.69
Intracranial hypertension	Neurological	Colon and rectum	0.11	0.08	0.14
Multiple sclerosis	Neurological	Colon and rectum	0.04	0.02	0.06
Peripheral neuropathies	Neurological	Colon and rectum	1.70	1.58	1.82
Subarachnoid haemorrhage	Neurological	Colon and rectum	0.54	0.47	0.61
Asthma	Pulmonary	Colon and rectum	2.84	2.68	2.99
Bronchiectasis	Pulmonary	Colon and rectum	0.37	0.31	0.43
COPD	Pulmonary	Colon and rectum	4.10	3.92	4.28
Other interstitial pulmonary diseases with fibrosis	Pulmonary	Colon and rectum	0.20	0.16	0.24
End stage renal disease	Renal	Colon and rectum	2.19	2.06	2.33
Glomerulonephritis	Renal	Colon and rectum	0.45	0.39	0.51
Obstructive and reflux uropathy	Renal	Colon and rectum	0.67	0.60	0.75
Urolithiasis	Renal	Colon and rectum	1.29	1.18	1.39
Abdominal aortic aneurysm	Cardiovascular	Gallbladder and biliary tract	2.39	1.54	3.23
Atrial fibrillation	Cardiovascular	Gallbladder and biliary tract	5.09	3.88	6.31
Atrioventricular block, complete	Cardiovascular	Gallbladder and biliary tract	1.59	0.90	2.28
Atrioventricular block, first degree	Cardiovascular	Gallbladder and biliary tract	1.59	0.90	2.28
Atrioventricular block, second degree	Cardiovascular	Gallbladder and biliary tract	1.59	0.90	2.28
Bifascicular block	Cardiovascular	Gallbladder and biliary tract	0.64	0.20	1.08
Coronary heart disease not otherwise specified	Cardiovascular	Gallbladder and biliary tract	7.00	5.59	8.41
Dilated cardiomyopathy	Cardiovascular	Gallbladder and biliary tract	0.08	0.00	0.24

Heart failure	Cardiovascular	Gallbladder and biliary tract	3.58	2.55	4.61
Hypertension	Cardiovascular	Gallbladder and biliary tract	14.80	12.83	16.76
Hypertrophic cardiomyopathy	Cardiovascular	Gallbladder and biliary tract	0.16	0.00	0.38
Ischaemic stroke	Cardiovascular	Gallbladder and biliary tract	1.51	0.84	2.19
Left bundle branch block	Cardiovascular	Gallbladder and biliary tract	1.51	0.84	2.19
Multiple valve disease	Cardiovascular	Gallbladder and biliary tract	0.40	0.05	0.75
Myocardial infarction	Cardiovascular	Gallbladder and biliary tract	5.73	4.44	7.01
Nonrheumatic aortic valve disorders	Cardiovascular	Gallbladder and biliary tract	0.88	0.36	1.39
Nonrheumatic mitral valve disorders	Cardiovascular	Gallbladder and biliary tract	0.72	0.25	1.18
Other cardiomyopathy	Cardiovascular	Gallbladder and biliary tract	6.28	4.94	7.63
Pericardial effusion (noninflammatory)	Cardiovascular	Gallbladder and biliary tract	0.16	0.00	0.38
Peripheral arterial disease	Cardiovascular	Gallbladder and biliary tract	1.75	1.03	2.48
Primary pulmonary hypertension	Cardiovascular	Gallbladder and biliary tract	0.48	0.10	0.86
Raynaud's syndrome	Cardiovascular	Gallbladder and biliary tract	0.95	0.42	1.49
Right bundle branch block	Cardiovascular	Gallbladder and biliary tract	0.64	0.20	1.08
Secondary pulmonary hypertension	Cardiovascular	Gallbladder and biliary tract	0.48	0.10	0.86
Sick sinus syndrome	Cardiovascular	Gallbladder and biliary tract	0.80	0.30	1.29
Stable angina	Cardiovascular	Gallbladder and biliary tract	3.74	2.69	4.79
Stroke (not otherwise specified)	Cardiovascular	Gallbladder and biliary tract	1.99	1.22	2.76
Supraventricular tachycardia	Cardiovascular	Gallbladder and biliary tract	0.72	0.25	1.18
Transient ischaemic attack	Cardiovascular	Gallbladder and biliary tract	2.47	1.61	3.32
Trifascicular block	Cardiovascular	Gallbladder and biliary tract	0.48	0.10	0.86
Unstable angina	Cardiovascular	Gallbladder and biliary tract	4.85	3.66	6.04
Venous thromboembolic disease	Cardiovascular	Gallbladder and biliary tract	2.63	1.74	3.51
Ventricular tachycardia	Cardiovascular	Gallbladder and biliary tract	0.64	0.20	1.08
Diabetes	Endocrine	Gallbladder and biliary tract	9.86	8.22	11.51
Diabetic neurological complications	Endocrine	Gallbladder and biliary tract	9.79	8.14	11.43
Diabetic ophthalmic complications	Endocrine	Gallbladder and biliary tract	10.42	8.73	12.11
Hyperparathyroidism	Endocrine	Gallbladder and biliary tract	0.16	0.00	0.38
Hypo or hyperthyroidism	Endocrine	Gallbladder and biliary tract	2.63	1.74	3.51
Alcoholic liver disease	Gastrointestinal	Gallbladder and biliary tract	0.88	0.36	1.39
Angiodysplasia of colon	Gastrointestinal	Gallbladder and biliary tract	0.40	0.05	0.75
Barrett's oesophagus	Gastrointestinal	Gallbladder and biliary tract	2.07	1.28	2.86
Cholangitis	Gastrointestinal	Gallbladder and biliary tract	49.96	47.20	52.72
Cholecystitis	Gastrointestinal	Gallbladder and biliary tract	30.71	28.16	33.26
Cholelithiasis	Gastrointestinal	Gallbladder and biliary tract	30.95	28.39	33.50
Coeliac disease	Gastrointestinal	Gallbladder and biliary tract	0.56	0.15	0.97
Crohn's disease	Gastrointestinal	Gallbladder and biliary tract	0.16	0.00	0.38
Diverticular disease of intestine	Gastrointestinal	Gallbladder and biliary tract	6.52	5.16	7.89
Fatty liver	Gastrointestinal	Gallbladder and biliary tract	6.21	4.87	7.54
Gastritis and duodenitis	Gastrointestinal	Gallbladder and biliary tract	8.27	6.75	9.80
Gastrooesophageal reflux disease	Gastrointestinal	Gallbladder and biliary tract	6.92	5.52	8.32
Hepatic failure	Gastrointestinal	Gallbladder and biliary tract	6.44	5.09	7.80
Liver fibrosis sclerosis and cirrhosis	Gastrointestinal	Gallbladder and biliary tract	1.35	0.71	1.99
Oesophageal varices	Gastrointestinal	Gallbladder and biliary tract	0.48	0.10	0.86
Oesophagitis and oesophageal ulcer	Gastrointestinal	Gallbladder and biliary tract	8.35	6.82	9.88
Portal hypertension	Gastrointestinal	Gallbladder and biliary tract	4.61	3.45	5.77
Ulcerative colitis	Gastrointestinal	Gallbladder and biliary tract	0.24	0.00	0.51
Agranulocytosis	Haematological	Gallbladder and biliary tract	0.16	0.00	0.38
Other anaemias	Haematological	Gallbladder and biliary tract	7.16	5.73	8.59
Other haemolytic anaemias	Haematological	Gallbladder and biliary tract	0.08	0.00	0.24
Primary or idiopathic thrombocytopaenia	Haematological	Gallbladder and biliary tract	0.24	0.00	0.51
Secondary or other thrombocytopaenia	Haematological	Gallbladder and biliary tract	0.56	0.15	0.97
Secondary polycythaemia	Haematological	Gallbladder and biliary tract	0.08	0.00	0.24
Thrombophilia	Haematological	Gallbladder and biliary tract	0.48	0.10	0.86
Autoimmune liver disease	Immunology and infection	Gallbladder and biliary tract	1.91	1.15	2.67
Chronic viral hepatitis	Immunology and infection	Gallbladder and biliary tract	4.53	3.38	5.68
Encephalitis	Immunology and infection	Gallbladder and biliary tract	0.24	0.00	0.51
HIV	Immunology and infection	Gallbladder and biliary tract	0.40	0.05	0.75
Immunodeficiencies	Immunology and infection	Gallbladder and biliary tract	0.08	0.00	0.24
Infection of anal and rectal regions	Immunology and infection	Gallbladder and biliary tract	0.08	0.00	0.24

Infection of bones and joints	Immunology and infection	Gallbladder and biliary tract	0.24	0.00	0.51
Infection of liver	Immunology and infection	Gallbladder and biliary tract	1.35	0.71	1.99
Infection of skin and subcutaneous tissues	Immunology and infection	Gallbladder and biliary tract	2.47	1.61	3.32
Infections of other or unspecified organs	Immunology and infection	Gallbladder and biliary tract	7.00	5.59	8.41
Infections of the digestive system	Immunology and infection	Gallbladder and biliary tract	4.85	3.66	6.04
Infections of the heart	Immunology and infection	Gallbladder and biliary tract	0.88	0.36	1.39
Lower respiratory tract infections	Immunology and infection	Gallbladder and biliary tract	8.59	7.04	10.14
Lupus erythematosus (local and systemic)	Immunology and infection	Gallbladder and biliary tract	0.08	0.00	0.24
Meningitis	Immunology and infection	Gallbladder and biliary tract	1.03	0.47	1.59
Non-acute cystitis	Immunology and infection	Gallbladder and biliary tract	0.08	0.00	0.24
Peritonitis	Immunology and infection	Gallbladder and biliary tract	12.41	10.59	14.23
Viral diseases (excluding chronic hepatitis/HIV)	Immunology and infection	Gallbladder and biliary tract	1.03	0.47	1.59
Enteropathic arthropathy	Musculoskeletal	Gallbladder and biliary tract	0.32	0.01	0.63
Enthesopathies and synovial disorders	Musculoskeletal	Gallbladder and biliary tract	5.09	3.88	6.31
Osteoarthritis (excluding spine)	Musculoskeletal	Gallbladder and biliary tract	7.00	5.59	8.41
Osteoporosis	Musculoskeletal	Gallbladder and biliary tract	3.74	2.69	4.79
Rheumatoid arthritis	Musculoskeletal	Gallbladder and biliary tract	0.95	0.42	1.49
Scoliosis	Musculoskeletal	Gallbladder and biliary tract	0.24	0.00	0.51
Spinal stenosis	Musculoskeletal	Gallbladder and biliary tract	1.11	0.53	1.69
Bell's palsy	Neurological	Gallbladder and biliary tract	0.40	0.05	0.75
Dementia	Neurological	Gallbladder and biliary tract	1.51	0.84	2.19
Disorders of autonomic nervous system	Neurological	Gallbladder and biliary tract	0.32	0.01	0.63
Epilepsy	Neurological	Gallbladder and biliary tract	0.40	0.05	0.75
Essential tremor	Neurological	Gallbladder and biliary tract	0.32	0.01	0.63
Intracerebral haemorrhage	Neurological	Gallbladder and biliary tract	0.72	0.25	1.18
Intracranial hypertension	Neurological	Gallbladder and biliary tract	0.32	0.01	0.63
Peripheral neuropathies	Neurological	Gallbladder and biliary tract	1.75	1.03	2.48
Subarachnoid haemorrhage	Neurological	Gallbladder and biliary tract	0.40	0.05	0.75
Asthma	Pulmonary	Gallbladder and biliary tract	3.26	2.28	4.24
Bronchiectasis	Pulmonary	Gallbladder and biliary tract	0.40	0.05	0.75
COPD	Pulmonary	Gallbladder and biliary tract	4.46	3.31	5.60
Other interstitial pulmonary diseases with fibrosis	Pulmonary	Gallbladder and biliary tract	0.32	0.01	0.63
End stage renal disease	Renal	Gallbladder and biliary tract	3.50	2.48	4.52
Glomerulonephritis	Renal	Gallbladder and biliary tract	0.88	0.36	1.39
Obstructive and reflux uropathy	Renal	Gallbladder and biliary tract	0.72	0.25	1.18
Urolithiasis	Renal	Gallbladder and biliary tract	1.59	0.90	2.28
Abdominal aortic aneurysm	Cardiovascular	Kidney and renal pelvis	2.02	1.70	2.34
Atrial fibrillation	Cardiovascular	Kidney and renal pelvis	4.83	4.34	5.33
Atrioventricular block, complete	Cardiovascular	Kidney and renal pelvis	0.93	0.71	1.15
Atrioventricular block, first degree	Cardiovascular	Kidney and renal pelvis	0.93	0.71	1.15
Atrioventricular block, second degree	Cardiovascular	Kidney and renal pelvis	0.93	0.71	1.15
Bifascicular block	Cardiovascular	Kidney and renal pelvis	0.54	0.37	0.70
Coronary heart disease not otherwise specified	Cardiovascular	Kidney and renal pelvis	8.75	8.10	9.40
Dilated cardiomyopathy	Cardiovascular	Kidney and renal pelvis	0.26	0.14	0.38
Heart failure	Cardiovascular	Kidney and renal pelvis	3.75	3.31	4.19
Hypertension	Cardiovascular	Kidney and renal pelvis	17.99	17.11	18.87
Hypertrophic cardiomyopathy	Cardiovascular	Kidney and renal pelvis	0.25	0.13	0.36
Ischaemic stroke	Cardiovascular	Kidney and renal pelvis	1.02	0.79	1.25
Left bundle branch block	Cardiovascular	Kidney and renal pelvis	0.96	0.74	1.19
Multiple valve disease	Cardiovascular	Kidney and renal pelvis	0.62	0.44	0.80
Myocardial infarction	Cardiovascular	Kidney and renal pelvis	5.67	5.14	6.20
Nonrheumatic aortic valve disorders	Cardiovascular	Kidney and renal pelvis	1.04	0.81	1.28
Nonrheumatic mitral valve disorders	Cardiovascular	Kidney and renal pelvis	0.70	0.51	0.89
Other cardiomyopathy	Cardiovascular	Kidney and renal pelvis	6.76	6.18	7.33
Pericardial effusion (noninflammatory)	Cardiovascular	Kidney and renal pelvis	0.08	0.02	0.15
Peripheral arterial disease	Cardiovascular	Kidney and renal pelvis	1.55	1.27	1.84
Primary pulmonary hypertension	Cardiovascular	Kidney and renal pelvis	0.25	0.13	0.36
Raynaud's syndrome	Cardiovascular	Kidney and renal pelvis	1.06	0.82	1.29
Right bundle branch block	Cardiovascular	Kidney and renal pelvis	0.52	0.36	0.69
Secondary pulmonary hypertension	Cardiovascular	Kidney and renal pelvis	0.19	0.09	0.29
Sick sinus syndrome	Cardiovascular	Kidney and renal pelvis	0.43	0.28	0.58
Stable angina	Cardiovascular	Kidney and renal pelvis	4.45	3.98	4.92

Stroke (not otherwise specified)	Cardiovascular	Kidney and renal pelvis	1.59	1.31	1.88
Supraventricular tachycardia	Cardiovascular	Kidney and renal pelvis	0.74	0.54	0.94
Transient ischaemic attack	Cardiovascular	Kidney and renal pelvis	2.02	1.70	2.34
Trifascicular block	Cardiovascular	Kidney and renal pelvis	0.45	0.30	0.61
Unstable angina	Cardiovascular	Kidney and renal pelvis	4.60	4.12	5.08
Venous thromboembolic disease	Cardiovascular	Kidney and renal pelvis	3.35	2.94	3.76
Ventricular tachycardia	Cardiovascular	Kidney and renal pelvis	0.74	0.54	0.94
Diabetes	Endocrine	Kidney and renal pelvis	7.69	7.08	8.30
Diabetic neurological complications	Endocrine	Kidney and renal pelvis	7.64	7.03	8.25
Diabetic ophthalmic complications	Endocrine	Kidney and renal pelvis	8.20	7.57	8.83
Hyperparathyroidism	Endocrine	Kidney and renal pelvis	0.36	0.22	0.49
Hypo or hyperthyroidism	Endocrine	Kidney and renal pelvis	2.36	2.01	2.71
Alcoholic liver disease	Gastrointestinal	Kidney and renal pelvis	0.33	0.20	0.46
Angiodysplasia of colon	Gastrointestinal	Kidney and renal pelvis	0.21	0.10	0.31
Barrett's oesophagus	Gastrointestinal	Kidney and renal pelvis	1.43	1.16	1.70
Cholangitis	Gastrointestinal	Kidney and renal pelvis	2.62	2.26	2.99
Cholecystitis	Gastrointestinal	Kidney and renal pelvis	2.87	2.49	3.25
Cholelithiasis	Gastrointestinal	Kidney and renal pelvis	3.10	2.71	3.50
Coeliac disease	Gastrointestinal	Kidney and renal pelvis	0.38	0.24	0.53
Crohn's disease	Gastrointestinal	Kidney and renal pelvis	0.16	0.07	0.26
Diverticular disease of intestine	Gastrointestinal	Kidney and renal pelvis	5.03	4.52	5.53
Fatty liver	Gastrointestinal	Kidney and renal pelvis	1.69	1.39	1.99
Gastritis and duodenitis	Gastrointestinal	Kidney and renal pelvis	4.66	4.17	5.14
Gastrooesophageal reflux disease	Gastrointestinal	Kidney and renal pelvis	4.27	3.81	4.74
Hepatic failure	Gastrointestinal	Kidney and renal pelvis	1.47	1.19	1.75
Liver fibrosis sclerosis and cirrhosis	Gastrointestinal	Kidney and renal pelvis	0.40	0.25	0.54
Oesophageal varices	Gastrointestinal	Kidney and renal pelvis	0.12	0.04	0.20
Oesophagitis and oesophageal ulcer	Gastrointestinal	Kidney and renal pelvis	5.80	5.26	6.33
Portal hypertension	Gastrointestinal	Kidney and renal pelvis	1.19	0.95	1.44
Ulcerative colitis	Gastrointestinal	Kidney and renal pelvis	0.21	0.10	0.31
Agranulocytosis	Haematological	Kidney and renal pelvis	0.10	0.02	0.17
Aplastic anaemias	Haematological	Kidney and renal pelvis	0.05	0.00	0.11
Other anaemias	Haematological	Kidney and renal pelvis	7.81	7.20	8.43
Other haemolytic anaemias	Haematological	Kidney and renal pelvis	0.01	0.00	0.04
Primary or idiopathic thrombocytopaenia	Haematological	Kidney and renal pelvis	0.27	0.15	0.39
Secondary or other thrombocytopaenia	Haematological	Kidney and renal pelvis	0.59	0.41	0.77
Secondary polycythaemia	Haematological	Kidney and renal pelvis	0.34	0.21	0.48
Thrombophilia	Haematological	Kidney and renal pelvis	0.19	0.09	0.29
Autoimmune liver disease	Immunology and infection	Kidney and renal pelvis	0.25	0.13	0.36
Chronic viral hepatitis	Immunology and infection	Kidney and renal pelvis	0.52	0.36	0.69
Encephalitis	Immunology and infection	Kidney and renal pelvis	0.16	0.07	0.26
HIV	Immunology and infection	Kidney and renal pelvis	0.12	0.04	0.20
Infection of anal and rectal regions	Immunology and infection	Kidney and renal pelvis	0.15	0.06	0.24
Infection of bones and joints	Immunology and infection	Kidney and renal pelvis	1.18	0.93	1.43
Infection of liver	Immunology and infection	Kidney and renal pelvis	0.27	0.15	0.39
Infection of skin and subcutaneous tissues	Immunology and infection	Kidney and renal pelvis	2.17	1.84	2.50
Infections of other or unspecified organs	Immunology and infection	Kidney and renal pelvis	5.05	4.55	5.56
Infections of the digestive system	Immunology and infection	Kidney and renal pelvis	2.87	2.49	3.25
Infections of the heart	Immunology and infection	Kidney and renal pelvis	0.67	0.49	0.86
Lower respiratory tract infections	Immunology and infection	Kidney and renal pelvis	7.03	6.44	7.62
Lupus erythematosus (local and systemic)	Immunology and infection	Kidney and renal pelvis	0.04	0.00	0.09
Meningitis	Immunology and infection	Kidney and renal pelvis	0.73	0.53	0.92
Non-acute cystitis	Immunology and infection	Kidney and renal pelvis	1.09	0.85	1.32
Peritonitis	Immunology and infection	Kidney and renal pelvis	7.02	6.43	7.61
Viral diseases (excluding chronic hepatitis/HIV)	Immunology and infection	Kidney and renal pelvis	0.81	0.60	1.02
Ankylosing spondylitis	Musculoskeletal	Kidney and renal pelvis	0.16	0.07	0.26
Enteropathic arthropathy	Musculoskeletal	Kidney and renal pelvis	0.21	0.10	0.31
Enthesopathies and synovial disorders	Musculoskeletal	Kidney and renal pelvis	6.07	5.52	6.62
Osteoarthritis (excluding spine)	Musculoskeletal	Kidney and renal pelvis	7.13	6.54	7.72
Osteoporosis	Musculoskeletal	Kidney and renal pelvis	2.16	1.82	2.49
Rheumatoid arthritis	Musculoskeletal	Kidney and renal pelvis	0.77	0.57	0.97

Scoliosis	Musculoskeletal	Kidney and renal pelvis	0.34	0.21	0.48
Spinal stenosis	Musculoskeletal	Kidney and renal pelvis	0.82	0.62	1.03
Bell's palsy	Neurological	Kidney and renal pelvis	0.25	0.13	0.36
Dementia	Neurological	Kidney and renal pelvis	0.40	0.25	0.54
Disorders of autonomic nervous system	Neurological	Kidney and renal pelvis	0.55	0.38	0.72
Epilepsy	Neurological	Kidney and renal pelvis	0.51	0.34	0.67
Essential tremor	Neurological	Kidney and renal pelvis	0.22	0.11	0.33
Intracerebral haemorrhage	Neurological	Kidney and renal pelvis	0.59	0.41	0.77
Intracranial hypertension	Neurological	Kidney and renal pelvis	0.15	0.06	0.24
Multiple sclerosis	Neurological	Kidney and renal pelvis	0.01	0.00	0.04
Peripheral neuropathies	Neurological	Kidney and renal pelvis	1.85	1.54	2.16
Subarachnoid haemorrhage	Neurological	Kidney and renal pelvis	0.54	0.37	0.70
Asthma	Pulmonary	Kidney and renal pelvis	3.53	3.11	3.95
Bronchiectasis	Pulmonary	Kidney and renal pelvis	0.41	0.26	0.56
COPD	Pulmonary	Kidney and renal pelvis	3.90	3.46	4.35
Other interstitial pulmonary diseases with fibrosis	Pulmonary	Kidney and renal pelvis	0.15	0.06	0.24
End stage renal disease	Renal	Kidney and renal pelvis	3.50	3.08	3.92
Glomerulonephritis	Renal	Kidney and renal pelvis	1.77	1.47	2.07
Obstructive and reflux uropathy	Renal	Kidney and renal pelvis	2.98	2.59	3.37
Urolithiasis	Renal	Kidney and renal pelvis	5.78	5.25	6.32
Abdominal aortic aneurysm	Cardiovascular	Liver and intrahepatic bile duct	1.97	1.32	2.61
Atrial fibrillation	Cardiovascular	Liver and intrahepatic bile duct	6.01	4.91	7.12
Atrioventricular block, complete	Cardiovascular	Liver and intrahepatic bile duct	1.12	0.63	1.61
Atrioventricular block, first degree	Cardiovascular	Liver and intrahepatic bile duct	1.12	0.63	1.61
Atrioventricular block, second degree	Cardiovascular	Liver and intrahepatic bile duct	1.12	0.63	1.61
Bifascicular block	Cardiovascular	Liver and intrahepatic bile duct	0.67	0.29	1.05
Coronary heart disease not otherwise specified	Cardiovascular	Liver and intrahepatic bile duct	7.08	5.89	8.27
Dilated cardiomyopathy	Cardiovascular	Liver and intrahepatic bile duct	0.28	0.04	0.53
Heart failure	Cardiovascular	Liver and intrahepatic bile duct	4.61	3.63	5.58
Hypertension	Cardiovascular	Liver and intrahepatic bile duct	15.23	13.56	16.90
Hypertrophic cardiomyopathy	Cardiovascular	Liver and intrahepatic bile duct	0.28	0.04	0.53
Ischaemic stroke	Cardiovascular	Liver and intrahepatic bile duct	1.41	0.86	1.95
Left bundle branch block	Cardiovascular	Liver and intrahepatic bile duct	1.18	0.68	1.68
Multiple valve disease	Cardiovascular	Liver and intrahepatic bile duct	0.56	0.21	0.91
Myocardial infarction	Cardiovascular	Liver and intrahepatic bile duct	6.91	5.74	8.09
Nonrheumatic aortic valve disorders	Cardiovascular	Liver and intrahepatic bile duct	1.01	0.55	1.48
Nonrheumatic mitral valve disorders	Cardiovascular	Liver and intrahepatic bile duct	1.01	0.55	1.48
Other cardiomyopathy	Cardiovascular	Liver and intrahepatic bile duct	6.80	5.63	7.97
Pericardial effusion (noninflammatory)	Cardiovascular	Liver and intrahepatic bile duct	0.22	0.00	0.44
Peripheral arterial disease	Cardiovascular	Liver and intrahepatic bile duct	2.19	1.51	2.87
Primary pulmonary hypertension	Cardiovascular	Liver and intrahepatic bile duct	0.39	0.10	0.68
Raynaud's syndrome	Cardiovascular	Liver and intrahepatic bile duct	1.52	0.95	2.09
Right bundle branch block	Cardiovascular	Liver and intrahepatic bile duct	0.67	0.29	1.05
Secondary pulmonary hypertension	Cardiovascular	Liver and intrahepatic bile duct	0.39	0.10	0.68
Sick sinus syndrome	Cardiovascular	Liver and intrahepatic bile duct	0.56	0.21	0.91
Stable angina	Cardiovascular	Liver and intrahepatic bile duct	3.77	2.88	4.65
Stroke (not otherwise specified)	Cardiovascular	Liver and intrahepatic bile duct	1.91	1.27	2.55
Supraventricular tachycardia	Cardiovascular	Liver and intrahepatic bile duct	0.73	0.33	1.13
Transient ischaemic attack	Cardiovascular	Liver and intrahepatic bile duct	2.36	1.66	3.07
Trifascicular block	Cardiovascular	Liver and intrahepatic bile duct	0.39	0.10	0.68
Unstable angina	Cardiovascular	Liver and intrahepatic bile duct	4.67	3.69	5.65
Venous thromboembolic disease	Cardiovascular	Liver and intrahepatic bile duct	4.72	3.74	5.71
Ventricular tachycardia	Cardiovascular	Liver and intrahepatic bile duct	0.67	0.29	1.05
Diabetes	Endocrine	Liver and intrahepatic bile duct	10.85	9.40	12.29
Diabetic neurological complications	Endocrine	Liver and intrahepatic bile duct	14.84	13.19	16.49
Diabetic ophthalmic complications	Endocrine	Liver and intrahepatic bile duct	15.18	13.51	16.84
Hyperparathyroidism	Endocrine	Liver and intrahepatic bile duct	0.45	0.14	0.76
Hypo or hyperthyroidism	Endocrine	Liver and intrahepatic bile duct	3.15	2.34	3.96
Alcoholic liver disease	Gastrointestinal	Liver and intrahepatic bile duct	8.88	7.56	10.20
Angiodysplasia of colon	Gastrointestinal	Liver and intrahepatic bile duct	0.56	0.21	0.91
Barrett's oesophagus	Gastrointestinal	Liver and intrahepatic bile duct	2.75	1.99	3.51
Cholangitis	Gastrointestinal	Liver and intrahepatic bile duct	19.28	17.45	21.11

Cholecystitis	Gastrointestinal	Liver and intrahepatic bile duct	10.01	8.61	11.40
Cholelithiasis	Gastrointestinal	Liver and intrahepatic bile duct	10.91	9.46	12.35
Coeliac disease	Gastrointestinal	Liver and intrahepatic bile duct	0.67	0.29	1.05
Crohn's disease	Gastrointestinal	Liver and intrahepatic bile duct	0.39	0.10	0.68
Diverticular disease of intestine	Gastrointestinal	Liver and intrahepatic bile duct	7.42	6.20	8.64
Fatty liver	Gastrointestinal	Liver and intrahepatic bile duct	19.51	17.66	21.35
Gastritis and duodenitis	Gastrointestinal	Liver and intrahepatic bile duct	12.31	10.78	13.84
Gastrooesophageal reflux disease	Gastrointestinal	Liver and intrahepatic bile duct	5.62	4.55	6.69
Hepatic failure	Gastrointestinal	Liver and intrahepatic bile duct	20.12	18.26	21.99
Liver fibrosis sclerosis and cirrhosis	Gastrointestinal	Liver and intrahepatic bile duct	15.51	13.83	17.20
Oesophageal varices	Gastrointestinal	Liver and intrahepatic bile duct	8.99	7.66	10.32
Oesophagitis and oesophageal ulcer	Gastrointestinal	Liver and intrahepatic bile duct	7.87	6.62	9.12
Portal hypertension	Gastrointestinal	Liver and intrahepatic bile duct	16.64	14.91	18.37
Ulcerative colitis	Gastrointestinal	Liver and intrahepatic bile duct	0.39	0.10	0.68
Agranulocytosis	Haematological	Liver and intrahepatic bile duct	0.45	0.14	0.76
Aplastic anaemias	Haematological	Liver and intrahepatic bile duct	0.79	0.38	1.20
Other anaemias	Haematological	Liver and intrahepatic bile duct	8.77	7.45	10.08
Primary or idiopathic thrombocytopaenia	Haematological	Liver and intrahepatic bile duct	2.59	1.85	3.32
Secondary or other thrombocytopaenia	Haematological	Liver and intrahepatic bile duct	3.43	2.58	4.27
Secondary polycythaemia	Haematological	Liver and intrahepatic bile duct	0.34	0.07	0.61
Thrombophilia	Haematological	Liver and intrahepatic bile duct	0.51	0.18	0.84
Autoimmune liver disease	Immunology and infection	Liver and intrahepatic bile duct	11.92	10.41	13.42
Chronic viral hepatitis	Immunology and infection	Liver and intrahepatic bile duct	8.99	7.66	10.32
Encephalitis	Immunology and infection	Liver and intrahepatic bile duct	0.06	0.00	0.17
HIV	Immunology and infection	Liver and intrahepatic bile duct	0.22	0.00	0.44
Infection of anal and rectal regions	Immunology and infection	Liver and intrahepatic bile duct	0.06	0.00	0.17
Infection of bones and joints	Immunology and infection	Liver and intrahepatic bile duct	1.18	0.68	1.68
Infection of liver	Immunology and infection	Liver and intrahepatic bile duct	7.48	6.25	8.70
Infection of skin and subcutaneous tissues	Immunology and infection	Liver and intrahepatic bile duct	4.38	3.43	5.34
Infections of other or unspecified organs	Immunology and infection	Liver and intrahepatic bile duct	7.81	6.57	9.06
Infections of the digestive system	Immunology and infection	Liver and intrahepatic bile duct	6.07	4.96	7.18
Infections of the heart	Immunology and infection	Liver and intrahepatic bile duct	6.01	4.91	7.12
Lower respiratory tract infections	Immunology and infection	Liver and intrahepatic bile duct	10.46	9.03	11.88
Lupus erythematosus (local and systemic)	Immunology and infection	Liver and intrahepatic bile duct	0.06	0.00	0.17
Meningitis	Immunology and infection	Liver and intrahepatic bile duct	1.18	0.68	1.68
Non-acute cystitis	Immunology and infection	Liver and intrahepatic bile duct	0.39	0.10	0.68
Peritonitis	Immunology and infection	Liver and intrahepatic bile duct	13.88	12.28	15.49
Systemic sclerosis	Immunology and infection	Liver and intrahepatic bile duct	0.06	0.00	0.17
Viral diseases (excluding chronic hepatitis/HIV)	Immunology and infection	Liver and intrahepatic bile duct	2.98	2.19	3.77
Ankylosing spondylitis	Musculoskeletal	Liver and intrahepatic bile duct	0.06	0.00	0.17
Enteropathic arthropathy	Musculoskeletal	Liver and intrahepatic bile duct	0.34	0.07	0.61
Enthesopathies and synovial disorders	Musculoskeletal	Liver and intrahepatic bile duct	5.17	4.14	6.20
Osteoarthritis (excluding spine)	Musculoskeletal	Liver and intrahepatic bile duct	6.86	5.68	8.03
Osteoporosis	Musculoskeletal	Liver and intrahepatic bile duct	3.54	2.68	4.40
Rheumatoid arthritis	Musculoskeletal	Liver and intrahepatic bile duct	0.51	0.18	0.84
Spinal stenosis	Musculoskeletal	Liver and intrahepatic bile duct	1.29	0.77	1.82
Bell's palsy	Neurological	Liver and intrahepatic bile duct	0.17	0.00	0.36
Dementia	Neurological	Liver and intrahepatic bile duct	1.29	0.77	1.82
Disorders of autonomic nervous system	Neurological	Liver and intrahepatic bile duct	0.96	0.50	1.41
Epilepsy	Neurological	Liver and intrahepatic bile duct	0.62	0.25	0.98
Essential tremor	Neurological	Liver and intrahepatic bile duct	0.34	0.07	0.61
Intracerebral haemorrhage	Neurological	Liver and intrahepatic bile duct	0.73	0.33	1.13
Intracranial hypertension	Neurological	Liver and intrahepatic bile duct	1.18	0.68	1.68
Multiple sclerosis	Neurological	Liver and intrahepatic bile duct	0.06	0.00	0.17
Peripheral neuropathies	Neurological	Liver and intrahepatic bile duct	3.26	2.43	4.09
Subarachnoid haemorrhage	Neurological	Liver and intrahepatic bile duct	0.67	0.29	1.05
Asthma	Pulmonary	Liver and intrahepatic bile duct	4.05	3.13	4.96
Bronchiectasis	Pulmonary	Liver and intrahepatic bile duct	0.39	0.10	0.68
COPD	Pulmonary	Liver and intrahepatic bile duct	5.28	4.24	6.32
Other interstitial pulmonary diseases with fibrosis	Pulmonary	Liver and intrahepatic bile duct	0.67	0.29	1.05

End stage renal disease	Renal	Liver and intrahepatic bile duct	4.89	3.89	5.89
Glomerulonephritis	Renal	Liver and intrahepatic bile duct	0.84	0.42	1.27
Obstructive and reflux uropathy	Renal	Liver and intrahepatic bile duct	0.34	0.07	0.61
Urolithiasis	Renal	Liver and intrahepatic bile duct	1.41	0.86	1.95
Abdominal aortic aneurysm	Cardiovascular	Lung and bronchus	2.30	2.13	2.46
Atrial fibrillation	Cardiovascular	Lung and bronchus	6.01	5.74	6.27
Atrioventricular block, complete	Cardiovascular	Lung and bronchus	1.04	0.93	1.15
Atrioventricular block, first degree	Cardiovascular	Lung and bronchus	1.04	0.93	1.15
Atrioventricular block, second degree	Cardiovascular	Lung and bronchus	1.04	0.93	1.15
Bifascicular block	Cardiovascular	Lung and bronchus	0.84	0.74	0.94
Coronary heart disease not otherwise specified	Cardiovascular	Lung and bronchus	9.11	8.79	9.43
Dilated cardiomyopathy	Cardiovascular	Lung and bronchus	0.19	0.14	0.23
Heart failure	Cardiovascular	Lung and bronchus	4.34	4.11	4.56
Hypertension	Cardiovascular	Lung and bronchus	15.28	14.88	15.68
Hypertrophic cardiomyopathy	Cardiovascular	Lung and bronchus	0.18	0.13	0.23
Ischaemic stroke	Cardiovascular	Lung and bronchus	1.44	1.31	1.57
Left bundle branch block	Cardiovascular	Lung and bronchus	1.10	0.98	1.21
Multiple valve disease	Cardiovascular	Lung and bronchus	0.63	0.54	0.72
Myocardial infarction	Cardiovascular	Lung and bronchus	6.73	6.45	7.01
Nonrheumatic aortic valve disorders	Cardiovascular	Lung and bronchus	0.94	0.83	1.04
Nonrheumatic mitral valve disorders	Cardiovascular	Lung and bronchus	0.70	0.61	0.80
Other cardiomyopathy	Cardiovascular	Lung and bronchus	7.62	7.32	7.91
Pericardial effusion (noninflammatory)	Cardiovascular	Lung and bronchus	0.63	0.55	0.72
Peripheral arterial disease	Cardiovascular	Lung and bronchus	3.71	3.50	3.92
Primary pulmonary hypertension	Cardiovascular	Lung and bronchus	0.33	0.27	0.39
Raynaud's syndrome	Cardiovascular	Lung and bronchus	2.72	2.54	2.90
Right bundle branch block	Cardiovascular	Lung and bronchus	0.84	0.74	0.94
Secondary pulmonary hypertension	Cardiovascular	Lung and bronchus	0.31	0.25	0.37
Sick sinus syndrome	Cardiovascular	Lung and bronchus	0.52	0.44	0.60
Stable angina	Cardiovascular	Lung and bronchus	5.10	4.86	5.35
Stroke (not otherwise specified)	Cardiovascular	Lung and bronchus	2.29	2.13	2.46
Supraventricular tachycardia	Cardiovascular	Lung and bronchus	0.80	0.70	0.90
Transient ischaemic attack	Cardiovascular	Lung and bronchus	3.09	2.90	3.28
Trifascicular block	Cardiovascular	Lung and bronchus	0.74	0.65	0.84
Unstable angina	Cardiovascular	Lung and bronchus	5.27	5.03	5.52
Venous thromboembolic disease	Cardiovascular	Lung and bronchus	3.37	3.17	3.57
Ventricular tachycardia	Cardiovascular	Lung and bronchus	0.79	0.69	0.89
Diabetes	Endocrine	Lung and bronchus	6.93	6.65	7.22
Diabetic neurological complications	Endocrine	Lung and bronchus	6.00	5.74	6.26
Diabetic ophthalmic complications	Endocrine	Lung and bronchus	6.82	6.54	7.10
Hyperparathyroidism	Endocrine	Lung and bronchus	0.16	0.12	0.21
Hypo or hyperthyroidism	Endocrine	Lung and bronchus	2.37	2.20	2.54
Alcoholic liver disease	Gastrointestinal	Lung and bronchus	0.46	0.38	0.53
Angiodysplasia of colon	Gastrointestinal	Lung and bronchus	0.26	0.21	0.32
Barrett's oesophagus	Gastrointestinal	Lung and bronchus	1.62	1.48	1.76
Cholangitis	Gastrointestinal	Lung and bronchus	2.03	1.87	2.18
Cholecystitis	Gastrointestinal	Lung and bronchus	2.09	1.93	2.24
Cholelithiasis	Gastrointestinal	Lung and bronchus	2.21	2.05	2.37
Coeliac disease	Gastrointestinal	Lung and bronchus	0.32	0.26	0.38
Crohn's disease	Gastrointestinal	Lung and bronchus	0.15	0.11	0.20
Diverticular disease of intestine	Gastrointestinal	Lung and bronchus	4.45	4.22	4.68
Fatty liver	Gastrointestinal	Lung and bronchus	1.47	1.33	1.60
Gastritis and duodenitis	Gastrointestinal	Lung and bronchus	5.00	4.76	5.24
Gastrooesophageal reflux disease	Gastrointestinal	Lung and bronchus	3.91	3.70	4.13
Hepatic failure	Gastrointestinal	Lung and bronchus	1.31	1.18	1.44
Liver fibrosis sclerosis and cirrhosis	Gastrointestinal	Lung and bronchus	0.41	0.34	0.48
Oesophageal varices	Gastrointestinal	Lung and bronchus	0.09	0.06	0.13
Oesophagitis and oesophageal ulcer	Gastrointestinal	Lung and bronchus	5.26	5.01	5.51
Portal hypertension	Gastrointestinal	Lung and bronchus	1.00	0.89	1.11
Ulcerative colitis	Gastrointestinal	Lung and bronchus	0.22	0.17	0.27
Agranulocytosis	Haematological	Lung and bronchus	0.26	0.20	0.32
Aplastic anaemias	Haematological	Lung and bronchus	0.06	0.03	0.09

Other anaemias	Haematological	Lung and bronchus	4.75	4.52	4.99
Other haemolytic anaemias	Haematological	Lung and bronchus	0.02	0.00	0.03
Primary or idiopathic thrombocytopaenia	Haematological	Lung and bronchus	0.28	0.22	0.34
Secondary or other thrombocytopaenia	Haematological	Lung and bronchus	0.48	0.40	0.55
Secondary polycythaemia	Haematological	Lung and bronchus	0.30	0.24	0.36
Thrombophilia	Haematological	Lung and bronchus	0.17	0.12	0.22
Autoimmune liver disease	Immunology and infection	Lung and bronchus	0.19	0.14	0.24
Chronic viral hepatitis	Immunology and infection	Lung and bronchus	0.40	0.33	0.47
Encephalitis	Immunology and infection	Lung and bronchus	0.15	0.11	0.20
HIV	Immunology and infection	Lung and bronchus	0.12	0.08	0.16
Immunodeficiencies	Immunology and infection	Lung and bronchus	0.04	0.01	0.06
Infection of anal and rectal regions	Immunology and infection	Lung and bronchus	0.13	0.09	0.18
Infection of bones and joints	Immunology and infection	Lung and bronchus	1.09	0.98	1.21
Infection of liver	Immunology and infection	Lung and bronchus	0.17	0.13	0.22
Infection of skin and subcutaneous tissues	Immunology and infection	Lung and bronchus	2.40	2.23	2.57
Infections of other or unspecified organs	Immunology and infection	Lung and bronchus	5.19	4.95	5.44
Infections of the digestive system	Immunology and infection	Lung and bronchus	2.96	2.77	3.15
Infections of the heart	Immunology and infection	Lung and bronchus	0.87	0.76	0.97
Lower respiratory tract infections	Immunology and infection	Lung and bronchus	18.75	18.31	19.18
Lupus erythematosus (local and systemic)	Immunology and infection	Lung and bronchus	0.11	0.08	0.15
Meningitis	Immunology and infection	Lung and bronchus	0.92	0.81	1.02
Non-acute cystitis	Immunology and infection	Lung and bronchus	0.38	0.31	0.45
Peritonitis	Immunology and infection	Lung and bronchus	6.50	6.23	6.78
Systemic sclerosis	Immunology and infection	Lung and bronchus	0.06	0.03	0.08
Viral diseases (excluding chronic hepatitis/HIV)	Immunology and infection	Lung and bronchus	1.10	0.98	1.21
Ankylosing spondylitis	Musculoskeletal	Lung and bronchus	0.09	0.06	0.12
Enteropathic arthropathy	Musculoskeletal	Lung and bronchus	0.16	0.12	0.21
Enthesopathies and synovial disorders	Musculoskeletal	Lung and bronchus	5.61	5.36	5.87
Osteoarthritis (excluding spine)	Musculoskeletal	Lung and bronchus	7.69	7.40	7.99
Osteoporosis	Musculoskeletal	Lung and bronchus	3.23	3.03	3.42
Rheumatoid arthritis	Musculoskeletal	Lung and bronchus	1.06	0.94	1.17
Scoliosis	Musculoskeletal	Lung and bronchus	0.33	0.26	0.39
Spinal stenosis	Musculoskeletal	Lung and bronchus	0.95	0.84	1.05
Bell's palsy	Neurological	Lung and bronchus	0.24	0.18	0.29
Dementia	Neurological	Lung and bronchus	0.76	0.66	0.86
Disorders of autonomic nervous system	Neurological	Lung and bronchus	0.74	0.64	0.83
Epilepsy	Neurological	Lung and bronchus	0.68	0.59	0.77
Essential tremor	Neurological	Lung and bronchus	0.25	0.19	0.31
Intracerebral haemorrhage	Neurological	Lung and bronchus	0.64	0.56	0.73
Intracranial hypertension	Neurological	Lung and bronchus	0.44	0.37	0.51
Multiple sclerosis	Neurological	Lung and bronchus	0.05	0.02	0.07
Peripheral neuropathies	Neurological	Lung and bronchus	2.24	2.07	2.40
Subarachnoid haemorrhage	Neurological	Lung and bronchus	0.57	0.48	0.65
Asthma	Pulmonary	Lung and bronchus	5.11	4.86	5.35
Bronchiectasis	Pulmonary	Lung and bronchus	0.85	0.75	0.95
COPD	Pulmonary	Lung and bronchus	15.78	15.37	16.18
Other interstitial pulmonary diseases with fibrosis	Pulmonary	Lung and bronchus	1.30	1.18	1.43
End stage renal disease	Renal	Lung and bronchus	2.70	2.52	2.88
Glomerulonephritis	Renal	Lung and bronchus	0.61	0.53	0.70
Obstructive and reflux uropathy	Renal	Lung and bronchus	0.44	0.37	0.52
Urolithiasis	Renal	Lung and bronchus	1.05	0.93	1.16
Abdominal aortic aneurysm	Cardiovascular	Melanoma	1.10	0.94	1.26
Atrial fibrillation	Cardiovascular	Melanoma	3.02	2.75	3.28
Atrioventricular block, complete	Cardiovascular	Melanoma	0.61	0.49	0.73
Atrioventricular block, first degree	Cardiovascular	Melanoma	0.61	0.49	0.73
Atrioventricular block, second degree	Cardiovascular	Melanoma	0.61	0.49	0.73
Bifascicular block	Cardiovascular	Melanoma	0.38	0.28	0.47
Coronary heart disease not otherwise specified	Cardiovascular	Melanoma	5.86	5.50	6.22
Dilated cardiomyopathy	Cardiovascular	Melanoma	0.11	0.06	0.16
Heart failure	Cardiovascular	Melanoma	2.39	2.16	2.63
Hypertension	Cardiovascular	Melanoma	8.87	8.43	9.31

Hypertrophic cardiomyopathy	Cardiovascular	Melanoma	0.10	0.05	0.15
Ischaemic stroke	Cardiovascular	Melanoma	0.71	0.58	0.84
Left bundle branch block	Cardiovascular	Melanoma	0.69	0.56	0.81
Multiple valve disease	Cardiovascular	Melanoma	0.37	0.28	0.47
Myocardial infarction	Cardiovascular	Melanoma	2.90	2.65	3.16
Nonrheumatic aortic valve disorders	Cardiovascular	Melanoma	0.53	0.41	0.64
Nonrheumatic mitral valve disorders	Cardiovascular	Melanoma	0.48	0.37	0.58
Other cardiomyopathy	Cardiovascular	Melanoma	3.10	2.83	3.36
Pericardial effusion (noninflammatory)	Cardiovascular	Melanoma	0.11	0.06	0.16
Peripheral arterial disease	Cardiovascular	Melanoma	0.92	0.78	1.07
Primary pulmonary hypertension	Cardiovascular	Melanoma	0.11	0.06	0.16
Raynaud's syndrome	Cardiovascular	Melanoma	0.59	0.48	0.71
Right bundle branch block	Cardiovascular	Melanoma	0.38	0.28	0.47
Secondary pulmonary hypertension	Cardiovascular	Melanoma	0.09	0.05	0.14
Sick sinus syndrome	Cardiovascular	Melanoma	0.25	0.18	0.33
Stable angina	Cardiovascular	Melanoma	2.17	1.94	2.39
Stroke (not otherwise specified)	Cardiovascular	Melanoma	1.18	1.02	1.35
Supraventricular tachycardia	Cardiovascular	Melanoma	0.33	0.25	0.42
Transient ischaemic attack	Cardiovascular	Melanoma	1.89	1.68	2.10
Trifascicular block	Cardiovascular	Melanoma	0.29	0.21	0.37
Unstable angina	Cardiovascular	Melanoma	2.12	1.90	2.35
Venous thromboembolic disease	Cardiovascular	Melanoma	1.59	1.40	1.78
Ventricular tachycardia	Cardiovascular	Melanoma	0.33	0.24	0.42
Diabetes	Endocrine	Melanoma	4.30	3.99	4.62
Diabetic neurological complications	Endocrine	Melanoma	2.42	2.18	2.65
Diabetic ophthalmic complications	Endocrine	Melanoma	3.11	2.84	3.38
Hyperparathyroidism	Endocrine	Melanoma	0.17	0.11	0.24
Hypo or hyperthyroidism	Endocrine	Melanoma	1.51	1.32	1.69
Alcoholic liver disease	Gastrointestinal	Melanoma	0.19	0.12	0.25
Angiodysplasia of colon	Gastrointestinal	Melanoma	0.09	0.04	0.13
Barrett's oesophagus	Gastrointestinal	Melanoma	0.73	0.60	0.86
Cholangitis	Gastrointestinal	Melanoma	1.24	1.07	1.42
Cholecystitis	Gastrointestinal	Melanoma	1.33	1.15	1.51
Cholelithiasis	Gastrointestinal	Melanoma	1.55	1.36	1.74
Coeliac disease	Gastrointestinal	Melanoma	0.27	0.19	0.35
Crohn's disease	Gastrointestinal	Melanoma	0.12	0.07	0.18
Diverticular disease of intestine	Gastrointestinal	Melanoma	2.49	2.25	2.73
Fatty liver	Gastrointestinal	Melanoma	0.51	0.40	0.62
Gastritis and duodenitis	Gastrointestinal	Melanoma	2.09	1.87	2.31
Gastrooesophageal reflux disease	Gastrointestinal	Melanoma	2.93	2.67	3.19
Hepatic failure	Gastrointestinal	Melanoma	0.36	0.27	0.45
Liver fibrosis sclerosis and cirrhosis	Gastrointestinal	Melanoma	0.09	0.04	0.13
Oesophageal varices	Gastrointestinal	Melanoma	0.04	0.01	0.08
Oesophagitis and oesophageal ulcer	Gastrointestinal	Melanoma	3.32	3.04	3.60
Portal hypertension	Gastrointestinal	Melanoma	0.32	0.23	0.40
Ulcerative colitis	Gastrointestinal	Melanoma	0.15	0.09	0.21
Agranulocytosis	Haematological	Melanoma	0.10	0.05	0.15
Aplastic anaemias	Haematological	Melanoma	0.03	0.00	0.06
Other anaemias	Haematological	Melanoma	2.04	1.82	2.26
Other haemolytic anaemias	Haematological	Melanoma	0.04	0.01	0.08
Primary or idiopathic thrombocytopaenia	Haematological	Melanoma	0.17	0.11	0.24
Secondary or other thrombocytopaenia	Haematological	Melanoma	0.29	0.21	0.37
Secondary polycythaemia	Haematological	Melanoma	0.07	0.03	0.12
Thrombophilia	Haematological	Melanoma	0.12	0.07	0.18
Autoimmune liver disease	Immunology and infection	Melanoma	0.07	0.03	0.11
Chronic viral hepatitis	Immunology and infection	Melanoma	0.40	0.30	0.49
Encephalitis	Immunology and infection	Melanoma	0.13	0.07	0.19
HIV	Immunology and infection	Melanoma	0.30	0.22	0.39
Infection of anal and rectal regions	Immunology and infection	Melanoma	0.09	0.05	0.14
Infection of bones and joints	Immunology and infection	Melanoma	0.19	0.12	0.26
Infection of liver	Immunology and infection	Melanoma	0.06	0.02	0.10
Infection of skin and subcutaneous tissues	Immunology and infection	Melanoma	1.42	1.24	1.61

Infections of other or unspecified organs	Immunology and infection	Melanoma	2.38	2.15	2.62
Infections of the digestive system	Immunology and infection	Melanoma	1.31	1.14	1.49
Infections of the heart	Immunology and infection	Melanoma	0.24	0.17	0.32
Lower respiratory tract infections	Immunology and infection	Melanoma	2.74	2.49	3.00
Lupus erythematosus (local and systemic)	Immunology and infection	Melanoma	0.03	0.00	0.06
Meningitis	Immunology and infection	Melanoma	0.35	0.26	0.44
Non-acute cystitis	Immunology and infection	Melanoma	0.28	0.20	0.36
Peritonitis	Immunology and infection	Melanoma	3.20	2.92	3.47
Systemic sclerosis	Immunology and infection	Melanoma	0.03	0.00	0.06
Viral diseases (excluding chronic hepatitis/HIV)	Immunology and infection	Melanoma	0.61	0.49	0.73
Ankylosing spondylitis	Musculoskeletal	Melanoma	0.02	0.00	0.05
Enteropathic arthropathy	Musculoskeletal	Melanoma	0.15	0.09	0.21
Enthesopathies and synovial disorders	Musculoskeletal	Melanoma	5.28	4.94	5.63
Osteoarthritis (excluding spine)	Musculoskeletal	Melanoma	5.98	5.62	6.35
Osteoporosis	Musculoskeletal	Melanoma	1.54	1.35	1.73
Rheumatoid arthritis	Musculoskeletal	Melanoma	0.43	0.33	0.53
Scoliosis	Musculoskeletal	Melanoma	0.22	0.15	0.29
Spinal stenosis	Musculoskeletal	Melanoma	0.48	0.37	0.58
Bell's palsy	Neurological	Melanoma	0.25	0.17	0.32
Dementia	Neurological	Melanoma	0.84	0.70	0.98
Disorders of autonomic nervous system	Neurological	Melanoma	0.20	0.13	0.27
Epilepsy	Neurological	Melanoma	0.40	0.30	0.49
Essential tremor	Neurological	Melanoma	0.17	0.11	0.24
Intracerebral haemorrhage	Neurological	Melanoma	0.38	0.29	0.48
Intracranial hypertension	Neurological	Melanoma	0.07	0.03	0.11
Multiple sclerosis	Neurological	Melanoma	0.04	0.01	0.08
Peripheral neuropathies	Neurological	Melanoma	1.67	1.47	1.86
Subarachnoid haemorrhage	Neurological	Melanoma	0.25	0.18	0.33
Asthma	Pulmonary	Melanoma	1.85	1.64	2.06
Bronchiectasis	Pulmonary	Melanoma	0.22	0.15	0.29
COPD	Pulmonary	Melanoma	1.74	1.54	1.94
Other interstitial pulmonary diseases with fibrosis	Pulmonary	Melanoma	0.14	0.08	0.20
End stage renal disease	Renal	Melanoma	1.34	1.17	1.52
Glomerulonephritis	Renal	Melanoma	0.18	0.11	0.24
Obstructive and reflux uropathy	Renal	Melanoma	0.28	0.20	0.36
Urolithiasis	Renal	Melanoma	0.71	0.58	0.84
Abdominal aortic aneurysm	Cardiovascular	Oesophagus	1.98	1.68	2.28
Atrial fibrillation	Cardiovascular	Oesophagus	4.88	4.41	5.34
Atrioventricular block, complete	Cardiovascular	Oesophagus	1.11	0.88	1.34
Atrioventricular block, first degree	Cardiovascular	Oesophagus	1.11	0.88	1.34
Atrioventricular block, second degree	Cardiovascular	Oesophagus	1.11	0.88	1.34
Bifascicular block	Cardiovascular	Oesophagus	0.65	0.47	0.82
Coronary heart disease not otherwise specified	Cardiovascular	Oesophagus	8.20	7.61	8.79
Dilated cardiomyopathy	Cardiovascular	Oesophagus	0.16	0.07	0.25
Heart failure	Cardiovascular	Oesophagus	3.73	3.32	4.14
Hypertension	Cardiovascular	Oesophagus	13.31	12.57	14.04
Hypertrophic cardiomyopathy	Cardiovascular	Oesophagus	0.18	0.09	0.28
Ischaemic stroke	Cardiovascular	Oesophagus	1.38	1.13	1.63
Left bundle branch block	Cardiovascular	Oesophagus	1.20	0.96	1.43
Multiple valve disease	Cardiovascular	Oesophagus	0.48	0.33	0.63
Myocardial infarction	Cardiovascular	Oesophagus	5.69	5.19	6.20
Nonrheumatic aortic valve disorders	Cardiovascular	Oesophagus	0.98	0.76	1.19
Nonrheumatic mitral valve disorders	Cardiovascular	Oesophagus	0.59	0.42	0.75
Other cardiomyopathy	Cardiovascular	Oesophagus	6.15	5.63	6.67
Pericardial effusion (noninflammatory)	Cardiovascular	Oesophagus	0.15	0.06	0.23
Peripheral arterial disease	Cardiovascular	Oesophagus	2.07	1.76	2.37
Primary pulmonary hypertension	Cardiovascular	Oesophagus	0.17	0.08	0.26
Raynaud's syndrome	Cardiovascular	Oesophagus	1.63	1.35	1.90
Right bundle branch block	Cardiovascular	Oesophagus	0.62	0.45	0.79
Secondary pulmonary hypertension	Cardiovascular	Oesophagus	0.16	0.07	0.25
Sick sinus syndrome	Cardiovascular	Oesophagus	0.37	0.24	0.50

Stable angina	Cardiovascular	Oesophagus	4.40	3.96	4.84
Stroke (not otherwise specified)	Cardiovascular	Oesophagus	2.04	1.73	2.35
Supraventricular tachycardia	Cardiovascular	Oesophagus	0.65	0.47	0.82
Transient ischaemic attack	Cardiovascular	Oesophagus	3.01	2.64	3.38
Trifascicular block	Cardiovascular	Oesophagus	0.53	0.37	0.68
Unstable angina	Cardiovascular	Oesophagus	4.44	3.99	4.88
Venous thromboembolic disease	Cardiovascular	Oesophagus	2.75	2.40	3.10
Ventricular tachycardia	Cardiovascular	Oesophagus	0.67	0.50	0.85
Diabetes	Endocrine	Oesophagus	6.42	5.88	6.95
Diabetic neurological complications	Endocrine	Oesophagus	4.99	4.51	5.46
Diabetic ophthalmic complications	Endocrine	Oesophagus	5.63	5.13	6.13
Hyperparathyroidism	Endocrine	Oesophagus	0.09	0.02	0.15
Hypo or hyperthyroidism	Endocrine	Oesophagus	1.86	1.56	2.15
Alcoholic liver disease	Gastrointestinal	Oesophagus	0.55	0.39	0.71
Angiodysplasia of colon	Gastrointestinal	Oesophagus	0.15	0.06	0.23
Barrett's oesophagus	Gastrointestinal	Oesophagus	12.92	12.19	13.64
Cholangitis	Gastrointestinal	Oesophagus	1.87	1.58	2.16
Cholecystitis	Gastrointestinal	Oesophagus	1.96	1.66	2.26
Cholelithiasis	Gastrointestinal	Oesophagus	2.36	2.03	2.69
Coeliac disease	Gastrointestinal	Oesophagus	0.42	0.28	0.55
Crohn's disease	Gastrointestinal	Oesophagus	0.10	0.03	0.17
Diverticular disease of intestine	Gastrointestinal	Oesophagus	4.15	3.72	4.59
Fatty liver	Gastrointestinal	Oesophagus	1.27	1.03	1.51
Gastritis and duodenitis	Gastrointestinal	Oesophagus	6.21	5.69	6.73
Gastrooesophageal reflux disease	Gastrointestinal	Oesophagus	8.82	8.21	9.44
Hepatic failure	Gastrointestinal	Oesophagus	1.20	0.96	1.43
Liver fibrosis sclerosis and cirrhosis	Gastrointestinal	Oesophagus	0.46	0.32	0.61
Oesophageal varices	Gastrointestinal	Oesophagus	0.26	0.15	0.37
Oesophagitis and oesophageal ulcer	Gastrointestinal	Oesophagus	18.14	17.30	18.97
Portal hypertension	Gastrointestinal	Oesophagus	0.79	0.60	0.99
Ulcerative colitis	Gastrointestinal	Oesophagus	0.27	0.16	0.38
Agranulocytosis	Haematological	Oesophagus	0.27	0.16	0.38
Aplastic anaemias	Haematological	Oesophagus	0.13	0.06	0.21
Other anaemias	Haematological	Oesophagus	5.63	5.13	6.13
Other haemolytic anaemias	Haematological	Oesophagus	0.04	0.00	0.08
Primary or idiopathic thrombocytopaenia	Haematological	Oesophagus	0.12	0.05	0.20
Secondary or other thrombocytopaenia	Haematological	Oesophagus	0.29	0.18	0.41
Secondary polycythaemia	Haematological	Oesophagus	0.07	0.01	0.13
Thrombophilia	Haematological	Oesophagus	0.10	0.03	0.17
Autoimmune liver disease	Immunology and infection	Oesophagus	0.15	0.06	0.23
Chronic viral hepatitis	Immunology and infection	Oesophagus	0.33	0.21	0.45
Encephalitis	Immunology and infection	Oesophagus	0.15	0.06	0.23
HIV	Immunology and infection	Oesophagus	0.10	0.03	0.17
Infection of anal and rectal regions	Immunology and infection	Oesophagus	0.06	0.01	0.11
Infection of bones and joints	Immunology and infection	Oesophagus	0.50	0.35	0.65
Infection of liver	Immunology and infection	Oesophagus	0.18	0.09	0.28
Infection of skin and subcutaneous tissues	Immunology and infection	Oesophagus	2.63	2.28	2.97
Infections of other or unspecified organs	Immunology and infection	Oesophagus	4.73	4.27	5.19
Infections of the digestive system	Immunology and infection	Oesophagus	2.59	2.25	2.93
Infections of the heart	Immunology and infection	Oesophagus	1.03	0.81	1.24
Lower respiratory tract infections	Immunology and infection	Oesophagus	7.42	6.85	7.99
Lupus erythematosus (local and systemic)	Immunology and infection	Oesophagus	0.09	0.02	0.15
Meningitis	Immunology and infection	Oesophagus	1.03	0.81	1.24
Non-acute cystitis	Immunology and infection	Oesophagus	0.27	0.16	0.38
Peritonitis	Immunology and infection	Oesophagus	5.79	5.29	6.30
Systemic sclerosis	Immunology and infection	Oesophagus	0.01	0.00	0.04
Viral diseases (excluding chronic hepatitis/HIV)	Immunology and infection	Oesophagus	0.68	0.51	0.86
Ankylosing spondylitis	Musculoskeletal	Oesophagus	0.02	0.00	0.06
Enteropathic arthropathy	Musculoskeletal	Oesophagus	0.13	0.06	0.21
Enthesopathies and synovial disorders	Musculoskeletal	Oesophagus	5.18	4.70	5.66
Osteoarthritis (excluding spine)	Musculoskeletal	Oesophagus	6.86	6.31	7.40
Osteoporosis	Musculoskeletal	Oesophagus	2.51	2.17	2.84

Rheumatoid arthritis	Musculoskeletal	Oesophagus	0.78	0.59	0.97
Scoliosis	Musculoskeletal	Oesophagus	0.18	0.09	0.28
Spinal stenosis	Musculoskeletal	Oesophagus	0.76	0.57	0.95
Bell's palsy	Neurological	Oesophagus	0.16	0.07	0.25
Dementia	Neurological	Oesophagus	1.05	0.83	1.27
Disorders of autonomic nervous system	Neurological	Oesophagus	0.27	0.16	0.38
Epilepsy	Neurological	Oesophagus	0.35	0.23	0.48
Essential tremor	Neurological	Oesophagus	0.17	0.08	0.26
Intracerebral haemorrhage	Neurological	Oesophagus	0.53	0.37	0.68
Intracranial hypertension	Neurological	Oesophagus	0.11	0.04	0.18
Multiple sclerosis	Neurological	Oesophagus	0.02	0.00	0.06
Peripheral neuropathies	Neurological	Oesophagus	1.97	1.67	2.27
Subarachnoid haemorrhage	Neurological	Oesophagus	0.43	0.29	0.57
Asthma	Pulmonary	Oesophagus	2.75	2.40	3.10
Bronchiectasis	Pulmonary	Oesophagus	0.46	0.32	0.61
COPD	Pulmonary	Oesophagus	5.49	4.99	5.98
Other interstitial pulmonary diseases with fibrosis	Pulmonary	Oesophagus	0.22	0.12	0.32
End stage renal disease	Renal	Oesophagus	2.42	2.09	2.75
Glomerulonephritis	Renal	Oesophagus	0.53	0.37	0.68
Obstructive and reflux uropathy	Renal	Oesophagus	0.28	0.17	0.40
Urolithiasis	Renal	Oesophagus	0.90	0.70	1.11
Abdominal aortic aneurysm	Cardiovascular	Oropharynx	1.16	0.93	1.40
Atrial fibrillation	Cardiovascular	Oropharynx	3.14	2.75	3.53
Atrioventricular block, complete	Cardiovascular	Oropharynx	0.65	0.47	0.83
Atrioventricular block, first degree	Cardiovascular	Oropharynx	0.65	0.47	0.83
Atrioventricular block, second degree	Cardiovascular	Oropharynx	0.65	0.47	0.83
Bifascicular block	Cardiovascular	Oropharynx	0.36	0.23	0.50
Coronary heart disease not otherwise specified	Cardiovascular	Oropharynx	7.24	6.67	7.82
Dilated cardiomyopathy	Cardiovascular	Oropharynx	0.21	0.11	0.31
Heart failure	Cardiovascular	Oropharynx	2.42	2.08	2.76
Hypertension	Cardiovascular	Oropharynx	11.16	10.46	11.87
Hypertrophic cardiomyopathy	Cardiovascular	Oropharynx	0.19	0.10	0.29
Ischaemic stroke	Cardiovascular	Oropharynx	1.01	0.79	1.23
Left bundle branch block	Cardiovascular	Oropharynx	0.69	0.50	0.87
Multiple valve disease	Cardiovascular	Oropharynx	0.27	0.16	0.39
Myocardial infarction	Cardiovascular	Oropharynx	3.83	3.40	4.26
Nonrheumatic aortic valve disorders	Cardiovascular	Oropharynx	0.48	0.32	0.63
Nonrheumatic mitral valve disorders	Cardiovascular	Oropharynx	0.44	0.29	0.59
Other cardiomyopathy	Cardiovascular	Oropharynx	4.26	3.81	4.71
Pericardial effusion (noninflammatory)	Cardiovascular	Oropharynx	0.13	0.05	0.21
Peripheral arterial disease	Cardiovascular	Oropharynx	2.81	2.44	3.18
Primary pulmonary hypertension	Cardiovascular	Oropharynx	0.10	0.03	0.18
Raynaud's syndrome	Cardiovascular	Oropharynx	1.73	1.44	2.02
Right bundle branch block	Cardiovascular	Oropharynx	0.36	0.23	0.50
Secondary pulmonary hypertension	Cardiovascular	Oropharynx	0.10	0.03	0.18
Sick sinus syndrome	Cardiovascular	Oropharynx	0.25	0.14	0.36
Stable angina	Cardiovascular	Oropharynx	3.22	2.83	3.61
Stroke (not otherwise specified)	Cardiovascular	Oropharynx	1.38	1.12	1.64
Supraventricular tachycardia	Cardiovascular	Oropharynx	0.45	0.30	0.60
Transient ischaemic attack	Cardiovascular	Oropharynx	1.84	1.54	2.14
Trifascicular block	Cardiovascular	Oropharynx	0.35	0.22	0.48
Unstable angina	Cardiovascular	Oropharynx	3.03	2.65	3.41
Venous thromboembolic disease	Cardiovascular	Oropharynx	1.60	1.32	1.88
Ventricular tachycardia	Cardiovascular	Oropharynx	0.45	0.30	0.60
Diabetes	Endocrine	Oropharynx	5.41	4.90	5.91
Diabetic neurological complications	Endocrine	Oropharynx	4.02	3.59	4.46
Diabetic ophthalmic complications	Endocrine	Oropharynx	4.49	4.03	4.95
Hyperparathyroidism	Endocrine	Oropharynx	0.13	0.05	0.21
Hypo or hyperthyroidism	Endocrine	Oropharynx	2.08	1.76	2.40
Alcoholic liver disease	Gastrointestinal	Oropharynx	1.63	1.35	1.91
Angiodysplasia of colon	Gastrointestinal	Oropharynx	0.23	0.13	0.34

Barrett's oesophagus	Gastrointestinal	Oropharynx	1.53	1.25	1.80
Cholangitis	Gastrointestinal	Oropharynx	1.23	0.98	1.47
Cholecystitis	Gastrointestinal	Oropharynx	1.19	0.95	1.43
Cholelithiasis	Gastrointestinal	Oropharynx	1.35	1.09	1.60
Coeliac disease	Gastrointestinal	Oropharynx	0.35	0.22	0.48
Crohn's disease	Gastrointestinal	Oropharynx	0.08	0.02	0.14
Diverticular disease of intestine	Gastrointestinal	Oropharynx	2.85	2.48	3.22
Fatty liver	Gastrointestinal	Oropharynx	2.04	1.73	2.36
Gastritis and duodenitis	Gastrointestinal	Oropharynx	3.98	3.55	4.42
Gastrooesophageal reflux disease	Gastrointestinal	Oropharynx	3.40	3.00	3.81
Hepatic failure	Gastrointestinal	Oropharynx	1.81	1.51	2.11
Liver fibrosis sclerosis and cirrhosis	Gastrointestinal	Oropharynx	1.55	1.28	1.83
Oesophageal varices	Gastrointestinal	Oropharynx	0.40	0.26	0.54
Oesophagitis and oesophageal ulcer	Gastrointestinal	Oropharynx	4.62	4.15	5.09
Portal hypertension	Gastrointestinal	Oropharynx	0.97	0.75	1.19
Ulcerative colitis	Gastrointestinal	Oropharynx	0.14	0.06	0.23
Agranulocytosis	Haematological	Oropharynx	0.21	0.11	0.31
Aplastic anaemias	Haematological	Oropharynx	0.05	0.00	0.10
Other anaemias	Haematological	Oropharynx	2.88	2.51	3.26
Other haemolytic anaemias	Haematological	Oropharynx	0.03	0.00	0.06
Primary or idiopathic thrombocytopaenia	Haematological	Oropharynx	0.16	0.07	0.24
Secondary or other thrombocytopaenia	Haematological	Oropharynx	0.32	0.20	0.45
Secondary polycythaemia	Haematological	Oropharynx	0.23	0.13	0.34
Thrombophilia	Haematological	Oropharynx	0.26	0.15	0.37
Autoimmune liver disease	Immunology and infection	Oropharynx	0.40	0.26	0.54
Chronic viral hepatitis	Immunology and infection	Oropharynx	0.49	0.34	0.65
Encephalitis	Immunology and infection	Oropharynx	0.13	0.05	0.21
HIV	Immunology and infection	Oropharynx	0.08	0.02	0.14
Immunodeficiencies	Immunology and infection	Oropharynx	0.04	0.00	0.08
Infection of anal and rectal regions	Immunology and infection	Oropharynx	0.21	0.11	0.31
Infection of bones and joints	Immunology and infection	Oropharynx	0.38	0.24	0.51
Infection of liver	Immunology and infection	Oropharynx	0.23	0.13	0.34
Infection of skin and subcutaneous tissues	Immunology and infection	Oropharynx	2.41	2.06	2.75
Infections of other or unspecified organs	Immunology and infection	Oropharynx	3.95	3.51	4.38
Infections of the digestive system	Immunology and infection	Oropharynx	2.02	1.70	2.33
Infections of the heart	Immunology and infection	Oropharynx	0.89	0.68	1.10
Lower respiratory tract infections	Immunology and infection	Oropharynx	6.13	5.60	6.67
Lupus erythematosus (local and systemic)	Immunology and infection	Oropharynx	0.05	0.00	0.10
Meningitis	Immunology and infection	Oropharynx	0.70	0.51	0.88
Non-acute cystitis	Immunology and infection	Oropharynx	0.16	0.07	0.24
Peritonitis	Immunology and infection	Oropharynx	4.40	3.94	4.86
Systemic sclerosis	Immunology and infection	Oropharynx	0.03	0.00	0.06
Viral diseases (excluding chronic hepatitis/HIV)	Immunology and infection	Oropharynx	0.78	0.58	0.97
Ankylosing spondylitis	Musculoskeletal	Oropharynx	0.01	0.00	0.04
Enteropathic arthropathy	Musculoskeletal	Oropharynx	0.16	0.07	0.24
Enthesopathies and synovial disorders	Musculoskeletal	Oropharynx	5.14	4.64	5.63
Osteoarthritis (excluding spine)	Musculoskeletal	Oropharynx	5.71	5.19	6.22
Osteoporosis	Musculoskeletal	Oropharynx	2.12	1.80	2.44
Rheumatoid arthritis	Musculoskeletal	Oropharynx	0.62	0.45	0.80
Scoliosis	Musculoskeletal	Oropharynx	0.18	0.09	0.28
Spinal stenosis	Musculoskeletal	Oropharynx	0.47	0.31	0.62
Bell's palsy	Neurological	Oropharynx	0.57	0.40	0.74
Dementia	Neurological	Oropharynx	0.76	0.57	0.96
Disorders of autonomic nervous system	Neurological	Oropharynx	0.40	0.26	0.54
Epilepsy	Neurological	Oropharynx	0.71	0.52	0.90
Essential tremor	Neurological	Oropharynx	0.14	0.06	0.23
Intracerebral haemorrhage	Neurological	Oropharynx	0.50	0.35	0.66
Intracranial hypertension	Neurological	Oropharynx	0.18	0.09	0.28
Multiple sclerosis	Neurological	Oropharynx	0.10	0.03	0.18
Peripheral neuropathies	Neurological	Oropharynx	1.67	1.38	1.95
Subarachnoid haemorrhage	Neurological	Oropharynx	0.40	0.26	0.54
Asthma	Pulmonary	Oropharynx	2.57	2.22	2.93

Bronchiectasis	Pulmonary	Oropharynx	0.44	0.29	0.59
COPD	Pulmonary	Oropharynx	5.37	4.87	5.87
Other interstitial pulmonary diseases with fibrosis	Pulmonary	Oropharynx	0.26	0.15	0.37
End stage renal disease	Renal	Oropharynx	1.72	1.43	2.01
Glomerulonephritis	Renal	Oropharynx	0.27	0.16	0.39
Obstructive and reflux uropathy	Renal	Oropharynx	0.18	0.09	0.28
Urolithiasis	Renal	Oropharynx	0.67	0.49	0.85
Abdominal aortic aneurysm	Cardiovascular	Ovary	0.28	0.16	0.40
Atrial fibrillation	Cardiovascular	Ovary	2.61	2.26	2.97
Atrioventricular block, complete	Cardiovascular	Ovary	0.44	0.29	0.58
Atrioventricular block, first degree	Cardiovascular	Ovary	0.44	0.29	0.58
Atrioventricular block, second degree	Cardiovascular	Ovary	0.44	0.29	0.58
Bifascicular block	Cardiovascular	Ovary	0.24	0.13	0.35
Coronary heart disease not otherwise specified	Cardiovascular	Ovary	6.06	5.53	6.59
Dilated cardiomyopathy	Cardiovascular	Ovary	0.06	0.01	0.12
Heart failure	Cardiovascular	Ovary	1.99	1.68	2.30
Hypertension	Cardiovascular	Ovary	10.80	10.11	11.49
Hypertrophic cardiomyopathy	Cardiovascular	Ovary	0.06	0.01	0.12
Ischaemic stroke	Cardiovascular	Ovary	0.51	0.35	0.67
Left bundle branch block	Cardiovascular	Ovary	0.51	0.35	0.67
Multiple valve disease	Cardiovascular	Ovary	0.42	0.28	0.57
Myocardial infarction	Cardiovascular	Ovary	2.29	1.96	2.63
Nonrheumatic aortic valve disorders	Cardiovascular	Ovary	0.59	0.42	0.76
Nonrheumatic mitral valve disorders	Cardiovascular	Ovary	0.47	0.32	0.63
Other cardiomyopathy	Cardiovascular	Ovary	2.50	2.15	2.85
Pericardial effusion (noninflammatory)	Cardiovascular	Ovary	0.18	0.09	0.27
Peripheral arterial disease	Cardiovascular	Ovary	0.94	0.72	1.15
Primary pulmonary hypertension	Cardiovascular	Ovary	0.09	0.02	0.16
Raynaud's syndrome	Cardiovascular	Ovary	0.83	0.63	1.03
Right bundle branch block	Cardiovascular	Ovary	0.24	0.13	0.35
Secondary pulmonary hypertension	Cardiovascular	Ovary	0.10	0.03	0.17
Sick sinus syndrome	Cardiovascular	Ovary	0.29	0.17	0.42
Stable angina	Cardiovascular	Ovary	2.37	2.03	2.71
Stroke (not otherwise specified)	Cardiovascular	Ovary	0.85	0.64	1.05
Supraventricular tachycardia	Cardiovascular	Ovary	0.44	0.29	0.58
Transient ischaemic attack	Cardiovascular	Ovary	1.20	0.96	1.45
Trifascicular block	Cardiovascular	Ovary	0.22	0.11	0.32
Unstable angina	Cardiovascular	Ovary	2.17	1.84	2.49
Venous thromboembolic disease	Cardiovascular	Ovary	2.77	2.40	3.13
Ventricular tachycardia	Cardiovascular	Ovary	0.44	0.29	0.58
Diabetes	Endocrine	Ovary	5.13	4.64	5.62
Diabetic neurological complications	Endocrine	Ovary	4.18	3.73	4.62
Diabetic ophthalmic complications	Endocrine	Ovary	4.58	4.11	5.04
Hyperparathyroidism	Endocrine	Ovary	0.14	0.06	0.22
Hypo or hyperthyroidism	Endocrine	Ovary	3.52	3.12	3.93
Alcoholic liver disease	Gastrointestinal	Ovary	0.21	0.10	0.31
Angiodysplasia of colon	Gastrointestinal	Ovary	0.13	0.05	0.21
Barrett's oesophagus	Gastrointestinal	Ovary	0.91	0.70	1.12
Cholangitis	Gastrointestinal	Ovary	2.17	1.84	2.49
Cholecystitis	Gastrointestinal	Ovary	2.41	2.07	2.75
Cholelithiasis	Gastrointestinal	Ovary	2.78	2.42	3.15
Coeliac disease	Gastrointestinal	Ovary	0.40	0.26	0.54
Crohn's disease	Gastrointestinal	Ovary	0.12	0.04	0.19
Diverticular disease of intestine	Gastrointestinal	Ovary	5.13	4.64	5.62
Fatty liver	Gastrointestinal	Ovary	1.33	1.08	1.59
Gastritis and duodenitis	Gastrointestinal	Ovary	3.54	3.13	3.95
Gastrooesophageal reflux disease	Gastrointestinal	Ovary	3.72	3.30	4.14
Hepatic failure	Gastrointestinal	Ovary	1.24	1.00	1.49
Liver fibrosis sclerosis and cirrhosis	Gastrointestinal	Ovary	0.18	0.09	0.27
Oesophageal varices	Gastrointestinal	Ovary	0.05	0.00	0.10

Oesophagitis and oesophageal ulcer	Gastrointestinal	Ovary	4.83	4.36	5.31
Portal hypertension	Gastrointestinal	Ovary	1.13	0.89	1.36
Ulcerative colitis	Gastrointestinal	Ovary	0.19	0.10	0.29
Agranulocytosis	Haematological	Ovary	0.18	0.09	0.27
Aplastic anaemias	Haematological	Ovary	0.04	0.00	0.08
Other anaemias	Haematological	Ovary	4.43	3.98	4.89
Other haemolytic anaemias	Haematological	Ovary	0.06	0.01	0.12
Primary or idiopathic thrombocytopaenia	Haematological	Ovary	0.21	0.10	0.31
Secondary or other thrombocytopaenia	Haematological	Ovary	0.28	0.16	0.40
Secondary polycythaemia	Haematological	Ovary	0.10	0.03	0.17
Thrombophilia	Haematological	Ovary	0.09	0.02	0.16
Autoimmune liver disease	Immunology and infection	Ovary	0.15	0.07	0.24
Chronic viral hepatitis	Immunology and infection	Ovary	0.33	0.21	0.46
Encephalitis	Immunology and infection	Ovary	0.17	0.08	0.26
HIV	Immunology and infection	Ovary	0.12	0.04	0.19
Immunodeficiencies	Immunology and infection	Ovary	0.01	0.00	0.04
Infection of anal and rectal regions	Immunology and infection	Ovary	0.08	0.02	0.14
Infection of bones and joints	Immunology and infection	Ovary	0.23	0.12	0.34
Infection of liver	Immunology and infection	Ovary	0.10	0.03	0.17
Infection of skin and subcutaneous tissues	Immunology and infection	Ovary	1.79	1.50	2.09
Infections of other or unspecified organs	Immunology and infection	Ovary	3.79	3.37	4.22
Infections of the digestive system	Immunology and infection	Ovary	2.38	2.05	2.72
Infections of the heart	Immunology and infection	Ovary	0.45	0.30	0.60
Lower respiratory tract infections	Immunology and infection	Ovary	4.59	4.12	5.05
Lupus erythematosus (local and systemic)	Immunology and infection	Ovary	0.06	0.01	0.12
Meningitis	Immunology and infection	Ovary	0.55	0.39	0.72
Non-acute cystitis	Immunology and infection	Ovary	0.31	0.18	0.43
Peritonitis	Immunology and infection	Ovary	6.04	5.51	6.56
Systemic sclerosis	Immunology and infection	Ovary	0.04	0.00	0.08
Viral diseases (excluding chronic hepatitis/HIV)	Immunology and infection	Ovary	0.62	0.44	0.79
Ankylosing spondylitis	Musculoskeletal	Ovary	0.08	0.02	0.14
Enteropathic arthropathy	Musculoskeletal	Ovary	0.17	0.08	0.26
Enthesopathies and synovial disorders	Musculoskeletal	Ovary	4.93	4.45	5.41
Osteoarthritis (excluding spine)	Musculoskeletal	Ovary	7.20	6.63	7.78
Osteoporosis	Musculoskeletal	Ovary	2.64	2.28	3.00
Rheumatoid arthritis	Musculoskeletal	Ovary	0.78	0.59	0.98
Scoliosis	Musculoskeletal	Ovary	0.15	0.07	0.24
Spinal stenosis	Musculoskeletal	Ovary	0.37	0.24	0.51
Bell's palsy	Neurological	Ovary	0.21	0.10	0.31
Dementia	Neurological	Ovary	0.45	0.30	0.60
Disorders of autonomic nervous system	Neurological	Ovary	0.27	0.15	0.38
Epilepsy	Neurological	Ovary	0.35	0.22	0.48
Essential tremor	Neurological	Ovary	0.15	0.07	0.24
Intracerebral haemorrhage	Neurological	Ovary	0.32	0.19	0.45
Intracranial hypertension	Neurological	Ovary	0.08	0.02	0.14
Multiple sclerosis	Neurological	Ovary	0.04	0.00	0.08
Peripheral neuropathies	Neurological	Ovary	1.49	1.22	1.76
Subarachnoid haemorrhage	Neurological	Ovary	0.31	0.18	0.43
Asthma	Pulmonary	Ovary	3.17	2.78	3.55
Bronchiectasis	Pulmonary	Ovary	0.24	0.13	0.35
COPD	Pulmonary	Ovary	2.70	2.34	3.06
Other interstitial pulmonary diseases with fibrosis	Pulmonary	Ovary	0.14	0.06	0.22
End stage renal disease	Renal	Ovary	1.51	1.24	1.78
Glomerulonephritis	Renal	Ovary	0.24	0.13	0.35
Obstructive and reflux uropathy	Renal	Ovary	1.81	1.51	2.10
Urolithiasis	Renal	Ovary	2.01	1.70	2.32
Abdominal aortic aneurysm	Cardiovascular	Pancreas	2.04	1.64	2.45
Atrial fibrillation	Cardiovascular	Pancreas	4.17	3.60	4.75
Atrioventricular block, complete	Cardiovascular	Pancreas	0.84	0.58	1.10
Atrioventricular block, first degree	Cardiovascular	Pancreas	0.84	0.58	1.10
Atrioventricular block, second degree	Cardiovascular	Pancreas	0.84	0.58	1.10
Bifascicular block	Cardiovascular	Pancreas	0.69	0.45	0.93

Coronary heart disease not otherwise specified	Cardiovascular	Pancreas	7.59	6.83	8.36
Dilated cardiomyopathy	Cardiovascular	Pancreas	0.04	0.00	0.10
Heart failure	Cardiovascular	Pancreas	3.06	2.56	3.55
Hypertension	Cardiovascular	Pancreas	14.59	13.57	15.60
Hypertrophic cardiomyopathy	Cardiovascular	Pancreas	0.06	0.00	0.14
Ischaemic stroke	Cardiovascular	Pancreas	1.14	0.84	1.45
Left bundle branch block	Cardiovascular	Pancreas	0.86	0.60	1.13
Multiple valve disease	Cardiovascular	Pancreas	0.54	0.33	0.75
Myocardial infarction	Cardiovascular	Pancreas	4.84	4.22	5.46
Nonrheumatic aortic valve disorders	Cardiovascular	Pancreas	0.80	0.54	1.05
Nonrheumatic mitral valve disorders	Cardiovascular	Pancreas	0.60	0.38	0.82
Other cardiomyopathy	Cardiovascular	Pancreas	5.64	4.97	6.30
Pericardial effusion (noninflammatory)	Cardiovascular	Pancreas	0.19	0.07	0.32
Peripheral arterial disease	Cardiovascular	Pancreas	1.91	1.52	2.31
Primary pulmonary hypertension	Cardiovascular	Pancreas	0.13	0.03	0.23
Raynaud's syndrome	Cardiovascular	Pancreas	1.55	1.19	1.90
Right bundle branch block	Cardiovascular	Pancreas	0.69	0.45	0.93
Secondary pulmonary hypertension	Cardiovascular	Pancreas	0.11	0.01	0.20
Sick sinus syndrome	Cardiovascular	Pancreas	0.45	0.26	0.64
Stable angina	Cardiovascular	Pancreas	3.81	3.26	4.36
Stroke (not otherwise specified)	Cardiovascular	Pancreas	1.64	1.27	2.00
Supraventricular tachycardia	Cardiovascular	Pancreas	0.45	0.26	0.64
Transient ischaemic attack	Cardiovascular	Pancreas	2.37	1.93	2.80
Trifascicular block	Cardiovascular	Pancreas	0.58	0.36	0.80
Unstable angina	Cardiovascular	Pancreas	4.07	3.50	4.63
Venous thromboembolic disease	Cardiovascular	Pancreas	3.94	3.38	4.50
Ventricular tachycardia	Cardiovascular	Pancreas	0.45	0.26	0.64
Diabetes	Endocrine	Pancreas	15.81	14.76	16.86
Diabetic neurological complications	Endocrine	Pancreas	14.24	13.24	15.25
Diabetic ophthalmic complications	Endocrine	Pancreas	14.54	13.53	15.56
Hyperparathyroidism	Endocrine	Pancreas	0.22	0.08	0.35
Hypo or hyperthyroidism	Endocrine	Pancreas	2.24	1.81	2.66
Alcoholic liver disease	Gastrointestinal	Pancreas	0.58	0.36	0.80
Angiodysplasia of colon	Gastrointestinal	Pancreas	0.41	0.23	0.59
Barrett's oesophagus	Gastrointestinal	Pancreas	2.84	2.36	3.32
Cholangitis	Gastrointestinal	Pancreas	26.87	25.60	28.15
Cholecystitis	Gastrointestinal	Pancreas	9.27	8.44	10.11
Cholelithiasis	Gastrointestinal	Pancreas	8.37	7.57	9.17
Coeliac disease	Gastrointestinal	Pancreas	1.10	0.80	1.40
Crohn's disease	Gastrointestinal	Pancreas	0.13	0.03	0.23
Diverticular disease of intestine	Gastrointestinal	Pancreas	7.06	6.32	7.79
Fatty liver	Gastrointestinal	Pancreas	4.82	4.20	5.44
Gastritis and duodenitis	Gastrointestinal	Pancreas	10.99	10.09	11.89
Gastrooesophageal reflux disease	Gastrointestinal	Pancreas	7.21	6.46	7.95
Hepatic failure	Gastrointestinal	Pancreas	4.91	4.28	5.53
Liver fibrosis sclerosis and cirrhosis	Gastrointestinal	Pancreas	0.67	0.43	0.90
Oesophageal varices	Gastrointestinal	Pancreas	0.39	0.21	0.57
Oesophagitis and oesophageal ulcer	Gastrointestinal	Pancreas	9.81	8.96	10.67
Portal hypertension	Gastrointestinal	Pancreas	4.11	3.54	4.68
Ulcerative colitis	Gastrointestinal	Pancreas	0.37	0.19	0.54
Agranulocytosis	Haematological	Pancreas	0.24	0.10	0.38
Aplastic anaemias	Haematological	Pancreas	0.13	0.03	0.23
Other anaemias	Haematological	Pancreas	5.19	4.55	5.82
Other haemolytic anaemias	Haematological	Pancreas	0.02	0.00	0.06
Primary or idiopathic thrombocytopaenia	Haematological	Pancreas	0.15	0.04	0.26
Secondary or other thrombocytopaenia	Haematological	Pancreas	0.26	0.11	0.40
Secondary polycythaemia	Haematological	Pancreas	0.06	0.00	0.14
Thrombophilia	Haematological	Pancreas	0.28	0.13	0.43
Autoimmune liver disease	Immunology and infection	Pancreas	0.65	0.42	0.88
Chronic viral hepatitis	Immunology and infection	Pancreas	3.46	2.94	3.99
Encephalitis	Immunology and infection	Pancreas	0.13	0.03	0.23

HIV	Immunology and infection	Pancreas	0.13	0.03	0.23
Immunodeficiencies	Immunology and infection	Pancreas	0.04	0.00	0.10
Infection of anal and rectal regions	Immunology and infection	Pancreas	0.09	0.00	0.17
Infection of bones and joints	Immunology and infection	Pancreas	0.56	0.34	0.77
Infection of liver	Immunology and infection	Pancreas	0.56	0.34	0.77
Infection of skin and subcutaneous tissues	Immunology and infection	Pancreas	2.45	2.01	2.90
Infections of other or unspecified organs	Immunology and infection	Pancreas	5.66	4.99	6.32
Infections of the digestive system	Immunology and infection	Pancreas	4.54	3.94	5.14
Infections of the heart	Immunology and infection	Pancreas	0.99	0.71	1.27
Lower respiratory tract infections	Immunology and infection	Pancreas	7.23	6.48	7.97
Lupus erythematosus (local and systemic)	Immunology and infection	Pancreas	0.15	0.04	0.26
Meningitis	Immunology and infection	Pancreas	1.05	0.76	1.35
Non-acute cystitis	Immunology and infection	Pancreas	0.52	0.31	0.72
Peritonitis	Immunology and infection	Pancreas	10.71	9.83	11.60
Systemic sclerosis	Immunology and infection	Pancreas	0.02	0.00	0.06
Viral diseases (excluding chronic hepatitis/HIV)	Immunology and infection	Pancreas	0.67	0.43	0.90
Ankylosing spondylitis	Musculoskeletal	Pancreas	0.09	0.00	0.17
Enteropathic arthropathy	Musculoskeletal	Pancreas	0.11	0.01	0.20
Enthesopathies and synovial disorders	Musculoskeletal	Pancreas	5.81	5.14	6.48
Osteoarthritis (excluding spine)	Musculoskeletal	Pancreas	8.20	7.41	8.99
Osteoporosis	Musculoskeletal	Pancreas	3.40	2.88	3.92
Rheumatoid arthritis	Musculoskeletal	Pancreas	0.82	0.56	1.08
Scoliosis	Musculoskeletal	Pancreas	0.17	0.05	0.29
Spinal stenosis	Musculoskeletal	Pancreas	0.69	0.45	0.93
Bell's palsy	Neurological	Pancreas	0.13	0.03	0.23
Dementia	Neurological	Pancreas	0.82	0.56	1.08
Disorders of autonomic nervous system	Neurological	Pancreas	0.41	0.23	0.59
Epilepsy	Neurological	Pancreas	0.45	0.26	0.64
Essential tremor	Neurological	Pancreas	0.19	0.07	0.32
Intracerebral haemorrhage	Neurological	Pancreas	0.52	0.31	0.72
Intracranial hypertension	Neurological	Pancreas	0.09	0.00	0.17
Multiple sclerosis	Neurological	Pancreas	0.06	0.00	0.14
Peripheral neuropathies	Neurological	Pancreas	2.13	1.71	2.55
Subarachnoid haemorrhage	Neurological	Pancreas	0.37	0.19	0.54
Asthma	Pulmonary	Pancreas	2.62	2.17	3.08
Bronchiectasis	Pulmonary	Pancreas	0.30	0.14	0.46
COPD	Pulmonary	Pancreas	4.37	3.78	4.96
Other interstitial pulmonary diseases with fibrosis	Pulmonary	Pancreas	0.22	0.08	0.35
End stage renal disease	Renal	Pancreas	2.99	2.50	3.48
Glomerulonephritis	Renal	Pancreas	0.69	0.45	0.93
Obstructive and reflux uropathy	Renal	Pancreas	0.95	0.67	1.23
Urolithiasis	Renal	Pancreas	2.22	1.79	2.64
Abdominal aortic aneurysm	Cardiovascular	Prostate	2.37	2.22	2.52
Atrial fibrillation	Cardiovascular	Prostate	4.27	4.07	4.47
Atrioventricular block, complete	Cardiovascular	Prostate	0.90	0.81	0.99
Atrioventricular block, first degree	Cardiovascular	Prostate	0.90	0.81	0.99
Atrioventricular block, second degree	Cardiovascular	Prostate	0.90	0.81	0.99
Bifascicular block	Cardiovascular	Prostate	0.72	0.64	0.80
Coronary heart disease not otherwise specified	Cardiovascular	Prostate	8.05	7.79	8.32
Dilated cardiomyopathy	Cardiovascular	Prostate	0.17	0.13	0.21
Heart failure	Cardiovascular	Prostate	2.97	2.81	3.13
Hypertension	Cardiovascular	Prostate	14.10	13.76	14.44
Hypertrophic cardiomyopathy	Cardiovascular	Prostate	0.16	0.12	0.20
Ischaemic stroke	Cardiovascular	Prostate	0.95	0.85	1.04
Left bundle branch block	Cardiovascular	Prostate	0.99	0.90	1.09
Multiple valve disease	Cardiovascular	Prostate	0.44	0.38	0.51
Myocardial infarction	Cardiovascular	Prostate	4.98	4.77	5.19
Nonrheumatic aortic valve disorders	Cardiovascular	Prostate	0.81	0.72	0.89
Nonrheumatic mitral valve disorders	Cardiovascular	Prostate	0.52	0.45	0.59
Other cardiomyopathy	Cardiovascular	Prostate	5.80	5.57	6.02
Pericardial effusion (noninflammatory)	Cardiovascular	Prostate	0.12	0.09	0.15
Peripheral arterial disease	Cardiovascular	Prostate	1.58	1.46	1.70

Primary pulmonary hypertension	Cardiovascular	Prostate	0.12	0.09	0.15
Raynaud's syndrome	Cardiovascular	Prostate	0.96	0.86	1.05
Right bundle branch block	Cardiovascular	Prostate	0.71	0.63	0.79
Secondary pulmonary hypertension	Cardiovascular	Prostate	0.10	0.07	0.14
Sick sinus syndrome	Cardiovascular	Prostate	0.40	0.33	0.46
Stable angina	Cardiovascular	Prostate	4.10	3.91	4.29
Stroke (not otherwise specified)	Cardiovascular	Prostate	1.61	1.48	1.73
Supraventricular tachycardia	Cardiovascular	Prostate	0.43	0.37	0.49
Transient ischaemic attack	Cardiovascular	Prostate	2.44	2.30	2.59
Trifascicular block	Cardiovascular	Prostate	0.58	0.50	0.65
Unstable angina	Cardiovascular	Prostate	3.99	3.80	4.18
Venous thromboembolic disease	Cardiovascular	Prostate	1.91	1.78	2.05
Ventricular tachycardia	Cardiovascular	Prostate	0.45	0.38	0.51
Diabetes	Endocrine	Prostate	6.41	6.17	6.64
Diabetic neurological complications	Endocrine	Prostate	4.33	4.13	4.52
Diabetic ophthalmic complications	Endocrine	Prostate	5.08	4.87	5.30
Hyperparathyroidism	Endocrine	Prostate	0.11	0.08	0.14
Hypo or hyperthyroidism	Endocrine	Prostate	1.15	1.05	1.25
Alcoholic liver disease	Gastrointestinal	Prostate	0.21	0.17	0.26
Angiodysplasia of colon	Gastrointestinal	Prostate	0.13	0.09	0.16
Barrett's oesophagus	Gastrointestinal	Prostate	1.33	1.22	1.44
Cholangitis	Gastrointestinal	Prostate	1.18	1.07	1.28
Cholecystitis	Gastrointestinal	Prostate	1.27	1.16	1.38
Cholelithiasis	Gastrointestinal	Prostate	1.52	1.40	1.64
Coeliac disease	Gastrointestinal	Prostate	0.20	0.15	0.24
Crohn's disease	Gastrointestinal	Prostate	0.10	0.07	0.14
Diverticular disease of intestine	Gastrointestinal	Prostate	3.63	3.45	3.81
Fatty liver	Gastrointestinal	Prostate	0.57	0.49	0.64
Gastritis and duodenitis	Gastrointestinal	Prostate	3.28	3.10	3.45
Gastrooesophageal reflux disease	Gastrointestinal	Prostate	3.42	3.24	3.59
Hepatic failure	Gastrointestinal	Prostate	0.43	0.37	0.49
Liver fibrosis sclerosis and cirrhosis	Gastrointestinal	Prostate	0.15	0.11	0.18
Oesophageal varices	Gastrointestinal	Prostate	0.04	0.02	0.05
Oesophagitis and oesophageal ulcer	Gastrointestinal	Prostate	4.26	4.06	4.45
Portal hypertension	Gastrointestinal	Prostate	0.31	0.25	0.36
Ulcerative colitis	Gastrointestinal	Prostate	0.20	0.16	0.24
Agranulocytosis	Haematological	Prostate	0.11	0.08	0.14
Aplastic anaemias	Haematological	Prostate	0.04	0.02	0.06
Other anaemias	Haematological	Prostate	2.83	2.67	2.99
Other haemolytic anaemias	Haematological	Prostate	0.03	0.02	0.05
Primary or idiopathic thrombocytopaenia	Haematological	Prostate	0.18	0.14	0.22
Secondary or other thrombocytopaenia	Haematological	Prostate	0.32	0.27	0.37
Secondary polycythaemia	Haematological	Prostate	0.10	0.07	0.13
Thrombophilia	Haematological	Prostate	0.13	0.09	0.16
Autoimmune liver disease	Immunology and infection	Prostate	0.08	0.06	0.11
Chronic viral hepatitis	Immunology and infection	Prostate	0.31	0.25	0.36
Encephalitis	Immunology and infection	Prostate	0.09	0.06	0.12
HIV	Immunology and infection	Prostate	0.10	0.07	0.13
Immunodeficiencies	Immunology and infection	Prostate	0.01	0.00	0.03
Infection of anal and rectal regions	Immunology and infection	Prostate	0.13	0.09	0.16
Infection of bones and joints	Immunology and infection	Prostate	0.41	0.35	0.47
Infection of liver	Immunology and infection	Prostate	0.08	0.06	0.11
Infection of skin and subcutaneous tissues	Immunology and infection	Prostate	1.35	1.24	1.46
Infections of other or unspecified organs	Immunology and infection	Prostate	3.06	2.89	3.22
Infections of the digestive system	Immunology and infection	Prostate	1.82	1.69	1.95
Infections of the heart	Immunology and infection	Prostate	0.24	0.19	0.29
Lower respiratory tract infections	Immunology and infection	Prostate	4.16	3.97	4.35
Lupus erythematosus (local and systemic)	Immunology and infection	Prostate	0.03	0.01	0.05
Meningitis	Immunology and infection	Prostate	0.26	0.21	0.31
Non-acute cystitis	Immunology and infection	Prostate	0.59	0.51	0.66
Peritonitis	Immunology and infection	Prostate	4.55	4.35	4.75

Systemic sclerosis	Immunology and infection	Prostate	0.01	0.00	0.02
Viral diseases (excluding chronic hepatitis/HIV)	Immunology and infection	Prostate	0.50	0.43	0.57
Ankylosing spondylitis	Musculoskeletal	Prostate	0.04	0.02	0.06
Enteropathic arthropathy	Musculoskeletal	Prostate	0.16	0.12	0.19
Enthesopathies and synovial disorders	Musculoskeletal	Prostate	5.79	5.57	6.02
Osteoarthritis (excluding spine)	Musculoskeletal	Prostate	7.65	7.39	7.90
Osteoporosis	Musculoskeletal	Prostate	0.96	0.87	1.05
Rheumatoid arthritis	Musculoskeletal	Prostate	0.40	0.34	0.46
Scoliosis	Musculoskeletal	Prostate	0.12	0.09	0.15
Spinal stenosis	Musculoskeletal	Prostate	0.72	0.64	0.80
Bell's palsy	Neurological	Prostate	0.21	0.16	0.25
Dementia	Neurological	Prostate	0.64	0.56	0.71
Disorders of autonomic nervous system	Neurological	Prostate	0.38	0.32	0.44
Epilepsy	Neurological	Prostate	0.38	0.32	0.43
Essential tremor	Neurological	Prostate	0.17	0.13	0.21
Intracerebral haemorrhage	Neurological	Prostate	0.39	0.33	0.45
Intracranial hypertension	Neurological	Prostate	0.06	0.04	0.08
Multiple sclerosis	Neurological	Prostate	0.03	0.02	0.05
Peripheral neuropathies	Neurological	Prostate	1.74	1.61	1.86
Subarachnoid haemorrhage	Neurological	Prostate	0.32	0.26	0.37
Asthma	Pulmonary	Prostate	2.30	2.16	2.45
Bronchiectasis	Pulmonary	Prostate	0.30	0.25	0.35
COPD	Pulmonary	Prostate	3.70	3.52	3.89
Other interstitial pulmonary diseases with fibrosis	Pulmonary	Prostate	0.19	0.15	0.24
End stage renal disease	Renal	Prostate	2.01	1.88	2.15
Glomerulonephritis	Renal	Prostate	0.50	0.43	0.57
Obstructive and reflux uropathy	Renal	Prostate	1.90	1.77	2.03
Urolithiasis	Renal	Prostate	3.03	2.87	3.20
Abdominal aortic aneurysm	Cardiovascular	Stomach	1.85	1.52	2.17
Atrial fibrillation	Cardiovascular	Stomach	5.52	4.97	6.07
Atrioventricular block, complete	Cardiovascular	Stomach	1.05	0.81	1.30
Atrioventricular block, first degree	Cardiovascular	Stomach	1.05	0.81	1.30
Atrioventricular block, second degree	Cardiovascular	Stomach	1.05	0.81	1.30
Bifascicular block	Cardiovascular	Stomach	0.64	0.45	0.83
Coronary heart disease not otherwise specified	Cardiovascular	Stomach	9.47	8.76	10.18
Dilated cardiomyopathy	Cardiovascular	Stomach	0.14	0.05	0.23
Heart failure	Cardiovascular	Stomach	4.42	3.93	4.92
Hypertension	Cardiovascular	Stomach	14.05	13.21	14.89
Hypertrophic cardiomyopathy	Cardiovascular	Stomach	0.17	0.07	0.27
Ischaemic stroke	Cardiovascular	Stomach	1.37	1.09	1.65
Left bundle branch block	Cardiovascular	Stomach	1.16	0.90	1.42
Multiple valve disease	Cardiovascular	Stomach	0.93	0.70	1.16
Myocardial infarction	Cardiovascular	Stomach	6.74	6.13	7.35
Nonrheumatic aortic valve disorders	Cardiovascular	Stomach	1.14	0.89	1.40
Nonrheumatic mitral valve disorders	Cardiovascular	Stomach	0.76	0.55	0.97
Other cardiomyopathy	Cardiovascular	Stomach	7.47	6.84	8.11
Pericardial effusion (noninflammatory)	Cardiovascular	Stomach	0.11	0.03	0.19
Peripheral arterial disease	Cardiovascular	Stomach	2.10	1.76	2.45
Primary pulmonary hypertension	Cardiovascular	Stomach	0.27	0.15	0.40
Raynaud's syndrome	Cardiovascular	Stomach	1.56	1.26	1.86
Right bundle branch block	Cardiovascular	Stomach	0.64	0.45	0.83
Secondary pulmonary hypertension	Cardiovascular	Stomach	0.24	0.12	0.36
Sick sinus syndrome	Cardiovascular	Stomach	0.37	0.22	0.51
Stable angina	Cardiovascular	Stomach	5.41	4.87	5.96
Stroke (not otherwise specified)	Cardiovascular	Stomach	2.20	1.84	2.55
Supraventricular tachycardia	Cardiovascular	Stomach	0.76	0.55	0.97
Transient ischaemic attack	Cardiovascular	Stomach	2.76	2.36	3.16
Trifascicular block	Cardiovascular	Stomach	0.52	0.34	0.69
Unstable angina	Cardiovascular	Stomach	5.15	4.62	5.69
Venous thromboembolic disease	Cardiovascular	Stomach	3.40	2.96	3.84
Ventricular tachycardia	Cardiovascular	Stomach	0.81	0.59	1.03
Diabetes	Endocrine	Stomach	7.23	6.60	7.86

Diabetic neurological complications	Endocrine	Stomach	6.42	5.83	7.01
Diabetic ophthalmic complications	Endocrine	Stomach	6.91	6.29	7.52
Hyperparathyroidism	Endocrine	Stomach	0.21	0.10	0.33
Hypo or hyperthyroidism	Endocrine	Stomach	2.01	1.67	2.35
Alcoholic liver disease	Gastrointestinal	Stomach	0.32	0.18	0.46
Angiodysplasia of colon	Gastrointestinal	Stomach	0.38	0.23	0.53
Barrett's oesophagus	Gastrointestinal	Stomach	5.19	4.65	5.72
Cholangitis	Gastrointestinal	Stomach	2.49	2.11	2.86
Cholecystitis	Gastrointestinal	Stomach	2.46	2.08	2.83
Cholelithiasis	Gastrointestinal	Stomach	2.90	2.49	3.30
Coeliac disease	Gastrointestinal	Stomach	0.31	0.17	0.44
Crohn's disease	Gastrointestinal	Stomach	0.15	0.06	0.25
Diverticular disease of intestine	Gastrointestinal	Stomach	5.28	4.74	5.82
Fatty liver	Gastrointestinal	Stomach	1.33	1.05	1.60
Gastritis and duodenitis	Gastrointestinal	Stomach	11.27	10.50	12.04
Gastrooesophageal reflux disease	Gastrointestinal	Stomach	7.78	7.13	8.43
Hepatic failure	Gastrointestinal	Stomach	1.13	0.87	1.38
Liver fibrosis sclerosis and cirrhosis	Gastrointestinal	Stomach	0.27	0.15	0.40
Oesophageal varices	Gastrointestinal	Stomach	0.21	0.10	0.33
Oesophagitis and oesophageal ulcer	Gastrointestinal	Stomach	13.04	12.22	13.85
Portal hypertension	Gastrointestinal	Stomach	0.88	0.66	1.11
Ulcerative colitis	Gastrointestinal	Stomach	0.23	0.11	0.34
Agranulocytosis	Haematological	Stomach	0.20	0.09	0.31
Aplastic anaemias	Haematological	Stomach	0.08	0.01	0.14
Other anaemias	Haematological	Stomach	13.16	12.34	13.98
Other haemolytic anaemias	Haematological	Stomach	0.02	0.00	0.05
Primary or idiopathic thrombocytopaenia	Haematological	Stomach	0.24	0.12	0.36
Secondary or other thrombocytopaenia	Haematological	Stomach	0.52	0.34	0.69
Secondary polycythaemia	Haematological	Stomach	0.14	0.05	0.23
Thrombophilia	Haematological	Stomach	0.18	0.08	0.29
Autoimmune liver disease	Immunology and infection	Stomach	0.27	0.15	0.40
Chronic viral hepatitis	Immunology and infection	Stomach	0.32	0.18	0.46
Encephalitis	Immunology and infection	Stomach	0.14	0.05	0.23
HIV	Immunology and infection	Stomach	0.12	0.04	0.21
Immunodeficiencies	Immunology and infection	Stomach	0.05	0.00	0.10
Infection of anal and rectal regions	Immunology and infection	Stomach	0.09	0.02	0.16
Infection of bones and joints	Immunology and infection	Stomach	0.44	0.28	0.60
Infection of liver	Immunology and infection	Stomach	0.12	0.04	0.21
Infection of skin and subcutaneous tissues	Immunology and infection	Stomach	1.97	1.63	2.30
Infections of other or unspecified organs	Immunology and infection	Stomach	6.25	5.67	6.84
Infections of the digestive system	Immunology and infection	Stomach	3.77	3.31	4.23
Infections of the heart	Immunology and infection	Stomach	0.67	0.47	0.87
Lower respiratory tract infections	Immunology and infection	Stomach	7.76	7.12	8.41
Lupus erythematosus (local and systemic)	Immunology and infection	Stomach	0.11	0.03	0.19
Meningitis	Immunology and infection	Stomach	0.66	0.46	0.85
Non-acute cystitis	Immunology and infection	Stomach	0.31	0.17	0.44
Peritonitis	Immunology and infection	Stomach	14.61	13.76	15.47
Systemic sclerosis	Immunology and infection	Stomach	0.02	0.00	0.05
Viral diseases (excluding chronic hepatitis/HIV)	Immunology and infection	Stomach	0.64	0.45	0.83
Ankylosing spondylitis	Musculoskeletal	Stomach	0.02	0.00	0.05
Enteropathic arthropathy	Musculoskeletal	Stomach	0.08	0.01	0.14
Enthesopathies and synovial disorders	Musculoskeletal	Stomach	4.77	4.26	5.29
Osteoarthritis (excluding spine)	Musculoskeletal	Stomach	7.23	6.60	7.86
Osteoporosis	Musculoskeletal	Stomach	2.49	2.11	2.86
Rheumatoid arthritis	Musculoskeletal	Stomach	0.73	0.53	0.94
Scoliosis	Musculoskeletal	Stomach	0.14	0.05	0.23
Spinal stenosis	Musculoskeletal	Stomach	0.76	0.55	0.97
Bell's palsy	Neurological	Stomach	0.23	0.11	0.34
Dementia	Neurological	Stomach	1.07	0.82	1.32
Disorders of autonomic nervous system	Neurological	Stomach	0.34	0.20	0.48
Epilepsy	Neurological	Stomach	0.49	0.32	0.66

Essential tremor	Neurological	Stomach	0.21	0.10	0.33
Intracerebral haemorrhage	Neurological	Stomach	0.49	0.32	0.66
Intracranial hypertension	Neurological	Stomach	0.09	0.02	0.16
Peripheral neuropathies	Neurological	Stomach	1.91	1.58	2.24
Subarachnoid haemorrhage	Neurological	Stomach	0.41	0.26	0.57
Asthma	Pulmonary	Stomach	3.40	2.96	3.84
Bronchiectasis	Pulmonary	Stomach	0.49	0.32	0.66
COPD	Pulmonary	Stomach	5.52	4.97	6.07
Other interstitial pulmonary diseases with fibrosis	Pulmonary	Stomach	0.38	0.23	0.53
End stage renal disease	Renal	Stomach	2.56	2.18	2.94
Glomerulonephritis	Renal	Stomach	0.26	0.14	0.38
Obstructive and reflux uropathy	Renal	Stomach	0.73	0.53	0.94
Urolithiasis	Renal	Stomach	1.42	1.13	1.70
Abdominal aortic aneurysm	Cardiovascular	Testis	0.19	0.02	0.35
Atrial fibrillation	Cardiovascular	Testis	0.56	0.28	0.85
Bifascicular block	Cardiovascular	Testis	0.04	0.00	0.11
Coronary heart disease not otherwise specified	Cardiovascular	Testis	3.03	2.38	3.68
Dilated cardiomyopathy	Cardiovascular	Testis	0.04	0.00	0.11
Heart failure	Cardiovascular	Testis	0.34	0.12	0.56
Hypertension	Cardiovascular	Testis	3.30	2.62	3.97
Hypertrophic cardiomyopathy	Cardiovascular	Testis	0.04	0.00	0.11
Ischaemic stroke	Cardiovascular	Testis	0.11	0.00	0.24
Multiple valve disease	Cardiovascular	Testis	0.11	0.00	0.24
Myocardial infarction	Cardiovascular	Testis	0.67	0.36	0.98
Nonrheumatic aortic valve disorders	Cardiovascular	Testis	0.07	0.00	0.18
Nonrheumatic mitral valve disorders	Cardiovascular	Testis	0.07	0.00	0.18
Other cardiomyopathy	Cardiovascular	Testis	0.75	0.42	1.08
Pericardial effusion (noninflammatory)	Cardiovascular	Testis	0.04	0.00	0.11
Peripheral arterial disease	Cardiovascular	Testis	0.30	0.09	0.51
Raynaud's syndrome	Cardiovascular	Testis	0.26	0.07	0.46
Right bundle branch block	Cardiovascular	Testis	0.04	0.00	0.11
Secondary pulmonary hypertension	Cardiovascular	Testis	0.04	0.00	0.11
Stable angina	Cardiovascular	Testis	0.71	0.39	1.03
Stroke (not otherwise specified)	Cardiovascular	Testis	0.22	0.05	0.40
Supraventricular tachycardia	Cardiovascular	Testis	0.22	0.05	0.40
Transient ischaemic attack	Cardiovascular	Testis	0.19	0.02	0.35
Trifascicular block	Cardiovascular	Testis	0.04	0.00	0.11
Unstable angina	Cardiovascular	Testis	0.71	0.39	1.03
Venous thromboembolic disease	Cardiovascular	Testis	0.90	0.54	1.26
Ventricular tachycardia	Cardiovascular	Testis	0.22	0.05	0.40
Diabetes	Endocrine	Testis	1.99	1.46	2.51
Diabetic neurological complications	Endocrine	Testis	1.05	0.66	1.44
Diabetic ophthalmic complications	Endocrine	Testis	1.05	0.66	1.44
Hyperparathyroidism	Endocrine	Testis	0.07	0.00	0.18
Hypo or hyperthyroidism	Endocrine	Testis	0.22	0.05	0.40
Alcoholic liver disease	Gastrointestinal	Testis	0.11	0.00	0.24
Barrett's oesophagus	Gastrointestinal	Testis	0.49	0.22	0.75
Cholangitis	Gastrointestinal	Testis	0.52	0.25	0.80
Cholecystitis	Gastrointestinal	Testis	0.49	0.22	0.75
Cholelithiasis	Gastrointestinal	Testis	0.49	0.22	0.75
Coeliac disease	Gastrointestinal	Testis	0.30	0.09	0.51
Crohn's disease	Gastrointestinal	Testis	0.22	0.05	0.40
Diverticular disease of intestine	Gastrointestinal	Testis	0.34	0.12	0.56
Fatty liver	Gastrointestinal	Testis	0.49	0.22	0.75
Gastritis and duodenitis	Gastrointestinal	Testis	1.39	0.94	1.83
Gastrooesophageal reflux disease	Gastrointestinal	Testis	1.46	1.01	1.92
Hepatic failure	Gastrointestinal	Testis	0.41	0.17	0.65
Liver fibrosis sclerosis and cirrhosis	Gastrointestinal	Testis	0.07	0.00	0.18
Oesophagitis and oesophageal ulcer	Gastrointestinal	Testis	1.95	1.42	2.47
Portal hypertension	Gastrointestinal	Testis	0.30	0.09	0.51
Ulcerative colitis	Gastrointestinal	Testis	0.04	0.00	0.11
Agranulocytosis	Haematological	Testis	0.19	0.02	0.35

Aplastic anaemias	Haematological	Testis	0.11	0.00	0.24
Other anaemias	Haematological	Testis	0.94	0.57	1.30
Other haemolytic anaemias	Haematological	Testis	0.04	0.00	0.11
Primary or idiopathic thrombocytopaenia	Haematological	Testis	0.07	0.00	0.18
Secondary or other thrombocytopaenia	Haematological	Testis	0.15	0.00	0.30
Secondary polycythaemia	Haematological	Testis	0.11	0.00	0.24
Thrombophilia	Haematological	Testis	0.11	0.00	0.24
Chronic viral hepatitis	Immunology and infection	Testis	0.26	0.07	0.46
Encephalitis	Immunology and infection	Testis	0.11	0.00	0.24
HIV	Immunology and infection	Testis	0.11	0.00	0.24
Infection of anal and rectal regions	Immunology and infection	Testis	0.22	0.05	0.40
Infection of bones and joints	Immunology and infection	Testis	0.49	0.22	0.75
Infection of liver	Immunology and infection	Testis	0.07	0.00	0.18
Infection of skin and subcutaneous tissues	Immunology and infection	Testis	1.20	0.79	1.61
Infections of other or unspecified organs	Immunology and infection	Testis	1.24	0.82	1.66
Infections of the digestive system	Immunology and infection	Testis	0.52	0.25	0.80
Infections of the heart	Immunology and infection	Testis	0.26	0.07	0.46
Lower respiratory tract infections	Immunology and infection	Testis	0.90	0.54	1.26
Lupus erythematosus (local and systemic)	Immunology and infection	Testis	0.04	0.00	0.11
Meningitis	Immunology and infection	Testis	0.26	0.07	0.46
Peritonitis	Immunology and infection	Testis	1.20	0.79	1.61
Viral diseases (excluding chronic hepatitis/HIV)	Immunology and infection	Testis	0.52	0.25	0.80
Ankylosing spondylitis	Musculoskeletal	Testis	0.04	0.00	0.11
Enteropathic arthropathy	Musculoskeletal	Testis	0.04	0.00	0.11
Enthesopathies and synovial disorders	Musculoskeletal	Testis	4.04	3.30	4.79
Osteoarthritis (excluding spine)	Musculoskeletal	Testis	1.16	0.75	1.57
Osteoporosis	Musculoskeletal	Testis	0.30	0.09	0.51
Rheumatoid arthritis	Musculoskeletal	Testis	0.15	0.00	0.30
Scoliosis	Musculoskeletal	Testis	0.11	0.00	0.24
Spinal stenosis	Musculoskeletal	Testis	0.07	0.00	0.18
Bell's palsy	Neurological	Testis	0.22	0.05	0.40
Dementia	Neurological	Testis	0.11	0.00	0.24
Disorders of autonomic nervous system	Neurological	Testis	0.19	0.02	0.35
Epilepsy	Neurological	Testis	0.34	0.12	0.56
Intracerebral haemorrhage	Neurological	Testis	0.04	0.00	0.11
Intracranial hypertension	Neurological	Testis	0.07	0.00	0.18
Multiple sclerosis	Neurological	Testis	0.04	0.00	0.11
Peripheral neuropathies	Neurological	Testis	0.64	0.33	0.94
Asthma	Pulmonary	Testis	1.42	0.97	1.87
COPD	Pulmonary	Testis	0.67	0.36	0.98
End stage renal disease	Renal	Testis	0.52	0.25	0.80
Glomerulonephritis	Renal	Testis	0.07	0.00	0.18
Obstructive and reflux uropathy	Renal	Testis	0.79	0.45	1.12
Urolithiasis	Renal	Testis	1.57	1.10	2.05
Abdominal aortic aneurysm	Cardiovascular	Thyroid	0.51	0.24	0.79
Atrial fibrillation	Cardiovascular	Thyroid	2.53	1.92	3.14
Atrioventricular block, complete	Cardiovascular	Thyroid	0.28	0.07	0.48
Atrioventricular block, first degree	Cardiovascular	Thyroid	0.28	0.07	0.48
Atrioventricular block, second degree	Cardiovascular	Thyroid	0.28	0.07	0.48
Bifascicular block	Cardiovascular	Thyroid	0.36	0.12	0.59
Coronary heart disease not otherwise specified	Cardiovascular	Thyroid	5.33	4.46	6.21
Dilated cardiomyopathy	Cardiovascular	Thyroid	0.04	0.00	0.12
Heart failure	Cardiovascular	Thyroid	1.66	1.16	2.16
Hypertension	Cardiovascular	Thyroid	9.04	7.93	10.16
Hypertrophic cardiomyopathy	Cardiovascular	Thyroid	0.04	0.00	0.12
Ischaemic stroke	Cardiovascular	Thyroid	0.51	0.24	0.79
Left bundle branch block	Cardiovascular	Thyroid	0.36	0.12	0.59
Multiple valve disease	Cardiovascular	Thyroid	0.36	0.12	0.59
Myocardial infarction	Cardiovascular	Thyroid	2.37	1.78	2.96
Nonrheumatic aortic valve disorders	Cardiovascular	Thyroid	0.36	0.12	0.59

Nonrheumatic mitral valve disorders	Cardiovascular	Thyroid	0.47	0.21	0.74
Other cardiomyopathy	Cardiovascular	Thyroid	2.49	1.88	3.09
Pericardial effusion (noninflammatory)	Cardiovascular	Thyroid	0.08	0.00	0.19
Peripheral arterial disease	Cardiovascular	Thyroid	0.71	0.38	1.04
Primary pulmonary hypertension	Cardiovascular	Thyroid	0.16	0.00	0.31
Raynaud's syndrome	Cardiovascular	Thyroid	0.59	0.29	0.89
Right bundle branch block	Cardiovascular	Thyroid	0.36	0.12	0.59
Secondary pulmonary hypertension	Cardiovascular	Thyroid	0.16	0.00	0.31
Sick sinus syndrome	Cardiovascular	Thyroid	0.20	0.02	0.37
Stable angina	Cardiovascular	Thyroid	1.94	1.40	2.47
Stroke (not otherwise specified)	Cardiovascular	Thyroid	0.63	0.32	0.94
Supraventricular tachycardia	Cardiovascular	Thyroid	0.43	0.18	0.69
Transient ischaemic attack	Cardiovascular	Thyroid	1.22	0.80	1.65
Trifascicular block	Cardiovascular	Thyroid	0.24	0.05	0.43
Unstable angina	Cardiovascular	Thyroid	1.94	1.40	2.47
Venous thromboembolic disease	Cardiovascular	Thyroid	1.11	0.70	1.51
Ventricular tachycardia	Cardiovascular	Thyroid	0.43	0.18	0.69
Diabetes	Endocrine	Thyroid	4.74	3.91	5.57
Diabetic neurological complications	Endocrine	Thyroid	3.08	2.41	3.75
Diabetic ophthalmic complications	Endocrine	Thyroid	3.63	2.90	4.36
Hyperparathyroidism	Endocrine	Thyroid	1.58	1.09	2.07
Hypo or hyperthyroidism	Endocrine	Thyroid	16.75	15.29	18.20
Alcoholic liver disease	Gastrointestinal	Thyroid	0.55	0.26	0.84
Barrett's oesophagus	Gastrointestinal	Thyroid	0.91	0.54	1.28
Cholangitis	Gastrointestinal	Thyroid	1.50	1.03	1.97
Cholecystitis	Gastrointestinal	Thyroid	1.54	1.06	2.02
Cholelithiasis	Gastrointestinal	Thyroid	1.70	1.19	2.20
Coeliac disease	Gastrointestinal	Thyroid	0.32	0.10	0.53
Crohn's disease	Gastrointestinal	Thyroid	0.08	0.00	0.19
Diverticular disease of intestine	Gastrointestinal	Thyroid	2.01	1.47	2.56
Fatty liver	Gastrointestinal	Thyroid	0.75	0.41	1.09
Gastritis and duodenitis	Gastrointestinal	Thyroid	2.49	1.88	3.09
Gastrooesophageal reflux disease	Gastrointestinal	Thyroid	3.59	2.87	4.32
Hepatic failure	Gastrointestinal	Thyroid	0.32	0.10	0.53
Liver fibrosis sclerosis and cirrhosis	Gastrointestinal	Thyroid	0.28	0.07	0.48
Oesophageal varices	Gastrointestinal	Thyroid	0.04	0.00	0.12
Oesophagitis and oesophageal ulcer	Gastrointestinal	Thyroid	3.71	2.98	4.45
Portal hypertension	Gastrointestinal	Thyroid	0.32	0.10	0.53
Ulcerative colitis	Gastrointestinal	Thyroid	0.12	0.00	0.25
Agranulocytosis	Haematological	Thyroid	0.12	0.00	0.25
Other anaemias	Haematological	Thyroid	2.29	1.71	2.87
Primary or idiopathic thrombocytopaenia	Haematological	Thyroid	0.20	0.02	0.37
Secondary or other thrombocytopaenia	Haematological	Thyroid	0.59	0.29	0.89
Secondary polycythaemia	Haematological	Thyroid	0.04	0.00	0.12
Thrombophilia	Haematological	Thyroid	0.24	0.05	0.43
Autoimmune liver disease	Immunology and infection	Thyroid	0.20	0.02	0.37
Chronic viral hepatitis	Immunology and infection	Thyroid	0.71	0.38	1.04
Encephalitis	Immunology and infection	Thyroid	0.04	0.00	0.12
HIV	Immunology and infection	Thyroid	0.47	0.21	0.74
Immunodeficiencies	Immunology and infection	Thyroid	0.12	0.00	0.25
Infection of anal and rectal regions	Immunology and infection	Thyroid	0.04	0.00	0.12
Infection of bones and joints	Immunology and infection	Thyroid	0.63	0.32	0.94
Infection of liver	Immunology and infection	Thyroid	0.16	0.00	0.31
Infection of skin and subcutaneous tissues	Immunology and infection	Thyroid	1.18	0.76	1.61
Infections of other or unspecified organs	Immunology and infection	Thyroid	2.49	1.88	3.09
Infections of the digestive system	Immunology and infection	Thyroid	1.42	0.96	1.88
Infections of the heart	Immunology and infection	Thyroid	0.24	0.05	0.43
Lower respiratory tract infections	Immunology and infection	Thyroid	3.20	2.51	3.88
Lupus erythematosus (local and systemic)	Immunology and infection	Thyroid	0.12	0.00	0.25
Meningitis	Immunology and infection	Thyroid	0.32	0.10	0.53
Non-acute cystitis	Immunology and infection	Thyroid	0.24	0.05	0.43
Peritonitis	Immunology and infection	Thyroid	3.20	2.51	3.88

Viral diseases (excluding chronic hepatitis/HIV)	Immunology and infection	Thyroid	0.67	0.35	0.99
Ankylosing spondylitis	Musculoskeletal	Thyroid	0.04	0.00	0.12
Enteropathic arthropathy	Musculoskeletal	Thyroid	0.08	0.00	0.19
Enthesopathies and synovial disorders	Musculoskeletal	Thyroid	5.37	4.49	6.25
Osteoarthritis (excluding spine)	Musculoskeletal	Thyroid	4.58	3.77	5.40
Osteoporosis	Musculoskeletal	Thyroid	2.01	1.47	2.56
Rheumatoid arthritis	Musculoskeletal	Thyroid	0.67	0.35	0.99
Scoliosis	Musculoskeletal	Thyroid	0.12	0.00	0.25
Spinal stenosis	Musculoskeletal	Thyroid	0.51	0.24	0.79
Bell's palsy	Neurological	Thyroid	0.28	0.07	0.48
Dementia	Neurological	Thyroid	0.24	0.05	0.43
Disorders of autonomic nervous system	Neurological	Thyroid	0.39	0.15	0.64
Epilepsy	Neurological	Thyroid	0.32	0.10	0.53
Essential tremor	Neurological	Thyroid	0.04	0.00	0.12
Intracerebral haemorrhage	Neurological	Thyroid	0.20	0.02	0.37
Intracranial hypertension	Neurological	Thyroid	0.32	0.10	0.53
Multiple sclerosis	Neurological	Thyroid	0.08	0.00	0.19
Peripheral neuropathies	Neurological	Thyroid	1.97	1.43	2.52
Subarachnoid haemorrhage	Neurological	Thyroid	0.12	0.00	0.25
Asthma	Pulmonary	Thyroid	2.92	2.27	3.58
Bronchiectasis	Pulmonary	Thyroid	0.24	0.05	0.43
COPD	Pulmonary	Thyroid	3.08	2.41	3.75
Other interstitial pulmonary diseases with fibrosis	Pulmonary	Thyroid	0.12	0.00	0.25
End stage renal disease	Renal	Thyroid	0.75	0.41	1.09
Glomerulonephritis	Renal	Thyroid	0.20	0.02	0.37
Obstructive and reflux uropathy	Renal	Thyroid	0.12	0.00	0.25
Urolithiasis	Renal	Thyroid	0.79	0.45	1.13
Abdominal aortic aneurysm	Cardiovascular	Uterus	0.16	0.08	0.25
Atrial fibrillation	Cardiovascular	Uterus	3.42	3.06	3.78
Atrioventricular block, complete	Cardiovascular	Uterus	0.56	0.41	0.70
Atrioventricular block, first degree	Cardiovascular	Uterus	0.56	0.41	0.70
Atrioventricular block, second degree	Cardiovascular	Uterus	0.56	0.41	0.70
Bifascicular block	Cardiovascular	Uterus	0.30	0.19	0.41
Coronary heart disease not otherwise specified	Cardiovascular	Uterus	6.38	5.89	6.87
Dilated cardiomyopathy	Cardiovascular	Uterus	0.05	0.01	0.10
Heart failure	Cardiovascular	Uterus	2.36	2.06	2.66
Hypertension	Cardiovascular	Uterus	13.48	12.80	14.16
Hypertrophic cardiomyopathy	Cardiovascular	Uterus	0.05	0.01	0.10
Ischaemic stroke	Cardiovascular	Uterus	0.77	0.60	0.95
Left bundle branch block	Cardiovascular	Uterus	0.62	0.46	0.77
Multiple valve disease	Cardiovascular	Uterus	0.36	0.24	0.48
Myocardial infarction	Cardiovascular	Uterus	2.54	2.22	2.85
Nonrheumatic aortic valve disorders	Cardiovascular	Uterus	0.56	0.41	0.70
Nonrheumatic mitral valve disorders	Cardiovascular	Uterus	0.43	0.30	0.56
Other cardiomyopathy	Cardiovascular	Uterus	2.63	2.31	2.95
Pericardial effusion (noninflammatory)	Cardiovascular	Uterus	0.08	0.03	0.14
Peripheral arterial disease	Cardiovascular	Uterus	0.94	0.75	1.13
Primary pulmonary hypertension	Cardiovascular	Uterus	0.18	0.09	0.26
Raynaud's syndrome	Cardiovascular	Uterus	0.49	0.36	0.63
Right bundle branch block	Cardiovascular	Uterus	0.30	0.19	0.41
Secondary pulmonary hypertension	Cardiovascular	Uterus	0.14	0.07	0.22
Sick sinus syndrome	Cardiovascular	Uterus	0.27	0.17	0.37
Stable angina	Cardiovascular	Uterus	2.44	2.14	2.75
Stroke (not otherwise specified)	Cardiovascular	Uterus	1.14	0.93	1.36
Supraventricular tachycardia	Cardiovascular	Uterus	0.46	0.33	0.60
Transient ischaemic attack	Cardiovascular	Uterus	1.85	1.59	2.12
Trifascicular block	Cardiovascular	Uterus	0.26	0.16	0.36
Unstable angina	Cardiovascular	Uterus	2.44	2.14	2.75
Venous thromboembolic disease	Cardiovascular	Uterus	1.84	1.58	2.11
Ventricular tachycardia	Cardiovascular	Uterus	0.48	0.35	0.62
Diabetes	Endocrine	Uterus	8.58	8.03	9.14

Diabetic neurological complications	Endocrine	Uterus	6.49	6.00	6.98
Diabetic ophthalmic complications	Endocrine	Uterus	7.20	6.69	7.72
Hyperparathyroidism	Endocrine	Uterus	0.19	0.10	0.27
Hypo or hyperthyroidism	Endocrine	Uterus	3.34	2.98	3.70
Alcoholic liver disease	Gastrointestinal	Uterus	0.29	0.18	0.40
Angiodysplasia of colon	Gastrointestinal	Uterus	0.12	0.05	0.19
Barrett's oesophagus	Gastrointestinal	Uterus	0.66	0.50	0.82
Cholangitis	Gastrointestinal	Uterus	1.72	1.46	1.98
Cholecystitis	Gastrointestinal	Uterus	1.90	1.62	2.17
Cholelithiasis	Gastrointestinal	Uterus	2.19	1.90	2.49
Coeliac disease	Gastrointestinal	Uterus	0.18	0.09	0.26
Crohn's disease	Gastrointestinal	Uterus	0.04	0.00	0.08
Diverticular disease of intestine	Gastrointestinal	Uterus	3.27	2.91	3.62
Fatty liver	Gastrointestinal	Uterus	0.78	0.61	0.96
Gastritis and duodenitis	Gastrointestinal	Uterus	2.23	1.93	2.52
Gastrooesophageal reflux disease	Gastrointestinal	Uterus	3.07	2.73	3.41
Hepatic failure	Gastrointestinal	Uterus	0.50	0.36	0.65
Liver fibrosis sclerosis and cirrhosis	Gastrointestinal	Uterus	0.12	0.05	0.19
Oesophageal varices	Gastrointestinal	Uterus	0.05	0.01	0.10
Oesophagitis and oesophageal ulcer	Gastrointestinal	Uterus	3.44	3.08	3.80
Portal hypertension	Gastrointestinal	Uterus	0.37	0.25	0.49
Ulcerative colitis	Gastrointestinal	Uterus	0.09	0.03	0.15
Agranulocytosis	Haematological	Uterus	0.10	0.04	0.17
Aplastic anaemias	Haematological	Uterus	0.02	0.00	0.05
Other anaemias	Haematological	Uterus	3.43	3.07	3.79
Other haemolytic anaemias	Haematological	Uterus	0.03	0.00	0.07
Primary or idiopathic thrombocytopaenia	Haematological	Uterus	0.12	0.05	0.19
Secondary or other thrombocytopaenia	Haematological	Uterus	0.24	0.14	0.33
Secondary polycythaemia	Haematological	Uterus	0.13	0.06	0.21
Thrombophilia	Haematological	Uterus	0.16	0.08	0.25
Autoimmune liver disease	Immunology and infection	Uterus	0.10	0.04	0.17
Chronic viral hepatitis	Immunology and infection	Uterus	0.30	0.19	0.41
Encephalitis	Immunology and infection	Uterus	0.12	0.05	0.19
HIV	Immunology and infection	Uterus	0.10	0.04	0.17
Immunodeficiencies	Immunology and infection	Uterus	0.01	0.00	0.03
Infection of anal and rectal regions	Immunology and infection	Uterus	0.13	0.06	0.21
Infection of bones and joints	Immunology and infection	Uterus	0.27	0.17	0.37
Infection of liver	Immunology and infection	Uterus	0.10	0.04	0.17
Infection of skin and subcutaneous tissues	Immunology and infection	Uterus	1.95	1.67	2.22
Infections of other or unspecified organs	Immunology and infection	Uterus	2.70	2.38	3.02
Infections of the digestive system	Immunology and infection	Uterus	1.29	1.06	1.51
Infections of the heart	Immunology and infection	Uterus	0.19	0.10	0.27
Lower respiratory tract infections	Immunology and infection	Uterus	3.00	2.66	3.34
Lupus erythematosus (local and systemic)	Immunology and infection	Uterus	0.05	0.01	0.10
Meningitis	Immunology and infection	Uterus	0.30	0.19	0.41
Non-acute cystitis	Immunology and infection	Uterus	0.34	0.22	0.46
Peritonitis	Immunology and infection	Uterus	3.78	3.40	4.16
Viral diseases (excluding chronic hepatitis/HIV)	Immunology and infection	Uterus	0.70	0.53	0.87
Ankylosing spondylitis	Musculoskeletal	Uterus	0.02	0.00	0.05
Enteropathic arthropathy	Musculoskeletal	Uterus	0.09	0.03	0.15
Enthesopathies and synovial disorders	Musculoskeletal	Uterus	5.12	4.68	5.56
Osteoarthritis (excluding spine)	Musculoskeletal	Uterus	8.17	7.63	8.72
Osteoporosis	Musculoskeletal	Uterus	2.25	1.95	2.54
Rheumatoid arthritis	Musculoskeletal	Uterus	0.49	0.36	0.63
Scoliosis	Musculoskeletal	Uterus	0.20	0.11	0.28
Spinal stenosis	Musculoskeletal	Uterus	0.35	0.23	0.47
Bell's palsy	Neurological	Uterus	0.15	0.08	0.23
Dementia	Neurological	Uterus	0.65	0.49	0.81
Disorders of autonomic nervous system	Neurological	Uterus	0.31	0.20	0.42
Epilepsy	Neurological	Uterus	0.30	0.19	0.41
Essential tremor	Neurological	Uterus	0.15	0.08	0.23
Intracerebral haemorrhage	Neurological	Uterus	0.34	0.22	0.46
Intracranial hypertension	Neurological	Uterus	0.08	0.03	0.14
Multiple sclerosis	Neurological	Uterus	0.02	0.00	0.05
Peripheral neuropathies	Neurological	Uterus	1.90	1.62	2.17
Subarachnoid haemorrhage	Neurological	Uterus	0.34	0.22	0.46
Asthma	Pulmonary	Uterus	2.88	2.54	3.21
Bronchiectasis	Pulmonary	Uterus	0.26	0.16	0.36
COPD	Pulmonary	Uterus	2.49	2.18	2.80
Other interstitial pulmonary diseases with fibrosis	Pulmonary	Uterus	0.15	0.08	0.23
End stage renal disease	Renal	Uterus	1.44	1.21	1.68
Glomerulonephritis	Renal	Uterus	0.48	0.35	0.62
Obstructive and reflux uropathy	Renal	Uterus	0.37	0.25	0.49
Urolithiasis	Renal	Uterus	0.77	0.60	0.95

Table S7. Multimorbidity patterns by cancer type. 95% confidence intervals (CIs) are shown.

Cancer type	Number of non-cancer conditions (%)	Proportion (%)	Lower CI	Upper CI
Bladder	0	10.71	10.18	11.24
Bladder	1	12.71	12.15	13.28
Bladder	2	10.70	10.17	11.22
Bladder	3	9.92	9.41	10.43
Bladder	4	8.60	8.12	9.08
Bladder	5	7.23	6.79	7.67
Bladder	≥ 6	40.12	39.29	40.96
Brain	0	14.28	13.25	15.31
Brain	1	14.89	13.84	15.94
Brain	2	14.32	13.29	15.36
Brain	3	11.03	10.10	11.95
Brain	4	9.74	8.86	10.61
Brain	5	7.39	6.62	8.16
Brain	≥ 6	28.36	27.03	29.68
Breast	0	18.23	17.93	18.52
Breast	1	18.24	17.94	18.53
Breast	2	14.64	14.37	14.91
Breast	3	11.59	11.35	11.84
Breast	4	8.67	8.45	8.88
Breast	5	6.82	6.63	7.01
Breast	≥ 6	21.81	21.50	22.13
Cervix	0	27.66	26.17	29.15
Cervix	1	20.82	19.47	22.17
Cervix	2	14.81	13.63	16.00
Cervix	3	9.82	8.83	10.81
Cervix	4	7.45	6.58	8.32
Cervix	5	4.56	3.87	5.26
Cervix	≥ 6	14.87	13.69	16.06
Colon and rectum	0	12.10	11.79	12.40
Colon and rectum	1	12.62	12.31	12.93
Colon and rectum	2	11.76	11.46	12.06
Colon and rectum	3	10.57	10.28	10.86
Colon and rectum	4	8.97	8.71	9.24
Colon and rectum	5	7.71	7.46	7.96
Colon and rectum	≥ 6	36.27	35.83	36.72
Gallbladder and biliary tract	0	4.46	3.31	5.60
Gallbladder and biliary tract	1	7.40	5.95	8.85
Gallbladder and biliary tract	2	8.59	7.04	10.14
Gallbladder and biliary tract	3	9.07	7.48	10.66
Gallbladder and biliary tract	4	8.43	6.90	9.97
Gallbladder and biliary tract	5	8.83	7.26	10.40
Gallbladder and biliary tract	≥ 6	53.22	50.46	55.98
Kidney and renal pelvis	0	9.66	8.98	10.33
Kidney and renal pelvis	1	12.13	11.38	12.88
Kidney and renal pelvis	2	11.78	11.04	12.52
Kidney and renal pelvis	3	10.40	9.70	11.10
Kidney and renal pelvis	4	9.15	8.48	9.81
Kidney and renal pelvis	5	7.65	7.04	8.26
Kidney and renal pelvis	≥ 6	39.24	38.12	40.36
Liver and intrahepatic bile duct	0	5.62	4.55	6.69
Liver and intrahepatic bile duct	1	6.41	5.27	7.55
Liver and intrahepatic bile duct	2	6.46	5.32	7.61
Liver and intrahepatic bile duct	3	5.79	4.70	6.88
Liver and intrahepatic bile duct	4	7.14	5.94	8.34
Liver and intrahepatic bile duct	5	5.51	4.45	6.57
Liver and intrahepatic bile duct	≥ 6	63.07	60.83	65.31
Lung and bronchus	0	10.15	9.82	10.49
Lung and bronchus	1	11.30	10.95	11.65
Lung and bronchus	2	10.54	10.20	10.88
Lung and bronchus	3	9.94	9.61	10.27
Lung and bronchus	4	8.82	8.51	9.14
Lung and bronchus	5	7.55	7.26	7.85
Lung and bronchus	≥ 6	41.69	41.14	42.24
Melanoma	0	20.96	20.34	21.59
Melanoma	1	17.90	17.31	18.49
Melanoma	2	14.02	13.48	14.55
Melanoma	3	10.69	10.21	11.17
Melanoma	4	7.77	7.35	8.18
Melanoma	5	5.88	5.52	6.25
Melanoma	≥ 6	22.78	22.13	23.43
Oesophagus	0	11.15	10.46	11.83
Oesophagus	1	11.94	11.24	12.64
Oesophagus	2	11.18	10.50	11.86
Oesophagus	3	10.09	9.44	10.75
Oesophagus	4	8.66	8.05	9.27
Oesophagus	5	7.75	7.17	8.33
Oesophagus	≥ 6	39.23	38.17	40.29
Oropharynx	0	17.93	17.07	18.79
Oropharynx	1	15.56	14.75	16.37
Oropharynx	2	12.23	11.49	12.96
Oropharynx	3	10.75	10.06	11.44
Oropharynx	4	8.37	7.75	8.99
Oropharynx	5	6.82	6.26	7.38
Oropharynx	≥ 6	28.34	27.34	29.35
Ovary	0	14.75	13.96	15.54
Ovary	1	15.35	14.55	16.15
Ovary	2	14.66	13.88	15.45
Ovary	3	10.88	10.19	11.57
Ovary	4	9.55	8.90	10.20
Ovary	5	7.70	7.11	8.29
Ovary	≥ 6	27.10	26.12	28.09
Pancreas	0	6.28	5.58	6.98
Pancreas	1	9.98	9.12	10.84
Pancreas	2	9.01	8.19	9.84
Pancreas	3	10.20	9.33	11.07
Pancreas	4	9.10	8.27	9.93
Pancreas	5	7.96	7.18	8.74
Pancreas	≥ 6	47.46	46.03	48.90
Prostate	0	11.99	11.67	12.30
Prostate	1	14.66	14.32	15.01
Prostate	2	13.77	13.43	14.10
Prostate	3	11.47	11.16	11.78
Prostate	4	9.21	8.93	9.49
Prostate	5	7.55	7.30	7.81
Prostate	≥ 6	31.35	30.90	31.79
Stomach	0	10.78	10.03	11.53
Stomach	1	12.37	11.57	13.17
Stomach	2	11.48	10.71	12.26
Stomach	3	10.39	9.65	11.12
Stomach	4	8.62	7.94	9.30
Stomach	5	7.61	6.97	8.25
Stomach	≥ 6	38.75	37.57	39.93
Testis	0	45.43	43.54	47.32
Testis	1	23.97	22.35	25.59
Testis	2	11.39	10.18	12.59
Testis	3	7.12	6.14	8.09
Testis	4	3.78	3.06	4.51
Testis	5	2.62	2.02	3.23
Testis	≥ 6	5.69	4.81	6.57
Thyroid	0	17.26	15.79	18.73
Thyroid	1	17.06	15.60	18.53
Thyroid	2	14.77	13.39	16.15
Thyroid	3	10.51	9.31	11.70
Thyroid	4	9.64	8.49	10.79
Thyroid	5	6.44	5.48	7.39
Thyroid	≥ 6	24.33	22.66	26.00
Uterus	0	10.93	10.31	11.55
Uterus	1	14.64	13.94	15.35
Uterus	2	14.63	13.93	15.34
Uterus	3	11.98	11.34	12.63
Uterus	4	9.65	9.06	10.23
Uterus	5	8.02	7.48	8.56
Uterus	≥ 6	30.14	29.23	31.06

Table S8. Multinomial logistic regression results for the associations between health conditions and cancer treatment decisions. Models were fitted using 'surgery alone' as the baseline choice of treatment. Odds ratios were adjusted for age, sex, socioeconomic status, tumour grade, tumour stage, tumour count and multimorbidity count. Strata with < 10 patients were not analysed.

Health condition	Organ system	Cancer type	Odds ratio	Lower CI	Upper CI	P value	Treatment type
Abdominal aortic aneurysm	Cardiovascular	Bladder	1.42	0.75	2.71	0.284	Chemotherapy & radiotherapy
Atrial fibrillation	Cardiovascular	Bladder	0.70	0.37	1.30	0.257	Chemotherapy & radiotherapy
Coronary heart disease not otherwise specified	Cardiovascular	Bladder	0.68	0.41	1.12	0.13	Chemotherapy & radiotherapy
Heart failure	Cardiovascular	Bladder	0.94	0.54	1.64	0.833	Chemotherapy & radiotherapy
Hypertension	Cardiovascular	Bladder	0.97	0.69	1.37	0.87	Chemotherapy & radiotherapy
Myocardial infarction	Cardiovascular	Bladder	0.98	0.58	1.66	0.946	Chemotherapy & radiotherapy
Other cardiomyopathy	Cardiovascular	Bladder	1.22	0.77	1.92	0.395	Chemotherapy & radiotherapy
Stable angina	Cardiovascular	Bladder	1.18	0.67	2.07	0.577	Chemotherapy & radiotherapy
Unstable angina	Cardiovascular	Bladder	1.24	0.73	2.11	0.419	Chemotherapy & radiotherapy
Diabetes	Endocrine	Bladder	1.15	0.74	1.79	0.52	Chemotherapy & radiotherapy
Diabetic neurological complications	Endocrine	Bladder	0.95	0.59	1.52	0.823	Chemotherapy & radiotherapy
Diabetic ophthalmic complications	Endocrine	Bladder	0.98	0.62	1.54	0.933	Chemotherapy & radiotherapy
Gastritis and duodenitis	Gastrointestinal	Bladder	1.40	0.74	2.64	0.298	Chemotherapy & radiotherapy
Oesophagitis and oesophageal ulcer	Gastrointestinal	Bladder	1.04	0.57	1.91	0.9	Chemotherapy & radiotherapy
Other anaemias	Haematological	Bladder	0.63	0.35	1.15	0.132	Chemotherapy & radiotherapy
Lower respiratory tract infections	Immunology and infection	Bladder	0.72	0.43	1.20	0.205	Chemotherapy & radiotherapy
Enthesopathies and synovial disorders	Musculoskeletal	Bladder	0.98	0.54	1.79	0.957	Chemotherapy & radiotherapy
Osteoarthritis (excluding spine)	Musculoskeletal	Bladder	0.82	0.49	1.38	0.456	Chemotherapy & radiotherapy
COPD	Pulmonary	Bladder	0.67	0.36	1.26	0.217	Chemotherapy & radiotherapy
End stage renal disease	Renal	Bladder	0.71	0.38	1.35	0.302	Chemotherapy & radiotherapy
Obstructive and reflux uropathy	Renal	Bladder	0.66	0.39	1.11	0.117	Chemotherapy & radiotherapy
Urolithiasis	Renal	Bladder	0.68	0.42	1.11	0.122	Chemotherapy & radiotherapy
Atrial fibrillation	Cardiovascular	Bladder	0.73	0.55	0.99	0.04	Chemotherapy & surgery
Heart failure	Cardiovascular	Bladder	0.71	0.52	0.98	0.038	Chemotherapy & surgery
Stroke (not otherwise specified)	Cardiovascular	Bladder	0.58	0.35	0.96	0.036	Chemotherapy & surgery
Raynaud's syndrome	Cardiovascular	Bladder	1.53	1.02	2.30	0.039	Chemotherapy & surgery
Abdominal aortic aneurysm	Cardiovascular	Bladder	1.18	0.81	1.71	0.387	Chemotherapy & surgery
Coronary heart disease not otherwise specified	Cardiovascular	Bladder	0.92	0.75	1.12	0.397	Chemotherapy & surgery
Hypertension	Cardiovascular	Bladder	1.05	0.90	1.24	0.521	Chemotherapy & surgery
Ischaemic stroke	Cardiovascular	Bladder	0.76	0.45	1.29	0.316	Chemotherapy & surgery
Myocardial infarction	Cardiovascular	Bladder	0.95	0.74	1.22	0.678	Chemotherapy & surgery
Nonrheumatic aortic valve disorders	Cardiovascular	Bladder	1.01	0.57	1.78	0.985	Chemotherapy & surgery
Nonrheumatic mitral valve disorders	Cardiovascular	Bladder	1.26	0.68	2.37	0.463	Chemotherapy & surgery
Other cardiomyopathy	Cardiovascular	Bladder	0.90	0.71	1.15	0.413	Chemotherapy & surgery
Peripheral arterial disease	Cardiovascular	Bladder	1.18	0.83	1.67	0.355	Chemotherapy & surgery
Sick sinus syndrome	Cardiovascular	Bladder	1.89	0.96	3.74	0.067	Chemotherapy & surgery
Stable angina	Cardiovascular	Bladder	0.76	0.56	1.04	0.081	Chemotherapy & surgery
Transient ischaemic attack	Cardiovascular	Bladder	0.97	0.68	1.39	0.89	Chemotherapy & surgery
Unstable angina	Cardiovascular	Bladder	0.81	0.61	1.08	0.155	Chemotherapy & surgery
Venous thromboembolic disease	Cardiovascular	Bladder	0.69	0.45	1.04	0.077	Chemotherapy & surgery
Diabetes	Endocrine	Bladder	0.97	0.77	1.22	0.799	Chemotherapy & surgery
Diabetic neurological complications	Endocrine	Bladder	0.80	0.62	1.03	0.081	Chemotherapy & surgery
Diabetic ophthalmic complications	Endocrine	Bladder	0.93	0.74	1.18	0.549	Chemotherapy & surgery
Hypo or hyperthyroidism	Endocrine	Bladder	1.07	0.70	1.64	0.761	Chemotherapy & surgery
Cholangitis	Gastrointestinal	Bladder	0.51	0.28	0.93	0.028	Chemotherapy & surgery
Gastrooesophageal reflux disease	Gastrointestinal	Bladder	1.44	1.06	1.95	0.02	Chemotherapy & surgery
Barrett's oesophagus	Gastrointestinal	Bladder	0.87	0.46	1.64	0.66	Chemotherapy & surgery
Cholecystitis	Gastrointestinal	Bladder	0.59	0.34	1.04	0.069	Chemotherapy & surgery
Cholelithiasis	Gastrointestinal	Bladder	0.62	0.38	1.04	0.07	Chemotherapy & surgery
Diverticular disease of intestine	Gastrointestinal	Bladder	1.04	0.75	1.44	0.816	Chemotherapy & surgery
Gastritis and duodenitis	Gastrointestinal	Bladder	1.09	0.78	1.52	0.612	Chemotherapy & surgery
Oesophagitis and oesophageal ulcer	Gastrointestinal	Bladder	1.22	0.92	1.62	0.163	Chemotherapy & surgery
Other anaemias	Haematological	Bladder	0.63	0.47	0.86	0.003	Chemotherapy & surgery
Infection of skin and subcutaneous tissues	Immunology and infection	Bladder	0.87	0.56	1.33	0.509	Chemotherapy & surgery
Infections of other or unspecified organs	Immunology and infection	Bladder	0.94	0.71	1.25	0.677	Chemotherapy & surgery
Infections of the digestive system	Immunology and infection	Bladder	0.64	0.39	1.04	0.07	Chemotherapy & surgery
Lower respiratory tract infections	Immunology and infection	Bladder	0.83	0.65	1.06	0.133	Chemotherapy & surgery
Non-acute cystitis	Immunology and infection	Bladder	0.99	0.71	1.37	0.944	Chemotherapy & surgery
Peritonitis	Immunology and infection	Bladder	0.91	0.68	1.21	0.516	Chemotherapy & surgery
Osteoporosis	Musculoskeletal	Bladder	0.58	0.37	0.92	0.021	Chemotherapy & surgery
Enthesopathies and synovial disorders	Musculoskeletal	Bladder	1.06	0.80	1.40	0.683	Chemotherapy & surgery
Osteoarthritis (excluding spine)	Musculoskeletal	Bladder	1.00	0.80	1.26	0.972	Chemotherapy & surgery
Rheumatoid arthritis	Musculoskeletal	Bladder	1.45	0.76	2.74	0.258	Chemotherapy & surgery
Peripheral neuropathies	Neurological	Bladder	1.33	0.89	1.99	0.158	Chemotherapy & surgery
Asthma	Pulmonary	Bladder	0.80	0.55	1.15	0.232	Chemotherapy & surgery
COPD	Pulmonary	Bladder	0.95	0.73	1.24	0.726	Chemotherapy & surgery
End stage renal disease	Renal	Bladder	0.62	0.42	0.89	0.011	Chemotherapy & surgery
Obstructive and reflux uropathy	Renal	Bladder	0.58	0.43	0.78	< 0.001	Chemotherapy & surgery
Urolithiasis	Renal	Bladder	0.74	0.58	0.94	0.015	Chemotherapy & surgery
Glomerulonephritis	Renal	Bladder	0.85	0.53	1.37	0.51	Chemotherapy & surgery
Hypertension	Cardiovascular	Bladder	0.77	0.61	0.97	0.028	Chemotherapy alone
Myocardial infarction	Cardiovascular	Bladder	0.63	0.43	0.92	0.017	Chemotherapy alone
Atrial fibrillation	Cardiovascular	Bladder	1.37	1.01	1.85	0.042	Chemotherapy alone
Transient ischaemic attack	Cardiovascular	Bladder	1.47	1.01	2.14	0.044	Chemotherapy alone
Abdominal aortic aneurysm	Cardiovascular	Bladder	1.19	0.77	1.85	0.427	Chemotherapy alone
Coronary heart disease not otherwise specified	Cardiovascular	Bladder	0.87	0.65	1.15	0.324	Chemotherapy alone
Heart failure	Cardiovascular	Bladder	1.06	0.75	1.49	0.754	Chemotherapy alone
Ischaemic stroke	Cardiovascular	Bladder	1.26	0.73	2.16	0.405	Chemotherapy alone
Other cardiomyopathy	Cardiovascular	Bladder	0.75	0.55	1.04	0.09	Chemotherapy alone
Peripheral arterial disease	Cardiovascular	Bladder	0.85	0.50	1.43	0.532	Chemotherapy alone
Raynaud's syndrome	Cardiovascular	Bladder	1.15	0.66	2.00	0.63	Chemotherapy alone

Stable angina	Cardiovascular	Bladder	1.05	0.73	1.51	0.802	Chemotherapy alone
Stroke (not otherwise specified)	Cardiovascular	Bladder	1.10	0.66	1.85	0.711	Chemotherapy alone
Unstable angina	Cardiovascular	Bladder	0.84	0.58	1.22	0.357	Chemotherapy alone
Venous thromboembolic disease	Cardiovascular	Bladder	0.90	0.57	1.44	0.663	Chemotherapy alone
Diabetes	Endocrine	Bladder	0.92	0.68	1.24	0.569	Chemotherapy alone
Diabetic neurological complications	Endocrine	Bladder	0.86	0.64	1.18	0.352	Chemotherapy alone
Diabetic ophthalmic complications	Endocrine	Bladder	0.73	0.53	1.00	0.051	Chemotherapy alone
Hypo or hyperthyroidism	Endocrine	Bladder	0.82	0.44	1.54	0.536	Chemotherapy alone
Cholangitis	Gastrointestinal	Bladder	0.93	0.51	1.68	0.806	Chemotherapy alone
Cholecystitis	Gastrointestinal	Bladder	1.01	0.57	1.79	0.982	Chemotherapy alone
Cholelithiasis	Gastrointestinal	Bladder	0.86	0.49	1.51	0.59	Chemotherapy alone
Diverticular disease of intestine	Gastrointestinal	Bladder	1.02	0.67	1.53	0.943	Chemotherapy alone
Gastritis and duodenitis	Gastrointestinal	Bladder	1.20	0.79	1.83	0.384	Chemotherapy alone
Gastrooesophageal reflux disease	Gastrointestinal	Bladder	1.14	0.75	1.72	0.536	Chemotherapy alone
Oesophagitis and oesophageal ulcer	Gastrointestinal	Bladder	0.76	0.49	1.16	0.199	Chemotherapy alone
Other anaemias	Haematological	Bladder	0.64	0.43	0.93	0.021	Chemotherapy alone
Infection of skin and subcutaneous tissues	Immunology and infection	Bladder	0.70	0.39	1.25	0.222	Chemotherapy alone
Infections of other or unspecified organs	Immunology and infection	Bladder	0.68	0.45	1.02	0.06	Chemotherapy alone
Infections of the digestive system	Immunology and infection	Bladder	0.86	0.52	1.44	0.569	Chemotherapy alone
Lower respiratory tract infections	Immunology and infection	Bladder	0.97	0.72	1.31	0.854	Chemotherapy alone
Non-acute cystitis	Immunology and infection	Bladder	0.65	0.38	1.10	0.106	Chemotherapy alone
Peritonitis	Immunology and infection	Bladder	0.99	0.70	1.39	0.936	Chemotherapy alone
Enthesopathies and synovial disorders	Musculoskeletal	Bladder	0.93	0.63	1.37	0.723	Chemotherapy alone
Osteoarthritis (excluding spine)	Musculoskeletal	Bladder	0.83	0.60	1.13	0.235	Chemotherapy alone
Osteoporosis	Musculoskeletal	Bladder	0.61	0.33	1.13	0.118	Chemotherapy alone
Asthma	Pulmonary	Bladder	1.35	0.90	2.01	0.147	Chemotherapy alone
COPD	Pulmonary	Bladder	1.04	0.75	1.46	0.797	Chemotherapy alone
End stage renal disease	Renal	Bladder	0.60	0.39	0.94	0.027	Chemotherapy alone
Glomerulonephritis	Renal	Bladder	0.75	0.40	1.41	0.371	Chemotherapy alone
Obstructive and reflux uropathy	Renal	Bladder	0.71	0.49	1.01	0.057	Chemotherapy alone
Urolithiasis	Renal	Bladder	0.75	0.54	1.02	0.07	Chemotherapy alone
Abdominal aortic aneurysm	Cardiovascular	Bladder	1.10	0.59	2.02	0.771	Chemotherapy, radiotherapy & surgery
Atrial fibrillation	Cardiovascular	Bladder	0.88	0.56	1.38	0.574	Chemotherapy, radiotherapy & surgery
Coronary heart disease not otherwise specified	Cardiovascular	Bladder	0.95	0.69	1.32	0.755	Chemotherapy, radiotherapy & surgery
Heart failure	Cardiovascular	Bladder	0.78	0.47	1.29	0.333	Chemotherapy, radiotherapy & surgery
Hypertension	Cardiovascular	Bladder	0.98	0.75	1.27	0.868	Chemotherapy, radiotherapy & surgery
Myocardial infarction	Cardiovascular	Bladder	1.24	0.86	1.81	0.251	Chemotherapy, radiotherapy & surgery
Other cardiomyopathy	Cardiovascular	Bladder	1.18	0.83	1.68	0.365	Chemotherapy, radiotherapy & surgery
Stable angina	Cardiovascular	Bladder	1.03	0.66	1.60	0.911	Chemotherapy, radiotherapy & surgery
Transient ischaemic attack	Cardiovascular	Bladder	1.03	0.59	1.82	0.915	Chemotherapy, radiotherapy & surgery
Unstable angina	Cardiovascular	Bladder	0.83	0.52	1.33	0.449	Chemotherapy, radiotherapy & surgery
Venous thromboembolic disease	Cardiovascular	Bladder	0.80	0.43	1.49	0.478	Chemotherapy, radiotherapy & surgery
Diabetes	Endocrine	Bladder	0.74	0.49	1.11	0.143	Chemotherapy, radiotherapy & surgery
Diabetic neurological complications	Endocrine	Bladder	0.84	0.57	1.24	0.384	Chemotherapy, radiotherapy & surgery
Diabetic ophthalmic complications	Endocrine	Bladder	0.91	0.63	1.31	0.602	Chemotherapy, radiotherapy & surgery
Diverticular disease of intestine	Gastrointestinal	Bladder	1.33	0.82	2.14	0.249	Chemotherapy, radiotherapy & surgery
Gastritis and duodenitis	Gastrointestinal	Bladder	1.26	0.75	2.11	0.38	Chemotherapy, radiotherapy & surgery
Gastrooesophageal reflux disease	Gastrointestinal	Bladder	0.87	0.48	1.59	0.659	Chemotherapy, radiotherapy & surgery
Oesophagitis and oesophageal ulcer	Gastrointestinal	Bladder	1.00	0.62	1.62	0.999	Chemotherapy, radiotherapy & surgery
Other anaemias	Haematological	Bladder	0.72	0.46	1.15	0.171	Chemotherapy, radiotherapy & surgery
Infections of other or unspecified organs	Immunology and infection	Bladder	0.98	0.63	1.50	0.913	Chemotherapy, radiotherapy & surgery
Infections of the digestive system	Immunology and infection	Bladder	0.98	0.53	1.81	0.941	Chemotherapy, radiotherapy & surgery
Lower respiratory tract infections	Immunology and infection	Bladder	0.81	0.54	1.20	0.286	Chemotherapy, radiotherapy & surgery
Non-acute cystitis	Immunology and infection	Bladder	0.87	0.52	1.44	0.585	Chemotherapy, radiotherapy & surgery
Peritonitis	Immunology and infection	Bladder	1.07	0.69	1.65	0.763	Chemotherapy, radiotherapy & surgery
Enthesopathies and synovial disorders	Musculoskeletal	Bladder	1.16	0.76	1.78	0.492	Chemotherapy, radiotherapy & surgery
Osteoarthritis (excluding spine)	Musculoskeletal	Bladder	0.78	0.52	1.18	0.24	Chemotherapy, radiotherapy & surgery
Peripheral neuropathies	Neurological	Bladder	1.37	0.73	2.58	0.321	Chemotherapy, radiotherapy & surgery
Asthma	Pulmonary	Bladder	0.81	0.45	1.48	0.496	Chemotherapy, radiotherapy & surgery
COPD	Pulmonary	Bladder	0.96	0.63	1.47	0.868	Chemotherapy, radiotherapy & surgery
End stage renal disease	Renal	Bladder	0.61	0.33	1.11	0.105	Chemotherapy, radiotherapy & surgery
Obstructive and reflux uropathy	Renal	Bladder	0.83	0.56	1.22	0.339	Chemotherapy, radiotherapy & surgery
Urolithiasis	Renal	Bladder	0.82	0.58	1.17	0.276	Chemotherapy, radiotherapy & surgery
Sick sinus syndrome	Cardiovascular	Bladder	1.83	1.03	3.23	0.038	Radiotherapy & surgery
Ventricular tachycardia	Cardiovascular	Bladder	1.35	0.84	2.16	0.211	Radiotherapy & surgery
Abdominal aortic aneurysm	Cardiovascular	Bladder	0.88	0.61	1.26	0.49	Radiotherapy & surgery
Atrial fibrillation	Cardiovascular	Bladder	1.02	0.83	1.25	0.861	Radiotherapy & surgery
Atrioventricular block, complete	Cardiovascular	Bladder	0.83	0.53	1.29	0.398	Radiotherapy & surgery
Atrioventricular block, first degree	Cardiovascular	Bladder	0.83	0.53	1.29	0.398	Radiotherapy & surgery
Atrioventricular block, second degree	Cardiovascular	Bladder	0.83	0.53	1.29	0.398	Radiotherapy & surgery
Bifascicular block	Cardiovascular	Bladder	1.16	0.68	1.98	0.591	Radiotherapy & surgery
Coronary heart disease not otherwise specified	Cardiovascular	Bladder	0.92	0.77	1.10	0.365	Radiotherapy & surgery
Heart failure	Cardiovascular	Bladder	0.81	0.64	1.03	0.082	Radiotherapy & surgery
Hypertension	Cardiovascular	Bladder	0.99	0.86	1.14	0.911	Radiotherapy & surgery
Ischaemic stroke	Cardiovascular	Bladder	0.70	0.46	1.07	0.1	Radiotherapy & surgery
Left bundle branch block	Cardiovascular	Bladder	0.78	0.50	1.22	0.273	Radiotherapy & surgery
Multiple valve disease	Cardiovascular	Bladder	0.88	0.48	1.62	0.675	Radiotherapy & surgery
Myocardial infarction	Cardiovascular	Bladder	1.00	0.81	1.23	0.981	Radiotherapy & surgery
Nonrheumatic aortic valve disorders	Cardiovascular	Bladder	0.75	0.46	1.24	0.264	Radiotherapy & surgery
Nonrheumatic mitral valve disorders	Cardiovascular	Bladder	0.92	0.52	1.62	0.771	Radiotherapy & surgery
Other cardiomyopathy	Cardiovascular	Bladder	1.07	0.89	1.30	0.473	Radiotherapy & surgery
Peripheral arterial disease	Cardiovascular	Bladder	0.71	0.50	1.01	0.055	Radiotherapy & surgery
Raynaud's syndrome	Cardiovascular	Bladder	1.10	0.76	1.60	0.608	Radiotherapy & surgery
Right bundle branch block	Cardiovascular	Bladder	1.16	0.68	1.98	0.591	Radiotherapy & surgery
Stable angina	Cardiovascular	Bladder	1.11	0.88	1.39	0.37	Radiotherapy & surgery
Stroke (not otherwise specified)	Cardiovascular	Bladder	0.92	0.67	1.27	0.626	Radiotherapy & surgery
Supraventricular tachycardia	Cardiovascular	Bladder	0.98	0.54	1.77	0.94	Radiotherapy & surgery
Transient ischaemic attack	Cardiovascular	Bladder	0.88	0.65	1.19	0.414	Radiotherapy & surgery

Trifascicular block	Cardiovascular	Bladder	1.10	0.61	1.96	0.758	Radiotherapy & surgery
Unstable angina	Cardiovascular	Bladder	1.16	0.94	1.44	0.165	Radiotherapy & surgery
Venous thromboembolic disease	Cardiovascular	Bladder	0.79	0.58	1.07	0.126	Radiotherapy & surgery
Hypo or hyperthyroidism	Endocrine	Bladder	1.39	1.01	1.91	0.045	Radiotherapy & surgery
Diabetes	Endocrine	Bladder	0.91	0.74	1.12	0.37	Radiotherapy & surgery
Diabetic neurological complications	Endocrine	Bladder	0.90	0.73	1.10	0.311	Radiotherapy & surgery
Diabetic ophthalmic complications	Endocrine	Bladder	0.85	0.69	1.04	0.105	Radiotherapy & surgery
Barrett's oesophagus	Gastrointestinal	Bladder	1.26	0.80	1.98	0.325	Radiotherapy & surgery
Cholangitis	Gastrointestinal	Bladder	0.73	0.49	1.10	0.136	Radiotherapy & surgery
Cholecystitis	Gastrointestinal	Bladder	0.79	0.53	1.18	0.254	Radiotherapy & surgery
Cholelithiasis	Gastrointestinal	Bladder	0.76	0.52	1.11	0.16	Radiotherapy & surgery
Diverticular disease of intestine	Gastrointestinal	Bladder	1.01	0.77	1.31	0.952	Radiotherapy & surgery
Fatty liver	Gastrointestinal	Bladder	1.28	0.76	2.18	0.354	Radiotherapy & surgery
Gastritis and duodenitis	Gastrointestinal	Bladder	1.12	0.84	1.49	0.456	Radiotherapy & surgery
Gastrooesophageal reflux disease	Gastrointestinal	Bladder	1.11	0.82	1.48	0.506	Radiotherapy & surgery
Hepatic failure	Gastrointestinal	Bladder	1.39	0.78	2.48	0.27	Radiotherapy & surgery
Oesophagitis and oesophageal ulcer	Gastrointestinal	Bladder	1.15	0.90	1.48	0.268	Radiotherapy & surgery
Other anaemias	Haematological	Bladder	0.85	0.68	1.05	0.127	Radiotherapy & surgery
Secondary or other thrombocytopaenia	Haematological	Bladder	1.61	0.88	2.94	0.12	Radiotherapy & surgery
Infection of skin and subcutaneous tissues	Immunology and infection	Bladder	0.92	0.65	1.30	0.638	Radiotherapy & surgery
Infections of other or unspecified organs	Immunology and infection	Bladder	0.86	0.68	1.09	0.22	Radiotherapy & surgery
Infections of the digestive system	Immunology and infection	Bladder	0.86	0.60	1.22	0.389	Radiotherapy & surgery
Lower respiratory tract infections	Immunology and infection	Bladder	1.03	0.85	1.23	0.788	Radiotherapy & surgery
Non-acute cystitis	Immunology and infection	Bladder	0.98	0.73	1.31	0.884	Radiotherapy & surgery
Peritonitis	Immunology and infection	Bladder	1.06	0.85	1.33	0.595	Radiotherapy & surgery
Enthesopathies and synovial disorders	Musculoskeletal	Bladder	1.13	0.88	1.45	0.348	Radiotherapy & surgery
Osteoarthritis (excluding spine)	Musculoskeletal	Bladder	1.04	0.86	1.26	0.673	Radiotherapy & surgery
Osteoporosis	Musculoskeletal	Bladder	1.07	0.80	1.45	0.642	Radiotherapy & surgery
Rheumatoid arthritis	Musculoskeletal	Bladder	0.96	0.51	1.82	0.903	Radiotherapy & surgery
Spinal stenosis	Musculoskeletal	Bladder	1.37	0.76	2.48	0.29	Radiotherapy & surgery
Dementia	Neurological	Bladder	0.57	0.38	0.85	0.006	Radiotherapy & surgery
Peripheral neuropathies	Neurological	Bladder	1.54	1.11	2.14	0.011	Radiotherapy & surgery
Epilepsy	Neurological	Bladder	1.32	0.68	2.59	0.412	Radiotherapy & surgery
Intracerebral haemorrhage	Neurological	Bladder	0.98	0.57	1.67	0.933	Radiotherapy & surgery
Subarachnoid haemorrhage	Neurological	Bladder	0.96	0.53	1.71	0.878	Radiotherapy & surgery
COPD	Pulmonary	Bladder	1.23	1.00	1.51	0.048	Radiotherapy & surgery
Asthma	Pulmonary	Bladder	1.09	0.81	1.46	0.565	Radiotherapy & surgery
End stage renal disease	Renal	Bladder	0.87	0.67	1.13	0.303	Radiotherapy & surgery
Glomerulonephritis	Renal	Bladder	0.96	0.66	1.40	0.845	Radiotherapy & surgery
Obstructive and reflux uropathy	Renal	Bladder	0.92	0.75	1.14	0.457	Radiotherapy & surgery
Urolithiasis	Renal	Bladder	0.89	0.74	1.09	0.259	Radiotherapy & surgery
Other cardiomyopathy	Cardiovascular	Bladder	0.65	0.45	0.94	0.021	Radiotherapy alone
Unstable angina	Cardiovascular	Bladder	0.61	0.40	0.95	0.029	Radiotherapy alone
Atrial fibrillation	Cardiovascular	Bladder	1.40	1.04	1.90	0.029	Radiotherapy alone
Transient ischaemic attack	Cardiovascular	Bladder	1.49	1.00	2.20	0.048	Radiotherapy alone
Abdominal aortic aneurysm	Cardiovascular	Bladder	0.69	0.36	1.29	0.245	Radiotherapy alone
Coronary heart disease not otherwise specified	Cardiovascular	Bladder	0.72	0.52	1.01	0.055	Radiotherapy alone
Heart failure	Cardiovascular	Bladder	0.94	0.67	1.33	0.745	Radiotherapy alone
Hypertension	Cardiovascular	Bladder	0.99	0.78	1.25	0.921	Radiotherapy alone
Ischaemic stroke	Cardiovascular	Bladder	1.25	0.73	2.16	0.415	Radiotherapy alone
Myocardial infarction	Cardiovascular	Bladder	0.70	0.47	1.02	0.064	Radiotherapy alone
Nonrheumatic aortic valve disorders	Cardiovascular	Bladder	1.33	0.73	2.43	0.352	Radiotherapy alone
Peripheral arterial disease	Cardiovascular	Bladder	1.23	0.77	1.96	0.386	Radiotherapy alone
Raynaud's syndrome	Cardiovascular	Bladder	1.53	0.91	2.55	0.106	Radiotherapy alone
Stable angina	Cardiovascular	Bladder	0.64	0.40	1.03	0.067	Radiotherapy alone
Stroke (not otherwise specified)	Cardiovascular	Bladder	0.91	0.54	1.53	0.72	Radiotherapy alone
Venous thromboembolic disease	Cardiovascular	Bladder	1.03	0.67	1.59	0.899	Radiotherapy alone
Diabetes	Endocrine	Bladder	0.91	0.65	1.27	0.572	Radiotherapy alone
Diabetic neurological complications	Endocrine	Bladder	0.79	0.56	1.11	0.175	Radiotherapy alone
Diabetic ophthalmic complications	Endocrine	Bladder	0.81	0.59	1.12	0.204	Radiotherapy alone
Hypo or hyperthyroidism	Endocrine	Bladder	1.27	0.75	2.15	0.378	Radiotherapy alone
Cholangitis	Gastrointestinal	Bladder	1.04	0.60	1.80	0.897	Radiotherapy alone
Cholecystitis	Gastrointestinal	Bladder	0.86	0.47	1.56	0.616	Radiotherapy alone
Cholelithiasis	Gastrointestinal	Bladder	0.99	0.58	1.71	0.974	Radiotherapy alone
Diverticular disease of intestine	Gastrointestinal	Bladder	0.85	0.55	1.33	0.477	Radiotherapy alone
Gastritis and duodenitis	Gastrointestinal	Bladder	0.85	0.49	1.46	0.554	Radiotherapy alone
Gastrooesophageal reflux disease	Gastrointestinal	Bladder	1.48	0.97	2.25	0.07	Radiotherapy alone
Oesophagitis and oesophageal ulcer	Gastrointestinal	Bladder	1.10	0.73	1.65	0.659	Radiotherapy alone
Other anaemias	Haematological	Bladder	0.96	0.69	1.31	0.777	Radiotherapy alone
Infection of skin and subcutaneous tissues	Immunology and infection	Bladder	1.17	0.72	1.92	0.528	Radiotherapy alone
Infections of other or unspecified organs	Immunology and infection	Bladder	0.86	0.60	1.23	0.397	Radiotherapy alone
Lower respiratory tract infections	Immunology and infection	Bladder	1.00	0.75	1.34	0.991	Radiotherapy alone
Non-acute cystitis	Immunology and infection	Bladder	0.97	0.60	1.57	0.886	Radiotherapy alone
Peritonitis	Immunology and infection	Bladder	0.92	0.64	1.33	0.659	Radiotherapy alone
Enthesopathies and synovial disorders	Musculoskeletal	Bladder	1.26	0.84	1.89	0.261	Radiotherapy alone
Osteoarthritis (excluding spine)	Musculoskeletal	Bladder	1.01	0.74	1.38	0.965	Radiotherapy alone
Osteoporosis	Musculoskeletal	Bladder	0.97	0.58	1.61	0.903	Radiotherapy alone
Dementia	Neurological	Bladder	0.60	0.33	1.08	0.089	Radiotherapy alone
Peripheral neuropathies	Neurological	Bladder	0.86	0.45	1.63	0.645	Radiotherapy alone
Asthma	Pulmonary	Bladder	0.86	0.49	1.50	0.583	Radiotherapy alone
COPD	Pulmonary	Bladder	1.05	0.74	1.50	0.766	Radiotherapy alone
End stage renal disease	Renal	Bladder	0.60	0.39	0.93	0.022	Radiotherapy alone
Obstructive and reflux uropathy	Renal	Bladder	1.28	0.96	1.71	0.096	Radiotherapy alone
Urolithiasis	Renal	Bladder	1.25	0.95	1.65	0.113	Radiotherapy alone
Coronary heart disease not otherwise specified	Cardiovascular	Brain	1.07	0.67	1.72	0.771	Chemotherapy & radiotherapy
Hypertension	Cardiovascular	Brain	0.74	0.52	1.06	0.102	Chemotherapy & radiotherapy
Ischaemic stroke	Cardiovascular	Brain	1.13	0.62	2.07	0.69	Chemotherapy & radiotherapy

Myocardial infarction	Cardiovascular	Brain	1.04	0.52	2.08	0.911	Chemotherapy & radiotherapy
Other cardiomyopathy	Cardiovascular	Brain	1.16	0.62	2.18	0.643	Chemotherapy & radiotherapy
Stroke (not otherwise specified)	Cardiovascular	Brain	0.72	0.41	1.26	0.248	Chemotherapy & radiotherapy
Transient ischaemic attack	Cardiovascular	Brain	1.53	0.94	2.49	0.085	Chemotherapy & radiotherapy
Unstable angina	Cardiovascular	Brain	1.84	0.94	3.61	0.077	Chemotherapy & radiotherapy
Venous thromboembolic disease	Cardiovascular	Brain	1.09	0.66	1.78	0.737	Chemotherapy & radiotherapy
Diabetes	Endocrine	Brain	0.88	0.54	1.43	0.604	Chemotherapy & radiotherapy
Diabetic neurological complications	Endocrine	Brain	0.71	0.40	1.28	0.254	Chemotherapy & radiotherapy
Diabetic ophthalmic complications	Endocrine	Brain	0.74	0.42	1.30	0.296	Chemotherapy & radiotherapy
Diverticular disease of intestine	Gastrointestinal	Brain	1.48	0.76	2.85	0.248	Chemotherapy & radiotherapy
Gastrooesophageal reflux disease	Gastrointestinal	Brain	1.14	0.60	2.17	0.683	Chemotherapy & radiotherapy
Oesophagitis and oesophageal ulcer	Gastrointestinal	Brain	1.21	0.68	2.15	0.513	Chemotherapy & radiotherapy
Infections of other or unspecified organs	Immunology and infection	Brain	0.49	0.26	0.92	0.026	Chemotherapy & radiotherapy
Lower respiratory tract infections	Immunology and infection	Brain	0.69	0.38	1.26	0.228	Chemotherapy & radiotherapy
Peritonitis	Immunology and infection	Brain	1.55	0.89	2.70	0.118	Chemotherapy & radiotherapy
Enthesopathies and synovial disorders	Musculoskeletal	Brain	0.72	0.42	1.22	0.222	Chemotherapy & radiotherapy
Osteoarthritis (excluding spine)	Musculoskeletal	Brain	1.19	0.71	1.99	0.518	Chemotherapy & radiotherapy
Epilepsy	Neurological	Brain	0.92	0.62	1.37	0.682	Chemotherapy & radiotherapy
Intracranial hypertension	Neurological	Brain	0.99	0.71	1.39	0.952	Chemotherapy & radiotherapy
Asthma	Pulmonary	Brain	1.14	0.60	2.16	0.681	Chemotherapy & radiotherapy
Hypertension	Cardiovascular	Brain	0.66	0.39	1.12	0.128	Chemotherapy & surgery
Infections of other or unspecified organs	Immunology and infection	Brain	0.84	0.42	1.69	0.633	Chemotherapy & surgery
Epilepsy	Neurological	Brain	1.04	0.63	1.74	0.865	Chemotherapy & surgery
Intracranial hypertension	Neurological	Brain	1.37	0.90	2.09	0.141	Chemotherapy & surgery
Coronary heart disease not otherwise specified	Cardiovascular	Brain	1.14	0.63	2.04	0.671	Chemotherapy alone
Hypertension	Cardiovascular	Brain	1.34	0.91	1.99	0.141	Chemotherapy alone
Stroke (not otherwise specified)	Cardiovascular	Brain	0.74	0.38	1.44	0.379	Chemotherapy alone
Diabetes	Endocrine	Brain	0.82	0.44	1.52	0.528	Chemotherapy alone
Diverticular disease of intestine	Gastrointestinal	Brain	2.11	1.04	4.24	0.037	Chemotherapy alone
Peritonitis	Immunology and infection	Brain	1.77	0.95	3.31	0.072	Chemotherapy alone
Epilepsy	Neurological	Brain	1.06	0.66	1.71	0.802	Chemotherapy alone
Intracranial hypertension	Neurological	Brain	1.31	0.89	1.94	0.177	Chemotherapy alone
Hypertension	Cardiovascular	Brain	0.66	0.45	0.97	0.034	Chemotherapy, radiotherapy & surgery
Coronary heart disease not otherwise specified	Cardiovascular	Brain	0.61	0.34	1.10	0.099	Chemotherapy, radiotherapy & surgery
Ischaemic stroke	Cardiovascular	Brain	0.83	0.41	1.66	0.592	Chemotherapy, radiotherapy & surgery
Stroke (not otherwise specified)	Cardiovascular	Brain	0.78	0.43	1.41	0.411	Chemotherapy, radiotherapy & surgery
Transient ischaemic attack	Cardiovascular	Brain	0.87	0.48	1.58	0.643	Chemotherapy, radiotherapy & surgery
Venous thromboembolic disease	Cardiovascular	Brain	0.68	0.38	1.21	0.191	Chemotherapy, radiotherapy & surgery
Diabetes	Endocrine	Brain	0.93	0.57	1.52	0.772	Chemotherapy, radiotherapy & surgery
Diabetic neurological complications	Endocrine	Brain	1.09	0.65	1.85	0.735	Chemotherapy, radiotherapy & surgery
Diabetic ophthalmic complications	Endocrine	Brain	0.99	0.59	1.68	0.974	Chemotherapy, radiotherapy & surgery
Hypo or hyperthyroidism	Endocrine	Brain	1.57	0.79	3.10	0.197	Chemotherapy, radiotherapy & surgery
Cholecystitis	Gastrointestinal	Brain	1.49	0.69	3.24	0.308	Chemotherapy, radiotherapy & surgery
Cholelithiasis	Gastrointestinal	Brain	1.74	0.80	3.81	0.165	Chemotherapy, radiotherapy & surgery
Diverticular disease of intestine	Gastrointestinal	Brain	1.60	0.84	3.08	0.156	Chemotherapy, radiotherapy & surgery
Gastrooesophageal reflux disease	Gastrointestinal	Brain	1.02	0.52	1.98	0.964	Chemotherapy, radiotherapy & surgery
Oesophagitis and oesophageal ulcer	Gastrointestinal	Brain	0.92	0.49	1.73	0.798	Chemotherapy, radiotherapy & surgery
Lower respiratory tract infections	Immunology and infection	Brain	0.54	0.28	1.05	0.068	Chemotherapy, radiotherapy & surgery
Peritonitis	Immunology and infection	Brain	0.96	0.51	1.81	0.898	Chemotherapy, radiotherapy & surgery
Enthesopathies and synovial disorders	Musculoskeletal	Brain	0.97	0.60	1.57	0.897	Chemotherapy, radiotherapy & surgery
Osteoarthritis (excluding spine)	Musculoskeletal	Brain	1.38	0.83	2.31	0.212	Chemotherapy, radiotherapy & surgery
Epilepsy	Neurological	Brain	1.50	1.05	2.14	0.027	Chemotherapy, radiotherapy & surgery
Peripheral neuropathies	Neurological	Brain	3.15	1.36	7.30	0.007	Chemotherapy, radiotherapy & surgery
Intracranial hypertension	Neurological	Brain	0.79	0.56	1.13	0.199	Chemotherapy, radiotherapy & surgery
Atrial fibrillation	Cardiovascular	Brain	0.94	0.56	1.57	0.812	Radiotherapy & surgery
Coronary heart disease not otherwise specified	Cardiovascular	Brain	1.36	0.95	1.93	0.09	Radiotherapy & surgery
Hypertension	Cardiovascular	Brain	0.99	0.77	1.29	0.965	Radiotherapy & surgery
Ischaemic stroke	Cardiovascular	Brain	0.66	0.38	1.14	0.132	Radiotherapy & surgery
Myocardial infarction	Cardiovascular	Brain	1.09	0.64	1.84	0.757	Radiotherapy & surgery
Other cardiomyopathy	Cardiovascular	Brain	1.20	0.74	1.96	0.463	Radiotherapy & surgery
Raynaud's syndrome	Cardiovascular	Brain	1.76	0.72	4.31	0.212	Radiotherapy & surgery
Stable angina	Cardiovascular	Brain	1.20	0.67	2.14	0.545	Radiotherapy & surgery
Stroke (not otherwise specified)	Cardiovascular	Brain	0.80	0.53	1.20	0.283	Radiotherapy & surgery
Transient ischaemic attack	Cardiovascular	Brain	1.11	0.73	1.67	0.632	Radiotherapy & surgery
Unstable angina	Cardiovascular	Brain	1.41	0.80	2.48	0.233	Radiotherapy & surgery
Venous thromboembolic disease	Cardiovascular	Brain	0.65	0.42	1.01	0.054	Radiotherapy & surgery
Diabetes	Endocrine	Brain	0.81	0.55	1.20	0.293	Radiotherapy & surgery
Diabetic neurological complications	Endocrine	Brain	1.15	0.77	1.72	0.49	Radiotherapy & surgery
Diabetic ophthalmic complications	Endocrine	Brain	1.21	0.82	1.79	0.339	Radiotherapy & surgery
Hypo or hyperthyroidism	Endocrine	Brain	1.09	0.61	1.95	0.765	Radiotherapy & surgery
Cholangitis	Gastrointestinal	Brain	1.00	0.47	2.12	0.996	Radiotherapy & surgery
Cholecystitis	Gastrointestinal	Brain	0.77	0.37	1.60	0.483	Radiotherapy & surgery
Cholelithiasis	Gastrointestinal	Brain	0.86	0.41	1.82	0.699	Radiotherapy & surgery
Diverticular disease of intestine	Gastrointestinal	Brain	1.41	0.84	2.39	0.197	Radiotherapy & surgery
Fatty liver	Gastrointestinal	Brain	1.95	0.92	4.14	0.081	Radiotherapy & surgery
Gastritis and duodenitis	Gastrointestinal	Brain	0.75	0.43	1.29	0.297	Radiotherapy & surgery
Gastrooesophageal reflux disease	Gastrointestinal	Brain	1.27	0.78	2.08	0.336	Radiotherapy & surgery
Hepatic failure	Gastrointestinal	Brain	2.02	0.88	4.67	0.099	Radiotherapy & surgery
Oesophagitis and oesophageal ulcer	Gastrointestinal	Brain	1.25	0.80	1.96	0.329	Radiotherapy & surgery

Other anaemias	Haematological	Brain	0.99	0.45	2.17	0.98	Radiotherapy & surgery
Infections of other or unspecified organs	Immunology and infection	Brain	0.54	0.34	0.86	0.009	Radiotherapy & surgery
Infection of skin and subcutaneous tissues	Immunology and infection	Brain	0.79	0.38	1.65	0.53	Radiotherapy & surgery
Infections of the digestive system	Immunology and infection	Brain	0.76	0.37	1.54	0.442	Radiotherapy & surgery
Lower respiratory tract infections	Immunology and infection	Brain	0.73	0.47	1.15	0.176	Radiotherapy & surgery
Peritonitis	Immunology and infection	Brain	1.16	0.73	1.84	0.54	Radiotherapy & surgery
Enthesopathies and synovial disorders	Musculoskeletal	Brain	1.02	0.70	1.48	0.906	Radiotherapy & surgery
Osteoarthritis (excluding spine)	Musculoskeletal	Brain	0.94	0.61	1.43	0.771	Radiotherapy & surgery
Osteoporosis	Musculoskeletal	Brain	1.22	0.63	2.36	0.552	Radiotherapy & surgery
Bell's palsy	Neurological	Brain	2.13	0.93	4.88	0.074	Radiotherapy & surgery
Epilepsy	Neurological	Brain	1.32	0.98	1.76	0.065	Radiotherapy & surgery
Intracerebral haemorrhage	Neurological	Brain	0.73	0.43	1.23	0.238	Radiotherapy & surgery
Intracranial hypertension	Neurological	Brain	0.93	0.71	1.22	0.604	Radiotherapy & surgery
Peripheral neuropathies	Neurological	Brain	1.34	0.58	3.09	0.498	Radiotherapy & surgery
Asthma	Pulmonary	Brain	1.31	0.80	2.15	0.289	Radiotherapy & surgery
COPD	Pulmonary	Brain	0.96	0.55	1.66	0.882	Radiotherapy & surgery
End stage renal disease	Renal	Brain	0.90	0.43	1.89	0.777	Radiotherapy & surgery
Urolithiasis	Renal	Brain	1.19	0.50	2.82	0.694	Radiotherapy & surgery
Atrial fibrillation	Cardiovascular	Brain	0.65	0.39	1.07	0.093	Radiotherapy alone
Coronary heart disease not otherwise specified	Cardiovascular	Brain	1.13	0.81	1.59	0.471	Radiotherapy alone
Heart failure	Cardiovascular	Brain	1.18	0.57	2.44	0.656	Radiotherapy alone
Hypertension	Cardiovascular	Brain	0.92	0.72	1.18	0.516	Radiotherapy alone
Ischaemic stroke	Cardiovascular	Brain	0.95	0.61	1.49	0.832	Radiotherapy alone
Myocardial infarction	Cardiovascular	Brain	1.08	0.68	1.73	0.731	Radiotherapy alone
Other cardiomyopathy	Cardiovascular	Brain	1.08	0.69	1.68	0.732	Radiotherapy alone
Peripheral arterial disease	Cardiovascular	Brain	1.26	0.53	3.03	0.599	Radiotherapy alone
Stable angina	Cardiovascular	Brain	1.37	0.82	2.26	0.226	Radiotherapy alone
Stroke (not otherwise specified)	Cardiovascular	Brain	0.77	0.53	1.10	0.149	Radiotherapy alone
Transient ischaemic attack	Cardiovascular	Brain	0.82	0.56	1.21	0.322	Radiotherapy alone
Unstable angina	Cardiovascular	Brain	1.57	0.96	2.58	0.075	Radiotherapy alone
Venous thromboembolic disease	Cardiovascular	Brain	0.83	0.56	1.21	0.33	Radiotherapy alone
Diabetes	Endocrine	Brain	0.96	0.68	1.36	0.814	Radiotherapy alone
Diabetic neurological complications	Endocrine	Brain	0.89	0.61	1.31	0.559	Radiotherapy alone
Diabetic ophthalmic complications	Endocrine	Brain	0.87	0.59	1.27	0.458	Radiotherapy alone
Hypo or hyperthyroidism	Endocrine	Brain	1.29	0.77	2.16	0.333	Radiotherapy alone
Barrett's oesophagus	Gastrointestinal	Brain	0.94	0.44	2.04	0.885	Radiotherapy alone
Cholangitis	Gastrointestinal	Brain	0.83	0.40	1.72	0.622	Radiotherapy alone
Cholecystitis	Gastrointestinal	Brain	0.67	0.33	1.34	0.258	Radiotherapy alone
Cholelithiasis	Gastrointestinal	Brain	0.74	0.36	1.51	0.412	Radiotherapy alone
Diverticular disease of intestine	Gastrointestinal	Brain	1.27	0.77	2.09	0.343	Radiotherapy alone
Fatty liver	Gastrointestinal	Brain	1.34	0.62	2.89	0.461	Radiotherapy alone
Gastritis and duodenitis	Gastrointestinal	Brain	0.88	0.54	1.42	0.601	Radiotherapy alone
Gastrooesophageal reflux disease	Gastrointestinal	Brain	1.39	0.89	2.18	0.148	Radiotherapy alone
Hepatic failure	Gastrointestinal	Brain	1.72	0.75	3.92	0.2	Radiotherapy alone
Oesophagitis and oesophageal ulcer	Gastrointestinal	Brain	1.31	0.87	1.98	0.192	Radiotherapy alone
Other anaemias	Haematological	Brain	1.06	0.52	2.15	0.868	Radiotherapy alone
Viral diseases (excluding chronic hepatitis/HIV)	Immunology and infection	Brain	0.75	0.49	1.15	0.189	Radiotherapy alone
Infection of skin and subcutaneous tissues	Immunology and infection	Brain	0.66	0.31	1.37	0.262	Radiotherapy alone
Infections of other or unspecified organs	Immunology and infection	Brain	0.68	0.45	1.03	0.07	Radiotherapy alone
Infections of the digestive system	Immunology and infection	Brain	0.69	0.36	1.33	0.269	Radiotherapy alone
Lower respiratory tract infections	Immunology and infection	Brain	0.75	0.51	1.12	0.163	Radiotherapy alone
Peritonitis	Immunology and infection	Brain	1.19	0.77	1.81	0.433	Radiotherapy alone
Enthesopathies and synovial disorders	Musculoskeletal	Brain	1.06	0.75	1.51	0.731	Radiotherapy alone
Osteoarthritis (excluding spine)	Musculoskeletal	Brain	1.18	0.82	1.69	0.375	Radiotherapy alone
Osteoporosis	Musculoskeletal	Brain	0.54	0.26	1.14	0.106	Radiotherapy alone
Peripheral neuropathies	Neurological	Brain	2.51	1.24	5.09	0.011	Radiotherapy alone
Bell's palsy	Neurological	Brain	1.92	0.86	4.30	0.111	Radiotherapy alone
Epilepsy	Neurological	Brain	1.09	0.81	1.47	0.566	Radiotherapy alone
Intracerebral haemorrhage	Neurological	Brain	0.74	0.46	1.20	0.228	Radiotherapy alone
Intracranial hypertension	Neurological	Brain	0.82	0.63	1.07	0.146	Radiotherapy alone
Subarachnoid haemorrhage	Neurological	Brain	0.95	0.43	2.11	0.903	Radiotherapy alone
Asthma	Pulmonary	Brain	0.88	0.53	1.49	0.645	Radiotherapy alone
COPD	Pulmonary	Brain	0.87	0.52	1.44	0.577	Radiotherapy alone
End stage renal disease	Renal	Brain	0.63	0.30	1.35	0.236	Radiotherapy alone
Atrial fibrillation	Cardiovascular	Breast	0.63	0.47	0.85	0.002	Chemotherapy & radiotherapy
Coronary heart disease not otherwise specified	Cardiovascular	Breast	0.75	0.63	0.88	< 0.001	Chemotherapy & radiotherapy
Heart failure	Cardiovascular	Breast	0.47	0.32	0.68	< 0.001	Chemotherapy & radiotherapy
Hypertension	Cardiovascular	Breast	0.76	0.67	0.86	< 0.001	Chemotherapy & radiotherapy
Peripheral arterial disease	Cardiovascular	Breast	0.44	0.25	0.77	0.004	Chemotherapy & radiotherapy
Stroke (not otherwise specified)	Cardiovascular	Breast	0.55	0.35	0.88	0.013	Chemotherapy & radiotherapy
Venous thromboembolic disease	Cardiovascular	Breast	0.64	0.44	0.92	0.015	Chemotherapy & radiotherapy
Abdominal aortic aneurysm	Cardiovascular	Breast	3.13	1.59	6.17	0.001	Chemotherapy & radiotherapy
Atrioventricular block, complete	Cardiovascular	Breast	0.67	0.35	1.29	0.236	Chemotherapy & radiotherapy
Atrioventricular block, first degree	Cardiovascular	Breast	0.67	0.35	1.29	0.236	Chemotherapy & radiotherapy
Atrioventricular block, second degree	Cardiovascular	Breast	0.67	0.35	1.29	0.236	Chemotherapy & radiotherapy
Left bundle branch block	Cardiovascular	Breast	0.75	0.41	1.36	0.344	Chemotherapy & radiotherapy
Myocardial infarction	Cardiovascular	Breast	0.82	0.62	1.08	0.16	Chemotherapy & radiotherapy
Nonrheumatic mitral valve disorders	Cardiovascular	Breast	0.91	0.52	1.61	0.745	Chemotherapy & radiotherapy
Other cardiomyopathy	Cardiovascular	Breast	0.86	0.65	1.13	0.274	Chemotherapy & radiotherapy
Raynaud's syndrome	Cardiovascular	Breast	0.64	0.38	1.06	0.082	Chemotherapy & radiotherapy
Stable angina	Cardiovascular	Breast	0.76	0.57	1.02	0.069	Chemotherapy & radiotherapy

Supraventricular tachycardia	Cardiovascular	Breast	1.05	0.60	1.84	0.872	Chemotherapy & radiotherapy
Transient ischaemic attack	Cardiovascular	Breast	0.84	0.60	1.18	0.31	Chemotherapy & radiotherapy
Unstable angina	Cardiovascular	Breast	0.93	0.69	1.24	0.617	Chemotherapy & radiotherapy
Ventricular tachycardia	Cardiovascular	Breast	1.00	0.56	1.79	0.987	Chemotherapy & radiotherapy
Diabetes	Endocrine	Breast	0.84	0.71	0.99	0.038	Chemotherapy & radiotherapy
Hypo or hyperthyroidism	Endocrine	Breast	0.77	0.61	0.98	0.031	Chemotherapy & radiotherapy
Diabetic neurological complications	Endocrine	Breast	0.89	0.69	1.14	0.345	Chemotherapy & radiotherapy
Diabetic ophthalmic complications	Endocrine	Breast	0.93	0.75	1.15	0.511	Chemotherapy & radiotherapy
Barrett's oesophagus	Gastrointestinal	Breast	0.50	0.30	0.84	0.009	Chemotherapy & radiotherapy
Diverticular disease of intestine	Gastrointestinal	Breast	1.34	1.09	1.66	0.006	Chemotherapy & radiotherapy
Cholangitis	Gastrointestinal	Breast	1.14	0.86	1.51	0.347	Chemotherapy & radiotherapy
Cholecystitis	Gastrointestinal	Breast	1.03	0.78	1.36	0.83	Chemotherapy & radiotherapy
Cholelithiasis	Gastrointestinal	Breast	0.97	0.74	1.26	0.808	Chemotherapy & radiotherapy
Coeliac disease	Gastrointestinal	Breast	1.05	0.59	1.89	0.858	Chemotherapy & radiotherapy
Fatty liver	Gastrointestinal	Breast	1.00	0.58	1.72	0.997	Chemotherapy & radiotherapy
Gastritis and duodenitis	Gastrointestinal	Breast	0.95	0.75	1.19	0.645	Chemotherapy & radiotherapy
Gastrooesophageal reflux disease	Gastrointestinal	Breast	1.01	0.82	1.24	0.934	Chemotherapy & radiotherapy
Oesophagitis and oesophageal ulcer	Gastrointestinal	Breast	0.83	0.68	1.02	0.078	Chemotherapy & radiotherapy
Other anaemias	Haematological	Breast	0.95	0.74	1.22	0.706	Chemotherapy & radiotherapy
Infections of the digestive system	Immunology and infection	Breast	1.56	1.17	2.07	0.002	Chemotherapy & radiotherapy
Peritonitis	Immunology and infection	Breast	1.43	1.18	1.74	< 0.001	Chemotherapy & radiotherapy
Chronic viral hepatitis	Immunology and infection	Breast	1.14	0.68	1.90	0.626	Chemotherapy & radiotherapy
HIV	Immunology and infection	Breast	0.88	0.40	1.95	0.76	Chemotherapy & radiotherapy
Infection of bones and joints	Immunology and infection	Breast	0.89	0.50	1.58	0.682	Chemotherapy & radiotherapy
Infection of skin and subcutaneous tissues	Immunology and infection	Breast	1.00	0.73	1.36	0.983	Chemotherapy & radiotherapy
Infections of other or unspecified organs	Immunology and infection	Breast	0.86	0.68	1.10	0.226	Chemotherapy & radiotherapy
Infections of the heart	Immunology and infection	Breast	0.86	0.44	1.69	0.655	Chemotherapy & radiotherapy
Lower respiratory tract infections	Immunology and infection	Breast	0.83	0.65	1.05	0.115	Chemotherapy & radiotherapy
Meningitis	Immunology and infection	Breast	1.15	0.63	2.10	0.642	Chemotherapy & radiotherapy
Non-acute cystitis	Immunology and infection	Breast	1.20	0.63	2.29	0.572	Chemotherapy & radiotherapy
Viral diseases (excluding chronic hepatitis/HIV)	Immunology and infection	Breast	0.76	0.48	1.21	0.254	Chemotherapy & radiotherapy
Enthesopathies and synovial disorders	Musculoskeletal	Breast	1.04	0.89	1.21	0.65	Chemotherapy & radiotherapy
Osteoarthritis (excluding spine)	Musculoskeletal	Breast	0.90	0.78	1.05	0.175	Chemotherapy & radiotherapy
Osteoporosis	Musculoskeletal	Breast	1.11	0.87	1.40	0.404	Chemotherapy & radiotherapy
Rheumatoid arthritis	Musculoskeletal	Breast	0.85	0.54	1.34	0.494	Chemotherapy & radiotherapy
Scoliosis	Musculoskeletal	Breast	0.81	0.40	1.61	0.539	Chemotherapy & radiotherapy
Spinal stenosis	Musculoskeletal	Breast	0.95	0.55	1.67	0.869	Chemotherapy & radiotherapy
Dementia	Neurological	Breast	0.59	0.34	1.03	0.062	Chemotherapy & radiotherapy
Epilepsy	Neurological	Breast	1.10	0.59	2.07	0.768	Chemotherapy & radiotherapy
Peripheral neuropathies	Neurological	Breast	1.03	0.79	1.34	0.85	Chemotherapy & radiotherapy
Asthma	Pulmonary	Breast	0.70	0.55	0.88	0.002	Chemotherapy & radiotherapy
COPD	Pulmonary	Breast	0.80	0.62	1.03	0.082	Chemotherapy & radiotherapy
End stage renal disease	Renal	Breast	1.20	0.85	1.70	0.3	Chemotherapy & radiotherapy
Glomerulonephritis	Renal	Breast	1.62	0.87	3.03	0.131	Chemotherapy & radiotherapy
Urolithiasis	Renal	Breast	1.17	0.75	1.82	0.482	Chemotherapy & radiotherapy
Hypertension	Cardiovascular	Breast	0.82	0.75	0.90	< 0.001	Chemotherapy & surgery
Ischaemic stroke	Cardiovascular	Breast	0.64	0.43	0.94	0.023	Chemotherapy & surgery
Nonrheumatic aortic valve disorders	Cardiovascular	Breast	0.53	0.34	0.84	0.007	Chemotherapy & surgery
Stroke (not otherwise specified)	Cardiovascular	Breast	0.69	0.52	0.92	0.013	Chemotherapy & surgery
Bifascicular block	Cardiovascular	Breast	1.94	1.14	3.31	0.014	Chemotherapy & surgery
Right bundle branch block	Cardiovascular	Breast	2.05	1.20	3.50	0.008	Chemotherapy & surgery
Trifascicular block	Cardiovascular	Breast	1.86	1.01	3.43	0.045	Chemotherapy & surgery
Abdominal aortic aneurysm	Cardiovascular	Breast	1.07	0.53	2.18	0.841	Chemotherapy & surgery
Atrial fibrillation	Cardiovascular	Breast	0.96	0.81	1.15	0.691	Chemotherapy & surgery
Atrioventricular block, complete	Cardiovascular	Breast	1.02	0.68	1.55	0.907	Chemotherapy & surgery
Atrioventricular block, first degree	Cardiovascular	Breast	1.02	0.68	1.55	0.907	Chemotherapy & surgery
Atrioventricular block, second degree	Cardiovascular	Breast	1.02	0.68	1.55	0.907	Chemotherapy & surgery
Coronary heart disease not otherwise specified	Cardiovascular	Breast	0.90	0.80	1.02	0.091	Chemotherapy & surgery
Heart failure	Cardiovascular	Breast	0.95	0.77	1.17	0.605	Chemotherapy & surgery
Left bundle branch block	Cardiovascular	Breast	0.94	0.63	1.40	0.771	Chemotherapy & surgery
Multiple valve disease	Cardiovascular	Breast	0.87	0.55	1.36	0.534	Chemotherapy & surgery
Myocardial infarction	Cardiovascular	Breast	0.93	0.76	1.13	0.443	Chemotherapy & surgery
Nonrheumatic mitral valve disorders	Cardiovascular	Breast	0.73	0.47	1.14	0.161	Chemotherapy & surgery
Other cardiomyopathy	Cardiovascular	Breast	0.92	0.76	1.12	0.431	Chemotherapy & surgery
Peripheral arterial disease	Cardiovascular	Breast	0.75	0.54	1.05	0.09	Chemotherapy & surgery
Raynaud's syndrome	Cardiovascular	Breast	0.70	0.47	1.04	0.078	Chemotherapy & surgery
Sick sinus syndrome	Cardiovascular	Breast	1.09	0.64	1.84	0.753	Chemotherapy & surgery
Stable angina	Cardiovascular	Breast	0.82	0.67	1.01	0.056	Chemotherapy & surgery
Supraventricular tachycardia	Cardiovascular	Breast	1.28	0.85	1.95	0.239	Chemotherapy & surgery
Transient ischaemic attack	Cardiovascular	Breast	0.87	0.68	1.11	0.266	Chemotherapy & surgery
Unstable angina	Cardiovascular	Breast	1.00	0.81	1.23	0.996	Chemotherapy & surgery
Venous thromboembolic disease	Cardiovascular	Breast	0.83	0.66	1.06	0.134	Chemotherapy & surgery
Ventricular tachycardia	Cardiovascular	Breast	1.30	0.86	1.98	0.219	Chemotherapy & surgery
Diabetes	Endocrine	Breast	0.95	0.83	1.08	0.405	Chemotherapy & surgery
Diabetic neurological complications	Endocrine	Breast	1.08	0.90	1.29	0.407	Chemotherapy & surgery
Diabetic ophthalmic complications	Endocrine	Breast	1.05	0.90	1.23	0.536	Chemotherapy & surgery
Hyperparathyroidism	Endocrine	Breast	1.45	0.75	2.81	0.268	Chemotherapy & surgery
Hypo or hyperthyroidism	Endocrine	Breast	1.13	0.95	1.33	0.157	Chemotherapy & surgery
Barrett's oesophagus	Gastrointestinal	Breast	0.63	0.43	0.93	0.021	Chemotherapy & surgery

Gastrooesophageal reflux disease	Gastrointestinal	Breast	0.84	0.70	1.00	0.048	Chemotherapy & surgery
Oesophagitis and oesophageal ulcer	Gastrointestinal	Breast	0.72	0.61	0.86	< 0.001	Chemotherapy & surgery
Crohn's disease	Gastrointestinal	Breast	2.78	1.35	5.71	0.006	Chemotherapy & surgery
Hepatic failure	Gastrointestinal	Breast	1.61	1.02	2.52	0.039	Chemotherapy & surgery
Ulcerative colitis	Gastrointestinal	Breast	2.60	1.35	5.00	0.004	Chemotherapy & surgery
Cholangitis	Gastrointestinal	Breast	0.95	0.74	1.21	0.665	Chemotherapy & surgery
Cholecystitis	Gastrointestinal	Breast	0.92	0.72	1.16	0.477	Chemotherapy & surgery
Cholelithiasis	Gastrointestinal	Breast	0.92	0.74	1.15	0.472	Chemotherapy & surgery
Coeliac disease	Gastrointestinal	Breast	1.00	0.55	1.80	0.996	Chemotherapy & surgery
Diverticular disease of intestine	Gastrointestinal	Breast	1.02	0.85	1.21	0.851	Chemotherapy & surgery
Fatty liver	Gastrointestinal	Breast	1.34	0.87	2.07	0.181	Chemotherapy & surgery
Gastritis and duodenitis	Gastrointestinal	Breast	0.86	0.71	1.04	0.109	Chemotherapy & surgery
Liver fibrosis sclerosis and cirrhosis	Gastrointestinal	Breast	1.33	0.65	2.76	0.436	Chemotherapy & surgery
Portal hypertension	Gastrointestinal	Breast	1.50	0.87	2.59	0.143	Chemotherapy & surgery
Agranulocytosis	Haematological	Breast	1.35	0.64	2.86	0.434	Chemotherapy & surgery
Other anaemias	Haematological	Breast	1.07	0.89	1.30	0.455	Chemotherapy & surgery
Infection of bones and joints	Immunology and infection	Breast	0.49	0.25	0.94	0.033	Chemotherapy & surgery
Infection of skin and subcutaneous tissues	Immunology and infection	Breast	0.75	0.58	0.97	0.031	Chemotherapy & surgery
Chronic viral hepatitis	Immunology and infection	Breast	1.20	0.76	1.90	0.432	Chemotherapy & surgery
Infections of other or unspecified organs	Immunology and infection	Breast	0.89	0.73	1.08	0.224	Chemotherapy & surgery
Infections of the heart	Immunology and infection	Breast	0.70	0.39	1.26	0.237	Chemotherapy & surgery
Infections of the digestive system	Immunology and infection	Breast	0.90	0.67	1.20	0.469	Chemotherapy & surgery
Lower respiratory tract infections	Immunology and infection	Breast	1.00	0.84	1.19	0.991	Chemotherapy & surgery
Meningitis	Immunology and infection	Breast	0.63	0.34	1.14	0.128	Chemotherapy & surgery
Non-acute cystitis	Immunology and infection	Breast	0.97	0.56	1.67	0.911	Chemotherapy & surgery
Peritonitis	Immunology and infection	Breast	1.07	0.91	1.27	0.403	Chemotherapy & surgery
Viral diseases (excluding chronic hepatitis/HIV)	Immunology and infection	Breast	0.86	0.59	1.25	0.424	Chemotherapy & surgery
Osteoporosis	Musculoskeletal	Breast	0.72	0.59	0.88	0.001	Chemotherapy & surgery
Enteropathic arthropathy	Musculoskeletal	Breast	2.35	1.13	4.91	0.023	Chemotherapy & surgery
Enthesopathies and synovial disorders	Musculoskeletal	Breast	0.97	0.85	1.10	0.659	Chemotherapy & surgery
Osteoarthritis (excluding spine)	Musculoskeletal	Breast	0.90	0.80	1.01	0.072	Chemotherapy & surgery
Rheumatoid arthritis	Musculoskeletal	Breast	0.92	0.64	1.32	0.651	Chemotherapy & surgery
Scoliosis	Musculoskeletal	Breast	0.63	0.36	1.09	0.095	Chemotherapy & surgery
Spinal stenosis	Musculoskeletal	Breast	1.13	0.74	1.71	0.571	Chemotherapy & surgery
Dementia	Neurological	Breast	1.01	0.76	1.34	0.951	Chemotherapy & surgery
Epilepsy	Neurological	Breast	0.61	0.34	1.09	0.094	Chemotherapy & surgery
Intracerebral haemorrhage	Neurological	Breast	0.56	0.31	1.01	0.053	Chemotherapy & surgery
Peripheral neuropathies	Neurological	Breast	0.82	0.65	1.04	0.105	Chemotherapy & surgery
Subarachnoid haemorrhage	Neurological	Breast	0.73	0.43	1.24	0.249	Chemotherapy & surgery
COPD	Pulmonary	Breast	0.77	0.64	0.94	0.01	Chemotherapy & surgery
Asthma	Pulmonary	Breast	0.94	0.80	1.11	0.478	Chemotherapy & surgery
Bronchiectasis	Pulmonary	Breast	0.52	0.26	1.04	0.063	Chemotherapy & surgery
End stage renal disease	Renal	Breast	1.13	0.85	1.49	0.413	Chemotherapy & surgery
Glomerulonephritis	Renal	Breast	0.62	0.31	1.25	0.181	Chemotherapy & surgery
Urolithiasis	Renal	Breast	1.06	0.72	1.55	0.766	Chemotherapy & surgery
Coronary heart disease not otherwise specified	Cardiovascular	Breast	0.81	0.70	0.94	0.004	Chemotherapy alone
Hypertension	Cardiovascular	Breast	0.69	0.61	0.77	< 0.001	Chemotherapy alone
Raynaud's syndrome	Cardiovascular	Breast	0.58	0.37	0.91	0.017	Chemotherapy alone
Stable angina	Cardiovascular	Breast	0.69	0.54	0.88	0.003	Chemotherapy alone
Heart failure	Cardiovascular	Breast	1.34	1.10	1.63	0.003	Chemotherapy alone
Trifascicular block	Cardiovascular	Breast	1.95	1.07	3.55	0.028	Chemotherapy alone
Abdominal aortic aneurysm	Cardiovascular	Breast	1.72	0.87	3.40	0.12	Chemotherapy alone
Atrial fibrillation	Cardiovascular	Breast	1.01	0.84	1.22	0.886	Chemotherapy alone
Atrioventricular block, complete	Cardiovascular	Breast	1.12	0.74	1.70	0.589	Chemotherapy alone
Atrioventricular block, first degree	Cardiovascular	Breast	1.12	0.74	1.70	0.589	Chemotherapy alone
Atrioventricular block, second degree	Cardiovascular	Breast	1.12	0.74	1.70	0.589	Chemotherapy alone
Bifascicular block	Cardiovascular	Breast	1.52	0.85	2.70	0.155	Chemotherapy alone
Ischaemic stroke	Cardiovascular	Breast	1.06	0.75	1.50	0.747	Chemotherapy alone
Left bundle branch block	Cardiovascular	Breast	1.07	0.71	1.60	0.749	Chemotherapy alone
Multiple valve disease	Cardiovascular	Breast	1.02	0.65	1.59	0.941	Chemotherapy alone
Myocardial infarction	Cardiovascular	Breast	0.86	0.69	1.07	0.173	Chemotherapy alone
Nonrheumatic aortic valve disorders	Cardiovascular	Breast	1.00	0.68	1.48	0.996	Chemotherapy alone
Nonrheumatic mitral valve disorders	Cardiovascular	Breast	1.11	0.73	1.68	0.632	Chemotherapy alone
Other cardiomyopathy	Cardiovascular	Breast	0.84	0.67	1.04	0.107	Chemotherapy alone
Peripheral arterial disease	Cardiovascular	Breast	0.70	0.48	1.01	0.058	Chemotherapy alone
Right bundle branch block	Cardiovascular	Breast	1.59	0.89	2.83	0.119	Chemotherapy alone
Sick sinus syndrome	Cardiovascular	Breast	1.12	0.64	1.97	0.699	Chemotherapy alone
Stroke (not otherwise specified)	Cardiovascular	Breast	1.25	0.96	1.62	0.094	Chemotherapy alone
Supraventricular tachycardia	Cardiovascular	Breast	0.53	0.28	1.00	0.05	Chemotherapy alone
Transient ischaemic attack	Cardiovascular	Breast	1.11	0.87	1.41	0.406	Chemotherapy alone
Unstable angina	Cardiovascular	Breast	0.81	0.63	1.03	0.079	Chemotherapy alone
Venous thromboembolic disease	Cardiovascular	Breast	1.18	0.94	1.49	0.154	Chemotherapy alone
Ventricular tachycardia	Cardiovascular	Breast	0.55	0.29	1.03	0.062	Chemotherapy alone
Diabetic neurological complications	Endocrine	Breast	0.79	0.64	0.97	0.028	Chemotherapy alone
Diabetic ophthalmic complications	Endocrine	Breast	0.81	0.67	0.98	0.027	Chemotherapy alone
Hypo or hyperthyroidism	Endocrine	Breast	0.81	0.66	0.99	0.046	Chemotherapy alone
Diabetes	Endocrine	Breast	0.89	0.77	1.03	0.13	Chemotherapy alone
Hyperparathyroidism	Endocrine	Breast	1.63	0.86	3.09	0.135	Chemotherapy alone
Gastritis and duodenitis	Gastrointestinal	Breast	0.76	0.61	0.94	0.012	Chemotherapy alone
Barrett's oesophagus	Gastrointestinal	Breast	0.87	0.61	1.24	0.443	Chemotherapy alone
Cholangitis	Gastrointestinal	Breast	0.75	0.56	1.01	0.058	Chemotherapy alone

Cholecystitis	Gastrointestinal	Breast	0.83	0.64	1.09	0.187	Chemotherapy alone
Cholelithiasis	Gastrointestinal	Breast	0.79	0.61	1.02	0.066	Chemotherapy alone
Coeliac disease	Gastrointestinal	Breast	1.10	0.62	1.95	0.746	Chemotherapy alone
Diverticular disease of intestine	Gastrointestinal	Breast	0.88	0.72	1.07	0.193	Chemotherapy alone
Fatty liver	Gastrointestinal	Breast	0.79	0.47	1.34	0.389	Chemotherapy alone
Gastrooesophageal reflux disease	Gastrointestinal	Breast	0.86	0.71	1.04	0.124	Chemotherapy alone
Hepatic failure	Gastrointestinal	Breast	1.16	0.70	1.94	0.558	Chemotherapy alone
Oesophagitis and oesophageal ulcer	Gastrointestinal	Breast	0.91	0.76	1.08	0.274	Chemotherapy alone
Portal hypertension	Gastrointestinal	Breast	0.78	0.40	1.50	0.448	Chemotherapy alone
Other anaemias	Haematological	Breast	1.19	0.97	1.45	0.096	Chemotherapy alone
Infections of the digestive system	Immunology and infection	Breast	1.36	1.05	1.76	0.019	Chemotherapy alone
Lower respiratory tract infections	Immunology and infection	Breast	1.43	1.21	1.69	< 0.001	Chemotherapy alone
Chronic viral hepatitis	Immunology and infection	Breast	1.30	0.76	2.20	0.337	Chemotherapy alone
HIV	Immunology and infection	Breast	1.77	0.84	3.75	0.133	Chemotherapy alone
Infection of bones and joints	Immunology and infection	Breast	0.79	0.47	1.33	0.375	Chemotherapy alone
Infection of skin and subcutaneous tissues	Immunology and infection	Breast	1.20	0.94	1.54	0.146	Chemotherapy alone
Infections of other or unspecified organs	Immunology and infection	Breast	1.12	0.92	1.35	0.264	Chemotherapy alone
Infections of the heart	Immunology and infection	Breast	1.01	0.58	1.75	0.978	Chemotherapy alone
Meningitis	Immunology and infection	Breast	1.31	0.79	2.15	0.294	Chemotherapy alone
Non-acute cystitis	Immunology and infection	Breast	1.12	0.62	2.04	0.709	Chemotherapy alone
Peritonitis	Immunology and infection	Breast	1.16	0.97	1.38	0.101	Chemotherapy alone
Viral diseases (excluding chronic hepatitis/HIV)	Immunology and infection	Breast	0.78	0.50	1.21	0.266	Chemotherapy alone
Enthesopathies and synovial disorders	Musculoskeletal	Breast	0.87	0.75	1.02	0.08	Chemotherapy alone
Osteoarthritis (excluding spine)	Musculoskeletal	Breast	0.93	0.81	1.05	0.234	Chemotherapy alone
Osteoporosis	Musculoskeletal	Breast	1.10	0.91	1.34	0.326	Chemotherapy alone
Rheumatoid arthritis	Musculoskeletal	Breast	0.72	0.48	1.09	0.123	Chemotherapy alone
Scoliosis	Musculoskeletal	Breast	1.27	0.78	2.06	0.342	Chemotherapy alone
Spinal stenosis	Musculoskeletal	Breast	1.17	0.77	1.79	0.461	Chemotherapy alone
Dementia	Neurological	Breast	2.67	2.12	3.36	< 0.001	Chemotherapy alone
Epilepsy	Neurological	Breast	1.80	1.12	2.90	0.016	Chemotherapy alone
Intracerebral haemorrhage	Neurological	Breast	1.58	1.03	2.42	0.037	Chemotherapy alone
Intracranial hypertension	Neurological	Breast	2.93	1.37	6.28	0.006	Chemotherapy alone
Disorders of autonomic nervous system	Neurological	Breast	1.11	0.62	1.99	0.722	Chemotherapy alone
Peripheral neuropathies	Neurological	Breast	0.82	0.63	1.07	0.151	Chemotherapy alone
Subarachnoid haemorrhage	Neurological	Breast	1.44	0.93	2.24	0.105	Chemotherapy alone
Asthma	Pulmonary	Breast	0.77	0.63	0.95	0.015	Chemotherapy alone
Other interstitial pulmonary diseases with fibrosis	Pulmonary	Breast	2.34	1.08	5.08	0.032	Chemotherapy alone
Bronchiectasis	Pulmonary	Breast	1.03	0.57	1.85	0.931	Chemotherapy alone
COPD	Pulmonary	Breast	0.97	0.79	1.18	0.762	Chemotherapy alone
End stage renal disease	Renal	Breast	1.18	0.88	1.57	0.264	Chemotherapy alone
Glomerulonephritis	Renal	Breast	1.14	0.64	2.04	0.646	Chemotherapy alone
Obstructive and reflux uropathy	Renal	Breast	0.62	0.30	1.25	0.178	Chemotherapy alone
Urolithiasis	Renal	Breast	0.61	0.36	1.02	0.057	Chemotherapy alone
Atrial fibrillation	Cardiovascular	Breast	0.73	0.60	0.89	0.002	Chemotherapy, radiotherapy & surgery
Coronary heart disease not otherwise specified	Cardiovascular	Breast	0.78	0.69	0.87	< 0.001	Chemotherapy, radiotherapy & surgery
Heart failure	Cardiovascular	Breast	0.51	0.39	0.67	< 0.001	Chemotherapy, radiotherapy & surgery
Hypertension	Cardiovascular	Breast	0.82	0.75	0.90	< 0.001	Chemotherapy, radiotherapy & surgery
Ischaemic stroke	Cardiovascular	Breast	0.60	0.40	0.91	0.016	Chemotherapy, radiotherapy & surgery
Left bundle branch block	Cardiovascular	Breast	0.30	0.16	0.57	< 0.001	Chemotherapy, radiotherapy & surgery
Myocardial infarction	Cardiovascular	Breast	0.67	0.54	0.84	< 0.001	Chemotherapy, radiotherapy & surgery
Other cardiomyopathy	Cardiovascular	Breast	0.65	0.52	0.81	< 0.001	Chemotherapy, radiotherapy & surgery
Peripheral arterial disease	Cardiovascular	Breast	0.50	0.34	0.73	< 0.001	Chemotherapy, radiotherapy & surgery
Raynaud's syndrome	Cardiovascular	Breast	0.62	0.42	0.91	0.015	Chemotherapy, radiotherapy & surgery
Stable angina	Cardiovascular	Breast	0.72	0.58	0.88	0.002	Chemotherapy, radiotherapy & surgery
Stroke (not otherwise specified)	Cardiovascular	Breast	0.63	0.46	0.86	0.003	Chemotherapy, radiotherapy & surgery
Unstable angina	Cardiovascular	Breast	0.65	0.51	0.82	< 0.001	Chemotherapy, radiotherapy & surgery
Venous thromboembolic disease	Cardiovascular	Breast	0.65	0.50	0.84	0.001	Chemotherapy, radiotherapy & surgery
Abdominal aortic aneurysm	Cardiovascular	Breast	1.18	0.60	2.34	0.631	Chemotherapy, radiotherapy & surgery
Nonrheumatic aortic valve disorders	Cardiovascular	Breast	0.73	0.48	1.09	0.124	Chemotherapy, radiotherapy & surgery
Nonrheumatic mitral valve disorders	Cardiovascular	Breast	0.69	0.44	1.08	0.102	Chemotherapy, radiotherapy & surgery
Sick sinus syndrome	Cardiovascular	Breast	0.85	0.48	1.50	0.571	Chemotherapy, radiotherapy & surgery
Supraventricular tachycardia	Cardiovascular	Breast	0.96	0.62	1.47	0.848	Chemotherapy, radiotherapy & surgery
Transient ischaemic attack	Cardiovascular	Breast	0.82	0.63	1.05	0.113	Chemotherapy, radiotherapy & surgery
Ventricular tachycardia	Cardiovascular	Breast	0.94	0.61	1.46	0.787	Chemotherapy, radiotherapy & surgery
Diabetes	Endocrine	Breast	0.88	0.78	0.99	0.033	Chemotherapy, radiotherapy & surgery
Diabetic neurological complications	Endocrine	Breast	0.96	0.80	1.15	0.675	Chemotherapy, radiotherapy & surgery
Diabetic ophthalmic complications	Endocrine	Breast	0.89	0.75	1.05	0.153	Chemotherapy, radiotherapy & surgery
Hyperparathyroidism	Endocrine	Breast	1.15	0.60	2.21	0.672	Chemotherapy, radiotherapy & surgery
Hypo or hyperthyroidism	Endocrine	Breast	0.98	0.84	1.15	0.818	Chemotherapy, radiotherapy & surgery
Barrett's oesophagus	Gastrointestinal	Breast	0.58	0.40	0.85	0.005	Chemotherapy, radiotherapy & surgery
Oesophagitis and oesophageal ulcer	Gastrointestinal	Breast	0.83	0.71	0.96	0.012	Chemotherapy, radiotherapy & surgery
Ulcerative colitis	Gastrointestinal	Breast	1.97	1.03	3.77	0.041	Chemotherapy, radiotherapy & surgery
Alcoholic liver disease	Gastrointestinal	Breast	0.93	0.48	1.78	0.827	Chemotherapy, radiotherapy & surgery
Cholangitis	Gastrointestinal	Breast	1.06	0.85	1.32	0.612	Chemotherapy, radiotherapy & surgery
Cholecystitis	Gastrointestinal	Breast	1.02	0.82	1.25	0.885	Chemotherapy, radiotherapy & surgery
Cholelithiasis	Gastrointestinal	Breast	1.06	0.87	1.28	0.558	Chemotherapy, radiotherapy & surgery
Coeliac disease	Gastrointestinal	Breast	1.33	0.85	2.09	0.214	Chemotherapy, radiotherapy & surgery
Diverticular disease of intestine	Gastrointestinal	Breast	1.00	0.85	1.19	0.971	Chemotherapy, radiotherapy & surgery
Fatty liver	Gastrointestinal	Breast	1.09	0.71	1.65	0.696	Chemotherapy, radiotherapy & surgery
Gastritis and duodenitis	Gastrointestinal	Breast	0.94	0.80	1.12	0.512	Chemotherapy, radiotherapy & surgery
Gastrooesophageal reflux disease	Gastrointestinal	Breast	0.88	0.75	1.03	0.111	Chemotherapy, radiotherapy & surgery

Hepatic failure	Gastrointestinal	Breast	0.86	0.52	1.42	0.556	Chemotherapy, radiotherapy & surgery
Portal hypertension	Gastrointestinal	Breast	0.69	0.36	1.32	0.267	Chemotherapy, radiotherapy & surgery
Other anaemias	Haematological	Breast	1.00	0.83	1.20	0.959	Chemotherapy, radiotherapy & surgery
Infections of other or unspecified organs	Immunology and infection	Breast	0.77	0.64	0.93	0.007	Chemotherapy, radiotherapy & surgery
Lower respiratory tract infections	Immunology and infection	Breast	0.75	0.62	0.89	0.002	Chemotherapy, radiotherapy & surgery
Meningitis	Immunology and infection	Breast	0.51	0.28	0.95	0.033	Chemotherapy, radiotherapy & surgery
Infections of the digestive system	Immunology and infection	Breast	1.30	1.02	1.64	0.031	Chemotherapy, radiotherapy & surgery
Chronic viral hepatitis	Immunology and infection	Breast	1.02	0.68	1.53	0.93	Chemotherapy, radiotherapy & surgery
HIV	Immunology and infection	Breast	0.88	0.46	1.67	0.691	Chemotherapy, radiotherapy & surgery
Infection of bones and joints	Immunology and infection	Breast	0.71	0.41	1.20	0.2	Chemotherapy, radiotherapy & surgery
Infection of skin and subcutaneous tissues	Immunology and infection	Breast	0.84	0.66	1.07	0.162	Chemotherapy, radiotherapy & surgery
Infections of the heart	Immunology and infection	Breast	0.55	0.30	1.01	0.054	Chemotherapy, radiotherapy & surgery
Lupus erythematosus (local and systemic)	Immunology and infection	Breast	1.67	0.74	3.74	0.217	Chemotherapy, radiotherapy & surgery
Non-acute cystitis	Immunology and infection	Breast	0.96	0.58	1.59	0.881	Chemotherapy, radiotherapy & surgery
Peritonitis	Immunology and infection	Breast	1.16	0.99	1.36	0.058	Chemotherapy, radiotherapy & surgery
Viral diseases (excluding chronic hepatitis/HIV)	Immunology and infection	Breast	0.87	0.62	1.21	0.402	Chemotherapy, radiotherapy & surgery
Enteropathic arthropathy	Musculoskeletal	Breast	1.15	0.51	2.61	0.734	Chemotherapy, radiotherapy & surgery
Enthesopathies and synovial disorders	Musculoskeletal	Breast	1.06	0.95	1.18	0.308	Chemotherapy, radiotherapy & surgery
Osteoarthritis (excluding spine)	Musculoskeletal	Breast	0.96	0.86	1.06	0.434	Chemotherapy, radiotherapy & surgery
Osteoporosis	Musculoskeletal	Breast	0.93	0.78	1.11	0.414	Chemotherapy, radiotherapy & surgery
Rheumatoid arthritis	Musculoskeletal	Breast	0.71	0.49	1.02	0.066	Chemotherapy, radiotherapy & surgery
Spinal stenosis	Musculoskeletal	Breast	0.89	0.58	1.36	0.582	Chemotherapy, radiotherapy & surgery
Dementia	Neurological	Breast	0.25	0.15	0.44	< 0.001	Chemotherapy, radiotherapy & surgery
Epilepsy	Neurological	Breast	0.51	0.29	0.92	0.025	Chemotherapy, radiotherapy & surgery
Bell's palsy	Neurological	Breast	0.86	0.47	1.58	0.619	Chemotherapy, radiotherapy & surgery
Disorders of autonomic nervous system	Neurological	Breast	1.01	0.59	1.74	0.97	Chemotherapy, radiotherapy & surgery
Essential tremor	Neurological	Breast	1.20	0.57	2.52	0.635	Chemotherapy, radiotherapy & surgery
Intracerebral haemorrhage	Neurological	Breast	0.68	0.39	1.18	0.168	Chemotherapy, radiotherapy & surgery
Peripheral neuropathies	Neurological	Breast	1.14	0.94	1.38	0.2	Chemotherapy, radiotherapy & surgery
Subarachnoid haemorrhage	Neurological	Breast	0.64	0.37	1.11	0.114	Chemotherapy, radiotherapy & surgery
Asthma	Pulmonary	Breast	0.74	0.63	0.87	< 0.001	Chemotherapy, radiotherapy & surgery
COPD	Pulmonary	Breast	0.81	0.68	0.97	0.022	Chemotherapy, radiotherapy & surgery
End stage renal disease	Renal	Breast	0.70	0.51	0.97	0.032	Chemotherapy, radiotherapy & surgery
Glomerulonephritis	Renal	Breast	1.35	0.81	2.26	0.244	Chemotherapy, radiotherapy & surgery
Obstructive and reflux uropathy	Renal	Breast	0.56	0.29	1.08	0.082	Chemotherapy, radiotherapy & surgery
Urolithiasis	Renal	Breast	0.78	0.53	1.14	0.194	Chemotherapy, radiotherapy & surgery
Atrial fibrillation	Cardiovascular	Breast	0.74	0.63	0.87	< 0.001	Radiotherapy & surgery
Atrioventricular block, complete	Cardiovascular	Breast	0.57	0.38	0.87	0.009	Radiotherapy & surgery
Atrioventricular block, first degree	Cardiovascular	Breast	0.57	0.38	0.87	0.009	Radiotherapy & surgery
Atrioventricular block, second degree	Cardiovascular	Breast	0.57	0.38	0.87	0.009	Radiotherapy & surgery
Heart failure	Cardiovascular	Breast	0.56	0.46	0.68	< 0.001	Radiotherapy & surgery
Hypertension	Cardiovascular	Breast	0.86	0.80	0.92	< 0.001	Radiotherapy & surgery
Ischaemic stroke	Cardiovascular	Breast	0.53	0.37	0.75	< 0.001	Radiotherapy & surgery
Left bundle branch block	Cardiovascular	Breast	0.63	0.43	0.93	0.019	Radiotherapy & surgery
Stroke (not otherwise specified)	Cardiovascular	Breast	0.77	0.60	0.98	0.031	Radiotherapy & surgery
Venous thromboembolic disease	Cardiovascular	Breast	0.69	0.56	0.85	< 0.001	Radiotherapy & surgery
Abdominal aortic aneurysm	Cardiovascular	Breast	1.57	0.93	2.65	0.091	Radiotherapy & surgery
Bifascicular block	Cardiovascular	Breast	0.98	0.57	1.67	0.929	Radiotherapy & surgery
Coronary heart disease not otherwise specified	Cardiovascular	Breast	0.96	0.88	1.06	0.403	Radiotherapy & surgery
Dilated cardiomyopathy	Cardiovascular	Breast	1.51	0.65	3.50	0.336	Radiotherapy & surgery
Hypertrophic cardiomyopathy	Cardiovascular	Breast	1.86	0.81	4.25	0.143	Radiotherapy & surgery
Multiple valve disease	Cardiovascular	Breast	0.70	0.47	1.02	0.064	Radiotherapy & surgery
Myocardial infarction	Cardiovascular	Breast	0.96	0.81	1.12	0.584	Radiotherapy & surgery
Nonrheumatic aortic valve disorders	Cardiovascular	Breast	0.76	0.55	1.05	0.1	Radiotherapy & surgery
Nonrheumatic mitral valve disorders	Cardiovascular	Breast	0.97	0.70	1.33	0.838	Radiotherapy & surgery
Other cardiomyopathy	Cardiovascular	Breast	0.89	0.76	1.05	0.182	Radiotherapy & surgery
Pericardial effusion (noninflammatory)	Cardiovascular	Breast	1.63	0.77	3.44	0.2	Radiotherapy & surgery
Peripheral arterial disease	Cardiovascular	Breast	0.83	0.64	1.08	0.162	Radiotherapy & surgery
Primary pulmonary hypertension	Cardiovascular	Breast	0.82	0.43	1.58	0.554	Radiotherapy & surgery
Raynaud's syndrome	Cardiovascular	Breast	1.07	0.81	1.41	0.625	Radiotherapy & surgery
Right bundle branch block	Cardiovascular	Breast	0.98	0.57	1.70	0.956	Radiotherapy & surgery
Sick sinus syndrome	Cardiovascular	Breast	0.85	0.53	1.36	0.489	Radiotherapy & surgery
Stable angina	Cardiovascular	Breast	0.92	0.78	1.08	0.298	Radiotherapy & surgery
Supraventricular tachycardia	Cardiovascular	Breast	0.98	0.68	1.41	0.899	Radiotherapy & surgery
Transient ischaemic attack	Cardiovascular	Breast	0.86	0.71	1.05	0.147	Radiotherapy & surgery
Trifascicular block	Cardiovascular	Breast	0.94	0.51	1.74	0.845	Radiotherapy & surgery
Unstable angina	Cardiovascular	Breast	1.01	0.85	1.19	0.936	Radiotherapy & surgery
Ventricular tachycardia	Cardiovascular	Breast	0.93	0.64	1.36	0.725	Radiotherapy & surgery
Diabetes	Endocrine	Breast	0.95	0.86	1.05	0.328	Radiotherapy & surgery
Diabetic neurological complications	Endocrine	Breast	0.93	0.80	1.09	0.38	Radiotherapy & surgery
Diabetic ophthalmic complications	Endocrine	Breast	0.95	0.83	1.09	0.451	Radiotherapy & surgery
Hyperparathyroidism	Endocrine	Breast	0.72	0.39	1.33	0.297	Radiotherapy & surgery
Hypo or hyperthyroidism	Endocrine	Breast	0.95	0.83	1.09	0.446	Radiotherapy & surgery
Alcoholic liver disease	Gastrointestinal	Breast	1.75	1.09	2.82	0.02	Radiotherapy & surgery
Angiodysplasia of colon	Gastrointestinal	Breast	1.05	0.53	2.10	0.882	Radiotherapy & surgery
Barrett's oesophagus	Gastrointestinal	Breast	0.80	0.61	1.06	0.123	Radiotherapy & surgery
Cholangitis	Gastrointestinal	Breast	1.04	0.86	1.26	0.689	Radiotherapy & surgery
Cholecystitis	Gastrointestinal	Breast	0.99	0.83	1.19	0.951	Radiotherapy & surgery
Cholelithiasis	Gastrointestinal	Breast	1.04	0.88	1.23	0.654	Radiotherapy & surgery

Coeliac disease	Gastrointestinal	Breast	0.63	0.39	1.01	0.053	Radiotherapy & surgery
Crohn's disease	Gastrointestinal	Breast	1.05	0.50	2.19	0.899	Radiotherapy & surgery
Diverticular disease of intestine	Gastrointestinal	Breast	0.99	0.87	1.14	0.943	Radiotherapy & surgery
Fatty liver	Gastrointestinal	Breast	1.21	0.86	1.70	0.271	Radiotherapy & surgery
Gastritis and duodenitis	Gastrointestinal	Breast	0.91	0.78	1.05	0.192	Radiotherapy & surgery
Gastrooesophageal reflux disease	Gastrointestinal	Breast	1.06	0.93	1.20	0.388	Radiotherapy & surgery
Hepatic failure	Gastrointestinal	Breast	0.78	0.51	1.20	0.26	Radiotherapy & surgery
Liver fibrosis sclerosis and cirrhosis	Gastrointestinal	Breast	1.07	0.56	2.06	0.828	Radiotherapy & surgery
Oesophagitis and oesophageal ulcer	Gastrointestinal	Breast	1.01	0.89	1.14	0.896	Radiotherapy & surgery
Portal hypertension	Gastrointestinal	Breast	0.67	0.40	1.12	0.127	Radiotherapy & surgery
Ulcerative colitis	Gastrointestinal	Breast	1.47	0.79	2.75	0.226	Radiotherapy & surgery
Other anaemias	Haematological	Breast	0.76	0.64	0.90	0.002	Radiotherapy & surgery
Agranulocytosis	Haematological	Breast	1.30	0.69	2.44	0.416	Radiotherapy & surgery
Primary or idiopathic thrombocytopaenia	Haematological	Breast	1.28	0.64	2.55	0.489	Radiotherapy & surgery
Secondary or other thrombocytopaenia	Haematological	Breast	0.71	0.43	1.18	0.188	Radiotherapy & surgery
Thrombophilia	Haematological	Breast	0.88	0.48	1.62	0.685	Radiotherapy & surgery
Infection of skin and subcutaneous tissues	Immunology and infection	Breast	0.63	0.51	0.79	< 0.001	Radiotherapy & surgery
Infections of other or unspecified organs	Immunology and infection	Breast	0.75	0.64	0.88	0.001	Radiotherapy & surgery
Infections of the heart	Immunology and infection	Breast	0.58	0.36	0.93	0.025	Radiotherapy & surgery
Lower respiratory tract infections	Immunology and infection	Breast	0.69	0.59	0.80	< 0.001	Radiotherapy & surgery
Meningitis	Immunology and infection	Breast	0.58	0.36	0.95	0.029	Radiotherapy & surgery
Viral diseases (excluding chronic hepatitis/HIV)	Immunology and infection	Breast	0.42	0.29	0.61	< 0.001	Radiotherapy & surgery
Autoimmune liver disease	Immunology and infection	Breast	1.31	0.60	2.86	0.494	Radiotherapy & surgery
Chronic viral hepatitis	Immunology and infection	Breast	1.09	0.75	1.58	0.644	Radiotherapy & surgery
HIV	Immunology and infection	Breast	0.92	0.50	1.71	0.791	Radiotherapy & surgery
Infection of bones and joints	Immunology and infection	Breast	0.70	0.44	1.10	0.123	Radiotherapy & surgery
Infection of liver	Immunology and infection	Breast	1.08	0.48	2.45	0.848	Radiotherapy & surgery
Infections of the digestive system	Immunology and infection	Breast	0.89	0.72	1.10	0.275	Radiotherapy & surgery
Non-acute cystitis	Immunology and infection	Breast	0.99	0.64	1.54	0.962	Radiotherapy & surgery
Peritonitis	Immunology and infection	Breast	1.01	0.89	1.16	0.832	Radiotherapy & surgery
Osteoporosis	Musculoskeletal	Breast	0.84	0.72	0.97	0.022	Radiotherapy & surgery
Scoliosis	Musculoskeletal	Breast	0.50	0.31	0.82	0.006	Radiotherapy & surgery
Enthesopathies and synovial disorders	Musculoskeletal	Breast	1.22	1.11	1.34	< 0.001	Radiotherapy & surgery
Enteropathic arthropathy	Musculoskeletal	Breast	1.31	0.64	2.67	0.453	Radiotherapy & surgery
Osteoarthritis (excluding spine)	Musculoskeletal	Breast	1.09	1.00	1.18	0.063	Radiotherapy & surgery
Rheumatoid arthritis	Musculoskeletal	Breast	0.92	0.69	1.22	0.551	Radiotherapy & surgery
Spinal stenosis	Musculoskeletal	Breast	0.86	0.60	1.22	0.396	Radiotherapy & surgery
Dementia	Neurological	Breast	0.36	0.25	0.51	< 0.001	Radiotherapy & surgery
Intracerebral haemorrhage	Neurological	Breast	0.42	0.24	0.72	0.002	Radiotherapy & surgery
Subarachnoid haemorrhage	Neurological	Breast	0.37	0.21	0.65	0.001	Radiotherapy & surgery
Bell's palsy	Neurological	Breast	0.67	0.38	1.16	0.152	Radiotherapy & surgery
Disorders of autonomic nervous system	Neurological	Breast	0.89	0.54	1.44	0.625	Radiotherapy & surgery
Epilepsy	Neurological	Breast	0.64	0.40	1.03	0.066	Radiotherapy & surgery
Essential tremor	Neurological	Breast	0.80	0.40	1.63	0.545	Radiotherapy & surgery
Intracranial hypertension	Neurological	Breast	0.92	0.43	1.95	0.824	Radiotherapy & surgery
Multiple sclerosis	Neurological	Breast	1.83	0.87	3.87	0.111	Radiotherapy & surgery
Peripheral neuropathies	Neurological	Breast	1.12	0.95	1.33	0.182	Radiotherapy & surgery
Bronchiectasis	Pulmonary	Breast	0.54	0.32	0.90	0.019	Radiotherapy & surgery
Asthma	Pulmonary	Breast	0.94	0.82	1.08	0.384	Radiotherapy & surgery
COPD	Pulmonary	Breast	0.87	0.75	1.01	0.069	Radiotherapy & surgery
End stage renal disease	Renal	Breast	0.52	0.39	0.69	< 0.001	Radiotherapy & surgery
Obstructive and reflux uropathy	Renal	Breast	0.56	0.33	0.96	0.035	Radiotherapy & surgery
Glomerulonephritis	Renal	Breast	1.07	0.68	1.68	0.786	Radiotherapy & surgery
Urolithiasis	Renal	Breast	0.83	0.60	1.15	0.26	Radiotherapy & surgery
Hypertension	Cardiovascular	Breast	0.78	0.71	0.87	< 0.001	Radiotherapy alone
Venous thromboembolic disease	Cardiovascular	Breast	0.70	0.54	0.91	0.009	Radiotherapy alone
Abdominal aortic aneurysm	Cardiovascular	Breast	2.12	1.16	3.86	0.014	Radiotherapy alone
Atrial fibrillation	Cardiovascular	Breast	0.96	0.79	1.15	0.635	Radiotherapy alone
Atrioventricular block, complete	Cardiovascular	Breast	0.81	0.51	1.28	0.364	Radiotherapy alone
Atrioventricular block, first degree	Cardiovascular	Breast	0.81	0.51	1.28	0.364	Radiotherapy alone
Atrioventricular block, second degree	Cardiovascular	Breast	0.81	0.51	1.28	0.364	Radiotherapy alone
Bifascicular block	Cardiovascular	Breast	1.42	0.80	2.53	0.231	Radiotherapy alone
Coronary heart disease not otherwise specified	Cardiovascular	Breast	0.91	0.80	1.03	0.138	Radiotherapy alone
Heart failure	Cardiovascular	Breast	0.82	0.66	1.03	0.087	Radiotherapy alone
Ischaemic stroke	Cardiovascular	Breast	0.91	0.63	1.32	0.624	Radiotherapy alone
Left bundle branch block	Cardiovascular	Breast	0.84	0.55	1.30	0.447	Radiotherapy alone
Multiple valve disease	Cardiovascular	Breast	0.62	0.37	1.04	0.072	Radiotherapy alone
Myocardial infarction	Cardiovascular	Breast	0.94	0.76	1.16	0.565	Radiotherapy alone
Nonrheumatic aortic valve disorders	Cardiovascular	Breast	0.93	0.63	1.36	0.71	Radiotherapy alone
Nonrheumatic mitral valve disorders	Cardiovascular	Breast	0.72	0.45	1.14	0.156	Radiotherapy alone
Other cardiomyopathy	Cardiovascular	Breast	0.99	0.80	1.21	0.893	Radiotherapy alone
Peripheral arterial disease	Cardiovascular	Breast	0.80	0.57	1.13	0.207	Radiotherapy alone
Raynaud's syndrome	Cardiovascular	Breast	0.67	0.44	1.01	0.054	Radiotherapy alone
Right bundle branch block	Cardiovascular	Breast	1.48	0.83	2.65	0.186	Radiotherapy alone
Sick sinus syndrome	Cardiovascular	Breast	0.70	0.36	1.35	0.289	Radiotherapy alone
Stable angina	Cardiovascular	Breast	0.98	0.80	1.20	0.847	Radiotherapy alone
Stroke (not otherwise specified)	Cardiovascular	Breast	0.96	0.72	1.27	0.774	Radiotherapy alone
Supraventricular tachycardia	Cardiovascular	Breast	1.04	0.65	1.67	0.86	Radiotherapy alone
Transient ischaemic attack	Cardiovascular	Breast	1.11	0.88	1.40	0.392	Radiotherapy alone
Trifascicular block	Cardiovascular	Breast	1.63	0.88	3.03	0.122	Radiotherapy alone
Unstable angina	Cardiovascular	Breast	0.89	0.71	1.11	0.292	Radiotherapy alone

Ventricular tachycardia	Cardiovascular	Breast	1.14	0.72	1.82	0.576	Radiotherapy alone
Diabetes	Endocrine	Breast	0.95	0.82	1.08	0.422	Radiotherapy alone
Diabetic neurological complications	Endocrine	Breast	1.11	0.92	1.33	0.291	Radiotherapy alone
Diabetic ophthalmic complications	Endocrine	Breast	1.00	0.84	1.18	0.971	Radiotherapy alone
Hyperparathyroidism	Endocrine	Breast	1.22	0.62	2.39	0.56	Radiotherapy alone
Hypo or hyperthyroidism	Endocrine	Breast	0.90	0.74	1.08	0.254	Radiotherapy alone
Barrett's oesophagus	Gastrointestinal	Breast	0.62	0.42	0.93	0.02	Radiotherapy alone
Fatty liver	Gastrointestinal	Breast	1.53	1.02	2.29	0.042	Radiotherapy alone
Alcoholic liver disease	Gastrointestinal	Breast	1.61	0.87	2.99	0.129	Radiotherapy alone
Cholangitis	Gastrointestinal	Breast	1.22	0.96	1.55	0.099	Radiotherapy alone
Cholecystitis	Gastrointestinal	Breast	1.15	0.91	1.45	0.227	Radiotherapy alone
Cholelithiasis	Gastrointestinal	Breast	1.19	0.97	1.48	0.099	Radiotherapy alone
Coeliac disease	Gastrointestinal	Breast	0.87	0.48	1.56	0.633	Radiotherapy alone
Diverticular disease of intestine	Gastrointestinal	Breast	1.00	0.83	1.19	0.974	Radiotherapy alone
Gastritis and duodenitis	Gastrointestinal	Breast	0.83	0.68	1.02	0.076	Radiotherapy alone
Gastrooesophageal reflux disease	Gastrointestinal	Breast	0.92	0.77	1.11	0.388	Radiotherapy alone
Hepatic failure	Gastrointestinal	Breast	1.44	0.91	2.28	0.115	Radiotherapy alone
Oesophagitis and oesophageal ulcer	Gastrointestinal	Breast	0.90	0.76	1.06	0.206	Radiotherapy alone
Portal hypertension	Gastrointestinal	Breast	1.56	0.93	2.60	0.091	Radiotherapy alone
Agranulocytosis	Haematological	Breast	2.02	0.99	4.12	0.053	Radiotherapy alone
Other anaemias	Haematological	Breast	1.01	0.82	1.24	0.928	Radiotherapy alone
Chronic viral hepatitis	Immunology and infection	Breast	1.24	0.76	2.03	0.392	Radiotherapy alone
Infection of bones and joints	Immunology and infection	Breast	1.41	0.90	2.19	0.13	Radiotherapy alone
Infection of skin and subcutaneous tissues	Immunology and infection	Breast	0.88	0.68	1.15	0.35	Radiotherapy alone
Infections of other or unspecified organs	Immunology and infection	Breast	0.94	0.77	1.15	0.56	Radiotherapy alone
Infections of the heart	Immunology and infection	Breast	0.85	0.48	1.50	0.57	Radiotherapy alone
Infections of the digestive system	Immunology and infection	Breast	1.17	0.91	1.51	0.23	Radiotherapy alone
Lower respiratory tract infections	Immunology and infection	Breast	0.94	0.79	1.13	0.536	Radiotherapy alone
Meningitis	Immunology and infection	Breast	1.20	0.72	1.98	0.484	Radiotherapy alone
Non-acute cystitis	Immunology and infection	Breast	0.76	0.39	1.46	0.406	Radiotherapy alone
Peritonitis	Immunology and infection	Breast	1.03	0.87	1.23	0.718	Radiotherapy alone
Viral diseases (excluding chronic hepatitis/HIV)	Immunology and infection	Breast	0.75	0.49	1.15	0.189	Radiotherapy alone
Enthesopathies and synovial disorders	Musculoskeletal	Breast	1.06	0.93	1.21	0.415	Radiotherapy alone
Osteoarthritis (excluding spine)	Musculoskeletal	Breast	0.96	0.85	1.08	0.508	Radiotherapy alone
Osteoporosis	Musculoskeletal	Breast	0.85	0.70	1.04	0.122	Radiotherapy alone
Rheumatoid arthritis	Musculoskeletal	Breast	0.74	0.50	1.10	0.137	Radiotherapy alone
Scoliosis	Musculoskeletal	Breast	0.61	0.33	1.12	0.11	Radiotherapy alone
Spinal stenosis	Musculoskeletal	Breast	1.17	0.77	1.77	0.461	Radiotherapy alone
Dementia	Neurological	Breast	0.47	0.32	0.70	< 0.001	Radiotherapy alone
Essential tremor	Neurological	Breast	2.19	1.12	4.28	0.022	Radiotherapy alone
Bell's palsy	Neurological	Breast	0.94	0.48	1.83	0.848	Radiotherapy alone
Disorders of autonomic nervous system	Neurological	Breast	1.43	0.84	2.44	0.184	Radiotherapy alone
Epilepsy	Neurological	Breast	0.94	0.54	1.65	0.831	Radiotherapy alone
Intracerebral haemorrhage	Neurological	Breast	0.83	0.49	1.42	0.499	Radiotherapy alone
Peripheral neuropathies	Neurological	Breast	0.86	0.67	1.10	0.226	Radiotherapy alone
Subarachnoid haemorrhage	Neurological	Breast	0.95	0.57	1.58	0.841	Radiotherapy alone
Asthma	Pulmonary	Breast	0.74	0.61	0.91	0.004	Radiotherapy alone
Bronchiectasis	Pulmonary	Breast	0.94	0.53	1.65	0.832	Radiotherapy alone
COPD	Pulmonary	Breast	0.94	0.77	1.13	0.502	Radiotherapy alone
End stage renal disease	Renal	Breast	1.13	0.85	1.50	0.407	Radiotherapy alone
Glomerulonephritis	Renal	Breast	1.07	0.60	1.90	0.827	Radiotherapy alone
Urolithiasis	Renal	Breast	0.86	0.56	1.33	0.495	Radiotherapy alone
Coronary heart disease not otherwise specified	Cardiovascular	Cervix	0.71	0.38	1.33	0.288	Chemotherapy & radiotherapy
Hypertension	Cardiovascular	Cervix	1.50	0.87	2.58	0.148	Chemotherapy & radiotherapy
Diabetes	Endocrine	Cervix	1.08	0.54	2.16	0.82	Chemotherapy & radiotherapy
Enthesopathies and synovial disorders	Musculoskeletal	Cervix	1.23	0.62	2.45	0.56	Chemotherapy & radiotherapy
Osteoarthritis (excluding spine)	Musculoskeletal	Cervix	0.85	0.41	1.77	0.672	Chemotherapy & radiotherapy
Asthma	Pulmonary	Cervix	1.25	0.63	2.49	0.53	Chemotherapy & radiotherapy
Urolithiasis	Renal	Cervix	1.84	0.80	4.21	0.151	Chemotherapy & radiotherapy
Obstructive and reflux uropathy	Renal	Cervix	1.74	0.74	4.08	0.206	Chemotherapy & radiotherapy
Hypertension	Cardiovascular	Cervix	1.27	0.67	2.40	0.47	Chemotherapy, radiotherapy & surgery
Coronary heart disease not otherwise specified	Cardiovascular	Cervix	1.31	0.80	2.13	0.285	Radiotherapy & surgery
Hypertension	Cardiovascular	Cervix	1.05	0.62	1.78	0.848	Radiotherapy & surgery
Diabetic ophthalmic complications	Endocrine	Cervix	2.77	1.19	6.44	0.018	Radiotherapy & surgery
Diabetes	Endocrine	Cervix	1.61	0.88	2.96	0.123	Radiotherapy & surgery
Hypo or hyperthyroidism	Endocrine	Cervix	1.28	0.59	2.76	0.53	Radiotherapy & surgery
Other anaemias	Haematological	Cervix	2.03	1.09	3.77	0.025	Radiotherapy & surgery
Enthesopathies and synovial disorders	Musculoskeletal	Cervix	1.15	0.60	2.19	0.672	Radiotherapy & surgery
Osteoarthritis (excluding spine)	Musculoskeletal	Cervix	1.29	0.71	2.37	0.403	Radiotherapy & surgery
Coronary heart disease not otherwise specified	Cardiovascular	Cervix	0.74	0.43	1.27	0.273	Radiotherapy alone
Hypertension	Cardiovascular	Cervix	1.10	0.67	1.79	0.713	Radiotherapy alone
Myocardial infarction	Cardiovascular	Cervix	0.86	0.36	2.02	0.721	Radiotherapy alone
Other cardiomyopathy	Cardiovascular	Cervix	0.79	0.33	1.85	0.581	Radiotherapy alone
Stable angina	Cardiovascular	Cervix	0.47	0.20	1.15	0.098	Radiotherapy alone
Diabetes	Endocrine	Cervix	1.48	0.83	2.64	0.182	Radiotherapy alone
Diabetic neurological complications	Endocrine	Cervix	2.00	0.84	4.75	0.118	Radiotherapy alone
Diabetic ophthalmic complications	Endocrine	Cervix	1.97	0.87	4.45	0.103	Radiotherapy alone
Hypo or hyperthyroidism	Endocrine	Cervix	0.95	0.43	2.12	0.901	Radiotherapy alone
Cholangitis	Gastrointestinal	Cervix	1.53	0.56	4.17	0.402	Radiotherapy alone

Cholecystitis	Gastrointestinal	Cervix	1.35	0.51	3.58	0.542	Radiotherapy alone
Cholelithiasis	Gastrointestinal	Cervix	1.40	0.55	3.53	0.481	Radiotherapy alone
Diverticular disease of intestine	Gastrointestinal	Cervix	0.71	0.30	1.67	0.435	Radiotherapy alone
Other anaemias	Haematological	Cervix	1.67	0.91	3.07	0.099	Radiotherapy alone
Infections of other or unspecified organs	Immunology and infection	Cervix	0.45	0.21	0.93	0.032	Radiotherapy alone
Lower respiratory tract infections	Immunology and infection	Cervix	1.98	1.05	3.75	0.036	Radiotherapy alone
Peritonitis	Immunology and infection	Cervix	0.79	0.37	1.69	0.543	Radiotherapy alone
Enthesopathies and synovial disorders	Musculoskeletal	Cervix	1.01	0.53	1.92	0.972	Radiotherapy alone
Osteoarthritis (excluding spine)	Musculoskeletal	Cervix	0.74	0.40	1.37	0.344	Radiotherapy alone
Osteoporosis	Musculoskeletal	Cervix	1.12	0.40	3.15	0.826	Radiotherapy alone
Asthma	Pulmonary	Cervix	1.57	0.84	2.93	0.156	Radiotherapy alone
COPD	Pulmonary	Cervix	1.33	0.67	2.64	0.414	Radiotherapy alone
Urolithiasis	Renal	Cervix	2.72	1.33	5.58	0.006	Radiotherapy alone
Obstructive and reflux uropathy	Renal	Cervix	2.84	1.37	5.91	0.005	Radiotherapy alone
Atrial fibrillation	Cardiovascular	Colon and rectum	0.63	0.47	0.83	0.001	Chemotherapy & radiotherapy
Coronary heart disease not otherwise specified	Cardiovascular	Colon and rectum	0.63	0.51	0.78	< 0.001	Chemotherapy & radiotherapy
Heart failure	Cardiovascular	Colon and rectum	0.63	0.46	0.87	0.005	Chemotherapy & radiotherapy
Ischaemic stroke	Cardiovascular	Colon and rectum	0.48	0.26	0.89	0.019	Chemotherapy & radiotherapy
Myocardial infarction	Cardiovascular	Colon and rectum	0.72	0.55	0.94	0.014	Chemotherapy & radiotherapy
Other cardiomyopathy	Cardiovascular	Colon and rectum	0.73	0.56	0.94	0.014	Chemotherapy & radiotherapy
Unstable angina	Cardiovascular	Colon and rectum	0.74	0.55	0.99	0.043	Chemotherapy & radiotherapy
Abdominal aortic aneurysm	Cardiovascular	Colon and rectum	0.98	0.66	1.45	0.921	Chemotherapy & radiotherapy
Atrioventricular block, complete	Cardiovascular	Colon and rectum	0.87	0.49	1.53	0.623	Chemotherapy & radiotherapy
Atrioventricular block, first degree	Cardiovascular	Colon and rectum	0.87	0.49	1.53	0.623	Chemotherapy & radiotherapy
Atrioventricular block, second degree	Cardiovascular	Colon and rectum	0.87	0.49	1.53	0.623	Chemotherapy & radiotherapy
Hypertension	Cardiovascular	Colon and rectum	0.96	0.83	1.11	0.58	Chemotherapy & radiotherapy
Left bundle branch block	Cardiovascular	Colon and rectum	0.87	0.50	1.51	0.623	Chemotherapy & radiotherapy
Peripheral arterial disease	Cardiovascular	Colon and rectum	0.65	0.41	1.03	0.068	Chemotherapy & radiotherapy
Raynaud's syndrome	Cardiovascular	Colon and rectum	0.79	0.47	1.33	0.376	Chemotherapy & radiotherapy
Stable angina	Cardiovascular	Colon and rectum	0.81	0.61	1.07	0.144	Chemotherapy & radiotherapy
Stroke (not otherwise specified)	Cardiovascular	Colon and rectum	0.65	0.41	1.03	0.064	Chemotherapy & radiotherapy
Transient ischaemic attack	Cardiovascular	Colon and rectum	0.85	0.59	1.22	0.376	Chemotherapy & radiotherapy
Venous thromboembolic disease	Cardiovascular	Colon and rectum	0.70	0.48	1.01	0.058	Chemotherapy & radiotherapy
Ventricular tachycardia	Cardiovascular	Colon and rectum	1.24	0.66	2.33	0.506	Chemotherapy & radiotherapy
Diabetes	Endocrine	Colon and rectum	1.01	0.83	1.23	0.91	Chemotherapy & radiotherapy
Diabetic neurological complications	Endocrine	Colon and rectum	1.18	0.95	1.46	0.125	Chemotherapy & radiotherapy
Diabetic ophthalmic complications	Endocrine	Colon and rectum	1.10	0.89	1.35	0.364	Chemotherapy & radiotherapy
Hypo or hyperthyroidism	Endocrine	Colon and rectum	0.81	0.55	1.20	0.295	Chemotherapy & radiotherapy
Diverticular disease of intestine	Gastrointestinal	Colon and rectum	0.70	0.57	0.86	0.001	Chemotherapy & radiotherapy
Gastritis and duodenitis	Gastrointestinal	Colon and rectum	0.58	0.45	0.76	< 0.001	Chemotherapy & radiotherapy
Hepatic failure	Gastrointestinal	Colon and rectum	0.58	0.34	0.99	0.046	Chemotherapy & radiotherapy
Barrett's oesophagus	Gastrointestinal	Colon and rectum	0.87	0.59	1.29	0.495	Chemotherapy & radiotherapy
Cholangitis	Gastrointestinal	Colon and rectum	0.91	0.62	1.32	0.607	Chemotherapy & radiotherapy
Cholecystitis	Gastrointestinal	Colon and rectum	0.95	0.66	1.35	0.76	Chemotherapy & radiotherapy
Cholelithiasis	Gastrointestinal	Colon and rectum	0.72	0.49	1.06	0.096	Chemotherapy & radiotherapy
Coeliac disease	Gastrointestinal	Colon and rectum	1.07	0.57	2.03	0.825	Chemotherapy & radiotherapy
Fatty liver	Gastrointestinal	Colon and rectum	0.67	0.41	1.10	0.118	Chemotherapy & radiotherapy
Gastrooesophageal reflux disease	Gastrointestinal	Colon and rectum	1.00	0.77	1.30	0.993	Chemotherapy & radiotherapy
Oesophagitis and oesophageal ulcer	Gastrointestinal	Colon and rectum	0.80	0.63	1.00	0.054	Chemotherapy & radiotherapy
Portal hypertension	Gastrointestinal	Colon and rectum	0.72	0.40	1.27	0.255	Chemotherapy & radiotherapy
Other anaemias	Haematological	Colon and rectum	0.40	0.33	0.50	< 0.001	Chemotherapy & radiotherapy
Infection of skin and subcutaneous tissues	Immunology and infection	Colon and rectum	0.63	0.42	0.95	0.029	Chemotherapy & radiotherapy
Infections of other or unspecified organs	Immunology and infection	Colon and rectum	0.60	0.46	0.79	< 0.001	Chemotherapy & radiotherapy
Infections of the digestive system	Immunology and infection	Colon and rectum	0.58	0.47	0.73	< 0.001	Chemotherapy & radiotherapy
Lower respiratory tract infections	Immunology and infection	Colon and rectum	0.72	0.57	0.91	0.007	Chemotherapy & radiotherapy
Peritonitis	Immunology and infection	Colon and rectum	0.56	0.47	0.66	< 0.001	Chemotherapy & radiotherapy
Enthesopathies and synovial disorders	Musculoskeletal	Colon and rectum	1.04	0.83	1.30	0.748	Chemotherapy & radiotherapy
Osteoarthritis (excluding spine)	Musculoskeletal	Colon and rectum	1.20	0.99	1.45	0.067	Chemotherapy & radiotherapy
Osteoporosis	Musculoskeletal	Colon and rectum	0.92	0.63	1.32	0.64	Chemotherapy & radiotherapy
Rheumatoid arthritis	Musculoskeletal	Colon and rectum	1.23	0.69	2.22	0.482	Chemotherapy & radiotherapy
Peripheral neuropathies	Neurological	Colon and rectum	0.98	0.67	1.43	0.897	Chemotherapy & radiotherapy
Asthma	Pulmonary	Colon and rectum	0.96	0.71	1.29	0.787	Chemotherapy & radiotherapy
Bronchiectasis	Pulmonary	Colon and rectum	1.86	0.98	3.52	0.058	Chemotherapy & radiotherapy
COPD	Pulmonary	Colon and rectum	0.84	0.64	1.11	0.213	Chemotherapy & radiotherapy
End stage renal disease	Renal	Colon and rectum	0.53	0.34	0.83	0.005	Chemotherapy & radiotherapy
Urolithiasis	Renal	Colon and rectum	0.87	0.56	1.35	0.544	Chemotherapy & radiotherapy
Atrial fibrillation	Cardiovascular	Colon and rectum	0.69	0.58	0.81	< 0.001	Chemotherapy & surgery
Coronary heart disease not otherwise specified	Cardiovascular	Colon and rectum	0.80	0.71	0.90	< 0.001	Chemotherapy & surgery
Heart failure	Cardiovascular	Colon and rectum	0.60	0.49	0.73	< 0.001	Chemotherapy & surgery
Ischaemic stroke	Cardiovascular	Colon and rectum	0.41	0.28	0.60	< 0.001	Chemotherapy & surgery
Myocardial infarction	Cardiovascular	Colon and rectum	0.78	0.67	0.91	0.001	Chemotherapy & surgery
Other cardiomyopathy	Cardiovascular	Colon and rectum	0.82	0.70	0.94	0.007	Chemotherapy & surgery
Peripheral arterial disease	Cardiovascular	Colon and rectum	0.75	0.57	0.97	0.028	Chemotherapy & surgery
Raynaud's syndrome	Cardiovascular	Colon and rectum	0.67	0.48	0.94	0.019	Chemotherapy & surgery
Stable angina	Cardiovascular	Colon and rectum	0.82	0.70	0.97	0.02	Chemotherapy & surgery
Stroke (not otherwise specified)	Cardiovascular	Colon and rectum	0.49	0.36	0.66	< 0.001	Chemotherapy & surgery
Transient ischaemic attack	Cardiovascular	Colon and rectum	0.73	0.58	0.93	0.01	Chemotherapy & surgery
Unstable angina	Cardiovascular	Colon and rectum	0.75	0.63	0.89	0.001	Chemotherapy & surgery
Venous thromboembolic disease	Cardiovascular	Colon and rectum	0.55	0.43	0.70	< 0.001	Chemotherapy & surgery
Abdominal aortic aneurysm	Cardiovascular	Colon and rectum	1.06	0.82	1.37	0.633	Chemotherapy & surgery
Atrioventricular block, complete	Cardiovascular	Colon and rectum	0.70	0.48	1.02	0.065	Chemotherapy & surgery

Atrioventricular block, first degree	Cardiovascular	Colon and rectum	0.70	0.48	1.02	0.065	Chemotherapy & surgery
Atrioventricular block, second degree	Cardiovascular	Colon and rectum	0.70	0.48	1.02	0.065	Chemotherapy & surgery
Bifascicular block	Cardiovascular	Colon and rectum	0.85	0.55	1.30	0.45	Chemotherapy & surgery
Hypertension	Cardiovascular	Colon and rectum	1.04	0.95	1.14	0.398	Chemotherapy & surgery
Left bundle branch block	Cardiovascular	Colon and rectum	0.81	0.57	1.13	0.217	Chemotherapy & surgery
Multiple valve disease	Cardiovascular	Colon and rectum	0.88	0.57	1.36	0.552	Chemotherapy & surgery
Nonrheumatic aortic valve disorders	Cardiovascular	Colon and rectum	0.74	0.51	1.08	0.12	Chemotherapy & surgery
Nonrheumatic mitral valve disorders	Cardiovascular	Colon and rectum	0.71	0.45	1.13	0.15	Chemotherapy & surgery
Right bundle branch block	Cardiovascular	Colon and rectum	0.87	0.56	1.33	0.511	Chemotherapy & surgery
Sick sinus syndrome	Cardiovascular	Colon and rectum	0.93	0.58	1.50	0.776	Chemotherapy & surgery
Supraventricular tachycardia	Cardiovascular	Colon and rectum	1.06	0.71	1.58	0.784	Chemotherapy & surgery
Trifascicular block	Cardiovascular	Colon and rectum	0.69	0.41	1.16	0.161	Chemotherapy & surgery
Ventricular tachycardia	Cardiovascular	Colon and rectum	1.05	0.71	1.57	0.796	Chemotherapy & surgery
Diabetes	Endocrine	Colon and rectum	0.98	0.87	1.12	0.79	Chemotherapy & surgery
Diabetic neurological complications	Endocrine	Colon and rectum	0.98	0.85	1.12	0.77	Chemotherapy & surgery
Diabetic ophthalmic complications	Endocrine	Colon and rectum	0.97	0.85	1.11	0.661	Chemotherapy & surgery
Hypo or hyperthyroidism	Endocrine	Colon and rectum	0.86	0.69	1.07	0.174	Chemotherapy & surgery
Crohn's disease	Gastrointestinal	Colon and rectum	0.56	0.34	0.93	0.024	Chemotherapy & surgery
Gastritis and duodenitis	Gastrointestinal	Colon and rectum	0.87	0.76	1.00	0.046	Chemotherapy & surgery
Coeliac disease	Gastrointestinal	Colon and rectum	1.57	1.11	2.24	0.012	Chemotherapy & surgery
Alcoholic liver disease	Gastrointestinal	Colon and rectum	1.03	0.60	1.76	0.91	Chemotherapy & surgery
Angiodysplasia of colon	Gastrointestinal	Colon and rectum	0.61	0.34	1.09	0.093	Chemotherapy & surgery
Barrett's oesophagus	Gastrointestinal	Colon and rectum	0.89	0.71	1.12	0.324	Chemotherapy & surgery
Cholangitis	Gastrointestinal	Colon and rectum	0.85	0.68	1.06	0.144	Chemotherapy & surgery
Cholecystitis	Gastrointestinal	Colon and rectum	0.86	0.70	1.07	0.177	Chemotherapy & surgery
Cholelithiasis	Gastrointestinal	Colon and rectum	0.95	0.77	1.16	0.59	Chemotherapy & surgery
Diverticular disease of intestine	Gastrointestinal	Colon and rectum	0.95	0.85	1.06	0.399	Chemotherapy & surgery
Fatty liver	Gastrointestinal	Colon and rectum	1.09	0.85	1.41	0.491	Chemotherapy & surgery
Gastrooesophageal reflux disease	Gastrointestinal	Colon and rectum	1.07	0.92	1.26	0.37	Chemotherapy & surgery
Hepatic failure	Gastrointestinal	Colon and rectum	0.97	0.74	1.27	0.84	Chemotherapy & surgery
Liver fibrosis sclerosis and cirrhosis	Gastrointestinal	Colon and rectum	0.72	0.41	1.29	0.273	Chemotherapy & surgery
Oesophagitis and oesophageal ulcer	Gastrointestinal	Colon and rectum	0.95	0.83	1.09	0.455	Chemotherapy & surgery
Portal hypertension	Gastrointestinal	Colon and rectum	1.11	0.83	1.49	0.48	Chemotherapy & surgery
Ulcerative colitis	Gastrointestinal	Colon and rectum	1.06	0.67	1.69	0.803	Chemotherapy & surgery
Other anaemias	Haematological	Colon and rectum	0.85	0.77	0.94	0.002	Chemotherapy & surgery
Secondary or other thrombocytopaenia	Haematological	Colon and rectum	0.72	0.43	1.21	0.214	Chemotherapy & surgery
Infections of other or unspecified organs	Immunology and infection	Colon and rectum	0.67	0.57	0.78	< 0.001	Chemotherapy & surgery
Lower respiratory tract infections	Immunology and infection	Colon and rectum	0.70	0.60	0.80	< 0.001	Chemotherapy & surgery
Peritonitis	Immunology and infection	Colon and rectum	0.91	0.83	1.00	0.039	Chemotherapy & surgery
Autoimmune liver disease	Immunology and infection	Colon and rectum	0.74	0.40	1.38	0.342	Chemotherapy & surgery
Chronic viral hepatitis	Immunology and infection	Colon and rectum	1.02	0.64	1.61	0.946	Chemotherapy & surgery
Infection of skin and subcutaneous tissues	Immunology and infection	Colon and rectum	0.82	0.65	1.03	0.087	Chemotherapy & surgery
Infections of the heart	Immunology and infection	Colon and rectum	1.13	0.77	1.67	0.525	Chemotherapy & surgery
Infections of the digestive system	Immunology and infection	Colon and rectum	0.91	0.80	1.02	0.103	Chemotherapy & surgery
Meningitis	Immunology and infection	Colon and rectum	1.04	0.70	1.54	0.85	Chemotherapy & surgery
Non-acute cystitis	Immunology and infection	Colon and rectum	1.19	0.71	1.99	0.513	Chemotherapy & surgery
Viral diseases (excluding chronic hepatitis/HIV)	Immunology and infection	Colon and rectum	0.71	0.47	1.09	0.118	Chemotherapy & surgery
Enteropathic arthropathy	Musculoskeletal	Colon and rectum	0.92	0.48	1.75	0.804	Chemotherapy & surgery
Enthesopathies and synovial disorders	Musculoskeletal	Colon and rectum	1.08	0.94	1.24	0.277	Chemotherapy & surgery
Osteoarthritis (excluding spine)	Musculoskeletal	Colon and rectum	1.10	0.98	1.24	0.115	Chemotherapy & surgery
Osteoporosis	Musculoskeletal	Colon and rectum	0.81	0.65	1.02	0.069	Chemotherapy & surgery
Rheumatoid arthritis	Musculoskeletal	Colon and rectum	0.64	0.41	1.02	0.06	Chemotherapy & surgery
Spinal stenosis	Musculoskeletal	Colon and rectum	1.22	0.79	1.89	0.372	Chemotherapy & surgery
Dementia	Neurological	Colon and rectum	0.27	0.15	0.47	< 0.001	Chemotherapy & surgery
Intracerebral haemorrhage	Neurological	Colon and rectum	0.33	0.18	0.62	0.001	Chemotherapy & surgery
Peripheral neuropathies	Neurological	Colon and rectum	0.70	0.53	0.91	0.007	Chemotherapy & surgery
Subarachnoid haemorrhage	Neurological	Colon and rectum	0.46	0.26	0.81	0.007	Chemotherapy & surgery
Disorders of autonomic nervous system	Neurological	Colon and rectum	0.84	0.44	1.58	0.585	Chemotherapy & surgery
Epilepsy	Neurological	Colon and rectum	0.70	0.42	1.17	0.17	Chemotherapy & surgery
Essential tremor	Neurological	Colon and rectum	1.38	0.70	2.71	0.354	Chemotherapy & surgery
Asthma	Pulmonary	Colon and rectum	0.79	0.65	0.96	0.019	Chemotherapy & surgery
Bronchiectasis	Pulmonary	Colon and rectum	0.63	0.34	1.18	0.146	Chemotherapy & surgery
COPD	Pulmonary	Colon and rectum	0.86	0.73	1.02	0.089	Chemotherapy & surgery
End stage renal disease	Renal	Colon and rectum	0.48	0.37	0.64	< 0.001	Chemotherapy & surgery
Glomerulonephritis	Renal	Colon and rectum	0.72	0.43	1.21	0.213	Chemotherapy & surgery
Obstructive and reflux uropathy	Renal	Colon and rectum	1.10	0.77	1.57	0.595	Chemotherapy & surgery
Urolithiasis	Renal	Colon and rectum	1.00	0.76	1.30	0.971	Chemotherapy & surgery
Atrial fibrillation	Cardiovascular	Colon and rectum	0.69	0.57	0.82	< 0.001	Chemotherapy alone
Atrioventricular block, complete	Cardiovascular	Colon and rectum	0.62	0.41	0.94	0.023	Chemotherapy alone
Atrioventricular block, first degree	Cardiovascular	Colon and rectum	0.62	0.41	0.94	0.023	Chemotherapy alone
Atrioventricular block, second degree	Cardiovascular	Colon and rectum	0.62	0.41	0.94	0.023	Chemotherapy alone
Coronary heart disease not otherwise specified	Cardiovascular	Colon and rectum	0.74	0.64	0.85	< 0.001	Chemotherapy alone
Heart failure	Cardiovascular	Colon and rectum	0.72	0.58	0.88	0.001	Chemotherapy alone
Ischaemic stroke	Cardiovascular	Colon and rectum	0.58	0.40	0.84	0.004	Chemotherapy alone
Left bundle branch block	Cardiovascular	Colon and rectum	0.62	0.42	0.92	0.019	Chemotherapy alone
Myocardial infarction	Cardiovascular	Colon and rectum	0.63	0.52	0.76	< 0.001	Chemotherapy alone
Nonrheumatic aortic valve disorders	Cardiovascular	Colon and rectum	0.59	0.37	0.93	0.023	Chemotherapy alone
Nonrheumatic mitral valve disorders	Cardiovascular	Colon and rectum	0.55	0.31	0.99	0.045	Chemotherapy alone
Other cardiomyopathy	Cardiovascular	Colon and rectum	0.64	0.53	0.77	< 0.001	Chemotherapy alone
Peripheral arterial disease	Cardiovascular	Colon and rectum	0.59	0.42	0.82	0.002	Chemotherapy alone
Stable angina	Cardiovascular	Colon and rectum	0.68	0.55	0.84	< 0.001	Chemotherapy alone

Stroke (not otherwise specified)	Cardiovascular	Colon and rectum	0.66	0.48	0.90	0.009	Chemotherapy alone
Unstable angina	Cardiovascular	Colon and rectum	0.66	0.54	0.82	< 0.001	Chemotherapy alone
Venous thromboembolic disease	Cardiovascular	Colon and rectum	0.76	0.60	0.97	0.027	Chemotherapy alone
Abdominal aortic aneurysm	Cardiovascular	Colon and rectum	1.50	1.18	1.91	0.001	Chemotherapy alone
Bifascicular block	Cardiovascular	Colon and rectum	0.82	0.51	1.33	0.422	Chemotherapy alone
Hypertension	Cardiovascular	Colon and rectum	0.93	0.83	1.03	0.161	Chemotherapy alone
Multiple valve disease	Cardiovascular	Colon and rectum	0.73	0.44	1.23	0.243	Chemotherapy alone
Raynaud's syndrome	Cardiovascular	Colon and rectum	0.75	0.52	1.07	0.114	Chemotherapy alone
Right bundle branch block	Cardiovascular	Colon and rectum	0.80	0.49	1.30	0.362	Chemotherapy alone
Sick sinus syndrome	Cardiovascular	Colon and rectum	0.57	0.31	1.07	0.079	Chemotherapy alone
Supraventricular tachycardia	Cardiovascular	Colon and rectum	0.76	0.45	1.29	0.318	Chemotherapy alone
Transient ischaemic attack	Cardiovascular	Colon and rectum	0.94	0.74	1.19	0.607	Chemotherapy alone
Trifascicular block	Cardiovascular	Colon and rectum	0.84	0.50	1.40	0.5	Chemotherapy alone
Ventricular tachycardia	Cardiovascular	Colon and rectum	0.77	0.46	1.31	0.337	Chemotherapy alone
Hypo or hyperthyroidism	Endocrine	Colon and rectum	0.76	0.58	0.98	0.038	Chemotherapy alone
Diabetes	Endocrine	Colon and rectum	0.99	0.86	1.14	0.909	Chemotherapy alone
Diabetic neurological complications	Endocrine	Colon and rectum	0.97	0.83	1.14	0.733	Chemotherapy alone
Diabetic ophthalmic complications	Endocrine	Colon and rectum	0.93	0.80	1.08	0.355	Chemotherapy alone
Crohn's disease	Gastrointestinal	Colon and rectum	0.46	0.25	0.85	0.013	Chemotherapy alone
Fatty liver	Gastrointestinal	Colon and rectum	1.29	1.00	1.67	0.048	Chemotherapy alone
Portal hypertension	Gastrointestinal	Colon and rectum	1.48	1.11	1.97	0.008	Chemotherapy alone
Barrett's oesophagus	Gastrointestinal	Colon and rectum	0.90	0.69	1.16	0.409	Chemotherapy alone
Cholangitis	Gastrointestinal	Colon and rectum	0.94	0.73	1.19	0.597	Chemotherapy alone
Cholecystitis	Gastrointestinal	Colon and rectum	1.02	0.81	1.28	0.863	Chemotherapy alone
Cholelithiasis	Gastrointestinal	Colon and rectum	0.90	0.71	1.13	0.343	Chemotherapy alone
Coeliac disease	Gastrointestinal	Colon and rectum	0.97	0.62	1.52	0.887	Chemotherapy alone
Diverticular disease of intestine	Gastrointestinal	Colon and rectum	0.99	0.87	1.12	0.858	Chemotherapy alone
Gastritis and duodenitis	Gastrointestinal	Colon and rectum	0.98	0.84	1.13	0.748	Chemotherapy alone
Gastrooesophageal reflux disease	Gastrointestinal	Colon and rectum	1.00	0.84	1.20	0.979	Chemotherapy alone
Hepatic failure	Gastrointestinal	Colon and rectum	1.18	0.90	1.54	0.23	Chemotherapy alone
Oesophagitis and oesophageal ulcer	Gastrointestinal	Colon and rectum	0.95	0.82	1.11	0.53	Chemotherapy alone
Ulcerative colitis	Gastrointestinal	Colon and rectum	1.04	0.61	1.77	0.896	Chemotherapy alone
Other anaemias	Haematological	Colon and rectum	0.85	0.76	0.96	0.007	Chemotherapy alone
Secondary or other thrombocytopaenia	Haematological	Colon and rectum	1.02	0.62	1.70	0.933	Chemotherapy alone
Infections of other or unspecified organs	Immunology and infection	Colon and rectum	0.72	0.61	0.86	< 0.001	Chemotherapy alone
Lower respiratory tract infections	Immunology and infection	Colon and rectum	0.77	0.66	0.90	0.001	Chemotherapy alone
Chronic viral hepatitis	Immunology and infection	Colon and rectum	1.27	0.80	2.02	0.307	Chemotherapy alone
Infection of skin and subcutaneous tissues	Immunology and infection	Colon and rectum	0.99	0.78	1.25	0.904	Chemotherapy alone
Infections of the heart	Immunology and infection	Colon and rectum	1.13	0.74	1.74	0.567	Chemotherapy alone
Infections of the digestive system	Immunology and infection	Colon and rectum	1.07	0.94	1.22	0.281	Chemotherapy alone
Meningitis	Immunology and infection	Colon and rectum	1.17	0.77	1.79	0.455	Chemotherapy alone
Non-acute cystitis	Immunology and infection	Colon and rectum	0.95	0.49	1.84	0.874	Chemotherapy alone
Peritonitis	Immunology and infection	Colon and rectum	0.97	0.88	1.08	0.614	Chemotherapy alone
Viral diseases (excluding chronic hepatitis/HIV)	Immunology and infection	Colon and rectum	0.92	0.60	1.41	0.706	Chemotherapy alone
Osteoporosis	Musculoskeletal	Colon and rectum	0.65	0.49	0.86	0.002	Chemotherapy alone
Rheumatoid arthritis	Musculoskeletal	Colon and rectum	0.57	0.34	0.97	0.038	Chemotherapy alone
Enthesopathies and synovial disorders	Musculoskeletal	Colon and rectum	0.93	0.79	1.10	0.408	Chemotherapy alone
Osteoarthritis (excluding spine)	Musculoskeletal	Colon and rectum	1.12	0.98	1.29	0.091	Chemotherapy alone
Spinal stenosis	Musculoskeletal	Colon and rectum	1.34	0.85	2.11	0.209	Chemotherapy alone
Dementia	Neurological	Colon and rectum	0.35	0.21	0.59	< 0.001	Chemotherapy alone
Disorders of autonomic nervous system	Neurological	Colon and rectum	0.77	0.39	1.53	0.461	Chemotherapy alone
Epilepsy	Neurological	Colon and rectum	0.53	0.28	1.01	0.055	Chemotherapy alone
Peripheral neuropathies	Neurological	Colon and rectum	0.76	0.57	1.01	0.063	Chemotherapy alone
Asthma	Pulmonary	Colon and rectum	0.87	0.70	1.08	0.193	Chemotherapy alone
Bronchiectasis	Pulmonary	Colon and rectum	1.46	0.89	2.39	0.134	Chemotherapy alone
COPD	Pulmonary	Colon and rectum	0.92	0.77	1.11	0.404	Chemotherapy alone
End stage renal disease	Renal	Colon and rectum	0.60	0.46	0.78	< 0.001	Chemotherapy alone
Obstructive and reflux uropathy	Renal	Colon and rectum	1.06	0.72	1.56	0.78	Chemotherapy alone
Urolithiasis	Renal	Colon and rectum	1.02	0.76	1.37	0.884	Chemotherapy alone
Atrial fibrillation	Cardiovascular	Colon and rectum	0.55	0.40	0.75	< 0.001	Chemotherapy, radiotherapy & surgery
Heart failure	Cardiovascular	Colon and rectum	0.42	0.28	0.64	< 0.001	Chemotherapy, radiotherapy & surgery
Myocardial infarction	Cardiovascular	Colon and rectum	0.62	0.46	0.84	0.002	Chemotherapy, radiotherapy & surgery
Other cardiomyopathy	Cardiovascular	Colon and rectum	0.66	0.50	0.88	0.005	Chemotherapy, radiotherapy & surgery
Peripheral arterial disease	Cardiovascular	Colon and rectum	0.57	0.34	0.96	0.035	Chemotherapy, radiotherapy & surgery
Stable angina	Cardiovascular	Colon and rectum	0.61	0.44	0.85	0.003	Chemotherapy, radiotherapy & surgery
Unstable angina	Cardiovascular	Colon and rectum	0.68	0.49	0.94	0.018	Chemotherapy, radiotherapy & surgery
Venous thromboembolic disease	Cardiovascular	Colon and rectum	0.55	0.35	0.86	0.009	Chemotherapy, radiotherapy & surgery
Abdominal aortic aneurysm	Cardiovascular	Colon and rectum	1.08	0.71	1.65	0.714	Chemotherapy, radiotherapy & surgery
Coronary heart disease not otherwise specified	Cardiovascular	Colon and rectum	0.89	0.73	1.08	0.223	Chemotherapy, radiotherapy & surgery
Hypertension	Cardiovascular	Colon and rectum	1.01	0.86	1.17	0.949	Chemotherapy, radiotherapy & surgery
Raynaud's syndrome	Cardiovascular	Colon and rectum	0.65	0.35	1.20	0.167	Chemotherapy, radiotherapy & surgery
Stroke (not otherwise specified)	Cardiovascular	Colon and rectum	0.74	0.47	1.17	0.199	Chemotherapy, radiotherapy & surgery
Transient ischaemic attack	Cardiovascular	Colon and rectum	0.70	0.46	1.08	0.108	Chemotherapy, radiotherapy & surgery
Diabetes	Endocrine	Colon and rectum	0.90	0.72	1.12	0.338	Chemotherapy, radiotherapy & surgery
Diabetic neurological complications	Endocrine	Colon and rectum	0.84	0.65	1.08	0.173	Chemotherapy, radiotherapy & surgery
Diabetic ophthalmic complications	Endocrine	Colon and rectum	0.82	0.64	1.05	0.118	Chemotherapy, radiotherapy & surgery
Hypo or hyperthyroidism	Endocrine	Colon and rectum	0.86	0.58	1.27	0.453	Chemotherapy, radiotherapy & surgery
Barrett's oesophagus	Gastrointestinal	Colon and rectum	0.59	0.36	0.97	0.036	Chemotherapy, radiotherapy & surgery
Gastritis and duodenitis	Gastrointestinal	Colon and rectum	0.62	0.47	0.81	0.001	Chemotherapy, radiotherapy & surgery

Oesophagitis and oesophageal ulcer	Gastrointestinal	Colon and rectum	0.67	0.52	0.87	0.003	Chemotherapy, radiotherapy & surgery
Cholangitis	Gastrointestinal	Colon and rectum	0.76	0.50	1.16	0.197	Chemotherapy, radiotherapy & surgery
Cholecystitis	Gastrointestinal	Colon and rectum	0.76	0.50	1.14	0.186	Chemotherapy, radiotherapy & surgery
Cholelithiasis	Gastrointestinal	Colon and rectum	0.80	0.54	1.17	0.249	Chemotherapy, radiotherapy & surgery
Coeliac disease	Gastrointestinal	Colon and rectum	1.53	0.86	2.75	0.151	Chemotherapy, radiotherapy & surgery
Diverticular disease of intestine	Gastrointestinal	Colon and rectum	0.83	0.68	1.01	0.062	Chemotherapy, radiotherapy & surgery
Fatty liver	Gastrointestinal	Colon and rectum	0.74	0.44	1.24	0.253	Chemotherapy, radiotherapy & surgery
Gastrooesophageal reflux disease	Gastrointestinal	Colon and rectum	0.77	0.56	1.05	0.097	Chemotherapy, radiotherapy & surgery
Hepatic failure	Gastrointestinal	Colon and rectum	0.68	0.39	1.19	0.178	Chemotherapy, radiotherapy & surgery
Portal hypertension	Gastrointestinal	Colon and rectum	0.75	0.40	1.39	0.361	Chemotherapy, radiotherapy & surgery
Other anaemias	Haematological	Colon and rectum	0.53	0.43	0.65	< 0.001	Chemotherapy, radiotherapy & surgery
Infections of other or unspecified organs	Immunology and infection	Colon and rectum	0.52	0.38	0.71	< 0.001	Chemotherapy, radiotherapy & surgery
Lower respiratory tract infections	Immunology and infection	Colon and rectum	0.50	0.37	0.67	< 0.001	Chemotherapy, radiotherapy & surgery
Peritonitis	Immunology and infection	Colon and rectum	0.74	0.63	0.88	< 0.001	Chemotherapy, radiotherapy & surgery
Infection of skin and subcutaneous tissues	Immunology and infection	Colon and rectum	0.67	0.43	1.04	0.075	Chemotherapy, radiotherapy & surgery
Infections of the digestive system	Immunology and infection	Colon and rectum	0.82	0.67	1.02	0.073	Chemotherapy, radiotherapy & surgery
Spinal stenosis	Musculoskeletal	Colon and rectum	2.49	1.41	4.38	0.002	Chemotherapy, radiotherapy & surgery
Enthesopathies and synovial disorders	Musculoskeletal	Colon and rectum	1.22	0.98	1.53	0.077	Chemotherapy, radiotherapy & surgery
Osteoarthritis (excluding spine)	Musculoskeletal	Colon and rectum	1.13	0.92	1.38	0.24	Chemotherapy, radiotherapy & surgery
Osteoporosis	Musculoskeletal	Colon and rectum	1.13	0.80	1.60	0.477	Chemotherapy, radiotherapy & surgery
Peripheral neuropathies	Neurological	Colon and rectum	0.68	0.42	1.09	0.111	Chemotherapy, radiotherapy & surgery
Asthma	Pulmonary	Colon and rectum	0.74	0.53	1.05	0.089	Chemotherapy, radiotherapy & surgery
COPD	Pulmonary	Colon and rectum	0.89	0.66	1.19	0.431	Chemotherapy, radiotherapy & surgery
End stage renal disease	Renal	Colon and rectum	0.47	0.28	0.80	0.005	Chemotherapy, radiotherapy & surgery
Obstructive and reflux uropathy	Renal	Colon and rectum	1.04	0.55	1.96	0.902	Chemotherapy, radiotherapy & surgery
Urolithiasis	Renal	Colon and rectum	1.10	0.71	1.68	0.677	Chemotherapy, radiotherapy & surgery
Atrial fibrillation	Cardiovascular	Colon and rectum	0.80	0.66	0.96	0.017	Radiotherapy & surgery
Abdominal aortic aneurysm	Cardiovascular	Colon and rectum	1.06	0.77	1.47	0.722	Radiotherapy & surgery
Atrioventricular block, complete	Cardiovascular	Colon and rectum	1.03	0.68	1.55	0.906	Radiotherapy & surgery
Atrioventricular block, first degree	Cardiovascular	Colon and rectum	1.03	0.68	1.55	0.906	Radiotherapy & surgery
Atrioventricular block, second degree	Cardiovascular	Colon and rectum	1.03	0.68	1.55	0.906	Radiotherapy & surgery
Bifascicular block	Cardiovascular	Colon and rectum	0.84	0.49	1.45	0.525	Radiotherapy & surgery
Coronary heart disease not otherwise specified	Cardiovascular	Colon and rectum	1.11	0.96	1.27	0.16	Radiotherapy & surgery
Heart failure	Cardiovascular	Colon and rectum	0.94	0.76	1.16	0.561	Radiotherapy & surgery
Hypertension	Cardiovascular	Colon and rectum	0.98	0.87	1.11	0.801	Radiotherapy & surgery
Ischaemic stroke	Cardiovascular	Colon and rectum	0.80	0.55	1.17	0.26	Radiotherapy & surgery
Left bundle branch block	Cardiovascular	Colon and rectum	1.04	0.70	1.55	0.843	Radiotherapy & surgery
Multiple valve disease	Cardiovascular	Colon and rectum	1.07	0.64	1.80	0.788	Radiotherapy & surgery
Myocardial infarction	Cardiovascular	Colon and rectum	0.82	0.68	1.00	0.055	Radiotherapy & surgery
Nonrheumatic aortic valve disorders	Cardiovascular	Colon and rectum	1.12	0.75	1.69	0.578	Radiotherapy & surgery
Nonrheumatic mitral valve disorders	Cardiovascular	Colon and rectum	0.84	0.48	1.45	0.532	Radiotherapy & surgery
Other cardiomyopathy	Cardiovascular	Colon and rectum	0.91	0.75	1.09	0.31	Radiotherapy & surgery
Peripheral arterial disease	Cardiovascular	Colon and rectum	0.94	0.69	1.28	0.706	Radiotherapy & surgery
Raynaud's syndrome	Cardiovascular	Colon and rectum	1.13	0.79	1.61	0.512	Radiotherapy & surgery
Right bundle branch block	Cardiovascular	Colon and rectum	0.79	0.45	1.39	0.413	Radiotherapy & surgery
Sick sinus syndrome	Cardiovascular	Colon and rectum	1.01	0.56	1.82	0.98	Radiotherapy & surgery
Stable angina	Cardiovascular	Colon and rectum	1.05	0.86	1.28	0.613	Radiotherapy & surgery
Stroke (not otherwise specified)	Cardiovascular	Colon and rectum	0.82	0.60	1.13	0.228	Radiotherapy & surgery
Supraventricular tachycardia	Cardiovascular	Colon and rectum	1.16	0.70	1.92	0.57	Radiotherapy & surgery
Transient ischaemic attack	Cardiovascular	Colon and rectum	1.07	0.82	1.39	0.613	Radiotherapy & surgery
Trifascicular block	Cardiovascular	Colon and rectum	0.74	0.40	1.38	0.345	Radiotherapy & surgery
Unstable angina	Cardiovascular	Colon and rectum	0.91	0.74	1.12	0.367	Radiotherapy & surgery
Venous thromboembolic disease	Cardiovascular	Colon and rectum	0.93	0.71	1.21	0.581	Radiotherapy & surgery
Ventricular tachycardia	Cardiovascular	Colon and rectum	1.35	0.84	2.16	0.211	Radiotherapy & surgery
Diabetes	Endocrine	Colon and rectum	1.13	0.96	1.33	0.13	Radiotherapy & surgery
Diabetic neurological complications	Endocrine	Colon and rectum	1.09	0.91	1.30	0.355	Radiotherapy & surgery
Diabetic ophthalmic complications	Endocrine	Colon and rectum	1.13	0.96	1.34	0.149	Radiotherapy & surgery
Hypo or hyperthyroidism	Endocrine	Colon and rectum	0.95	0.72	1.27	0.741	Radiotherapy & surgery
Diverticular disease of intestine	Gastrointestinal	Colon and rectum	0.82	0.70	0.95	0.009	Radiotherapy & surgery
Gastritis and duodenitis	Gastrointestinal	Colon and rectum	0.65	0.53	0.80	< 0.001	Radiotherapy & surgery
Barrett's oesophagus	Gastrointestinal	Colon and rectum	0.82	0.59	1.13	0.22	Radiotherapy & surgery
Cholangitis	Gastrointestinal	Colon and rectum	0.79	0.57	1.09	0.155	Radiotherapy & surgery
Cholecystitis	Gastrointestinal	Colon and rectum	0.84	0.62	1.14	0.271	Radiotherapy & surgery
Cholelithiasis	Gastrointestinal	Colon and rectum	0.84	0.62	1.12	0.229	Radiotherapy & surgery
Fatty liver	Gastrointestinal	Colon and rectum	1.00	0.68	1.47	0.983	Radiotherapy & surgery
Gastrooesophageal reflux disease	Gastrointestinal	Colon and rectum	0.87	0.68	1.10	0.234	Radiotherapy & surgery
Hepatic failure	Gastrointestinal	Colon and rectum	0.79	0.51	1.23	0.293	Radiotherapy & surgery
Oesophagitis and oesophageal ulcer	Gastrointestinal	Colon and rectum	0.84	0.69	1.01	0.063	Radiotherapy & surgery
Portal hypertension	Gastrointestinal	Colon and rectum	0.86	0.53	1.40	0.541	Radiotherapy & surgery
Other anaemias	Haematological	Colon and rectum	0.63	0.55	0.73	< 0.001	Radiotherapy & surgery
Infections of the digestive system	Immunology and infection	Colon and rectum	0.65	0.54	0.79	< 0.001	Radiotherapy & surgery
Lower respiratory tract infections	Immunology and infection	Colon and rectum	0.77	0.64	0.93	0.005	Radiotherapy & surgery
Peritonitis	Immunology and infection	Colon and rectum	0.77	0.68	0.87	< 0.001	Radiotherapy & surgery
Infection of skin and subcutaneous tissues	Immunology and infection	Colon and rectum	1.06	0.80	1.41	0.675	Radiotherapy & surgery
Infections of other or unspecified organs	Immunology and infection	Colon and rectum	0.85	0.69	1.03	0.092	Radiotherapy & surgery
Infections of the heart	Immunology and infection	Colon and rectum	1.07	0.63	1.84	0.792	Radiotherapy & surgery
Non-acute cystitis	Immunology and infection	Colon and rectum	1.73	0.99	3.02	0.053	Radiotherapy & surgery
Enthesopathies and synovial disorders	Musculoskeletal	Colon and rectum	0.98	0.80	1.19	0.816	Radiotherapy & surgery
Osteoarthritis (excluding spine)	Musculoskeletal	Colon and rectum	0.95	0.81	1.12	0.569	Radiotherapy & surgery
Osteoporosis	Musculoskeletal	Colon and rectum	0.94	0.72	1.24	0.678	Radiotherapy & surgery
Rheumatoid arthritis	Musculoskeletal	Colon and rectum	1.36	0.87	2.15	0.181	Radiotherapy & surgery

Dementia	Neurological	Colon and rectum	0.40	0.22	0.71	0.002	Radiotherapy & surgery
Intracerebral haemorrhage	Neurological	Colon and rectum	0.60	0.33	1.11	0.107	Radiotherapy & surgery
Peripheral neuropathies	Neurological	Colon and rectum	1.04	0.77	1.42	0.779	Radiotherapy & surgery
COPD	Pulmonary	Colon and rectum	1.29	1.07	1.56	0.008	Radiotherapy & surgery
Asthma	Pulmonary	Colon and rectum	1.06	0.84	1.34	0.621	Radiotherapy & surgery
End stage renal disease	Renal	Colon and rectum	0.90	0.67	1.20	0.469	Radiotherapy & surgery
Glomerulonephritis	Renal	Colon and rectum	1.47	0.88	2.45	0.143	Radiotherapy & surgery
Obstructive and reflux uropathy	Renal	Colon and rectum	1.01	0.60	1.70	0.971	Radiotherapy & surgery
Urolithiasis	Renal	Colon and rectum	1.11	0.78	1.57	0.569	Radiotherapy & surgery
Primary pulmonary hypertension	Cardiovascular	Colon and rectum	2.03	1.04	3.95	0.038	Radiotherapy alone
Ventricular tachycardia	Cardiovascular	Colon and rectum	1.59	1.00	2.53	0.049	Radiotherapy alone
Abdominal aortic aneurysm	Cardiovascular	Colon and rectum	0.81	0.56	1.17	0.268	Radiotherapy alone
Atrial fibrillation	Cardiovascular	Colon and rectum	0.84	0.69	1.01	0.07	Radiotherapy alone
Atrioventricular block, complete	Cardiovascular	Colon and rectum	0.72	0.46	1.14	0.163	Radiotherapy alone
Atrioventricular block, first degree	Cardiovascular	Colon and rectum	0.72	0.46	1.14	0.163	Radiotherapy alone
Atrioventricular block, second degree	Cardiovascular	Colon and rectum	0.72	0.46	1.14	0.163	Radiotherapy alone
Bifascicular block	Cardiovascular	Colon and rectum	1.11	0.69	1.80	0.663	Radiotherapy alone
Coronary heart disease not otherwise specified	Cardiovascular	Colon and rectum	0.94	0.80	1.11	0.469	Radiotherapy alone
Heart failure	Cardiovascular	Colon and rectum	0.86	0.69	1.07	0.182	Radiotherapy alone
Hypertension	Cardiovascular	Colon and rectum	1.05	0.92	1.19	0.459	Radiotherapy alone
Ischaemic stroke	Cardiovascular	Colon and rectum	0.80	0.55	1.16	0.241	Radiotherapy alone
Left bundle branch block	Cardiovascular	Colon and rectum	0.75	0.49	1.16	0.195	Radiotherapy alone
Multiple valve disease	Cardiovascular	Colon and rectum	1.21	0.75	1.96	0.436	Radiotherapy alone
Myocardial infarction	Cardiovascular	Colon and rectum	1.09	0.91	1.31	0.371	Radiotherapy alone
Nonrheumatic aortic valve disorders	Cardiovascular	Colon and rectum	1.05	0.69	1.60	0.825	Radiotherapy alone
Nonrheumatic mitral valve disorders	Cardiovascular	Colon and rectum	0.92	0.54	1.57	0.764	Radiotherapy alone
Other cardiomyopathy	Cardiovascular	Colon and rectum	1.06	0.89	1.27	0.511	Radiotherapy alone
Peripheral arterial disease	Cardiovascular	Colon and rectum	1.06	0.79	1.44	0.685	Radiotherapy alone
Raynaud's syndrome	Cardiovascular	Colon and rectum	1.27	0.90	1.80	0.176	Radiotherapy alone
Right bundle branch block	Cardiovascular	Colon and rectum	1.13	0.70	1.83	0.611	Radiotherapy alone
Sick sinus syndrome	Cardiovascular	Colon and rectum	1.29	0.77	2.15	0.336	Radiotherapy alone
Stable angina	Cardiovascular	Colon and rectum	0.86	0.69	1.07	0.183	Radiotherapy alone
Stroke (not otherwise specified)	Cardiovascular	Colon and rectum	1.00	0.74	1.35	0.998	Radiotherapy alone
Supraventricular tachycardia	Cardiovascular	Colon and rectum	1.45	0.89	2.35	0.133	Radiotherapy alone
Transient ischaemic attack	Cardiovascular	Colon and rectum	1.07	0.83	1.39	0.595	Radiotherapy alone
Trifascicular block	Cardiovascular	Colon and rectum	1.12	0.67	1.87	0.655	Radiotherapy alone
Unstable angina	Cardiovascular	Colon and rectum	0.88	0.71	1.10	0.258	Radiotherapy alone
Venous thromboembolic disease	Cardiovascular	Colon and rectum	0.86	0.65	1.13	0.282	Radiotherapy alone
Diabetes	Endocrine	Colon and rectum	1.03	0.86	1.23	0.73	Radiotherapy alone
Diabetic neurological complications	Endocrine	Colon and rectum	1.12	0.93	1.34	0.226	Radiotherapy alone
Diabetic ophthalmic complications	Endocrine	Colon and rectum	1.10	0.93	1.31	0.267	Radiotherapy alone
Hypo or hyperthyroidism	Endocrine	Colon and rectum	0.99	0.73	1.33	0.935	Radiotherapy alone
Diverticular disease of intestine	Gastrointestinal	Colon and rectum	0.62	0.52	0.74	< 0.001	Radiotherapy alone
Gastritis and duodenitis	Gastrointestinal	Colon and rectum	0.74	0.60	0.91	0.004	Radiotherapy alone
Cholangitis	Gastrointestinal	Colon and rectum	1.32	1.00	1.72	0.046	Radiotherapy alone
Barrett's oesophagus	Gastrointestinal	Colon and rectum	0.82	0.59	1.14	0.239	Radiotherapy alone
Cholecystitis	Gastrointestinal	Colon and rectum	1.19	0.91	1.57	0.2	Radiotherapy alone
Cholelithiasis	Gastrointestinal	Colon and rectum	1.06	0.81	1.39	0.672	Radiotherapy alone
Coeliac disease	Gastrointestinal	Colon and rectum	1.13	0.62	2.07	0.679	Radiotherapy alone
Fatty liver	Gastrointestinal	Colon and rectum	0.62	0.39	1.00	0.051	Radiotherapy alone
Gastrooesophageal reflux disease	Gastrointestinal	Colon and rectum	0.95	0.75	1.20	0.661	Radiotherapy alone
Hepatic failure	Gastrointestinal	Colon and rectum	0.71	0.45	1.13	0.148	Radiotherapy alone
Liver fibrosis sclerosis and cirrhosis	Gastrointestinal	Colon and rectum	1.43	0.75	2.73	0.272	Radiotherapy alone
Oesophagitis and oesophageal ulcer	Gastrointestinal	Colon and rectum	0.97	0.80	1.17	0.724	Radiotherapy alone
Other anaemias	Haematological	Colon and rectum	0.61	0.53	0.72	< 0.001	Radiotherapy alone
Infections of the digestive system	Immunology and infection	Colon and rectum	0.48	0.39	0.60	< 0.001	Radiotherapy alone
Peritonitis	Immunology and infection	Colon and rectum	0.50	0.43	0.58	< 0.001	Radiotherapy alone
Infection of bones and joints	Immunology and infection	Colon and rectum	1.80	1.03	3.15	0.038	Radiotherapy alone
Infection of skin and subcutaneous tissues	Immunology and infection	Colon and rectum	0.88	0.65	1.20	0.43	Radiotherapy alone
Infections of other or unspecified organs	Immunology and infection	Colon and rectum	0.83	0.68	1.02	0.077	Radiotherapy alone
Infections of the heart	Immunology and infection	Colon and rectum	0.77	0.41	1.43	0.411	Radiotherapy alone
Lower respiratory tract infections	Immunology and infection	Colon and rectum	0.89	0.75	1.06	0.208	Radiotherapy alone
Meningitis	Immunology and infection	Colon and rectum	1.03	0.60	1.76	0.913	Radiotherapy alone
Viral diseases (excluding chronic hepatitis/HIV)	Immunology and infection	Colon and rectum	1.26	0.78	2.04	0.353	Radiotherapy alone
Enthesopathies and synovial disorders	Musculoskeletal	Colon and rectum	1.27	1.05	1.54	0.016	Radiotherapy alone
Osteoarthritis (excluding spine)	Musculoskeletal	Colon and rectum	0.98	0.82	1.16	0.781	Radiotherapy alone
Osteoporosis	Musculoskeletal	Colon and rectum	0.95	0.71	1.26	0.717	Radiotherapy alone
Rheumatoid arthritis	Musculoskeletal	Colon and rectum	0.77	0.42	1.40	0.394	Radiotherapy alone
Spinal stenosis	Musculoskeletal	Colon and rectum	1.65	0.99	2.77	0.057	Radiotherapy alone
Dementia	Neurological	Colon and rectum	1.34	0.97	1.85	0.074	Radiotherapy alone
Intracerebral haemorrhage	Neurological	Colon and rectum	0.94	0.57	1.55	0.81	Radiotherapy alone
Peripheral neuropathies	Neurological	Colon and rectum	1.26	0.93	1.70	0.139	Radiotherapy alone
Subarachnoid haemorrhage	Neurological	Colon and rectum	0.79	0.44	1.39	0.41	Radiotherapy alone
Asthma	Pulmonary	Colon and rectum	0.89	0.68	1.17	0.416	Radiotherapy alone
COPD	Pulmonary	Colon and rectum	1.00	0.81	1.24	0.991	Radiotherapy alone
End stage renal disease	Renal	Colon and rectum	1.11	0.86	1.44	0.423	Radiotherapy alone
Glomerulonephritis	Renal	Colon and rectum	1.01	0.56	1.80	0.975	Radiotherapy alone
Obstructive and reflux uropathy	Renal	Colon and rectum	0.81	0.46	1.45	0.482	Radiotherapy alone
Urolithiasis	Renal	Colon and rectum	0.79	0.52	1.21	0.278	Radiotherapy alone

Hypertension	Cardiovascular	Gallbladder and biliary tract	0.54	0.31	0.96	0.037	Chemotherapy & surgery
Diabetes	Endocrine	Gallbladder and biliary tract	0.82	0.42	1.57	0.544	Chemotherapy & surgery
Diabetic neurological complications	Endocrine	Gallbladder and biliary tract	1.05	0.56	2.00	0.874	Chemotherapy & surgery
Diabetic ophthalmic complications	Endocrine	Gallbladder and biliary tract	0.92	0.48	1.76	0.799	Chemotherapy & surgery
Diverticular disease of intestine	Gastrointestinal	Gallbladder and biliary tract	1.96	1.00	3.82	0.049	Chemotherapy & surgery
Cholangitis	Gastrointestinal	Gallbladder and biliary tract	0.75	0.52	1.09	0.137	Chemotherapy & surgery
Cholecystitis	Gastrointestinal	Gallbladder and biliary tract	0.69	0.46	1.04	0.073	Chemotherapy & surgery
Cholelithiasis	Gastrointestinal	Gallbladder and biliary tract	0.73	0.49	1.10	0.133	Chemotherapy & surgery
Fatty liver	Gastrointestinal	Gallbladder and biliary tract	1.30	0.67	2.55	0.438	Chemotherapy & surgery
Gastritis and duodenitis	Gastrointestinal	Gallbladder and biliary tract	1.33	0.72	2.47	0.359	Chemotherapy & surgery
Gastrooesophageal reflux disease	Gastrointestinal	Gallbladder and biliary tract	1.51	0.79	2.89	0.214	Chemotherapy & surgery
Hepatic failure	Gastrointestinal	Gallbladder and biliary tract	1.57	0.81	3.04	0.182	Chemotherapy & surgery
Oesophagitis and oesophageal ulcer	Gastrointestinal	Gallbladder and biliary tract	0.89	0.46	1.74	0.737	Chemotherapy & surgery
Portal hypertension	Gastrointestinal	Gallbladder and biliary tract	1.47	0.69	3.15	0.323	Chemotherapy & surgery
Chronic viral hepatitis	Immunology and infection	Gallbladder and biliary tract	3.22	1.63	6.35	0.001	Chemotherapy & surgery
Infections of the digestive system	Immunology and infection	Gallbladder and biliary tract	1.35	0.64	2.84	0.434	Chemotherapy & surgery
Peritonitis	Immunology and infection	Gallbladder and biliary tract	1.26	0.74	2.15	0.393	Chemotherapy & surgery
Enthesopathies and synovial disorders	Musculoskeletal	Gallbladder and biliary tract	2.15	1.08	4.29	0.029	Chemotherapy & surgery
Coronary heart disease not otherwise specified	Cardiovascular	Gallbladder and biliary tract	0.66	0.32	1.33	0.241	Chemotherapy alone
Hypertension	Cardiovascular	Gallbladder and biliary tract	1.12	0.72	1.75	0.623	Chemotherapy alone
Myocardial infarction	Cardiovascular	Gallbladder and biliary tract	0.87	0.42	1.81	0.703	Chemotherapy alone
Other cardiomyopathy	Cardiovascular	Gallbladder and biliary tract	1.10	0.55	2.19	0.796	Chemotherapy alone
Diabetes	Endocrine	Gallbladder and biliary tract	1.22	0.71	2.09	0.479	Chemotherapy alone
Diabetic neurological complications	Endocrine	Gallbladder and biliary tract	1.58	0.92	2.70	0.096	Chemotherapy alone
Diabetic ophthalmic complications	Endocrine	Gallbladder and biliary tract	1.47	0.86	2.49	0.157	Chemotherapy alone
Cholangitis	Gastrointestinal	Gallbladder and biliary tract	0.66	0.47	0.94	0.023	Chemotherapy alone
Cholecystitis	Gastrointestinal	Gallbladder and biliary tract	0.43	0.28	0.64	< 0.001	Chemotherapy alone
Cholelithiasis	Gastrointestinal	Gallbladder and biliary tract	0.44	0.30	0.67	< 0.001	Chemotherapy alone
Diverticular disease of intestine	Gastrointestinal	Gallbladder and biliary tract	1.92	1.04	3.56	0.037	Chemotherapy alone
Gastrooesophageal reflux disease	Gastrointestinal	Gallbladder and biliary tract	1.95	1.08	3.52	0.028	Chemotherapy alone
Hepatic failure	Gastrointestinal	Gallbladder and biliary tract	1.94	1.05	3.60	0.034	Chemotherapy alone
Oesophagitis and oesophageal ulcer	Gastrointestinal	Gallbladder and biliary tract	1.75	1.02	3.02	0.043	Chemotherapy alone
Fatty liver	Gastrointestinal	Gallbladder and biliary tract	1.15	0.59	2.25	0.681	Chemotherapy alone
Gastritis and duodenitis	Gastrointestinal	Gallbladder and biliary tract	1.27	0.71	2.29	0.422	Chemotherapy alone
Portal hypertension	Gastrointestinal	Gallbladder and biliary tract	1.47	0.70	3.12	0.31	Chemotherapy alone
Other anaemias	Haematological	Gallbladder and biliary tract	0.82	0.42	1.60	0.564	Chemotherapy alone
Lower respiratory tract infections	Immunology and infection	Gallbladder and biliary tract	0.42	0.22	0.82	0.01	Chemotherapy alone
Infections of other or unspecified organs	Immunology and infection	Gallbladder and biliary tract	0.66	0.33	1.32	0.239	Chemotherapy alone
Peritonitis	Immunology and infection	Gallbladder and biliary tract	1.04	0.62	1.75	0.881	Chemotherapy alone
Enthesopathies and synovial disorders	Musculoskeletal	Gallbladder and biliary tract	1.31	0.62	2.74	0.481	Chemotherapy alone
Osteoarthritis (excluding spine)	Musculoskeletal	Gallbladder and biliary tract	1.15	0.60	2.19	0.679	Chemotherapy alone
Cholangitis	Gastrointestinal	Gallbladder and biliary tract	1.48	0.59	3.71	0.401	Radiotherapy & surgery
Coronary heart disease not otherwise specified	Cardiovascular	Kidney and renal pelvis	1.15	0.67	1.97	0.616	Chemotherapy & radiotherapy
Hypertension	Cardiovascular	Kidney and renal pelvis	0.79	0.51	1.21	0.269	Chemotherapy & radiotherapy
Diabetes	Endocrine	Kidney and renal pelvis	0.85	0.47	1.53	0.581	Chemotherapy & radiotherapy
Diabetic neurological complications	Endocrine	Kidney and renal pelvis	1.02	0.56	1.86	0.946	Chemotherapy & radiotherapy
Diabetic ophthalmic complications	Endocrine	Kidney and renal pelvis	1.31	0.77	2.25	0.32	Chemotherapy & radiotherapy
Other anaemias	Haematological	Kidney and renal pelvis	1.19	0.69	2.06	0.53	Chemotherapy & radiotherapy
Infection of bones and joints	Immunology and infection	Kidney and renal pelvis	8.84	3.65	21.40	< 0.001	Chemotherapy & radiotherapy
Enthesopathies and synovial disorders	Musculoskeletal	Kidney and renal pelvis	1.23	0.66	2.31	0.518	Chemotherapy & radiotherapy
Osteoarthritis (excluding spine)	Musculoskeletal	Kidney and renal pelvis	1.44	0.82	2.52	0.203	Chemotherapy & radiotherapy
Atrial fibrillation	Cardiovascular	Kidney and renal pelvis	0.98	0.59	1.63	0.93	Chemotherapy & surgery
Coronary heart disease not otherwise specified	Cardiovascular	Kidney and renal pelvis	0.78	0.51	1.18	0.232	Chemotherapy & surgery
Hypertension	Cardiovascular	Kidney and renal pelvis	1.03	0.79	1.35	0.81	Chemotherapy & surgery
Myocardial infarction	Cardiovascular	Kidney and renal pelvis	0.74	0.44	1.24	0.249	Chemotherapy & surgery
Other cardiomyopathy	Cardiovascular	Kidney and renal pelvis	0.61	0.37	1.02	0.058	Chemotherapy & surgery
Venous thromboembolic disease	Cardiovascular	Kidney and renal pelvis	1.59	0.96	2.64	0.071	Chemotherapy & surgery
Hypo or hyperthyroidism	Endocrine	Kidney and renal pelvis	1.85	1.06	3.23	0.031	Chemotherapy & surgery
Diabetes	Endocrine	Kidney and renal pelvis	0.85	0.56	1.29	0.448	Chemotherapy & surgery
Diabetic neurological complications	Endocrine	Kidney and renal pelvis	0.79	0.51	1.21	0.277	Chemotherapy & surgery
Diabetic ophthalmic complications	Endocrine	Kidney and renal pelvis	0.76	0.50	1.17	0.217	Chemotherapy & surgery
Cholelithiasis	Gastrointestinal	Kidney and renal pelvis	1.09	0.59	2.02	0.771	Chemotherapy & surgery
Diverticular disease of intestine	Gastrointestinal	Kidney and renal pelvis	0.93	0.58	1.50	0.766	Chemotherapy & surgery
Gastritis and duodenitis	Gastrointestinal	Kidney and renal pelvis	0.58	0.31	1.09	0.09	Chemotherapy & surgery
Gastrooesophageal reflux disease	Gastrointestinal	Kidney and renal pelvis	1.00	0.59	1.67	0.986	Chemotherapy & surgery
Oesophagitis and oesophageal ulcer	Gastrointestinal	Kidney and renal pelvis	0.70	0.42	1.16	0.165	Chemotherapy & surgery
Other anaemias	Haematological	Kidney and renal pelvis	1.36	0.95	1.95	0.094	Chemotherapy & surgery
Infections of other or unspecified organs	Immunology and infection	Kidney and renal pelvis	0.74	0.43	1.28	0.286	Chemotherapy & surgery
Infections of the digestive system	Immunology and infection	Kidney and renal pelvis	1.49	0.87	2.56	0.147	Chemotherapy & surgery
Lower respiratory tract infections	Immunology and infection	Kidney and renal pelvis	0.77	0.49	1.21	0.256	Chemotherapy & surgery
Peritonitis	Immunology and infection	Kidney and renal pelvis	0.91	0.60	1.40	0.678	Chemotherapy & surgery
Osteoporosis	Musculoskeletal	Kidney and renal pelvis	1.89	1.04	3.46	0.038	Chemotherapy & surgery
Enthesopathies and synovial disorders	Musculoskeletal	Kidney and renal pelvis	1.03	0.67	1.58	0.905	Chemotherapy & surgery
Osteoarthritis (excluding spine)	Musculoskeletal	Kidney and renal pelvis	0.94	0.61	1.45	0.775	Chemotherapy & surgery
COPD	Pulmonary	Kidney and renal pelvis	1.08	0.64	1.82	0.772	Chemotherapy & surgery
End stage renal disease	Renal	Kidney and renal pelvis	1.01	0.58	1.76	0.98	Chemotherapy & surgery
Obstructive and reflux uropathy	Renal	Kidney and renal pelvis	1.42	0.84	2.40	0.195	Chemotherapy & surgery
Urolithiasis	Renal	Kidney and renal pelvis	1.17	0.78	1.77	0.448	Chemotherapy & surgery
Atrial fibrillation	Cardiovascular	Kidney and renal pelvis	0.56	0.33	0.96	0.036	Chemotherapy alone
Abdominal aortic aneurysm	Cardiovascular	Kidney and renal pelvis	1.15	0.63	2.11	0.655	Chemotherapy alone
Coronary heart disease not otherwise specified	Cardiovascular	Kidney and renal pelvis	0.96	0.67	1.38	0.823	Chemotherapy alone

Heart failure	Cardiovascular	Kidney and renal pelvis	0.91	0.55	1.52	0.721	Chemotherapy alone
Hypertension	Cardiovascular	Kidney and renal pelvis	0.95	0.72	1.24	0.685	Chemotherapy alone
Myocardial infarction	Cardiovascular	Kidney and renal pelvis	0.85	0.55	1.32	0.468	Chemotherapy alone
Other cardiomyopathy	Cardiovascular	Kidney and renal pelvis	0.86	0.57	1.29	0.467	Chemotherapy alone
Stable angina	Cardiovascular	Kidney and renal pelvis	0.67	0.39	1.16	0.156	Chemotherapy alone
Transient ischaemic attack	Cardiovascular	Kidney and renal pelvis	1.42	0.74	2.75	0.296	Chemotherapy alone
Unstable angina	Cardiovascular	Kidney and renal pelvis	0.68	0.40	1.16	0.153	Chemotherapy alone
Venous thromboembolic disease	Cardiovascular	Kidney and renal pelvis	0.97	0.56	1.67	0.907	Chemotherapy alone
Diabetes	Endocrine	Kidney and renal pelvis	0.99	0.69	1.42	0.935	Chemotherapy alone
Diabetic neurological complications	Endocrine	Kidney and renal pelvis	0.74	0.49	1.11	0.147	Chemotherapy alone
Diabetic ophthalmic complications	Endocrine	Kidney and renal pelvis	0.81	0.55	1.18	0.278	Chemotherapy alone
Hypo or hyperthyroidism	Endocrine	Kidney and renal pelvis	0.80	0.40	1.60	0.534	Chemotherapy alone
Diverticular disease of intestine	Gastrointestinal	Kidney and renal pelvis	0.37	0.21	0.67	0.001	Chemotherapy alone
Gastritis and duodenitis	Gastrointestinal	Kidney and renal pelvis	1.11	0.69	1.77	0.677	Chemotherapy alone
Gastroesophageal reflux disease	Gastrointestinal	Kidney and renal pelvis	1.37	0.87	2.18	0.177	Chemotherapy alone
Oesophagitis and oesophageal ulcer	Gastrointestinal	Kidney and renal pelvis	1.20	0.80	1.81	0.372	Chemotherapy alone
Other anaemias	Haematological	Kidney and renal pelvis	1.53	1.10	2.15	0.012	Chemotherapy alone
Peritonitis	Immunology and infection	Kidney and renal pelvis	0.45	0.28	0.72	0.001	Chemotherapy alone
Infection of skin and subcutaneous tissues	Immunology and infection	Kidney and renal pelvis	0.88	0.44	1.78	0.721	Chemotherapy alone
Infections of other or unspecified organs	Immunology and infection	Kidney and renal pelvis	0.89	0.55	1.43	0.63	Chemotherapy alone
Infections of the digestive system	Immunology and infection	Kidney and renal pelvis	0.74	0.40	1.36	0.33	Chemotherapy alone
Lower respiratory tract infections	Immunology and infection	Kidney and renal pelvis	1.03	0.69	1.53	0.889	Chemotherapy alone
Enthesopathies and synovial disorders	Musculoskeletal	Kidney and renal pelvis	0.88	0.56	1.39	0.581	Chemotherapy alone
Osteoarthritis (excluding spine)	Musculoskeletal	Kidney and renal pelvis	1.00	0.67	1.50	0.987	Chemotherapy alone
Asthma	Pulmonary	Kidney and renal pelvis	0.74	0.39	1.42	0.369	Chemotherapy alone
COPD	Pulmonary	Kidney and renal pelvis	0.95	0.55	1.63	0.853	Chemotherapy alone
End stage renal disease	Renal	Kidney and renal pelvis	0.67	0.39	1.16	0.154	Chemotherapy alone
Obstructive and reflux uropathy	Renal	Kidney and renal pelvis	1.05	0.58	1.91	0.875	Chemotherapy alone
Urolithiasis	Renal	Kidney and renal pelvis	1.26	0.82	1.94	0.287	Chemotherapy alone
Coronary heart disease not otherwise specified	Cardiovascular	Kidney and renal pelvis	0.62	0.33	1.16	0.134	Chemotherapy, radiotherapy & surgery
Hypertension	Cardiovascular	Kidney and renal pelvis	1.03	0.72	1.48	0.877	Chemotherapy, radiotherapy & surgery
Diabetes	Endocrine	Kidney and renal pelvis	0.96	0.56	1.64	0.885	Chemotherapy, radiotherapy & surgery
Diabetic neurological complications	Endocrine	Kidney and renal pelvis	1.13	0.66	1.94	0.657	Chemotherapy, radiotherapy & surgery
Diabetic ophthalmic complications	Endocrine	Kidney and renal pelvis	1.21	0.72	2.02	0.478	Chemotherapy, radiotherapy & surgery
Diverticular disease of intestine	Gastrointestinal	Kidney and renal pelvis	1.23	0.69	2.21	0.48	Chemotherapy, radiotherapy & surgery
Oesophagitis and oesophageal ulcer	Gastrointestinal	Kidney and renal pelvis	0.91	0.48	1.71	0.76	Chemotherapy, radiotherapy & surgery
Other anaemias	Haematological	Kidney and renal pelvis	1.14	0.68	1.91	0.62	Chemotherapy, radiotherapy & surgery
Infections of other or unspecified organs	Immunology and infection	Kidney and renal pelvis	2.50	1.49	4.21	0.001	Chemotherapy, radiotherapy & surgery
Lower respiratory tract infections	Immunology and infection	Kidney and renal pelvis	1.31	0.78	2.21	0.311	Chemotherapy, radiotherapy & surgery
Peritonitis	Immunology and infection	Kidney and renal pelvis	1.06	0.61	1.84	0.836	Chemotherapy, radiotherapy & surgery
Enthesopathies and synovial disorders	Musculoskeletal	Kidney and renal pelvis	1.05	0.60	1.86	0.858	Chemotherapy, radiotherapy & surgery
Osteoarthritis (excluding spine)	Musculoskeletal	Kidney and renal pelvis	1.41	0.85	2.35	0.186	Chemotherapy, radiotherapy & surgery
Urolithiasis	Renal	Kidney and renal pelvis	1.23	0.70	2.14	0.47	Chemotherapy, radiotherapy & surgery
Myocardial infarction	Cardiovascular	Kidney and renal pelvis	1.48	1.03	2.12	0.034	Radiotherapy & surgery
Atrial fibrillation	Cardiovascular	Kidney and renal pelvis	0.97	0.63	1.50	0.9	Radiotherapy & surgery
Coronary heart disease not otherwise specified	Cardiovascular	Kidney and renal pelvis	1.33	0.98	1.79	0.064	Radiotherapy & surgery
Hypertension	Cardiovascular	Kidney and renal pelvis	1.20	0.96	1.51	0.109	Radiotherapy & surgery
Other cardiomyopathy	Cardiovascular	Kidney and renal pelvis	1.19	0.83	1.70	0.338	Radiotherapy & surgery
Stable angina	Cardiovascular	Kidney and renal pelvis	1.22	0.81	1.85	0.332	Radiotherapy & surgery
Unstable angina	Cardiovascular	Kidney and renal pelvis	0.91	0.58	1.41	0.664	Radiotherapy & surgery
Venous thromboembolic disease	Cardiovascular	Kidney and renal pelvis	1.40	0.88	2.22	0.15	Radiotherapy & surgery
Diabetes	Endocrine	Kidney and renal pelvis	1.04	0.74	1.45	0.829	Radiotherapy & surgery
Diabetic neurological complications	Endocrine	Kidney and renal pelvis	1.11	0.79	1.55	0.556	Radiotherapy & surgery
Diabetic ophthalmic complications	Endocrine	Kidney and renal pelvis	1.17	0.84	1.61	0.357	Radiotherapy & surgery
Hypo or hyperthyroidism	Endocrine	Kidney and renal pelvis	0.97	0.53	1.80	0.931	Radiotherapy & surgery
Cholangitis	Gastrointestinal	Kidney and renal pelvis	1.06	0.61	1.85	0.835	Radiotherapy & surgery
Cholecystitis	Gastrointestinal	Kidney and renal pelvis	1.26	0.76	2.09	0.367	Radiotherapy & surgery
Cholelithiasis	Gastrointestinal	Kidney and renal pelvis	1.18	0.72	1.95	0.506	Radiotherapy & surgery
Diverticular disease of intestine	Gastrointestinal	Kidney and renal pelvis	1.11	0.75	1.65	0.593	Radiotherapy & surgery
Gastritis and duodenitis	Gastrointestinal	Kidney and renal pelvis	0.89	0.57	1.40	0.629	Radiotherapy & surgery
Gastrooesophageal reflux disease	Gastrointestinal	Kidney and renal pelvis	0.90	0.56	1.45	0.672	Radiotherapy & surgery
Oesophagitis and oesophageal ulcer	Gastrointestinal	Kidney and renal pelvis	0.72	0.46	1.11	0.136	Radiotherapy & surgery
Other anaemias	Haematological	Kidney and renal pelvis	1.15	0.83	1.60	0.39	Radiotherapy & surgery
Infection of skin and subcutaneous tissues	Immunology and infection	Kidney and renal pelvis	1.75	1.04	2.94	0.036	Radiotherapy & surgery
Infections of other or unspecified organs	Immunology and infection	Kidney and renal pelvis	0.71	0.44	1.14	0.157	Radiotherapy & surgery
Infections of the digestive system	Immunology and infection	Kidney and renal pelvis	1.03	0.60	1.77	0.914	Radiotherapy & surgery
Lower respiratory tract infections	Immunology and infection	Kidney and renal pelvis	0.83	0.57	1.21	0.336	Radiotherapy & surgery
Non-acute cystitis	Immunology and infection	Kidney and renal pelvis	1.60	0.81	3.17	0.18	Radiotherapy & surgery
Peritonitis	Immunology and infection	Kidney and renal pelvis	1.09	0.77	1.55	0.615	Radiotherapy & surgery
Enthesopathies and synovial disorders	Musculoskeletal	Kidney and renal pelvis	0.98	0.67	1.43	0.914	Radiotherapy & surgery
Osteoarthritis (excluding spine)	Musculoskeletal	Kidney and renal pelvis	0.84	0.58	1.22	0.363	Radiotherapy & surgery
Osteoporosis	Musculoskeletal	Kidney and renal pelvis	1.16	0.62	2.14	0.644	Radiotherapy & surgery
Asthma	Pulmonary	Kidney and renal pelvis	0.73	0.42	1.28	0.272	Radiotherapy & surgery
COPD	Pulmonary	Kidney and renal pelvis	0.79	0.48	1.31	0.359	Radiotherapy & surgery
End stage renal disease	Renal	Kidney and renal pelvis	0.88	0.53	1.47	0.636	Radiotherapy & surgery
Glomerulonephritis	Renal	Kidney and renal pelvis	1.56	0.88	2.76	0.13	Radiotherapy & surgery
Obstructive and reflux uropathy	Renal	Kidney and renal pelvis	0.92	0.54	1.56	0.764	Radiotherapy & surgery
Urolithiasis	Renal	Kidney and renal pelvis	0.89	0.60	1.32	0.554	Radiotherapy & surgery
Abdominal aortic aneurysm	Cardiovascular	Kidney and renal pelvis	1.13	0.59	2.15	0.717	Radiotherapy alone

Atrial fibrillation	Cardiovascular	Kidney and renal pelvis	1.30	0.86	1.96	0.211	Radiotherapy alone
Coronary heart disease not otherwise specified	Cardiovascular	Kidney and renal pelvis	1.25	0.90	1.74	0.186	Radiotherapy alone
Heart failure	Cardiovascular	Kidney and renal pelvis	0.98	0.59	1.61	0.93	Radiotherapy alone
Hypertension	Cardiovascular	Kidney and renal pelvis	0.96	0.73	1.26	0.77	Radiotherapy alone
Myocardial infarction	Cardiovascular	Kidney and renal pelvis	1.10	0.73	1.67	0.653	Radiotherapy alone
Other cardiomyopathy	Cardiovascular	Kidney and renal pelvis	1.01	0.68	1.51	0.957	Radiotherapy alone
Stable angina	Cardiovascular	Kidney and renal pelvis	1.00	0.63	1.60	0.994	Radiotherapy alone
Stroke (not otherwise specified)	Cardiovascular	Kidney and renal pelvis	1.75	0.91	3.37	0.092	Radiotherapy alone
Transient ischaemic attack	Cardiovascular	Kidney and renal pelvis	1.79	0.96	3.33	0.068	Radiotherapy alone
Unstable angina	Cardiovascular	Kidney and renal pelvis	0.94	0.59	1.51	0.813	Radiotherapy alone
Venous thromboembolic disease	Cardiovascular	Kidney and renal pelvis	1.44	0.89	2.33	0.14	Radiotherapy alone
Diabetes	Endocrine	Kidney and renal pelvis	0.89	0.61	1.29	0.539	Radiotherapy alone
Diabetic neurological complications	Endocrine	Kidney and renal pelvis	0.73	0.49	1.11	0.141	Radiotherapy alone
Diabetic ophthalmic complications	Endocrine	Kidney and renal pelvis	0.78	0.53	1.15	0.219	Radiotherapy alone
Hypo or hyperthyroidism	Endocrine	Kidney and renal pelvis	0.69	0.34	1.40	0.299	Radiotherapy alone
Diverticular disease of intestine	Gastrointestinal	Kidney and renal pelvis	0.44	0.25	0.78	0.005	Radiotherapy alone
Cholangitis	Gastrointestinal	Kidney and renal pelvis	1.10	0.55	2.19	0.783	Radiotherapy alone
Cholecystitis	Gastrointestinal	Kidney and renal pelvis	0.90	0.45	1.78	0.753	Radiotherapy alone
Cholelithiasis	Gastrointestinal	Kidney and renal pelvis	0.95	0.48	1.84	0.869	Radiotherapy alone
Gastritis and duodenitis	Gastrointestinal	Kidney and renal pelvis	1.32	0.85	2.06	0.217	Radiotherapy alone
Gastrooesophageal reflux disease	Gastrointestinal	Kidney and renal pelvis	1.22	0.74	2.01	0.429	Radiotherapy alone
Oesophagitis and oesophageal ulcer	Gastrointestinal	Kidney and renal pelvis	1.24	0.83	1.87	0.292	Radiotherapy alone
Other anaemias	Haematological	Kidney and renal pelvis	1.10	0.77	1.59	0.597	Radiotherapy alone
Peritonitis	Immunology and infection	Kidney and renal pelvis	0.63	0.40	0.99	0.046	Radiotherapy alone
Infection of bones and joints	Immunology and infection	Kidney and renal pelvis	8.95	4.50	17.82	< 0.001	Radiotherapy alone
Infection of skin and subcutaneous tissues	Immunology and infection	Kidney and renal pelvis	0.97	0.49	1.94	0.942	Radiotherapy alone
Infections of other or unspecified organs	Immunology and infection	Kidney and renal pelvis	0.99	0.62	1.59	0.969	Radiotherapy alone
Infections of the digestive system	Immunology and infection	Kidney and renal pelvis	0.64	0.33	1.26	0.199	Radiotherapy alone
Lower respiratory tract infections	Immunology and infection	Kidney and renal pelvis	0.99	0.67	1.47	0.962	Radiotherapy alone
Enthesopathies and synovial disorders	Musculoskeletal	Kidney and renal pelvis	1.40	0.92	2.12	0.117	Radiotherapy alone
Osteoarthritis (excluding spine)	Musculoskeletal	Kidney and renal pelvis	1.11	0.77	1.62	0.57	Radiotherapy alone
Peripheral neuropathies	Neurological	Kidney and renal pelvis	1.31	0.67	2.56	0.434	Radiotherapy alone
Asthma	Pulmonary	Kidney and renal pelvis	1.51	0.92	2.47	0.1	Radiotherapy alone
COPD	Pulmonary	Kidney and renal pelvis	1.01	0.59	1.73	0.966	Radiotherapy alone
End stage renal disease	Renal	Kidney and renal pelvis	0.83	0.49	1.40	0.491	Radiotherapy alone
Obstructive and reflux uropathy	Renal	Kidney and renal pelvis	1.03	0.54	1.95	0.934	Radiotherapy alone
Urolithiasis	Renal	Kidney and renal pelvis	0.70	0.41	1.22	0.212	Radiotherapy alone
Hypertension	Cardiovascular	Liver and intrahepatic bile duct	0.79	0.47	1.32	0.364	Chemotherapy & surgery
Diabetes	Endocrine	Liver and intrahepatic bile duct	0.86	0.46	1.59	0.626	Chemotherapy & surgery
Diabetic neurological complications	Endocrine	Liver and intrahepatic bile duct	1.01	0.58	1.76	0.981	Chemotherapy & surgery
Diabetic ophthalmic complications	Endocrine	Liver and intrahepatic bile duct	1.06	0.61	1.84	0.825	Chemotherapy & surgery
Cholecystitis	Gastrointestinal	Liver and intrahepatic bile duct	0.51	0.27	0.96	0.037	Chemotherapy & surgery
Cholelithiasis	Gastrointestinal	Liver and intrahepatic bile duct	0.46	0.24	0.88	0.019	Chemotherapy & surgery
Alcoholic liver disease	Gastrointestinal	Liver and intrahepatic bile duct	0.70	0.35	1.38	0.298	Chemotherapy & surgery
Cholangitis	Gastrointestinal	Liver and intrahepatic bile duct	0.79	0.51	1.22	0.285	Chemotherapy & surgery
Diverticular disease of intestine	Gastrointestinal	Liver and intrahepatic bile duct	1.46	0.74	2.89	0.275	Chemotherapy & surgery
Fatty liver	Gastrointestinal	Liver and intrahepatic bile duct	0.76	0.46	1.25	0.278	Chemotherapy & surgery
Gastritis and duodenitis	Gastrointestinal	Liver and intrahepatic bile duct	0.73	0.41	1.31	0.291	Chemotherapy & surgery
Hepatic failure	Gastrointestinal	Liver and intrahepatic bile duct	0.75	0.46	1.22	0.253	Chemotherapy & surgery
Liver fibrosis sclerosis and cirrhosis	Gastrointestinal	Liver and intrahepatic bile duct	0.94	0.56	1.58	0.813	Chemotherapy & surgery
Oesophageal varices	Gastrointestinal	Liver and intrahepatic bile duct	0.72	0.36	1.42	0.342	Chemotherapy & surgery
Oesophagitis and oesophageal ulcer	Gastrointestinal	Liver and intrahepatic bile duct	0.96	0.49	1.89	0.902	Chemotherapy & surgery
Portal hypertension	Gastrointestinal	Liver and intrahepatic bile duct	0.85	0.51	1.43	0.538	Chemotherapy & surgery
Other anaemias	Haematological	Liver and intrahepatic bile duct	1.07	0.55	2.08	0.84	Chemotherapy & surgery
Autoimmune liver disease	Immunology and infection	Liver and intrahepatic bile duct	1.07	0.62	1.85	0.816	Chemotherapy & surgery
Chronic viral hepatitis	Immunology and infection	Liver and intrahepatic bile duct	0.75	0.40	1.40	0.366	Chemotherapy & surgery
Lower respiratory tract infections	Immunology and infection	Liver and intrahepatic bile duct	0.69	0.37	1.31	0.261	Chemotherapy & surgery
Peritonitis	Immunology and infection	Liver and intrahepatic bile duct	0.74	0.41	1.31	0.298	Chemotherapy & surgery
Enthesopathies and synovial disorders	Musculoskeletal	Liver and intrahepatic bile duct	2.40	1.24	4.64	0.009	Chemotherapy & surgery
Venous thromboembolic disease	Cardiovascular	Liver and intrahepatic bile duct	0.45	0.28	0.72	0.001	Chemotherapy alone
Heart failure	Cardiovascular	Liver and intrahepatic bile duct	0.41	0.22	0.76	0.005	Chemotherapy alone
Other cardiomyopathy	Cardiovascular	Liver and intrahepatic bile duct	0.59	0.37	0.94	0.028	Chemotherapy alone
Stable angina	Cardiovascular	Liver and intrahepatic bile duct	0.54	0.29	0.98	0.044	Chemotherapy alone
Abdominal aortic aneurysm	Cardiovascular	Liver and intrahepatic bile duct	1.24	0.55	2.78	0.601	Chemotherapy alone
Atrial fibrillation	Cardiovascular	Liver and intrahepatic bile duct	0.73	0.45	1.18	0.197	Chemotherapy alone
Coronary heart disease not otherwise specified	Cardiovascular	Liver and intrahepatic bile duct	0.79	0.51	1.22	0.29	Chemotherapy alone
Hypertension	Cardiovascular	Liver and intrahepatic bile duct	0.78	0.57	1.07	0.126	Chemotherapy alone
Myocardial infarction	Cardiovascular	Liver and intrahepatic bile duct	0.69	0.43	1.08	0.107	Chemotherapy alone
Peripheral arterial disease	Cardiovascular	Liver and intrahepatic bile duct	1.35	0.64	2.86	0.426	Chemotherapy alone
Raynaud's syndrome	Cardiovascular	Liver and intrahepatic bile duct	1.30	0.55	3.10	0.551	Chemotherapy alone
Transient ischaemic attack	Cardiovascular	Liver and intrahepatic bile duct	1.09	0.53	2.26	0.807	Chemotherapy alone
Unstable angina	Cardiovascular	Liver and intrahepatic bile duct	0.61	0.36	1.05	0.075	Chemotherapy alone
Diabetes	Endocrine	Liver and intrahepatic bile duct	0.94	0.65	1.34	0.727	Chemotherapy alone
Diabetic neurological complications	Endocrine	Liver and intrahepatic bile duct	0.98	0.71	1.35	0.897	Chemotherapy alone
Diabetic ophthalmic complications	Endocrine	Liver and intrahepatic bile duct	1.03	0.75	1.42	0.858	Chemotherapy alone
Hypo or hyperthyroidism	Endocrine	Liver and intrahepatic bile duct	1.44	0.77	2.67	0.252	Chemotherapy alone
Cholangitis	Gastrointestinal	Liver and intrahepatic bile duct	0.38	0.28	0.52	< 0.001	Chemotherapy alone
Cholecystitis	Gastrointestinal	Liver and intrahepatic bile duct	0.40	0.27	0.60	< 0.001	Chemotherapy alone
Cholelithiasis	Gastrointestinal	Liver and intrahepatic bile duct	0.50	0.35	0.72	< 0.001	Chemotherapy alone
Hepatic failure	Gastrointestinal	Liver and intrahepatic bile duct	0.73	0.54	0.98	0.036	Chemotherapy alone
Alcoholic liver disease	Gastrointestinal	Liver and intrahepatic bile duct	0.74	0.49	1.10	0.136	Chemotherapy alone

Barrett's oesophagus	Gastrointestinal	Liver and intrahepatic bile duct	0.71	0.36	1.40	0.322	Chemotherapy alone
Diverticular disease of intestine	Gastrointestinal	Liver and intrahepatic bile duct	1.25	0.81	1.92	0.318	Chemotherapy alone
Fatty liver	Gastrointestinal	Liver and intrahepatic bile duct	0.80	0.59	1.08	0.147	Chemotherapy alone
Gastritis and duodenitis	Gastrointestinal	Liver and intrahepatic bile duct	0.82	0.59	1.16	0.265	Chemotherapy alone
Gastrooesophageal reflux disease	Gastrointestinal	Liver and intrahepatic bile duct	1.09	0.67	1.76	0.737	Chemotherapy alone
Liver fibrosis sclerosis and cirrhosis	Gastrointestinal	Liver and intrahepatic bile duct	0.75	0.54	1.04	0.085	Chemotherapy alone
Oesophageal varices	Gastrointestinal	Liver and intrahepatic bile duct	0.73	0.49	1.09	0.127	Chemotherapy alone
Oesophagitis and oesophageal ulcer	Gastrointestinal	Liver and intrahepatic bile duct	1.02	0.67	1.55	0.926	Chemotherapy alone
Portal hypertension	Gastrointestinal	Liver and intrahepatic bile duct	0.91	0.66	1.25	0.554	Chemotherapy alone
Other anaemias	Haematological	Liver and intrahepatic bile duct	0.89	0.59	1.33	0.558	Chemotherapy alone
Primary or idiopathic thrombocytopaenia	Haematological	Liver and intrahepatic bile duct	1.49	0.71	3.12	0.288	Chemotherapy alone
Secondary or other thrombocytopaenia	Haematological	Liver and intrahepatic bile duct	1.15	0.62	2.15	0.659	Chemotherapy alone
Viral diseases (excluding chronic hepatitis/HIV)	Immunology and infection	Liver and intrahepatic bile duct	0.32	0.16	0.64	0.001	Chemotherapy alone
Autoimmune liver disease	Immunology and infection	Liver and intrahepatic bile duct	0.56	0.39	0.81	0.002	Chemotherapy alone
Chronic viral hepatitis	Immunology and infection	Liver and intrahepatic bile duct	0.67	0.45	0.99	0.046	Chemotherapy alone
Infection of liver	Immunology and infection	Liver and intrahepatic bile duct	0.49	0.32	0.76	0.001	Chemotherapy alone
Infections of other or unspecified organs	Immunology and infection	Liver and intrahepatic bile duct	0.62	0.40	0.95	0.028	Chemotherapy alone
Lower respiratory tract infections	Immunology and infection	Liver and intrahepatic bile duct	0.60	0.41	0.88	0.008	Chemotherapy alone
Infection of skin and subcutaneous tissues	Immunology and infection	Liver and intrahepatic bile duct	0.73	0.43	1.25	0.253	Chemotherapy alone
Infections of the heart	Immunology and infection	Liver and intrahepatic bile duct	0.71	0.44	1.15	0.167	Chemotherapy alone
Infections of the digestive system	Immunology and infection	Liver and intrahepatic bile duct	1.40	0.89	2.22	0.149	Chemotherapy alone
Peritonitis	Immunology and infection	Liver and intrahepatic bile duct	0.95	0.69	1.32	0.764	Chemotherapy alone
Enthesopathies and synovial disorders	Musculoskeletal	Liver and intrahepatic bile duct	0.82	0.48	1.40	0.473	Chemotherapy alone
Osteoarthritis (excluding spine)	Musculoskeletal	Liver and intrahepatic bile duct	0.99	0.63	1.55	0.971	Chemotherapy alone
Osteoporosis	Musculoskeletal	Liver and intrahepatic bile duct	1.03	0.55	1.94	0.925	Chemotherapy alone
Peripheral neuropathies	Neurological	Liver and intrahepatic bile duct	0.76	0.39	1.46	0.407	Chemotherapy alone
Asthma	Pulmonary	Liver and intrahepatic bile duct	0.50	0.27	0.93	0.029	Chemotherapy alone
COPD	Pulmonary	Liver and intrahepatic bile duct	1.01	0.61	1.68	0.964	Chemotherapy alone
End stage renal disease	Renal	Liver and intrahepatic bile duct	0.60	0.36	1.01	0.055	Chemotherapy alone
Heart failure	Cardiovascular	Liver and intrahepatic bile duct	2.16	1.07	4.39	0.033	Radiotherapy alone
Coronary heart disease not otherwise specified	Cardiovascular	Liver and intrahepatic bile duct	1.43	0.70	2.88	0.325	Radiotherapy alone
Hypertension	Cardiovascular	Liver and intrahepatic bile duct	1.04	0.60	1.82	0.885	Radiotherapy alone
Diabetic neurological complications	Endocrine	Liver and intrahepatic bile duct	1.41	0.80	2.49	0.238	Radiotherapy alone
Diabetic ophthalmic complications	Endocrine	Liver and intrahepatic bile duct	1.44	0.82	2.55	0.207	Radiotherapy alone
Hepatic failure	Gastrointestinal	Liver and intrahepatic bile duct	0.51	0.27	0.96	0.038	Radiotherapy alone
Fatty liver	Gastrointestinal	Liver and intrahepatic bile duct	0.57	0.30	1.08	0.085	Radiotherapy alone
Portal hypertension	Gastrointestinal	Liver and intrahepatic bile duct	0.58	0.29	1.16	0.125	Radiotherapy alone
Other anaemias	Haematological	Liver and intrahepatic bile duct	1.23	0.62	2.45	0.551	Radiotherapy alone
Lower respiratory tract infections	Immunology and infection	Liver and intrahepatic bile duct	0.84	0.42	1.68	0.621	Radiotherapy alone
Peritonitis	Immunology and infection	Liver and intrahepatic bile duct	0.89	0.47	1.69	0.726	Radiotherapy alone
Osteoarthritis (excluding spine)	Musculoskeletal	Liver and intrahepatic bile duct	1.92	0.97	3.81	0.062	Radiotherapy alone
Atrial fibrillation	Cardiovascular	Lung and bronchus	0.53	0.44	0.64	< 0.001	Chemotherapy & radiotherapy
Bifascicular block	Cardiovascular	Lung and bronchus	0.61	0.38	0.98	0.039	Chemotherapy & radiotherapy
Heart failure	Cardiovascular	Lung and bronchus	0.63	0.50	0.79	< 0.001	Chemotherapy & radiotherapy
Ischaemic stroke	Cardiovascular	Lung and bronchus	0.58	0.40	0.83	0.003	Chemotherapy & radiotherapy
Multiple valve disease	Cardiovascular	Lung and bronchus	0.44	0.25	0.79	0.006	Chemotherapy & radiotherapy
Nonrheumatic mitral valve disorders	Cardiovascular	Lung and bronchus	0.55	0.32	0.94	0.028	Chemotherapy & radiotherapy
Other cardiomyopathy	Cardiovascular	Lung and bronchus	0.81	0.68	0.96	0.014	Chemotherapy & radiotherapy
Peripheral arterial disease	Cardiovascular	Lung and bronchus	0.77	0.62	0.95	0.017	Chemotherapy & radiotherapy
Right bundle branch block	Cardiovascular	Lung and bronchus	0.61	0.38	0.99	0.044	Chemotherapy & radiotherapy
Trifascicular block	Cardiovascular	Lung and bronchus	0.44	0.26	0.74	0.002	Chemotherapy & radiotherapy
Venous thromboembolic disease	Cardiovascular	Lung and bronchus	0.48	0.37	0.61	< 0.001	Chemotherapy & radiotherapy
Coronary heart disease not otherwise specified	Cardiovascular	Lung and bronchus	1.19	1.03	1.38	0.017	Chemotherapy & radiotherapy
Ventricular tachycardia	Cardiovascular	Lung and bronchus	0.74	0.46	1.19	0.215	Chemotherapy & radiotherapy
Abdominal aortic aneurysm	Cardiovascular	Lung and bronchus	1.13	0.86	1.47	0.382	Chemotherapy & radiotherapy
Atrioventricular block, complete	Cardiovascular	Lung and bronchus	0.82	0.54	1.25	0.365	Chemotherapy & radiotherapy
Atrioventricular block, first degree	Cardiovascular	Lung and bronchus	0.82	0.54	1.25	0.365	Chemotherapy & radiotherapy
Atrioventricular block, second degree	Cardiovascular	Lung and bronchus	0.82	0.54	1.25	0.365	Chemotherapy & radiotherapy
Hypertension	Cardiovascular	Lung and bronchus	1.03	0.92	1.15	0.611	Chemotherapy & radiotherapy
Left bundle branch block	Cardiovascular	Lung and bronchus	0.81	0.54	1.23	0.325	Chemotherapy & radiotherapy
Myocardial infarction	Cardiovascular	Lung and bronchus	0.88	0.74	1.05	0.164	Chemotherapy & radiotherapy
Nonrheumatic aortic valve disorders	Cardiovascular	Lung and bronchus	1.08	0.69	1.68	0.73	Chemotherapy & radiotherapy
Pericardial effusion (noninflammatory)	Cardiovascular	Lung and bronchus	0.67	0.39	1.15	0.15	Chemotherapy & radiotherapy
Primary pulmonary hypertension	Cardiovascular	Lung and bronchus	0.51	0.24	1.04	0.065	Chemotherapy & radiotherapy
Raynaud's syndrome	Cardiovascular	Lung and bronchus	0.81	0.62	1.05	0.109	Chemotherapy & radiotherapy
Sick sinus syndrome	Cardiovascular	Lung and bronchus	0.56	0.29	1.07	0.078	Chemotherapy & radiotherapy
Stable angina	Cardiovascular	Lung and bronchus	1.07	0.88	1.30	0.476	Chemotherapy & radiotherapy
Stroke (not otherwise specified)	Cardiovascular	Lung and bronchus	0.89	0.68	1.18	0.434	Chemotherapy & radiotherapy
Supraventricular tachycardia	Cardiovascular	Lung and bronchus	0.73	0.46	1.16	0.18	Chemotherapy & radiotherapy
Transient ischaemic attack	Cardiovascular	Lung and bronchus	0.89	0.70	1.15	0.385	Chemotherapy & radiotherapy
Unstable angina	Cardiovascular	Lung and bronchus	1.06	0.87	1.29	0.551	Chemotherapy & radiotherapy
Diabetic neurological complications	Endocrine	Lung and bronchus	0.80	0.67	0.95	0.012	Chemotherapy & radiotherapy
Diabetic ophthalmic complications	Endocrine	Lung and bronchus	0.81	0.69	0.96	0.017	Chemotherapy & radiotherapy
Diabetes	Endocrine	Lung and bronchus	1.14	0.97	1.34	0.111	Chemotherapy & radiotherapy
Hypo or hyperthyroidism	Endocrine	Lung and bronchus	0.90	0.70	1.17	0.434	Chemotherapy & radiotherapy
Barrett's oesophagus	Gastrointestinal	Lung and bronchus	0.71	0.51	0.99	0.042	Chemotherapy & radiotherapy
Fatty liver	Gastrointestinal	Lung and bronchus	0.61	0.43	0.86	0.005	Chemotherapy & radiotherapy
Gastritis and duodenitis	Gastrointestinal	Lung and bronchus	0.82	0.68	0.99	0.042	Chemotherapy & radiotherapy
Hepatic failure	Gastrointestinal	Lung and bronchus	0.54	0.37	0.77	0.001	Chemotherapy & radiotherapy
Liver fibrosis sclerosis and cirrhosis	Gastrointestinal	Lung and bronchus	0.44	0.22	0.89	0.023	Chemotherapy & radiotherapy
Portal hypertension	Gastrointestinal	Lung and bronchus	0.50	0.33	0.76	0.001	Chemotherapy & radiotherapy

Alcoholic liver disease	Gastrointestinal	Lung and bronchus	0.80	0.43	1.48	0.477	Chemotherapy & radiotherapy
Cholangitis	Gastrointestinal	Lung and bronchus	0.80	0.59	1.09	0.159	Chemotherapy & radiotherapy
Cholecystitis	Gastrointestinal	Lung and bronchus	0.77	0.57	1.04	0.092	Chemotherapy & radiotherapy
Cholelithiasis	Gastrointestinal	Lung and bronchus	1.00	0.75	1.33	0.997	Chemotherapy & radiotherapy
Coeliac disease	Gastrointestinal	Lung and bronchus	1.21	0.57	2.57	0.619	Chemotherapy & radiotherapy
Diverticular disease of intestine	Gastrointestinal	Lung and bronchus	0.94	0.77	1.14	0.523	Chemotherapy & radiotherapy
Gastrooesophageal reflux disease	Gastrointestinal	Lung and bronchus	1.00	0.81	1.24	0.982	Chemotherapy & radiotherapy
Oesophagitis and oesophageal ulcer	Gastrointestinal	Lung and bronchus	0.84	0.70	1.01	0.061	Chemotherapy & radiotherapy
Other anaemias	Haematological	Lung and bronchus	0.62	0.51	0.76	< 0.001	Chemotherapy & radiotherapy
Secondary or other thrombocytopaenia	Haematological	Lung and bronchus	0.81	0.42	1.57	0.536	Chemotherapy & radiotherapy
Secondary polycythaemia	Haematological	Lung and bronchus	1.03	0.46	2.27	0.948	Chemotherapy & radiotherapy
Viral diseases (excluding chronic hepatitis/HIV)	Immunology and infection	Lung and bronchus	0.45	0.30	0.68	< 0.001	Chemotherapy & radiotherapy
Infection of skin and subcutaneous tissues	Immunology and infection	Lung and bronchus	0.55	0.41	0.73	< 0.001	Chemotherapy & radiotherapy
Infections of other or unspecified organs	Immunology and infection	Lung and bronchus	0.64	0.53	0.78	< 0.001	Chemotherapy & radiotherapy
Infections of the heart	Immunology and infection	Lung and bronchus	0.38	0.23	0.61	< 0.001	Chemotherapy & radiotherapy
Lower respiratory tract infections	Immunology and infection	Lung and bronchus	0.61	0.54	0.68	< 0.001	Chemotherapy & radiotherapy
Meningitis	Immunology and infection	Lung and bronchus	0.40	0.25	0.65	< 0.001	Chemotherapy & radiotherapy
Chronic viral hepatitis	Immunology and infection	Lung and bronchus	0.95	0.51	1.77	0.869	Chemotherapy & radiotherapy
Infection of bones and joints	Immunology and infection	Lung and bronchus	1.24	0.79	1.93	0.352	Chemotherapy & radiotherapy
Infections of the digestive system	Immunology and infection	Lung and bronchus	0.89	0.70	1.14	0.374	Chemotherapy & radiotherapy
Non-acute cystitis	Immunology and infection	Lung and bronchus	1.01	0.49	2.09	0.971	Chemotherapy & radiotherapy
Peritonitis	Immunology and infection	Lung and bronchus	0.86	0.72	1.02	0.081	Chemotherapy & radiotherapy
Osteoporosis	Musculoskeletal	Lung and bronchus	0.72	0.57	0.91	0.005	Chemotherapy & radiotherapy
Enthesopathies and synovial disorders	Musculoskeletal	Lung and bronchus	1.42	1.19	1.70	< 0.001	Chemotherapy & radiotherapy
Osteoarthritis (excluding spine)	Musculoskeletal	Lung and bronchus	1.00	0.86	1.17	0.993	Chemotherapy & radiotherapy
Rheumatoid arthritis	Musculoskeletal	Lung and bronchus	0.81	0.54	1.20	0.292	Chemotherapy & radiotherapy
Spinal stenosis	Musculoskeletal	Lung and bronchus	1.23	0.81	1.87	0.336	Chemotherapy & radiotherapy
Intracerebral haemorrhage	Neurological	Lung and bronchus	0.43	0.25	0.75	0.003	Chemotherapy & radiotherapy
Intracranial hypertension	Neurological	Lung and bronchus	0.28	0.14	0.55	< 0.001	Chemotherapy & radiotherapy
Subarachnoid haemorrhage	Neurological	Lung and bronchus	0.54	0.31	0.96	0.035	Chemotherapy & radiotherapy
Disorders of autonomic nervous system	Neurological	Lung and bronchus	0.88	0.53	1.47	0.631	Chemotherapy & radiotherapy
Epilepsy	Neurological	Lung and bronchus	0.73	0.43	1.24	0.251	Chemotherapy & radiotherapy
Essential tremor	Neurological	Lung and bronchus	0.89	0.40	1.97	0.767	Chemotherapy & radiotherapy
Peripheral neuropathies	Neurological	Lung and bronchus	0.94	0.71	1.24	0.662	Chemotherapy & radiotherapy
Bronchiectasis	Pulmonary	Lung and bronchus	0.43	0.26	0.71	0.001	Chemotherapy & radiotherapy
COPD	Pulmonary	Lung and bronchus	0.79	0.70	0.88	< 0.001	Chemotherapy & radiotherapy
Other interstitial pulmonary diseases with fibrosis	Pulmonary	Lung and bronchus	0.39	0.27	0.57	< 0.001	Chemotherapy & radiotherapy
Asthma	Pulmonary	Lung and bronchus	0.86	0.71	1.04	0.113	Chemotherapy & radiotherapy
End stage renal disease	Renal	Lung and bronchus	0.46	0.35	0.61	< 0.001	Chemotherapy & radiotherapy
Glomerulonephritis	Renal	Lung and bronchus	0.40	0.22	0.74	0.004	Chemotherapy & radiotherapy
Obstructive and reflux uropathy	Renal	Lung and bronchus	0.63	0.33	1.19	0.151	Chemotherapy & radiotherapy
Urolithiasis	Renal	Lung and bronchus	0.72	0.48	1.08	0.113	Chemotherapy & radiotherapy
Atrial fibrillation	Cardiovascular	Lung and bronchus	0.66	0.50	0.87	0.003	Chemotherapy & surgery
Stroke (not otherwise specified)	Cardiovascular	Lung and bronchus	0.38	0.21	0.70	0.002	Chemotherapy & surgery
Abdominal aortic aneurysm	Cardiovascular	Lung and bronchus	0.78	0.50	1.22	0.28	Chemotherapy & surgery
Atrioventricular block, complete	Cardiovascular	Lung and bronchus	1.36	0.81	2.30	0.243	Chemotherapy & surgery
Atrioventricular block, first degree	Cardiovascular	Lung and bronchus	1.36	0.81	2.30	0.243	Chemotherapy & surgery
Atrioventricular block, second degree	Cardiovascular	Lung and bronchus	1.36	0.81	2.30	0.243	Chemotherapy & surgery
Coronary heart disease not otherwise specified	Cardiovascular	Lung and bronchus	1.09	0.87	1.35	0.464	Chemotherapy & surgery
Heart failure	Cardiovascular	Lung and bronchus	0.76	0.55	1.07	0.113	Chemotherapy & surgery
Hypertension	Cardiovascular	Lung and bronchus	1.07	0.90	1.26	0.438	Chemotherapy & surgery
Left bundle branch block	Cardiovascular	Lung and bronchus	1.26	0.75	2.11	0.385	Chemotherapy & surgery
Myocardial infarction	Cardiovascular	Lung and bronchus	1.13	0.89	1.45	0.322	Chemotherapy & surgery
Nonrheumatic aortic valve disorders	Cardiovascular	Lung and bronchus	1.36	0.75	2.47	0.305	Chemotherapy & surgery
Other cardiomyopathy	Cardiovascular	Lung and bronchus	1.16	0.92	1.46	0.211	Chemotherapy & surgery
Pericardial effusion (noninflammatory)	Cardiovascular	Lung and bronchus	1.13	0.56	2.25	0.738	Chemotherapy & surgery
Peripheral arterial disease	Cardiovascular	Lung and bronchus	0.98	0.73	1.33	0.914	Chemotherapy & surgery
Raynaud's syndrome	Cardiovascular	Lung and bronchus	1.06	0.74	1.52	0.742	Chemotherapy & surgery
Stable angina	Cardiovascular	Lung and bronchus	0.90	0.67	1.22	0.503	Chemotherapy & surgery
Transient ischaemic attack	Cardiovascular	Lung and bronchus	0.75	0.50	1.12	0.159	Chemotherapy & surgery
Unstable angina	Cardiovascular	Lung and bronchus	1.03	0.77	1.39	0.818	Chemotherapy & surgery
Venous thromboembolic disease	Cardiovascular	Lung and bronchus	0.73	0.52	1.02	0.066	Chemotherapy & surgery
Diabetes	Endocrine	Lung and bronchus	1.09	0.86	1.38	0.478	Chemotherapy & surgery
Diabetic neurological complications	Endocrine	Lung and bronchus	0.93	0.71	1.20	0.558	Chemotherapy & surgery
Diabetic ophthalmic complications	Endocrine	Lung and bronchus	0.79	0.61	1.02	0.075	Chemotherapy & surgery
Hypo or hyperthyroidism	Endocrine	Lung and bronchus	0.66	0.43	1.02	0.062	Chemotherapy & surgery
Hepatic failure	Gastrointestinal	Lung and bronchus	0.54	0.30	0.95	0.033	Chemotherapy & surgery
Barrett's oesophagus	Gastrointestinal	Lung and bronchus	0.58	0.33	1.01	0.053	Chemotherapy & surgery
Cholangitis	Gastrointestinal	Lung and bronchus	1.08	0.71	1.64	0.719	Chemotherapy & surgery
Cholecystitis	Gastrointestinal	Lung and bronchus	0.97	0.63	1.48	0.88	Chemotherapy & surgery
Cholelithiasis	Gastrointestinal	Lung and bronchus	1.24	0.84	1.83	0.284	Chemotherapy & surgery
Diverticular disease of intestine	Gastrointestinal	Lung and bronchus	0.80	0.59	1.10	0.168	Chemotherapy & surgery
Fatty liver	Gastrointestinal	Lung and bronchus	0.64	0.38	1.08	0.095	Chemotherapy & surgery
Gastritis and duodenitis	Gastrointestinal	Lung and bronchus	1.24	0.97	1.60	0.087	Chemotherapy & surgery
Gastrooesophageal reflux disease	Gastrointestinal	Lung and bronchus	1.06	0.78	1.44	0.722	Chemotherapy & surgery
Oesophagitis and oesophageal ulcer	Gastrointestinal	Lung and bronchus	0.94	0.72	1.23	0.646	Chemotherapy & surgery
Portal hypertension	Gastrointestinal	Lung and bronchus	0.69	0.39	1.24	0.217	Chemotherapy & surgery
Secondary or other thrombocytopaenia	Haematological	Lung and bronchus	2.40	1.22	4.73	0.011	Chemotherapy & surgery
Other anaemias	Haematological	Lung and bronchus	0.82	0.61	1.08	0.161	Chemotherapy & surgery
Infections of other or unspecified organs	Immunology and infection	Lung and bronchus	0.53	0.39	0.72	< 0.001	Chemotherapy & surgery
Lower respiratory tract infections	Immunology and infection	Lung and bronchus	0.71	0.60	0.84	< 0.001	Chemotherapy & surgery

Infection of skin and subcutaneous tissues	Immunology and infection	Lung and bronchus	0.77	0.52	1.15	0.204	Chemotherapy & surgery
Infections of the digestive system	Immunology and infection	Lung and bronchus	0.92	0.64	1.32	0.649	Chemotherapy & surgery
Peritonitis	Immunology and infection	Lung and bronchus	0.78	0.60	1.02	0.067	Chemotherapy & surgery
Enthesopathies and synovial disorders	Musculoskeletal	Lung and bronchus	1.24	0.95	1.61	0.108	Chemotherapy & surgery
Osteoarthritis (excluding spine)	Musculoskeletal	Lung and bronchus	0.98	0.78	1.23	0.861	Chemotherapy & surgery
Osteoporosis	Musculoskeletal	Lung and bronchus	0.83	0.59	1.17	0.29	Chemotherapy & surgery
Peripheral neuropathies	Neurological	Lung and bronchus	0.91	0.60	1.38	0.664	Chemotherapy & surgery
Asthma	Pulmonary	Lung and bronchus	0.92	0.70	1.22	0.579	Chemotherapy & surgery
COPD	Pulmonary	Lung and bronchus	0.96	0.81	1.13	0.602	Chemotherapy & surgery
Other interstitial pulmonary diseases with fibrosis	Pulmonary	Lung and bronchus	0.64	0.38	1.07	0.088	Chemotherapy & surgery
End stage renal disease	Renal	Lung and bronchus	0.50	0.32	0.77	0.002	Chemotherapy & surgery
Urolithiasis	Renal	Lung and bronchus	0.89	0.51	1.57	0.689	Chemotherapy & surgery
Atrial fibrillation	Cardiovascular	Lung and bronchus	0.77	0.66	0.90	0.001	Chemotherapy alone
Ischaemic stroke	Cardiovascular	Lung and bronchus	0.57	0.41	0.80	0.001	Chemotherapy alone
Peripheral arterial disease	Cardiovascular	Lung and bronchus	0.78	0.64	0.96	0.016	Chemotherapy alone
Stroke (not otherwise specified)	Cardiovascular	Lung and bronchus	0.70	0.53	0.92	0.01	Chemotherapy alone
Trifascicular block	Cardiovascular	Lung and bronchus	0.62	0.40	0.95	0.028	Chemotherapy alone
Venous thromboembolic disease	Cardiovascular	Lung and bronchus	0.70	0.57	0.87	0.001	Chemotherapy alone
Ventricular tachycardia	Cardiovascular	Lung and bronchus	0.55	0.29	1.03	0.062	Chemotherapy alone
Abdominal aortic aneurysm	Cardiovascular	Lung and bronchus	1.22	0.95	1.55	0.117	Chemotherapy alone
Atrioventricular block, complete	Cardiovascular	Lung and bronchus	0.83	0.57	1.21	0.343	Chemotherapy alone
Atrioventricular block, first degree	Cardiovascular	Lung and bronchus	0.83	0.57	1.21	0.343	Chemotherapy alone
Atrioventricular block, second degree	Cardiovascular	Lung and bronchus	0.83	0.57	1.21	0.343	Chemotherapy alone
Bifascicular block	Cardiovascular	Lung and bronchus	0.69	0.46	1.04	0.077	Chemotherapy alone
Coronary heart disease not otherwise specified	Cardiovascular	Lung and bronchus	1.14	0.99	1.31	0.063	Chemotherapy alone
Heart failure	Cardiovascular	Lung and bronchus	0.93	0.77	1.13	0.483	Chemotherapy alone
Hypertension	Cardiovascular	Lung and bronchus	1.01	0.90	1.12	0.925	Chemotherapy alone
Left bundle branch block	Cardiovascular	Lung and bronchus	0.82	0.57	1.17	0.271	Chemotherapy alone
Multiple valve disease	Cardiovascular	Lung and bronchus	0.75	0.49	1.17	0.209	Chemotherapy alone
Myocardial infarction	Cardiovascular	Lung and bronchus	1.03	0.88	1.20	0.726	Chemotherapy alone
Nonrheumatic aortic valve disorders	Cardiovascular	Lung and bronchus	1.26	0.85	1.85	0.246	Chemotherapy alone
Nonrheumatic mitral valve disorders	Cardiovascular	Lung and bronchus	0.76	0.49	1.18	0.217	Chemotherapy alone
Other cardiomyopathy	Cardiovascular	Lung and bronchus	0.98	0.85	1.14	0.813	Chemotherapy alone
Pericardial effusion (noninflammatory)	Cardiovascular	Lung and bronchus	1.43	0.91	2.23	0.119	Chemotherapy alone
Primary pulmonary hypertension	Cardiovascular	Lung and bronchus	0.61	0.33	1.12	0.108	Chemotherapy alone
Raynaud's syndrome	Cardiovascular	Lung and bronchus	0.82	0.64	1.04	0.1	Chemotherapy alone
Right bundle branch block	Cardiovascular	Lung and bronchus	0.70	0.46	1.05	0.086	Chemotherapy alone
Secondary pulmonary hypertension	Cardiovascular	Lung and bronchus	0.57	0.31	1.05	0.072	Chemotherapy alone
Sick sinus syndrome	Cardiovascular	Lung and bronchus	0.79	0.47	1.32	0.366	Chemotherapy alone
Stable angina	Cardiovascular	Lung and bronchus	1.03	0.87	1.24	0.709	Chemotherapy alone
Supraventricular tachycardia	Cardiovascular	Lung and bronchus	0.67	0.44	1.04	0.071	Chemotherapy alone
Transient ischaemic attack	Cardiovascular	Lung and bronchus	1.15	0.92	1.43	0.21	Chemotherapy alone
Unstable angina	Cardiovascular	Lung and bronchus	1.04	0.87	1.24	0.674	Chemotherapy alone
Diabetes	Endocrine	Lung and bronchus	1.19	1.02	1.38	0.024	Chemotherapy alone
Diabetic neurological complications	Endocrine	Lung and bronchus	0.92	0.78	1.08	0.291	Chemotherapy alone
Diabetic ophthalmic complications	Endocrine	Lung and bronchus	0.93	0.80	1.08	0.337	Chemotherapy alone
Hyperparathyroidism	Endocrine	Lung and bronchus	0.96	0.40	2.34	0.936	Chemotherapy alone
Hypo or hyperthyroidism	Endocrine	Lung and bronchus	0.79	0.62	1.00	0.053	Chemotherapy alone
Gastritis and duodenitis	Gastrointestinal	Lung and bronchus	0.80	0.67	0.96	0.014	Chemotherapy alone
Alcoholic liver disease	Gastrointestinal	Lung and bronchus	1.03	0.60	1.79	0.909	Chemotherapy alone
Angiodysplasia of colon	Gastrointestinal	Lung and bronchus	0.82	0.38	1.80	0.627	Chemotherapy alone
Barrett's oesophagus	Gastrointestinal	Lung and bronchus	0.94	0.71	1.24	0.657	Chemotherapy alone
Cholangitis	Gastrointestinal	Lung and bronchus	0.97	0.74	1.26	0.804	Chemotherapy alone
Cholecystitis	Gastrointestinal	Lung and bronchus	0.92	0.71	1.20	0.55	Chemotherapy alone
Cholelithiasis	Gastrointestinal	Lung and bronchus	0.99	0.76	1.29	0.953	Chemotherapy alone
Coeliac disease	Gastrointestinal	Lung and bronchus	1.08	0.52	2.24	0.836	Chemotherapy alone
Diverticular disease of intestine	Gastrointestinal	Lung and bronchus	0.90	0.75	1.09	0.282	Chemotherapy alone
Fatty liver	Gastrointestinal	Lung and bronchus	0.85	0.63	1.14	0.264	Chemotherapy alone
Gastrooesophageal reflux disease	Gastrointestinal	Lung and bronchus	1.11	0.91	1.35	0.292	Chemotherapy alone
Hepatic failure	Gastrointestinal	Lung and bronchus	0.73	0.54	1.00	0.052	Chemotherapy alone
Liver fibrosis sclerosis and cirrhosis	Gastrointestinal	Lung and bronchus	0.64	0.35	1.16	0.144	Chemotherapy alone
Oesophagitis and oesophageal ulcer	Gastrointestinal	Lung and bronchus	0.92	0.78	1.09	0.338	Chemotherapy alone
Portal hypertension	Gastrointestinal	Lung and bronchus	0.73	0.52	1.04	0.084	Chemotherapy alone
Ulcerative colitis	Gastrointestinal	Lung and bronchus	0.85	0.37	1.95	0.701	Chemotherapy alone
Other anaemias	Haematological	Lung and bronchus	0.66	0.55	0.79	< 0.001	Chemotherapy alone
Agranulocytosis	Haematological	Lung and bronchus	2.25	1.09	4.64	0.029	Chemotherapy alone
Primary or idiopathic thrombocytopaenia	Haematological	Lung and bronchus	1.78	0.88	3.58	0.108	Chemotherapy alone
Secondary or other thrombocytopaenia	Haematological	Lung and bronchus	1.20	0.69	2.08	0.516	Chemotherapy alone
Secondary polycythaemia	Haematological	Lung and bronchus	1.44	0.73	2.85	0.292	Chemotherapy alone
Viral diseases (excluding chronic hepatitis/HIV)	Immunology and infection	Lung and bronchus	0.58	0.41	0.82	0.002	Chemotherapy alone
Infections of other or unspecified organs	Immunology and infection	Lung and bronchus	0.66	0.55	0.78	< 0.001	Chemotherapy alone
Infections of the heart	Immunology and infection	Lung and bronchus	0.59	0.40	0.87	0.007	Chemotherapy alone
Lower respiratory tract infections	Immunology and infection	Lung and bronchus	0.72	0.65	0.79	< 0.001	Chemotherapy alone
Meningitis	Immunology and infection	Lung and bronchus	0.67	0.46	0.98	0.04	Chemotherapy alone
Autoimmune liver disease	Immunology and infection	Lung and bronchus	0.80	0.37	1.71	0.562	Chemotherapy alone
Chronic viral hepatitis	Immunology and infection	Lung and bronchus	0.80	0.43	1.47	0.472	Chemotherapy alone
Infection of bones and joints	Immunology and infection	Lung and bronchus	0.68	0.42	1.09	0.105	Chemotherapy alone
Infection of skin and subcutaneous tissues	Immunology and infection	Lung and bronchus	0.79	0.62	1.00	0.052	Chemotherapy alone
Infections of the digestive system	Immunology and infection	Lung and bronchus	1.00	0.80	1.25	0.992	Chemotherapy alone

Non-acute cystitis	Immunology and infection	Lung and bronchus	1.52	0.84	2.77	0.169	Chemotherapy alone
Peritonitis	Immunology and infection	Lung and bronchus	0.89	0.76	1.04	0.146	Chemotherapy alone
Osteoporosis	Musculoskeletal	Lung and bronchus	0.67	0.54	0.83	< 0.001	Chemotherapy alone
Enthesopathies and synovial disorders	Musculoskeletal	Lung and bronchus	1.22	1.03	1.45	0.025	Chemotherapy alone
Osteoarthritis (excluding spine)	Musculoskeletal	Lung and bronchus	0.96	0.83	1.11	0.57	Chemotherapy alone
Rheumatoid arthritis	Musculoskeletal	Lung and bronchus	0.87	0.61	1.25	0.46	Chemotherapy alone
Scoliosis	Musculoskeletal	Lung and bronchus	0.96	0.49	1.85	0.898	Chemotherapy alone
Spinal stenosis	Musculoskeletal	Lung and bronchus	0.75	0.49	1.16	0.198	Chemotherapy alone
Dementia	Neurological	Lung and bronchus	0.21	0.13	0.34	< 0.001	Chemotherapy alone
Disorders of autonomic nervous system	Neurological	Lung and bronchus	0.34	0.18	0.62	0.001	Chemotherapy alone
Intracerebral haemorrhage	Neurological	Lung and bronchus	0.40	0.24	0.66	< 0.001	Chemotherapy alone
Intracranial hypertension	Neurological	Lung and bronchus	0.31	0.17	0.56	< 0.001	Chemotherapy alone
Subarachnoid haemorrhage	Neurological	Lung and bronchus	0.46	0.27	0.78	0.004	Chemotherapy alone
Epilepsy	Neurological	Lung and bronchus	0.63	0.38	1.05	0.078	Chemotherapy alone
Essential tremor	Neurological	Lung and bronchus	0.88	0.42	1.82	0.731	Chemotherapy alone
Peripheral neuropathies	Neurological	Lung and bronchus	0.93	0.72	1.20	0.572	Chemotherapy alone
Bronchiectasis	Pulmonary	Lung and bronchus	0.60	0.40	0.89	0.012	Chemotherapy alone
COPD	Pulmonary	Lung and bronchus	0.87	0.78	0.97	0.01	Chemotherapy alone
Other interstitial pulmonary diseases with fibrosis	Pulmonary	Lung and bronchus	0.51	0.37	0.71	< 0.001	Chemotherapy alone
Asthma	Pulmonary	Lung and bronchus	0.88	0.74	1.04	0.142	Chemotherapy alone
End stage renal disease	Renal	Lung and bronchus	0.69	0.55	0.87	0.001	Chemotherapy alone
Glomerulonephritis	Renal	Lung and bronchus	0.39	0.22	0.68	0.001	Chemotherapy alone
Obstructive and reflux uropathy	Renal	Lung and bronchus	0.54	0.29	0.99	0.046	Chemotherapy alone
Urolithiasis	Renal	Lung and bronchus	0.68	0.47	1.00	0.048	Chemotherapy alone
Abdominal aortic aneurysm	Cardiovascular	Lung and bronchus	0.53	0.31	0.91	0.021	Chemotherapy, radiotherapy & surgery
Atrial fibrillation	Cardiovascular	Lung and bronchus	0.63	0.46	0.84	0.002	Chemotherapy, radiotherapy & surgery
Peripheral arterial disease	Cardiovascular	Lung and bronchus	0.62	0.43	0.90	0.012	Chemotherapy, radiotherapy & surgery
Venous thromboembolic disease	Cardiovascular	Lung and bronchus	0.52	0.35	0.78	0.002	Chemotherapy, radiotherapy & surgery
Bifascicular block	Cardiovascular	Lung and bronchus	0.97	0.51	1.86	0.93	Chemotherapy, radiotherapy & surgery
Coronary heart disease not otherwise specified	Cardiovascular	Lung and bronchus	0.88	0.69	1.12	0.312	Chemotherapy, radiotherapy & surgery
Heart failure	Cardiovascular	Lung and bronchus	0.79	0.55	1.12	0.183	Chemotherapy, radiotherapy & surgery
Hypertension	Cardiovascular	Lung and bronchus	1.02	0.86	1.21	0.807	Chemotherapy, radiotherapy & surgery
Ischaemic stroke	Cardiovascular	Lung and bronchus	0.65	0.36	1.16	0.143	Chemotherapy, radiotherapy & surgery
Myocardial infarction	Cardiovascular	Lung and bronchus	0.96	0.73	1.26	0.774	Chemotherapy, radiotherapy & surgery
Other cardiomyopathy	Cardiovascular	Lung and bronchus	0.88	0.68	1.16	0.365	Chemotherapy, radiotherapy & surgery
Raynaud's syndrome	Cardiovascular	Lung and bronchus	0.81	0.53	1.23	0.32	Chemotherapy, radiotherapy & surgery
Right bundle branch block	Cardiovascular	Lung and bronchus	0.98	0.51	1.88	0.949	Chemotherapy, radiotherapy & surgery
Stable angina	Cardiovascular	Lung and bronchus	0.95	0.70	1.30	0.765	Chemotherapy, radiotherapy & surgery
Stroke (not otherwise specified)	Cardiovascular	Lung and bronchus	0.79	0.49	1.27	0.332	Chemotherapy, radiotherapy & surgery
Transient ischaemic attack	Cardiovascular	Lung and bronchus	0.73	0.48	1.12	0.153	Chemotherapy, radiotherapy & surgery
Unstable angina	Cardiovascular	Lung and bronchus	0.91	0.66	1.26	0.572	Chemotherapy, radiotherapy & surgery
Diabetes	Endocrine	Lung and bronchus	0.91	0.70	1.17	0.459	Chemotherapy, radiotherapy & surgery
Diabetic neurological complications	Endocrine	Lung and bronchus	0.81	0.61	1.08	0.154	Chemotherapy, radiotherapy & surgery
Diabetic ophthalmic complications	Endocrine	Lung and bronchus	0.88	0.68	1.15	0.362	Chemotherapy, radiotherapy & surgery
Hypo or hyperthyroidism	Endocrine	Lung and bronchus	0.76	0.50	1.15	0.194	Chemotherapy, radiotherapy & surgery
Barrett's oesophagus	Gastrointestinal	Lung and bronchus	0.67	0.39	1.16	0.149	Chemotherapy, radiotherapy & surgery
Cholangitis	Gastrointestinal	Lung and bronchus	1.16	0.75	1.78	0.51	Chemotherapy, radiotherapy & surgery
Cholecystitis	Gastrointestinal	Lung and bronchus	1.07	0.69	1.64	0.768	Chemotherapy, radiotherapy & surgery
Cholelithiasis	Gastrointestinal	Lung and bronchus	1.17	0.77	1.77	0.466	Chemotherapy, radiotherapy & surgery
Diverticular disease of intestine	Gastrointestinal	Lung and bronchus	0.90	0.66	1.23	0.509	Chemotherapy, radiotherapy & surgery
Fatty liver	Gastrointestinal	Lung and bronchus	0.77	0.47	1.28	0.315	Chemotherapy, radiotherapy & surgery
Gastritis and duodenitis	Gastrointestinal	Lung and bronchus	1.12	0.86	1.46	0.41	Chemotherapy, radiotherapy & surgery
Gastrooesophageal reflux disease	Gastrointestinal	Lung and bronchus	1.02	0.74	1.41	0.905	Chemotherapy, radiotherapy & surgery
Hepatic failure	Gastrointestinal	Lung and bronchus	0.86	0.52	1.40	0.541	Chemotherapy, radiotherapy & surgery
Oesophagitis and oesophageal ulcer	Gastrointestinal	Lung and bronchus	0.77	0.57	1.04	0.086	Chemotherapy, radiotherapy & surgery
Portal hypertension	Gastrointestinal	Lung and bronchus	0.90	0.52	1.56	0.706	Chemotherapy, radiotherapy & surgery
Other anaemias	Haematological	Lung and bronchus	0.58	0.41	0.81	0.002	Chemotherapy, radiotherapy & surgery
Infection of skin and subcutaneous tissues	Immunology and infection	Lung and bronchus	0.60	0.38	0.95	0.029	Chemotherapy, radiotherapy & surgery
Infections of other or unspecified organs	Immunology and infection	Lung and bronchus	0.69	0.51	0.92	0.013	Chemotherapy, radiotherapy & surgery
Lower respiratory tract infections	Immunology and infection	Lung and bronchus	0.54	0.45	0.65	< 0.001	Chemotherapy, radiotherapy & surgery
Viral diseases (excluding chronic hepatitis/HIV)	Immunology and infection	Lung and bronchus	0.62	0.35	1.11	0.107	Chemotherapy, radiotherapy & surgery
Infection of bones and joints	Immunology and infection	Lung and bronchus	1.70	0.94	3.07	0.079	Chemotherapy, radiotherapy & surgery
Infections of the digestive system	Immunology and infection	Lung and bronchus	1.11	0.78	1.58	0.568	Chemotherapy, radiotherapy & surgery
Peritonitis	Immunology and infection	Lung and bronchus	0.99	0.77	1.28	0.936	Chemotherapy, radiotherapy & surgery
Enthesopathies and synovial disorders	Musculoskeletal	Lung and bronchus	1.61	1.26	2.05	< 0.001	Chemotherapy, radiotherapy & surgery
Osteoarthritis (excluding spine)	Musculoskeletal	Lung and bronchus	0.87	0.68	1.11	0.263	Chemotherapy, radiotherapy & surgery
Osteoporosis	Musculoskeletal	Lung and bronchus	0.72	0.50	1.05	0.087	Chemotherapy, radiotherapy & surgery
Rheumatoid arthritis	Musculoskeletal	Lung and bronchus	0.97	0.55	1.72	0.916	Chemotherapy, radiotherapy & surgery
Spinal stenosis	Musculoskeletal	Lung and bronchus	1.10	0.58	2.11	0.765	Chemotherapy, radiotherapy & surgery
Disorders of autonomic nervous system	Neurological	Lung and bronchus	1.69	0.91	3.14	0.094	Chemotherapy, radiotherapy & surgery
Peripheral neuropathies	Neurological	Lung and bronchus	0.97	0.64	1.48	0.888	Chemotherapy, radiotherapy & surgery
Other interstitial pulmonary diseases with fibrosis	Pulmonary	Lung and bronchus	0.43	0.23	0.81	0.008	Chemotherapy, radiotherapy & surgery
Asthma	Pulmonary	Lung and bronchus	0.86	0.65	1.15	0.316	Chemotherapy, radiotherapy & surgery
COPD	Pulmonary	Lung and bronchus	0.95	0.80	1.13	0.558	Chemotherapy, radiotherapy & surgery
End stage renal disease	Renal	Lung and bronchus	0.43	0.26	0.70	0.001	Chemotherapy, radiotherapy & surgery
Urolithiasis	Renal	Lung and bronchus	0.95	0.54	1.68	0.858	Chemotherapy, radiotherapy & surgery
Atrial fibrillation	Cardiovascular	Lung and bronchus	0.72	0.58	0.88	0.002	Radiotherapy & surgery
Ischaemic stroke	Cardiovascular	Lung and bronchus	0.50	0.30	0.81	0.006	Radiotherapy & surgery
Ventricular tachycardia	Cardiovascular	Lung and bronchus	1.16	0.71	1.89	0.56	Radiotherapy & surgery

Abdominal aortic aneurysm	Cardiovascular	Lung and bronchus	0.85	0.61	1.20	0.364	Radiotherapy & surgery
Atrioventricular block, complete	Cardiovascular	Lung and bronchus	0.77	0.47	1.27	0.312	Radiotherapy & surgery
Atrioventricular block, first degree	Cardiovascular	Lung and bronchus	0.77	0.47	1.27	0.312	Radiotherapy & surgery
Atrioventricular block, second degree	Cardiovascular	Lung and bronchus	0.77	0.47	1.27	0.312	Radiotherapy & surgery
Coronary heart disease not otherwise specified	Cardiovascular	Lung and bronchus	1.16	0.97	1.39	0.095	Radiotherapy & surgery
Heart failure	Cardiovascular	Lung and bronchus	0.92	0.72	1.18	0.518	Radiotherapy & surgery
Hypertension	Cardiovascular	Lung and bronchus	1.02	0.89	1.17	0.752	Radiotherapy & surgery
Left bundle branch block	Cardiovascular	Lung and bronchus	0.83	0.52	1.32	0.435	Radiotherapy & surgery
Myocardial infarction	Cardiovascular	Lung and bronchus	1.02	0.83	1.25	0.837	Radiotherapy & surgery
Nonrheumatic aortic valve disorders	Cardiovascular	Lung and bronchus	1.04	0.62	1.73	0.88	Radiotherapy & surgery
Nonrheumatic mitral valve disorders	Cardiovascular	Lung and bronchus	1.03	0.61	1.76	0.901	Radiotherapy & surgery
Other cardiomyopathy	Cardiovascular	Lung and bronchus	0.90	0.74	1.10	0.326	Radiotherapy & surgery
Peripheral arterial disease	Cardiovascular	Lung and bronchus	0.79	0.61	1.03	0.086	Radiotherapy & surgery
Raynaud's syndrome	Cardiovascular	Lung and bronchus	0.93	0.69	1.27	0.667	Radiotherapy & surgery
Stable angina	Cardiovascular	Lung and bronchus	1.17	0.94	1.47	0.164	Radiotherapy & surgery
Stroke (not otherwise specified)	Cardiovascular	Lung and bronchus	1.04	0.75	1.44	0.821	Radiotherapy & surgery
Supraventricular tachycardia	Cardiovascular	Lung and bronchus	1.16	0.71	1.87	0.558	Radiotherapy & surgery
Transient ischaemic attack	Cardiovascular	Lung and bronchus	0.90	0.67	1.21	0.478	Radiotherapy & surgery
Unstable angina	Cardiovascular	Lung and bronchus	1.14	0.91	1.43	0.259	Radiotherapy & surgery
Venous thromboembolic disease	Cardiovascular	Lung and bronchus	0.83	0.63	1.08	0.171	Radiotherapy & surgery
Hypo or hyperthyroidism	Endocrine	Lung and bronchus	0.62	0.44	0.88	0.008	Radiotherapy & surgery
Diabetes	Endocrine	Lung and bronchus	0.88	0.72	1.09	0.247	Radiotherapy & surgery
Diabetic neurological complications	Endocrine	Lung and bronchus	0.88	0.71	1.09	0.232	Radiotherapy & surgery
Diabetic ophthalmic complications	Endocrine	Lung and bronchus	0.87	0.71	1.07	0.189	Radiotherapy & surgery
Barrett's oesophagus	Gastrointestinal	Lung and bronchus	0.61	0.40	0.94	0.026	Radiotherapy & surgery
Hepatic failure	Gastrointestinal	Lung and bronchus	0.53	0.32	0.88	0.014	Radiotherapy & surgery
Portal hypertension	Gastrointestinal	Lung and bronchus	0.46	0.25	0.83	0.011	Radiotherapy & surgery
Cholangitis	Gastrointestinal	Lung and bronchus	1.03	0.73	1.46	0.859	Radiotherapy & surgery
Cholecystitis	Gastrointestinal	Lung and bronchus	0.94	0.66	1.33	0.709	Radiotherapy & surgery
Cholelithiasis	Gastrointestinal	Lung and bronchus	1.09	0.78	1.53	0.604	Radiotherapy & surgery
Diverticular disease of intestine	Gastrointestinal	Lung and bronchus	0.94	0.74	1.19	0.586	Radiotherapy & surgery
Fatty liver	Gastrointestinal	Lung and bronchus	0.69	0.44	1.08	0.101	Radiotherapy & surgery
Gastritis and duodenitis	Gastrointestinal	Lung and bronchus	1.01	0.81	1.26	0.928	Radiotherapy & surgery
Gastrooesophageal reflux disease	Gastrointestinal	Lung and bronchus	1.13	0.88	1.45	0.349	Radiotherapy & surgery
Oesophagitis and oesophageal ulcer	Gastrointestinal	Lung and bronchus	0.86	0.69	1.08	0.206	Radiotherapy & surgery
Other anaemias	Haematological	Lung and bronchus	0.77	0.61	0.97	0.028	Radiotherapy & surgery
Secondary or other thrombocytopaenia	Haematological	Lung and bronchus	1.21	0.60	2.47	0.592	Radiotherapy & surgery
Viral diseases (excluding chronic hepatitis/HIV)	Immunology and infection	Lung and bronchus	0.42	0.29	0.61	< 0.001	Radiotherapy & surgery
Infections of other or unspecified organs	Immunology and infection	Lung and bronchus	0.79	0.63	0.99	0.042	Radiotherapy & surgery
Lower respiratory tract infections	Immunology and infection	Lung and bronchus	0.70	0.61	0.81	< 0.001	Radiotherapy & surgery
Infection of bones and joints	Immunology and infection	Lung and bronchus	1.99	1.22	3.25	0.006	Radiotherapy & surgery
Infection of skin and subcutaneous tissues	Immunology and infection	Lung and bronchus	1.01	0.74	1.37	0.955	Radiotherapy & surgery
Infections of the heart	Immunology and infection	Lung and bronchus	0.79	0.47	1.31	0.355	Radiotherapy & surgery
Infections of the digestive system	Immunology and infection	Lung and bronchus	1.00	0.74	1.34	1	Radiotherapy & surgery
Meningitis	Immunology and infection	Lung and bronchus	0.71	0.42	1.19	0.196	Radiotherapy & surgery
Peritonitis	Immunology and infection	Lung and bronchus	1.08	0.89	1.31	0.454	Radiotherapy & surgery
Enthesopathies and synovial disorders	Musculoskeletal	Lung and bronchus	1.34	1.08	1.66	0.009	Radiotherapy & surgery
Osteoarthritis (excluding spine)	Musculoskeletal	Lung and bronchus	1.14	0.95	1.36	0.154	Radiotherapy & surgery
Osteoporosis	Musculoskeletal	Lung and bronchus	1.06	0.82	1.37	0.653	Radiotherapy & surgery
Rheumatoid arthritis	Musculoskeletal	Lung and bronchus	1.07	0.68	1.68	0.782	Radiotherapy & surgery
Scoliosis	Musculoskeletal	Lung and bronchus	1.53	0.74	3.14	0.249	Radiotherapy & surgery
Spinal stenosis	Musculoskeletal	Lung and bronchus	1.38	0.85	2.23	0.193	Radiotherapy & surgery
Dementia	Neurological	Lung and bronchus	0.35	0.20	0.62	< 0.001	Radiotherapy & surgery
Bell's palsy	Neurological	Lung and bronchus	2.09	0.94	4.66	0.072	Radiotherapy & surgery
Disorders of autonomic nervous system	Neurological	Lung and bronchus	1.06	0.57	1.99	0.844	Radiotherapy & surgery
Epilepsy	Neurological	Lung and bronchus	1.39	0.80	2.42	0.239	Radiotherapy & surgery
Intracranial hypertension	Neurological	Lung and bronchus	1.24	0.67	2.27	0.495	Radiotherapy & surgery
Peripheral neuropathies	Neurological	Lung and bronchus	1.16	0.84	1.60	0.368	Radiotherapy & surgery
Other interstitial pulmonary diseases with fibrosis	Pulmonary	Lung and bronchus	0.64	0.42	0.96	0.029	Radiotherapy & surgery
Asthma	Pulmonary	Lung and bronchus	1.02	0.82	1.28	0.832	Radiotherapy & surgery
Bronchiectasis	Pulmonary	Lung and bronchus	0.71	0.43	1.19	0.198	Radiotherapy & surgery
COPD	Pulmonary	Lung and bronchus	0.95	0.83	1.09	0.482	Radiotherapy & surgery
End stage renal disease	Renal	Lung and bronchus	0.70	0.52	0.95	0.022	Radiotherapy & surgery
Glomerulonephritis	Renal	Lung and bronchus	0.89	0.50	1.58	0.695	Radiotherapy & surgery
Obstructive and reflux uropathy	Renal	Lung and bronchus	0.97	0.50	1.91	0.938	Radiotherapy & surgery
Urolithiasis	Renal	Lung and bronchus	1.03	0.66	1.60	0.903	Radiotherapy & surgery
Atrial fibrillation	Cardiovascular	Lung and bronchus	0.82	0.72	0.93	0.003	Radiotherapy alone
Multiple valve disease	Cardiovascular	Lung and bronchus	0.64	0.44	0.94	0.021	Radiotherapy alone
Primary pulmonary hypertension	Cardiovascular	Lung and bronchus	0.54	0.32	0.92	0.022	Radiotherapy alone
Secondary pulmonary hypertension	Cardiovascular	Lung and bronchus	0.47	0.27	0.81	0.006	Radiotherapy alone
Coronary heart disease not otherwise specified	Cardiovascular	Lung and bronchus	1.35	1.20	1.52	< 0.001	Radiotherapy alone
Other cardiomyopathy	Cardiovascular	Lung and bronchus	1.16	1.02	1.32	0.023	Radiotherapy alone
Stable angina	Cardiovascular	Lung and bronchus	1.26	1.08	1.46	0.003	Radiotherapy alone
Unstable angina	Cardiovascular	Lung and bronchus	1.32	1.13	1.53	< 0.001	Radiotherapy alone
Ventricular tachycardia	Cardiovascular	Lung and bronchus	1.14	0.72	1.82	0.576	Radiotherapy alone
Abdominal aortic aneurysm	Cardiovascular	Lung and bronchus	0.80	0.63	1.00	0.05	Radiotherapy alone
Atrioventricular block, complete	Cardiovascular	Lung and bronchus	0.85	0.63	1.16	0.301	Radiotherapy alone
Atrioventricular block, first degree	Cardiovascular	Lung and bronchus	0.85	0.63	1.16	0.301	Radiotherapy alone
Atrioventricular block, second degree	Cardiovascular	Lung and bronchus	0.85	0.63	1.16	0.301	Radiotherapy alone
Bifascicular block	Cardiovascular	Lung and bronchus	0.82	0.59	1.15	0.246	Radiotherapy alone
Dilated cardiomyopathy	Cardiovascular	Lung and bronchus	1.35	0.65	2.83	0.42	Radiotherapy alone

Heart failure	Cardiovascular	Lung and bronchus	1.06	0.91	1.24	0.437	Radiotherapy alone
Hypertension	Cardiovascular	Lung and bronchus	0.94	0.86	1.04	0.232	Radiotherapy alone
Hypertrophic cardiomyopathy	Cardiovascular	Lung and bronchus	1.37	0.66	2.86	0.394	Radiotherapy alone
Ischaemic stroke	Cardiovascular	Lung and bronchus	1.04	0.81	1.34	0.751	Radiotherapy alone
Left bundle branch block	Cardiovascular	Lung and bronchus	0.84	0.62	1.13	0.254	Radiotherapy alone
Myocardial infarction	Cardiovascular	Lung and bronchus	1.14	1.00	1.30	0.054	Radiotherapy alone
Nonrheumatic aortic valve disorders	Cardiovascular	Lung and bronchus	0.98	0.69	1.38	0.906	Radiotherapy alone
Nonrheumatic mitral valve disorders	Cardiovascular	Lung and bronchus	0.82	0.57	1.18	0.291	Radiotherapy alone
Pericardial effusion (noninflammatory)	Cardiovascular	Lung and bronchus	0.74	0.46	1.18	0.203	Radiotherapy alone
Peripheral arterial disease	Cardiovascular	Lung and bronchus	0.91	0.77	1.08	0.281	Radiotherapy alone
Raynaud's syndrome	Cardiovascular	Lung and bronchus	1.09	0.89	1.33	0.407	Radiotherapy alone
Right bundle branch block	Cardiovascular	Lung and bronchus	0.82	0.59	1.15	0.255	Radiotherapy alone
Sick sinus syndrome	Cardiovascular	Lung and bronchus	0.87	0.57	1.33	0.519	Radiotherapy alone
Stroke (not otherwise specified)	Cardiovascular	Lung and bronchus	1.23	1.00	1.52	0.055	Radiotherapy alone
Supraventricular tachycardia	Cardiovascular	Lung and bronchus	0.92	0.65	1.30	0.625	Radiotherapy alone
Transient ischaemic attack	Cardiovascular	Lung and bronchus	1.13	0.93	1.36	0.221	Radiotherapy alone
Trifascicular block	Cardiovascular	Lung and bronchus	0.73	0.51	1.03	0.072	Radiotherapy alone
Venous thromboembolic disease	Cardiovascular	Lung and bronchus	0.89	0.75	1.06	0.188	Radiotherapy alone
Hypo or hyperthyroidism	Endocrine	Lung and bronchus	0.77	0.63	0.95	0.016	Radiotherapy alone
Diabetes	Endocrine	Lung and bronchus	0.94	0.82	1.08	0.387	Radiotherapy alone
Diabetic neurological complications	Endocrine	Lung and bronchus	0.88	0.76	1.01	0.066	Radiotherapy alone
Diabetic ophthalmic complications	Endocrine	Lung and bronchus	0.92	0.81	1.05	0.234	Radiotherapy alone
Hyperparathyroidism	Endocrine	Lung and bronchus	0.77	0.33	1.79	0.536	Radiotherapy alone
Diverticular disease of intestine	Gastrointestinal	Lung and bronchus	0.83	0.71	0.97	0.02	Radiotherapy alone
Hepatic failure	Gastrointestinal	Lung and bronchus	0.71	0.53	0.94	0.019	Radiotherapy alone
Portal hypertension	Gastrointestinal	Lung and bronchus	0.65	0.47	0.90	0.009	Radiotherapy alone
Alcoholic liver disease	Gastrointestinal	Lung and bronchus	1.32	0.80	2.18	0.281	Radiotherapy alone
Angiodysplasia of colon	Gastrointestinal	Lung and bronchus	1.23	0.66	2.29	0.508	Radiotherapy alone
Barrett's oesophagus	Gastrointestinal	Lung and bronchus	0.85	0.66	1.09	0.203	Radiotherapy alone
Cholangitis	Gastrointestinal	Lung and bronchus	1.05	0.84	1.33	0.659	Radiotherapy alone
Cholecystitis	Gastrointestinal	Lung and bronchus	1.05	0.84	1.31	0.691	Radiotherapy alone
Cholelithiasis	Gastrointestinal	Lung and bronchus	1.11	0.88	1.39	0.374	Radiotherapy alone
Coeliac disease	Gastrointestinal	Lung and bronchus	1.43	0.76	2.67	0.266	Radiotherapy alone
Fatty liver	Gastrointestinal	Lung and bronchus	0.77	0.58	1.01	0.059	Radiotherapy alone
Gastritis and duodenitis	Gastrointestinal	Lung and bronchus	0.96	0.82	1.12	0.588	Radiotherapy alone
Gastrooesophageal reflux disease	Gastrointestinal	Lung and bronchus	1.04	0.87	1.24	0.661	Radiotherapy alone
Liver fibrosis sclerosis and cirrhosis	Gastrointestinal	Lung and bronchus	1.28	0.78	2.09	0.326	Radiotherapy alone
Oesophagitis and oesophageal ulcer	Gastrointestinal	Lung and bronchus	0.95	0.82	1.10	0.483	Radiotherapy alone
Ulcerative colitis	Gastrointestinal	Lung and bronchus	0.98	0.47	2.03	0.953	Radiotherapy alone
Agranulocytosis	Haematological	Lung and bronchus	1.25	0.60	2.60	0.548	Radiotherapy alone
Other anaemias	Haematological	Lung and bronchus	0.87	0.75	1.01	0.07	Radiotherapy alone
Primary or idiopathic thrombocytopaenia	Haematological	Lung and bronchus	1.00	0.51	1.97	0.996	Radiotherapy alone
Secondary or other thrombocytopaenia	Haematological	Lung and bronchus	1.03	0.63	1.68	0.909	Radiotherapy alone
Secondary polycythaemia	Haematological	Lung and bronchus	1.55	0.84	2.87	0.164	Radiotherapy alone
Thrombophilia	Haematological	Lung and bronchus	0.76	0.37	1.54	0.442	Radiotherapy alone
Viral diseases (excluding chronic hepatitis/HIV)	Immunology and infection	Lung and bronchus	0.73	0.54	0.98	0.035	Radiotherapy alone
Infection of bones and joints	Immunology and infection	Lung and bronchus	2.16	1.49	3.12	< 0.001	Radiotherapy alone
Autoimmune liver disease	Immunology and infection	Lung and bronchus	0.72	0.36	1.46	0.361	Radiotherapy alone
Chronic viral hepatitis	Immunology and infection	Lung and bronchus	0.94	0.53	1.67	0.836	Radiotherapy alone
Encephalitis	Immunology and infection	Lung and bronchus	0.80	0.39	1.64	0.536	Radiotherapy alone
Infection of anal and rectal regions	Immunology and infection	Lung and bronchus	0.57	0.25	1.30	0.182	Radiotherapy alone
Infection of liver	Immunology and infection	Lung and bronchus	0.80	0.38	1.66	0.542	Radiotherapy alone
Infection of skin and subcutaneous tissues	Immunology and infection	Lung and bronchus	0.86	0.70	1.07	0.176	Radiotherapy alone
Infections of other or unspecified organs	Immunology and infection	Lung and bronchus	0.89	0.77	1.03	0.13	Radiotherapy alone
Infections of the heart	Immunology and infection	Lung and bronchus	0.76	0.54	1.06	0.1	Radiotherapy alone
Infections of the digestive system	Immunology and infection	Lung and bronchus	0.86	0.70	1.05	0.132	Radiotherapy alone
Lower respiratory tract infections	Immunology and infection	Lung and bronchus	0.92	0.85	1.01	0.077	Radiotherapy alone
Meningitis	Immunology and infection	Lung and bronchus	0.81	0.59	1.12	0.211	Radiotherapy alone
Non-acute cystitis	Immunology and infection	Lung and bronchus	1.01	0.57	1.77	0.98	Radiotherapy alone
Peritonitis	Immunology and infection	Lung and bronchus	0.90	0.78	1.03	0.112	Radiotherapy alone
Osteoporosis	Musculoskeletal	Lung and bronchus	0.84	0.70	1.00	0.049	Radiotherapy alone
Enthesopathies and synovial disorders	Musculoskeletal	Lung and bronchus	1.20	1.03	1.41	0.02	Radiotherapy alone
Osteoarthritis (excluding spine)	Musculoskeletal	Lung and bronchus	0.95	0.83	1.07	0.398	Radiotherapy alone
Rheumatoid arthritis	Musculoskeletal	Lung and bronchus	0.92	0.67	1.27	0.623	Radiotherapy alone
Scoliosis	Musculoskeletal	Lung and bronchus	1.04	0.59	1.82	0.892	Radiotherapy alone
Spinal stenosis	Musculoskeletal	Lung and bronchus	1.14	0.80	1.63	0.466	Radiotherapy alone
Dementia	Neurological	Lung and bronchus	0.44	0.32	0.59	< 0.001	Radiotherapy alone
Disorders of autonomic nervous system	Neurological	Lung and bronchus	1.82	1.22	2.72	0.003	Radiotherapy alone
Bell's palsy	Neurological	Lung and bronchus	1.23	0.63	2.42	0.546	Radiotherapy alone
Epilepsy	Neurological	Lung and bronchus	1.45	0.98	2.15	0.063	Radiotherapy alone
Essential tremor	Neurological	Lung and bronchus	0.87	0.46	1.64	0.665	Radiotherapy alone
Intracerebral haemorrhage	Neurological	Lung and bronchus	0.90	0.63	1.28	0.562	Radiotherapy alone
Intracranial hypertension	Neurological	Lung and bronchus	0.79	0.49	1.26	0.315	Radiotherapy alone
Peripheral neuropathies	Neurological	Lung and bronchus	0.99	0.79	1.25	0.96	Radiotherapy alone
Subarachnoid haemorrhage	Neurological	Lung and bronchus	0.96	0.65	1.41	0.824	Radiotherapy alone
Other interstitial pulmonary diseases with fibrosis	Pulmonary	Lung and bronchus	0.53	0.41	0.69	< 0.001	Radiotherapy alone
Asthma	Pulmonary	Lung and bronchus	1.10	0.95	1.27	0.214	Radiotherapy alone
Bronchiectasis	Pulmonary	Lung and bronchus	0.80	0.58	1.11	0.186	Radiotherapy alone
COPD	Pulmonary	Lung and bronchus	1.07	0.97	1.17	0.18	Radiotherapy alone
End stage renal disease	Renal	Lung and bronchus	0.73	0.60	0.88	0.001	Radiotherapy alone
Glomerulonephritis	Renal	Lung and bronchus	0.86	0.59	1.26	0.435	Radiotherapy alone

Obstructive and reflux uropathy	Renal	Lung and bronchus	0.85	0.53	1.37	0.506	Radiotherapy alone
Urolithiasis	Renal	Lung and bronchus	0.86	0.63	1.18	0.349	Radiotherapy alone
Abdominal aortic aneurysm	Cardiovascular	Melanoma	2.58	1.47	4.54	0.001	Chemotherapy & surgery
Coronary heart disease not otherwise specified	Cardiovascular	Melanoma	1.01	0.70	1.46	0.967	Chemotherapy & surgery
Hypertension	Cardiovascular	Melanoma	0.95	0.70	1.30	0.752	Chemotherapy & surgery
Myocardial infarction	Cardiovascular	Melanoma	0.82	0.49	1.39	0.46	Chemotherapy & surgery
Other cardiomyopathy	Cardiovascular	Melanoma	1.20	0.75	1.92	0.45	Chemotherapy & surgery
Diabetes	Endocrine	Melanoma	1.21	0.81	1.81	0.347	Chemotherapy & surgery
Diabetic neurological complications	Endocrine	Melanoma	1.05	0.60	1.84	0.861	Chemotherapy & surgery
Diabetic ophthalmic complications	Endocrine	Melanoma	1.15	0.70	1.88	0.578	Chemotherapy & surgery
Diverticular disease of intestine	Gastrointestinal	Melanoma	0.71	0.37	1.36	0.302	Chemotherapy & surgery
Gastritis and duodenitis	Gastrointestinal	Melanoma	1.27	0.73	2.22	0.391	Chemotherapy & surgery
Gastrooesophageal reflux disease	Gastrointestinal	Melanoma	0.67	0.36	1.24	0.203	Chemotherapy & surgery
Oesophagitis and oesophageal ulcer	Gastrointestinal	Melanoma	0.70	0.40	1.24	0.218	Chemotherapy & surgery
Infections of other or unspecified organs	Immunology and infection	Melanoma	1.08	0.63	1.87	0.775	Chemotherapy & surgery
Lower respiratory tract infections	Immunology and infection	Melanoma	0.63	0.35	1.16	0.141	Chemotherapy & surgery
Peritonitis	Immunology and infection	Melanoma	0.88	0.52	1.47	0.616	Chemotherapy & surgery
Enthesopathies and synovial disorders	Musculoskeletal	Melanoma	1.34	0.94	1.92	0.103	Chemotherapy & surgery
Osteoarthritis (excluding spine)	Musculoskeletal	Melanoma	1.00	0.69	1.46	0.997	Chemotherapy & surgery
Hypertension	Cardiovascular	Melanoma	1.71	0.86	3.41	0.128	Chemotherapy alone
Hypertension	Cardiovascular	Melanoma	1.75	1.20	2.56	0.004	Chemotherapy, radiotherapy & surgery
Coronary heart disease not otherwise specified	Cardiovascular	Melanoma	0.88	0.49	1.58	0.662	Chemotherapy, radiotherapy & surgery
Diabetes	Endocrine	Melanoma	1.16	0.62	2.16	0.648	Chemotherapy, radiotherapy & surgery
Myocardial infarction	Cardiovascular	Melanoma	0.42	0.24	0.72	0.002	Radiotherapy & surgery
Other cardiomyopathy	Cardiovascular	Melanoma	0.61	0.38	0.98	0.041	Radiotherapy & surgery
Atrial fibrillation	Cardiovascular	Melanoma	0.77	0.51	1.17	0.224	Radiotherapy & surgery
Coronary heart disease not otherwise specified	Cardiovascular	Melanoma	1.18	0.90	1.55	0.234	Radiotherapy & surgery
Heart failure	Cardiovascular	Melanoma	1.09	0.71	1.68	0.701	Radiotherapy & surgery
Hypertension	Cardiovascular	Melanoma	0.89	0.70	1.14	0.357	Radiotherapy & surgery
Stable angina	Cardiovascular	Melanoma	0.68	0.40	1.13	0.134	Radiotherapy & surgery
Stroke (not otherwise specified)	Cardiovascular	Melanoma	1.10	0.64	1.90	0.727	Radiotherapy & surgery
Transient ischaemic attack	Cardiovascular	Melanoma	0.76	0.44	1.31	0.322	Radiotherapy & surgery
Unstable angina	Cardiovascular	Melanoma	0.83	0.51	1.35	0.462	Radiotherapy & surgery
Venous thromboembolic disease	Cardiovascular	Melanoma	0.99	0.57	1.72	0.975	Radiotherapy & surgery
Diabetes	Endocrine	Melanoma	0.82	0.56	1.20	0.311	Radiotherapy & surgery
Diabetic neurological complications	Endocrine	Melanoma	0.66	0.39	1.11	0.117	Radiotherapy & surgery
Diabetic ophthalmic complications	Endocrine	Melanoma	0.86	0.56	1.32	0.492	Radiotherapy & surgery
Cholecystitis	Gastrointestinal	Melanoma	1.15	0.63	2.10	0.649	Radiotherapy & surgery
Cholelithiasis	Gastrointestinal	Melanoma	1.16	0.68	2.00	0.58	Radiotherapy & surgery
Diverticular disease of intestine	Gastrointestinal	Melanoma	1.00	0.64	1.56	0.985	Radiotherapy & surgery
Gastritis and duodenitis	Gastrointestinal	Melanoma	1.45	0.95	2.22	0.087	Radiotherapy & surgery
Gastrooesophageal reflux disease	Gastrointestinal	Melanoma	0.71	0.43	1.15	0.161	Radiotherapy & surgery
Oesophagitis and oesophageal ulcer	Gastrointestinal	Melanoma	1.12	0.77	1.63	0.555	Radiotherapy & surgery
Other anaemias	Haematological	Melanoma	0.61	0.34	1.09	0.097	Radiotherapy & surgery
Infections of other or unspecified organs	Immunology and infection	Melanoma	1.18	0.76	1.84	0.452	Radiotherapy & surgery
Infections of the digestive system	Immunology and infection	Melanoma	1.36	0.74	2.50	0.328	Radiotherapy & surgery
Lower respiratory tract infections	Immunology and infection	Melanoma	0.81	0.51	1.27	0.358	Radiotherapy & surgery
Peritonitis	Immunology and infection	Melanoma	0.98	0.66	1.47	0.937	Radiotherapy & surgery
Enthesopathies and synovial disorders	Musculoskeletal	Melanoma	1.07	0.79	1.46	0.661	Radiotherapy & surgery
Osteoarthritis (excluding spine)	Musculoskeletal	Melanoma	0.99	0.73	1.33	0.932	Radiotherapy & surgery
Osteoporosis	Musculoskeletal	Melanoma	0.95	0.53	1.70	0.866	Radiotherapy & surgery
Asthma	Pulmonary	Melanoma	1.12	0.68	1.83	0.658	Radiotherapy & surgery
COPD	Pulmonary	Melanoma	0.85	0.49	1.49	0.57	Radiotherapy & surgery
End stage renal disease	Renal	Melanoma	1.02	0.56	1.85	0.953	Radiotherapy & surgery
Coronary heart disease not otherwise specified	Cardiovascular	Melanoma	1.72	0.89	3.31	0.107	Radiotherapy alone
Hypertension	Cardiovascular	Melanoma	1.04	0.56	1.91	0.903	Radiotherapy alone
Atrial fibrillation	Cardiovascular	Oesophagus	0.62	0.39	0.99	0.044	Chemotherapy & radiotherapy
Heart failure	Cardiovascular	Oesophagus	0.48	0.27	0.85	0.011	Chemotherapy & radiotherapy
Myocardial infarction	Cardiovascular	Oesophagus	0.57	0.37	0.87	0.009	Chemotherapy & radiotherapy
Other cardiomyopathy	Cardiovascular	Oesophagus	0.61	0.41	0.93	0.021	Chemotherapy & radiotherapy
Venous thromboembolic disease	Cardiovascular	Oesophagus	0.53	0.30	0.94	0.029	Chemotherapy & radiotherapy
Raynaud's syndrome	Cardiovascular	Oesophagus	2.00	1.13	3.56	0.018	Chemotherapy & radiotherapy
Coronary heart disease not otherwise specified	Cardiovascular	Oesophagus	0.77	0.55	1.09	0.14	Chemotherapy & radiotherapy
Hypertension	Cardiovascular	Oesophagus	0.88	0.68	1.14	0.335	Chemotherapy & radiotherapy
Peripheral arterial disease	Cardiovascular	Oesophagus	1.68	0.97	2.89	0.064	Chemotherapy & radiotherapy
Stable angina	Cardiovascular	Oesophagus	0.74	0.46	1.18	0.201	Chemotherapy & radiotherapy
Stroke (not otherwise specified)	Cardiovascular	Oesophagus	0.75	0.40	1.39	0.361	Chemotherapy & radiotherapy
Transient ischaemic attack	Cardiovascular	Oesophagus	0.88	0.53	1.48	0.636	Chemotherapy & radiotherapy
Unstable angina	Cardiovascular	Oesophagus	0.82	0.51	1.29	0.386	Chemotherapy & radiotherapy
Diabetes	Endocrine	Oesophagus	1.11	0.79	1.58	0.543	Chemotherapy & radiotherapy
Diabetic neurological complications	Endocrine	Oesophagus	0.98	0.65	1.47	0.921	Chemotherapy & radiotherapy
Diabetic ophthalmic complications	Endocrine	Oesophagus	1.04	0.71	1.52	0.839	Chemotherapy & radiotherapy
Hypo or hyperthyroidism	Endocrine	Oesophagus	1.01	0.56	1.82	0.978	Chemotherapy & radiotherapy
Barrett's oesophagus	Gastrointestinal	Oesophagus	0.62	0.48	0.80	< 0.001	Chemotherapy & radiotherapy
Gastritis and duodenitis	Gastrointestinal	Oesophagus	0.66	0.46	0.95	0.024	Chemotherapy & radiotherapy
Oesophagitis and oesophageal ulcer	Gastrointestinal	Oesophagus	0.80	0.64	1.00	0.047	Chemotherapy & radiotherapy
Cholangitis	Gastrointestinal	Oesophagus	0.69	0.35	1.36	0.278	Chemotherapy & radiotherapy
Cholecystitis	Gastrointestinal	Oesophagus	0.65	0.33	1.29	0.22	Chemotherapy & radiotherapy
Cholelithiasis	Gastrointestinal	Oesophagus	0.82	0.46	1.44	0.488	Chemotherapy & radiotherapy
Diverticular disease of intestine	Gastrointestinal	Oesophagus	0.77	0.49	1.21	0.25	Chemotherapy & radiotherapy
Gastrooesophageal reflux disease	Gastrointestinal	Oesophagus	1.14	0.85	1.51	0.386	Chemotherapy & radiotherapy

Other anaemias	Haematological	Oesophagus	0.53	0.34	0.83	0.005	Chemotherapy & radiotherapy
Lower respiratory tract infections	Immunology and infection	Oesophagus	0.58	0.41	0.82	0.002	Chemotherapy & radiotherapy
Infection of skin and subcutaneous tissues	Immunology and infection	Oesophagus	0.68	0.40	1.16	0.157	Chemotherapy & radiotherapy
Infections of other or unspecified organs	Immunology and infection	Oesophagus	0.73	0.49	1.08	0.112	Chemotherapy & radiotherapy
Infections of the digestive system	Immunology and infection	Oesophagus	0.71	0.43	1.19	0.192	Chemotherapy & radiotherapy
Peritonitis	Immunology and infection	Oesophagus	0.82	0.57	1.18	0.275	Chemotherapy & radiotherapy
Osteoarthritis (excluding spine)	Musculoskeletal	Oesophagus	1.37	1.00	1.87	0.049	Chemotherapy & radiotherapy
Enthesopathies and synovial disorders	Musculoskeletal	Oesophagus	0.83	0.55	1.24	0.354	Chemotherapy & radiotherapy
Osteoporosis	Musculoskeletal	Oesophagus	0.64	0.35	1.16	0.143	Chemotherapy & radiotherapy
Peripheral neuropathies	Neurological	Oesophagus	1.11	0.62	1.97	0.728	Chemotherapy & radiotherapy
Asthma	Pulmonary	Oesophagus	0.96	0.57	1.61	0.873	Chemotherapy & radiotherapy
COPD	Pulmonary	Oesophagus	1.17	0.83	1.67	0.373	Chemotherapy & radiotherapy
Atrial fibrillation	Cardiovascular	Oesophagus	0.63	0.41	0.98	0.04	Chemotherapy & surgery
Myocardial infarction	Cardiovascular	Oesophagus	0.65	0.44	0.96	0.029	Chemotherapy & surgery
Abdominal aortic aneurysm	Cardiovascular	Oesophagus	1.22	0.75	1.99	0.43	Chemotherapy & surgery
Coronary heart disease not otherwise specified	Cardiovascular	Oesophagus	1.17	0.87	1.57	0.294	Chemotherapy & surgery
Heart failure	Cardiovascular	Oesophagus	0.84	0.54	1.30	0.431	Chemotherapy & surgery
Hypertension	Cardiovascular	Oesophagus	1.06	0.84	1.34	0.631	Chemotherapy & surgery
Ischaemic stroke	Cardiovascular	Oesophagus	0.73	0.37	1.47	0.383	Chemotherapy & surgery
Other cardiomyopathy	Cardiovascular	Oesophagus	0.83	0.58	1.18	0.301	Chemotherapy & surgery
Peripheral arterial disease	Cardiovascular	Oesophagus	0.83	0.43	1.62	0.583	Chemotherapy & surgery
Raynaud's syndrome	Cardiovascular	Oesophagus	0.94	0.46	1.92	0.868	Chemotherapy & surgery
Stable angina	Cardiovascular	Oesophagus	0.83	0.53	1.28	0.391	Chemotherapy & surgery
Transient ischaemic attack	Cardiovascular	Oesophagus	1.02	0.64	1.64	0.921	Chemotherapy & surgery
Unstable angina	Cardiovascular	Oesophagus	0.72	0.45	1.14	0.16	Chemotherapy & surgery
Venous thromboembolic disease	Cardiovascular	Oesophagus	0.69	0.42	1.15	0.156	Chemotherapy & surgery
Diabetes	Endocrine	Oesophagus	1.48	1.09	2.02	0.013	Chemotherapy & surgery
Diabetic neurological complications	Endocrine	Oesophagus	1.07	0.73	1.57	0.724	Chemotherapy & surgery
Diabetic ophthalmic complications	Endocrine	Oesophagus	1.08	0.75	1.54	0.685	Chemotherapy & surgery
Hypo or hyperthyroidism	Endocrine	Oesophagus	1.11	0.62	1.99	0.725	Chemotherapy & surgery
Barrett's oesophagus	Gastrointestinal	Oesophagus	0.42	0.32	0.56	< 0.001	Chemotherapy & surgery
Gastritis and duodenitis	Gastrointestinal	Oesophagus	0.65	0.45	0.92	0.015	Chemotherapy & surgery
Oesophagitis and oesophageal ulcer	Gastrointestinal	Oesophagus	0.64	0.51	0.80	< 0.001	Chemotherapy & surgery
Cholangitis	Gastrointestinal	Oesophagus	1.01	0.56	1.85	0.965	Chemotherapy & surgery
Cholecystitis	Gastrointestinal	Oesophagus	0.93	0.51	1.70	0.82	Chemotherapy & surgery
Cholelithiasis	Gastrointestinal	Oesophagus	0.75	0.42	1.34	0.325	Chemotherapy & surgery
Diverticular disease of intestine	Gastrointestinal	Oesophagus	0.81	0.53	1.24	0.338	Chemotherapy & surgery
Fatty liver	Gastrointestinal	Oesophagus	0.72	0.37	1.43	0.352	Chemotherapy & surgery
Gastrooesophageal reflux disease	Gastrointestinal	Oesophagus	1.06	0.80	1.41	0.674	Chemotherapy & surgery
Other anaemias	Haematological	Oesophagus	0.84	0.59	1.21	0.361	Chemotherapy & surgery
Infections of the digestive system	Immunology and infection	Oesophagus	0.48	0.27	0.82	0.008	Chemotherapy & surgery
Lower respiratory tract infections	Immunology and infection	Oesophagus	0.48	0.33	0.68	< 0.001	Chemotherapy & surgery
Peritonitis	Immunology and infection	Oesophagus	0.69	0.48	0.99	0.047	Chemotherapy & surgery
Infection of skin and subcutaneous tissues	Immunology and infection	Oesophagus	0.66	0.39	1.12	0.126	Chemotherapy & surgery
Infections of other or unspecified organs	Immunology and infection	Oesophagus	0.69	0.47	1.03	0.067	Chemotherapy & surgery
Enthesopathies and synovial disorders	Musculoskeletal	Oesophagus	1.15	0.81	1.64	0.433	Chemotherapy & surgery
Osteoarthritis (excluding spine)	Musculoskeletal	Oesophagus	1.24	0.91	1.69	0.177	Chemotherapy & surgery
Peripheral neuropathies	Neurological	Oesophagus	1.16	0.66	2.02	0.602	Chemotherapy & surgery
Asthma	Pulmonary	Oesophagus	1.14	0.70	1.84	0.596	Chemotherapy & surgery
COPD	Pulmonary	Oesophagus	0.81	0.55	1.19	0.289	Chemotherapy & surgery
End stage renal disease	Renal	Oesophagus	0.36	0.19	0.69	0.002	Chemotherapy & surgery
Heart failure	Cardiovascular	Oesophagus	0.67	0.45	1.00	0.047	Chemotherapy alone
Venous thromboembolic disease	Cardiovascular	Oesophagus	0.45	0.28	0.72	0.001	Chemotherapy alone
Abdominal aortic aneurysm	Cardiovascular	Oesophagus	1.11	0.71	1.73	0.659	Chemotherapy alone
Atrial fibrillation	Cardiovascular	Oesophagus	0.84	0.60	1.16	0.294	Chemotherapy alone
Coronary heart disease not otherwise specified	Cardiovascular	Oesophagus	1.16	0.91	1.49	0.238	Chemotherapy alone
Hypertension	Cardiovascular	Oesophagus	1.07	0.88	1.30	0.517	Chemotherapy alone
Ischaemic stroke	Cardiovascular	Oesophagus	0.53	0.28	1.01	0.052	Chemotherapy alone
Myocardial infarction	Cardiovascular	Oesophagus	0.80	0.59	1.08	0.139	Chemotherapy alone
Nonrheumatic aortic valve disorders	Cardiovascular	Oesophagus	1.44	0.69	2.98	0.33	Chemotherapy alone
Other cardiomyopathy	Cardiovascular	Oesophagus	0.90	0.67	1.20	0.469	Chemotherapy alone
Peripheral arterial disease	Cardiovascular	Oesophagus	1.31	0.81	2.13	0.273	Chemotherapy alone
Raynaud's syndrome	Cardiovascular	Oesophagus	1.20	0.69	2.09	0.513	Chemotherapy alone
Stable angina	Cardiovascular	Oesophagus	1.01	0.72	1.41	0.975	Chemotherapy alone
Stroke (not otherwise specified)	Cardiovascular	Oesophagus	0.59	0.35	1.01	0.052	Chemotherapy alone
Transient ischaemic attack	Cardiovascular	Oesophagus	0.84	0.55	1.28	0.408	Chemotherapy alone
Unstable angina	Cardiovascular	Oesophagus	1.27	0.91	1.76	0.161	Chemotherapy alone
Diabetes	Endocrine	Oesophagus	1.26	0.96	1.67	0.097	Chemotherapy alone
Diabetic neurological complications	Endocrine	Oesophagus	1.32	0.97	1.80	0.08	Chemotherapy alone
Diabetic ophthalmic complications	Endocrine	Oesophagus	1.26	0.94	1.69	0.129	Chemotherapy alone
Hypo or hyperthyroidism	Endocrine	Oesophagus	0.67	0.38	1.17	0.16	Chemotherapy alone
Barrett's oesophagus	Gastrointestinal	Oesophagus	0.68	0.56	0.83	< 0.001	Chemotherapy alone
Hepatic failure	Gastrointestinal	Oesophagus	0.33	0.17	0.64	0.001	Chemotherapy alone
Oesophagitis and oesophageal ulcer	Gastrointestinal	Oesophagus	0.83	0.69	0.99	0.042	Chemotherapy alone
Alcoholic liver disease	Gastrointestinal	Oesophagus	0.64	0.27	1.49	0.302	Chemotherapy alone
Cholangitis	Gastrointestinal	Oesophagus	0.75	0.44	1.28	0.288	Chemotherapy alone
Cholecystitis	Gastrointestinal	Oesophagus	0.73	0.43	1.23	0.236	Chemotherapy alone
Cholelithiasis	Gastrointestinal	Oesophagus	0.79	0.50	1.26	0.332	Chemotherapy alone
Diverticular disease of intestine	Gastrointestinal	Oesophagus	1.03	0.74	1.44	0.859	Chemotherapy alone

Fatty liver	Gastrointestinal	Oesophagus	0.59	0.32	1.07	0.083	Chemotherapy alone
Gastritis and duodenitis	Gastrointestinal	Oesophagus	0.83	0.63	1.10	0.192	Chemotherapy alone
Gastrooesophageal reflux disease	Gastrointestinal	Oesophagus	0.99	0.77	1.26	0.908	Chemotherapy alone
Portal hypertension	Gastrointestinal	Oesophagus	0.52	0.24	1.12	0.092	Chemotherapy alone
Other anaemias	Haematological	Oesophagus	0.75	0.55	1.03	0.079	Chemotherapy alone
Infections of other or unspecified organs	Immunology and infection	Oesophagus	0.52	0.37	0.74	< 0.001	Chemotherapy alone
Infections of the heart	Immunology and infection	Oesophagus	0.42	0.20	0.89	0.023	Chemotherapy alone
Infections of the digestive system	Immunology and infection	Oesophagus	0.64	0.42	0.98	0.041	Chemotherapy alone
Lower respiratory tract infections	Immunology and infection	Oesophagus	0.54	0.41	0.72	< 0.001	Chemotherapy alone
Infection of skin and subcutaneous tissues	Immunology and infection	Oesophagus	0.66	0.42	1.02	0.06	Chemotherapy alone
Meningitis	Immunology and infection	Oesophagus	0.53	0.26	1.07	0.076	Chemotherapy alone
Peritonitis	Immunology and infection	Oesophagus	0.86	0.65	1.15	0.313	Chemotherapy alone
Enthesopathies and synovial disorders	Musculoskeletal	Oesophagus	1.10	0.81	1.49	0.544	Chemotherapy alone
Osteoarthritis (excluding spine)	Musculoskeletal	Oesophagus	1.12	0.86	1.47	0.395	Chemotherapy alone
Osteoporosis	Musculoskeletal	Oesophagus	0.76	0.48	1.20	0.242	Chemotherapy alone
Rheumatoid arthritis	Musculoskeletal	Oesophagus	0.81	0.39	1.71	0.584	Chemotherapy alone
Spinal stenosis	Musculoskeletal	Oesophagus	1.59	0.72	3.54	0.251	Chemotherapy alone
Peripheral neuropathies	Neurological	Oesophagus	0.93	0.56	1.53	0.773	Chemotherapy alone
Asthma	Pulmonary	Oesophagus	1.24	0.83	1.84	0.29	Chemotherapy alone
COPD	Pulmonary	Oesophagus	0.90	0.66	1.23	0.517	Chemotherapy alone
End stage renal disease	Renal	Oesophagus	0.72	0.47	1.10	0.127	Chemotherapy alone
Urolithiasis	Renal	Oesophagus	0.60	0.30	1.18	0.139	Chemotherapy alone
Atrial fibrillation	Cardiovascular	Oesophagus	0.52	0.27	0.97	0.04	Chemotherapy, radiotherapy & surgery
Coronary heart disease not otherwise specified	Cardiovascular	Oesophagus	1.37	0.96	1.96	0.08	Chemotherapy, radiotherapy & surgery
Hypertension	Cardiovascular	Oesophagus	0.93	0.68	1.27	0.638	Chemotherapy, radiotherapy & surgery
Myocardial infarction	Cardiovascular	Oesophagus	0.98	0.63	1.53	0.937	Chemotherapy, radiotherapy & surgery
Other cardiomyopathy	Cardiovascular	Oesophagus	0.99	0.63	1.55	0.968	Chemotherapy, radiotherapy & surgery
Stable angina	Cardiovascular	Oesophagus	1.39	0.86	2.24	0.179	Chemotherapy, radiotherapy & surgery
Unstable angina	Cardiovascular	Oesophagus	0.74	0.40	1.35	0.32	Chemotherapy, radiotherapy & surgery
Diabetes	Endocrine	Oesophagus	1.22	0.80	1.85	0.35	Chemotherapy, radiotherapy & surgery
Diabetic neurological complications	Endocrine	Oesophagus	1.44	0.92	2.27	0.112	Chemotherapy, radiotherapy & surgery
Diabetic ophthalmic complications	Endocrine	Oesophagus	1.47	0.96	2.26	0.076	Chemotherapy, radiotherapy & surgery
Barrett's oesophagus	Gastrointestinal	Oesophagus	0.62	0.45	0.86	0.004	Chemotherapy, radiotherapy & surgery
Diverticular disease of intestine	Gastrointestinal	Oesophagus	1.10	0.67	1.82	0.705	Chemotherapy, radiotherapy & surgery
Gastritis and duodenitis	Gastrointestinal	Oesophagus	0.64	0.40	1.01	0.057	Chemotherapy, radiotherapy & surgery
Gastrooesophageal reflux disease	Gastrointestinal	Oesophagus	1.09	0.77	1.56	0.618	Chemotherapy, radiotherapy & surgery
Oesophagitis and oesophageal ulcer	Gastrointestinal	Oesophagus	0.78	0.59	1.03	0.078	Chemotherapy, radiotherapy & surgery
Other anaemias	Haematological	Oesophagus	0.72	0.44	1.19	0.2	Chemotherapy, radiotherapy & surgery
Infection of skin and subcutaneous tissues	Immunology and infection	Oesophagus	0.74	0.38	1.44	0.381	Chemotherapy, radiotherapy & surgery
Infections of other or unspecified organs	Immunology and infection	Oesophagus	0.73	0.44	1.20	0.212	Chemotherapy, radiotherapy & surgery
Infections of the digestive system	Immunology and infection	Oesophagus	0.78	0.42	1.45	0.433	Chemotherapy, radiotherapy & surgery
Lower respiratory tract infections	Immunology and infection	Oesophagus	0.67	0.44	1.02	0.063	Chemotherapy, radiotherapy & surgery
Peritonitis	Immunology and infection	Oesophagus	0.84	0.53	1.32	0.442	Chemotherapy, radiotherapy & surgery
Enthesopathies and synovial disorders	Musculoskeletal	Oesophagus	1.55	1.03	2.33	0.034	Chemotherapy, radiotherapy & surgery
Osteoarthritis (excluding spine)	Musculoskeletal	Oesophagus	1.03	0.68	1.56	0.883	Chemotherapy, radiotherapy & surgery
Osteoporosis	Musculoskeletal	Oesophagus	0.96	0.50	1.86	0.908	Chemotherapy, radiotherapy & surgery
COPD	Pulmonary	Oesophagus	0.91	0.56	1.47	0.698	Chemotherapy, radiotherapy & surgery
Nonrheumatic aortic valve disorders	Cardiovascular	Oesophagus	2.76	1.38	5.52	0.004	Radiotherapy & surgery
Atrial fibrillation	Cardiovascular	Oesophagus	1.08	0.74	1.58	0.676	Radiotherapy & surgery
Coronary heart disease not otherwise specified	Cardiovascular	Oesophagus	1.09	0.77	1.54	0.641	Radiotherapy & surgery
Heart failure	Cardiovascular	Oesophagus	0.98	0.64	1.51	0.938	Radiotherapy & surgery
Hypertension	Cardiovascular	Oesophagus	1.03	0.78	1.35	0.853	Radiotherapy & surgery
Myocardial infarction	Cardiovascular	Oesophagus	0.79	0.52	1.18	0.248	Radiotherapy & surgery
Other cardiomyopathy	Cardiovascular	Oesophagus	0.83	0.56	1.23	0.355	Radiotherapy & surgery
Peripheral arterial disease	Cardiovascular	Oesophagus	1.09	0.55	2.16	0.802	Radiotherapy & surgery
Stable angina	Cardiovascular	Oesophagus	0.88	0.55	1.40	0.582	Radiotherapy & surgery
Stroke (not otherwise specified)	Cardiovascular	Oesophagus	0.74	0.39	1.38	0.339	Radiotherapy & surgery
Transient ischaemic attack	Cardiovascular	Oesophagus	1.20	0.74	1.93	0.464	Radiotherapy & surgery
Unstable angina	Cardiovascular	Oesophagus	1.13	0.73	1.75	0.578	Radiotherapy & surgery
Venous thromboembolic disease	Cardiovascular	Oesophagus	0.84	0.50	1.41	0.51	Radiotherapy & surgery
Diabetes	Endocrine	Oesophagus	1.12	0.75	1.68	0.577	Radiotherapy & surgery
Diabetic neurological complications	Endocrine	Oesophagus	0.77	0.48	1.25	0.288	Radiotherapy & surgery
Diabetic ophthalmic complications	Endocrine	Oesophagus	0.82	0.52	1.27	0.366	Radiotherapy & surgery
Hypo or hyperthyroidism	Endocrine	Oesophagus	0.81	0.42	1.58	0.537	Radiotherapy & surgery
Cholangitis	Gastrointestinal	Oesophagus	1.83	1.08	3.12	0.026	Radiotherapy & surgery
Cholecystitis	Gastrointestinal	Oesophagus	2.05	1.24	3.42	0.006	Radiotherapy & surgery
Cholelithiasis	Gastrointestinal	Oesophagus	1.87	1.16	3.02	0.011	Radiotherapy & surgery
Barrett's oesophagus	Gastrointestinal	Oesophagus	0.87	0.66	1.14	0.306	Radiotherapy & surgery
Diverticular disease of intestine	Gastrointestinal	Oesophagus	0.68	0.42	1.10	0.119	Radiotherapy & surgery
Gastritis and duodenitis	Gastrointestinal	Oesophagus	0.76	0.50	1.15	0.189	Radiotherapy & surgery
Gastrooesophageal reflux disease	Gastrointestinal	Oesophagus	1.25	0.90	1.73	0.178	Radiotherapy & surgery
Oesophagitis and oesophageal ulcer	Gastrointestinal	Oesophagus	1.15	0.91	1.46	0.245	Radiotherapy & surgery
Other anaemias	Haematological	Oesophagus	0.98	0.68	1.41	0.897	Radiotherapy & surgery
Peritonitis	Immunology and infection	Oesophagus	0.64	0.42	0.97	0.038	Radiotherapy & surgery
Infection of skin and subcutaneous tissues	Immunology and infection	Oesophagus	0.86	0.49	1.50	0.6	Radiotherapy & surgery
Infections of other or unspecified organs	Immunology and infection	Oesophagus	0.88	0.59	1.33	0.556	Radiotherapy & surgery
Lower respiratory tract infections	Immunology and infection	Oesophagus	0.84	0.60	1.18	0.316	Radiotherapy & surgery
Enthesopathies and synovial disorders	Musculoskeletal	Oesophagus	1.01	0.65	1.57	0.962	Radiotherapy & surgery
Osteoarthritis (excluding spine)	Musculoskeletal	Oesophagus	0.96	0.66	1.39	0.83	Radiotherapy & surgery
Osteoporosis	Musculoskeletal	Oesophagus	1.02	0.62	1.68	0.93	Radiotherapy & surgery
Asthma	Pulmonary	Oesophagus	0.97	0.55	1.72	0.91	Radiotherapy & surgery

COPD	Pulmonary	Oesophagus	1.38	0.95	2.00	0.092	Radiotherapy & surgery
End stage renal disease	Renal	Oesophagus	0.72	0.41	1.25	0.242	Radiotherapy & surgery
Coronary heart disease not otherwise specified	Cardiovascular	Oesophagus	1.36	1.07	1.73	0.013	Radiotherapy alone
Peripheral arterial disease	Cardiovascular	Oesophagus	1.70	1.09	2.66	0.02	Radiotherapy alone
Stable angina	Cardiovascular	Oesophagus	1.36	1.01	1.84	0.044	Radiotherapy alone
Unstable angina	Cardiovascular	Oesophagus	1.47	1.09	1.98	0.012	Radiotherapy alone
Abdominal aortic aneurysm	Cardiovascular	Oesophagus	0.63	0.36	1.11	0.111	Radiotherapy alone
Atrial fibrillation	Cardiovascular	Oesophagus	0.99	0.75	1.31	0.953	Radiotherapy alone
Atrioventricular block, complete	Cardiovascular	Oesophagus	0.61	0.33	1.11	0.105	Radiotherapy alone
Atrioventricular block, first degree	Cardiovascular	Oesophagus	0.61	0.33	1.11	0.105	Radiotherapy alone
Atrioventricular block, second degree	Cardiovascular	Oesophagus	0.61	0.33	1.11	0.105	Radiotherapy alone
Heart failure	Cardiovascular	Oesophagus	0.89	0.65	1.23	0.483	Radiotherapy alone
Hypertension	Cardiovascular	Oesophagus	1.09	0.89	1.33	0.402	Radiotherapy alone
Ischaemic stroke	Cardiovascular	Oesophagus	0.76	0.45	1.31	0.328	Radiotherapy alone
Left bundle branch block	Cardiovascular	Oesophagus	0.63	0.35	1.11	0.107	Radiotherapy alone
Multiple valve disease	Cardiovascular	Oesophagus	1.50	0.66	3.41	0.329	Radiotherapy alone
Myocardial infarction	Cardiovascular	Oesophagus	0.97	0.74	1.28	0.828	Radiotherapy alone
Nonrheumatic aortic valve disorders	Cardiovascular	Oesophagus	1.56	0.82	2.97	0.172	Radiotherapy alone
Nonrheumatic mitral valve disorders	Cardiovascular	Oesophagus	2.19	0.95	5.04	0.064	Radiotherapy alone
Other cardiomyopathy	Cardiovascular	Oesophagus	1.04	0.79	1.35	0.795	Radiotherapy alone
Raynaud's syndrome	Cardiovascular	Oesophagus	1.50	0.90	2.49	0.122	Radiotherapy alone
Stroke (not otherwise specified)	Cardiovascular	Oesophagus	0.96	0.63	1.45	0.831	Radiotherapy alone
Supraventricular tachycardia	Cardiovascular	Oesophagus	0.78	0.38	1.60	0.497	Radiotherapy alone
Transient ischaemic attack	Cardiovascular	Oesophagus	1.08	0.75	1.56	0.668	Radiotherapy alone
Venous thromboembolic disease	Cardiovascular	Oesophagus	0.93	0.65	1.34	0.709	Radiotherapy alone
Diabetes	Endocrine	Oesophagus	1.24	0.93	1.65	0.139	Radiotherapy alone
Diabetic neurological complications	Endocrine	Oesophagus	1.09	0.79	1.49	0.605	Radiotherapy alone
Diabetic ophthalmic complications	Endocrine	Oesophagus	1.15	0.86	1.54	0.358	Radiotherapy alone
Hypo or hyperthyroidism	Endocrine	Oesophagus	1.07	0.68	1.68	0.77	Radiotherapy alone
Barrett's oesophagus	Gastrointestinal	Oesophagus	0.59	0.48	0.73	< 0.001	Radiotherapy alone
Oesophagitis and oesophageal ulcer	Gastrointestinal	Oesophagus	0.68	0.57	0.83	< 0.001	Radiotherapy alone
Cholangitis	Gastrointestinal	Oesophagus	0.82	0.50	1.36	0.442	Radiotherapy alone
Cholecystitis	Gastrointestinal	Oesophagus	0.91	0.56	1.47	0.69	Radiotherapy alone
Cholelithiasis	Gastrointestinal	Oesophagus	0.93	0.60	1.44	0.748	Radiotherapy alone
Diverticular disease of intestine	Gastrointestinal	Oesophagus	0.82	0.59	1.14	0.242	Radiotherapy alone
Fatty liver	Gastrointestinal	Oesophagus	0.90	0.50	1.62	0.736	Radiotherapy alone
Gastritis and duodenitis	Gastrointestinal	Oesophagus	1.01	0.77	1.33	0.934	Radiotherapy alone
Gastrooesophageal reflux disease	Gastrointestinal	Oesophagus	1.00	0.78	1.29	0.985	Radiotherapy alone
Hepatic failure	Gastrointestinal	Oesophagus	0.76	0.43	1.35	0.354	Radiotherapy alone
Portal hypertension	Gastrointestinal	Oesophagus	0.94	0.47	1.87	0.852	Radiotherapy alone
Other anaemias	Haematological	Oesophagus	1.07	0.82	1.40	0.607	Radiotherapy alone
Infections of the digestive system	Immunology and infection	Oesophagus	0.56	0.36	0.87	0.009	Radiotherapy alone
Lower respiratory tract infections	Immunology and infection	Oesophagus	0.78	0.61	0.99	0.043	Radiotherapy alone
Viral diseases (excluding chronic hepatitis/HIV)	Immunology and infection	Oesophagus	1.26	0.78	2.04	0.353	Radiotherapy alone
Infection of skin and subcutaneous tissues	Immunology and infection	Oesophagus	0.80	0.53	1.21	0.29	Radiotherapy alone
Infections of other or unspecified organs	Immunology and infection	Oesophagus	0.75	0.55	1.03	0.076	Radiotherapy alone
Infections of the heart	Immunology and infection	Oesophagus	0.86	0.47	1.59	0.634	Radiotherapy alone
Meningitis	Immunology and infection	Oesophagus	0.81	0.44	1.51	0.512	Radiotherapy alone
Peritonitis	Immunology and infection	Oesophagus	0.76	0.57	1.01	0.057	Radiotherapy alone
Spinal stenosis	Musculoskeletal	Oesophagus	2.51	1.22	5.17	0.013	Radiotherapy alone
Enthesopathies and synovial disorders	Musculoskeletal	Oesophagus	1.20	0.88	1.63	0.253	Radiotherapy alone
Osteoarthritis (excluding spine)	Musculoskeletal	Oesophagus	1.00	0.76	1.31	0.976	Radiotherapy alone
Osteoporosis	Musculoskeletal	Oesophagus	0.91	0.62	1.34	0.635	Radiotherapy alone
Rheumatoid arthritis	Musculoskeletal	Oesophagus	0.97	0.49	1.94	0.94	Radiotherapy alone
Dementia	Neurological	Oesophagus	0.38	0.21	0.70	0.002	Radiotherapy alone
Peripheral neuropathies	Neurological	Oesophagus	1.25	0.79	1.98	0.35	Radiotherapy alone
Asthma	Pulmonary	Oesophagus	0.97	0.64	1.47	0.889	Radiotherapy alone
COPD	Pulmonary	Oesophagus	1.18	0.89	1.57	0.253	Radiotherapy alone
End stage renal disease	Renal	Oesophagus	0.65	0.43	0.98	0.038	Radiotherapy alone
Glomerulonephritis	Renal	Oesophagus	1.55	0.76	3.15	0.226	Radiotherapy alone
Urolithiasis	Renal	Oesophagus	0.75	0.39	1.46	0.399	Radiotherapy alone
Abdominal aortic aneurysm	Cardiovascular	Oropharynx	1.22	0.58	2.57	0.599	Chemotherapy & radiotherapy
Atrial fibrillation	Cardiovascular	Oropharynx	0.58	0.31	1.08	0.087	Chemotherapy & radiotherapy
Coronary heart disease not otherwise specified	Cardiovascular	Oropharynx	0.84	0.60	1.18	0.321	Chemotherapy & radiotherapy
Heart failure	Cardiovascular	Oropharynx	0.58	0.29	1.15	0.116	Chemotherapy & radiotherapy
Hypertension	Cardiovascular	Oropharynx	1.16	0.89	1.52	0.257	Chemotherapy & radiotherapy
Myocardial infarction	Cardiovascular	Oropharynx	0.87	0.55	1.40	0.573	Chemotherapy & radiotherapy
Other cardiomyopathy	Cardiovascular	Oropharynx	1.05	0.68	1.61	0.835	Chemotherapy & radiotherapy
Peripheral arterial disease	Cardiovascular	Oropharynx	0.64	0.35	1.16	0.14	Chemotherapy & radiotherapy
Raynaud's syndrome	Cardiovascular	Oropharynx	0.81	0.40	1.63	0.556	Chemotherapy & radiotherapy
Stable angina	Cardiovascular	Oropharynx	1.30	0.83	2.05	0.257	Chemotherapy & radiotherapy
Unstable angina	Cardiovascular	Oropharynx	1.46	0.91	2.35	0.116	Chemotherapy & radiotherapy
Diabetes	Endocrine	Oropharynx	0.90	0.61	1.32	0.585	Chemotherapy & radiotherapy
Diabetic neurological complications	Endocrine	Oropharynx	0.82	0.52	1.31	0.414	Chemotherapy & radiotherapy
Diabetic ophthalmic complications	Endocrine	Oropharynx	0.81	0.52	1.26	0.359	Chemotherapy & radiotherapy
Hypo or hyperthyroidism	Endocrine	Oropharynx	1.08	0.60	1.95	0.785	Chemotherapy & radiotherapy
Alcoholic liver disease	Gastrointestinal	Oropharynx	0.73	0.39	1.38	0.333	Chemotherapy & radiotherapy
Barrett's oesophagus	Gastrointestinal	Oropharynx	0.83	0.42	1.68	0.612	Chemotherapy & radiotherapy
Diverticular disease of intestine	Gastrointestinal	Oropharynx	0.89	0.52	1.52	0.665	Chemotherapy & radiotherapy

Fatty liver	Gastrointestinal	Oropharynx	0.76	0.43	1.34	0.338	Chemotherapy & radiotherapy
Gastritis and duodenitis	Gastrointestinal	Oropharynx	0.97	0.62	1.52	0.895	Chemotherapy & radiotherapy
Gastrooesophageal reflux disease	Gastrointestinal	Oropharynx	1.17	0.75	1.83	0.488	Chemotherapy & radiotherapy
Hepatic failure	Gastrointestinal	Oropharynx	0.77	0.42	1.42	0.408	Chemotherapy & radiotherapy
Liver fibrosis sclerosis and cirrhosis	Gastrointestinal	Oropharynx	0.67	0.34	1.32	0.242	Chemotherapy & radiotherapy
Oesophagitis and oesophageal ulcer	Gastrointestinal	Oropharynx	0.75	0.49	1.13	0.171	Chemotherapy & radiotherapy
Other anaemias	Haematological	Oropharynx	0.70	0.38	1.27	0.239	Chemotherapy & radiotherapy
Viral diseases (excluding chronic hepatitis/HIV)	Immunology and infection	Oropharynx	0.49	0.21	1.15	0.102	Chemotherapy & radiotherapy
Infection of skin and subcutaneous tissues	Immunology and infection	Oropharynx	1.10	0.66	1.85	0.713	Chemotherapy & radiotherapy
Infections of other or unspecified organs	Immunology and infection	Oropharynx	1.13	0.74	1.72	0.574	Chemotherapy & radiotherapy
Infections of the digestive system	Immunology and infection	Oropharynx	0.91	0.51	1.65	0.766	Chemotherapy & radiotherapy
Lower respiratory tract infections	Immunology and infection	Oropharynx	0.82	0.57	1.18	0.284	Chemotherapy & radiotherapy
Peritonitis	Immunology and infection	Oropharynx	1.05	0.68	1.61	0.83	Chemotherapy & radiotherapy
Enthesopathies and synovial disorders	Musculoskeletal	Oropharynx	0.96	0.65	1.42	0.84	Chemotherapy & radiotherapy
Osteoarthritis (excluding spine)	Musculoskeletal	Oropharynx	0.92	0.64	1.33	0.666	Chemotherapy & radiotherapy
Osteoporosis	Musculoskeletal	Oropharynx	0.79	0.41	1.52	0.477	Chemotherapy & radiotherapy
Peripheral neuropathies	Neurological	Oropharynx	1.10	0.58	2.10	0.771	Chemotherapy & radiotherapy
COPD	Pulmonary	Oropharynx	1.59	1.11	2.29	0.011	Chemotherapy & radiotherapy
Asthma	Pulmonary	Oropharynx	1.01	0.58	1.77	0.963	Chemotherapy & radiotherapy
End stage renal disease	Renal	Oropharynx	0.77	0.39	1.51	0.444	Chemotherapy & radiotherapy
Coronary heart disease not otherwise specified	Cardiovascular	Oropharynx	1.07	0.62	1.83	0.818	Chemotherapy & surgery
Hypertension	Cardiovascular	Oropharynx	0.73	0.44	1.22	0.232	Chemotherapy & surgery
Diabetes	Endocrine	Oropharynx	1.63	0.93	2.84	0.088	Chemotherapy & surgery
Diabetic neurological complications	Endocrine	Oropharynx	1.50	0.77	2.93	0.236	Chemotherapy & surgery
Diabetic ophthalmic complications	Endocrine	Oropharynx	1.16	0.59	2.29	0.673	Chemotherapy & surgery
Gastritis and duodenitis	Gastrointestinal	Oropharynx	1.59	0.83	3.06	0.163	Chemotherapy & surgery
Lower respiratory tract infections	Immunology and infection	Oropharynx	0.74	0.38	1.43	0.371	Chemotherapy & surgery
Enthesopathies and synovial disorders	Musculoskeletal	Oropharynx	1.43	0.79	2.59	0.242	Chemotherapy & surgery
COPD	Pulmonary	Oropharynx	1.22	0.63	2.36	0.563	Chemotherapy & surgery
Abdominal aortic aneurysm	Cardiovascular	Oropharynx	2.11	0.96	4.65	0.063	Chemotherapy alone
Coronary heart disease not otherwise specified	Cardiovascular	Oropharynx	0.62	0.36	1.06	0.082	Chemotherapy alone
Hypertension	Cardiovascular	Oropharynx	0.70	0.46	1.06	0.093	Chemotherapy alone
Myocardial infarction	Cardiovascular	Oropharynx	0.90	0.47	1.71	0.744	Chemotherapy alone
Other cardiomyopathy	Cardiovascular	Oropharynx	0.78	0.41	1.48	0.452	Chemotherapy alone
Stable angina	Cardiovascular	Oropharynx	1.14	0.59	2.22	0.698	Chemotherapy alone
Diverticular disease of intestine	Gastrointestinal	Oropharynx	1.12	0.56	2.23	0.759	Chemotherapy alone
Gastritis and duodenitis	Gastrointestinal	Oropharynx	1.24	0.68	2.26	0.482	Chemotherapy alone
Gastrooesophageal reflux disease	Gastrointestinal	Oropharynx	1.41	0.79	2.52	0.243	Chemotherapy alone
Oesophagitis and oesophageal ulcer	Gastrointestinal	Oropharynx	0.91	0.52	1.59	0.746	Chemotherapy alone
Infections of other or unspecified organs	Immunology and infection	Oropharynx	0.87	0.46	1.64	0.664	Chemotherapy alone
Lower respiratory tract infections	Immunology and infection	Oropharynx	0.73	0.43	1.24	0.244	Chemotherapy alone
Peritonitis	Immunology and infection	Oropharynx	1.04	0.58	1.87	0.894	Chemotherapy alone
Enthesopathies and synovial disorders	Musculoskeletal	Oropharynx	1.24	0.74	2.08	0.408	Chemotherapy alone
Osteoarthritis (excluding spine)	Musculoskeletal	Oropharynx	0.68	0.38	1.21	0.189	Chemotherapy alone
COPD	Pulmonary	Oropharynx	1.47	0.89	2.45	0.133	Chemotherapy alone
Coronary heart disease not otherwise specified	Cardiovascular	Oropharynx	0.82	0.56	1.21	0.325	Chemotherapy, radiotherapy & surgery
Hypertension	Cardiovascular	Oropharynx	1.00	0.73	1.38	0.975	Chemotherapy, radiotherapy & surgery
Myocardial infarction	Cardiovascular	Oropharynx	0.62	0.33	1.18	0.145	Chemotherapy, radiotherapy & surgery
Other cardiomyopathy	Cardiovascular	Oropharynx	0.80	0.46	1.39	0.42	Chemotherapy, radiotherapy & surgery
Stable angina	Cardiovascular	Oropharynx	0.90	0.50	1.62	0.725	Chemotherapy, radiotherapy & surgery
Transient ischaemic attack	Cardiovascular	Oropharynx	1.39	0.72	2.67	0.324	Chemotherapy, radiotherapy & surgery
Unstable angina	Cardiovascular	Oropharynx	1.02	0.56	1.89	0.94	Chemotherapy, radiotherapy & surgery
Diabetes	Endocrine	Oropharynx	1.08	0.70	1.67	0.724	Chemotherapy, radiotherapy & surgery
Diabetic neurological complications	Endocrine	Oropharynx	0.73	0.41	1.31	0.29	Chemotherapy, radiotherapy & surgery
Diabetic ophthalmic complications	Endocrine	Oropharynx	0.87	0.52	1.46	0.603	Chemotherapy, radiotherapy & surgery
Diverticular disease of intestine	Gastrointestinal	Oropharynx	0.98	0.53	1.83	0.95	Chemotherapy, radiotherapy & surgery
Fatty liver	Gastrointestinal	Oropharynx	0.69	0.34	1.40	0.305	Chemotherapy, radiotherapy & surgery
Gastritis and duodenitis	Gastrointestinal	Oropharynx	0.85	0.49	1.47	0.562	Chemotherapy, radiotherapy & surgery
Gastrooesophageal reflux disease	Gastrointestinal	Oropharynx	1.02	0.59	1.75	0.944	Chemotherapy, radiotherapy & surgery
Hepatic failure	Gastrointestinal	Oropharynx	0.80	0.39	1.63	0.538	Chemotherapy, radiotherapy & surgery
Oesophagitis and oesophageal ulcer	Gastrointestinal	Oropharynx	0.92	0.58	1.45	0.708	Chemotherapy, radiotherapy & surgery
Other anaemias	Haematological	Oropharynx	0.88	0.45	1.72	0.7	Chemotherapy, radiotherapy & surgery
Infections of other or unspecified organs	Immunology and infection	Oropharynx	0.52	0.27	1.00	0.049	Chemotherapy, radiotherapy & surgery
Infection of skin and subcutaneous tissues	Immunology and infection	Oropharynx	0.90	0.46	1.74	0.749	Chemotherapy, radiotherapy & surgery
Infections of the digestive system	Immunology and infection	Oropharynx	1.32	0.69	2.54	0.398	Chemotherapy, radiotherapy & surgery
Lower respiratory tract infections	Immunology and infection	Oropharynx	0.73	0.46	1.15	0.172	Chemotherapy, radiotherapy & surgery
Peritonitis	Immunology and infection	Oropharynx	1.28	0.79	2.06	0.319	Chemotherapy, radiotherapy & surgery
Enthesopathies and synovial disorders	Musculoskeletal	Oropharynx	1.50	1.00	2.23	0.047	Chemotherapy, radiotherapy & surgery
Osteoarthritis (excluding spine)	Musculoskeletal	Oropharynx	0.66	0.41	1.07	0.089	Chemotherapy, radiotherapy & surgery
Asthma	Pulmonary	Oropharynx	1.21	0.68	2.16	0.516	Chemotherapy, radiotherapy & surgery
COPD	Pulmonary	Oropharynx	1.12	0.71	1.78	0.621	Chemotherapy, radiotherapy & surgery
Abdominal aortic aneurysm	Cardiovascular	Oropharynx	0.78	0.38	1.59	0.493	Radiotherapy & surgery
Atrial fibrillation	Cardiovascular	Oropharynx	1.09	0.76	1.57	0.647	Radiotherapy & surgery
Coronary heart disease not otherwise specified	Cardiovascular	Oropharynx	0.90	0.70	1.16	0.417	Radiotherapy & surgery
Heart failure	Cardiovascular	Oropharynx	0.94	0.61	1.44	0.76	Radiotherapy & surgery
Hypertension	Cardiovascular	Oropharynx	1.13	0.92	1.39	0.244	Radiotherapy & surgery
Ischaemic stroke	Cardiovascular	Oropharynx	1.47	0.76	2.84	0.257	Radiotherapy & surgery
Myocardial infarction	Cardiovascular	Oropharynx	1.07	0.75	1.52	0.708	Radiotherapy & surgery
Other cardiomyopathy	Cardiovascular	Oropharynx	0.81	0.57	1.16	0.257	Radiotherapy & surgery
Peripheral arterial disease	Cardiovascular	Oropharynx	0.76	0.50	1.16	0.205	Radiotherapy & surgery
Raynaud's syndrome	Cardiovascular	Oropharynx	0.79	0.46	1.35	0.383	Radiotherapy & surgery

Stable angina	Cardiovascular	Oropharynx	0.75	0.51	1.12	0.166	Radiotherapy & surgery
Stroke (not otherwise specified)	Cardiovascular	Oropharynx	0.85	0.48	1.52	0.585	Radiotherapy & surgery
Transient ischaemic attack	Cardiovascular	Oropharynx	0.74	0.44	1.23	0.24	Radiotherapy & surgery
Unstable angina	Cardiovascular	Oropharynx	0.88	0.58	1.32	0.53	Radiotherapy & surgery
Venous thromboembolic disease	Cardiovascular	Oropharynx	1.41	0.86	2.33	0.172	Radiotherapy & surgery
Diabetes	Endocrine	Oropharynx	1.46	1.11	1.93	0.008	Radiotherapy & surgery
Diabetic neurological complications	Endocrine	Oropharynx	1.19	0.85	1.67	0.321	Radiotherapy & surgery
Diabetic ophthalmic complications	Endocrine	Oropharynx	1.18	0.86	1.63	0.313	Radiotherapy & surgery
Hypo or hyperthyroidism	Endocrine	Oropharynx	0.97	0.62	1.52	0.901	Radiotherapy & surgery
Alcoholic liver disease	Gastrointestinal	Oropharynx	0.95	0.58	1.57	0.843	Radiotherapy & surgery
Barrett's oesophagus	Gastrointestinal	Oropharynx	1.02	0.60	1.75	0.934	Radiotherapy & surgery
Cholangitis	Gastrointestinal	Oropharynx	1.07	0.62	1.84	0.816	Radiotherapy & surgery
Cholecystitis	Gastrointestinal	Oropharynx	0.98	0.57	1.70	0.956	Radiotherapy & surgery
Cholelithiasis	Gastrointestinal	Oropharynx	1.17	0.70	1.95	0.556	Radiotherapy & surgery
Diverticular disease of intestine	Gastrointestinal	Oropharynx	1.01	0.68	1.50	0.953	Radiotherapy & surgery
Fatty liver	Gastrointestinal	Oropharynx	0.89	0.56	1.44	0.646	Radiotherapy & surgery
Gastritis and duodenitis	Gastrointestinal	Oropharynx	1.20	0.86	1.69	0.281	Radiotherapy & surgery
Gastrooesophageal reflux disease	Gastrointestinal	Oropharynx	0.87	0.59	1.30	0.501	Radiotherapy & surgery
Hepatic failure	Gastrointestinal	Oropharynx	0.87	0.52	1.45	0.594	Radiotherapy & surgery
Liver fibrosis sclerosis and cirrhosis	Gastrointestinal	Oropharynx	1.08	0.65	1.80	0.765	Radiotherapy & surgery
Oesophagitis and oesophageal ulcer	Gastrointestinal	Oropharynx	0.85	0.61	1.18	0.324	Radiotherapy & surgery
Other anaemias	Haematological	Oropharynx	0.98	0.64	1.49	0.923	Radiotherapy & surgery
Infection of skin and subcutaneous tissues	Immunology and infection	Oropharynx	0.59	0.36	0.98	0.044	Radiotherapy & surgery
Lower respiratory tract infections	Immunology and infection	Oropharynx	0.71	0.52	0.95	0.023	Radiotherapy & surgery
Infections of other or unspecified organs	Immunology and infection	Oropharynx	0.73	0.50	1.06	0.098	Radiotherapy & surgery
Infections of the digestive system	Immunology and infection	Oropharynx	0.61	0.34	1.07	0.085	Radiotherapy & surgery
Peritonitis	Immunology and infection	Oropharynx	1.09	0.78	1.52	0.633	Radiotherapy & surgery
Enthesopathies and synovial disorders	Musculoskeletal	Oropharynx	1.32	0.99	1.78	0.061	Radiotherapy & surgery
Osteoarthritis (excluding spine)	Musculoskeletal	Oropharynx	1.08	0.82	1.41	0.588	Radiotherapy & surgery
Osteoporosis	Musculoskeletal	Oropharynx	0.98	0.63	1.50	0.913	Radiotherapy & surgery
Rheumatoid arthritis	Musculoskeletal	Oropharynx	1.15	0.54	2.46	0.72	Radiotherapy & surgery
Peripheral neuropathies	Neurological	Oropharynx	1.63	1.02	2.61	0.041	Radiotherapy & surgery
Asthma	Pulmonary	Oropharynx	0.90	0.58	1.38	0.62	Radiotherapy & surgery
COPD	Pulmonary	Oropharynx	1.17	0.86	1.59	0.321	Radiotherapy & surgery
End stage renal disease	Renal	Oropharynx	0.85	0.51	1.42	0.547	Radiotherapy & surgery
Abdominal aortic aneurysm	Cardiovascular	Oropharynx	0.76	0.39	1.47	0.413	Radiotherapy alone
Atrial fibrillation	Cardiovascular	Oropharynx	1.13	0.79	1.60	0.506	Radiotherapy alone
Atrioventricular block, complete	Cardiovascular	Oropharynx	0.91	0.44	1.87	0.795	Radiotherapy alone
Atrioventricular block, first degree	Cardiovascular	Oropharynx	0.91	0.44	1.87	0.795	Radiotherapy alone
Atrioventricular block, second degree	Cardiovascular	Oropharynx	0.91	0.44	1.87	0.795	Radiotherapy alone
Coronary heart disease not otherwise specified	Cardiovascular	Oropharynx	1.04	0.81	1.33	0.754	Radiotherapy alone
Heart failure	Cardiovascular	Oropharynx	1.05	0.71	1.56	0.802	Radiotherapy alone
Hypertension	Cardiovascular	Oropharynx	1.06	0.86	1.32	0.563	Radiotherapy alone
Ischaemic stroke	Cardiovascular	Oropharynx	1.20	0.62	2.32	0.597	Radiotherapy alone
Left bundle branch block	Cardiovascular	Oropharynx	0.83	0.41	1.67	0.596	Radiotherapy alone
Myocardial infarction	Cardiovascular	Oropharynx	1.11	0.80	1.55	0.523	Radiotherapy alone
Nonrheumatic mitral valve disorders	Cardiovascular	Oropharynx	1.17	0.51	2.70	0.715	Radiotherapy alone
Other cardiomyopathy	Cardiovascular	Oropharynx	1.10	0.80	1.51	0.557	Radiotherapy alone
Peripheral arterial disease	Cardiovascular	Oropharynx	1.28	0.89	1.84	0.187	Radiotherapy alone
Raynaud's syndrome	Cardiovascular	Oropharynx	1.22	0.77	1.95	0.396	Radiotherapy alone
Stable angina	Cardiovascular	Oropharynx	0.86	0.59	1.25	0.439	Radiotherapy alone
Stroke (not otherwise specified)	Cardiovascular	Oropharynx	1.09	0.65	1.82	0.743	Radiotherapy alone
Transient ischaemic attack	Cardiovascular	Oropharynx	0.81	0.50	1.28	0.362	Radiotherapy alone
Unstable angina	Cardiovascular	Oropharynx	1.11	0.77	1.62	0.566	Radiotherapy alone
Venous thromboembolic disease	Cardiovascular	Oropharynx	1.03	0.61	1.74	0.905	Radiotherapy alone
Diabetes	Endocrine	Oropharynx	0.96	0.71	1.30	0.803	Radiotherapy alone
Diabetic neurological complications	Endocrine	Oropharynx	1.02	0.73	1.42	0.918	Radiotherapy alone
Diabetic ophthalmic complications	Endocrine	Oropharynx	0.96	0.70	1.33	0.823	Radiotherapy alone
Hypo or hyperthyroidism	Endocrine	Oropharynx	0.81	0.51	1.29	0.365	Radiotherapy alone
Cholangitis	Gastrointestinal	Oropharynx	0.46	0.24	0.89	0.02	Radiotherapy alone
Cholecystitis	Gastrointestinal	Oropharynx	0.41	0.21	0.80	0.009	Radiotherapy alone
Cholelithiasis	Gastrointestinal	Oropharynx	0.43	0.22	0.83	0.012	Radiotherapy alone
Alcoholic liver disease	Gastrointestinal	Oropharynx	0.71	0.42	1.21	0.209	Radiotherapy alone
Barrett's oesophagus	Gastrointestinal	Oropharynx	1.04	0.62	1.75	0.885	Radiotherapy alone
Diverticular disease of intestine	Gastrointestinal	Oropharynx	0.92	0.63	1.36	0.691	Radiotherapy alone
Fatty liver	Gastrointestinal	Oropharynx	0.73	0.45	1.19	0.212	Radiotherapy alone
Gastritis and duodenitis	Gastrointestinal	Oropharynx	1.27	0.91	1.77	0.158	Radiotherapy alone
Gastrooesophageal reflux disease	Gastrointestinal	Oropharynx	1.00	0.69	1.45	0.991	Radiotherapy alone
Hepatic failure	Gastrointestinal	Oropharynx	0.86	0.52	1.42	0.562	Radiotherapy alone
Liver fibrosis sclerosis and cirrhosis	Gastrointestinal	Oropharynx	0.91	0.53	1.55	0.723	Radiotherapy alone
Oesophagitis and oesophageal ulcer	Gastrointestinal	Oropharynx	1.03	0.75	1.40	0.863	Radiotherapy alone
Portal hypertension	Gastrointestinal	Oropharynx	0.98	0.49	1.97	0.964	Radiotherapy alone
Other anaemias	Haematological	Oropharynx	1.63	1.14	2.34	0.008	Radiotherapy alone
Viral diseases (excluding chronic hepatitis/HIV)	Immunology and infection	Oropharynx	1.26	0.78	2.04	0.353	Radiotherapy alone
Infection of skin and subcutaneous tissues	Immunology and infection	Oropharynx	1.07	0.71	1.61	0.762	Radiotherapy alone
Infections of other or unspecified organs	Immunology and infection	Oropharynx	1.16	0.84	1.60	0.375	Radiotherapy alone
Infections of the heart	Immunology and infection	Oropharynx	0.58	0.30	1.15	0.118	Radiotherapy alone
Infections of the digestive system	Immunology and infection	Oropharynx	1.04	0.66	1.64	0.87	Radiotherapy alone
Lower respiratory tract infections	Immunology and infection	Oropharynx	1.05	0.80	1.36	0.733	Radiotherapy alone
Meningitis	Immunology and infection	Oropharynx	0.62	0.30	1.29	0.202	Radiotherapy alone
Peritonitis	Immunology and infection	Oropharynx	1.20	0.87	1.65	0.264	Radiotherapy alone

Enthesopathies and synovial disorders	Musculoskeletal	Oropharynx	1.02	0.74	1.40	0.915	Radiotherapy alone
Osteoarthritis (excluding spine)	Musculoskeletal	Oropharynx	0.83	0.62	1.10	0.193	Radiotherapy alone
Osteoporosis	Musculoskeletal	Oropharynx	0.89	0.58	1.38	0.614	Radiotherapy alone
Dementia	Neurological	Oropharynx	1.06	0.57	1.95	0.861	Radiotherapy alone
Epilepsy	Neurological	Oropharynx	0.84	0.41	1.68	0.616	Radiotherapy alone
Intracerebral haemorrhage	Neurological	Oropharynx	1.24	0.52	2.97	0.623	Radiotherapy alone
Peripheral neuropathies	Neurological	Oropharynx	1.00	0.59	1.71	0.995	Radiotherapy alone
Subarachnoid haemorrhage	Neurological	Oropharynx	1.38	0.54	3.54	0.505	Radiotherapy alone
Asthma	Pulmonary	Oropharynx	1.70	1.17	2.49	0.006	Radiotherapy alone
COPD	Pulmonary	Oropharynx	1.41	1.05	1.88	0.02	Radiotherapy alone
End stage renal disease	Renal	Oropharynx	0.88	0.54	1.42	0.589	Radiotherapy alone
Urolithiasis	Renal	Oropharynx	0.82	0.36	1.88	0.643	Radiotherapy alone
Oesophagitis and oesophageal ulcer	Gastrointestinal	Ovary	2.51	1.32	4.75	0.005	Chemotherapy & radiotherapy
Atrial fibrillation	Cardiovascular	Ovary	0.61	0.39	0.94	0.026	Chemotherapy & surgery
Coronary heart disease not otherwise specified	Cardiovascular	Ovary	1.07	0.84	1.37	0.581	Chemotherapy & surgery
Heart failure	Cardiovascular	Ovary	0.89	0.56	1.42	0.624	Chemotherapy & surgery
Hypertension	Cardiovascular	Ovary	0.96	0.79	1.17	0.68	Chemotherapy & surgery
Myocardial infarction	Cardiovascular	Ovary	1.03	0.67	1.57	0.894	Chemotherapy & surgery
Other cardiomyopathy	Cardiovascular	Ovary	0.79	0.52	1.20	0.272	Chemotherapy & surgery
Peripheral arterial disease	Cardiovascular	Ovary	0.64	0.32	1.26	0.196	Chemotherapy & surgery
Raynaud's syndrome	Cardiovascular	Ovary	0.89	0.45	1.74	0.727	Chemotherapy & surgery
Stable angina	Cardiovascular	Ovary	0.91	0.61	1.35	0.649	Chemotherapy & surgery
Stroke (not otherwise specified)	Cardiovascular	Ovary	0.60	0.30	1.22	0.157	Chemotherapy & surgery
Supraventricular tachycardia	Cardiovascular	Ovary	2.04	0.89	4.68	0.092	Chemotherapy & surgery
Transient ischaemic attack	Cardiovascular	Ovary	1.12	0.64	1.95	0.69	Chemotherapy & surgery
Unstable angina	Cardiovascular	Ovary	1.01	0.67	1.54	0.954	Chemotherapy & surgery
Venous thromboembolic disease	Cardiovascular	Ovary	0.98	0.66	1.45	0.918	Chemotherapy & surgery
Ventricular tachycardia	Cardiovascular	Ovary	2.04	0.89	4.68	0.092	Chemotherapy & surgery
Diabetes	Endocrine	Ovary	1.00	0.74	1.34	0.991	Chemotherapy & surgery
Diabetic neurological complications	Endocrine	Ovary	1.02	0.73	1.43	0.905	Chemotherapy & surgery
Diabetic ophthalmic complications	Endocrine	Ovary	1.08	0.78	1.49	0.635	Chemotherapy & surgery
Hypo or hyperthyroidism	Endocrine	Ovary	1.03	0.74	1.42	0.872	Chemotherapy & surgery
Diverticular disease of intestine	Gastrointestinal	Ovary	1.35	1.02	1.80	0.038	Chemotherapy & surgery
Barrett's oesophagus	Gastrointestinal	Ovary	0.82	0.42	1.60	0.558	Chemotherapy & surgery
Cholangitis	Gastrointestinal	Ovary	1.20	0.77	1.85	0.418	Chemotherapy & surgery
Cholecystitis	Gastrointestinal	Ovary	1.24	0.82	1.87	0.31	Chemotherapy & surgery
Cholelithiasis	Gastrointestinal	Ovary	1.16	0.80	1.69	0.426	Chemotherapy & surgery
Fatty liver	Gastrointestinal	Ovary	0.83	0.45	1.52	0.543	Chemotherapy & surgery
Gastritis and duodenitis	Gastrointestinal	Ovary	1.26	0.90	1.76	0.179	Chemotherapy & surgery
Gastrooesophageal reflux disease	Gastrointestinal	Ovary	1.26	0.90	1.77	0.183	Chemotherapy & surgery
Hepatic failure	Gastrointestinal	Ovary	0.74	0.40	1.38	0.347	Chemotherapy & surgery
Oesophagitis and oesophageal ulcer	Gastrointestinal	Ovary	1.18	0.88	1.59	0.265	Chemotherapy & surgery
Portal hypertension	Gastrointestinal	Ovary	0.60	0.30	1.21	0.152	Chemotherapy & surgery
Other anaemias	Haematological	Ovary	0.77	0.56	1.05	0.093	Chemotherapy & surgery
Peritonitis	Immunology and infection	Ovary	1.35	1.03	1.76	0.028	Chemotherapy & surgery
Infection of skin and subcutaneous tissues	Immunology and infection	Ovary	0.80	0.48	1.33	0.384	Chemotherapy & surgery
Infections of other or unspecified organs	Immunology and infection	Ovary	0.82	0.59	1.14	0.237	Chemotherapy & surgery
Infections of the digestive system	Immunology and infection	Ovary	1.07	0.70	1.65	0.744	Chemotherapy & surgery
Lower respiratory tract infections	Immunology and infection	Ovary	0.94	0.68	1.29	0.687	Chemotherapy & surgery
Enthesopathies and synovial disorders	Musculoskeletal	Ovary	0.95	0.71	1.27	0.71	Chemotherapy & surgery
Osteoarthritis (excluding spine)	Musculoskeletal	Ovary	1.05	0.82	1.34	0.684	Chemotherapy & surgery
Osteoporosis	Musculoskeletal	Ovary	1.01	0.69	1.47	0.973	Chemotherapy & surgery
Rheumatoid arthritis	Musculoskeletal	Ovary	0.80	0.38	1.67	0.548	Chemotherapy & surgery
Peripheral neuropathies	Neurological	Ovary	1.55	0.95	2.54	0.08	Chemotherapy & surgery
Asthma	Pulmonary	Ovary	0.82	0.58	1.17	0.272	Chemotherapy & surgery
COPD	Pulmonary	Ovary	0.73	0.48	1.10	0.137	Chemotherapy & surgery
End stage renal disease	Renal	Ovary	1.06	0.61	1.84	0.837	Chemotherapy & surgery
Obstructive and reflux uropathy	Renal	Ovary	0.93	0.57	1.51	0.761	Chemotherapy & surgery
Urolithiasis	Renal	Ovary	0.83	0.52	1.33	0.438	Chemotherapy & surgery
Atrial fibrillation	Cardiovascular	Ovary	0.66	0.46	0.94	0.023	Chemotherapy alone
Coronary heart disease not otherwise specified	Cardiovascular	Ovary	0.73	0.56	0.94	0.014	Chemotherapy alone
Stroke (not otherwise specified)	Cardiovascular	Ovary	0.50	0.26	0.94	0.033	Chemotherapy alone
Heart failure	Cardiovascular	Ovary	0.80	0.53	1.20	0.279	Chemotherapy alone
Hypertension	Cardiovascular	Ovary	0.84	0.69	1.01	0.068	Chemotherapy alone
Ischaemic stroke	Cardiovascular	Ovary	0.73	0.34	1.56	0.413	Chemotherapy alone
Multiple valve disease	Cardiovascular	Ovary	1.15	0.49	2.71	0.742	Chemotherapy alone
Myocardial infarction	Cardiovascular	Ovary	0.84	0.57	1.24	0.378	Chemotherapy alone
Nonrheumatic aortic valve disorders	Cardiovascular	Ovary	0.96	0.48	1.89	0.896	Chemotherapy alone
Nonrheumatic mitral valve disorders	Cardiovascular	Ovary	0.91	0.38	2.15	0.821	Chemotherapy alone
Other cardiomyopathy	Cardiovascular	Ovary	0.76	0.52	1.09	0.138	Chemotherapy alone
Peripheral arterial disease	Cardiovascular	Ovary	0.65	0.36	1.18	0.155	Chemotherapy alone
Raynaud's syndrome	Cardiovascular	Ovary	0.78	0.41	1.47	0.44	Chemotherapy alone
Stable angina	Cardiovascular	Ovary	0.74	0.50	1.09	0.123	Chemotherapy alone
Transient ischaemic attack	Cardiovascular	Ovary	0.83	0.49	1.41	0.486	Chemotherapy alone
Unstable angina	Cardiovascular	Ovary	0.77	0.52	1.16	0.217	Chemotherapy alone
Venous thromboembolic disease	Cardiovascular	Ovary	1.14	0.81	1.61	0.462	Chemotherapy alone
Diabetes	Endocrine	Ovary	1.11	0.85	1.46	0.426	Chemotherapy alone
Diabetic neurological complications	Endocrine	Ovary	1.27	0.95	1.70	0.111	Chemotherapy alone
Diabetic ophthalmic complications	Endocrine	Ovary	1.24	0.94	1.65	0.132	Chemotherapy alone

Hypo or hyperthyroidism	Endocrine	Ovary	0.87	0.63	1.19	0.368	Chemotherapy alone
Gastrooesophageal reflux disease	Gastrointestinal	Ovary	1.38	1.01	1.89	0.044	Chemotherapy alone
Barrett's oesophagus	Gastrointestinal	Ovary	0.75	0.42	1.35	0.335	Chemotherapy alone
Cholangitis	Gastrointestinal	Ovary	1.19	0.79	1.78	0.409	Chemotherapy alone
Cholecystitis	Gastrointestinal	Ovary	1.22	0.83	1.79	0.321	Chemotherapy alone
Cholelithiasis	Gastrointestinal	Ovary	1.00	0.70	1.44	0.986	Chemotherapy alone
Diverticular disease of intestine	Gastrointestinal	Ovary	1.23	0.94	1.61	0.134	Chemotherapy alone
Fatty liver	Gastrointestinal	Ovary	1.20	0.73	1.97	0.481	Chemotherapy alone
Gastritis and duodenitis	Gastrointestinal	Ovary	1.12	0.81	1.54	0.502	Chemotherapy alone
Hepatic failure	Gastrointestinal	Ovary	1.01	0.61	1.69	0.962	Chemotherapy alone
Oesophagitis and oesophageal ulcer	Gastrointestinal	Ovary	1.12	0.85	1.48	0.419	Chemotherapy alone
Portal hypertension	Gastrointestinal	Ovary	1.08	0.63	1.83	0.785	Chemotherapy alone
Other anaemias	Haematological	Ovary	0.75	0.56	1.00	0.047	Chemotherapy alone
Infections of other or unspecified organs	Immunology and infection	Ovary	0.65	0.47	0.89	0.008	Chemotherapy alone
Infection of skin and subcutaneous tissues	Immunology and infection	Ovary	1.17	0.76	1.81	0.466	Chemotherapy alone
Infections of the heart	Immunology and infection	Ovary	2.02	0.88	4.65	0.098	Chemotherapy alone
Infections of the digestive system	Immunology and infection	Ovary	1.22	0.83	1.80	0.301	Chemotherapy alone
Lower respiratory tract infections	Immunology and infection	Ovary	1.10	0.83	1.45	0.523	Chemotherapy alone
Meningitis	Immunology and infection	Ovary	1.48	0.70	3.11	0.305	Chemotherapy alone
Peritonitis	Immunology and infection	Ovary	1.11	0.86	1.43	0.407	Chemotherapy alone
Viral diseases (excluding chronic hepatitis/HIV)	Immunology and infection	Ovary	0.77	0.36	1.65	0.508	Chemotherapy alone
Enthesopathies and synovial disorders	Musculoskeletal	Ovary	1.17	0.89	1.53	0.251	Chemotherapy alone
Osteoarthritis (excluding spine)	Musculoskeletal	Ovary	1.09	0.87	1.37	0.435	Chemotherapy alone
Osteoporosis	Musculoskeletal	Ovary	0.75	0.52	1.07	0.116	Chemotherapy alone
Rheumatoid arthritis	Musculoskeletal	Ovary	1.30	0.70	2.41	0.407	Chemotherapy alone
Peripheral neuropathies	Neurological	Ovary	1.16	0.70	1.91	0.574	Chemotherapy alone
Asthma	Pulmonary	Ovary	0.65	0.46	0.93	0.018	Chemotherapy alone
COPD	Pulmonary	Ovary	0.93	0.65	1.33	0.698	Chemotherapy alone
End stage renal disease	Renal	Ovary	1.40	0.87	2.26	0.169	Chemotherapy alone
Obstructive and reflux uropathy	Renal	Ovary	1.16	0.75	1.78	0.503	Chemotherapy alone
Urolithiasis	Renal	Ovary	1.08	0.72	1.61	0.727	Chemotherapy alone
Hypertension	Cardiovascular	Ovary	0.71	0.39	1.28	0.258	Chemotherapy, radiotherapy & surgery
Diabetes	Endocrine	Ovary	1.83	0.95	3.51	0.071	Chemotherapy, radiotherapy & surgery
Osteoarthritis (excluding spine)	Musculoskeletal	Ovary	1.60	0.91	2.83	0.106	Chemotherapy, radiotherapy & surgery
Coronary heart disease not otherwise specified	Cardiovascular	Ovary	1.43	0.80	2.56	0.234	Radiotherapy & surgery
Hypertension	Cardiovascular	Ovary	1.17	0.72	1.91	0.524	Radiotherapy & surgery
Osteoarthritis (excluding spine)	Musculoskeletal	Ovary	1.16	0.61	2.22	0.649	Radiotherapy & surgery
Atrial fibrillation	Cardiovascular	Ovary	3.23	1.54	6.79	0.002	Radiotherapy alone
Hypertension	Cardiovascular	Pancreas	1.00	0.61	1.66	0.989	Chemotherapy & radiotherapy
Diabetes	Endocrine	Pancreas	1.22	0.77	1.93	0.403	Chemotherapy & radiotherapy
Diabetic neurological complications	Endocrine	Pancreas	0.94	0.56	1.58	0.814	Chemotherapy & radiotherapy
Diabetic ophthalmic complications	Endocrine	Pancreas	0.93	0.55	1.56	0.78	Chemotherapy & radiotherapy
Cholangitis	Gastrointestinal	Pancreas	0.61	0.40	0.94	0.024	Chemotherapy & radiotherapy
Gastritis and duodenitis	Gastrointestinal	Pancreas	1.32	0.78	2.21	0.298	Chemotherapy & radiotherapy
Gastrooesophageal reflux disease	Gastrointestinal	Pancreas	1.29	0.69	2.41	0.424	Chemotherapy & radiotherapy
Oesophagitis and oesophageal ulcer	Gastrointestinal	Pancreas	1.06	0.60	1.86	0.847	Chemotherapy & radiotherapy
Peritonitis	Immunology and infection	Pancreas	0.91	0.48	1.72	0.779	Chemotherapy & radiotherapy
Osteoarthritis (excluding spine)	Musculoskeletal	Pancreas	1.89	1.09	3.28	0.023	Chemotherapy & radiotherapy
Enthesopathies and synovial disorders	Musculoskeletal	Pancreas	1.10	0.56	2.14	0.788	Chemotherapy & radiotherapy
Coronary heart disease not otherwise specified	Cardiovascular	Pancreas	0.66	0.44	0.99	0.042	Chemotherapy & surgery
Abdominal aortic aneurysm	Cardiovascular	Pancreas	1.06	0.52	2.19	0.867	Chemotherapy & surgery
Atrial fibrillation	Cardiovascular	Pancreas	0.83	0.51	1.35	0.442	Chemotherapy & surgery
Hypertension	Cardiovascular	Pancreas	1.08	0.83	1.42	0.563	Chemotherapy & surgery
Myocardial infarction	Cardiovascular	Pancreas	0.66	0.40	1.09	0.102	Chemotherapy & surgery
Other cardiomyopathy	Cardiovascular	Pancreas	0.76	0.48	1.19	0.224	Chemotherapy & surgery
Stable angina	Cardiovascular	Pancreas	1.06	0.65	1.75	0.809	Chemotherapy & surgery
Unstable angina	Cardiovascular	Pancreas	1.07	0.64	1.79	0.79	Chemotherapy & surgery
Venous thromboembolic disease	Cardiovascular	Pancreas	0.64	0.38	1.08	0.096	Chemotherapy & surgery
Diabetes	Endocrine	Pancreas	1.11	0.85	1.45	0.427	Chemotherapy & surgery
Diabetic neurological complications	Endocrine	Pancreas	1.08	0.82	1.43	0.58	Chemotherapy & surgery
Diabetic ophthalmic complications	Endocrine	Pancreas	1.02	0.77	1.35	0.9	Chemotherapy & surgery
Hypo or hyperthyroidism	Endocrine	Pancreas	0.81	0.43	1.54	0.523	Chemotherapy & surgery
Barrett's oesophagus	Gastrointestinal	Pancreas	0.48	0.25	0.94	0.032	Chemotherapy & surgery
Cholangitis	Gastrointestinal	Pancreas	1.34	1.09	1.66	0.006	Chemotherapy & surgery
Cholecystitis	Gastrointestinal	Pancreas	0.94	0.69	1.30	0.725	Chemotherapy & surgery
Cholelithiasis	Gastrointestinal	Pancreas	0.83	0.59	1.17	0.285	Chemotherapy & surgery
Diverticular disease of intestine	Gastrointestinal	Pancreas	1.36	0.92	2.00	0.125	Chemotherapy & surgery
Fatty liver	Gastrointestinal	Pancreas	1.27	0.81	1.98	0.293	Chemotherapy & surgery
Gastritis and duodenitis	Gastrointestinal	Pancreas	0.90	0.65	1.25	0.54	Chemotherapy & surgery
Gastrooesophageal reflux disease	Gastrointestinal	Pancreas	0.99	0.67	1.46	0.975	Chemotherapy & surgery
Hepatic failure	Gastrointestinal	Pancreas	1.18	0.76	1.82	0.455	Chemotherapy & surgery
Oesophagitis and oesophageal ulcer	Gastrointestinal	Pancreas	0.84	0.60	1.18	0.316	Chemotherapy & surgery
Portal hypertension	Gastrointestinal	Pancreas	1.27	0.79	2.04	0.334	Chemotherapy & surgery
Other anaemias	Haematological	Pancreas	1.00	0.65	1.54	0.992	Chemotherapy & surgery
Chronic viral hepatitis	Immunology and infection	Pancreas	0.76	0.46	1.26	0.295	Chemotherapy & surgery
Infection of skin and subcutaneous tissues	Immunology and infection	Pancreas	0.64	0.34	1.21	0.17	Chemotherapy & surgery
Infections of other or unspecified organs	Immunology and infection	Pancreas	0.99	0.66	1.47	0.955	Chemotherapy & surgery
Infections of the digestive system	Immunology and infection	Pancreas	1.19	0.77	1.84	0.431	Chemotherapy & surgery
Lower respiratory tract infections	Immunology and infection	Pancreas	0.74	0.50	1.09	0.124	Chemotherapy & surgery
Peritonitis	Immunology and infection	Pancreas	1.24	0.90	1.70	0.188	Chemotherapy & surgery
Enthesopathies and synovial disorders	Musculoskeletal	Pancreas	0.93	0.63	1.37	0.709	Chemotherapy & surgery

Osteoarthritis (excluding spine)	Musculoskeletal	Pancreas	1.04	0.72	1.49	0.849	Chemotherapy & surgery
Osteoporosis	Musculoskeletal	Pancreas	0.87	0.53	1.45	0.601	Chemotherapy & surgery
Rheumatoid arthritis	Musculoskeletal	Pancreas	2.17	0.95	4.99	0.067	Chemotherapy & surgery
Peripheral neuropathies	Neurological	Pancreas	1.01	0.54	1.87	0.986	Chemotherapy & surgery
Asthma	Pulmonary	Pancreas	1.29	0.74	2.22	0.368	Chemotherapy & surgery
COPD	Pulmonary	Pancreas	1.12	0.71	1.77	0.615	Chemotherapy & surgery
End stage renal disease	Renal	Pancreas	0.84	0.47	1.49	0.552	Chemotherapy & surgery
Urolithiasis	Renal	Pancreas	0.81	0.40	1.60	0.538	Chemotherapy & surgery
Atrial fibrillation	Cardiovascular	Pancreas	0.57	0.38	0.84	0.005	Chemotherapy alone
Stroke (not otherwise specified)	Cardiovascular	Pancreas	0.47	0.24	0.91	0.024	Chemotherapy alone
Venous thromboembolic disease	Cardiovascular	Pancreas	0.66	0.45	0.97	0.034	Chemotherapy alone
Abdominal aortic aneurysm	Cardiovascular	Pancreas	1.47	0.87	2.48	0.153	Chemotherapy alone
Coronary heart disease not otherwise specified	Cardiovascular	Pancreas	0.95	0.72	1.25	0.728	Chemotherapy alone
Heart failure	Cardiovascular	Pancreas	0.66	0.43	1.02	0.064	Chemotherapy alone
Hypertension	Cardiovascular	Pancreas	1.19	0.96	1.46	0.107	Chemotherapy alone
Left bundle branch block	Cardiovascular	Pancreas	0.98	0.45	2.11	0.952	Chemotherapy alone
Myocardial infarction	Cardiovascular	Pancreas	0.80	0.57	1.13	0.204	Chemotherapy alone
Other cardiomyopathy	Cardiovascular	Pancreas	0.87	0.63	1.20	0.404	Chemotherapy alone
Peripheral arterial disease	Cardiovascular	Pancreas	0.83	0.48	1.41	0.484	Chemotherapy alone
Raynaud's syndrome	Cardiovascular	Pancreas	0.98	0.53	1.79	0.945	Chemotherapy alone
Stable angina	Cardiovascular	Pancreas	0.91	0.62	1.35	0.655	Chemotherapy alone
Transient ischaemic attack	Cardiovascular	Pancreas	0.84	0.53	1.33	0.45	Chemotherapy alone
Unstable angina	Cardiovascular	Pancreas	1.21	0.83	1.77	0.316	Chemotherapy alone
Diabetes	Endocrine	Pancreas	1.17	0.96	1.44	0.121	Chemotherapy alone
Diabetic neurological complications	Endocrine	Pancreas	0.96	0.77	1.20	0.722	Chemotherapy alone
Diabetic ophthalmic complications	Endocrine	Pancreas	0.98	0.79	1.22	0.854	Chemotherapy alone
Hypo or hyperthyroidism	Endocrine	Pancreas	0.83	0.51	1.36	0.468	Chemotherapy alone
Cholangitis	Gastrointestinal	Pancreas	0.82	0.69	0.97	0.024	Chemotherapy alone
Cholecystitis	Gastrointestinal	Pancreas	0.77	0.59	1.00	0.048	Chemotherapy alone
Diverticular disease of intestine	Gastrointestinal	Pancreas	1.92	1.44	2.56	< 0.001	Chemotherapy alone
Fatty liver	Gastrointestinal	Pancreas	1.55	1.10	2.19	0.013	Chemotherapy alone
Gastritis and duodenitis	Gastrointestinal	Pancreas	1.33	1.05	1.68	0.018	Chemotherapy alone
Gastrooesophageal reflux disease	Gastrointestinal	Pancreas	1.43	1.08	1.90	0.012	Chemotherapy alone
Portal hypertension	Gastrointestinal	Pancreas	1.53	1.05	2.21	0.025	Chemotherapy alone
Barrett's oesophagus	Gastrointestinal	Pancreas	0.82	0.53	1.26	0.371	Chemotherapy alone
Cholelithiasis	Gastrointestinal	Pancreas	0.83	0.64	1.09	0.184	Chemotherapy alone
Coeliac disease	Gastrointestinal	Pancreas	0.66	0.33	1.31	0.236	Chemotherapy alone
Hepatic failure	Gastrointestinal	Pancreas	1.34	0.96	1.89	0.087	Chemotherapy alone
Liver fibrosis sclerosis and cirrhosis	Gastrointestinal	Pancreas	0.82	0.32	2.10	0.684	Chemotherapy alone
Oesophagitis and oesophageal ulcer	Gastrointestinal	Pancreas	1.14	0.89	1.45	0.299	Chemotherapy alone
Other anaemias	Haematological	Pancreas	1.00	0.72	1.40	0.989	Chemotherapy alone
Chronic viral hepatitis	Immunology and infection	Pancreas	0.65	0.44	0.98	0.041	Chemotherapy alone
Infection of skin and subcutaneous tissues	Immunology and infection	Pancreas	0.57	0.35	0.93	0.025	Chemotherapy alone
Lower respiratory tract infections	Immunology and infection	Pancreas	0.75	0.56	1.00	0.049	Chemotherapy alone
Peritonitis	Immunology and infection	Pancreas	1.54	1.21	1.96	< 0.001	Chemotherapy alone
Infections of other or unspecified organs	Immunology and infection	Pancreas	0.81	0.58	1.12	0.2	Chemotherapy alone
Infections of the heart	Immunology and infection	Pancreas	0.64	0.30	1.35	0.24	Chemotherapy alone
Infections of the digestive system	Immunology and infection	Pancreas	0.99	0.69	1.41	0.947	Chemotherapy alone
Meningitis	Immunology and infection	Pancreas	1.05	0.52	2.11	0.889	Chemotherapy alone
Viral diseases (excluding chronic hepatitis/HIV)	Immunology and infection	Pancreas	1.16	0.49	2.78	0.734	Chemotherapy alone
Enthesopathies and synovial disorders	Musculoskeletal	Pancreas	0.72	0.52	0.99	0.043	Chemotherapy alone
Osteoporosis	Musculoskeletal	Pancreas	0.54	0.35	0.83	0.005	Chemotherapy alone
Osteoarthritis (excluding spine)	Musculoskeletal	Pancreas	1.19	0.91	1.56	0.197	Chemotherapy alone
Spinal stenosis	Musculoskeletal	Pancreas	0.87	0.37	2.04	0.743	Chemotherapy alone
Peripheral neuropathies	Neurological	Pancreas	0.51	0.29	0.88	0.016	Chemotherapy alone
Asthma	Pulmonary	Pancreas	0.98	0.62	1.55	0.921	Chemotherapy alone
COPD	Pulmonary	Pancreas	1.06	0.75	1.51	0.74	Chemotherapy alone
End stage renal disease	Renal	Pancreas	0.73	0.47	1.13	0.157	Chemotherapy alone
Obstructive and reflux uropathy	Renal	Pancreas	1.08	0.50	2.32	0.845	Chemotherapy alone
Urolithiasis	Renal	Pancreas	1.14	0.71	1.85	0.589	Chemotherapy alone
Hypertension	Cardiovascular	Pancreas	1.63	0.92	2.90	0.096	Chemotherapy, radiotherapy & surgery
Diabetes	Endocrine	Pancreas	1.04	0.54	2.00	0.899	Chemotherapy, radiotherapy & surgery
Cholangitis	Gastrointestinal	Pancreas	1.03	0.63	1.70	0.901	Chemotherapy, radiotherapy & surgery
Gastritis and duodenitis	Gastrointestinal	Pancreas	1.51	0.79	2.90	0.21	Chemotherapy, radiotherapy & surgery
Hypertension	Cardiovascular	Pancreas	1.19	0.61	2.33	0.606	Radiotherapy & surgery
Cholangitis	Gastrointestinal	Pancreas	1.16	0.68	1.99	0.584	Radiotherapy & surgery
Unstable angina	Cardiovascular	Pancreas	2.14	1.11	4.12	0.023	Radiotherapy alone
Coronary heart disease not otherwise specified	Cardiovascular	Pancreas	1.15	0.67	1.97	0.613	Radiotherapy alone
Hypertension	Cardiovascular	Pancreas	1.29	0.85	1.96	0.224	Radiotherapy alone
Myocardial infarction	Cardiovascular	Pancreas	1.14	0.59	2.22	0.698	Radiotherapy alone
Diabetes	Endocrine	Pancreas	1.24	0.82	1.87	0.316	Radiotherapy alone
Diabetic neurological complications	Endocrine	Pancreas	0.71	0.42	1.18	0.183	Radiotherapy alone
Diabetic ophthalmic complications	Endocrine	Pancreas	0.74	0.45	1.21	0.23	Radiotherapy alone
Cholangitis	Gastrointestinal	Pancreas	0.60	0.40	0.89	0.011	Radiotherapy alone
Cholecystitis	Gastrointestinal	Pancreas	0.74	0.41	1.33	0.319	Radiotherapy alone
Cholelithiasis	Gastrointestinal	Pancreas	0.89	0.50	1.58	0.695	Radiotherapy alone
Diverticular disease of intestine	Gastrointestinal	Pancreas	1.50	0.83	2.73	0.182	Radiotherapy alone
Gastritis and duodenitis	Gastrointestinal	Pancreas	1.09	0.66	1.80	0.735	Radiotherapy alone
Gastrooesophageal reflux disease	Gastrointestinal	Pancreas	1.02	0.55	1.92	0.943	Radiotherapy alone

Oesophagitis and oesophageal ulcer	Gastrointestinal	Pancreas	1.07	0.64	1.79	0.801	Radiotherapy alone
Peritonitis	Immunology and infection	Pancreas	1.32	0.79	2.19	0.293	Radiotherapy alone
Osteoarthritis (excluding spine)	Musculoskeletal	Pancreas	1.92	1.20	3.07	0.007	Radiotherapy alone
Enthesopathies and synovial disorders	Musculoskeletal	Pancreas	1.33	0.76	2.32	0.324	Radiotherapy alone
Heart failure	Cardiovascular	Prostate	0.74	0.57	0.95	0.02	Chemotherapy & radiotherapy
Peripheral arterial disease	Cardiovascular	Prostate	0.54	0.37	0.80	0.002	Chemotherapy & radiotherapy
Abdominal aortic aneurysm	Cardiovascular	Prostate	0.82	0.63	1.06	0.122	Chemotherapy & radiotherapy
Atrial fibrillation	Cardiovascular	Prostate	0.89	0.72	1.09	0.259	Chemotherapy & radiotherapy
Atrioventricular block, complete	Cardiovascular	Prostate	0.88	0.56	1.38	0.579	Chemotherapy & radiotherapy
Atrioventricular block, first degree	Cardiovascular	Prostate	0.88	0.56	1.38	0.579	Chemotherapy & radiotherapy
Atrioventricular block, second degree	Cardiovascular	Prostate	0.88	0.56	1.38	0.579	Chemotherapy & radiotherapy
Bifascicular block	Cardiovascular	Prostate	0.87	0.51	1.48	0.604	Chemotherapy & radiotherapy
Coronary heart disease not otherwise specified	Cardiovascular	Prostate	1.13	0.97	1.31	0.118	Chemotherapy & radiotherapy
Hypertension	Cardiovascular	Prostate	1.02	0.91	1.14	0.762	Chemotherapy & radiotherapy
Ischaemic stroke	Cardiovascular	Prostate	1.04	0.67	1.61	0.856	Chemotherapy & radiotherapy
Left bundle branch block	Cardiovascular	Prostate	0.89	0.58	1.36	0.589	Chemotherapy & radiotherapy
Myocardial infarction	Cardiovascular	Prostate	1.07	0.89	1.30	0.457	Chemotherapy & radiotherapy
Nonrheumatic aortic valve disorders	Cardiovascular	Prostate	1.00	0.64	1.58	0.986	Chemotherapy & radiotherapy
Nonrheumatic mitral valve disorders	Cardiovascular	Prostate	1.17	0.67	2.03	0.586	Chemotherapy & radiotherapy
Other cardiomyopathy	Cardiovascular	Prostate	1.10	0.92	1.31	0.28	Chemotherapy & radiotherapy
Raynaud's syndrome	Cardiovascular	Prostate	0.90	0.59	1.37	0.618	Chemotherapy & radiotherapy
Right bundle branch block	Cardiovascular	Prostate	0.89	0.52	1.52	0.665	Chemotherapy & radiotherapy
Stable angina	Cardiovascular	Prostate	1.04	0.84	1.28	0.716	Chemotherapy & radiotherapy
Stroke (not otherwise specified)	Cardiovascular	Prostate	0.98	0.70	1.37	0.905	Chemotherapy & radiotherapy
Supraventricular tachycardia	Cardiovascular	Prostate	0.83	0.44	1.58	0.576	Chemotherapy & radiotherapy
Transient ischaemic attack	Cardiovascular	Prostate	1.03	0.79	1.34	0.832	Chemotherapy & radiotherapy
Trifascicular block	Cardiovascular	Prostate	0.74	0.40	1.39	0.352	Chemotherapy & radiotherapy
Unstable angina	Cardiovascular	Prostate	1.05	0.85	1.29	0.676	Chemotherapy & radiotherapy
Venous thromboembolic disease	Cardiovascular	Prostate	0.98	0.72	1.32	0.882	Chemotherapy & radiotherapy
Ventricular tachycardia	Cardiovascular	Prostate	0.80	0.42	1.51	0.494	Chemotherapy & radiotherapy
Hypo or hyperthyroidism	Endocrine	Prostate	1.57	1.11	2.22	0.011	Chemotherapy & radiotherapy
Diabetes	Endocrine	Prostate	1.13	0.96	1.32	0.14	Chemotherapy & radiotherapy
Diabetic neurological complications	Endocrine	Prostate	0.83	0.68	1.03	0.088	Chemotherapy & radiotherapy
Diabetic ophthalmic complications	Endocrine	Prostate	0.87	0.72	1.06	0.161	Chemotherapy & radiotherapy
Cholangitis	Gastrointestinal	Prostate	0.61	0.40	0.94	0.026	Chemotherapy & radiotherapy
Cholecystitis	Gastrointestinal	Prostate	0.61	0.40	0.92	0.019	Chemotherapy & radiotherapy
Cholelithiasis	Gastrointestinal	Prostate	0.62	0.42	0.90	0.012	Chemotherapy & radiotherapy
Barrett's oesophagus	Gastrointestinal	Prostate	1.07	0.77	1.48	0.699	Chemotherapy & radiotherapy
Diverticular disease of intestine	Gastrointestinal	Prostate	0.90	0.72	1.12	0.328	Chemotherapy & radiotherapy
Fatty liver	Gastrointestinal	Prostate	0.71	0.41	1.25	0.239	Chemotherapy & radiotherapy
Gastritis and duodenitis	Gastrointestinal	Prostate	1.02	0.81	1.28	0.873	Chemotherapy & radiotherapy
Gastrooesophageal reflux disease	Gastrointestinal	Prostate	1.02	0.82	1.27	0.837	Chemotherapy & radiotherapy
Hepatic failure	Gastrointestinal	Prostate	0.73	0.38	1.40	0.346	Chemotherapy & radiotherapy
Oesophagitis and oesophageal ulcer	Gastrointestinal	Prostate	0.97	0.79	1.19	0.77	Chemotherapy & radiotherapy
Other anaemias	Haematological	Prostate	0.66	0.50	0.86	0.002	Chemotherapy & radiotherapy
Infection of skin and subcutaneous tissues	Immunology and infection	Prostate	0.56	0.38	0.84	0.005	Chemotherapy & radiotherapy
Infections of other or unspecified organs	Immunology and infection	Prostate	0.63	0.48	0.81	< 0.001	Chemotherapy & radiotherapy
Lower respiratory tract infections	Immunology and infection	Prostate	0.77	0.62	0.96	0.018	Chemotherapy & radiotherapy
Non-acute cystitis	Immunology and infection	Prostate	0.46	0.26	0.83	0.009	Chemotherapy & radiotherapy
Infections of the digestive system	Immunology and infection	Prostate	0.91	0.68	1.22	0.519	Chemotherapy & radiotherapy
Peritonitis	Immunology and infection	Prostate	0.97	0.80	1.17	0.75	Chemotherapy & radiotherapy
Viral diseases (excluding chronic hepatitis/HIV)	Immunology and infection	Prostate	1.45	0.88	2.40	0.149	Chemotherapy & radiotherapy
Enthesopathies and synovial disorders	Musculoskeletal	Prostate	1.13	0.96	1.34	0.144	Chemotherapy & radiotherapy
Osteoarthritis (excluding spine)	Musculoskeletal	Prostate	1.10	0.95	1.28	0.196	Chemotherapy & radiotherapy
Osteoporosis	Musculoskeletal	Prostate	0.93	0.62	1.39	0.734	Chemotherapy & radiotherapy
Spinal stenosis	Musculoskeletal	Prostate	1.30	0.86	1.97	0.218	Chemotherapy & radiotherapy
Disorders of autonomic nervous system	Neurological	Prostate	2.09	1.21	3.62	0.009	Chemotherapy & radiotherapy
Dementia	Neurological	Prostate	0.60	0.34	1.06	0.078	Chemotherapy & radiotherapy
Peripheral neuropathies	Neurological	Prostate	1.16	0.87	1.55	0.318	Chemotherapy & radiotherapy
Asthma	Pulmonary	Prostate	1.04	0.80	1.37	0.753	Chemotherapy & radiotherapy
COPD	Pulmonary	Prostate	0.80	0.64	1.01	0.06	Chemotherapy & radiotherapy
End stage renal disease	Renal	Prostate	0.47	0.34	0.66	< 0.001	Chemotherapy & radiotherapy
Obstructive and reflux uropathy	Renal	Prostate	0.37	0.26	0.52	< 0.001	Chemotherapy & radiotherapy
Urolithiasis	Renal	Prostate	0.34	0.25	0.46	< 0.001	Chemotherapy & radiotherapy
Coronary heart disease not otherwise specified	Cardiovascular	Prostate	1.20	1.03	1.40	0.023	Chemotherapy & surgery
Ischaemic stroke	Cardiovascular	Prostate	1.67	1.13	2.44	0.009	Chemotherapy & surgery
Venous thromboembolic disease	Cardiovascular	Prostate	1.42	1.06	1.88	0.017	Chemotherapy & surgery
Abdominal aortic aneurysm	Cardiovascular	Prostate	1.26	0.95	1.67	0.114	Chemotherapy & surgery
Atrial fibrillation	Cardiovascular	Prostate	0.90	0.73	1.12	0.345	Chemotherapy & surgery
Atrioventricular block, complete	Cardiovascular	Prostate	0.68	0.41	1.12	0.129	Chemotherapy & surgery
Atrioventricular block, first degree	Cardiovascular	Prostate	0.68	0.41	1.12	0.129	Chemotherapy & surgery
Atrioventricular block, second degree	Cardiovascular	Prostate	0.68	0.41	1.12	0.129	Chemotherapy & surgery
Bifascicular block	Cardiovascular	Prostate	1.18	0.72	1.96	0.511	Chemotherapy & surgery
Heart failure	Cardiovascular	Prostate	0.98	0.77	1.25	0.868	Chemotherapy & surgery
Hypertension	Cardiovascular	Prostate	1.04	0.92	1.18	0.532	Chemotherapy & surgery
Left bundle branch block	Cardiovascular	Prostate	0.72	0.45	1.16	0.174	Chemotherapy & surgery
Myocardial infarction	Cardiovascular	Prostate	1.08	0.88	1.31	0.47	Chemotherapy & surgery
Nonrheumatic aortic valve disorders	Cardiovascular	Prostate	0.92	0.57	1.48	0.728	Chemotherapy & surgery
Other cardiomyopathy	Cardiovascular	Prostate	1.10	0.92	1.33	0.303	Chemotherapy & surgery
Peripheral arterial disease	Cardiovascular	Prostate	0.91	0.66	1.25	0.571	Chemotherapy & surgery
Raynaud's syndrome	Cardiovascular	Prostate	0.99	0.65	1.51	0.967	Chemotherapy & surgery

Right bundle branch block	Cardiovascular	Prostate	1.20	0.72	1.99	0.479	Chemotherapy & surgery
Sick sinus syndrome	Cardiovascular	Prostate	1.69	0.97	2.96	0.064	Chemotherapy & surgery
Stable angina	Cardiovascular	Prostate	1.21	0.98	1.49	0.07	Chemotherapy & surgery
Stroke (not otherwise specified)	Cardiovascular	Prostate	1.14	0.83	1.58	0.412	Chemotherapy & surgery
Transient ischaemic attack	Cardiovascular	Prostate	0.93	0.70	1.25	0.652	Chemotherapy & surgery
Trifascicular block	Cardiovascular	Prostate	1.06	0.60	1.86	0.84	Chemotherapy & surgery
Unstable angina	Cardiovascular	Prostate	1.08	0.87	1.35	0.465	Chemotherapy & surgery
Diabetes	Endocrine	Prostate	0.91	0.75	1.10	0.329	Chemotherapy & surgery
Diabetic neurological complications	Endocrine	Prostate	1.13	0.92	1.40	0.243	Chemotherapy & surgery
Diabetic ophthalmic complications	Endocrine	Prostate	1.02	0.84	1.25	0.809	Chemotherapy & surgery
Hypo or hyperthyroidism	Endocrine	Prostate	0.98	0.64	1.50	0.927	Chemotherapy & surgery
Cholangitis	Gastrointestinal	Prostate	0.62	0.39	0.97	0.037	Chemotherapy & surgery
Cholelithiasis	Gastrointestinal	Prostate	0.67	0.45	0.99	0.044	Chemotherapy & surgery
Barrett's oesophagus	Gastrointestinal	Prostate	0.90	0.61	1.32	0.59	Chemotherapy & surgery
Cholecystitis	Gastrointestinal	Prostate	0.72	0.48	1.10	0.131	Chemotherapy & surgery
Diverticular disease of intestine	Gastrointestinal	Prostate	0.85	0.67	1.08	0.191	Chemotherapy & surgery
Fatty liver	Gastrointestinal	Prostate	1.08	0.56	2.06	0.819	Chemotherapy & surgery
Gastritis and duodenitis	Gastrointestinal	Prostate	1.20	0.95	1.51	0.129	Chemotherapy & surgery
Gastrooesophageal reflux disease	Gastrointestinal	Prostate	0.82	0.63	1.08	0.153	Chemotherapy & surgery
Hepatic failure	Gastrointestinal	Prostate	1.37	0.71	2.65	0.352	Chemotherapy & surgery
Oesophagitis and oesophageal ulcer	Gastrointestinal	Prostate	0.96	0.76	1.20	0.696	Chemotherapy & surgery
Other anaemias	Haematological	Prostate	1.04	0.82	1.33	0.721	Chemotherapy & surgery
Infections of other or unspecified organs	Immunology and infection	Prostate	0.75	0.58	0.98	0.034	Chemotherapy & surgery
Infection of skin and subcutaneous tissues	Immunology and infection	Prostate	1.06	0.75	1.51	0.734	Chemotherapy & surgery
Infections of the digestive system	Immunology and infection	Prostate	0.94	0.66	1.34	0.743	Chemotherapy & surgery
Lower respiratory tract infections	Immunology and infection	Prostate	0.90	0.73	1.12	0.365	Chemotherapy & surgery
Non-acute cystitis	Immunology and infection	Prostate	1.23	0.82	1.84	0.313	Chemotherapy & surgery
Peritonitis	Immunology and infection	Prostate	0.80	0.64	1.00	0.051	Chemotherapy & surgery
Enthesopathies and synovial disorders	Musculoskeletal	Prostate	1.10	0.90	1.34	0.345	Chemotherapy & surgery
Osteoarthritis (excluding spine)	Musculoskeletal	Prostate	0.91	0.77	1.08	0.29	Chemotherapy & surgery
Osteoporosis	Musculoskeletal	Prostate	0.77	0.50	1.17	0.222	Chemotherapy & surgery
Rheumatoid arthritis	Musculoskeletal	Prostate	1.24	0.65	2.40	0.514	Chemotherapy & surgery
Spinal stenosis	Musculoskeletal	Prostate	1.04	0.61	1.75	0.894	Chemotherapy & surgery
Intracerebral haemorrhage	Neurological	Prostate	1.99	1.17	3.40	0.011	Chemotherapy & surgery
Subarachnoid haemorrhage	Neurological	Prostate	2.03	1.15	3.59	0.015	Chemotherapy & surgery
Dementia	Neurological	Prostate	0.86	0.53	1.42	0.561	Chemotherapy & surgery
Peripheral neuropathies	Neurological	Prostate	1.11	0.79	1.56	0.539	Chemotherapy & surgery
Asthma	Pulmonary	Prostate	1.25	0.96	1.64	0.101	Chemotherapy & surgery
COPD	Pulmonary	Prostate	1.03	0.82	1.28	0.827	Chemotherapy & surgery
Urolithiasis	Renal	Prostate	0.73	0.58	0.93	0.01	Chemotherapy & surgery
End stage renal disease	Renal	Prostate	0.88	0.66	1.17	0.371	Chemotherapy & surgery
Glomerulonephritis	Renal	Prostate	0.99	0.57	1.73	0.978	Chemotherapy & surgery
Obstructive and reflux uropathy	Renal	Prostate	0.92	0.70	1.21	0.541	Chemotherapy & surgery
Hypertension	Cardiovascular	Prostate	0.84	0.75	0.95	0.004	Chemotherapy alone
Abdominal aortic aneurysm	Cardiovascular	Prostate	1.31	1.06	1.64	0.015	Chemotherapy alone
Ischaemic stroke	Cardiovascular	Prostate	1.71	1.24	2.36	0.001	Chemotherapy alone
Stroke (not otherwise specified)	Cardiovascular	Prostate	1.42	1.10	1.83	0.007	Chemotherapy alone
Venous thromboembolic disease	Cardiovascular	Prostate	1.29	1.02	1.64	0.034	Chemotherapy alone
Atrial fibrillation	Cardiovascular	Prostate	1.00	0.84	1.19	0.994	Chemotherapy alone
Atrioventricular block, complete	Cardiovascular	Prostate	1.10	0.79	1.54	0.569	Chemotherapy alone
Atrioventricular block, first degree	Cardiovascular	Prostate	1.10	0.79	1.54	0.569	Chemotherapy alone
Atrioventricular block, second degree	Cardiovascular	Prostate	1.10	0.79	1.54	0.569	Chemotherapy alone
Bifascicular block	Cardiovascular	Prostate	1.09	0.72	1.63	0.693	Chemotherapy alone
Coronary heart disease not otherwise specified	Cardiovascular	Prostate	1.00	0.86	1.15	0.966	Chemotherapy alone
Heart failure	Cardiovascular	Prostate	1.08	0.89	1.31	0.42	Chemotherapy alone
Left bundle branch block	Cardiovascular	Prostate	1.06	0.76	1.48	0.719	Chemotherapy alone
Multiple valve disease	Cardiovascular	Prostate	1.38	0.86	2.23	0.182	Chemotherapy alone
Myocardial infarction	Cardiovascular	Prostate	0.99	0.84	1.18	0.954	Chemotherapy alone
Nonrheumatic aortic valve disorders	Cardiovascular	Prostate	0.92	0.63	1.36	0.681	Chemotherapy alone
Nonrheumatic mitral valve disorders	Cardiovascular	Prostate	1.03	0.63	1.70	0.894	Chemotherapy alone
Other cardiomyopathy	Cardiovascular	Prostate	0.96	0.82	1.13	0.663	Chemotherapy alone
Peripheral arterial disease	Cardiovascular	Prostate	0.94	0.71	1.23	0.636	Chemotherapy alone
Raynaud's syndrome	Cardiovascular	Prostate	0.97	0.67	1.39	0.852	Chemotherapy alone
Right bundle branch block	Cardiovascular	Prostate	1.05	0.69	1.59	0.812	Chemotherapy alone
Sick sinus syndrome	Cardiovascular	Prostate	0.74	0.41	1.35	0.332	Chemotherapy alone
Stable angina	Cardiovascular	Prostate	0.97	0.80	1.18	0.748	Chemotherapy alone
Supraventricular tachycardia	Cardiovascular	Prostate	0.59	0.32	1.10	0.097	Chemotherapy alone
Transient ischaemic attack	Cardiovascular	Prostate	1.13	0.91	1.41	0.274	Chemotherapy alone
Trifascicular block	Cardiovascular	Prostate	0.99	0.63	1.57	0.966	Chemotherapy alone
Unstable angina	Cardiovascular	Prostate	0.95	0.78	1.15	0.581	Chemotherapy alone
Ventricular tachycardia	Cardiovascular	Prostate	0.56	0.30	1.04	0.069	Chemotherapy alone
Diabetes	Endocrine	Prostate	0.97	0.83	1.14	0.726	Chemotherapy alone
Diabetic neurological complications	Endocrine	Prostate	0.88	0.73	1.05	0.162	Chemotherapy alone
Diabetic ophthalmic complications	Endocrine	Prostate	0.84	0.71	1.00	0.052	Chemotherapy alone
Hypo or hyperthyroidism	Endocrine	Prostate	1.04	0.73	1.49	0.816	Chemotherapy alone
Cholangitis	Gastrointestinal	Prostate	0.69	0.49	0.98	0.041	Chemotherapy alone
Cholelithiasis	Gastrointestinal	Prostate	0.61	0.44	0.85	0.003	Chemotherapy alone
Alcoholic liver disease	Gastrointestinal	Prostate	2.05	1.02	4.10	0.043	Chemotherapy alone
Barrett's oesophagus	Gastrointestinal	Prostate	0.97	0.71	1.33	0.856	Chemotherapy alone
Cholecystitis	Gastrointestinal	Prostate	0.83	0.60	1.15	0.263	Chemotherapy alone
Coeliac disease	Gastrointestinal	Prostate	1.05	0.47	2.32	0.913	Chemotherapy alone

Diverticular disease of intestine	Gastrointestinal	Prostate	1.01	0.83	1.23	0.93	Chemotherapy alone
Fatty liver	Gastrointestinal	Prostate	1.48	0.95	2.32	0.083	Chemotherapy alone
Gastritis and duodenitis	Gastrointestinal	Prostate	1.11	0.90	1.37	0.328	Chemotherapy alone
Gastrooesophageal reflux disease	Gastrointestinal	Prostate	0.85	0.68	1.06	0.149	Chemotherapy alone
Hepatic failure	Gastrointestinal	Prostate	1.06	0.61	1.85	0.824	Chemotherapy alone
Oesophagitis and oesophageal ulcer	Gastrointestinal	Prostate	1.00	0.83	1.21	0.99	Chemotherapy alone
Portal hypertension	Gastrointestinal	Prostate	1.08	0.57	2.05	0.803	Chemotherapy alone
Secondary polycythaemia	Haematological	Prostate	3.90	1.65	9.24	0.002	Chemotherapy alone
Other anaemias	Haematological	Prostate	1.04	0.86	1.27	0.688	Chemotherapy alone
Primary or idiopathic thrombocytopaenia	Haematological	Prostate	1.07	0.50	2.29	0.858	Chemotherapy alone
Secondary or other thrombocytopaenia	Haematological	Prostate	1.29	0.71	2.32	0.402	Chemotherapy alone
Infection of bones and joints	Immunology and infection	Prostate	0.50	0.27	0.92	0.027	Chemotherapy alone
Infections of other or unspecified organs	Immunology and infection	Prostate	0.69	0.55	0.86	0.001	Chemotherapy alone
Non-acute cystitis	Immunology and infection	Prostate	0.30	0.16	0.54	< 0.001	Chemotherapy alone
Chronic viral hepatitis	Immunology and infection	Prostate	1.24	0.66	2.36	0.505	Chemotherapy alone
Infection of skin and subcutaneous tissues	Immunology and infection	Prostate	1.01	0.76	1.35	0.924	Chemotherapy alone
Infections of the heart	Immunology and infection	Prostate	0.99	0.51	1.95	0.986	Chemotherapy alone
Infections of the digestive system	Immunology and infection	Prostate	0.81	0.61	1.08	0.149	Chemotherapy alone
Lower respiratory tract infections	Immunology and infection	Prostate	1.08	0.91	1.27	0.4	Chemotherapy alone
Meningitis	Immunology and infection	Prostate	0.97	0.50	1.86	0.918	Chemotherapy alone
Peritonitis	Immunology and infection	Prostate	0.84	0.70	1.01	0.071	Chemotherapy alone
Viral diseases (excluding chronic hepatitis/HIV)	Immunology and infection	Prostate	1.38	0.85	2.23	0.195	Chemotherapy alone
Enthesopathies and synovial disorders	Musculoskeletal	Prostate	0.98	0.82	1.17	0.834	Chemotherapy alone
Osteoarthritis (excluding spine)	Musculoskeletal	Prostate	0.86	0.74	1.00	0.053	Chemotherapy alone
Osteoporosis	Musculoskeletal	Prostate	0.85	0.60	1.22	0.379	Chemotherapy alone
Rheumatoid arthritis	Musculoskeletal	Prostate	0.93	0.51	1.69	0.8	Chemotherapy alone
Spinal stenosis	Musculoskeletal	Prostate	0.86	0.55	1.33	0.487	Chemotherapy alone
Dementia	Neurological	Prostate	1.12	0.80	1.59	0.501	Chemotherapy alone
Disorders of autonomic nervous system	Neurological	Prostate	0.63	0.31	1.28	0.2	Chemotherapy alone
Epilepsy	Neurological	Prostate	1.20	0.69	2.09	0.521	Chemotherapy alone
Intracerebral haemorrhage	Neurological	Prostate	1.25	0.75	2.09	0.393	Chemotherapy alone
Peripheral neuropathies	Neurological	Prostate	0.84	0.61	1.14	0.253	Chemotherapy alone
Subarachnoid haemorrhage	Neurological	Prostate	1.15	0.65	2.01	0.634	Chemotherapy alone
Bronchiectasis	Pulmonary	Prostate	1.91	1.09	3.34	0.023	Chemotherapy alone
Asthma	Pulmonary	Prostate	1.03	0.80	1.33	0.793	Chemotherapy alone
COPD	Pulmonary	Prostate	1.09	0.90	1.31	0.377	Chemotherapy alone
Other interstitial pulmonary diseases with fibrosis	Pulmonary	Prostate	1.17	0.57	2.43	0.665	Chemotherapy alone
End stage renal disease	Renal	Prostate	0.78	0.61	0.98	0.036	Chemotherapy alone
Glomerulonephritis	Renal	Prostate	0.57	0.32	0.99	0.045	Chemotherapy alone
Obstructive and reflux uropathy	Renal	Prostate	0.45	0.34	0.59	< 0.001	Chemotherapy alone
Urolithiasis	Renal	Prostate	0.48	0.38	0.60	< 0.001	Chemotherapy alone
Abdominal aortic aneurysm	Cardiovascular	Prostate	0.47	0.32	0.70	< 0.001	Chemotherapy, radiotherapy & surgery
Heart failure	Cardiovascular	Prostate	0.57	0.40	0.79	0.001	Chemotherapy, radiotherapy & surgery
Peripheral arterial disease	Cardiovascular	Prostate	0.58	0.38	0.87	0.009	Chemotherapy, radiotherapy & surgery
Atrial fibrillation	Cardiovascular	Prostate	0.79	0.62	1.01	0.061	Chemotherapy, radiotherapy & surgery
Atrioventricular block, complete	Cardiovascular	Prostate	0.98	0.59	1.62	0.94	Chemotherapy, radiotherapy & surgery
Atrioventricular block, first degree	Cardiovascular	Prostate	0.98	0.59	1.62	0.94	Chemotherapy, radiotherapy & surgery
Atrioventricular block, second degree	Cardiovascular	Prostate	0.98	0.59	1.62	0.94	Chemotherapy, radiotherapy & surgery
Bifascicular block	Cardiovascular	Prostate	1.55	0.93	2.57	0.094	Chemotherapy, radiotherapy & surgery
Coronary heart disease not otherwise specified	Cardiovascular	Prostate	0.94	0.79	1.11	0.444	Chemotherapy, radiotherapy & surgery
Hypertension	Cardiovascular	Prostate	1.06	0.94	1.20	0.317	Chemotherapy, radiotherapy & surgery
Ischaemic stroke	Cardiovascular	Prostate	0.94	0.57	1.56	0.819	Chemotherapy, radiotherapy & surgery
Left bundle branch block	Cardiovascular	Prostate	1.10	0.70	1.74	0.684	Chemotherapy, radiotherapy & surgery
Myocardial infarction	Cardiovascular	Prostate	1.00	0.81	1.23	0.969	Chemotherapy, radiotherapy & surgery
Nonrheumatic aortic valve disorders	Cardiovascular	Prostate	0.90	0.53	1.55	0.707	Chemotherapy, radiotherapy & surgery
Nonrheumatic mitral valve disorders	Cardiovascular	Prostate	1.08	0.58	2.02	0.802	Chemotherapy, radiotherapy & surgery
Other cardiomyopathy	Cardiovascular	Prostate	0.95	0.77	1.16	0.589	Chemotherapy, radiotherapy & surgery
Raynaud's syndrome	Cardiovascular	Prostate	0.83	0.51	1.35	0.46	Chemotherapy, radiotherapy & surgery
Right bundle branch block	Cardiovascular	Prostate	1.57	0.94	2.61	0.085	Chemotherapy, radiotherapy & surgery
Stable angina	Cardiovascular	Prostate	0.88	0.69	1.12	0.291	Chemotherapy, radiotherapy & surgery
Stroke (not otherwise specified)	Cardiovascular	Prostate	0.99	0.68	1.45	0.976	Chemotherapy, radiotherapy & surgery
Transient ischaemic attack	Cardiovascular	Prostate	0.87	0.63	1.20	0.404	Chemotherapy, radiotherapy & surgery
Trifascicular block	Cardiovascular	Prostate	1.34	0.74	2.39	0.332	Chemotherapy, radiotherapy & surgery
Unstable angina	Cardiovascular	Prostate	0.94	0.74	1.20	0.642	Chemotherapy, radiotherapy & surgery
Venous thromboembolic disease	Cardiovascular	Prostate	1.05	0.75	1.47	0.798	Chemotherapy, radiotherapy & surgery
Ventricular tachycardia	Cardiovascular	Prostate	1.30	0.70	2.43	0.405	Chemotherapy, radiotherapy & surgery
Diabetes	Endocrine	Prostate	0.96	0.80	1.15	0.645	Chemotherapy, radiotherapy & surgery
Diabetic neurological complications	Endocrine	Prostate	1.06	0.86	1.32	0.575	Chemotherapy, radiotherapy & surgery
Diabetic ophthalmic complications	Endocrine	Prostate	1.12	0.92	1.37	0.256	Chemotherapy, radiotherapy & surgery
Hypo or hyperthyroidism	Endocrine	Prostate	1.45	0.99	2.14	0.058	Chemotherapy, radiotherapy & surgery
Barrett's oesophagus	Gastrointestinal	Prostate	0.94	0.64	1.37	0.736	Chemotherapy, radiotherapy & surgery
Cholangitis	Gastrointestinal	Prostate	0.85	0.56	1.30	0.461	Chemotherapy, radiotherapy & surgery
Cholecystitis	Gastrointestinal	Prostate	0.91	0.61	1.36	0.651	Chemotherapy, radiotherapy & surgery
Cholelithiasis	Gastrointestinal	Prostate	0.98	0.68	1.41	0.923	Chemotherapy, radiotherapy & surgery
Diverticular disease of intestine	Gastrointestinal	Prostate	0.83	0.65	1.07	0.144	Chemotherapy, radiotherapy & surgery
Fatty liver	Gastrointestinal	Prostate	0.81	0.42	1.56	0.53	Chemotherapy, radiotherapy & surgery
Gastritis and duodenitis	Gastrointestinal	Prostate	0.88	0.67	1.14	0.321	Chemotherapy, radiotherapy & surgery
Gastrooesophageal reflux disease	Gastrointestinal	Prostate	1.00	0.79	1.27	0.993	Chemotherapy, radiotherapy & surgery
Hepatic failure	Gastrointestinal	Prostate	1.25	0.66	2.36	0.503	Chemotherapy, radiotherapy & surgery

Oesophagitis and oesophageal ulcer	Gastrointestinal	Prostate	0.99	0.80	1.24	0.953	Chemotherapy, radiotherapy & surgery
Other anaemias	Haematological	Prostate	0.76	0.57	1.02	0.072	Chemotherapy, radiotherapy & surgery
Infections of other or unspecified organs	Immunology and infection	Prostate	0.71	0.54	0.94	0.017	Chemotherapy, radiotherapy & surgery
Infection of skin and subcutaneous tissues	Immunology and infection	Prostate	0.71	0.46	1.10	0.126	Chemotherapy, radiotherapy & surgery
Infections of the digestive system	Immunology and infection	Prostate	0.91	0.64	1.29	0.587	Chemotherapy, radiotherapy & surgery
Lower respiratory tract infections	Immunology and infection	Prostate	0.87	0.68	1.10	0.242	Chemotherapy, radiotherapy & surgery
Peritonitis	Immunology and infection	Prostate	0.97	0.78	1.21	0.797	Chemotherapy, radiotherapy & surgery
Viral diseases (excluding chronic hepatitis/HIV)	Immunology and infection	Prostate	0.84	0.43	1.67	0.625	Chemotherapy, radiotherapy & surgery
Enthesopathies and synovial disorders	Musculoskeletal	Prostate	1.19	0.99	1.42	0.061	Chemotherapy, radiotherapy & surgery
Osteoarthritis (excluding spine)	Musculoskeletal	Prostate	1.06	0.90	1.25	0.491	Chemotherapy, radiotherapy & surgery
Osteoporosis	Musculoskeletal	Prostate	0.64	0.39	1.05	0.078	Chemotherapy, radiotherapy & surgery
Spinal stenosis	Musculoskeletal	Prostate	1.00	0.58	1.72	0.99	Chemotherapy, radiotherapy & surgery
Epilepsy	Neurological	Prostate	1.53	0.82	2.85	0.177	Chemotherapy, radiotherapy & surgery
Peripheral neuropathies	Neurological	Prostate	1.14	0.82	1.58	0.451	Chemotherapy, radiotherapy & surgery
Asthma	Pulmonary	Prostate	0.86	0.63	1.17	0.333	Chemotherapy, radiotherapy & surgery
COPD	Pulmonary	Prostate	0.80	0.62	1.03	0.088	Chemotherapy, radiotherapy & surgery
End stage renal disease	Renal	Prostate	0.50	0.34	0.74	0.001	Chemotherapy, radiotherapy & surgery
Obstructive and reflux uropathy	Renal	Prostate	0.51	0.35	0.75	< 0.001	Chemotherapy, radiotherapy & surgery
Urolithiasis	Renal	Prostate	0.51	0.38	0.68	< 0.001	Chemotherapy, radiotherapy & surgery
Glomerulonephritis	Renal	Prostate	1.00	0.55	1.80	0.993	Chemotherapy, radiotherapy & surgery
Abdominal aortic aneurysm	Cardiovascular	Prostate	0.64	0.50	0.83	0.001	Radiotherapy & surgery
Heart failure	Cardiovascular	Prostate	0.70	0.55	0.89	0.004	Radiotherapy & surgery
Other cardiomyopathy	Cardiovascular	Prostate	0.81	0.68	0.95	0.012	Radiotherapy & surgery
Peripheral arterial disease	Cardiovascular	Prostate	0.72	0.53	0.97	0.03	Radiotherapy & surgery
Stable angina	Cardiovascular	Prostate	0.79	0.65	0.96	0.019	Radiotherapy & surgery
Unstable angina	Cardiovascular	Prostate	0.80	0.66	0.98	0.028	Radiotherapy & surgery
Atrial fibrillation	Cardiovascular	Prostate	0.88	0.73	1.06	0.183	Radiotherapy & surgery
Atrioventricular block, complete	Cardiovascular	Prostate	1.21	0.84	1.74	0.315	Radiotherapy & surgery
Atrioventricular block, first degree	Cardiovascular	Prostate	1.21	0.84	1.74	0.315	Radiotherapy & surgery
Atrioventricular block, second degree	Cardiovascular	Prostate	1.21	0.84	1.74	0.315	Radiotherapy & surgery
Bifascicular block	Cardiovascular	Prostate	1.23	0.80	1.90	0.338	Radiotherapy & surgery
Coronary heart disease not otherwise specified	Cardiovascular	Prostate	1.02	0.90	1.16	0.765	Radiotherapy & surgery
Hypertension	Cardiovascular	Prostate	0.93	0.84	1.03	0.167	Radiotherapy & surgery
Ischaemic stroke	Cardiovascular	Prostate	0.86	0.57	1.30	0.475	Radiotherapy & surgery
Left bundle branch block	Cardiovascular	Prostate	1.12	0.79	1.60	0.526	Radiotherapy & surgery
Multiple valve disease	Cardiovascular	Prostate	1.18	0.68	2.04	0.549	Radiotherapy & surgery
Myocardial infarction	Cardiovascular	Prostate	0.88	0.74	1.05	0.151	Radiotherapy & surgery
Nonrheumatic aortic valve disorders	Cardiovascular	Prostate	0.86	0.55	1.34	0.504	Radiotherapy & surgery
Nonrheumatic mitral valve disorders	Cardiovascular	Prostate	1.08	0.66	1.78	0.76	Radiotherapy & surgery
Raynaud's syndrome	Cardiovascular	Prostate	0.97	0.67	1.39	0.856	Radiotherapy & surgery
Right bundle branch block	Cardiovascular	Prostate	1.25	0.81	1.92	0.316	Radiotherapy & surgery
Sick sinus syndrome	Cardiovascular	Prostate	0.90	0.49	1.65	0.741	Radiotherapy & surgery
Stroke (not otherwise specified)	Cardiovascular	Prostate	0.72	0.52	1.01	0.054	Radiotherapy & surgery
Supraventricular tachycardia	Cardiovascular	Prostate	1.40	0.88	2.24	0.158	Radiotherapy & surgery
Transient ischaemic attack	Cardiovascular	Prostate	0.83	0.64	1.07	0.159	Radiotherapy & surgery
Trifascicular block	Cardiovascular	Prostate	1.22	0.76	1.96	0.414	Radiotherapy & surgery
Venous thromboembolic disease	Cardiovascular	Prostate	0.83	0.62	1.10	0.197	Radiotherapy & surgery
Ventricular tachycardia	Cardiovascular	Prostate	1.27	0.79	2.04	0.319	Radiotherapy & surgery
Hypo or hyperthyroidism	Endocrine	Prostate	1.43	1.05	1.97	0.025	Radiotherapy & surgery
Diabetes	Endocrine	Prostate	0.99	0.86	1.15	0.923	Radiotherapy & surgery
Diabetic neurological complications	Endocrine	Prostate	1.02	0.86	1.22	0.781	Radiotherapy & surgery
Diabetic ophthalmic complications	Endocrine	Prostate	1.01	0.86	1.18	0.932	Radiotherapy & surgery
Barrett's oesophagus	Gastrointestinal	Prostate	0.64	0.45	0.90	0.01	Radiotherapy & surgery
Cholangitis	Gastrointestinal	Prostate	1.06	0.77	1.45	0.729	Radiotherapy & surgery
Cholecystitis	Gastrointestinal	Prostate	1.18	0.88	1.59	0.266	Radiotherapy & surgery
Cholelithiasis	Gastrointestinal	Prostate	0.98	0.74	1.31	0.893	Radiotherapy & surgery
Diverticular disease of intestine	Gastrointestinal	Prostate	0.90	0.74	1.08	0.255	Radiotherapy & surgery
Fatty liver	Gastrointestinal	Prostate	0.96	0.61	1.51	0.851	Radiotherapy & surgery
Gastritis and duodenitis	Gastrointestinal	Prostate	0.94	0.77	1.15	0.559	Radiotherapy & surgery
Gastrooesophageal reflux disease	Gastrointestinal	Prostate	0.86	0.70	1.04	0.125	Radiotherapy & surgery
Hepatic failure	Gastrointestinal	Prostate	0.89	0.51	1.53	0.671	Radiotherapy & surgery
Oesophagitis and oesophageal ulcer	Gastrointestinal	Prostate	0.87	0.73	1.04	0.124	Radiotherapy & surgery
Portal hypertension	Gastrointestinal	Prostate	1.15	0.61	2.15	0.669	Radiotherapy & surgery
Other anaemias	Haematological	Prostate	0.69	0.54	0.87	0.002	Radiotherapy & surgery
Secondary or other thrombocytopaenia	Haematological	Prostate	1.10	0.58	2.07	0.776	Radiotherapy & surgery
Infections of other or unspecified organs	Immunology and infection	Prostate	0.79	0.64	0.97	0.024	Radiotherapy & surgery
Lower respiratory tract infections	Immunology and infection	Prostate	0.74	0.61	0.90	0.003	Radiotherapy & surgery
Non-acute cystitis	Immunology and infection	Prostate	0.49	0.30	0.81	0.005	Radiotherapy & surgery
Peritonitis	Immunology and infection	Prostate	0.84	0.70	1.00	0.045	Radiotherapy & surgery
Chronic viral hepatitis	Immunology and infection	Prostate	1.04	0.58	1.87	0.888	Radiotherapy & surgery
Infection of bones and joints	Immunology and infection	Prostate	1.20	0.69	2.08	0.516	Radiotherapy & surgery
Infection of skin and subcutaneous tissues	Immunology and infection	Prostate	0.72	0.52	1.00	0.05	Radiotherapy & surgery
Infections of the digestive system	Immunology and infection	Prostate	0.90	0.69	1.17	0.42	Radiotherapy & surgery
Meningitis	Immunology and infection	Prostate	1.08	0.55	2.13	0.828	Radiotherapy & surgery
Viral diseases (excluding chronic hepatitis/HIV)	Immunology and infection	Prostate	1.18	0.73	1.89	0.506	Radiotherapy & surgery
Enthesopathies and synovial disorders	Musculoskeletal	Prostate	1.12	0.97	1.30	0.112	Radiotherapy & surgery
Osteoarthritis (excluding spine)	Musculoskeletal	Prostate	1.01	0.88	1.15	0.923	Radiotherapy & surgery
Osteoporosis	Musculoskeletal	Prostate	0.70	0.47	1.03	0.07	Radiotherapy & surgery
Rheumatoid arthritis	Musculoskeletal	Prostate	0.97	0.56	1.69	0.924	Radiotherapy & surgery
Spinal stenosis	Musculoskeletal	Prostate	0.90	0.58	1.38	0.62	Radiotherapy & surgery
Disorders of autonomic nervous system	Neurological	Prostate	0.93	0.48	1.78	0.819	Radiotherapy & surgery

Epilepsy	Neurological	Prostate	0.99	0.57	1.75	0.985	Radiotherapy & surgery
Intracerebral haemorrhage	Neurological	Prostate	1.01	0.56	1.83	0.972	Radiotherapy & surgery
Peripheral neuropathies	Neurological	Prostate	1.03	0.79	1.34	0.847	Radiotherapy & surgery
Asthma	Pulmonary	Prostate	1.08	0.86	1.35	0.517	Radiotherapy & surgery
COPD	Pulmonary	Prostate	0.98	0.82	1.19	0.865	Radiotherapy & surgery
End stage renal disease	Renal	Prostate	0.76	0.59	0.99	0.041	Radiotherapy & surgery
Obstructive and reflux uropathy	Renal	Prostate	0.77	0.60	0.99	0.038	Radiotherapy & surgery
Urolithiasis	Renal	Prostate	0.70	0.58	0.86	0.001	Radiotherapy & surgery
Glomerulonephritis	Renal	Prostate	1.26	0.82	1.95	0.294	Radiotherapy & surgery
Abdominal aortic aneurysm	Cardiovascular	Prostate	0.67	0.56	0.80	< 0.001	Radiotherapy alone
Heart failure	Cardiovascular	Prostate	0.79	0.67	0.93	0.005	Radiotherapy alone
Peripheral arterial disease	Cardiovascular	Prostate	0.75	0.61	0.93	0.01	Radiotherapy alone
Atrial fibrillation	Cardiovascular	Prostate	0.90	0.78	1.02	0.106	Radiotherapy alone
Atrioventricular block, complete	Cardiovascular	Prostate	0.78	0.58	1.05	0.105	Radiotherapy alone
Atrioventricular block, first degree	Cardiovascular	Prostate	0.78	0.58	1.05	0.105	Radiotherapy alone
Atrioventricular block, second degree	Cardiovascular	Prostate	0.78	0.58	1.05	0.105	Radiotherapy alone
Bifascicular block	Cardiovascular	Prostate	1.30	0.95	1.78	0.099	Radiotherapy alone
Coronary heart disease not otherwise specified	Cardiovascular	Prostate	1.10	0.99	1.21	0.064	Radiotherapy alone
Dilated cardiomyopathy	Cardiovascular	Prostate	1.14	0.64	2.04	0.652	Radiotherapy alone
Hypertension	Cardiovascular	Prostate	1.00	0.93	1.08	0.911	Radiotherapy alone
Hypertrophic cardiomyopathy	Cardiovascular	Prostate	1.05	0.58	1.91	0.864	Radiotherapy alone
Ischaemic stroke	Cardiovascular	Prostate	0.97	0.72	1.29	0.819	Radiotherapy alone
Left bundle branch block	Cardiovascular	Prostate	0.83	0.63	1.10	0.196	Radiotherapy alone
Multiple valve disease	Cardiovascular	Prostate	1.18	0.79	1.76	0.429	Radiotherapy alone
Myocardial infarction	Cardiovascular	Prostate	0.96	0.85	1.09	0.561	Radiotherapy alone
Nonrheumatic aortic valve disorders	Cardiovascular	Prostate	1.21	0.91	1.62	0.187	Radiotherapy alone
Nonrheumatic mitral valve disorders	Cardiovascular	Prostate	1.16	0.81	1.66	0.419	Radiotherapy alone
Other cardiomyopathy	Cardiovascular	Prostate	0.97	0.86	1.09	0.627	Radiotherapy alone
Pericardial effusion (noninflammatory)	Cardiovascular	Prostate	0.79	0.38	1.65	0.536	Radiotherapy alone
Raynaud's syndrome	Cardiovascular	Prostate	0.90	0.68	1.18	0.442	Radiotherapy alone
Right bundle branch block	Cardiovascular	Prostate	1.32	0.97	1.81	0.081	Radiotherapy alone
Sick sinus syndrome	Cardiovascular	Prostate	1.06	0.70	1.59	0.798	Radiotherapy alone
Stable angina	Cardiovascular	Prostate	1.14	0.99	1.30	0.059	Radiotherapy alone
Stroke (not otherwise specified)	Cardiovascular	Prostate	1.06	0.85	1.31	0.61	Radiotherapy alone
Supraventricular tachycardia	Cardiovascular	Prostate	0.79	0.52	1.20	0.268	Radiotherapy alone
Transient ischaemic attack	Cardiovascular	Prostate	1.12	0.94	1.32	0.206	Radiotherapy alone
Trifascicular block	Cardiovascular	Prostate	1.30	0.92	1.84	0.136	Radiotherapy alone
Unstable angina	Cardiovascular	Prostate	1.01	0.88	1.15	0.935	Radiotherapy alone
Venous thromboembolic disease	Cardiovascular	Prostate	0.90	0.74	1.10	0.301	Radiotherapy alone
Ventricular tachycardia	Cardiovascular	Prostate	0.79	0.53	1.19	0.264	Radiotherapy alone
Diabetic neurological complications	Endocrine	Prostate	0.83	0.73	0.96	0.009	Radiotherapy alone
Diabetic ophthalmic complications	Endocrine	Prostate	0.87	0.76	0.98	0.024	Radiotherapy alone
Diabetes	Endocrine	Prostate	1.01	0.91	1.13	0.816	Radiotherapy alone
Hypo or hyperthyroidism	Endocrine	Prostate	1.21	0.94	1.56	0.135	Radiotherapy alone
Barrett's oesophagus	Gastrointestinal	Prostate	0.75	0.59	0.95	0.017	Radiotherapy alone
Diverticular disease of intestine	Gastrointestinal	Prostate	0.86	0.74	0.99	0.035	Radiotherapy alone
Alcoholic liver disease	Gastrointestinal	Prostate	0.87	0.49	1.54	0.63	Radiotherapy alone
Angiodysplasia of colon	Gastrointestinal	Prostate	0.58	0.28	1.19	0.139	Radiotherapy alone
Cholangitis	Gastrointestinal	Prostate	1.00	0.79	1.27	0.972	Radiotherapy alone
Cholecystitis	Gastrointestinal	Prostate	0.95	0.75	1.20	0.654	Radiotherapy alone
Cholelithiasis	Gastrointestinal	Prostate	1.01	0.82	1.24	0.937	Radiotherapy alone
Coeliac disease	Gastrointestinal	Prostate	0.67	0.36	1.24	0.2	Radiotherapy alone
Fatty liver	Gastrointestinal	Prostate	0.81	0.56	1.15	0.238	Radiotherapy alone
Gastritis and duodenitis	Gastrointestinal	Prostate	1.08	0.94	1.26	0.281	Radiotherapy alone
Gastrooesophageal reflux disease	Gastrointestinal	Prostate	0.98	0.85	1.13	0.737	Radiotherapy alone
Hepatic failure	Gastrointestinal	Prostate	0.88	0.59	1.32	0.535	Radiotherapy alone
Liver fibrosis sclerosis and cirrhosis	Gastrointestinal	Prostate	0.89	0.46	1.72	0.722	Radiotherapy alone
Oesophagitis and oesophageal ulcer	Gastrointestinal	Prostate	0.99	0.87	1.13	0.873	Radiotherapy alone
Portal hypertension	Gastrointestinal	Prostate	1.06	0.66	1.71	0.811	Radiotherapy alone
Ulcerative colitis	Gastrointestinal	Prostate	1.03	0.57	1.88	0.922	Radiotherapy alone
Other anaemias	Haematological	Prostate	0.69	0.58	0.81	< 0.001	Radiotherapy alone
Agranulocytosis	Haematological	Prostate	1.30	0.59	2.86	0.516	Radiotherapy alone
Primary or idiopathic thrombocytopaenia	Haematological	Prostate	1.21	0.67	2.20	0.532	Radiotherapy alone
Secondary or other thrombocytopaenia	Haematological	Prostate	1.25	0.79	1.97	0.339	Radiotherapy alone
Thrombophilia	Haematological	Prostate	0.73	0.37	1.46	0.377	Radiotherapy alone
Infection of skin and subcutaneous tissues	Immunology and infection	Prostate	0.79	0.62	0.99	0.041	Radiotherapy alone
Infections of other or unspecified organs	Immunology and infection	Prostate	0.77	0.66	0.90	0.001	Radiotherapy alone
Infections of the digestive system	Immunology and infection	Prostate	0.80	0.66	0.98	0.03	Radiotherapy alone
Lower respiratory tract infections	Immunology and infection	Prostate	0.78	0.68	0.90	< 0.001	Radiotherapy alone
Non-acute cystitis	Immunology and infection	Prostate	0.30	0.19	0.46	< 0.001	Radiotherapy alone
Infection of anal and rectal regions	Immunology and infection	Prostate	2.43	1.17	5.03	0.017	Radiotherapy alone
Chronic viral hepatitis	Immunology and infection	Prostate	0.74	0.45	1.20	0.218	Radiotherapy alone
Infection of bones and joints	Immunology and infection	Prostate	1.37	0.93	2.03	0.116	Radiotherapy alone
Infections of the heart	Immunology and infection	Prostate	0.92	0.54	1.54	0.744	Radiotherapy alone
Meningitis	Immunology and infection	Prostate	0.74	0.43	1.28	0.276	Radiotherapy alone
Peritonitis	Immunology and infection	Prostate	0.89	0.78	1.01	0.078	Radiotherapy alone
Viral diseases (excluding chronic hepatitis/HIV)	Immunology and infection	Prostate	0.97	0.66	1.42	0.863	Radiotherapy alone
Osteoporosis	Musculoskeletal	Prostate	0.69	0.52	0.92	0.011	Radiotherapy alone
Enteropathic arthropathy	Musculoskeletal	Prostate	0.73	0.36	1.46	0.373	Radiotherapy alone

Enthesopathies and synovial disorders	Musculoskeletal	Prostate	1.09	0.97	1.22	0.132	Radiotherapy alone
Osteoarthritis (excluding spine)	Musculoskeletal	Prostate	1.08	0.98	1.19	0.115	Radiotherapy alone
Rheumatoid arthritis	Musculoskeletal	Prostate	1.15	0.77	1.71	0.496	Radiotherapy alone
Scoliosis	Musculoskeletal	Prostate	1.03	0.51	2.11	0.928	Radiotherapy alone
Spinal stenosis	Musculoskeletal	Prostate	0.84	0.62	1.15	0.282	Radiotherapy alone
Dementia	Neurological	Prostate	0.39	0.26	0.58	< 0.001	Radiotherapy alone
Disorders of autonomic nervous system	Neurological	Prostate	1.64	1.08	2.49	0.019	Radiotherapy alone
Bell's palsy	Neurological	Prostate	1.02	0.60	1.73	0.95	Radiotherapy alone
Epilepsy	Neurological	Prostate	0.86	0.55	1.34	0.497	Radiotherapy alone
Essential tremor	Neurological	Prostate	1.12	0.58	2.16	0.726	Radiotherapy alone
Intracerebral haemorrhage	Neurological	Prostate	0.80	0.50	1.27	0.342	Radiotherapy alone
Peripheral neuropathies	Neurological	Prostate	1.05	0.86	1.28	0.64	Radiotherapy alone
Subarachnoid haemorrhage	Neurological	Prostate	0.81	0.49	1.32	0.398	Radiotherapy alone
COPD	Pulmonary	Prostate	0.85	0.74	0.98	0.027	Radiotherapy alone
Asthma	Pulmonary	Prostate	0.95	0.80	1.14	0.608	Radiotherapy alone
Bronchiectasis	Pulmonary	Prostate	0.99	0.61	1.59	0.957	Radiotherapy alone
Other interstitial pulmonary diseases with fibrosis	Pulmonary	Prostate	1.06	0.62	1.83	0.824	Radiotherapy alone
End stage renal disease	Renal	Prostate	0.55	0.45	0.67	< 0.001	Radiotherapy alone
Obstructive and reflux uropathy	Renal	Prostate	0.35	0.28	0.44	< 0.001	Radiotherapy alone
Urolithiasis	Renal	Prostate	0.41	0.34	0.48	< 0.001	Radiotherapy alone
Glomerulonephritis	Renal	Prostate	0.68	0.46	1.00	0.05	Radiotherapy alone
Coronary heart disease not otherwise specified	Cardiovascular	Stomach	0.77	0.46	1.28	0.309	Chemotherapy & radiotherapy
Hypertension	Cardiovascular	Stomach	0.78	0.50	1.22	0.274	Chemotherapy & radiotherapy
Other cardiomyopathy	Cardiovascular	Stomach	0.59	0.31	1.11	0.099	Chemotherapy & radiotherapy
Diabetes	Endocrine	Stomach	1.75	1.11	2.78	0.017	Chemotherapy & radiotherapy
Diabetic neurological complications	Endocrine	Stomach	0.90	0.50	1.62	0.727	Chemotherapy & radiotherapy
Diabetic ophthalmic complications	Endocrine	Stomach	1.23	0.73	2.08	0.429	Chemotherapy & radiotherapy
Gastritis and duodenitis	Gastrointestinal	Stomach	0.51	0.31	0.87	0.013	Chemotherapy & radiotherapy
Barrett's oesophagus	Gastrointestinal	Stomach	1.89	1.16	3.07	0.011	Chemotherapy & radiotherapy
Gastrooesophageal reflux disease	Gastrointestinal	Stomach	0.87	0.52	1.47	0.604	Chemotherapy & radiotherapy
Oesophagitis and oesophageal ulcer	Gastrointestinal	Stomach	1.06	0.72	1.56	0.782	Chemotherapy & radiotherapy
Other anaemias	Haematological	Stomach	0.76	0.48	1.19	0.234	Chemotherapy & radiotherapy
Peritonitis	Immunology and infection	Stomach	0.51	0.32	0.81	0.004	Chemotherapy & radiotherapy
Lower respiratory tract infections	Immunology and infection	Stomach	0.94	0.55	1.61	0.814	Chemotherapy & radiotherapy
Enthesopathies and synovial disorders	Musculoskeletal	Stomach	1.57	0.90	2.73	0.114	Chemotherapy & radiotherapy
COPD	Pulmonary	Stomach	1.24	0.70	2.18	0.463	Chemotherapy & radiotherapy
Atrial fibrillation	Cardiovascular	Stomach	0.47	0.29	0.76	0.002	Chemotherapy & surgery
Heart failure	Cardiovascular	Stomach	0.58	0.36	0.95	0.029	Chemotherapy & surgery
Abdominal aortic aneurysm	Cardiovascular	Stomach	1.09	0.62	1.94	0.764	Chemotherapy & surgery
Coronary heart disease not otherwise specified	Cardiovascular	Stomach	0.76	0.56	1.03	0.073	Chemotherapy & surgery
Hypertension	Cardiovascular	Stomach	1.08	0.85	1.37	0.519	Chemotherapy & surgery
Multiple valve disease	Cardiovascular	Stomach	1.76	0.86	3.61	0.123	Chemotherapy & surgery
Myocardial infarction	Cardiovascular	Stomach	0.80	0.56	1.15	0.233	Chemotherapy & surgery
Other cardiomyopathy	Cardiovascular	Stomach	0.79	0.56	1.12	0.191	Chemotherapy & surgery
Peripheral arterial disease	Cardiovascular	Stomach	0.73	0.39	1.34	0.303	Chemotherapy & surgery
Raynaud's syndrome	Cardiovascular	Stomach	0.78	0.41	1.49	0.447	Chemotherapy & surgery
Stable angina	Cardiovascular	Stomach	1.06	0.73	1.52	0.77	Chemotherapy & surgery
Stroke (not otherwise specified)	Cardiovascular	Stomach	0.83	0.46	1.52	0.555	Chemotherapy & surgery
Transient ischaemic attack	Cardiovascular	Stomach	0.71	0.40	1.27	0.25	Chemotherapy & surgery
Unstable angina	Cardiovascular	Stomach	0.85	0.57	1.29	0.449	Chemotherapy & surgery
Venous thromboembolic disease	Cardiovascular	Stomach	1.00	0.64	1.55	0.983	Chemotherapy & surgery
Diabetes	Endocrine	Stomach	1.22	0.89	1.67	0.22	Chemotherapy & surgery
Diabetic neurological complications	Endocrine	Stomach	0.96	0.67	1.36	0.815	Chemotherapy & surgery
Diabetic ophthalmic complications	Endocrine	Stomach	0.88	0.62	1.26	0.493	Chemotherapy & surgery
Hypo or hyperthyroidism	Endocrine	Stomach	0.71	0.38	1.35	0.299	Chemotherapy & surgery
Gastritis and duodenitis	Gastrointestinal	Stomach	0.75	0.57	0.99	0.041	Chemotherapy & surgery
Barrett's oesophagus	Gastrointestinal	Stomach	0.75	0.50	1.13	0.171	Chemotherapy & surgery
Cholangitis	Gastrointestinal	Stomach	1.03	0.62	1.73	0.902	Chemotherapy & surgery
Cholecystitis	Gastrointestinal	Stomach	0.74	0.43	1.30	0.297	Chemotherapy & surgery
Cholelithiasis	Gastrointestinal	Stomach	0.89	0.55	1.47	0.661	Chemotherapy & surgery
Diverticular disease of intestine	Gastrointestinal	Stomach	1.00	0.68	1.46	0.997	Chemotherapy & surgery
Gastrooesophageal reflux disease	Gastrointestinal	Stomach	1.01	0.74	1.37	0.962	Chemotherapy & surgery
Oesophagitis and oesophageal ulcer	Gastrointestinal	Stomach	0.81	0.63	1.05	0.109	Chemotherapy & surgery
Other anaemias	Haematological	Stomach	0.72	0.55	0.94	0.016	Chemotherapy & surgery
Infections of other or unspecified organs	Immunology and infection	Stomach	0.66	0.45	0.95	0.027	Chemotherapy & surgery
Lower respiratory tract infections	Immunology and infection	Stomach	0.59	0.41	0.86	0.006	Chemotherapy & surgery
Peritonitis	Immunology and infection	Stomach	0.69	0.54	0.88	0.003	Chemotherapy & surgery
Infections of the digestive system	Immunology and infection	Stomach	0.88	0.56	1.38	0.582	Chemotherapy & surgery
Enthesopathies and synovial disorders	Musculoskeletal	Stomach	1.54	1.08	2.19	0.016	Chemotherapy & surgery
Osteoarthritis (excluding spine)	Musculoskeletal	Stomach	1.34	0.99	1.81	0.056	Chemotherapy & surgery
Osteoporosis	Musculoskeletal	Stomach	1.27	0.76	2.13	0.363	Chemotherapy & surgery
Peripheral neuropathies	Neurological	Stomach	1.46	0.84	2.56	0.18	Chemotherapy & surgery
Asthma	Pulmonary	Stomach	0.74	0.45	1.24	0.259	Chemotherapy & surgery
COPD	Pulmonary	Stomach	1.11	0.78	1.59	0.562	Chemotherapy & surgery
Heart failure	Cardiovascular	Stomach	0.50	0.34	0.76	0.001	Chemotherapy alone
Myocardial infarction	Cardiovascular	Stomach	0.73	0.54	0.99	0.046	Chemotherapy alone
Raynaud's syndrome	Cardiovascular	Stomach	0.45	0.24	0.86	0.015	Chemotherapy alone
Stroke (not otherwise specified)	Cardiovascular	Stomach	0.56	0.32	0.96	0.036	Chemotherapy alone
Venous thromboembolic disease	Cardiovascular	Stomach	0.63	0.41	0.95	0.028	Chemotherapy alone
Abdominal aortic aneurysm	Cardiovascular	Stomach	0.74	0.44	1.27	0.277	Chemotherapy alone
Atrial fibrillation	Cardiovascular	Stomach	0.74	0.54	1.03	0.072	Chemotherapy alone

Coronary heart disease not otherwise specified	Cardiovascular	Stomach	0.85	0.67	1.08	0.186	Chemotherapy alone
Hypertension	Cardiovascular	Stomach	1.06	0.87	1.28	0.584	Chemotherapy alone
Left bundle branch block	Cardiovascular	Stomach	0.76	0.38	1.55	0.455	Chemotherapy alone
Other cardiomyopathy	Cardiovascular	Stomach	0.83	0.62	1.10	0.192	Chemotherapy alone
Peripheral arterial disease	Cardiovascular	Stomach	0.61	0.36	1.03	0.062	Chemotherapy alone
Stable angina	Cardiovascular	Stomach	0.86	0.62	1.17	0.334	Chemotherapy alone
Transient ischaemic attack	Cardiovascular	Stomach	0.66	0.41	1.06	0.086	Chemotherapy alone
Unstable angina	Cardiovascular	Stomach	0.81	0.58	1.14	0.224	Chemotherapy alone
Diabetic ophthalmic complications	Endocrine	Stomach	0.74	0.55	1.00	0.049	Chemotherapy alone
Diabetes	Endocrine	Stomach	1.03	0.79	1.35	0.814	Chemotherapy alone
Diabetic neurological complications	Endocrine	Stomach	0.80	0.59	1.08	0.146	Chemotherapy alone
Hypo or hyperthyroidism	Endocrine	Stomach	0.58	0.33	1.01	0.053	Chemotherapy alone
Gastritis and duodenitis	Gastrointestinal	Stomach	0.71	0.57	0.89	0.003	Chemotherapy alone
Barrett's oesophagus	Gastrointestinal	Stomach	0.92	0.67	1.26	0.606	Chemotherapy alone
Cholangitis	Gastrointestinal	Stomach	0.89	0.58	1.39	0.62	Chemotherapy alone
Cholecystitis	Gastrointestinal	Stomach	0.69	0.44	1.09	0.11	Chemotherapy alone
Cholelithiasis	Gastrointestinal	Stomach	0.88	0.59	1.32	0.537	Chemotherapy alone
Diverticular disease of intestine	Gastrointestinal	Stomach	1.04	0.77	1.41	0.795	Chemotherapy alone
Fatty liver	Gastrointestinal	Stomach	0.67	0.36	1.23	0.198	Chemotherapy alone
Gastrooesophageal reflux disease	Gastrointestinal	Stomach	1.09	0.86	1.40	0.473	Chemotherapy alone
Hepatic failure	Gastrointestinal	Stomach	0.53	0.27	1.03	0.06	Chemotherapy alone
Oesophagitis and oesophageal ulcer	Gastrointestinal	Stomach	0.88	0.72	1.08	0.234	Chemotherapy alone
Portal hypertension	Gastrointestinal	Stomach	0.64	0.31	1.30	0.216	Chemotherapy alone
Other anaemias	Haematological	Stomach	0.57	0.46	0.72	< 0.001	Chemotherapy alone
Infections of other or unspecified organs	Immunology and infection	Stomach	0.57	0.42	0.78	< 0.001	Chemotherapy alone
Lower respiratory tract infections	Immunology and infection	Stomach	0.71	0.54	0.94	0.018	Chemotherapy alone
Peritonitis	Immunology and infection	Stomach	0.58	0.47	0.71	< 0.001	Chemotherapy alone
Infection of skin and subcutaneous tissues	Immunology and infection	Stomach	0.71	0.44	1.16	0.174	Chemotherapy alone
Infections of the digestive system	Immunology and infection	Stomach	0.84	0.58	1.21	0.345	Chemotherapy alone
Enthesopathies and synovial disorders	Musculoskeletal	Stomach	1.05	0.76	1.45	0.77	Chemotherapy alone
Osteoarthritis (excluding spine)	Musculoskeletal	Stomach	1.09	0.84	1.41	0.515	Chemotherapy alone
Osteoporosis	Musculoskeletal	Stomach	0.80	0.50	1.29	0.356	Chemotherapy alone
Peripheral neuropathies	Neurological	Stomach	1.31	0.82	2.12	0.261	Chemotherapy alone
Asthma	Pulmonary	Stomach	1.07	0.74	1.54	0.736	Chemotherapy alone
COPD	Pulmonary	Stomach	0.79	0.57	1.08	0.143	Chemotherapy alone
End stage renal disease	Renal	Stomach	0.53	0.32	0.87	0.013	Chemotherapy alone
Urolithiasis	Renal	Stomach	0.51	0.28	0.94	0.031	Chemotherapy alone
Hypertension	Cardiovascular	Stomach	0.92	0.57	1.48	0.729	Chemotherapy, radiotherapy & surgery
Diabetes	Endocrine	Stomach	1.25	0.68	2.27	0.475	Chemotherapy, radiotherapy & surgery
Diabetic neurological complications	Endocrine	Stomach	1.23	0.65	2.34	0.52	Chemotherapy, radiotherapy & surgery
Diabetic ophthalmic complications	Endocrine	Stomach	1.27	0.69	2.36	0.446	Chemotherapy, radiotherapy & surgery
Gastritis and duodenitis	Gastrointestinal	Stomach	0.86	0.52	1.41	0.543	Chemotherapy, radiotherapy & surgery
Gastrooesophageal reflux disease	Gastrointestinal	Stomach	1.43	0.85	2.41	0.174	Chemotherapy, radiotherapy & surgery
Oesophagitis and oesophageal ulcer	Gastrointestinal	Stomach	0.77	0.48	1.26	0.303	Chemotherapy, radiotherapy & surgery
Other anaemias	Haematological	Stomach	0.59	0.34	1.05	0.073	Chemotherapy, radiotherapy & surgery
Lower respiratory tract infections	Immunology and infection	Stomach	1.00	0.54	1.89	0.989	Chemotherapy, radiotherapy & surgery
Peritonitis	Immunology and infection	Stomach	0.61	0.37	1.01	0.053	Chemotherapy, radiotherapy & surgery
Transient ischaemic attack	Cardiovascular	Stomach	1.83	1.03	3.25	0.041	Radiotherapy & surgery
Atrial fibrillation	Cardiovascular	Stomach	0.60	0.33	1.10	0.098	Radiotherapy & surgery
Coronary heart disease not otherwise specified	Cardiovascular	Stomach	0.85	0.54	1.35	0.498	Radiotherapy & surgery
Heart failure	Cardiovascular	Stomach	0.69	0.37	1.28	0.237	Radiotherapy & surgery
Hypertension	Cardiovascular	Stomach	1.00	0.68	1.46	0.984	Radiotherapy & surgery
Myocardial infarction	Cardiovascular	Stomach	0.88	0.54	1.46	0.63	Radiotherapy & surgery
Other cardiomyopathy	Cardiovascular	Stomach	0.95	0.59	1.52	0.816	Radiotherapy & surgery
Stable angina	Cardiovascular	Stomach	0.67	0.36	1.23	0.197	Radiotherapy & surgery
Unstable angina	Cardiovascular	Stomach	1.04	0.61	1.78	0.876	Radiotherapy & surgery
Diabetic neurological complications	Endocrine	Stomach	1.63	1.04	2.56	0.033	Radiotherapy & surgery
Diabetes	Endocrine	Stomach	1.35	0.84	2.16	0.215	Radiotherapy & surgery
Diabetic ophthalmic complications	Endocrine	Stomach	1.45	0.93	2.27	0.102	Radiotherapy & surgery
Barrett's oesophagus	Gastrointestinal	Stomach	1.74	1.05	2.87	0.031	Radiotherapy & surgery
Diverticular disease of intestine	Gastrointestinal	Stomach	0.89	0.49	1.61	0.71	Radiotherapy & surgery
Gastritis and duodenitis	Gastrointestinal	Stomach	0.73	0.46	1.14	0.168	Radiotherapy & surgery
Gastrooesophageal reflux disease	Gastrointestinal	Stomach	0.85	0.50	1.45	0.544	Radiotherapy & surgery
Oesophagitis and oesophageal ulcer	Gastrointestinal	Stomach	1.13	0.77	1.66	0.536	Radiotherapy & surgery
Other anaemias	Haematological	Stomach	0.93	0.64	1.36	0.721	Radiotherapy & surgery
Peritonitis	Immunology and infection	Stomach	0.63	0.42	0.95	0.026	Radiotherapy & surgery
Infections of other or unspecified organs	Immunology and infection	Stomach	0.63	0.36	1.12	0.113	Radiotherapy & surgery
Lower respiratory tract infections	Immunology and infection	Stomach	0.74	0.45	1.21	0.229	Radiotherapy & surgery
Enthesopathies and synovial disorders	Musculoskeletal	Stomach	1.56	0.90	2.70	0.114	Radiotherapy & surgery
Osteoarthritis (excluding spine)	Musculoskeletal	Stomach	1.14	0.71	1.84	0.597	Radiotherapy & surgery
COPD	Pulmonary	Stomach	0.66	0.35	1.24	0.198	Radiotherapy & surgery
Atrial fibrillation	Cardiovascular	Stomach	0.84	0.54	1.31	0.444	Radiotherapy alone
Coronary heart disease not otherwise specified	Cardiovascular	Stomach	1.23	0.88	1.73	0.231	Radiotherapy alone
Heart failure	Cardiovascular	Stomach	0.82	0.50	1.35	0.44	Radiotherapy alone
Hypertension	Cardiovascular	Stomach	0.89	0.64	1.23	0.469	Radiotherapy alone
Ischaemic stroke	Cardiovascular	Stomach	1.82	0.95	3.49	0.072	Radiotherapy alone
Myocardial infarction	Cardiovascular	Stomach	1.20	0.81	1.78	0.364	Radiotherapy alone
Other cardiomyopathy	Cardiovascular	Stomach	1.19	0.81	1.74	0.384	Radiotherapy alone
Stable angina	Cardiovascular	Stomach	0.69	0.41	1.16	0.16	Radiotherapy alone
Stroke (not otherwise specified)	Cardiovascular	Stomach	1.59	0.91	2.79	0.104	Radiotherapy alone
Transient ischaemic attack	Cardiovascular	Stomach	1.05	0.58	1.88	0.874	Radiotherapy alone

Unstable angina	Cardiovascular	Stomach	1.25	0.81	1.92	0.315	Radiotherapy alone
Venous thromboembolic disease	Cardiovascular	Stomach	1.17	0.69	1.96	0.563	Radiotherapy alone
Diabetes	Endocrine	Stomach	1.22	0.81	1.82	0.348	Radiotherapy alone
Diabetic neurological complications	Endocrine	Stomach	0.96	0.62	1.49	0.862	Radiotherapy alone
Diabetic ophthalmic complications	Endocrine	Stomach	1.12	0.74	1.67	0.597	Radiotherapy alone
Barrett's oesophagus	Gastrointestinal	Stomach	1.45	0.94	2.23	0.096	Radiotherapy alone
Diverticular disease of intestine	Gastrointestinal	Stomach	0.92	0.57	1.47	0.721	Radiotherapy alone
Gastritis and duodenitis	Gastrointestinal	Stomach	0.80	0.56	1.14	0.224	Radiotherapy alone
Gastrooesophageal reflux disease	Gastrointestinal	Stomach	1.12	0.75	1.67	0.572	Radiotherapy alone
Oesophagitis and oesophageal ulcer	Gastrointestinal	Stomach	0.96	0.70	1.34	0.83	Radiotherapy alone
Other anaemias	Haematological	Stomach	1.17	0.87	1.57	0.303	Radiotherapy alone
Infections of other or unspecified organs	Immunology and infection	Stomach	0.56	0.34	0.92	0.023	Radiotherapy alone
Peritonitis	Immunology and infection	Stomach	0.70	0.51	0.97	0.032	Radiotherapy alone
Infections of the digestive system	Immunology and infection	Stomach	0.60	0.32	1.13	0.112	Radiotherapy alone
Lower respiratory tract infections	Immunology and infection	Stomach	1.03	0.70	1.51	0.878	Radiotherapy alone
Enthesopathies and synovial disorders	Musculoskeletal	Stomach	1.11	0.67	1.86	0.685	Radiotherapy alone
Osteoarthritis (excluding spine)	Musculoskeletal	Stomach	0.70	0.44	1.12	0.134	Radiotherapy alone
Osteoporosis	Musculoskeletal	Stomach	1.44	0.78	2.64	0.241	Radiotherapy alone
Asthma	Pulmonary	Stomach	1.28	0.74	2.20	0.372	Radiotherapy alone
COPD	Pulmonary	Stomach	0.87	0.54	1.41	0.574	Radiotherapy alone
End stage renal disease	Renal	Stomach	0.99	0.55	1.79	0.983	Radiotherapy alone
Coronary heart disease not otherwise specified	Cardiovascular	Testis	0.49	0.25	0.96	0.039	Chemotherapy & surgery
Hypertension	Cardiovascular	Testis	1.02	0.56	1.86	0.939	Chemotherapy & surgery
Diabetes	Endocrine	Testis	1.63	0.80	3.34	0.18	Chemotherapy & surgery
Enthesopathies and synovial disorders	Musculoskeletal	Testis	0.94	0.56	1.59	0.822	Chemotherapy & surgery
Hypertension	Cardiovascular	Testis	1.12	0.56	2.25	0.745	Chemotherapy alone
Urolithiasis	Renal	Testis	0.48	0.38	0.60	< 0.001	Chemotherapy alone
Hypertension	Cardiovascular	Testis	1.73	0.85	3.52	0.128	Radiotherapy & surgery
Enthesopathies and synovial disorders	Musculoskeletal	Testis	1.51	0.78	2.90	0.219	Radiotherapy & surgery
Hypo or hyperthyroidism	Endocrine	Thyroid	1.71	0.91	3.21	0.096	Chemotherapy & surgery
Atrial fibrillation	Cardiovascular	Thyroid	0.49	0.24	0.98	0.044	Radiotherapy & surgery
Coronary heart disease not otherwise specified	Cardiovascular	Thyroid	1.11	0.73	1.68	0.633	Radiotherapy & surgery
Hypertension	Cardiovascular	Thyroid	1.23	0.89	1.72	0.214	Radiotherapy & surgery
Myocardial infarction	Cardiovascular	Thyroid	1.03	0.52	2.03	0.939	Radiotherapy & surgery
Other cardiomyopathy	Cardiovascular	Thyroid	0.83	0.42	1.66	0.599	Radiotherapy & surgery
Stable angina	Cardiovascular	Thyroid	1.42	0.73	2.79	0.304	Radiotherapy & surgery
Unstable angina	Cardiovascular	Thyroid	1.01	0.48	2.11	0.985	Radiotherapy & surgery
Hypo or hyperthyroidism	Endocrine	Thyroid	0.71	0.54	0.93	0.012	Radiotherapy & surgery
Diabetes	Endocrine	Thyroid	0.93	0.58	1.49	0.773	Radiotherapy & surgery
Diabetic neurological complications	Endocrine	Thyroid	1.14	0.64	2.05	0.654	Radiotherapy & surgery
Diabetic ophthalmic complications	Endocrine	Thyroid	1.09	0.64	1.88	0.745	Radiotherapy & surgery
Cholangitis	Gastrointestinal	Thyroid	1.36	0.63	2.90	0.432	Radiotherapy & surgery
Cholecystitis	Gastrointestinal	Thyroid	1.47	0.70	3.08	0.305	Radiotherapy & surgery
Cholelithiasis	Gastrointestinal	Thyroid	1.72	0.87	3.42	0.12	Radiotherapy & surgery
Gastritis and duodenitis	Gastrointestinal	Thyroid	0.65	0.32	1.30	0.225	Radiotherapy & surgery
Gastrooesophageal reflux disease	Gastrointestinal	Thyroid	0.69	0.39	1.24	0.213	Radiotherapy & surgery
Oesophagitis and oesophageal ulcer	Gastrointestinal	Thyroid	0.74	0.42	1.31	0.299	Radiotherapy & surgery
Lower respiratory tract infections	Immunology and infection	Thyroid	0.55	0.29	1.08	0.081	Radiotherapy & surgery
Peritonitis	Immunology and infection	Thyroid	0.62	0.32	1.20	0.156	Radiotherapy & surgery
Enthesopathies and synovial disorders	Musculoskeletal	Thyroid	1.06	0.68	1.65	0.789	Radiotherapy & surgery
Osteoarthritis (excluding spine)	Musculoskeletal	Thyroid	1.12	0.70	1.78	0.635	Radiotherapy & surgery
Osteoporosis	Musculoskeletal	Thyroid	1.11	0.53	2.29	0.787	Radiotherapy & surgery
Peripheral neuropathies	Neurological	Thyroid	1.84	0.96	3.51	0.066	Radiotherapy & surgery
Asthma	Pulmonary	Thyroid	0.91	0.52	1.59	0.744	Radiotherapy & surgery
COPD	Pulmonary	Thyroid	1.08	0.60	1.94	0.799	Radiotherapy & surgery
Coronary heart disease not otherwise specified	Cardiovascular	Thyroid	1.24	0.69	2.22	0.47	Radiotherapy alone
Hypertension	Cardiovascular	Thyroid	0.84	0.51	1.40	0.51	Radiotherapy alone
Diabetes	Endocrine	Thyroid	0.88	0.47	1.67	0.705	Radiotherapy alone
Diabetic ophthalmic complications	Endocrine	Thyroid	1.20	0.60	2.42	0.607	Radiotherapy alone
Hypo or hyperthyroidism	Endocrine	Thyroid	0.69	0.45	1.06	0.09	Radiotherapy alone
Oesophagitis and oesophageal ulcer	Gastrointestinal	Thyroid	1.07	0.53	2.14	0.859	Radiotherapy alone
Other anaemias	Haematological	Thyroid	1.32	0.60	2.93	0.491	Radiotherapy alone
Lower respiratory tract infections	Immunology and infection	Thyroid	1.11	0.57	2.16	0.75	Radiotherapy alone
Peritonitis	Immunology and infection	Thyroid	0.98	0.48	2.02	0.967	Radiotherapy alone
Enthesopathies and synovial disorders	Musculoskeletal	Thyroid	1.81	1.06	3.07	0.029	Radiotherapy alone
Osteoarthritis (excluding spine)	Musculoskeletal	Thyroid	0.86	0.44	1.68	0.667	Radiotherapy alone
COPD	Pulmonary	Thyroid	1.32	0.65	2.67	0.441	Radiotherapy alone
Hypertension	Cardiovascular	Uterus	1.14	0.77	1.71	0.51	Chemotherapy & radiotherapy
Diabetes	Endocrine	Uterus	0.85	0.50	1.44	0.547	Chemotherapy & radiotherapy
Diabetic neurological complications	Endocrine	Uterus	1.01	0.56	1.80	0.979	Chemotherapy & radiotherapy
Diabetic ophthalmic complications	Endocrine	Uterus	0.97	0.55	1.73	0.921	Chemotherapy & radiotherapy
Other anaemias	Haematological	Uterus	1.05	0.54	2.04	0.885	Chemotherapy & radiotherapy
Enthesopathies and synovial disorders	Musculoskeletal	Uterus	1.18	0.65	2.13	0.595	Chemotherapy & radiotherapy
Osteoarthritis (excluding spine)	Musculoskeletal	Uterus	0.82	0.47	1.43	0.482	Chemotherapy & radiotherapy
Atrial fibrillation	Cardiovascular	Uterus	1.15	0.69	1.91	0.597	Chemotherapy & surgery
Coronary heart disease not otherwise specified	Cardiovascular	Uterus	0.78	0.50	1.20	0.255	Chemotherapy & surgery
Hypertension	Cardiovascular	Uterus	1.21	0.92	1.60	0.174	Chemotherapy & surgery
Myocardial infarction	Cardiovascular	Uterus	0.89	0.48	1.67	0.721	Chemotherapy & surgery
Other cardiomyopathy	Cardiovascular	Uterus	0.85	0.45	1.59	0.605	Chemotherapy & surgery

Diabetes	Endocrine	Uterus	0.78	0.53	1.13	0.184	Chemotherapy & surgery
Diabetic neurological complications	Endocrine	Uterus	0.91	0.60	1.38	0.668	Chemotherapy & surgery
Diabetic ophthalmic complications	Endocrine	Uterus	0.94	0.63	1.40	0.757	Chemotherapy & surgery
Hypo or hyperthyroidism	Endocrine	Uterus	0.89	0.50	1.57	0.691	Chemotherapy & surgery
Diverticular disease of intestine	Gastrointestinal	Uterus	1.47	0.92	2.36	0.109	Chemotherapy & surgery
Gastrooesophageal reflux disease	Gastrointestinal	Uterus	1.00	0.56	1.78	0.998	Chemotherapy & surgery
Oesophagitis and oesophageal ulcer	Gastrointestinal	Uterus	0.88	0.51	1.54	0.664	Chemotherapy & surgery
Other anaemias	Haematological	Uterus	1.26	0.81	1.98	0.309	Chemotherapy & surgery
Infections of other or unspecified organs	Immunology and infection	Uterus	0.91	0.50	1.66	0.761	Chemotherapy & surgery
Lower respiratory tract infections	Immunology and infection	Uterus	0.91	0.51	1.62	0.745	Chemotherapy & surgery
Peritonitis	Immunology and infection	Uterus	1.34	0.85	2.12	0.2	Chemotherapy & surgery
Enthesopathies and synovial disorders	Musculoskeletal	Uterus	0.88	0.54	1.41	0.584	Chemotherapy & surgery
Osteoarthritis (excluding spine)	Musculoskeletal	Uterus	1.12	0.79	1.59	0.529	Chemotherapy & surgery
Asthma	Pulmonary	Uterus	0.90	0.49	1.66	0.744	Chemotherapy & surgery
Atrial fibrillation	Cardiovascular	Uterus	1.10	0.64	1.90	0.724	Chemotherapy alone
Coronary heart disease not otherwise specified	Cardiovascular	Uterus	0.88	0.54	1.41	0.59	Chemotherapy alone
Heart failure	Cardiovascular	Uterus	1.58	0.89	2.77	0.115	Chemotherapy alone
Hypertension	Cardiovascular	Uterus	0.80	0.55	1.15	0.222	Chemotherapy alone
Myocardial infarction	Cardiovascular	Uterus	1.24	0.69	2.25	0.472	Chemotherapy alone
Other cardiomyopathy	Cardiovascular	Uterus	1.27	0.71	2.26	0.421	Chemotherapy alone
Transient ischaemic attack	Cardiovascular	Uterus	1.21	0.60	2.42	0.59	Chemotherapy alone
Unstable angina	Cardiovascular	Uterus	0.77	0.39	1.51	0.449	Chemotherapy alone
Venous thromboembolic disease	Cardiovascular	Uterus	1.13	0.58	2.21	0.711	Chemotherapy alone
Diabetes	Endocrine	Uterus	0.62	0.40	0.98	0.039	Chemotherapy alone
Diabetic neurological complications	Endocrine	Uterus	0.73	0.46	1.17	0.192	Chemotherapy alone
Diabetic ophthalmic complications	Endocrine	Uterus	0.88	0.57	1.36	0.565	Chemotherapy alone
Hypo or hyperthyroidism	Endocrine	Uterus	0.94	0.51	1.73	0.847	Chemotherapy alone
Diverticular disease of intestine	Gastrointestinal	Uterus	0.95	0.52	1.72	0.861	Chemotherapy alone
Oesophagitis and oesophageal ulcer	Gastrointestinal	Uterus	0.77	0.40	1.49	0.443	Chemotherapy alone
Other anaemias	Haematological	Uterus	0.78	0.45	1.36	0.385	Chemotherapy alone
Infection of skin and subcutaneous tissues	Immunology and infection	Uterus	1.27	0.67	2.39	0.468	Chemotherapy alone
Infections of other or unspecified organs	Immunology and infection	Uterus	1.37	0.79	2.38	0.259	Chemotherapy alone
Lower respiratory tract infections	Immunology and infection	Uterus	1.57	0.94	2.61	0.085	Chemotherapy alone
Peritonitis	Immunology and infection	Uterus	0.81	0.45	1.43	0.46	Chemotherapy alone
Enthesopathies and synovial disorders	Musculoskeletal	Uterus	0.88	0.52	1.49	0.628	Chemotherapy alone
Osteoarthritis (excluding spine)	Musculoskeletal	Uterus	0.85	0.56	1.29	0.45	Chemotherapy alone
Asthma	Pulmonary	Uterus	1.37	0.76	2.46	0.3	Chemotherapy alone
COPD	Pulmonary	Uterus	1.24	0.65	2.37	0.511	Chemotherapy alone
End stage renal disease	Renal	Uterus	2.43	1.24	4.75	0.01	Chemotherapy alone
Coronary heart disease not otherwise specified	Cardiovascular	Uterus	1.15	0.75	1.78	0.518	Chemotherapy, radiotherapy & surgery
Hypertension	Cardiovascular	Uterus	0.76	0.53	1.10	0.145	Chemotherapy, radiotherapy & surgery
Diabetes	Endocrine	Uterus	0.63	0.40	0.99	0.047	Chemotherapy, radiotherapy & surgery
Diabetic neurological complications	Endocrine	Uterus	0.55	0.31	0.98	0.043	Chemotherapy, radiotherapy & surgery
Diabetic ophthalmic complications	Endocrine	Uterus	0.85	0.52	1.39	0.516	Chemotherapy, radiotherapy & surgery
Hypo or hyperthyroidism	Endocrine	Uterus	1.05	0.58	1.91	0.875	Chemotherapy, radiotherapy & surgery
Cholecystitis	Gastrointestinal	Uterus	1.75	0.89	3.46	0.105	Chemotherapy, radiotherapy & surgery
Diverticular disease of intestine	Gastrointestinal	Uterus	1.40	0.80	2.46	0.239	Chemotherapy, radiotherapy & surgery
Oesophagitis and oesophageal ulcer	Gastrointestinal	Uterus	0.95	0.51	1.76	0.86	Chemotherapy, radiotherapy & surgery
Peritonitis	Immunology and infection	Uterus	1.11	0.63	1.94	0.718	Chemotherapy, radiotherapy & surgery
Enthesopathies and synovial disorders	Musculoskeletal	Uterus	1.10	0.67	1.79	0.708	Chemotherapy, radiotherapy & surgery
Osteoarthritis (excluding spine)	Musculoskeletal	Uterus	0.81	0.52	1.26	0.345	Chemotherapy, radiotherapy & surgery
Asthma	Pulmonary	Uterus	1.43	0.80	2.56	0.227	Chemotherapy, radiotherapy & surgery
Hypertension	Cardiovascular	Uterus	1.17	1.00	1.37	0.049	Radiotherapy & surgery
Atrial fibrillation	Cardiovascular	Uterus	0.77	0.55	1.08	0.131	Radiotherapy & surgery
Atrioventricular block, complete	Cardiovascular	Uterus	1.37	0.64	2.93	0.418	Radiotherapy & surgery
Atrioventricular block, first degree	Cardiovascular	Uterus	1.37	0.64	2.93	0.418	Radiotherapy & surgery
Atrioventricular block, second degree	Cardiovascular	Uterus	1.37	0.64	2.93	0.418	Radiotherapy & surgery
Coronary heart disease not otherwise specified	Cardiovascular	Uterus	1.21	0.97	1.50	0.09	Radiotherapy & surgery
Heart failure	Cardiovascular	Uterus	0.66	0.43	1.00	0.051	Radiotherapy & surgery
Left bundle branch block	Cardiovascular	Uterus	1.68	0.82	3.41	0.154	Radiotherapy & surgery
Myocardial infarction	Cardiovascular	Uterus	0.85	0.58	1.24	0.388	Radiotherapy & surgery
Nonrheumatic aortic valve disorders	Cardiovascular	Uterus	1.21	0.57	2.57	0.627	Radiotherapy & surgery
Other cardiomyopathy	Cardiovascular	Uterus	0.85	0.59	1.25	0.414	Radiotherapy & surgery
Peripheral arterial disease	Cardiovascular	Uterus	1.04	0.58	1.86	0.895	Radiotherapy & surgery
Stable angina	Cardiovascular	Uterus	0.90	0.62	1.31	0.588	Radiotherapy & surgery
Stroke (not otherwise specified)	Cardiovascular	Uterus	0.64	0.36	1.16	0.141	Radiotherapy & surgery
Transient ischaemic attack	Cardiovascular	Uterus	0.68	0.43	1.07	0.096	Radiotherapy & surgery
Unstable angina	Cardiovascular	Uterus	0.78	0.53	1.16	0.218	Radiotherapy & surgery
Venous thromboembolic disease	Cardiovascular	Uterus	1.07	0.71	1.61	0.759	Radiotherapy & surgery
Diabetes	Endocrine	Uterus	0.98	0.80	1.19	0.817	Radiotherapy & surgery
Diabetic neurological complications	Endocrine	Uterus	0.94	0.74	1.20	0.633	Radiotherapy & surgery
Diabetic ophthalmic complications	Endocrine	Uterus	1.06	0.84	1.33	0.615	Radiotherapy & surgery
Hypo or hyperthyroidism	Endocrine	Uterus	1.10	0.81	1.48	0.552	Radiotherapy & surgery
Barrett's oesophagus	Gastrointestinal	Uterus	1.03	0.52	2.06	0.933	Radiotherapy & surgery
Cholangitis	Gastrointestinal	Uterus	1.16	0.77	1.76	0.48	Radiotherapy & surgery
Cholecystitis	Gastrointestinal	Uterus	1.14	0.76	1.72	0.513	Radiotherapy & surgery
Cholelithiasis	Gastrointestinal	Uterus	1.25	0.87	1.80	0.228	Radiotherapy & surgery
Diverticular disease of intestine	Gastrointestinal	Uterus	0.96	0.69	1.32	0.782	Radiotherapy & surgery
Gastritis and duodenitis	Gastrointestinal	Uterus	0.72	0.48	1.08	0.112	Radiotherapy & surgery
Gastrooesophageal reflux disease	Gastrointestinal	Uterus	0.99	0.71	1.38	0.963	Radiotherapy & surgery
Oesophagitis and oesophageal ulcer	Gastrointestinal	Uterus	1.02	0.75	1.38	0.898	Radiotherapy & surgery

Other anaemias	Haematological	Uterus	0.76	0.54	1.06	0.104	Radiotherapy & surgery
Viral diseases (excluding chronic hepatitis/HIV)	Immunology and infection	Uterus	0.00	0.00	0.00	< 0.001	Radiotherapy & surgery
Lower respiratory tract infections	Immunology and infection	Uterus	0.68	0.46	0.99	0.045	Radiotherapy & surgery
Infection of skin and subcutaneous tissues	Immunology and infection	Uterus	0.88	0.57	1.34	0.539	Radiotherapy & surgery
Infections of other or unspecified organs	Immunology and infection	Uterus	0.68	0.46	1.00	0.053	Radiotherapy & surgery
Infections of the digestive system	Immunology and infection	Uterus	0.66	0.37	1.17	0.159	Radiotherapy & surgery
Peritonitis	Immunology and infection	Uterus	0.78	0.56	1.07	0.118	Radiotherapy & surgery
Osteoporosis	Musculoskeletal	Uterus	1.54	1.09	2.18	0.015	Radiotherapy & surgery
Enthesopathies and synovial disorders	Musculoskeletal	Uterus	1.01	0.78	1.30	0.948	Radiotherapy & surgery
Osteoarthritis (excluding spine)	Musculoskeletal	Uterus	1.03	0.84	1.26	0.755	Radiotherapy & surgery
Peripheral neuropathies	Neurological	Uterus	0.64	0.41	1.01	0.057	Radiotherapy & surgery
Asthma	Pulmonary	Uterus	0.97	0.69	1.36	0.867	Radiotherapy & surgery
COPD	Pulmonary	Uterus	1.20	0.84	1.71	0.315	Radiotherapy & surgery
End stage renal disease	Renal	Uterus	1.19	0.73	1.94	0.476	Radiotherapy & surgery
Urolithiasis	Renal	Uterus	1.01	0.52	1.94	0.975	Radiotherapy & surgery
Atrial fibrillation	Cardiovascular	Uterus	1.10	0.76	1.60	0.614	Radiotherapy alone
Coronary heart disease not otherwise specified	Cardiovascular	Uterus	0.82	0.58	1.15	0.246	Radiotherapy alone
Heart failure	Cardiovascular	Uterus	1.14	0.74	1.74	0.56	Radiotherapy alone
Hypertension	Cardiovascular	Uterus	1.02	0.81	1.27	0.894	Radiotherapy alone
Myocardial infarction	Cardiovascular	Uterus	0.78	0.48	1.27	0.325	Radiotherapy alone
Other cardiomyopathy	Cardiovascular	Uterus	0.91	0.58	1.43	0.678	Radiotherapy alone
Peripheral arterial disease	Cardiovascular	Uterus	1.30	0.67	2.52	0.431	Radiotherapy alone
Stable angina	Cardiovascular	Uterus	1.03	0.66	1.62	0.887	Radiotherapy alone
Stroke (not otherwise specified)	Cardiovascular	Uterus	0.92	0.49	1.72	0.799	Radiotherapy alone
Transient ischaemic attack	Cardiovascular	Uterus	0.96	0.58	1.59	0.862	Radiotherapy alone
Unstable angina	Cardiovascular	Uterus	0.81	0.50	1.30	0.372	Radiotherapy alone
Venous thromboembolic disease	Cardiovascular	Uterus	0.89	0.51	1.55	0.684	Radiotherapy alone
Diabetes	Endocrine	Uterus	0.84	0.63	1.11	0.215	Radiotherapy alone
Diabetic neurological complications	Endocrine	Uterus	0.89	0.65	1.22	0.456	Radiotherapy alone
Diabetic ophthalmic complications	Endocrine	Uterus	0.91	0.68	1.23	0.552	Radiotherapy alone
Hypo or hyperthyroidism	Endocrine	Uterus	0.98	0.64	1.50	0.929	Radiotherapy alone
Fatty liver	Gastrointestinal	Uterus	3.13	1.66	5.89	< 0.001	Radiotherapy alone
Cholangitis	Gastrointestinal	Uterus	0.90	0.50	1.62	0.72	Radiotherapy alone
Cholecystitis	Gastrointestinal	Uterus	0.91	0.52	1.61	0.751	Radiotherapy alone
Cholelithiasis	Gastrointestinal	Uterus	0.92	0.54	1.56	0.754	Radiotherapy alone
Diverticular disease of intestine	Gastrointestinal	Uterus	0.96	0.63	1.45	0.835	Radiotherapy alone
Gastritis and duodenitis	Gastrointestinal	Uterus	0.85	0.51	1.42	0.527	Radiotherapy alone
Gastrooesophageal reflux disease	Gastrointestinal	Uterus	0.88	0.55	1.40	0.594	Radiotherapy alone
Oesophagitis and oesophageal ulcer	Gastrointestinal	Uterus	0.74	0.47	1.17	0.2	Radiotherapy alone
Other anaemias	Haematological	Uterus	1.01	0.68	1.51	0.953	Radiotherapy alone
Infection of skin and subcutaneous tissues	Immunology and infection	Uterus	0.87	0.51	1.49	0.614	Radiotherapy alone
Infections of other or unspecified organs	Immunology and infection	Uterus	0.88	0.56	1.39	0.595	Radiotherapy alone
Infections of the digestive system	Immunology and infection	Uterus	1.10	0.60	2.01	0.758	Radiotherapy alone
Lower respiratory tract infections	Immunology and infection	Uterus	1.44	0.98	2.09	0.06	Radiotherapy alone
Peritonitis	Immunology and infection	Uterus	0.99	0.68	1.44	0.962	Radiotherapy alone
Enthesopathies and synovial disorders	Musculoskeletal	Uterus	0.83	0.57	1.21	0.334	Radiotherapy alone
Osteoarthritis (excluding spine)	Musculoskeletal	Uterus	0.87	0.65	1.15	0.324	Radiotherapy alone
Osteoporosis	Musculoskeletal	Uterus	1.13	0.69	1.84	0.623	Radiotherapy alone
Peripheral neuropathies	Neurological	Uterus	1.14	0.70	1.85	0.591	Radiotherapy alone
Asthma	Pulmonary	Uterus	0.68	0.40	1.16	0.155	Radiotherapy alone
COPD	Pulmonary	Uterus	1.31	0.85	2.04	0.222	Radiotherapy alone
End stage renal disease	Renal	Uterus	1.95	1.18	3.23	0.01	Radiotherapy alone

Table S9. Binomial logistic regression, for each chemotherapy type, for the associations between health conditions and chemotherapy decisions. Patients who were not prescribed with a particular chemotherapy type were used as the reference group (i.e., when considering anthracyclines, patients who did not receive anthracyclines were used as the reference group). Odds ratios were adjusted for cancer type, age, sex, socioeconomic status, tumour grade, tumour stage, tumour count and multimorbidity count. Strata with < 10 patients were not analysed.

Health condition	Organ system	Odds ratio	Lower CI	Upper CI	P value	Chemotherapy type
Abdominal aortic aneurysm	Cardiovascular	1.39	1.14	1.68	0.001	Alkylating agents
Atrial fibrillation	Cardiovascular	0.59	0.51	0.67	< 0.001	Alkylating agents
Atrioventricular block, complete	Cardiovascular	0.94	0.73	1.19	0.617	Alkylating agents
Atrioventricular block, first degree	Cardiovascular	0.94	0.73	1.19	0.617	Alkylating agents
Atrioventricular block, second degree	Cardiovascular	0.94	0.73	1.19	0.617	Alkylating agents
Bifascicular block	Cardiovascular	1.08	0.80	1.42	0.605	Alkylating agents
Coronary heart disease not otherwise specified	Cardiovascular	0.76	0.70	0.83	< 0.001	Alkylating agents
Heart failure	Cardiovascular	0.60	0.52	0.69	< 0.001	Alkylating agents
Hypertension	Cardiovascular	0.78	0.73	0.84	< 0.001	Alkylating agents
Ischaemic stroke	Cardiovascular	0.49	0.38	0.64	< 0.001	Alkylating agents
Left bundle branch block	Cardiovascular	0.92	0.72	1.16	0.484	Alkylating agents
Multiple valve disease	Cardiovascular	0.74	0.54	0.99	0.05	Alkylating agents
Myocardial infarction	Cardiovascular	0.67	0.58	0.76	< 0.001	Alkylating agents
Nonrheumatic aortic valve disorders	Cardiovascular	0.85	0.65	1.09	0.206	Alkylating agents
Nonrheumatic mitral valve disorders	Cardiovascular	0.91	0.68	1.19	0.49	Alkylating agents
Other cardiomyopathy	Cardiovascular	0.65	0.57	0.74	< 0.001	Alkylating agents
Pericardial effusion (noninflammatory)	Cardiovascular	0.74	0.49	1.08	0.131	Alkylating agents
Peripheral arterial disease	Cardiovascular	0.55	0.43	0.68	< 0.001	Alkylating agents
Primary pulmonary hypertension	Cardiovascular	0.47	0.26	0.79	0.007	Alkylating agents
Raynaud's syndrome	Cardiovascular	0.64	0.50	0.80	< 0.001	Alkylating agents
Right bundle branch block	Cardiovascular	1.04	0.77	1.38	0.794	Alkylating agents
Secondary pulmonary hypertension	Cardiovascular	0.45	0.24	0.77	0.007	Alkylating agents
Sick sinus syndrome	Cardiovascular	0.59	0.38	0.87	0.013	Alkylating agents
Stable angina	Cardiovascular	0.65	0.56	0.75	< 0.001	Alkylating agents
Stroke (not otherwise specified)	Cardiovascular	0.51	0.41	0.63	< 0.001	Alkylating agents
Supraventricular tachycardia	Cardiovascular	0.81	0.59	1.08	0.171	Alkylating agents
Transient ischaemic attack	Cardiovascular	0.78	0.67	0.91	0.002	Alkylating agents
Trifascicular block	Cardiovascular	0.94	0.68	1.28	0.713	Alkylating agents
Unstable angina	Cardiovascular	0.64	0.55	0.74	< 0.001	Alkylating agents
Venous thromboembolic disease	Cardiovascular	0.63	0.54	0.74	< 0.001	Alkylating agents
Ventricular tachycardia	Cardiovascular	0.81	0.59	1.08	0.169	Alkylating agents
Diabetes	Endocrine	0.91	0.84	1.00	0.04	Alkylating agents
Diabetic neurological complications	Endocrine	0.76	0.68	0.85	< 0.001	Alkylating agents
Diabetic ophthalmic complications	Endocrine	0.79	0.71	0.87	< 0.001	Alkylating agents
Hyperparathyroidism	Endocrine	0.88	0.56	1.33	0.561	Alkylating agents
Hypo or hyperthyroidism	Endocrine	0.87	0.76	0.99	0.034	Alkylating agents
Alcoholic liver disease	Gastrointestinal	0.92	0.63	1.29	0.623	Alkylating agents
Angiodysplasia of colon	Gastrointestinal	0.92	0.52	1.51	0.744	Alkylating agents
Barrett's oesophagus	Gastrointestinal	0.90	0.73	1.09	0.289	Alkylating agents
Cholangitis	Gastrointestinal	0.79	0.67	0.91	0.002	Alkylating agents
Cholecystitis	Gastrointestinal	0.83	0.71	0.96	0.017	Alkylating agents
Cholelithiasis	Gastrointestinal	0.81	0.70	0.93	0.004	Alkylating agents
Crohn's disease	Gastrointestinal	0.72	0.40	1.20	0.235	Alkylating agents
Diverticular disease of intestine	Gastrointestinal	1.02	0.91	1.13	0.768	Alkylating agents
Fatty liver	Gastrointestinal	1.18	0.98	1.41	0.074	Alkylating agents
Gastritis and duodenitis	Gastrointestinal	0.92	0.83	1.03	0.148	Alkylating agents
Gastrooesophageal reflux disease	Gastrointestinal	1.07	0.96	1.18	0.221	Alkylating agents
Hepatic failure	Gastrointestinal	1.12	0.92	1.36	0.262	Alkylating agents
Liver fibrosis sclerosis and cirrhosis	Gastrointestinal	0.63	0.39	0.95	0.038	Alkylating agents
Oesophagitis and oesophageal ulcer	Gastrointestinal	0.93	0.84	1.03	0.164	Alkylating agents
Portal hypertension	Gastrointestinal	1.18	0.94	1.47	0.136	Alkylating agents
Ulcerative colitis	Gastrointestinal	0.61	0.36	0.96	0.046	Alkylating agents
Agranulocytosis	Haematological	1.03	0.85	1.24	0.762	Alkylating agents
Aplastic anaemias	Haematological	0.69	0.51	0.91	0.012	Alkylating agents
Other anaemias	Haematological	0.88	0.80	0.97	0.009	Alkylating agents
Other haemolytic anaemias	Haematological	0.92	0.59	1.39	0.714	Alkylating agents
Primary or idiopathic thrombocytopaenia	Haematological	0.73	0.55	0.96	0.026	Alkylating agents
Secondary or other thrombocytopaenia	Haematological	0.82	0.65	1.02	0.077	Alkylating agents
Secondary polycythaemia	Haematological	0.56	0.32	0.91	0.027	Alkylating agents
Thrombophilia	Haematological	1.09	0.71	1.62	0.691	Alkylating agents
Autoimmune liver disease	Immunology and infection	0.70	0.42	1.10	0.142	Alkylating agents
Chronic viral hepatitis	Immunology and infection	1.03	0.80	1.31	0.834	Alkylating agents
Encephalitis	Immunology and infection	1.20	0.80	1.76	0.357	Alkylating agents
HIV	Immunology and infection	0.85	0.61	1.17	0.325	Alkylating agents
Immunodeficiencies	Immunology and infection	0.95	0.55	1.55	0.854	Alkylating agents
Infection of bones and joints	Immunology and infection	0.80	0.63	1.02	0.074	Alkylating agents
Infection of liver	Immunology and infection	0.75	0.46	1.17	0.229	Alkylating agents
Infection of skin and subcutaneous tissues	Immunology and infection	0.74	0.64	0.85	< 0.001	Alkylating agents
Infections of other or unspecified organs	Immunology and infection	0.80	0.72	0.89	< 0.001	Alkylating agents
Infections of the digestive system	Immunology and infection	1.16	1.02	1.30	0.02	Alkylating agents
Infections of the heart	Immunology and infection	0.72	0.54	0.93	0.016	Alkylating agents
Lower respiratory tract infections	Immunology and infection	0.75	0.68	0.82	< 0.001	Alkylating agents
Lupus erythematosus (local and systemic)	Immunology and infection	1.10	0.59	1.93	0.748	Alkylating agents
Meningitis	Immunology and infection	0.90	0.70	1.13	0.367	Alkylating agents
Non-acute cystitis	Immunology and infection	0.95	0.66	1.34	0.793	Alkylating agents
Peritonitis	Immunology and infection	1.06	0.97	1.17	0.192	Alkylating agents
Viral diseases (excluding chronic hepatitis/HIV)	Immunology and infection	1.04	0.87	1.25	0.638	Alkylating agents
Ankylosing spondylitis	Musculoskeletal	1.63	0.82	3.03	0.142	Alkylating agents
Enteropathic arthropathy	Musculoskeletal	0.66	0.37	1.11	0.141	Alkylating agents
Enthesopathies and synovial disorders	Musculoskeletal	1.02	0.94	1.11	0.607	Alkylating agents
Osteoarthritis (excluding spine)	Musculoskeletal	0.99	0.91	1.07	0.731	Alkylating agents
Osteoporosis	Musculoskeletal	0.86	0.75	0.99	0.037	Alkylating agents
Rheumatoid arthritis	Musculoskeletal	0.90	0.72	1.13	0.387	Alkylating agents
Scoliosis	Musculoskeletal	0.62	0.38	0.97	0.047	Alkylating agents
Spinal stenosis	Musculoskeletal	1.55	1.27	1.88	< 0.001	Alkylating agents
Bell's palsy	Neurological	0.73	0.50	1.05	0.099	Alkylating agents
Dementia	Neurological	0.42	0.31	0.55	< 0.001	Alkylating agents
Disorders of autonomic nervous system	Neurological	1.21	0.95	1.52	0.112	Alkylating agents
Epilepsy	Neurological	0.80	0.65	0.99	0.04	Alkylating agents
Essential tremor	Neurological	1.54	1.01	2.27	0.035	Alkylating agents
Intracerebral haemorrhage	Neurological	0.49	0.34	0.68	< 0.001	Alkylating agents
Intracranial hypertension	Neurological	1.30	1.08	1.56	0.004	Alkylating agents
Peripheral neuropathies	Neurological	0.94	0.82	1.08	0.407	Alkylating agents
Subarachnoid haemorrhage	Neurological	0.33	0.20	0.51	< 0.001	Alkylating agents
Asthma	Pulmonary	0.77	0.68	0.88	< 0.001	Alkylating agents
Bronchiectasis	Pulmonary	0.82	0.57	1.13	0.246	Alkylating agents
COPD	Pulmonary	0.89	0.79	0.99	0.04	Alkylating agents
Other interstitial pulmonary diseases with fibrosis	Pulmonary	0.62	0.37	0.97	0.049	Alkylating agents
End stage renal disease	Renal	0.80	0.70	0.92	0.002	Alkylating agents
Glomerulonephritis	Renal	0.74	0.53	1.00	0.055	Alkylating agents
Obstructive and reflux uropathy	Renal	1.05	0.83	1.31	0.688	Alkylating agents
Urolithiasis	Renal	1.02	0.85	1.21	0.835	Alkylating agents
Abdominal aortic aneurysm	Cardiovascular	1.43	1.19	1.72	< 0.001	Anthracyclines
Atrial fibrillation	Cardiovascular	0.50	0.42	0.58	< 0.001	Anthracyclines
Atrioventricular block, complete	Cardiovascular	0.52	0.36	0.72	< 0.001	Anthracyclines
Atrioventricular block, first degree	Cardiovascular	0.52	0.36	0.72	< 0.001	Anthracyclines
Atrioventricular block, second degree	Cardiovascular	0.52	0.36	0.72	< 0.001	Anthracyclines
Bifascicular block	Cardiovascular	0.63	0.41	0.92	0.022	Anthracyclines
Coronary heart disease not otherwise specified	Cardiovascular	0.72	0.65	0.79	< 0.001	Anthracyclines
Heart failure	Cardiovascular	0.48	0.40	0.57	< 0.001	Anthracyclines
Hypertension	Cardiovascular	0.75	0.70	0.81	< 0.001	Anthracyclines
Ischaemic stroke	Cardiovascular	0.40	0.28	0.56	< 0.001	Anthracyclines
Left bundle branch block	Cardiovascular	0.48	0.34	0.66	< 0.001	Anthracyclines
Multiple valve disease	Cardiovascular	0.55	0.37	0.79	0.002	Anthracyclines
Myocardial infarction	Cardiovascular	0.56	0.49	0.65	< 0.001	Anthracyclines
Nonrheumatic aortic valve disorders	Cardiovascular	0.57	0.40	0.78	0.001	Anthracyclines
Nonrheumatic mitral valve disorders	Cardiovascular	0.61	0.41	0.86	0.007	Anthracyclines
Other cardiomyopathy	Cardiovascular	0.56	0.48	0.64	< 0.001	Anthracyclines
Pericardial effusion (noninflammatory)	Cardiovascular	0.75	0.47	1.13	0.19	Anthracyclines
Peripheral arterial disease	Cardiovascular	0.44	0.33	0.56	< 0.001	Anthracyclines
Raynaud's syndrome	Cardiovascular	0.56	0.43	0.72	< 0.001	Anthracyclines
Right bundle branch block	Cardiovascular	0.63	0.42	0.92	0.024	Anthracyclines
Sick sinus syndrome	Cardiovascular	0.46	0.26	0.74	0.003	Anthracyclines
Stable angina	Cardiovascular	0.58	0.49	0.68	< 0.001	Anthracyclines
Stroke (not otherwise specified)	Cardiovascular	0.46	0.35	0.60	< 0.001	Anthracyclines
Supraventricular tachycardia	Cardiovascular	0.66	0.45	0.92	0.021	Anthracyclines
Transient ischaemic attack	Cardiovascular	0.66	0.55	0.79	< 0.001	Anthracyclines
Trifascicular block	Cardiovascular	0.63	0.40	0.94	0.031	Anthracyclines
Unstable angina	Cardiovascular	0.55	0.46	0.65	< 0.001	Anthracyclines
Venous thromboembolic disease	Cardiovascular	0.56	0.47	0.67	< 0.001	Anthracyclines
Ventricular tachycardia	Cardiovascular	0.68	0.47	0.95	0.031	Anthracyclines
Diabetes	Endocrine	0.88	0.81	0.97	0.007	Anthracyclines
Diabetic neurological complications	Endocrine	0.80	0.71	0.89	< 0.001	Anthracyclines
Diabetic ophthalmic complications	Endocrine	0.80	0.72	0.90	< 0.001	Anthracyclines
Hyperparathyroidism	Endocrine	0.87	0.52	1.38	0.572	Anthracyclines
Hypo or hyperthyroidism	Endocrine	0.78	0.68	0.90	0.001	Anthracyclines
Alcoholic liver disease	Gastrointestinal	0.76	0.53	1.07	0.128	Anthracyclines
Barrett's oesophagus	Gastrointestinal	0.83	0.71	0.97	0.023	Anthracyclines
Cholangitis	Gastrointestinal	0.81	0.69	0.95	0.009	Anthracyclines
Cholecystitis	Gastrointestinal	0.81	0.69	0.95	0.01	Anthracyclines
Cholelithiasis	Gastrointestinal	0.78	0.67	0.90	0.001	Anthracyclines
Coeliac disease	Gastrointestinal	1.13	0.84	1.48	0.412	Anthracyclines
Crohn's disease	Gastrointestinal	1.04	0.62	1.66	0.873	Anthracyclines
Diverticular disease of intestine	Gastrointestinal	1.07	0.95	1.20	0.252	Anthracyclines
Fatty liver	Gastrointestinal	0.94	0.77	1.13	0.523	Anthracyclines
Gastritis and duodenitis	Gastrointestinal	0.91	0.81	1.01	0.088	Anthracyclines
Gastrooesophageal reflux disease	Gastrointestinal	1.03	0.93	1.14	0.601	Anthracyclines
Hepatic failure	Gastrointestinal	0.82	0.66	1.01	0.066	Anthracyclines
Liver fibrosis sclerosis and cirrhosis	Gastrointestinal	0.64	0.42	0.93	0.026	Anthracyclines
Oesophageal varices	Gastrointestinal	0.83	0.44	1.44	0.535	Anthracyclines
Oesophagitis and oesophageal ulcer	Gastrointestinal	0.86	0.78	0.95	0.002	Anthracyclines
Portal hypertension	Gastrointestinal	0.90	0.70	1.13	0.371	Anthracyclines
Ulcerative colitis	Gastrointestinal	0.59	0.33	0.96	0.046	Anthracyclines
Agranulocytosis	Haematological	1.87	1.56	2.24	< 0.001	Anthracyclines
Aplastic anaemias	Haematological	1.30	0.98	1.70	0.057	Anthracyclines
Other anaemias	Haematological	0.76	0.68	0.84	< 0.001	Anthracyclines
Primary or idiopathic thrombocytopaenia	Haematological	0.78	0.56	1.05	0.117	Anthracyclines
Secondary or other thrombocytopaenia	Haematological	0.76	0.58	0.99	0.048	Anthracyclines
Thrombophilia	Haematological	1.13	0.72	1.72	0.578	Anthracyclines
Autoimmune liver disease	Immunology and infection	0.70	0.42	1.11	0.155	Anthracyclines
Chronic viral hepatitis	Immunology and infection	1.15	0.89	1.45	0.275	Anthracyclines
Encephalitis	Immunology and infection	1.20	0.72	1.90	0.45	Anthracyclines
HIV	Immunology and infection	0.81	0.57	1.14	0.244	Anthracyclines
Infection of anal and rectal regions	Immunology and infection	0.98	0.49	1.77	0.95	Anthracyclines
Infection of bones and joints	Immunology and infection	0.68	0.49	0.92	0.017	Anthracyclines
Infection of liver	Immunology and infection	0.65	0.37	1.07	0.113	Anthracyclines
Infection of skin and subcutaneous tissues	Immunology and infection	0.73	0.62	0.84	< 0.001	Anthracyclines
Infections of other or unspecified organs	Immunology and infection	0.84	0.75	0.94	0.002	Anthracyclines
Infections of the digestive system	Immunology and infection	1.17	1.03	1.32	0.014	Anthracyclines
Infections of the heart	Immunology and infection	0.78	0.59	1.02	0.078	Anthracyclines
Lower respiratory tract infections	Immunology and infection	0.68	0.61	0.75	< 0.001	Anthracyclines
Meningitis	Immunology and infection	0.90	0.69	1.15	0.402	Anthracyclines
Non-acute cystitis	Immunology and infection	1.11	0.76	1.56	0.576	Anthracyclines
Peritonitis	Immunology and infection	1.02	0.93	1.13	0.612	Anthracyclines
Viral diseases (excluding chronic hepatitis/HIV)	Immunology and infection	1.00	0.81	1.23	0.969	Anthracyclines
Enthesopathies and synovial disorders	Musculoskeletal	0.99	0.91	1.08	0.812	Anthracyclines
Osteoarthritis (excluding spine)	Musculoskeletal	0.95	0.87	1.03	0.227	Anthracyclines
Osteoporosis	Musculoskeletal	0.79	0.68	0.93	0.005	Anthracyclines
Rheumatoid arthritis	Musculoskeletal	0.83	0.64	1.06	0.157	Anthracyclines
Scoliosis	Musculoskeletal	0.42	0.21	0.73	0.005	Anthracyclines
Spinal stenosis	Musculoskeletal	1.17	0.90	1.49	0.22	Anthracyclines
Bell's palsy	Neurological	0.92	0.60	1.37	0.707	Anthracyclines
Dementia	Neurological	0.27	0.18	0.39	< 0.001	Anthracyclines
Disorders of autonomic nervous system	Neurological	1.13	0.84	1.51	0.401	Anthracyclines
Epilepsy	Neurological	0.83	0.57	1.17	0.316	Anthracyclines
Essential tremor	Neurological	1.43	0.89	2.19	0.122	Anthracyclines
Intracerebral haemorrhage	Neurological	0.34	0.19	0.56	< 0.001	Anthracyclines
Intracranial hypertension	Neurological	0.65	0.39	1.03	0.085	Anthracyclines
Peripheral neuropathies	Neurological	0.83	0.71	0.97	0.019	Anthracyclines
Subarachnoid haemorrhage	Neurological	0.33	0.18	0.54	< 0.001	Anthracyclines
Asthma	Pulmonary	0.72	0.62	0.82	< 0.001	Anthracyclines
Bronchiectasis	Pulmonary	0.72	0.48	1.04	0.097	Anthracyclines
COPD	Pulmonary	0.83	0.74	0.94	0.003	Anthracyclines
Other interstitial pulmonary diseases with fibrosis	Pulmonary	0.63	0.37	1.00	0.068	Anthracyclines
End stage renal disease	Renal	0.79	0.67	0.92	0.003	Anthracyclines
Glomerulonephritis	Renal	0.55	0.36	0.82	0.005	Anthracyclines
Obstructive and reflux uropathy	Renal	1.07	0.84	1.33	0.587	Anthracyclines
Urolithiasis	Renal	1.02	0.85	1.22	0.823	Anthracyclines
Abdominal aortic aneurysm	Cardiovascular	1.39	1.26	1.54	< 0.001	Antimetabolites
Atrial fibrillation	Cardiovascular	0.59	0.54	0.65	< 0.001	Antimetabolites
Atrioventricular block, complete	Cardiovascular	0.65	0.54	0.78	< 0.001	Antimetabolites
Atrioventricular block, first degree	Cardiovascular	0.65	0.54	0.78	< 0.001	Antimetabolites
Atrioventricular block, second degree	Cardiovascular	0.65	0.54	0.78	< 0.001	Antimetabolites
Bifascicular block	Cardiovascular	0.67	0.54	0.83	< 0.001	Antimetabolites
Coronary heart disease not otherwise specified	Cardiovascular	0.75	0.70	0.80	< 0.001	Antimetabolites
Dilated cardiomyopathy	Cardiovascular	0.56	0.36	0.84	0.007	Antimetabolites
Heart failure	Cardiovascular	0.63	0.57	0.69	< 0.001	Antimetabolites
Hypertension	Cardiovascular	0.91	0.87	0.95	< 0.001	Antimetabolites
Hypertrophic cardiomyopathy	Cardiovascular	0.52	0.33	0.78	0.003	Antimetabolites
Ischaemic stroke	Cardiovascular	0.42	0.35	0.51	< 0.001	Antimetabolites
Left bundle branch block	Cardiovascular	0.67	0.56	0.80	< 0.001	Antimetabolites
Multiple valve disease	Cardiovascular	0.72	0.57	0.89	0.003	Antimetabolites
Myocardial infarction	Cardiovascular	0.65	0.60	0.71	< 0.001	Antimetabolites
Nonrheumatic aortic valve disorders	Cardiovascular	0.63	0.51	0.76	< 0.001	Antimetabolites
Nonrheumatic mitral valve disorders	Cardiovascular	0.64	0.50	0.80	< 0.001	Antimetabolites
Other cardiomyopathy	Cardiovascular	0.65	0.60	0.70	< 0.001	Antimetabolites
Pericardial effusion (noninflammatory)	Cardiovascular	0.82	0.61	1.08	0.178	Antimetabolites
Peripheral arterial disease	Cardiovascular	0.64	0.55	0.72	< 0.001	Antimetabolites
Primary pulmonary hypertension	Cardiovascular	0.41	0.27	0.61	< 0.001	Antimetabolites
Raynaud's syndrome	Cardiovascular	0.68	0.58	0.78	< 0.001	Antimetabolites
Right bundle branch block	Cardiovascular	0.67	0.54	0.83	< 0.001	Antimetabolites
Secondary pulmonary hypertension	Cardiovascular	0.41	0.26	0.61	< 0.001	Antimetabolites
Sick sinus syndrome	Cardiovascular	0.87	0.67	1.10	0.264	Antimetabolites
Stable angina	Cardiovascular	0.68	0.61	0.74	< 0.001	Antimetabolites
Stroke (not otherwise specified)	Cardiovascular	0.56	0.48	0.64	< 0.001	Antimetabolites
Supraventricular tachycardia	Cardiovascular	0.67	0.53	0.83	0.001	Antimetabolites
Transient ischaemic attack	Cardiovascular	0.78	0.71	0.87	< 0.001	Antimetabolites
Trifascicular block	Cardiovascular	0.62	0.48	0.78	< 0.001	Antimetabolites
Unstable angina	Cardiovascular	0.66	0.60	0.73	< 0.001	Antimetabolites
Venous thromboembolic disease	Cardiovascular	0.51	0.46	0.57	< 0.001	Antimetabolites
Ventricular tachycardia	Cardiovascular	0.68	0.54	0.85	0.001	Antimetabolites
Diabetes	Endocrine	0.96	0.90	1.02	0.149	Antimetabolites
Diabetic neurological complications	Endocrine	0.91	0.85	0.98	0.008	Antimetabolites
Diabetic ophthalmic complications	Endocrine	0.92	0.86	0.98	0.011	Antimetabolites
Hyperparathyroidism	Endocrine	0.82	0.56	1.16	0.291	Antimetabolites
Hypo or hyperthyroidism	Endocrine	0.78	0.70	0.86	< 0.001	Antimetabolites
Alcoholic liver disease	Gastrointestinal	0.53	0.41	0.67	< 0.001	Antimetabolites
Angiodysplasia of colon	Gastrointestinal	0.60	0.42	0.83	0.003	Antimetabolites
Barrett's oesophagus	Gastrointestinal	0.88	0.80	0.98	0.021	Antimetabolites
Cholangitis	Gastrointestinal	1.06	0.98	1.15	0.142	Antimetabolites
Cholecystitis	Gastrointestinal	0.87	0.79	0.95	0.004	Antimetabolites
Cholelithiasis	Gastrointestinal	0.86	0.79	0.95	0.002	Antimetabolites
Coeliac disease	Gastrointestinal	1.13	0.93	1.36	0.209	Antimetabolites
Crohn's disease	Gastrointestinal	0.71	0.50	0.97	0.038	Antimetabolites
Diverticular disease of intestine	Gastrointestinal	1.08	1.01	1.16	0.016	Antimetabolites
Fatty liver	Gastrointestinal	0.89	0.79	0.99	0.034	Antimetabolites
Gastritis and duodenitis	Gastrointestinal	0.86	0.81	0.93	< 0.001	Antimetabolites
Gastrooesophageal reflux disease	Gastrointestinal	1.06	0.99	1.13	0.112	Antimetabolites
Hepatic failure	Gastrointestinal	0.81	0.72	0.91	< 0.001	Antimetabolites
Liver fibrosis sclerosis and cirrhosis	Gastrointestinal	0.40	0.31	0.51	< 0.001	Antimetabolites
Oesophageal varices	Gastrointestinal	0.35	0.22	0.52	< 0.001	Antimetabolites
Oesophagitis and oesophageal ulcer	Gastrointestinal	0.94	0.88	1.00	0.04	Antimetabolites
Portal hypertension	Gastrointestinal	0.88	0.78	1.00	0.058	Antimetabolites
Ulcerative colitis	Gastrointestinal	0.82	0.60	1.09	0.192	Antimetabolites
Agranulocytosis	Haematological	1.59	1.35	1.87	< 0.001	Antimetabolites
Aplastic anaemias	Haematological	1.71	1.37	2.10	< 0.001	Antimetabolites
Other anaemias	Haematological	0.71	0.67	0.76	< 0.001	Antimetabolites
Other haemolytic anaemias	Haematological	0.82	0.46	1.36	0.479	Antimetabolites
Primary or idiopathic thrombocytopaenia	Haematological	0.88	0.69	1.10	0.26	Antimetabolites
Secondary or other thrombocytopaenia	Haematological	0.90	0.74	1.08	0.256	Antimetabolites
Secondary polycythaemia	Haematological	0.90	0.62	1.25	0.538	Antimetabolites
Thrombophilia	Haematological	0.92	0.65	1.27	0.63	Antimetabolites
Autoimmune liver disease	Immunology and infection	0.57	0.43	0.75	< 0.001	Antimetabolites
Chronic viral hepatitis	Immunology and infection	1.15	0.98	1.35	0.079	Antimetabolites
Encephalitis	Immunology and infection	0.68	0.42	1.03	0.086	Antimetabolites
HIV	Immunology and infection	1.00	0.74	1.33	1	Antimetabolites
Infection of anal and rectal regions	Immunology and infection	0.76	0.50	1.12	0.187	Antimetabolites
Infection of bones and joints	Immunology and infection	0.67	0.53	0.83	< 0.001	Antimetabolites
Infection of liver	Immunology and infection	0.65	0.48	0.87	0.005	Antimetabolites
Infection of skin and subcutaneous tissues	Immunology and infection	0.73	0.65	0.80	< 0.001	Antimetabolites
Infections of other or unspecified organs	Immunology and infection	0.70	0.65	0.75	< 0.001	Antimetabolites
Infections of the digestive system	Immunology and infection	1.14	1.06	1.22	< 0.001	Antimetabolites
Infections of the heart	Immunology and infection	0.56	0.46	0.68	< 0.001	Antimetabolites
Lower respiratory tract infections	Immunology and infection	0.69	0.65	0.73	< 0.001	Antimetabolites
Lupus erythematosus (local and systemic)	Immunology and infection	0.82	0.47	1.34	0.452	Antimetabolites
Meningitis	Immunology and infection	0.65	0.54	0.78	< 0.001	Antimetabolites
Non-acute cystitis	Immunology and infection	0.75	0.58	0.95	0.023	Antimetabolites
Peritonitis	Immunology and infection	1.03	0.97	1.09	0.295	Antimetabolites
Viral diseases (excluding chronic hepatitis/HIV)	Immunology and infection	0.83	0.70	0.97	0.019	Antimetabolites
Ankylosing spondylitis	Musculoskeletal	0.68	0.33	1.26	0.248	Antimetabolites
Enteropathic arthropathy	Musculoskeletal	0.69	0.45	1.02	0.078	Antimetabolites
Enthesopathies and synovial disorders	Musculoskeletal	0.99	0.93	1.05	0.742	Antimetabolites
Osteoarthritis (excluding spine)	Musculoskeletal	1.00	0.94	1.06	0.878	Antimetabolites
Osteoporosis	Musculoskeletal	0.66	0.59	0.74	< 0.001	Antimetabolites
Rheumatoid arthritis	Musculoskeletal	0.66	0.54	0.80	< 0.001	Antimetabolites
Scoliosis	Musculoskeletal	0.55	0.37	0.79	0.002	Antimetabolites
Spinal stenosis	Musculoskeletal	1.06	0.89	1.26	0.481	Antimetabolites
Bell's palsy	Neurological	0.66	0.45	0.94	0.025	Antimetabolites
Dementia	Neurological	0.19	0.14	0.25	< 0.001	Antimetabolites
Disorders of autonomic nervous system	Neurological	0.90	0.71	1.13	0.393	Antimetabolites
Epilepsy	Neurological	0.68	0.53	0.86	0.002	Antimetabolites
Essential tremor	Neurological	1.16	0.85	1.56	0.339	Antimetabolites
Intracerebral haemorrhage	Neurological	0.31	0.22	0.42	< 0.001	Antimetabolites
Intracranial hypertension	Neurological	0.73	0.53	0.97	0.04	Antimetabolites
Multiple sclerosis	Neurological	0.81	0.43	1.40	0.478	Antimetabolites
Peripheral neuropathies	Neurological	0.83	0.74	0.92	0.001	Antimetabolites
Subarachnoid haemorrhage	Neurological	0.31	0.21	0.43	< 0.001	Antimetabolites
Asthma	Pulmonary	0.72	0.65	0.79	< 0.001	Antimetabolites
Bronchiectasis	Pulmonary	0.77	0.61	0.97	0.028	Antimetabolites
COPD	Pulmonary	0.84	0.79	0.91	< 0.001	Antimetabolites
Other interstitial pulmonary diseases with fibrosis	Pulmonary	0.51	0.37	0.67	< 0.001	Antimetabolites
End stage renal disease	Renal	0.62	0.55	0.68	< 0.001	Antimetabolites
Glomerulonephritis	Renal	0.56	0.43	0.71	< 0.001	Antimetabolites
Obstructive and reflux uropathy	Renal	0.84	0.72	0.98	0.033	Antimetabolites
Urolithiasis	Renal	0.92	0.81	1.03	0.155	Antimetabolites
Abdominal aortic aneurysm	Cardiovascular	1.36	1.16	1.58	< 0.001	Biological response modifiers including immunotherapy
Atrial fibrillation	Cardiovascular	0.69	0.62	0.77	< 0.001	Biological response modifiers including immunotherapy
Atrioventricular block, complete	Cardiovascular	0.77	0.62	0.96	0.024	Biological response modifiers including immunotherapy
Atrioventricular block, first degree	Cardiovascular	0.77	0.62	0.96	0.024	Biological response modifiers including immunotherapy
Atrioventricular block, second degree	Cardiovascular	0.77	0.62	0.96	0.024	Biological response modifiers including immunotherapy
Bifascicular block	Cardiovascular	0.83	0.63	1.08	0.177	Biological response modifiers including immunotherapy
Coronary heart disease not otherwise specified	Cardiovascular	0.74	0.68	0.80	< 0.001	Biological response modifiers including immunotherapy
Dilated cardiomyopathy	Cardiovascular	0.68	0.40	1.09	0.132	Biological response modifiers including immunotherapy
Heart failure	Cardiovascular	0.64	0.57	0.72	< 0.001	Biological response modifiers including immunotherapy
Hypertension	Cardiovascular	0.89	0.83	0.94	< 0.001	Biological response modifiers including immunotherapy
Hypertrophic cardiomyopathy	Cardiovascular	0.67	0.40	1.07	0.119	Biological response modifiers including immunotherapy
Ischaemic stroke	Cardiovascular	0.52	0.41	0.66	< 0.001	Biological response modifiers including immunotherapy
Left bundle branch block	Cardiovascular	0.80	0.64	0.98	0.036	Biological response modifiers including immunotherapy
Multiple valve disease	Cardiovascular	0.86	0.67	1.09	0.231	Biological response modifiers including immunotherapy
Myocardial infarction	Cardiovascular	0.70	0.63	0.78	< 0.001	Biological response modifiers including immunotherapy
Nonrheumatic aortic valve disorders	Cardiovascular	0.85	0.67	1.05	0.142	Biological response modifiers including immunotherapy
Nonrheumatic mitral valve disorders	Cardiovascular	0.88	0.68	1.12	0.32	Biological response modifiers including immunotherapy
Other cardiomyopathy	Cardiovascular	0.71	0.64	0.79	< 0.001	Biological response modifiers including immunotherapy
Pericardial effusion (noninflammatory)	Cardiovascular	0.75	0.52	1.06	0.121	Biological response modifiers including immunotherapy
Peripheral arterial disease	Cardiovascular	0.73	0.61	0.86	< 0.001	Biological response modifiers including immunotherapy
Primary pulmonary hypertension	Cardiovascular	0.50	0.31	0.77	0.003	Biological response modifiers including immunotherapy
Raynaud's syndrome	Cardiovascular	0.68	0.55	0.83	< 0.001	Biological response modifiers including immunotherapy
Right bundle branch block	Cardiovascular	0.81	0.61	1.05	0.123	Biological response modifiers including immunotherapy
Secondary pulmonary hypertension	Cardiovascular	0.49	0.29	0.76	0.003	Biological response modifiers including immunotherapy
Sick sinus syndrome	Cardiovascular	0.78	0.55	1.07	0.144	Biological response modifiers including immunotherapy
Stable angina	Cardiovascular	0.68	0.60	0.77	< 0.001	Biological response modifiers including immunotherapy
Stroke (not otherwise specified)	Cardiovascular	0.55	0.44	0.66	< 0.001	Biological response modifiers including immunotherapy
Supraventricular tachycardia	Cardiovascular	0.71	0.52	0.94	0.023	Biological response modifiers including immunotherapy
Transient ischaemic attack	Cardiovascular	0.79	0.69	0.91	0.001	Biological response modifiers including immunotherapy
Trifascicular block	Cardiovascular	0.76	0.56	1.01	0.065	Biological response modifiers including immunotherapy
Unstable angina	Cardiovascular	0.68	0.60	0.77	< 0.001	Biological response modifiers including immunotherapy
Venous thromboembolic disease	Cardiovascular	0.70	0.61	0.79	< 0.001	Biological response modifiers including immunotherapy
Ventricular tachycardia	Cardiovascular	0.65	0.47	0.87	0.006	Biological response modifiers including immunotherapy
Diabetes	Endocrine	0.91	0.84	0.98	0.02	Biological response modifiers including immunotherapy
Diabetic neurological complications	Endocrine	0.87	0.79	0.96	0.004	Biological response modifiers including immunotherapy
Diabetic ophthalmic complications	Endocrine	0.88	0.81	0.96	0.006	Biological response modifiers including immunotherapy
Hyperparathyroidism	Endocrine	0.87	0.58	1.27	0.494	Biological response modifiers including immunotherapy
Hypo or hyperthyroidism	Endocrine	0.87	0.77	0.99	0.031	Biological response modifiers including immunotherapy
Alcoholic liver disease	Gastrointestinal	0.77	0.54	1.05	0.117	Biological response modifiers including immunotherapy
Angiodysplasia of colon	Gastrointestinal	0.97	0.61	1.45	0.873	Biological response modifiers including immunotherapy
Barrett's oesophagus	Gastrointestinal	0.82	0.69	0.97	0.021	Biological response modifiers including immunotherapy
Cholangitis	Gastrointestinal	0.85	0.74	0.97	0.022	Biological response modifiers including immunotherapy
Cholecystitis	Gastrointestinal	0.83	0.72	0.96	0.011	Biological response modifiers including immunotherapy
Cholelithiasis	Gastrointestinal	0.81	0.71	0.93	0.003	Biological response modifiers including immunotherapy
Coeliac disease	Gastrointestinal	1.18	0.91	1.52	0.194	Biological response modifiers including immunotherapy
Crohn's disease	Gastrointestinal	1.16	0.76	1.71	0.461	Biological response modifiers including immunotherapy
Diverticular disease of intestine	Gastrointestinal	1.04	0.94	1.14	0.467	Biological response modifiers including immunotherapy
Fatty liver	Gastrointestinal	0.99	0.83	1.16	0.883	Biological response modifiers including immunotherapy
Gastritis and duodenitis	Gastrointestinal	0.91	0.83	1.01	0.069	Biological response modifiers including immunotherapy
Gastrooesophageal reflux disease	Gastrointestinal	1.05	0.95	1.16	0.32	Biological response modifiers including immunotherapy
Hepatic failure	Gastrointestinal	0.98	0.83	1.16	0.853	Biological response modifiers including immunotherapy
Liver fibrosis sclerosis and cirrhosis	Gastrointestinal	0.75	0.51	1.05	0.107	Biological response modifiers including immunotherapy
Oesophageal varices	Gastrointestinal	0.84	0.45	1.45	0.561	Biological response modifiers including immunotherapy
Oesophagitis and oesophageal ulcer	Gastrointestinal	0.89	0.81	0.97	0.012	Biological response modifiers including immunotherapy
Portal hypertension	Gastrointestinal	1.07	0.88	1.29	0.515	Biological response modifiers including immunotherapy
Ulcerative colitis	Gastrointestinal	0.71	0.45	1.07	0.125	Biological response modifiers including immunotherapy
Agranulocytosis	Haematological	1.01	0.84	1.21	0.918	Biological response modifiers including immunotherapy
Aplastic anaemias	Haematological	0.69	0.53	0.90	0.007	Biological response modifiers including immunotherapy
Other anaemias	Haematological	0.80	0.74	0.86	< 0.001	Biological response modifiers including immunotherapy
Other haemolytic anaemias	Haematological	0.93	0.61	1.37	0.708	Biological response modifiers including immunotherapy
Primary or idiopathic thrombocytopaenia	Haematological	0.79	0.62	1.00	0.059	Biological response modifiers including immunotherapy
Secondary or other thrombocytopaenia	Haematological	0.81	0.66	0.99	0.047	Biological response modifiers including immunotherapy
Secondary polycythaemia	Haematological	0.72	0.47	1.07	0.124	Biological response modifiers including immunotherapy
Thrombophilia	Haematological	0.76	0.48	1.15	0.222	Biological response modifiers including immunotherapy
Autoimmune liver disease	Immunology and infection	0.85	0.56	1.24	0.425	Biological response modifiers including immunotherapy
Chronic viral hepatitis	Immunology and infection	1.11	0.87	1.40	0.388	Biological response modifiers including immunotherapy
Encephalitis	Immunology and infection	0.95	0.61	1.40	0.794	Biological response modifiers including immunotherapy
HIV	Immunology and infection	0.82	0.57	1.15	0.257	Biological response modifiers including immunotherapy
Immunodeficiencies	Immunology and infection	1.15	0.73	1.77	0.529	Biological response modifiers including immunotherapy
Infection of anal and rectal regions	Immunology and infection	0.99	0.56	1.61	0.959	Biological response modifiers including immunotherapy
Infection of bones and joints	Immunology and infection	1.13	0.93	1.36	0.198	Biological response modifiers including immunotherapy
Infection of liver	Immunology and infection	0.54	0.32	0.85	0.012	Biological response modifiers including immunotherapy
Infection of skin and subcutaneous tissues	Immunology and infection	0.73	0.64	0.83	< 0.001	Biological response modifiers including immunotherapy
Infections of other or unspecified organs	Immunology and infection	0.80	0.73	0.89	< 0.001	Biological response modifiers including immunotherapy
Infections of the digestive system	Immunology and infection	1.16	1.05	1.28	0.003	Biological response modifiers including immunotherapy
Infections of the heart	Immunology and infection	0.72	0.57	0.91	0.008	Biological response modifiers including immunotherapy
Lower respiratory tract infections	Immunology and infection	0.67	0.62	0.73	< 0.001	Biological response modifiers including immunotherapy
Lupus erythematosus (local and systemic)	Immunology and infection	1.07	0.57	1.86	0.812	Biological response modifiers including immunotherapy
Meningitis	Immunology and infection	0.84	0.67	1.04	0.114	Biological response modifiers including immunotherapy
Non-acute cystitis	Immunology and infection	0.86	0.61	1.19	0.388	Biological response modifiers including immunotherapy
Peritonitis	Immunology and infection	1.06	0.98	1.14	0.14	Biological response modifiers including immunotherapy
Viral diseases (excluding chronic hepatitis/HIV)	Immunology and infection	0.93	0.77	1.11	0.416	Biological response modifiers including immunotherapy
Ankylosing spondylitis	Musculoskeletal	1.26	0.63	2.31	0.49	Biological response modifiers including immunotherapy
Enteropathic arthropathy	Musculoskeletal	0.79	0.46	1.28	0.371	Biological response modifiers including immunotherapy
Enthesopathies and synovial disorders	Musculoskeletal	1.05	0.97	1.14	0.215	Biological response modifiers including immunotherapy
Osteoarthritis (excluding spine)	Musculoskeletal	1.06	0.99	1.14	0.099	Biological response modifiers including immunotherapy
Osteoporosis	Musculoskeletal	0.89	0.78	1.01	0.066	Biological response modifiers including immunotherapy
Rheumatoid arthritis	Musculoskeletal	0.72	0.56	0.90	0.005	Biological response modifiers including immunotherapy
Scoliosis	Musculoskeletal	0.70	0.45	1.03	0.089	Biological response modifiers including immunotherapy
Spinal stenosis	Musculoskeletal	1.19	0.99	1.43	0.063	Biological response modifiers including immunotherapy
Bell's palsy	Neurological	0.68	0.45	0.99	0.055	Biological response modifiers including immunotherapy
Dementia	Neurological	0.51	0.41	0.63	< 0.001	Biological response modifiers including immunotherapy
Disorders of autonomic nervous system	Neurological	1.38	1.12	1.69	0.002	Biological response modifiers including immunotherapy
Epilepsy	Neurological	0.85	0.63	1.13	0.275	Biological response modifiers including immunotherapy
Essential tremor	Neurological	1.21	0.80	1.76	0.348	Biological response modifiers including immunotherapy
Intracerebral haemorrhage	Neurological	0.38	0.25	0.56	< 0.001	Biological response modifiers including immunotherapy
Intracranial hypertension	Neurological	0.73	0.49	1.04	0.095	Biological response modifiers including immunotherapy
Peripheral neuropathies	Neurological	0.93	0.82	1.05	0.265	Biological response modifiers including immunotherapy
Subarachnoid haemorrhage	Neurological	0.41	0.26	0.60	< 0.001	Biological response modifiers including immunotherapy
Asthma	Pulmonary	0.71	0.63	0.81	< 0.001	Biological response modifiers including immunotherapy
Bronchiectasis	Pulmonary	0.85	0.62	1.13	0.274	Biological response modifiers including immunotherapy
COPD	Pulmonary	0.77	0.69	0.85	< 0.001	Biological response modifiers including immunotherapy
Other interstitial pulmonary diseases with fibrosis	Pulmonary	0.57	0.37	0.85	0.008	Biological response modifiers including immunotherapy
End stage renal disease	Renal	0.78	0.69	0.88	< 0.001	Biological response modifiers including immunotherapy
Glomerulonephritis	Renal	0.86	0.67	1.11	0.26	Biological response modifiers including immunotherapy
Obstructive and reflux uropathy	Renal	1.01	0.83	1.21	0.957	Biological response modifiers including immunotherapy
Urolithiasis	Renal	1.01	0.86	1.17	0.934	Biological response modifiers including immunotherapy
Abdominal aortic aneurysm	Cardiovascular	1.03	0.93	1.13	0.538	Hormonal agents
Atrial fibrillation	Cardiovascular	0.78	0.74	0.83	< 0.001	Hormonal agents
Atrioventricular block, complete	Cardiovascular	0.88	0.77	1.00	0.058	Hormonal agents
Atrioventricular block, first degree	Cardiovascular	0.88	0.77	1.00	0.058	Hormonal agents
Atrioventricular block, second degree	Cardiovascular	0.88	0.77	1.00	0.058	Hormonal agents
Bifascicular block	Cardiovascular	0.90	0.77	1.05	0.182	Hormonal agents
Coronary heart disease not otherwise specified	Cardiovascular	0.94	0.89	0.98	0.005	Hormonal agents
Dilated cardiomyopathy	Cardiovascular	0.78	0.56	1.07	0.146	Hormonal agents
Heart failure	Cardiovascular	0.76	0.71	0.82	< 0.001	Hormonal agents
Hypertension	Cardiovascular	0.97	0.93	1.00	0.075	Hormonal agents
Hypertrophic cardiomyopathy	Cardiovascular	0.73	0.51	1.02	0.076	Hormonal agents
Ischaemic stroke	Cardiovascular	0.78	0.69	0.88	< 0.001	Hormonal agents
Left bundle branch block	Cardiovascular	0.90	0.80	1.02	0.111	Hormonal agents
Multiple valve disease	Cardiovascular	0.86	0.73	1.01	0.079	Hormonal agents
Myocardial infarction	Cardiovascular	0.89	0.83	0.94	< 0.001	Hormonal agents
Nonrheumatic aortic valve disorders	Cardiovascular	0.82	0.71	0.94	0.005	Hormonal agents
Nonrheumatic mitral valve disorders	Cardiovascular	0.85	0.72	1.00	0.049	Hormonal agents
Other cardiomyopathy	Cardiovascular	0.89	0.84	0.95	< 0.001	Hormonal agents
Pericardial effusion (noninflammatory)	Cardiovascular	0.88	0.66	1.14	0.35	Hormonal agents
Peripheral arterial disease	Cardiovascular	0.78	0.70	0.86	< 0.001	Hormonal agents
Primary pulmonary hypertension	Cardiovascular	0.67	0.50	0.88	0.005	Hormonal agents
Raynaud's syndrome	Cardiovascular	0.84	0.74	0.94	0.003	Hormonal agents
Right bundle branch block	Cardiovascular	0.89	0.76	1.04	0.157	Hormonal agents
Secondary pulmonary hypertension	Cardiovascular	0.69	0.51	0.91	0.012	Hormonal agents
Sick sinus syndrome	Cardiovascular	0.89	0.72	1.07	0.224	Hormonal agents
Stable angina	Cardiovascular	0.86	0.80	0.92	< 0.001	Hormonal agents
Stroke (not otherwise specified)	Cardiovascular	0.78	0.71	0.86	< 0.001	Hormonal agents
Supraventricular tachycardia	Cardiovascular	0.73	0.60	0.87	0.001	Hormonal agents
Transient ischaemic attack	Cardiovascular	0.83	0.76	0.90	< 0.001	Hormonal agents
Trifascicular block	Cardiovascular	0.81	0.67	0.96	0.017	Hormonal agents
Unstable angina	Cardiovascular	0.85	0.79	0.91	< 0.001	Hormonal agents
Venous thromboembolic disease	Cardiovascular	0.82	0.75	0.88	< 0.001	Hormonal agents
Ventricular tachycardia	Cardiovascular	0.71	0.59	0.85	< 0.001	Hormonal agents
Diabetes	Endocrine	0.95	0.91	1.00	0.062	Hormonal agents
Diabetic neurological complications	Endocrine	0.95	0.89	1.00	0.063	Hormonal agents
Diabetic ophthalmic complications	Endocrine	0.94	0.89	0.99	0.032	Hormonal agents
Hyperparathyroidism	Endocrine	0.94	0.71	1.23	0.679	Hormonal agents
Hypo or hyperthyroidism	Endocrine	1.02	0.95	1.11	0.53	Hormonal agents
Alcoholic liver disease	Gastrointestinal	0.80	0.63	0.99	0.048	Hormonal agents
Angiodysplasia of colon	Gastrointestinal	0.94	0.70	1.25	0.701	Hormonal agents
Barrett's oesophagus	Gastrointestinal	0.94	0.85	1.04	0.233	Hormonal agents
Cholangitis	Gastrointestinal	0.89	0.81	0.97	0.01	Hormonal agents
Cholecystitis	Gastrointestinal	0.89	0.81	0.97	0.008	Hormonal agents
Cholelithiasis	Gastrointestinal	0.88	0.81	0.96	0.004	Hormonal agents
Coeliac disease	Gastrointestinal	1.42	1.18	1.69	< 0.001	Hormonal agents
Diverticular disease of intestine	Gastrointestinal	1.02	0.96	1.08	0.573	Hormonal agents
Fatty liver	Gastrointestinal	1.05	0.93	1.18	0.401	Hormonal agents
Gastritis and duodenitis	Gastrointestinal	0.96	0.90	1.02	0.159	Hormonal agents
Gastrooesophageal reflux disease	Gastrointestinal	1.00	0.94	1.06	0.92	Hormonal agents
Hepatic failure	Gastrointestinal	0.96	0.85	1.09	0.533	Hormonal agents
Liver fibrosis sclerosis and cirrhosis	Gastrointestinal	0.70	0.54	0.89	0.004	Hormonal agents
Oesophageal varices	Gastrointestinal	0.54	0.33	0.82	0.007	Hormonal agents
Oesophagitis and oesophageal ulcer	Gastrointestinal	0.97	0.91	1.02	0.215	Hormonal agents
Portal hypertension	Gastrointestinal	1.05	0.91	1.21	0.506	Hormonal agents
Ulcerative colitis	Gastrointestinal	0.97	0.75	1.24	0.829	Hormonal agents
Agranulocytosis	Haematological	1.00	0.82	1.21	0.973	Hormonal agents
Aplastic anaemias	Haematological	0.85	0.64	1.11	0.257	Hormonal agents
Other anaemias	Haematological	0.79	0.74	0.84	< 0.001	Hormonal agents
Other haemolytic anaemias	Haematological	1.24	0.85	1.75	0.241	Hormonal agents
Primary or idiopathic thrombocytopaenia	Haematological	0.66	0.51	0.83	0.001	Hormonal agents
Secondary or other thrombocytopaenia	Haematological	0.73	0.60	0.87	0.001	Hormonal agents
Secondary polycythaemia	Haematological	0.68	0.47	0.94	0.027	Hormonal agents
Thrombophilia	Haematological	0.66	0.47	0.91	0.015	Hormonal agents
Autoimmune liver disease	Immunology and infection	0.82	0.61	1.07	0.156	Hormonal agents
Chronic viral hepatitis	Immunology and infection	1.34	1.14	1.56	< 0.001	Hormonal agents
Encephalitis	Immunology and infection	0.92	0.66	1.24	0.593	Hormonal agents
HIV	Immunology and infection	1.18	0.91	1.50	0.206	Hormonal agents
Infection of anal and rectal regions	Immunology and infection	0.87	0.58	1.26	0.496	Hormonal agents
Infection of bones and joints	Immunology and infection	0.74	0.62	0.87	0.001	Hormonal agents
Infection of liver	Immunology and infection	1.04	0.77	1.37	0.801	Hormonal agents
Infection of skin and subcutaneous tissues	Immunology and infection	0.88	0.80	0.96	0.003	Hormonal agents
Infections of other or unspecified organs	Immunology and infection	0.75	0.71	0.81	< 0.001	Hormonal agents
Infections of the digestive system	Immunology and infection	1.13	1.06	1.22	0.001	Hormonal agents
Infections of the heart	Immunology and infection	0.76	0.63	0.90	0.002	Hormonal agents
Lower respiratory tract infections	Immunology and infection	0.82	0.77	0.86	< 0.001	Hormonal agents
Lupus erythematosus (local and systemic)	Immunology and infection	1.19	0.76	1.78	0.42	Hormonal agents
Meningitis	Immunology and infection	0.83	0.70	0.98	0.028	Hormonal agents
Non-acute cystitis	Immunology and infection	0.94	0.78	1.14	0.557	Hormonal agents
Peritonitis	Immunology and infection	1.04	0.99	1.10	0.107	Hormonal agents
Viral diseases (excluding chronic hepatitis/HIV)	Immunology and infection	0.95	0.82	1.08	0.412	Hormonal agents
Ankylosing spondylitis	Musculoskeletal	1.29	0.78	2.03	0.291	Hormonal agents
Enteropathic arthropathy	Musculoskeletal	1.00	0.73	1.33	0.994	Hormonal agents
Enthesopathies and synovial disorders	Musculoskeletal	1.02	0.97	1.07	0.46	Hormonal agents
Osteoarthritis (excluding spine)	Musculoskeletal	1.01	0.97	1.06	0.519	Hormonal agents
Osteoporosis	Musculoskeletal	0.90	0.83	0.97	0.009	Hormonal agents
Rheumatoid arthritis	Musculoskeletal	0.84	0.72	0.98	0.025	Hormonal agents
Scoliosis	Musculoskeletal	0.80	0.62	1.02	0.086	Hormonal agents
Spinal stenosis	Musculoskeletal	1.01	0.88	1.16	0.858	Hormonal agents
Bell's palsy	Neurological	0.79	0.60	1.02	0.074	Hormonal agents
Dementia	Neurological	0.72	0.65	0.81	< 0.001	Hormonal agents
Disorders of autonomic nervous system	Neurological	1.03	0.86	1.22	0.756	Hormonal agents
Epilepsy	Neurological	0.91	0.76	1.09	0.309	Hormonal agents
Essential tremor	Neurological	1.17	0.90	1.50	0.22	Hormonal agents
Intracerebral haemorrhage	Neurological	0.73	0.60	0.86	< 0.001	Hormonal agents
Intracranial hypertension	Neurological	0.94	0.72	1.19	0.612	Hormonal agents
Multiple sclerosis	Neurological	0.70	0.40	1.15	0.188	Hormonal agents
Peripheral neuropathies	Neurological	0.90	0.83	0.99	0.024	Hormonal agents
Subarachnoid haemorrhage	Neurological	0.71	0.59	0.86	0.001	Hormonal agents
Asthma	Pulmonary	0.95	0.88	1.02	0.126	Hormonal agents
Bronchiectasis	Pulmonary	0.81	0.66	0.99	0.043	Hormonal agents
COPD	Pulmonary	0.94	0.89	1.00	0.047	Hormonal agents
Other interstitial pulmonary diseases with fibrosis	Pulmonary	0.69	0.53	0.87	0.003	Hormonal agents
End stage renal disease	Renal	0.80	0.73	0.86	< 0.001	Hormonal agents
Glomerulonephritis	Renal	0.86	0.72	1.02	0.081	Hormonal agents
Obstructive and reflux uropathy	Renal	0.96	0.85	1.08	0.476	Hormonal agents
Urolithiasis	Renal	0.91	0.83	1.00	0.063	Hormonal agents
Abdominal aortic aneurysm	Cardiovascular	1.15	0.85	1.53	0.346	Kinase inhibitors
Atrial fibrillation	Cardiovascular	0.68	0.53	0.86	0.002	Kinase inhibitors
Atrioventricular block, complete	Cardiovascular	0.62	0.34	1.02	0.085	Kinase inhibitors
Atrioventricular block, first degree	Cardiovascular	0.62	0.34	1.02	0.085	Kinase inhibitors
Atrioventricular block, second degree	Cardiovascular	0.62	0.34	1.02	0.085	Kinase inhibitors
Bifascicular block	Cardiovascular	1.15	0.69	1.80	0.556	Kinase inhibitors
Coronary heart disease not otherwise specified	Cardiovascular	0.76	0.62	0.91	0.004	Kinase inhibitors
Heart failure	Cardiovascular	0.63	0.47	0.81	0.001	Kinase inhibitors
Hypertension	Cardiovascular	0.88	0.76	1.01	0.07	Kinase inhibitors
Ischaemic stroke	Cardiovascular	0.42	0.22	0.70	0.003	Kinase inhibitors
Left bundle branch block	Cardiovascular	0.59	0.32	0.97	0.058	Kinase inhibitors
Multiple valve disease	Cardiovascular	0.76	0.39	1.33	0.38	Kinase inhibitors
Myocardial infarction	Cardiovascular	0.63	0.49	0.81	< 0.001	Kinase inhibitors
Nonrheumatic aortic valve disorders	Cardiovascular	0.68	0.37	1.14	0.179	Kinase inhibitors
Other cardiomyopathy	Cardiovascular	0.65	0.51	0.82	< 0.001	Kinase inhibitors
Peripheral arterial disease	Cardiovascular	0.54	0.35	0.79	0.002	Kinase inhibitors
Raynaud's syndrome	Cardiovascular	0.56	0.35	0.85	0.011	Kinase inhibitors
Right bundle branch block	Cardiovascular	1.16	0.70	1.82	0.53	Kinase inhibitors
Stable angina	Cardiovascular	0.75	0.56	0.98	0.041	Kinase inhibitors
Stroke (not otherwise specified)	Cardiovascular	0.45	0.27	0.71	0.001	Kinase inhibitors
Transient ischaemic attack	Cardiovascular	0.82	0.60	1.09	0.181	Kinase inhibitors
Trifascicular block	Cardiovascular	1.14	0.66	1.82	0.614	Kinase inhibitors
Unstable angina	Cardiovascular	0.76	0.57	0.98	0.045	Kinase inhibitors
Venous thromboembolic disease	Cardiovascular	0.55	0.40	0.73	< 0.001	Kinase inhibitors
Diabetes	Endocrine	1.01	0.85	1.20	0.869	Kinase inhibitors
Diabetic neurological complications	Endocrine	0.83	0.68	1.01	0.073	Kinase inhibitors
Diabetic ophthalmic complications	Endocrine	0.86	0.71	1.04	0.12	Kinase inhibitors
Hypo or hyperthyroidism	Endocrine	1.00	0.74	1.33	0.98	Kinase inhibitors
Alcoholic liver disease	Gastrointestinal	1.22	0.75	1.87	0.388	Kinase inhibitors
Barrett's oesophagus	Gastrointestinal	1.02	0.70	1.42	0.928	Kinase inhibitors
Cholangitis	Gastrointestinal	0.67	0.48	0.90	0.012	Kinase inhibitors
Cholecystitis	Gastrointestinal	0.75	0.53	1.02	0.082	Kinase inhibitors
Cholelithiasis	Gastrointestinal	0.70	0.50	0.96	0.034	Kinase inhibitors
Coeliac disease	Gastrointestinal	1.26	0.64	2.21	0.462	Kinase inhibitors
Diverticular disease of intestine	Gastrointestinal	1.03	0.82	1.27	0.823	Kinase inhibitors
Fatty liver	Gastrointestinal	1.30	0.98	1.68	0.058	Kinase inhibitors
Gastritis and duodenitis	Gastrointestinal	0.90	0.72	1.11	0.348	Kinase inhibitors
Gastrooesophageal reflux disease	Gastrointestinal	0.91	0.71	1.14	0.411	Kinase inhibitors
Hepatic failure	Gastrointestinal	1.25	0.94	1.64	0.11	Kinase inhibitors
Liver fibrosis sclerosis and cirrhosis	Gastrointestinal	1.35	0.89	1.97	0.14	Kinase inhibitors
Oesophageal varices	Gastrointestinal	1.21	0.65	2.08	0.515	Kinase inhibitors
Oesophagitis and oesophageal ulcer	Gastrointestinal	0.97	0.79	1.17	0.729	Kinase inhibitors
Portal hypertension	Gastrointestinal	1.49	1.10	1.97	0.007	Kinase inhibitors
Other anaemias	Haematological	0.82	0.67	0.99	0.039	Kinase inhibitors
Primary or idiopathic thrombocytopaenia	Haematological	0.61	0.33	1.04	0.098	Kinase inhibitors
Secondary or other thrombocytopaenia	Haematological	0.71	0.43	1.09	0.146	Kinase inhibitors
Autoimmune liver disease	Immunology and infection	1.41	0.87	2.17	0.14	Kinase inhibitors
Chronic viral hepatitis	Immunology and infection	1.11	0.68	1.70	0.651	Kinase inhibitors
Infection of bones and joints	Immunology and infection	1.43	0.98	2.03	0.05	Kinase inhibitors
Infection of liver	Immunology and infection	1.46	0.84	2.37	0.15	Kinase inhibitors
Infection of skin and subcutaneous tissues	Immunology and infection	0.80	0.59	1.05	0.123	Kinase inhibitors
Infections of other or unspecified organs	Immunology and infection	0.83	0.67	1.02	0.078	Kinase inhibitors
Infections of the digestive system	Immunology and infection	1.12	0.88	1.39	0.345	Kinase inhibitors
Infections of the heart	Immunology and infection	0.82	0.51	1.25	0.386	Kinase inhibitors
Lower respiratory tract infections	Immunology and infection	0.53	0.45	0.63	< 0.001	Kinase inhibitors
Meningitis	Immunology and infection	0.77	0.45	1.21	0.288	Kinase inhibitors
Peritonitis	Immunology and infection	1.04	0.87	1.23	0.687	Kinase inhibitors
Viral diseases (excluding chronic hepatitis/HIV)	Immunology and infection	0.89	0.57	1.31	0.569	Kinase inhibitors
Enthesopathies and synovial disorders	Musculoskeletal	1.23	1.03	1.47	0.022	Kinase inhibitors
Osteoarthritis (excluding spine)	Musculoskeletal	1.06	0.89	1.25	0.521	Kinase inhibitors
Osteoporosis	Musculoskeletal	0.88	0.63	1.18	0.411	Kinase inhibitors
Rheumatoid arthritis	Musculoskeletal	0.93	0.56	1.44	0.759	Kinase inhibitors
Spinal stenosis	Musculoskeletal	1.14	0.71	1.72	0.571	Kinase inhibitors
Peripheral neuropathies	Neurological	1.12	0.83	1.48	0.43	Kinase inhibitors
Asthma	Pulmonary	0.85	0.65	1.09	0.217	Kinase inhibitors
Bronchiectasis	Pulmonary	1.03	0.56	1.71	0.927	Kinase inhibitors
COPD	Pulmonary	0.64	0.52	0.78	< 0.001	Kinase inhibitors
End stage renal disease	Renal	0.61	0.45	0.80	0.001	Kinase inhibitors
Glomerulonephritis	Renal	0.99	0.57	1.58	0.96	Kinase inhibitors
Obstructive and reflux uropathy	Renal	0.48	0.25	0.81	0.012	Kinase inhibitors
Urolithiasis	Renal	0.76	0.53	1.06	0.125	Kinase inhibitors
Abdominal aortic aneurysm	Cardiovascular	1.04	0.64	1.58	0.879	Non-anthracycline antitumour antibiotics
Atrial fibrillation	Cardiovascular	0.73	0.50	1.02	0.077	Non-anthracycline antitumour antibiotics
Atrioventricular block, complete	Cardiovascular	1.59	0.90	2.59	0.087	Non-anthracycline antitumour antibiotics
Atrioventricular block, first degree	Cardiovascular	1.59	0.90	2.59	0.087	Non-anthracycline antitumour antibiotics
Atrioventricular block, second degree	Cardiovascular	1.59	0.90	2.59	0.087	Non-anthracycline antitumour antibiotics
Coronary heart disease not otherwise specified	Cardiovascular	0.86	0.66	1.10	0.239	Non-anthracycline antitumour antibiotics
Heart failure	Cardiovascular	0.67	0.45	0.98	0.047	Non-anthracycline antitumour antibiotics
Hypertension	Cardiovascular	1.01	0.82	1.23	0.92	Non-anthracycline antitumour antibiotics
Ischaemic stroke	Cardiovascular	1.09	0.58	1.86	0.773	Non-anthracycline antitumour antibiotics
Left bundle branch block	Cardiovascular	1.57	0.90	2.53	0.086	Non-anthracycline antitumour antibiotics
Myocardial infarction	Cardiovascular	1.02	0.74	1.36	0.91	Non-anthracycline antitumour antibiotics
Other cardiomyopathy	Cardiovascular	0.95	0.70	1.26	0.73	Non-anthracycline antitumour antibiotics
Peripheral arterial disease	Cardiovascular	0.69	0.36	1.18	0.209	Non-anthracycline antitumour antibiotics
Stable angina	Cardiovascular	1.17	0.82	1.60	0.366	Non-anthracycline antitumour antibiotics
Stroke (not otherwise specified)	Cardiovascular	0.80	0.43	1.34	0.434	Non-anthracycline antitumour antibiotics
Transient ischaemic attack	Cardiovascular	1.08	0.72	1.56	0.699	Non-anthracycline antitumour antibiotics
Unstable angina	Cardiovascular	0.99	0.69	1.38	0.952	Non-anthracycline antitumour antibiotics
Venous thromboembolic disease	Cardiovascular	0.85	0.54	1.25	0.432	Non-anthracycline antitumour antibiotics
Diabetes	Endocrine	0.96	0.74	1.23	0.745	Non-anthracycline antitumour antibiotics
Diabetic neurological complications	Endocrine	0.88	0.65	1.18	0.408	Non-anthracycline antitumour antibiotics
Diabetic ophthalmic complications	Endocrine	0.94	0.71	1.23	0.676	Non-anthracycline antitumour antibiotics
Hypo or hyperthyroidism	Endocrine	0.96	0.59	1.47	0.851	Non-anthracycline antitumour antibiotics
Barrett's oesophagus	Gastrointestinal	1.23	0.74	1.91	0.399	Non-anthracycline antitumour antibiotics
Cholangitis	Gastrointestinal	0.84	0.51	1.32	0.483	Non-anthracycline antitumour antibiotics
Cholecystitis	Gastrointestinal	0.75	0.43	1.21	0.274	Non-anthracycline antitumour antibiotics
Cholelithiasis	Gastrointestinal	0.69	0.40	1.10	0.146	Non-anthracycline antitumour antibiotics
Diverticular disease of intestine	Gastrointestinal	1.07	0.78	1.45	0.651	Non-anthracycline antitumour antibiotics
Fatty liver	Gastrointestinal	0.76	0.40	1.31	0.362	Non-anthracycline antitumour antibiotics
Gastritis and duodenitis	Gastrointestinal	1.02	0.73	1.37	0.921	Non-anthracycline antitumour antibiotics
Gastrooesophageal reflux disease	Gastrointestinal	1.13	0.81	1.53	0.446	Non-anthracycline antitumour antibiotics
Hepatic failure	Gastrointestinal	0.74	0.38	1.31	0.342	Non-anthracycline antitumour antibiotics
Oesophagitis and oesophageal ulcer	Gastrointestinal	1.14	0.86	1.49	0.358	Non-anthracycline antitumour antibiotics
Other anaemias	Haematological	0.75	0.57	0.98	0.043	Non-anthracycline antitumour antibiotics
HIV	Immunology and infection	1.54	0.73	3.00	0.227	Non-anthracycline antitumour antibiotics
Infection of skin and subcutaneous tissues	Immunology and infection	0.93	0.62	1.34	0.721	Non-anthracycline antitumour antibiotics
Infections of other or unspecified organs	Immunology and infection	0.78	0.56	1.06	0.122	Non-anthracycline antitumour antibiotics
Infections of the digestive system	Immunology and infection	0.96	0.67	1.33	0.796	Non-anthracycline antitumour antibiotics
Lower respiratory tract infections	Immunology and infection	0.72	0.55	0.95	0.022	Non-anthracycline antitumour antibiotics
Meningitis	Immunology and infection	1.18	0.59	2.11	0.606	Non-anthracycline antitumour antibiotics
Peritonitis	Immunology and infection	1.06	0.82	1.36	0.626	Non-anthracycline antitumour antibiotics
Viral diseases (excluding chronic hepatitis/HIV)	Immunology and infection	1.26	0.75	2.00	0.345	Non-anthracycline antitumour antibiotics
Enthesopathies and synovial disorders	Musculoskeletal	1.03	0.78	1.34	0.841	Non-anthracycline antitumour antibiotics
Osteoarthritis (excluding spine)	Musculoskeletal	0.94	0.71	1.21	0.636	Non-anthracycline antitumour antibiotics
Osteoporosis	Musculoskeletal	0.74	0.42	1.21	0.267	Non-anthracycline antitumour antibiotics
Rheumatoid arthritis	Musculoskeletal	1.29	0.65	2.30	0.424	Non-anthracycline antitumour antibiotics
Peripheral neuropathies	Neurological	0.86	0.52	1.35	0.55	Non-anthracycline antitumour antibiotics
Asthma	Pulmonary	0.96	0.66	1.35	0.828	Non-anthracycline antitumour antibiotics
COPD	Pulmonary	0.87	0.62	1.19	0.396	Non-anthracycline antitumour antibiotics
End stage renal disease	Renal	0.62	0.39	0.93	0.031	Non-anthracycline antitumour antibiotics
Obstructive and reflux uropathy	Renal	0.56	0.33	0.89	0.023	Non-anthracycline antitumour antibiotics
Urolithiasis	Renal	0.61	0.40	0.90	0.017	Non-anthracycline antitumour antibiotics
Abdominal aortic aneurysm	Cardiovascular	1.35	1.17	1.54	< 0.001	Plant alkaloids (excluding vinca alkaloids)
Atrial fibrillation	Cardiovascular	0.63	0.56	0.71	< 0.001	Plant alkaloids (excluding vinca alkaloids)
Atrioventricular block, complete	Cardiovascular	0.86	0.67	1.08	0.21	Plant alkaloids (excluding vinca alkaloids)
Atrioventricular block, first degree	Cardiovascular	0.86	0.67	1.08	0.21	Plant alkaloids (excluding vinca alkaloids)
Atrioventricular block, second degree	Cardiovascular	0.86	0.67	1.08	0.21	Plant alkaloids (excluding vinca alkaloids)
Bifascicular block	Cardiovascular	0.98	0.74	1.26	0.857	Plant alkaloids (excluding vinca alkaloids)
Coronary heart disease not otherwise specified	Cardiovascular	0.78	0.72	0.85	< 0.001	Plant alkaloids (excluding vinca alkaloids)
Dilated cardiomyopathy	Cardiovascular	0.84	0.49	1.35	0.505	Plant alkaloids (excluding vinca alkaloids)
Heart failure	Cardiovascular	0.64	0.56	0.73	< 0.001	Plant alkaloids (excluding vinca alkaloids)
Hypertension	Cardiovascular	0.86	0.81	0.91	< 0.001	Plant alkaloids (excluding vinca alkaloids)
Hypertrophic cardiomyopathy	Cardiovascular	0.85	0.50	1.36	0.532	Plant alkaloids (excluding vinca alkaloids)
Ischaemic stroke	Cardiovascular	0.52	0.40	0.66	< 0.001	Plant alkaloids (excluding vinca alkaloids)
Left bundle branch block	Cardiovascular	0.85	0.67	1.06	0.171	Plant alkaloids (excluding vinca alkaloids)
Multiple valve disease	Cardiovascular	0.85	0.62	1.12	0.26	Plant alkaloids (excluding vinca alkaloids)
Myocardial infarction	Cardiovascular	0.70	0.62	0.78	< 0.001	Plant alkaloids (excluding vinca alkaloids)
Nonrheumatic aortic valve disorders	Cardiovascular	0.76	0.58	0.98	0.043	Plant alkaloids (excluding vinca alkaloids)
Nonrheumatic mitral valve disorders	Cardiovascular	0.81	0.59	1.07	0.156	Plant alkaloids (excluding vinca alkaloids)
Other cardiomyopathy	Cardiovascular	0.71	0.63	0.79	< 0.001	Plant alkaloids (excluding vinca alkaloids)
Pericardial effusion (noninflammatory)	Cardiovascular	0.87	0.60	1.21	0.416	Plant alkaloids (excluding vinca alkaloids)
Peripheral arterial disease	Cardiovascular	0.61	0.50	0.73	< 0.001	Plant alkaloids (excluding vinca alkaloids)
Primary pulmonary hypertension	Cardiovascular	0.40	0.21	0.68	0.002	Plant alkaloids (excluding vinca alkaloids)
Raynaud's syndrome	Cardiovascular	0.67	0.55	0.82	< 0.001	Plant alkaloids (excluding vinca alkaloids)
Right bundle branch block	Cardiovascular	0.99	0.75	1.27	0.927	Plant alkaloids (excluding vinca alkaloids)
Secondary pulmonary hypertension	Cardiovascular	0.42	0.22	0.72	0.003	Plant alkaloids (excluding vinca alkaloids)
Sick sinus syndrome	Cardiovascular	0.87	0.60	1.20	0.411	Plant alkaloids (excluding vinca alkaloids)
Stable angina	Cardiovascular	0.64	0.56	0.74	< 0.001	Plant alkaloids (excluding vinca alkaloids)
Stroke (not otherwise specified)	Cardiovascular	0.57	0.46	0.70	< 0.001	Plant alkaloids (excluding vinca alkaloids)
Supraventricular tachycardia	Cardiovascular	0.84	0.63	1.10	0.224	Plant alkaloids (excluding vinca alkaloids)
Transient ischaemic attack	Cardiovascular	0.84	0.72	0.96	0.012	Plant alkaloids (excluding vinca alkaloids)
Trifascicular block	Cardiovascular	0.99	0.74	1.30	0.971	Plant alkaloids (excluding vinca alkaloids)
Unstable angina	Cardiovascular	0.65	0.56	0.74	< 0.001	Plant alkaloids (excluding vinca alkaloids)
Venous thromboembolic disease	Cardiovascular	0.62	0.53	0.71	< 0.001	Plant alkaloids (excluding vinca alkaloids)
Ventricular tachycardia	Cardiovascular	0.86	0.65	1.13	0.294	Plant alkaloids (excluding vinca alkaloids)
Diabetes	Endocrine	0.93	0.86	1.00	0.05	Plant alkaloids (excluding vinca alkaloids)
Diabetic neurological complications	Endocrine	0.87	0.80	0.96	0.005	Plant alkaloids (excluding vinca alkaloids)
Diabetic ophthalmic complications	Endocrine	0.88	0.80	0.96	0.004	Plant alkaloids (excluding vinca alkaloids)
Hyperparathyroidism	Endocrine	0.93	0.58	1.41	0.742	Plant alkaloids (excluding vinca alkaloids)
Hypo or hyperthyroidism	Endocrine	0.83	0.73	0.94	0.006	Plant alkaloids (excluding vinca alkaloids)
Alcoholic liver disease	Gastrointestinal	0.64	0.45	0.89	0.011	Plant alkaloids (excluding vinca alkaloids)
Angiodysplasia of colon	Gastrointestinal	0.76	0.46	1.20	0.27	Plant alkaloids (excluding vinca alkaloids)
Barrett's oesophagus	Gastrointestinal	0.96	0.82	1.12	0.642	Plant alkaloids (excluding vinca alkaloids)
Cholangitis	Gastrointestinal	1.00	0.88	1.12	0.943	Plant alkaloids (excluding vinca alkaloids)
Cholecystitis	Gastrointestinal	0.90	0.78	1.02	0.103	Plant alkaloids (excluding vinca alkaloids)
Cholelithiasis	Gastrointestinal	0.87	0.76	0.99	0.031	Plant alkaloids (excluding vinca alkaloids)
Coeliac disease	Gastrointestinal	1.44	1.14	1.81	0.002	Plant alkaloids (excluding vinca alkaloids)
Crohn's disease	Gastrointestinal	0.82	0.52	1.24	0.372	Plant alkaloids (excluding vinca alkaloids)
Diverticular disease of intestine	Gastrointestinal	1.05	0.96	1.16	0.281	Plant alkaloids (excluding vinca alkaloids)
Fatty liver	Gastrointestinal	1.01	0.86	1.17	0.929	Plant alkaloids (excluding vinca alkaloids)
Gastritis and duodenitis	Gastrointestinal	0.95	0.87	1.05	0.349	Plant alkaloids (excluding vinca alkaloids)
Gastrooesophageal reflux disease	Gastrointestinal	1.06	0.96	1.16	0.228	Plant alkaloids (excluding vinca alkaloids)
Hepatic failure	Gastrointestinal	0.97	0.83	1.14	0.749	Plant alkaloids (excluding vinca alkaloids)
Liver fibrosis sclerosis and cirrhosis	Gastrointestinal	0.47	0.30	0.70	< 0.001	Plant alkaloids (excluding vinca alkaloids)
Oesophagitis and oesophageal ulcer	Gastrointestinal	0.91	0.83	0.99	0.04	Plant alkaloids (excluding vinca alkaloids)
Portal hypertension	Gastrointestinal	1.07	0.90	1.27	0.43	Plant alkaloids (excluding vinca alkaloids)
Ulcerative colitis	Gastrointestinal	0.92	0.61	1.33	0.686	Plant alkaloids (excluding vinca alkaloids)
Agranulocytosis	Haematological	1.27	0.93	1.70	0.117	Plant alkaloids (excluding vinca alkaloids)
Aplastic anaemias	Haematological	0.59	0.31	1.01	0.077	Plant alkaloids (excluding vinca alkaloids)
Other anaemias	Haematological	0.64	0.58	0.70	< 0.001	Plant alkaloids (excluding vinca alkaloids)
Primary or idiopathic thrombocytopaenia	Haematological	0.63	0.41	0.94	0.031	Plant alkaloids (excluding vinca alkaloids)
Secondary or other thrombocytopaenia	Haematological	0.76	0.55	1.03	0.095	Plant alkaloids (excluding vinca alkaloids)
Thrombophilia	Haematological	0.80	0.48	1.26	0.367	Plant alkaloids (excluding vinca alkaloids)
Autoimmune liver disease	Immunology and infection	0.71	0.45	1.06	0.11	Plant alkaloids (excluding vinca alkaloids)
Chronic viral hepatitis	Immunology and infection	1.29	1.04	1.58	0.016	Plant alkaloids (excluding vinca alkaloids)
Encephalitis	Immunology and infection	0.97	0.55	1.58	0.911	Plant alkaloids (excluding vinca alkaloids)
HIV	Immunology and infection	0.84	0.58	1.18	0.32	Plant alkaloids (excluding vinca alkaloids)
Infection of anal and rectal regions	Immunology and infection	0.80	0.45	1.32	0.41	Plant alkaloids (excluding vinca alkaloids)
Infection of bones and joints	Immunology and infection	0.60	0.45	0.78	< 0.001	Plant alkaloids (excluding vinca alkaloids)
Infection of liver	Immunology and infection	0.91	0.57	1.37	0.663	Plant alkaloids (excluding vinca alkaloids)
Infection of skin and subcutaneous tissues	Immunology and infection	0.76	0.66	0.87	< 0.001	Plant alkaloids (excluding vinca alkaloids)
Infections of other or unspecified organs	Immunology and infection	0.72	0.64	0.80	< 0.001	Plant alkaloids (excluding vinca alkaloids)
Infections of the digestive system	Immunology and infection	1.14	1.03	1.25	0.01	Plant alkaloids (excluding vinca alkaloids)
Infections of the heart	Immunology and infection	0.56	0.41	0.73	< 0.001	Plant alkaloids (excluding vinca alkaloids)
Lower respiratory tract infections	Immunology and infection	0.72	0.66	0.78	< 0.001	Plant alkaloids (excluding vinca alkaloids)
Meningitis	Immunology and infection	0.66	0.50	0.85	0.002	Plant alkaloids (excluding vinca alkaloids)
Non-acute cystitis	Immunology and infection	0.86	0.61	1.19	0.39	Plant alkaloids (excluding vinca alkaloids)
Peritonitis	Immunology and infection	1.04	0.96	1.13	0.296	Plant alkaloids (excluding vinca alkaloids)
Viral diseases (excluding chronic hepatitis/HIV)	Immunology and infection	0.93	0.75	1.12	0.445	Plant alkaloids (excluding vinca alkaloids)
Ankylosing spondylitis	Musculoskeletal	1.50	0.75	2.73	0.218	Plant alkaloids (excluding vinca alkaloids)
Enteropathic arthropathy	Musculoskeletal	0.60	0.32	1.01	0.077	Plant alkaloids (excluding vinca alkaloids)
Enthesopathies and synovial disorders	Musculoskeletal	1.04	0.96	1.13	0.32	Plant alkaloids (excluding vinca alkaloids)
Osteoarthritis (excluding spine)	Musculoskeletal	0.96	0.89	1.04	0.295	Plant alkaloids (excluding vinca alkaloids)
Osteoporosis	Musculoskeletal	0.66	0.56	0.76	< 0.001	Plant alkaloids (excluding vinca alkaloids)
Rheumatoid arthritis	Musculoskeletal	0.78	0.61	0.98	0.042	Plant alkaloids (excluding vinca alkaloids)
Scoliosis	Musculoskeletal	0.50	0.29	0.79	0.007	Plant alkaloids (excluding vinca alkaloids)
Spinal stenosis	Musculoskeletal	1.17	0.94	1.44	0.147	Plant alkaloids (excluding vinca alkaloids)
Bell's palsy	Neurological	0.72	0.46	1.09	0.143	Plant alkaloids (excluding vinca alkaloids)
Dementia	Neurological	0.17	0.11	0.25	< 0.001	Plant alkaloids (excluding vinca alkaloids)
Disorders of autonomic nervous system	Neurological	0.86	0.63	1.13	0.287	Plant alkaloids (excluding vinca alkaloids)
Epilepsy	Neurological	0.68	0.48	0.93	0.022	Plant alkaloids (excluding vinca alkaloids)
Essential tremor	Neurological	1.37	0.92	1.98	0.101	Plant alkaloids (excluding vinca alkaloids)
Intracerebral haemorrhage	Neurological	0.42	0.27	0.61	< 0.001	Plant alkaloids (excluding vinca alkaloids)
Intracranial hypertension	Neurological	0.83	0.57	1.17	0.315	Plant alkaloids (excluding vinca alkaloids)
Peripheral neuropathies	Neurological	0.87	0.76	1.00	0.063	Plant alkaloids (excluding vinca alkaloids)
Subarachnoid haemorrhage	Neurological	0.47	0.31	0.69	< 0.001	Plant alkaloids (excluding vinca alkaloids)
Asthma	Pulmonary	0.77	0.68	0.87	< 0.001	Plant alkaloids (excluding vinca alkaloids)
Bronchiectasis	Pulmonary	0.78	0.56	1.06	0.136	Plant alkaloids (excluding vinca alkaloids)
COPD	Pulmonary	0.88	0.80	0.96	0.006	Plant alkaloids (excluding vinca alkaloids)
Other interstitial pulmonary diseases with fibrosis	Pulmonary	0.73	0.51	1.01	0.067	Plant alkaloids (excluding vinca alkaloids)
End stage renal disease	Renal	0.73	0.64	0.84	< 0.001	Plant alkaloids (excluding vinca alkaloids)
Glomerulonephritis	Renal	0.57	0.39	0.80	0.002	Plant alkaloids (excluding vinca alkaloids)
Obstructive and reflux uropathy	Renal	0.99	0.82	1.19	0.929	Plant alkaloids (excluding vinca alkaloids)
Urolithiasis	Renal	0.97	0.83	1.12	0.656	Plant alkaloids (excluding vinca alkaloids)
Abdominal aortic aneurysm	Cardiovascular	1.36	1.23	1.51	< 0.001	Platinum agents
Atrial fibrillation	Cardiovascular	0.61	0.55	0.66	< 0.001	Platinum agents
Atrioventricular block, complete	Cardiovascular	0.70	0.58	0.84	< 0.001	Platinum agents
Atrioventricular block, first degree	Cardiovascular	0.70	0.58	0.84	< 0.001	Platinum agents
Atrioventricular block, second degree	Cardiovascular	0.70	0.58	0.84	< 0.001	Platinum agents
Bifascicular block	Cardiovascular	0.65	0.52	0.80	< 0.001	Platinum agents
Coronary heart disease not otherwise specified	Cardiovascular	0.80	0.74	0.85	< 0.001	Platinum agents
Dilated cardiomyopathy	Cardiovascular	0.86	0.58	1.23	0.426	Platinum agents
Heart failure	Cardiovascular	0.65	0.59	0.71	< 0.001	Platinum agents
Hypertension	Cardiovascular	0.94	0.89	0.98	0.01	Platinum agents
Hypertrophic cardiomyopathy	Cardiovascular	0.85	0.57	1.22	0.409	Platinum agents
Ischaemic stroke	Cardiovascular	0.47	0.39	0.56	< 0.001	Platinum agents
Left bundle branch block	Cardiovascular	0.72	0.60	0.85	< 0.001	Platinum agents
Multiple valve disease	Cardiovascular	0.74	0.59	0.93	0.011	Platinum agents
Myocardial infarction	Cardiovascular	0.71	0.65	0.78	< 0.001	Platinum agents
Nonrheumatic aortic valve disorders	Cardiovascular	0.79	0.65	0.95	0.013	Platinum agents
Nonrheumatic mitral valve disorders	Cardiovascular	0.77	0.61	0.96	0.024	Platinum agents
Other cardiomyopathy	Cardiovascular	0.71	0.65	0.76	< 0.001	Platinum agents
Pericardial effusion (noninflammatory)	Cardiovascular	0.78	0.59	1.03	0.084	Platinum agents
Peripheral arterial disease	Cardiovascular	0.66	0.58	0.75	< 0.001	Platinum agents
Primary pulmonary hypertension	Cardiovascular	0.31	0.19	0.47	< 0.001	Platinum agents
Raynaud's syndrome	Cardiovascular	0.69	0.60	0.80	< 0.001	Platinum agents
Right bundle branch block	Cardiovascular	0.66	0.52	0.81	< 0.001	Platinum agents
Secondary pulmonary hypertension	Cardiovascular	0.31	0.19	0.48	< 0.001	Platinum agents
Sick sinus syndrome	Cardiovascular	0.93	0.71	1.19	0.56	Platinum agents
Stable angina	Cardiovascular	0.70	0.63	0.77	< 0.001	Platinum agents
Stroke (not otherwise specified)	Cardiovascular	0.60	0.51	0.69	< 0.001	Platinum agents
Supraventricular tachycardia	Cardiovascular	0.75	0.60	0.94	0.016	Platinum agents
Transient ischaemic attack	Cardiovascular	0.80	0.72	0.89	< 0.001	Platinum agents
Trifascicular block	Cardiovascular	0.65	0.51	0.82	< 0.001	Platinum agents
Unstable angina	Cardiovascular	0.64	0.57	0.70	< 0.001	Platinum agents
Venous thromboembolic disease	Cardiovascular	0.57	0.51	0.64	< 0.001	Platinum agents
Ventricular tachycardia	Cardiovascular	0.81	0.64	1.00	0.058	Platinum agents
Diabetes	Endocrine	0.91	0.85	0.97	0.003	Platinum agents
Diabetic neurological complications	Endocrine	0.86	0.80	0.92	< 0.001	Platinum agents
Diabetic ophthalmic complications	Endocrine	0.85	0.79	0.91	< 0.001	Platinum agents
Hyperparathyroidism	Endocrine	0.90	0.60	1.31	0.603	Platinum agents
Hypo or hyperthyroidism	Endocrine	0.75	0.67	0.85	< 0.001	Platinum agents
Alcoholic liver disease	Gastrointestinal	0.56	0.44	0.71	< 0.001	Platinum agents
Angiodysplasia of colon	Gastrointestinal	0.68	0.47	0.96	0.034	Platinum agents
Barrett's oesophagus	Gastrointestinal	0.92	0.83	1.02	0.128	Platinum agents
Cholangitis	Gastrointestinal	1.02	0.92	1.12	0.739	Platinum agents
Cholecystitis	Gastrointestinal	0.91	0.82	1.01	0.077	Platinum agents
Cholelithiasis	Gastrointestinal	0.91	0.82	1.01	0.08	Platinum agents
Coeliac disease	Gastrointestinal	1.11	0.89	1.36	0.354	Platinum agents
Crohn's disease	Gastrointestinal	0.80	0.55	1.13	0.219	Platinum agents
Diverticular disease of intestine	Gastrointestinal	1.06	0.99	1.14	0.096	Platinum agents
Fatty liver	Gastrointestinal	0.99	0.88	1.10	0.811	Platinum agents
Gastritis and duodenitis	Gastrointestinal	0.88	0.82	0.95	0.001	Platinum agents
Gastrooesophageal reflux disease	Gastrointestinal	1.06	0.98	1.14	0.152	Platinum agents
Hepatic failure	Gastrointestinal	0.93	0.82	1.04	0.215	Platinum agents
Liver fibrosis sclerosis and cirrhosis	Gastrointestinal	0.43	0.33	0.55	< 0.001	Platinum agents
Oesophageal varices	Gastrointestinal	0.38	0.23	0.58	< 0.001	Platinum agents
Oesophagitis and oesophageal ulcer	Gastrointestinal	0.95	0.88	1.01	0.101	Platinum agents
Portal hypertension	Gastrointestinal	1.07	0.94	1.22	0.283	Platinum agents
Ulcerative colitis	Gastrointestinal	0.96	0.69	1.31	0.818	Platinum agents
Agranulocytosis	Haematological	0.87	0.62	1.19	0.392	Platinum agents
Aplastic anaemias	Haematological	0.53	0.28	0.90	0.029	Platinum agents
Other anaemias	Haematological	0.61	0.57	0.66	< 0.001	Platinum agents
Primary or idiopathic thrombocytopaenia	Haematological	0.52	0.36	0.73	< 0.001	Platinum agents
Secondary or other thrombocytopaenia	Haematological	0.56	0.42	0.73	< 0.001	Platinum agents
Secondary polycythaemia	Haematological	0.61	0.39	0.92	0.023	Platinum agents
Thrombophilia	Haematological	0.65	0.41	0.96	0.042	Platinum agents
Autoimmune liver disease	Immunology and infection	0.55	0.41	0.74	< 0.001	Platinum agents
Chronic viral hepatitis	Immunology and infection	1.17	0.97	1.40	0.091	Platinum agents
Encephalitis	Immunology and infection	0.80	0.50	1.23	0.341	Platinum agents
HIV	Immunology and infection	0.81	0.57	1.14	0.251	Platinum agents
Infection of anal and rectal regions	Immunology and infection	0.66	0.41	1.01	0.071	Platinum agents
Infection of bones and joints	Immunology and infection	0.66	0.53	0.81	< 0.001	Platinum agents
Infection of liver	Immunology and infection	0.64	0.45	0.88	0.008	Platinum agents
Infection of skin and subcutaneous tissues	Immunology and infection	0.73	0.65	0.81	< 0.001	Platinum agents
Infections of other or unspecified organs	Immunology and infection	0.65	0.59	0.70	< 0.001	Platinum agents
Infections of the digestive system	Immunology and infection	1.08	1.00	1.16	0.046	Platinum agents
Infections of the heart	Immunology and infection	0.57	0.47	0.69	< 0.001	Platinum agents
Lower respiratory tract infections	Immunology and infection	0.69	0.65	0.73	< 0.001	Platinum agents
Lupus erythematosus (local and systemic)	Immunology and infection	0.79	0.43	1.36	0.423	Platinum agents
Meningitis	Immunology and infection	0.67	0.55	0.80	< 0.001	Platinum agents
Non-acute cystitis	Immunology and infection	0.71	0.54	0.92	0.011	Platinum agents
Peritonitis	Immunology and infection	1.02	0.96	1.08	0.481	Platinum agents
Viral diseases (excluding chronic hepatitis/HIV)	Immunology and infection	0.71	0.59	0.85	< 0.001	Platinum agents
Ankylosing spondylitis	Musculoskeletal	1.02	0.52	1.81	0.962	Platinum agents
Enteropathic arthropathy	Musculoskeletal	0.92	0.60	1.35	0.674	Platinum agents
Enthesopathies and synovial disorders	Musculoskeletal	1.09	1.01	1.17	0.02	Platinum agents
Osteoarthritis (excluding spine)	Musculoskeletal	0.99	0.93	1.06	0.804	Platinum agents
Osteoporosis	Musculoskeletal	0.68	0.60	0.76	< 0.001	Platinum agents
Rheumatoid arthritis	Musculoskeletal	0.72	0.59	0.87	0.001	Platinum agents
Scoliosis	Musculoskeletal	0.51	0.33	0.75	0.001	Platinum agents
Spinal stenosis	Musculoskeletal	0.93	0.77	1.11	0.45	Platinum agents
Bell's palsy	Neurological	0.55	0.36	0.81	0.004	Platinum agents
Dementia	Neurological	0.15	0.11	0.21	< 0.001	Platinum agents
Disorders of autonomic nervous system	Neurological	0.83	0.64	1.04	0.116	Platinum agents
Epilepsy	Neurological	0.69	0.54	0.88	0.004	Platinum agents
Essential tremor	Neurological	1.14	0.82	1.55	0.419	Platinum agents
Intracerebral haemorrhage	Neurological	0.38	0.28	0.50	< 0.001	Platinum agents
Intracranial hypertension	Neurological	0.62	0.45	0.84	0.003	Platinum agents
Peripheral neuropathies	Neurological	0.81	0.72	0.92	0.001	Platinum agents
Subarachnoid haemorrhage	Neurological	0.41	0.30	0.55	< 0.001	Platinum agents
Asthma	Pulmonary	0.77	0.70	0.85	< 0.001	Platinum agents
Bronchiectasis	Pulmonary	0.82	0.65	1.01	0.073	Platinum agents
COPD	Pulmonary	0.88	0.82	0.93	< 0.001	Platinum agents
Other interstitial pulmonary diseases with fibrosis	Pulmonary	0.59	0.46	0.75	< 0.001	Platinum agents
End stage renal disease	Renal	0.62	0.56	0.69	< 0.001	Platinum agents
Glomerulonephritis	Renal	0.48	0.37	0.63	< 0.001	Platinum agents
Obstructive and reflux uropathy	Renal	0.89	0.76	1.03	0.122	Platinum agents
Urolithiasis	Renal	0.89	0.79	1.01	0.065	Platinum agents
Abdominal aortic aneurysm	Cardiovascular	1.21	0.98	1.47	0.071	Vinca alkaloids
Atrial fibrillation	Cardiovascular	0.78	0.67	0.90	0.001	Vinca alkaloids
Atrioventricular block, complete	Cardiovascular	0.85	0.62	1.14	0.301	Vinca alkaloids
Atrioventricular block, first degree	Cardiovascular	0.85	0.62	1.14	0.301	Vinca alkaloids
Atrioventricular block, second degree	Cardiovascular	0.85	0.62	1.14	0.301	Vinca alkaloids
Bifascicular block	Cardiovascular	0.94	0.66	1.30	0.719	Vinca alkaloids
Coronary heart disease not otherwise specified	Cardiovascular	0.77	0.67	0.87	< 0.001	Vinca alkaloids
Heart failure	Cardiovascular	0.69	0.58	0.81	< 0.001	Vinca alkaloids
Hypertension	Cardiovascular	0.89	0.81	0.98	0.021	Vinca alkaloids
Ischaemic stroke	Cardiovascular	0.54	0.39	0.73	< 0.001	Vinca alkaloids
Left bundle branch block	Cardiovascular	0.85	0.62	1.14	0.305	Vinca alkaloids
Multiple valve disease	Cardiovascular	0.74	0.50	1.05	0.106	Vinca alkaloids
Myocardial infarction	Cardiovascular	0.83	0.71	0.96	0.012	Vinca alkaloids
Nonrheumatic aortic valve disorders	Cardiovascular	0.80	0.58	1.09	0.181	Vinca alkaloids
Nonrheumatic mitral valve disorders	Cardiovascular	0.88	0.60	1.26	0.503	Vinca alkaloids
Other cardiomyopathy	Cardiovascular	0.78	0.68	0.90	0.001	Vinca alkaloids
Pericardial effusion (noninflammatory)	Cardiovascular	0.87	0.57	1.27	0.49	Vinca alkaloids
Peripheral arterial disease	Cardiovascular	0.64	0.50	0.81	< 0.001	Vinca alkaloids
Raynaud's syndrome	Cardiovascular	0.64	0.49	0.83	0.001	Vinca alkaloids
Right bundle branch block	Cardiovascular	0.94	0.66	1.31	0.744	Vinca alkaloids
Sick sinus syndrome	Cardiovascular	0.74	0.45	1.16	0.222	Vinca alkaloids
Stable angina	Cardiovascular	0.87	0.73	1.03	0.115	Vinca alkaloids
Stroke (not otherwise specified)	Cardiovascular	0.57	0.43	0.74	< 0.001	Vinca alkaloids
Supraventricular tachycardia	Cardiovascular	0.64	0.39	0.98	0.055	Vinca alkaloids
Transient ischaemic attack	Cardiovascular	0.93	0.77	1.11	0.428	Vinca alkaloids
Trifascicular block	Cardiovascular	0.85	0.57	1.21	0.381	Vinca alkaloids
Unstable angina	Cardiovascular	0.74	0.62	0.88	0.001	Vinca alkaloids
Venous thromboembolic disease	Cardiovascular	0.60	0.49	0.73	< 0.001	Vinca alkaloids
Ventricular tachycardia	Cardiovascular	0.64	0.39	0.98	0.055	Vinca alkaloids
Diabetes	Endocrine	0.93	0.82	1.04	0.21	Vinca alkaloids
Diabetic neurological complications	Endocrine	0.86	0.75	0.99	0.035	Vinca alkaloids
Diabetic ophthalmic complications	Endocrine	0.85	0.75	0.97	0.018	Vinca alkaloids
Hypo or hyperthyroidism	Endocrine	0.94	0.77	1.14	0.542	Vinca alkaloids
Alcoholic liver disease	Gastrointestinal	1.06	0.69	1.58	0.766	Vinca alkaloids
Angiodysplasia of colon	Gastrointestinal	1.07	0.58	1.81	0.828	Vinca alkaloids
Barrett's oesophagus	Gastrointestinal	1.06	0.83	1.32	0.648	Vinca alkaloids
Cholangitis	Gastrointestinal	0.90	0.73	1.10	0.301	Vinca alkaloids
Cholecystitis	Gastrointestinal	0.88	0.71	1.08	0.245	Vinca alkaloids
Cholelithiasis	Gastrointestinal	0.82	0.66	1.00	0.058	Vinca alkaloids
Coeliac disease	Gastrointestinal	0.80	0.50	1.23	0.331	Vinca alkaloids
Diverticular disease of intestine	Gastrointestinal	1.13	0.97	1.30	0.105	Vinca alkaloids
Fatty liver	Gastrointestinal	1.07	0.85	1.34	0.55	Vinca alkaloids
Gastritis and duodenitis	Gastrointestinal	0.96	0.83	1.10	0.577	Vinca alkaloids
Gastrooesophageal reflux disease	Gastrointestinal	1.06	0.91	1.23	0.439	Vinca alkaloids
Hepatic failure	Gastrointestinal	1.02	0.80	1.28	0.872	Vinca alkaloids
Liver fibrosis sclerosis and cirrhosis	Gastrointestinal	0.61	0.35	0.99	0.06	Vinca alkaloids
Oesophagitis and oesophageal ulcer	Gastrointestinal	0.99	0.86	1.13	0.866	Vinca alkaloids
Portal hypertension	Gastrointestinal	1.07	0.81	1.38	0.623	Vinca alkaloids
Agranulocytosis	Haematological	1.30	1.01	1.65	0.034	Vinca alkaloids
Aplastic anaemias	Haematological	0.85	0.58	1.22	0.403	Vinca alkaloids
Other anaemias	Haematological	0.80	0.70	0.91	0.001	Vinca alkaloids
Other haemolytic anaemias	Haematological	0.69	0.38	1.15	0.174	Vinca alkaloids
Primary or idiopathic thrombocytopaenia	Haematological	0.82	0.58	1.14	0.254	Vinca alkaloids
Secondary or other thrombocytopaenia	Haematological	0.81	0.60	1.07	0.153	Vinca alkaloids
Secondary polycythaemia	Haematological	0.61	0.30	1.11	0.136	Vinca alkaloids
Autoimmune liver disease	Immunology and infection	0.68	0.36	1.18	0.2	Vinca alkaloids
Chronic viral hepatitis	Immunology and infection	1.05	0.73	1.48	0.773	Vinca alkaloids
Encephalitis	Immunology and infection	1.09	0.61	1.81	0.768	Vinca alkaloids
HIV	Immunology and infection	0.85	0.53	1.32	0.489	Vinca alkaloids
Infection of bones and joints	Immunology and infection	0.71	0.49	1.01	0.071	Vinca alkaloids
Infection of liver	Immunology and infection	0.71	0.36	1.25	0.266	Vinca alkaloids
Infection of skin and subcutaneous tissues	Immunology and infection	0.76	0.63	0.91	0.004	Vinca alkaloids
Infections of other or unspecified organs	Immunology and infection	0.78	0.67	0.89	< 0.001	Vinca alkaloids
Infections of the digestive system	Immunology and infection	1.09	0.94	1.27	0.249	Vinca alkaloids
Infections of the heart	Immunology and infection	0.71	0.51	0.97	0.037	Vinca alkaloids
Lower respiratory tract infections	Immunology and infection	0.75	0.68	0.83	< 0.001	Vinca alkaloids
Meningitis	Immunology and infection	0.82	0.60	1.08	0.176	Vinca alkaloids
Non-acute cystitis	Immunology and infection	0.89	0.52	1.42	0.641	Vinca alkaloids
Peritonitis	Immunology and infection	1.08	0.96	1.21	0.197	Vinca alkaloids
Viral diseases (excluding chronic hepatitis/HIV)	Immunology and infection	0.93	0.72	1.19	0.562	Vinca alkaloids
Enthesopathies and synovial disorders	Musculoskeletal	1.06	0.93	1.20	0.412	Vinca alkaloids
Osteoarthritis (excluding spine)	Musculoskeletal	1.00	0.89	1.13	0.961	Vinca alkaloids
Osteoporosis	Musculoskeletal	0.92	0.75	1.12	0.44	Vinca alkaloids
Rheumatoid arthritis	Musculoskeletal	0.98	0.73	1.29	0.883	Vinca alkaloids
Spinal stenosis	Musculoskeletal	1.23	0.92	1.62	0.149	Vinca alkaloids
Bell's palsy	Neurological	0.99	0.60	1.55	0.971	Vinca alkaloids
Dementia	Neurological	0.37	0.24	0.53	< 0.001	Vinca alkaloids
Disorders of autonomic nervous system	Neurological	1.12	0.79	1.54	0.5	Vinca alkaloids
Epilepsy	Neurological	0.91	0.66	1.22	0.531	Vinca alkaloids
Essential tremor	Neurological	1.37	0.77	2.25	0.25	Vinca alkaloids
Intracerebral haemorrhage	Neurological	0.36	0.20	0.60	< 0.001	Vinca alkaloids
Intracranial hypertension	Neurological	0.95	0.70	1.28	0.749	Vinca alkaloids
Peripheral neuropathies	Neurological	0.83	0.66	1.02	0.087	Vinca alkaloids
Subarachnoid haemorrhage	Neurological	0.36	0.18	0.62	0.001	Vinca alkaloids
Asthma	Pulmonary	0.87	0.73	1.03	0.119	Vinca alkaloids
Bronchiectasis	Pulmonary	0.83	0.56	1.18	0.324	Vinca alkaloids
COPD	Pulmonary	0.92	0.81	1.03	0.166	Vinca alkaloids
Other interstitial pulmonary diseases with fibrosis	Pulmonary	0.80	0.54	1.14	0.243	Vinca alkaloids
End stage renal disease	Renal	0.82	0.69	0.98	0.029	Vinca alkaloids
Glomerulonephritis	Renal	0.64	0.40	0.96	0.041	Vinca alkaloids
Obstructive and reflux uropathy	Renal	1.21	0.93	1.55	0.143	Vinca alkaloids
Urolithiasis	Renal	1.12	0.90	1.37	0.305	Vinca alkaloids
